# Electrochemical synthesis of heterodehydro[7]helicenes

**DOI:** 10.1038/s42004-022-00780-7

**Published:** 2022-12-03

**Authors:** Md. Imrul Khalid, Mohamed S. H. Salem, Makoto Sako, Masaru Kondo, Hiroaki Sasai, Shinobu Takizawa

**Affiliations:** 1 SANKEN, Osaka University, Mihogaoka, Ibaraki-shi, Osaka, 567-0047 Japan; 2 Pharmaceutical Organic Chemistry Department, Faculty of Pharmacy, Suez Canal University, Ismailia, 41522 Egypt; 3 Graduate School of Pharmaceutical Sciences, Osaka University, Yamada-oka, Suita-shi, Osaka, 565-0871 Japan; 4 Department of Materials Science and Engineering, Graduate School of Science and Engineering, Ibaraki University, Naka-narusawa, Hitachi, Ibaraki, 316-8511 Japan

**Keywords:** Electrochemistry, Materials for optics, Asymmetric catalysis

## Abstract

Dehydrohelicenes are some of the most attractive chiroptical materials with unique helical chirality. However, to our knowledge, there are no prior reports on their direct construction by asymmetric methods. In this work, sequential synthesis of aza-oxa-dehydro[7]helicenes *via* the electrochemical oxidative hetero-coupling of 3-hydoxycarbazoles and 2-naphthols followed by dehydrative cyclization and intramolecular C–C bond formation has been realized. In addition, an efficient enantioselective synthesis through chiral vanadium-catalyzed hetero-coupling and electrochemical oxidative transformations afforded heterodehydro[7]helicene without any racemization. The obtained dehydro[7]helicenes showed intense blue-colored circularly polarized luminescence (|*g*_*lum*_| ≈ 2.5 × 10^−3^ at 433 nm). Thermodynamic and kinetic studies of the racemization barrier of heterodehydro[7]helicenes indicated significant chiral stability with ΔG^‡^> 140 kJ mol^−1^.

## Abstract (web summary)

Heterodehydrohelicenes exhibit attractive chiroptical properties, but their direct construction by asymmetric synthesis remains very challenging. Here, the authors report the electrochemical sequential synthesis of azaoxadehydro[7]helicenes from arenols through oxidative hetero-coupling followed by dehydrative cyclization and intramolecular C–C bond formation.

## Introduction

Heterodehydrohelicenes, also known as quasi-heterocirculenes with helical chirality, can be classified as polycyclic heteroaromatics (PHAs)^1^ in which the two helical termini of a helicene are connected by a sigma bond^2–10^. Their unique helical chirality has led to extraordinary chiroptical responses^11^ which can be implemented in various material-based applications, such as organic light-emitting diodes (OLEDs) and field-effect transistors (FETs). Moreover, the superior chiroptical properties of heterodehydrohelicenes such as circular dichroism (CD) and circularly polarized luminescence (CPL) open the gate for various applications in optical information storage and transfer, in such cases the level of CPL can promote a further dimension to the information content transported through light^12^. In 1969, Zander and Franke first reported a aza-dehydro[6]helicene synthesized from the corresponding aza[6]helicene as a precursor through metal(Al)-mediated terminal ring closure^2^. Similar protocols have been subsequently applied to synthesize thiophene-based dehydro[5]^3^, [6] (using Al)^4^, and [7]helicenes (using Pd and Sn)^5^. Tanaka and Osuka have synthesized aza-dehydro[7]helicene derivatives with three pyrrole rings using bis(trifluoroacetoxy)iodobenzene (PIFA); these molecules exhibit interesting photophysical properties^6^. However, these heterodehydrohelicenes were difficult to isolate as optically pure forms due to their low racemization barriers^2–6^.

**Fig. 1:**
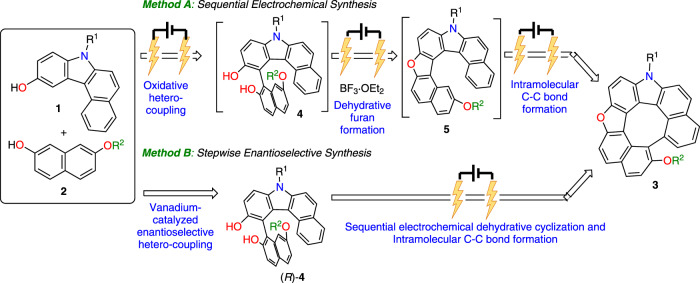
Sequential electrochemical synthesis of aza-oxa-dehydro[7]helicenes (this work). Two methods for synthesizing dehydro[7]helicenes **3**: (**A**) electrochemical sequential synthesis through the oxidative hetero-coupling of **1** and **2** followed by dehydrative cyclization and intramolecular C-C bond formation; (**B**) stepwise enantioselective synthesis through chiral vanadium-catalyzed hetero-coupling and electrochemical oxidative transformations.

In 2017, Itami and Segawa first reported thia-dehydro[6]helicenes as saddle-helix molecules with *tert*-butyl substitutions at the terminal aromatic rings that prevent racemization; HPLC with a chiral stationary phase could be used for enantiomeric separation^7^. Tanaka, Osuka^8^, and Pittelkow^9^ have also reported heterodehydro[7]helicene modifications that lead to significantly high racemization barriers. In 2021, Maeda and Ema reported an aza-dehydro[7]helicene with intense CPL and Cotton effects^10^. Despite the immense potential exhibited by heterodehydrohelicenes, to our knowledge, there are no reports on their straightforward construction including asymmetric synthesis. This could be due to the limitations associated with the synthetic steps: low total yields, harsh reaction conditions (such as high temperature), easy racemization, and/or overuse of oxidants (narrow functional group tolerance). In 2016, we reported an efficient vanadium-catalyzed synthesis of oxa[9]helicenes^13^ *via* the oxidative coupling of arenol compounds^14^ followed by intramolecular dehydrative cyclization. Recently we also developed the catalytic enantioselective oxidative hetero-coupling of arenols using a chiral vanadium(V) complex^15^. Based on these radical-anion coupling mechanism, we envisioned applying an organic electrochemical method to develop more environmentally benign syntheses of heterodehydrohelicenes. Electrochemical syntheses have many advantages, since no oxidant is required and oxidative transformation can be conducted under mild reaction conditions^16^. Indeed, many metal- and oxidant-free electrochemical oxidative coupling reactions of arenols have been reported^17^. Also, some reports describing a cascade electrochemical approach for synthesizing PHAs.^18^ However, to our knowledge, there have been no reports on heterodehydrohelicene syntheses using arenol as a starting material under electrochemical conditions. Herein, we describe two efficient methods for synthesizing aza-oxa-dehydro[7]helicenes **3** possessing multiple heteroaromatic rings; the electrochemical sequential synthesis of **3** through the oxidative hetero-coupling of readily available arenols **1** and **2** followed by dehydrative cyclization and intramolecular C-C bond formation, and the enantioselective approach through chiral vanadium-catalyzed hetero-coupling and electrochemical oxidative transformations (Fig. 1).

## Results and discussion

### Screening of reaction conditions and evaluation of substrate scope

After examining various conditions and performing sedulous optimization (see Tables S1–S4 in Supplementary Method 3), a sequential protocol generating aza-oxa-dehydro[7]helicenes **3aa** in 84% yield (81% isolated yield, current efficiency = 30%) was developed; hydroxybenzo[*c*]carbazole **1a** and 7-methoxy-2-naphthol **2a** were utilized as model substrates based on CV studies (see Fig. S18 in Supplementary Note 6), with fluorine-doped tin oxide (FTO)^19^ electrodes and Bu_4_NPF_6_ as the electrolyte (0.1 M) at 25 °C, in the presence of BF_3_·OEt_2_ (as an additive) in CH_2_Cl_2_ and no homocoupling products were observed (Table 1, entry 1)^20^. The structure of **3aa** was confirmed by X-ray crystallography (see Supplementary Note 7). Other solvents [THF, MeCN, and MeOH (5 mL)] formed diol **4aa** as the major side product in 9 to 22% yields (entries 2–4). The FTO electrodes showed high conversion of substrates (entry 1), while changing any one of the electrodes resulted in reduced conversion yields (entries 5–6). Doubling the current density reduced the yield of **3aa** to 65% (entry 7); its reduction to 0.8 mA/cm^2^ did not significantly affect the yield but prolonged the reaction time (entry 8). High concentrations of the electrolyte, and other electrolytes such as LiClO_4_ (0.1 M) formed diol **4aa** as the major side product (entries 9–10). Decreasing the amount of BF_3_·OEt_2_ or replacing it with other Brønsted acids (as acidic additives) produced low yields of aza-oxa-dehydro[7]helicene **3aa** (**4aa** was isolated as the major side product in 5 to 20% yields) (entries 11–13). BF_3_·OEt_2_ transformed diol **4aa** to helicene intermediate **5aa**, which underwent facile anodic oxidation to form **3aa** (see Scheme S1 in Supplementary Note 3). No reaction occurred in the absence of electricity (entry 14). Finally, to generalize this protocol, **3aa** was synthesized using ElectraSyn® 2.0 (designed by IKA)^21^; it exhibited comparable results (entry 15).

**Table 1:** Optimization of reaction conditions for the electrochemical synthesis of heterodehydrohelicene **3aa**^a^.


Entry	Solvent (X M)	Variation	% Yield
1	CH_2_Cl_2_ (0.02 M)	None	84 (81)^[d]^
2	THF (0.02 M)	Solvent	21
3	MeCN (0.02 M)	Solvent	36
4	MeOH (0.02 M)	Solvent	45
5	CH_2_Cl_2_ (0.02 M)	With C( + ) / FTO(-)	42
6	CH_2_Cl_2_ (0.02 M)	With FTO( + ) / Pt(-)	55
7	CH_2_Cl_2_ (0.02 M)	*J* = 2.4 mA/cm^2^	65
8	CH_2_Cl_2_ (0.02 M)	*J* = 0.8 mA/cm^2^	77^[b]^
9	CH_2_Cl_2_ (0.02 M)	0.2 M of Bu_4_NPF_6_	65
10	CH_2_Cl_2_ (0.02 M)	LiClO_4_ instead of Bu_4_NPF_6_	47
11	CH_2_Cl_2_ (0.02 M)	Without BF_3_·OEt_2_	37
12	CH_2_Cl_2_ (0.02 M)	Using 0.1 M of BF_3_·OEt_2_	68
13	CH_2_Cl_2_ (0.02 M)	Using TFA instead of BF_3_·OEt_2_	67
14	CH_2_Cl_2_ (0.02 M)	No electricity	No reaction^[c]^
15	CH_2_Cl_2_ (0.02 M)	ElectraSyn® 2.0 with Pt(+) / Pt(-)	75

[a] Electrolysis conditions: FTO anode, FTO cathode, constant current = 3 mA (*J* = 1.2 mA/cm^2^), **1a** and **2a** (0.1 mmol), Bu_4_NPF_6_ (0.1 M), BF_3_·OEt_2_ (0.2 M), CH_2_Cl_2_ (5 mL), 25 °C, 10 h. [b] 12.5 h. [c] No conversion. [d] Isolated yield.

**Fig. 2:**
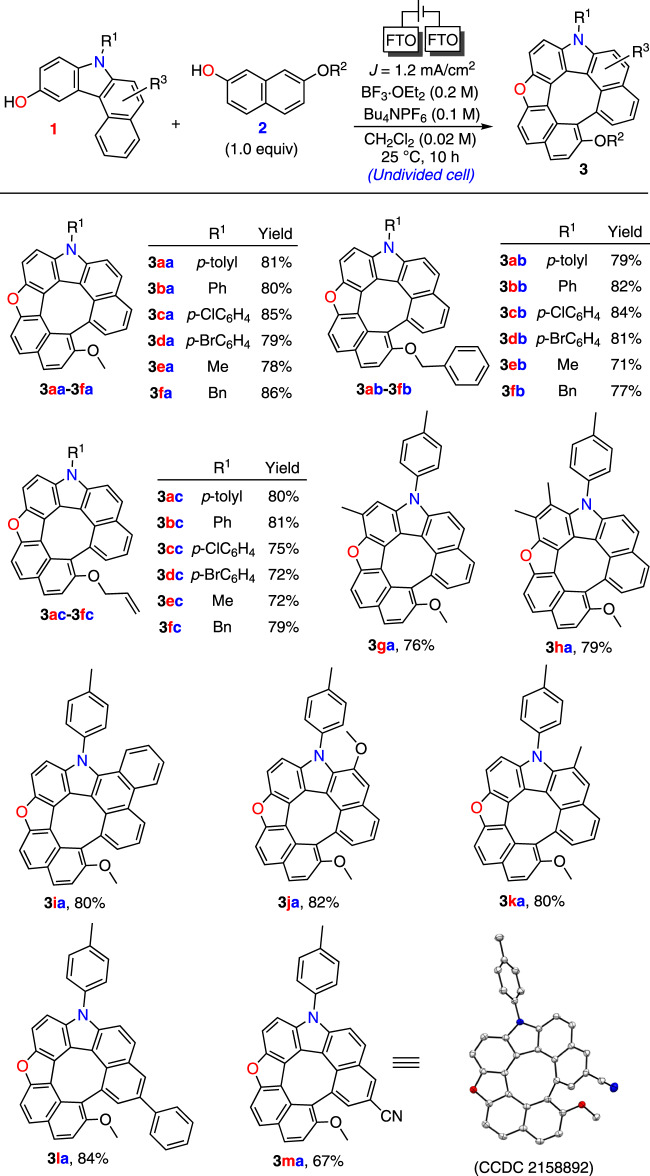
Substrate-scope studies. Electrolysis conditions: FTO anode, FTO cathode, constant current = 3 mA (*J* = 1.2 mA/cm^2^), **1** and **2** (0.1 mmol), Bu_4_NPF_6_ (0.1 M), BF_3_·OEt_2_ (0.2 M), CH_2_Cl_2_ (5 mL), 25 °C, 10 h.

Subsequently, the substrate scope of various hydroxycarbazoles **1** and 2-naphthols **2** were investigated under the optimal reaction conditions (Fig. 2). *N*-Aryl- and *N*-alkyl-substituted derivatives **1a**–**1f** underwent facile conversion to aza-oxa-dehydro[7]helicenes **3aa**–**3fa** in 78–86% yields. Different combinations of **2** with 7-benzyloxy or allyloxy groups **2b**–**2c** were used to synthesize aza-oxa-dehydro[7]helicenes **3ab**–**3fc** in 71–84% yields. A series of compounds **1** substituted by Me, Ph, and OMe groups at various positions on the aromatic ring formed products **3ga**–**3la** in 76–84% yields. π-Expanded substrate **1i** also reacted with **2a** to form **3ia** in 80% yield. Hydroxycarbazole **1m** with an electron-withdrawing cyano group afforded **3ma** in 67% yield.

### Two-pot synthesis and transformation of product

**Fig. 3:**
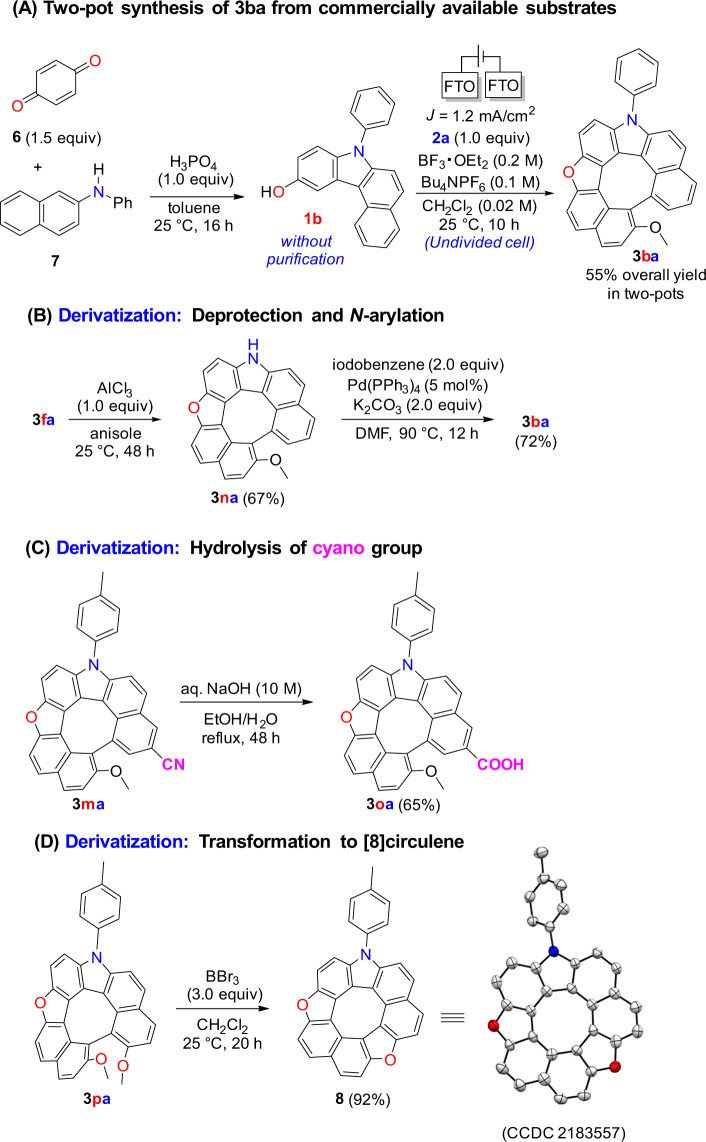
Two-pot synthesis and derivatization of aza-oxa-dehydro[7]helicenes. **A** Synthesis of dehydrohelicene **3ba** from commercially available substrates. **B** AlCl_3_- medicated deprotection of Bn- group followed by Pd-catalyzed *N*-arylation. **C** Basic hydrolysis of cyano group to afford the corresponding carboxylic acid. **D** Demethylation of dehydrohelicene **3pa** to give the corresponding circulene **8**.

To establish the applicability of this method for concise synthesis, a two-pot protocol using commercially available substrates *p*-benzoquinone (**6**) and *N*-phenyl-2-naphthylamine (**7**) was tested; it produced aza-oxa-dehydro[7]helicene **3ba** in 55% overall yield (Fig. 3A).

Transformations of aza-oxa-dehydro[7]helicenes **3** were also investigated (see Supplementary Method 6). The benzyl group on the nitrogen of **3fa** was removed using AlCl_3_ to give **3na**, which underwent Pd-catalyzed *N*-arylation to form **3ba** (Fig. 3B). The carboxylic acid derivative **3oa** was formed after the hydrolysis of **3ma** under basic conditions (Fig. 3C). Furthermore, a treatment of **3pa** with BBr_3_ at 25 °C afforded the corresponding aza-dioxa[8]circulene **8** in 92% yield (Fig. 3D).

### Circular dichroism (CD) and circularly polarized luminescence (CPL) analysis of aza-oxa- dehydro[7]helicenes

**Fig. 4:**
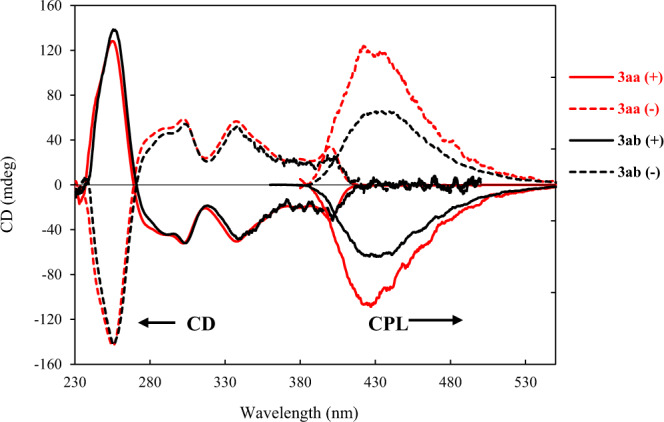
CD and CPL spectra of aza-oxa-dehydro[7]helicenes (3aa and 3ab). Chiroptical properties of **3aa** and **3ab** (CD and CPL) were studied in CHCl_3_ (2 × 10^−5^ M). **3aa** showed high CPL activity with *g*_*lum*_ = 2.5 × 10^−3^ at 433 nm and **3ab** showed *g*_*lum*_ = 2.4 × 10^−3^ at 418 nm.

HPLC using a chiral stationary phase (CHIRALPAK IC, hexane/*i*-PrOH=30:1), was used for the optical resolution of **3aa**, **3ca** and **3fa** to analyze the racemization barriers of the obtained aza- oxa-dehydro[7]helicenes, (see Supplementary Note 2). The optical rotation values of optically pure **3aa** first peak (*M*)-**3aa**: [α]_D_^24^ = –1464 (*c* = 0.0175 g/mL), second peak (*P*)-**3aa**: [α]_D_^24^ = +1462 (*c* = 0.0173 g/mL). The absolute configuration of (−)-**3aa** was determined as *M* through X-ray crystallographic analysis after recrystallization of the first peak of enantiomer **3aa**. Eyring plots indicated the significant chiral stability of aza-oxa-dehydro[7]helicenes **3** (racemization barrier >140 kJ mol^−1^) (see Fig. S2–S10 in Supplementary Note 2); the t_1/2_ of compound **3aa** was estimated to be greater than 9.5 × 10^3^ years at 25 °C. Aza-oxa-dehydro[7]helicene **3aa** showed higher chiral stability than that of corresponding aza-oxa[7]helicene **5aa** (110 kJ mol^−1^). Subsequently, the chiroptical properties of the optically pure aza-oxa-dehydro[7]helicenes were investigated. All the helical dyes **3** showed absorption in the wavelength range of 340–404 nm and fluorescence maximum at 450 nm (see Figs. S11-S13 in Supplementary Note 4); CD and CPL^11^ signals were observed in these regions (see Figs. S14-S17 in Supplementary Note 5). Among synthesized aza-oxa-dehydro[7]helicenes, **3aa** showed moderate quantum yield Φ = 0.25, and significant CPL activity with *g*_lum_ = 2.5 × 10^−3^ at 433 nm (Fig. 4).

### An efficient enantioselective synthesis of heterodehydro[7]helicenes *via* chiral vanadium complex catalyzed hetero-coupling and electrochemical oxidative transformations

**Fig. 5:**
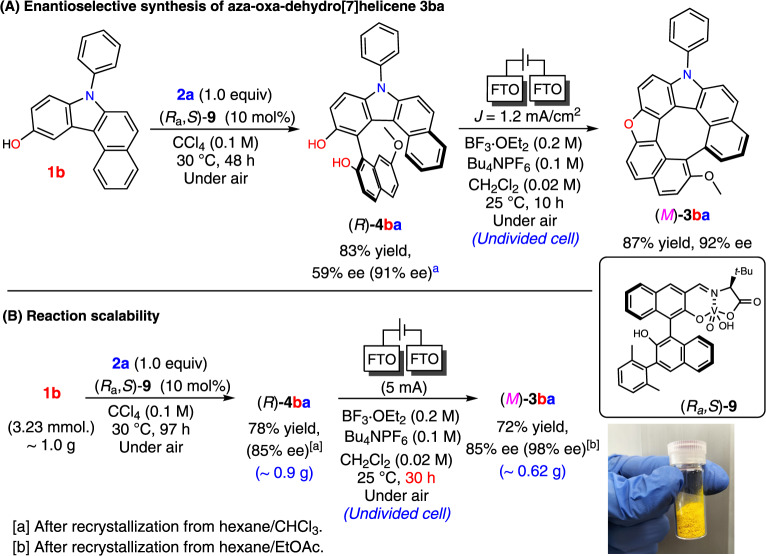
Stepwise enantioselective synthesis of aza-oxa-dehydro[7]helicene 3ba, and verification of the reaction scalability. **A** Stepwise enantioselective synthesis of dehydrohelicene (*M*)-**3ba** using hybrid vanadium and electrochemistry. **B** Scaling up the enantioselective synthesis to semi-gram scale.

Although many reports are found on the enantioselective synthesis of helicenes^22–26^, to our knowledge, there are no such reports for heterodehydrohelicenes. An efficient chemo- and enantioselective protocol for the oxidative hetero-coupling of hydroxycarbazoles and naphthols using a chiral vanadium(v) complex, producing axially chiral biaryl derivatives, has been previously reported^15^; based on this study, a stepwise enantioselective synthesis of diol **4** was examined, which was followed by their electrochemical transformation to the corresponding heterodehydrohelicenes (see Supplementary Method 7). Diol (*R*)-**4ba** was synthesized with a good optical purity using mononuclear vanadium complex (*R*_a_*,S*)-**9**. Under electrooxidation conditions, (*R*)-**4ba** underwent a sequential dehydrative furan ring formation followed by the coupling of the two helical termini to afford the corresponding aza-oxa-dehydro[7]helicene, (*M*)-**3ba** (see Supplementary Note 1), in 87% yield without any loss in optical purity (Fig. 5A). Notably, the protocol was scaled up, as depicted in Fig. 5B, to form 0.62 g of (*M*)-**3ba** (current efficiency = 48%).

In conclusion, an efficient synthetic method utilizing an electrochemical reaction under mild conditions was developed for aza-oxa-dehydro[7]helicenes **3**. The racemization barriers of the heterodehydrohelicenes synthesized in this study were significantly higher than those of the corresponding helicenes with sustained racemization half-lives. Furthermore, this paper reports the enantioselective synthesis of heterodehydrohelicenes for the first time, with a scaled-up reaction (to the gram-scale). Applications of the chiroptical properties are currently under investigation.

## Methods

### General methods

For synthetic details and analytical data of all reaction starting materials **1**, see Supplementary Methods 1 and 2. For synthetic and analytical details of all dehydro[7]helicenes **3**, see Supplementary method 4. For the general procedure of two-pot synthesis and derivatization of dehydro[7]helicene, see Supplementary Methods 5 and 6. Enantioselective synthesis and scaling-up details are highlighted in Supplementary Method 7. For NMR spectra seeSupplementary Data 1.

### General procedure for the sequential preparation of heterodehydrohelicenes 3

A solution of benzo[*c*]carbazol-10-ol derivatives **1** (0.1 mmol), 2-naphthols **2** (0.1 mmol), tetrabutylammonium hexafluorophosphate(V) (193.7 mg, 0.5 mmol) and BF_3_・EtO_2_ (0.2 M) in CH_2_Cl_2_ (5.0 mL) was transferred into an undivided electrolysis cell. This cell is equipped with two FTO electrodes (1.0^ × ^2.5 cm^2^), which are connected to DC power supply (see Fig. S1 in Supplementary Method 2). At rt, a constant current electrolysis with a current density of 1.20 mA/cm^2^ was applied. After stirring for 10 h, the electrolysis was stopped and purification of the crude products by column chromatography (SiO_2_, EtOAc/hexane) provided the desired heterodehydro[7]helicene **3**.

## Supplementary information

Takizawa_PR File

Supplementary information

Description of Additional Supplementary Files

Supplementary data 1

Supplementary data 2

Supplementary data 3

Supplementary data 4

Supplementary data 5

Supplementary data 6

## Data Availability

Additional data supporting the findings described in this manuscript are available in Supplementary Information and Supplementary Data 1. All CIFs are available in Supplementary Data 2-6. The X-ray crystallographic coordinate for structures reported in this study have been deposited at the Cambridge Crystallographic Data Center (CCDC) under deposition numbers CCDC-2091483 (**3aa**), CCDC-2128351 (**5aa**), CCDC-158892 (**3ma**), CCDC-2183557 (**8**), and CCDC 2057234 (**10aa**). These data can be obtained free of charge from The Cambridge Crystallographic Data Center *via* www.ccdc.cam.ac.uk/data_request/cif. The authors declare that all other data supporting the findings of this study are available within the article and Supplementary Information files, and also are available from the corresponding author upon reasonable request.

## References

[1] Borissov A (2022). Recent advances in heterocyclic nanographenes and other polycyclic heteroaromatic compounds. Chem. Rev..

[2] Zander M, Franke WH (1969). Über carbazolo-carbazole. Chem. Ber..

[3] Wynberg H, Groen MB, Schadenberg H (1971). Synthesis and resolution of some heterohelicenes. J. Org. Chem..

[4] Dopper JH, Oudman D, Wynberg H (1975). Dehydrogenation of heterohelicenes by a Scholl type reaction. dehydrohelicenes. J. Org. Chem..

[5] Rajca A (2009). Intramolecular cyclization of thiophene-based [7]helicenes to quasi- [8]circulenes. J. Org. Chem..

[6] Chen F, Tanaka T, Mori T, Osuka A (2018). Synthesis, structures, and optical properties of azahelicene derivatives and unexpected formation of azahepta[8]circulenes. Chem. Eur. J..

[7] Fujikawa T, Segawa Y, Itami K (2017). Laterally π-extended dithia[6]helicenes with heptagons: saddle-helix hybrid molecules. J. Org. Chem..

[8] Matsuo Y, Chen F, Kise K, Tanaka T, Osuka A (2019). Facile synthesis of fluorescent hetero[8]circulene analogues with tunable solubilities and optical properties. Chem. Sci..

[9] Lousen B (2020). Compressing a non‐planar aromatic heterocyclic [7]helicene to a planar hetero[8]circulene. Chem. Eur. J..

[10] Maeda C, Nomoto S, Akiyama K, Tanaka T, Ema T (2021). Facile synthesis of azahelicenes and diaza[8]circulenes through the intramolecular Scholl reaction. Chem. Eur. J..

[11] Mori T (2021). Chiroptical properties of symmetric double, triple, and multiple helicenes. Chem. Rev..

[12] Peeters E (1997). Circularly polarized electroluminescence from a polymer light-emitting diode. J. Am. Chem. Soc..

[13] Sako M (2016). Efficient enantioselective synthesis of oxahelicenes using redox/acid cooperative catalysts. J. Am. Chem. Soc..

[14] Sako M, Takizawa S, Sasai H (2020). Chiral vanadium complex-catalyzed oxidative coupling of arenols. Teterahedron.

[15] Sako M (2021). Chemo- and enantioselective hetero-coupling of hydroxycarbazoles catalyzed by a chiral vanadium(v) complex. Org. Chem. Front..

[16] Cheng X (2022). Recent applications of homogeneous catalysis in electrochemical organic synthesis. CCS Chem..

[17] Röckl JL, Pollok D, Franke F, Waldvogel SR (2020). A decade of electrochemical dehydrogenative C,C-coupling of aryls. Acc. Chem. Res..

[18] Zhang L, Hu X (2020). Nickel catalysis enables convergent paired electrolysis for direct arylation of benzylic C–H bonds. Chem. Sci..

[19] Hou ZW, Mao ZY, Song J, Xu HC (2017). Electrochemical synthesis of polycyclic *N*- heteroaromatics through cascade radical cyclization of diynes. ACS Catal..

[20] Kingston C (2020). A survival guide for the “electro-curious”. Acc. Chem. Res..

[21] https://www.ika.com/en/Products-Lab-Eq/Electrochemistry-Kit-csp-516/ElectraSyn-20- pro-Package-cpdt-40003261/

[22] Pelliccioli V (2022). Enantioselective synthesis of dithia[5]helicenes and their postsynthetic functionalization to access dithia[9]helicenes. Angew. Chem. Int. Ed..

[23] Wang Q, Zhang WW, Zheng C, Gu Q, You SL (2020). Enantioselective synthesis ofazoniahelicenes by Rh-catalyzed C–H annulation with alkynes. J. Am. Chem. Soc..

[24] Stará IG, Starý I (2020). Helically chiral aromatics: The synthesis of helicenes by [2+2+2] cycloisomerization of π-electron systems. Acc. Chem. Res.

[25] Yubuta A (2020). Enantioselective synthesis of triple helicenes by cross-cyclotrimerization of a helicenyl aryne and alkynes *via* dynamic kinetic resolution. J. Am. Chem. Soc..

[26] Dhbaibi K, Favereau L, Crassous J (2019). Enantioenriched helicenes and helicenoids containing main-group elements (B, Si, N, P). Chem. Rev..

