# Supplementary material for: Electrochemical synthesis of heterodehydro[7]helicenes
Source: Commun Chem. 2022 Dec 3;5:166. doi: 10.1038/s42004-022-00780-7 (PMC9814689; doi:10.1038/s42004-022-00780-7)
Supplement: Supplementary file 2 — Supplementary information [file 42004_2022_780_MOESM2_ESM.pdf]

Supporting Information

Electrochemical Synthesis of Heterodehydro[7]helicenes

Md. Imrul Khalid,†[1] Mohamed S. H. Salem,† [1,2] Makoto Sako,[3] Masaru Kondo,[4] Hiroaki Sasai*,[1,3] and Shinobu Takizawa*[1]

[1] SANKEN, Osaka University, Mihogaoka, Ibaraki-shi, Osaka 567-0047, Japan

[2] Pharmaceutical Organic Chemistry Department, Faculty of Pharmacy, Suez Canal University, Ismailia 41522, Egypt

[3] Graduate School of Pharmaceutical Sciences, Osaka University, Yamada-oka, Suita-shi, Osaka 565-0871, Japan

[4] Department of Materials Science and Engineering, Graduate School of Science and Engineering, Ibaraki University, Naka-narusawa, Hitachi, Ibaraki 316-8511, Japan

† These authors contributed equally to this work.

E-mail: sasai@sanken.osaka-u.ac.jp; taki@sanken.osaka-u.ac.jp

Tel: +81-6-6879-8467; Fax: +81-6-6879-8469

Table of Contents

1.	**Supplementary Method 1:** *general information*	S2
2.	**Supplementary Method 2:** *experimental procedures for the synthesis of **1***	S4
3.	**Supplementary Method 3:** *optimization of reaction conditions*	S13
4.	**Supplementary Method 4:** *general procedure for the sequential preparation of dehydro[7]helicenes **3***	S15
5.	**Supplementary Method 5:** *general procedure for the two-pot synthesis of dehydro[7]helicene **3ba***	S26
6.	**Supplementary Method 6:** *derivatization of dehydro[7]helicenes **3***	S27
7.	**Supplementary Method 7:** *stepwise enantioselective synthesis*	S29
8.	**Supplementary Note 1:** *determination of the absolute configuration of dehydro[7]helicenes 3**ba***	S35
9.	**Supplementary Note 2:** *racemization barrier of dehydro[7]helicenes **3***	S35
10.	**Supplementary Note 3:** *plausible reaction mechanism for the electrochemical sequential reaction*	S41
11.	**Supplementary Note 4:** *photophysical properties (UV and PL)*	S42
12.	**Supplementary Note 5:** *chiroptical properties (CD and CPL)*	S44
13.	**Supplementary Note 6:** *CV charts*	S46
14.	**Supplementary Note 7:** *X-ray crystallographic Analysis*	S47
15.	**Supplementary References**	S63

1. Supplementary Method 1: *general information*

^{1}H-, and ^{13}C-NMR spectra were recorded with JEOL JMN ECS400 FT NMR, JNM ECA600 FT NMR or Bruker AVANCE II (^{1}H-NMR 400, 600 or 700 MHz, ^{13}C-NMR 100, 150 or 175 MHz) ^{1}H-NMR spectra are reported as follows: the chemical shift in ppm downfield of tetramethylsilane (TMS) and referenced to residual solvent peak ($CDCl_3$) at 7.26 ppm, or ($(CD_3)_2CO$) at 2.05 ppm, integration, multiplicities (s = singlet, d = doublet, t = triplet, q = quartet, m = multiplet), and coupling constants (Hz). ^{13}C-NMR spectra were reported in ppm relative to the central line of triplet for $CDCl_3$ at 77.16 ppm, or the central line of septet for ($(CD_3)_2CO$) at 29.84 ppm. ESI-MS spectra were obtained with JMS-T100LC (JEOL). Optical rotations were measured with JASCO P-1030 polarimeter. HPLC analyses were performed on JASCO HPLC system (JASCO PU 980 pump and UV-975 UV/Vis detector) using a mixture of hexane and *i*-PrOH as eluents. FT-IR spectra were recorded on JASCO FT-IR system (FT/IR4100). Thin-layer chromatography (TLC) analysis of reaction mixtures was performed using Merck silica-gel 60 F254 TLC plates and visualized under UV Column chromatography on SiO_2 was performed with Kanto silica-gel 60 (63-210 μm). Melting points were measured with melting point apparatus MP-S9 (Yanaco, Japan) and were uncorrected. CD and CPL spectra were recorded with JASCO J-820AC and JASCO CPL-300 spectrometers. Cyclic voltammetry was carried out on BAS CV-620C voltammetric analyzer using a platinum disk as the working electrode, platinum wire as the counter electrode, and Ag/$AgNO_3$ as the reference electrode at a scan rate of 100 mVs^{-1}. Quantum yields were determined with a Hamamatsu C11347 spectrometer. Vanadium complexes were synthesized according to the literature.[1-3)] Coupling precursors **2a-c** were prepared according to the literature procedure.[4-6)] Commercially available organic and inorganic compounds were used without further purification. The electrochemical reactions were performed in a 10 mL reaction vessel equipped with two FTO electrodes (1.0 × 2.5 cm^2) connected to Cu wire (**Figure S1a** and **S1b**). The two electrodes are connected to DC power supply (KIKUSUI PMX 35-1A) (**Figure S1c**). The reactions were carried out at rt, under air (1 atm.), and at a constant current of 3 mA. Constant current mode of electrolysis –in the case of our substrates- offers many advantages over constant potential mode; related to its easier setup and full conversion of the substrates.

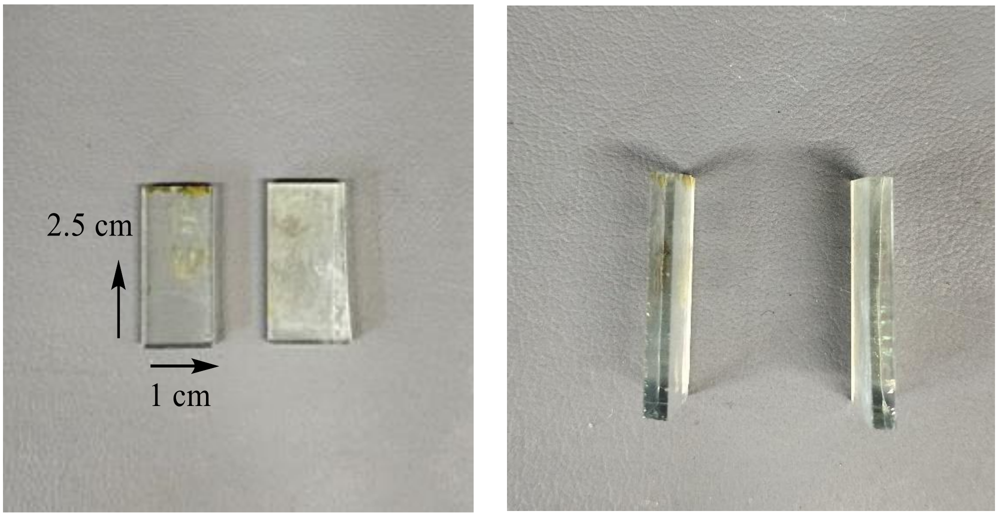

Figure S1a: FTO electrodes

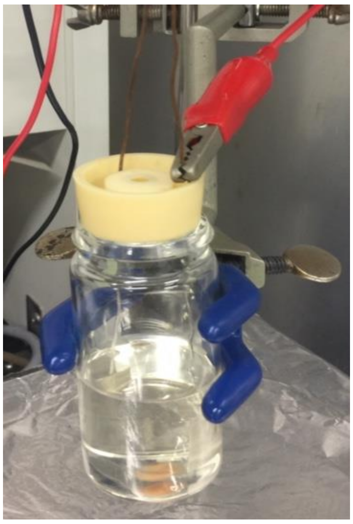

Figure S1b: Reaction vessel

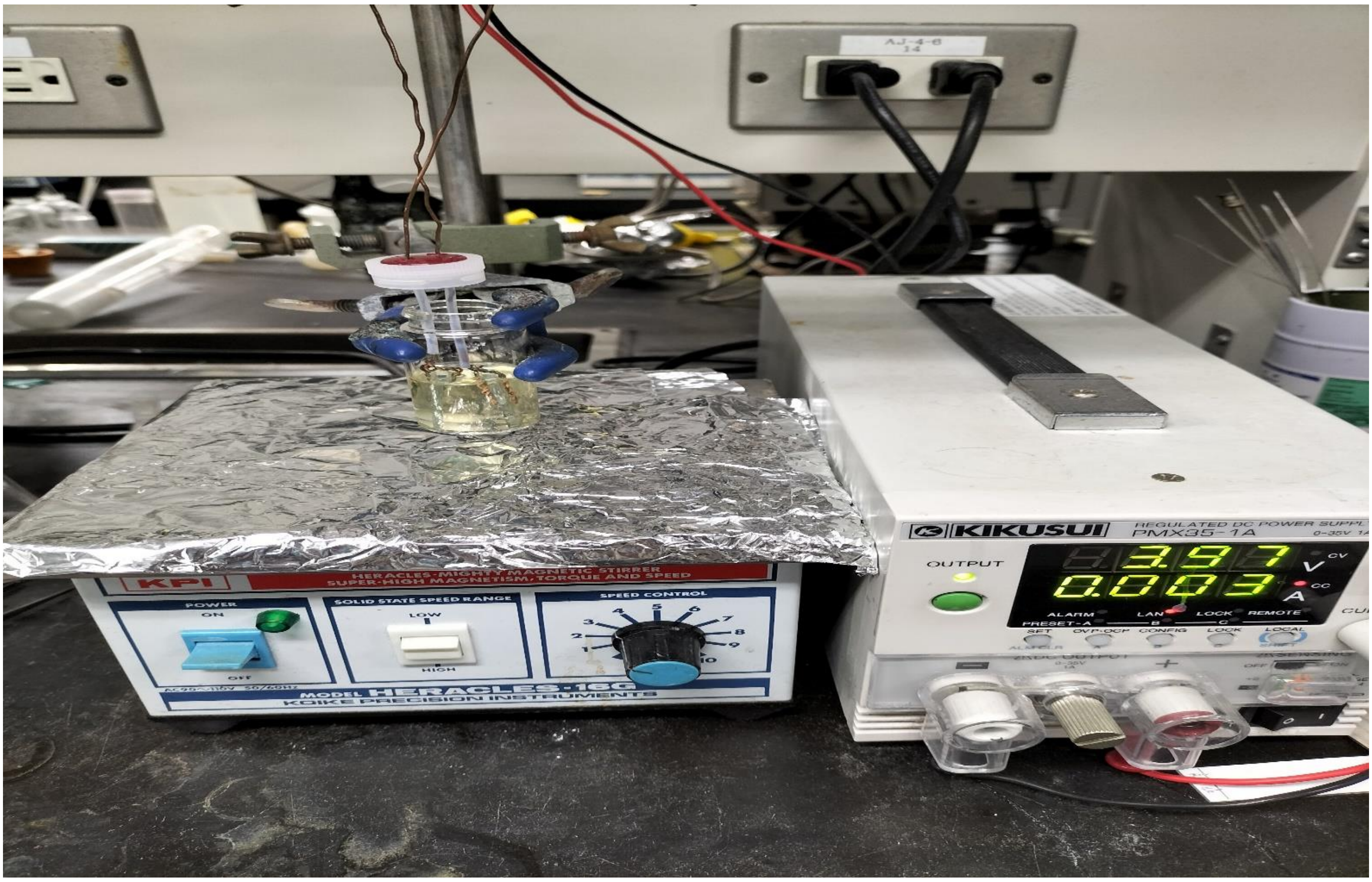

Figure S1c: DC power supply (KIKUSUI PMX 35.0-1.0A)

2. Supplementary Method 2: *experimental procedures for the synthesis of 1*

2-1. Synthesis of 1' and 1a-f

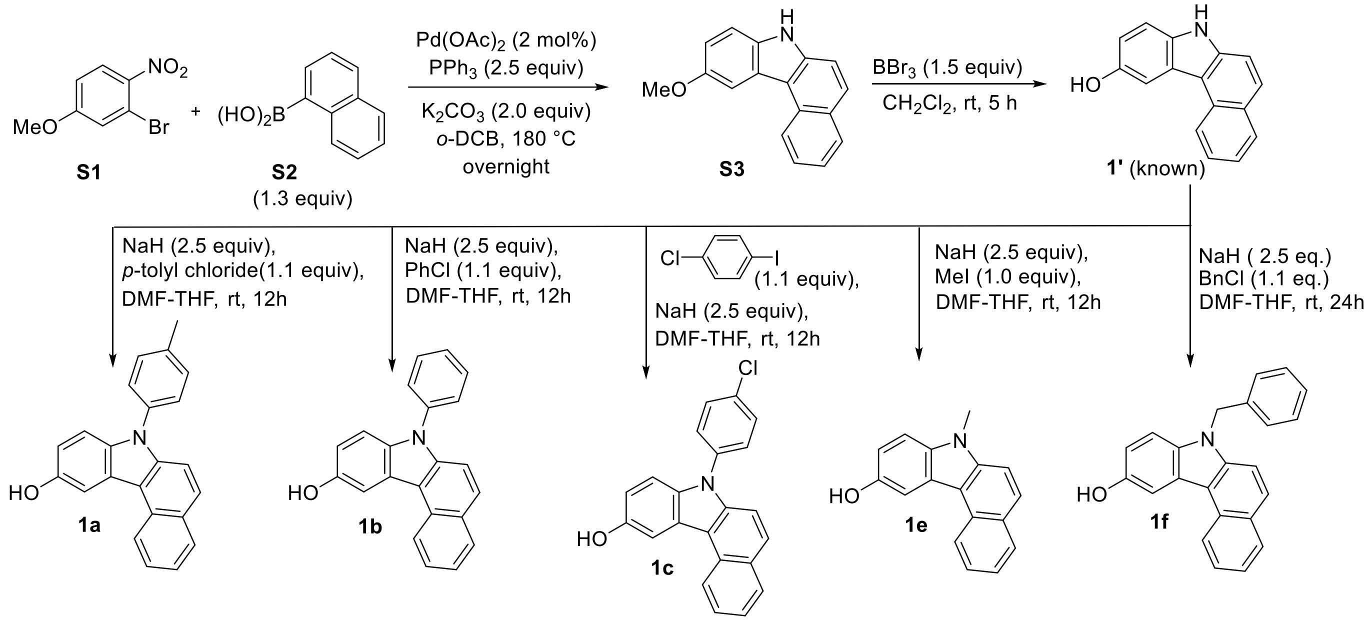

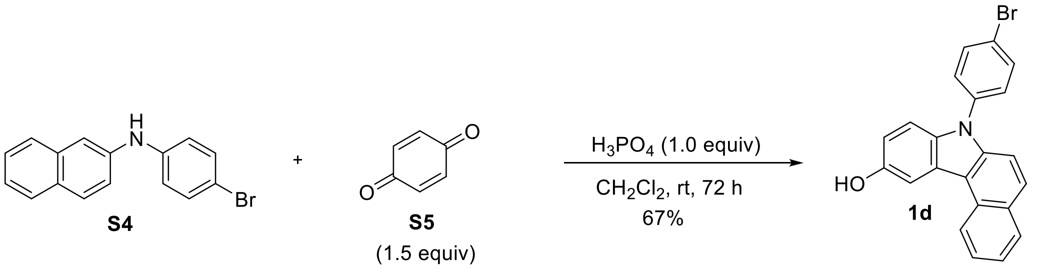

7*H*-Benzo[*c*]carbazol-10-ol (**1'**)[7)]

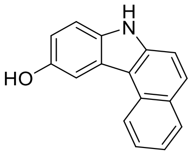

A mixture of 2-bromo-4-methoxy-1-nitrobenzene (**S1**) (574.6 mg, 2.48 mmol), naphthalen-1-boronic acid (**S2**) (553.7 mg, 3.22 mmol), $Pd(OAc)_2$ (11.1 mg, 0.05 mmol), PPh_3 (1.62 g, 6.19 mmol) and K_2CO_3 (684.5 mg, 4.95 mmol) in *o*-DCB (5.0 mL) was heated at 180 °C (oil bath temperature). After stirring for 20 h at the same temprature, the reaction mixture was filtrated through celite and concentrated in vacuo. The residue was purified by silica-gel column chromatography to afford 10-methoxy-7*H*-benzo[*c*]carbazole[7)] (**S3**, 71% yield) as a deep green solid. Then, BBr_3 (1.0 M in CH_2Cl_2, 4.5 mL, 4.5 mmol) was added to a solution of **S3** (592 mg, 3.00 mmol) in CH_2Cl_2 (30 mL) at 0 °C, and then the mixture was stirred for 5 h at rt. The reaction was quenched with sat. $NaHCO_3$ at 0 °C and the resulting mixture was extracted with EtOAc, drying over anhydrous Na_2SO_4, filtered, and evaporated *in vacuo.* The residue was purified by silica-gel column chromatography to afford **1'** (85% yield) as a brown solid (60% overall yield in 2 steps).

^{1}H NMR (400 MHz, $(CD_3)_2CO$): δ 10.58 (s, 1H), 8.67 (d, J = 8.7 Hz, 1H), 7.99-8.02 (m, 3H), 7.85 (d, J = 8.7 Hz, 1H), 7.72 (d, J = 8.7 Hz, 1H), 7.68 (ddd, J = 7.8, 7.3, 0.9 Hz, 1H), 7.50 (d, J = 8.2 Hz, 1H), 7.43 (ddd, J = 7.8, 7.3, 0.9 Hz, 1H), 7.02 (ddd, J = 8.7, 2.3, 0.9 Hz, 1H).

^{13}C NMR (100 MHz, $(CD_3)_2CO$): δ 152.33, 139.33, 134.40, 131.07, 129.97, 129.85, 127.66, 127.43, 125.16, 123.52, 123.20, 115.27, 114.59, 114.23, 112.76, 107.37.

HRMS (APCI): calcd for $C_{16}H_{12}NO$: *m/z* 234.0913 $[M + H]^+$, found 234.0910.

IR (KBr): 3524, 3400, 3348, 1492, 1209, 1164, 832, 803, 753, 737 cm^{-1}.

mp: 212-214 °C (*n*-hexane/EtOAc).

7-(*p*-Tolyl)-7*H*-benzo[*c*]carbazol-10-ol (**1a**)

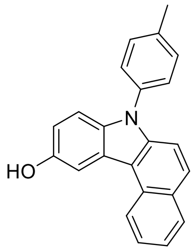

To a stirring solution of DMF (0.27 mL) and NaH (171.5 mg, 4.29 mmol) was added a solution of **1'** (401.2 mg,1.72 mmol) in THF (8.6 mL) at 0 °C. After stirring for 10 min at rt, *p*-tolyl chloride (217.7 mg, 1.72 mmol) was added to the solution and the reaction mixture was allowed to stir at 50 ºC. After stirring for 12 h, water was added to the reaction at 0 ºC. The resulting mixture was extracted with EtOAc. The combined organic layers were washed with brine, dried over anhydrous Na_2SO_4, filtered, and evaporated *in vacuo*. The residue was purified by silica-gel column chromatography to afford **1a** (67% yield) as a white solid.

^{1}H NMR (400 MHz, $CDCl_3$): δ 8.72 (d, J = 8.2 Hz, 1H), 8.07 (d, J = 2.7 Hz, 1H), 7.99 (d, J = 7.8 Hz, 1H), 7.80 (d, J = 8.7 Hz, 1H), 7.7 (ddd, J = 7.8, 7.3, 0.9 Hz, 1H), 7.52 (d, J = 9.2 Hz, 1H), 7.47 (ddd, J = 7.8, 7.3, 0.9 Hz, 1H), 7.40-7.45 (m, 4H), 7.35 (d, J = 8.7 Hz, 1H), 6.99 (dd, J = 8.9, 2.5 Hz, 1H), 4.90 (s, 1H), 2.51 (s, 3H).

^{13}C NMR (100 MHz, $CDCl_3$): δ 150.14, 139.48, 137.86, 135.65, 134.86, 130.61, 130.02, 129.31, 127.54, 127.07, 124.39, 123.14, 123.05, 114.90, 113.59, 112.01, 111.13, 107.41, 21.40 (Two carbons overlapped).

HRMS (APCI): calcd for $C_{23}H_{18}NO$: *m/z* 324.1383 [M + H]$^+$, found 324.1380.

IR (KBr): 3317, 3052, 3033, 2920, 1619, 1585, 1515, 1196, 1164, 819 cm^{-1}.

mp: 95-97 °C (*n*-hexane/EtOAc).

7-Phenyl-7*H*-benzo[*c*]carbazol-10-ol (**1b**)

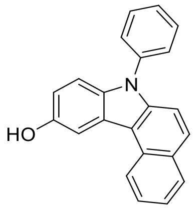

Following the same procedures as that of **1a**.

1b (55% yield): a white solid.

^{1}H NMR (400 MHz, $CDCl_3$): δ 8.73 (d, J = 8.7 Hz, 1H), 8.08 (d, J = 2.3 Hz, 1H), 7.99 (d, J = 8.2 Hz, 1H), 7.81 (d, J = 8.7 Hz, 1H), 7.71 (ddd, J = 7.8, 7.3, 0.9 Hz, 1H), 7.61-7.65 (m, 2H), 7.46-7.58 (m, 5H), 7.37 (d, J = 8.7 Hz, 1H), 7.00 (dd, J = 8.9, 2.5 Hz, 1H), 4.91 (s, 1H).

^{13}C NMR (100 MHz, $CDCl_3$): δ 150.24, 139.34, 137.58, 135.50, 130.04, 130.00, 129.40, 129.34, 127.92, 127.74, 127.64, 127.13, 124.53, 123.15, 115.05, 113.63, 111.94, 111.11, 107.45 (One carbon overlapped).

HRMS (APCI): calcd for $C_{22}H_{16}NO$: *m/z* 310.1226 [M + H]$^+$, found 310.1225.

IR (KBr): 3318, 3062, 1620, 1589, 1503, 1446, 1388, 1197, 1156, 803 cm^{-1}.

mp: 81-83 °C (*n*-hexane/EtOAc).

7-(4-Chlorophenyl)-7*H*-benzo[*c*]carbazol-10-ol (**1c**)

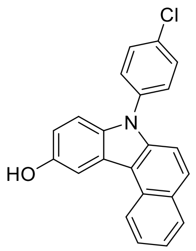

Following the same procedures as that of **1a**.

1c (61% yield): a white solid.

^{1}H NMR (400 MHz, $CDCl_3$): δ 8.70 (d, J = 8.2 Hz, 1H), 8.06 (d, J = 1.4 Hz, 1H), 7.99 (d, J = 7.8 Hz, 1H), 7.82 (d, J = 9.2 Hz, 1H), 7.71 (t, J = 7.6 Hz, 1H), 7.59 (d, J = 8.2 Hz, 2H), 7.48-7.50 (m, 4H), 7.32 (d, J = 8.7 Hz, 1H), 7.00 (dd, J = 8.7, 2.3 Hz, 1H), 4.94 (s, 1H).

^{13}C NMR (100 MHz, $CDCl_3$): δ 150.42, 139.12, 136.12, 135.27, 133.55, 130.29, 129.92, 129.46, 129.36, 128.98, 127.83, 127.24, 124.68, 123.31, 123.15, 115.27, 113.79, 111.58, 110.86, 107.59.

HRMS (APCI): calcd for $C_{22}H_{15}ClNO$: *m/z* 344.0837 [M + H]$^+$, found 344.0836.

IR (KBr): 3326, 3063, 1621, 1586, 1496, 1474, 1198, 1163, 802, 741 cm^{-1}.

mp: 88-90 °C (*n*-hexane/EtOAc).

7-(4-Bromophenyl)-7*H*-benzo[*c*]carbazol-10-ol (**1d**)

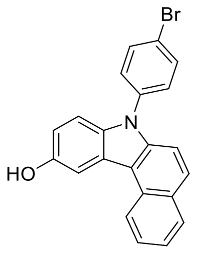

A mixture of **S4** (2.5 mmol), *p*-quinone (**S5**) (3.75 mmol), and *o*-phosphoric acid (130 μL, 2.5 mmol) in CH_2Cl_2 (25.0 mL) was stirred at rt. After stirring for 72 h, the reaction mixture was quenched by water and further extracted with CH_2Cl_2. The combined organic extracts were washed with brine and dried over anhydrous Na_2SO_4, filtered, and evaporated *in vacuo*. The crude product was purified by silica-gel column chromatography to afford **1d** (67% yield) as a white solid.

^{1}H NMR (400 MHz, $CDCl_3$) δ 8.72 (d, *J* = 8.7 Hz, 1H), 8.06 (d, *J* = 2.3 Hz, 1H), 7.99 (d, *J* = 8.2 Hz, 1H), 7.82 (d, *J* = 8.7 Hz, 1H), 7.70-7.77 (m, 3H), 7.50 (d, *J* = 8.7 Hz, 2H), 7.46 (dd, *J* = 6.4, 1.8 Hz, 2H), 7.34 (d, *J* = 9.2 Hz, 1H), 7.00 (dd, *J* = 8.7, 2.3 Hz, 1H), 4.83 (s, 1H).

^{13}C NMR (100 MHz, $CDCl_3$) δ 150.42, 139.05, 136.66, 135.21, 133.27, 129.92, 129.48, 129.36, 129.29, 127.84, 127.25, 124.73, 123.33, 123.15, 121.45, 115.32, 113.80, 111.56, 110.86, 107.62.

HRMS (APCI): calcd for $C_{22}H_{15}BrNO$: *m/z* 388.0332 $[M + H]^+$, found 388.0345.

IR (KBr): 3358, 3061, 2955, 2925, 1620, 1493, 1472, 1162, 803, 742 cm^{-1}.

mp: 95-97 °C (*n*-hexane/EtOAc).

7-Methyl-7*H*-benzo[*c*]carbazol-10-ol (**1e**)

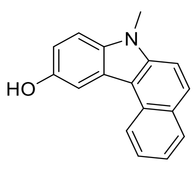

Following the same procedures as that of **1a**.

1e (75% yield): a white solid.

^{1}H NMR (400 MHz, $(CD_3)_2CO$): *δ* 8.68 (d, *J* = 8.2 Hz, 1H), 8.04-8.06 (m, 2H), 8.02 (s, 1H), 7.93 (d, *J* = 9.2 Hz, 1H), 7.80 (d, *J* = 8.7 Hz, 1H), 7.69 (ddd, *J* = 7.8, 7.3, 0.9 Hz, 1H), 7.54 (d, *J* = 9.2 Hz, 1H), 7.43 (ddd, *J* = 7.8, 7.3, 0.9 Hz, 1H), 7.10 (dd, *J* = 8.7, 2.3 Hz, 1H), 4.02 (s, 3H).

^{13}C NMR (100 MHz, $(CD_3)_2CO$): *δ* 152.45, 139.96, 135.58, 130.94, 130.02, 129.65, 127.72, 127.59, 124.56, 123.45, 123.20, 114.71, 114.51, 112.03, 110.79, 107.53, 29.53.

HRMS (APCI): calcd for $C_{17}H_{14}NO$: *m/z* 248.1070 $[M + H]^+$, found 248.1068.

IR (KBr): 3189, 3072, 3036, 2923, 1616, 1528, 1479, 1173, 1156, 789 cm^{-1}.

mp: 100-102 °C (*n*-hexane/EtOAc).

7-Benzyl-7*H*-benzo[*c*]carbazol-10-ol (**1f**)

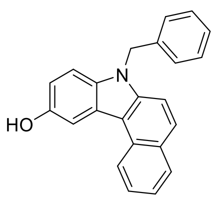

Following the same procedures used as that of **1a**.

1f (80% yield): a white solid.

^{1}H NMR (400 MHz, $CDCl_3$): *δ* 8.71 (d, *J* = 8.2 Hz, 1H), 8.06 (d, *J* = 2.3 Hz, 1H), 7.99 (d, *J* = 8.2 Hz, 1H), 7.85 (d, *J* = 8.7 Hz, 1H), 7.71 (ddd, *J* = 8.0, 7.6, 1.4 Hz, 1H), 7.58 (d, *J* = 9.2 Hz, 1H), 7.46 (ddd, *J* = 7.8, 7.3, 0.9 Hz, 1H), 7.36 (d, *J* = 8.7 Hz, 1H), 7.22-7.25 (m, 3H), 7.09 (d, *J* = 7.8 Hz, 2H), 7.02 (dd, *J* = 8.7, 2.3 Hz, 1H), 5.64 (s, 2H), 4.78 (s, 1H).

^{13}C NMR (100 MHz, $CDCl_3$): *δ* 149.86, 139.01, 137.33, 134.8:6, 130.09, 129.31, 129.13, 128.61, 127.61, 127.33, 127.10, 126.29, 124.22, 122.99, 122.92, 114.64, 113.58, 111.07, 110.19, 107.68, 46.68.

HRMS (APCI): calcd for $C_{23}H_{18}NO$: *m/z* 324.1383 $[M + H]^+$, found 324.1380.

IR (KBr): 3335, 3061, 3033, 2926, 1698, 1620, 1475, 1353, 1170, 801 cm^{-1}.

mp: 92-94 °C (*n*-hexane/EtOAc).

2-2. Synthesis of 1g-h

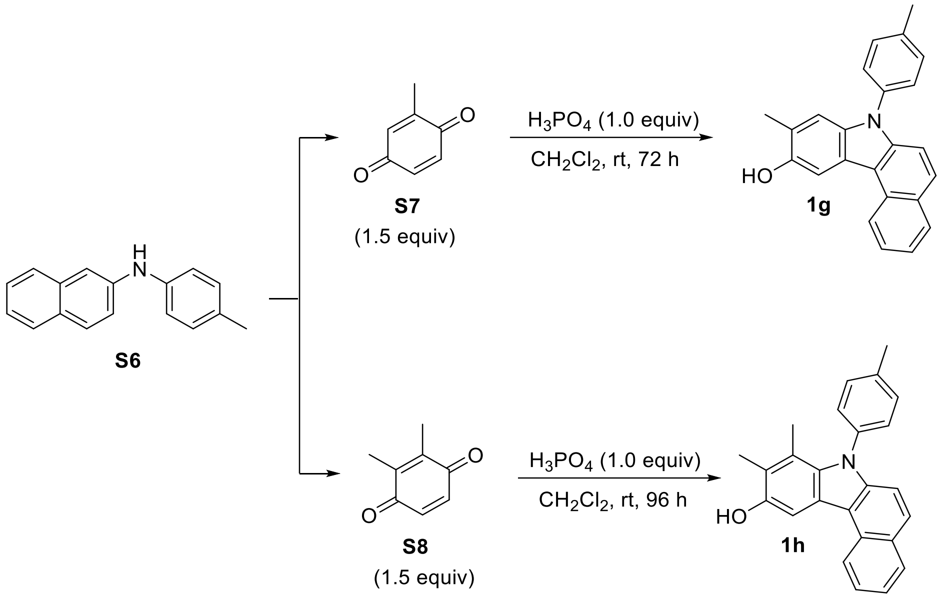

9-Methyl-7-(*p*-tolyl)-7*H*-benzo[*c*]carbazol-10-ol (**1g**)/8,9-Dimethyl-7-(*p*-tolyl)-7*H*-benzo[*c*]carbazol-10-ol (**1h**)

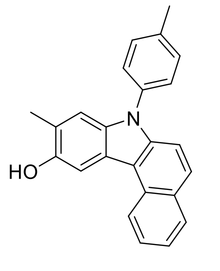

A mixture of **S6** (582.8 mg, 2.5 mmol), corresponding dione (**S7**/**S8**) (3.75 mmol), and orthophosphoric acid (130 μL, 2.5 mmol) in CH_2Cl_2 (25.0 mL) was stirred at rt. After stirring for 72 h, the reaction mixture was quenched by water and further extracted with CH_2Cl_2. The combined organic extracts were washed with brine and dried over anhydrous Na_2SO_4, filtered, and evaporated *in vacuo*. The residue was purified by silica-gel column chromatography to afford **1g** (85% yield)/**1h** (70% yield) as white solids.

^{1}H NMR (400 MHz, $CDCl_3$): δ 8.70 (d, J = 8.2 Hz, 1H), 8.01 (s, 1H), 7.98 (d, J = 8.2 Hz, 1H), 7.78 (d, J = 8.7 Hz, 1H), 7.69 (t, J = 7.6 Hz, 1H), 7.50 (d, J = 8.7 Hz, 1H), 7.44-7.48 (m, 5H), 7.23 (s, 1H), 4.79 (s, 1H), 2.52 (s, 3H), 2.43 (s, 3H).

^{13}C NMR (100 MHz, $CDCl_3$): 148.83, 138.93, 137.75, 135.73, 135.03, 130.60, 129.84, 129.30, 129.24, 127.59, 126.84, 123.15, 122.91, 122.52, 122.36, 115.05, 111.98, 111.86, 107.02, 21.40, 17.06 (One carbon overlapped).

HRMS (APCI): calcd for $C_{24}H_{20}NO$: *m/z* 338.1539 $[M + H]^+$, found 338.1533.

IR (KBr): 3393, 3059, 3035, 2920, 2858, 1620, 1517, 1260, 1176, 803 cm^{-1}.

mp: 73-74 °C (*n*-hexane/EtOAc).

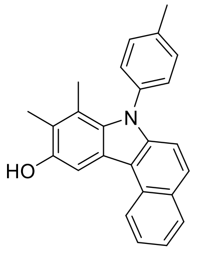

^{1}H NMR (400 MHz, $CDCl_3$): δ 8.72 (d, J = 8.7 Hz, 1H), 7.95-7.97 (m, 2H), 7.67-7.72 (m, 2H), 7.45 (t, J = 7.3 Hz, 1H), 7.36 (d, J = 8.2 Hz, 2H), 7.31 (d, J = 7.8 Hz, 2H), 7.22 (d, J = 8.7 Hz, 1H), 4.80 (s, 1H), 2.51 (s, 3H), 2.36 (s, 3H), 2.03 (s, 3H).

^{13}C NMR (100 MHz, $CDCl_3$): δ 148.66, 141.26, 138.25, 137.89, 135.22, 130.09, 129.68, 129.41, 129.28, 129.21, 126.79, 126.77, 123.12, 122.85, 121.72, 121.61, 114.73, 112.37, 104.78, 21.45, 15.70, 12.52 (One carbon overlapped).

HRMS (APCI): calcd for $C_{25}H_{22}NO$: *m/z* 352.1696 $[M + H]^+$, found 352.1694.

IR (KBr): 3465, 3071, 2955, 2924, 2861, 1621, 1513, 1443, 1202, 822 cm^{-1}.

mp: 90-92 °C (*n*-hexane/EtOAc).

2-3. Synthesis of 1i

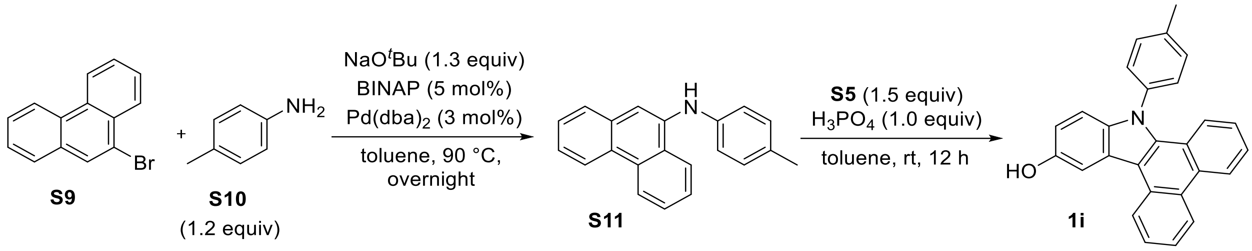

N-(*p*-Tolyl)phenanthren-9-amine (**S11**)

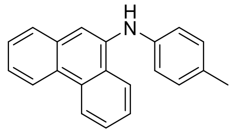

A toluene solution (30 mL) of **S9** (30 mmol), *p*-toluidine (**S10**) (3.8 g, 36 mmol), $Pd(dba)_2$ (3 mol%), BINAP (5 mol%), and NaO*t*-Bu (4.5 g, 40 mmol) was stirred at 90 °C under N_2 atmosphere. After stirring for 12 h, the reaction mixture was filtered and the filtrate was directly purified on silica-gel to give **S11** (54% yield).

^{1}H NMR (400 MHz, $CDCl_3$): δ 8.75 (d, *J* = 7.8 Hz, 1H), 8.62 (dd, *J* = 6.4, 2.8 Hz, 1H), 8.14 (d, *J* = 8.2 Hz, 1H), 7.67-7.72 (m, 2H), 7.63 (ddd, J = 7.8, 7.3, 0.9 Hz, 1H), 7.51-7.54 (m, 2H), 7.49 (s, 1H), 7.14 (d, *J* = 7.8 Hz, 2H), 7.02 (d, *J* = 8.2 Hz, 2H), 5.89 (s, 1H), 2.34 (s, 3H).

^{13}C NMR (100 MHz, $CDCl_3$): δ 141.73, 137.87, 132.78, 131.44, 130.58, 129.98, 127.77, 127.41, 127.37, 126.92, 126.83, 126.58, 124.70, 123.31, 122.51, 122.14, 119.03, 112.67, 20.79.

HRMS (APCI): calcd for $C_{21}H_{18}N$: *m/z* 284.1434 $[M + H]^+$, found 284.1439.

IR (KBr): 3411, 3052, 3024, 2911, 2862, 1600, 1518, 1325, 1239, 818 cm^{-1}.

mp: 110-112 °C (*n*-hexane/EtOAc).

9-(*p*-Tolyl)-9*H*-dibenzo[*a,c*]carbazol-12-ol (**1i**)

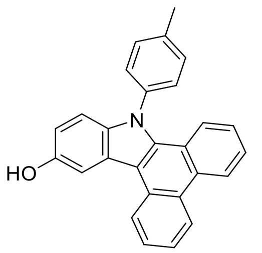

Following the same procedures as that of **1g**.

1i (52% yield): a white solid.

^{1}H NMR (400 MHz, $CDCl_3$): δ 8.77-8.81 (m, 3H), 8.07 (d, *J* = 2.3 Hz, 1H), 7.78 (ddd, *J* = 7.8, 7.3, 1.4 Hz, 1H), 7.60 (ddd, *J* = 7.8, 7.3, 0.9 Hz, 1H), 7.52-7.58 (m, 2H), 7.44 (d, *J* = 8.2 Hz, 2H), 7.38 (dd, *J* = 6.4, 1.8 Hz, 2H), 7.29 (ddd, *J* = 7.8, 7.3, 0.9 Hz, 1H), 7.07 (d, *J* = 9.2 Hz, 1H), 6.93 (dd, *J* = 8.7, 2.3 Hz, 1H), 4.82 (s, 1H), 2.56 (s, 3H).

^{13}C NMR (100 MHz, $CDCl_3$): δ 150.52, 138.93, 137.75, 135.47, 131.01, 130.94, 130.08, 128.87, 127.47, 127.26, 126.02, 125.88, 124.28, 123.92, 123.89, 123.68, 123.60, 123.51, 123.37, 113.69, 113.33, 111.79, 106.92, 21.57 (One carbon overlapped).

HRMS (APCI): calcd for $C_{27}H_{20}NO$: *m/z* 374.1539 $[M + H]^+$, found 374.1537.

IR (KBr): 3382, 3086, 3034, 2924, 1701, 1609, 1512, 1375, 1202, 908 cm^{-1}.

mp: 73-75 °C (*n*-hexane/EtOAc).

2-4. Synthesis of 1j

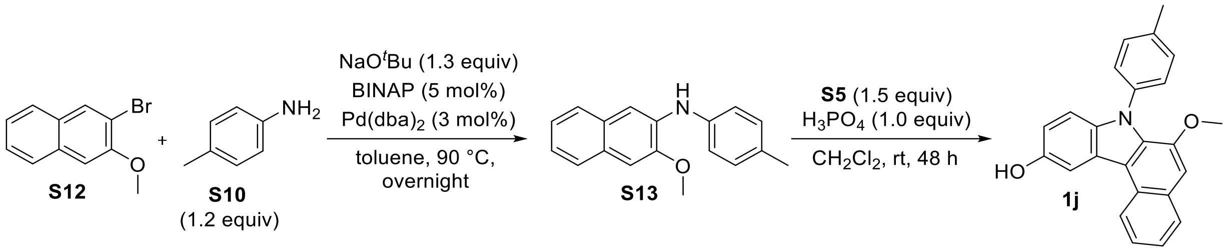

3-Methoxy-*N*-(*p*-tolyl)naphthalen-2-amine (**S13**)

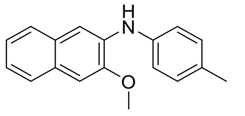

S13 was prepared according to the literature procedure.[8)]

S13 (89% yield): a white solid.

^{1}H NMR (400 MHz, $CDCl_3$): δ 7.65 (dd, J = 7.8, 1.8 Hz, 1H), 7.55 (dd, J = 8.2, 1.8 Hz, 1H), 7.44 (s, 1H), 7.24-7.29 (m, 2H), 7.16-7.22 (m, 4H), 7.11 (s, 1H), 6.35 (s, 1H), 4.03 (s, 3H), 2.36 (s, 3H).

^{13}C NMR (100 MHz, $CDCl_3$): δ 148.50, 139.28, 134.62, 131.98, 130.06, 129.81, 128.40, 126.36, 125.89, 124.22, 123.15, 120.77, 106.98, 105.33, 55.75, 20.94.

HRMS (APCI): calcd for $C_{18}H_{18}NO$: *m/z* 264.1383 $[M + H]^+$, found 264.1382.

IR (KBr): 3419, 3007, 2982, 2938, 2917, 1608, 1524, 1257, 1017, 751 cm^{-1}.

mp: 76-78 °C (*n*-hexane/EtOAc).

6-Methoxy-7-(*p*-tolyl)-7*H*-benzo[*c*]carbazol-10-ol (**1j**)

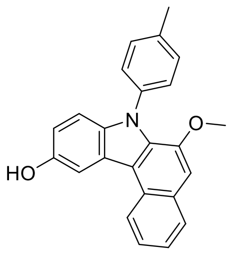

Following the same procedures as that of **1g**.

1j (56% yield): a white solid.

^{1}H NMR (400 MHz, $CDCl_3$): δ 8.66 (d, J = 8.2 Hz, 1H), 8.04 (d, J = 1.8 Hz, 1H), 7.87 (d, J = 8.0 Hz, 1H), 7.57 (ddd, J = 7.8, 7.3, 0.9 Hz, 1H), 7.44 (ddd, J = 7.8, 7.3, 0.9 Hz, 1H), 7.28-7.33 (m, 4H), 7.13 (d, J = 9.2 Hz, 1H), 7.11 (s, 1H), 6.95 (dd, J = 8.6, 2.2 Hz, 1H), 4.90 (s, 1H), 3.75 (s, 3H), 2.49 (s, 3H).

^{13}C NMR (100 MHz, $CDCl_3$): δ 150.23, 147.81, 137.38, 137.29, 137.16, 131.41, 130.20, 129.13, 128.36, 127.66, 126.02, 124.61, 124.23, 123.53, 122.87, 117.12, 113.83, 111.78, 107.14, 104.83, 55.55, 21.43.

HRMS (APCI): calcd for $C_{24}H_{20}NO_2$: *m/z* 354.1489 $[M + H]^+$, found 354.1486.

IR (KBr): 3362, 3033, 2933, 2832, 1698, 1515, 1448, 1271, 1197, 812 cm^{-1}.

mp: 75-77 °C (*n*-hexane/EtOAc).

2-5. Synthesis of 1k

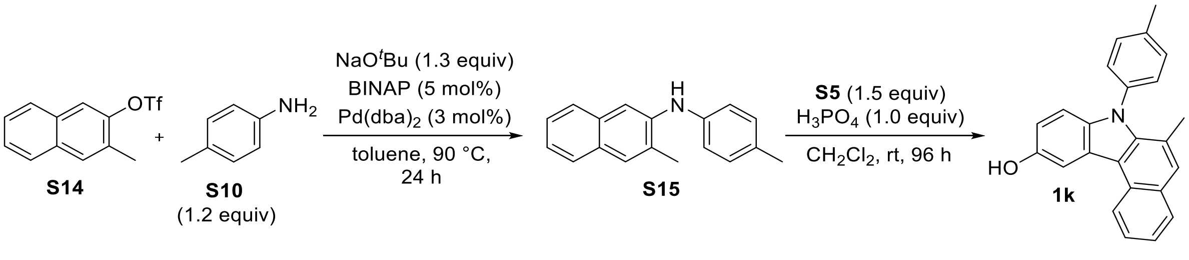

3-Methyl-*N*-(*p*-tolyl)naphthalen-2-amine (**S15**)

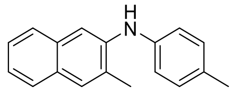

A coupling **S14** with **S10** was performed under the reported Buchwald–Hartwig amination conditions.[9)]

S15 (82% yield): a white solid.

^{1}H NMR (400 MHz, $CDCl_3$): δ 7.68 (d, J = 8.2 Hz, 1H), 7.64 (s, 1H), 7.58 (d, J = 8.2 Hz, 1H), 7.48 (s, 1H), 7.33 (ddd, J = 7.8, 7.3, 0.9 Hz, 1H), 7.27 (ddd, J = 7.8, 7.3, 0.9 Hz, 1H), 7.16 (d, J = 8.5 Hz, 2H), 7.08 (d, J = 8.2 Hz, 2H), 5.49 (s, 1H), 2.44 (s, 3H), 2.35 (s, 3H).

^{13}C NMR (100 MHz, $CDCl_3$): δ 141.37, 140.50, 133.46, 131.51, 130.10, 129.13, 129.08, 127.71, 126.94, 126.22, 125.57, 123.37, 120.03, 110.38, 20.90, 18.45.

HRMS (APCI): calcd for $C_{18}H_{18}N$: *m/z* 248.1434 $[M + H]^+$, found 248.1430.

IR (KBr): 3423, 3055, 3019, 2918, 2860, 1613, 1524, 1313, 1248, 813 cm^{-1}.

mp: 79-81 °C (*n*-hexane/EtOAc).

6-Methyl-7-(*p*-tolyl)-7*H*-benzo[*c*]carbazol-10-ol (**1k**)

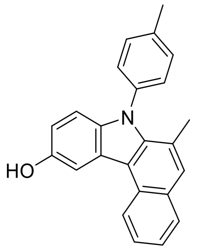

Following the same procedures used as that of **1g**.

1k (69% yield): a white solid.

^{1}H NMR (400 MHz, $CDCl_3$): δ 8.71 (d, J = 8.2 Hz, 1H), 8.07 (s, 1H), 7.90 (d, J = 7.8 Hz, 1H), 7.64 (ddd, J = 7.8, 7.3, 1.8 Hz, 1H), 7.54 (s, 1H), 7.45 (ddd, J = 8.1, 7.1, 2.3 Hz, 1H), 7.24-7.31 (m, 4H), 7.00 (dd, J = 8.7, 3.2 Hz, 1H), 6.94 (dt, J = 8.7, 2.8 Hz, 1H), 4.97 (s, 1H), 2.51 (s, 3H), 2.13 (s, 3H).

^{13}C NMR (100 MHz, $CDCl_3$): δ 150.13, 139.27, 138.69, 137.70, 137.12, 129.88, 129.53, 129.33, 129.04, 128.77, 128.24, 126.16, 123.99, 123.36, 123.11, 122.84, 115.61, 113.51, 111.54, 107.14, 21.48, 20.64.

HRMS (APCI): calcd for $C_{24}H_{20}NO$: *m/z* 338.1539 $[M + H]^+$, found 338.1536.

IR (KBr): 3370, 3035, 2959, 2924, 1700, 1620, 1515, 1378, 1171, 740 cm^{-1}.

mp: 82-84 °C (*n*-hexane/EtOAc).

2-6. Synthesis of 1l

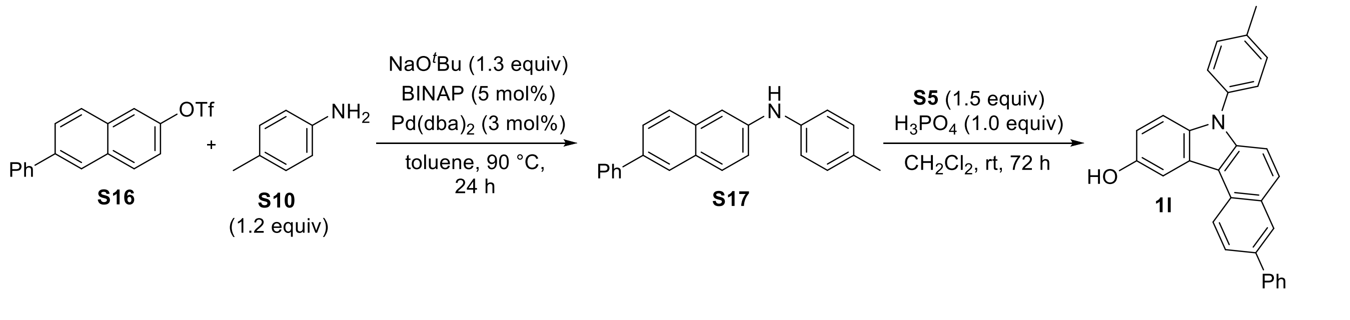

6-Phenyl-*N*-(*p*-tolyl)naphthalen-2-amine (**S17**)

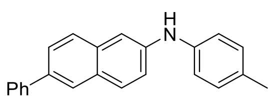

A coupling of **S16** and **S10** was performed following the same procedures used as that of **S11**.

S17 (51% yield): a white solid.

^{1}H NMR (400 MHz, $CDCl_3$): δ 7.93 (s, 1H), 7.78 (d, J = 9.2 Hz, 1H), 7.67-7.71 (m, 4H), 7.47 (ddd, J = 7.8, 7.3, 1.4 Hz, 2H), 7.38 (d, J = 2.3 Hz, 1H), 7.35 (tt, J = 7.3, 1.5 Hz, 1H), 7.20 (dd, J = 8.7, 2.3 Hz, 1H), 7.10-7.16 (m, 4H), 5.82 (s, 1H), 2.34 (s, 3H).

^{13}**C NMR** (100 MHz, $CDCl_3$): δ 142.02, 141.43, 140.10, 135.98, 134.07, 131.63, 130.11, 129.60, 129.17, 128.93, 127.26, 127.07, 126.99, 126.20, 125.70, 120.06, 119.61, 110.02, 20.90.

HRMS (APCI): calcd for $C_{23}H_{20}N$: *m/z* 310.1590 $[M + H]^+$, found 310.1586.

IR (KBr): 3421, 3060, 3021, 2922, 2857, 1605, 1523, 1445, 1307, 816 cm^{-1}.

mp: 162-163 °C (*n*-hexane/EtOAc).

3-Phenyl-7-(*p*-tolyl)-7*H*-benzo[*c*]carbazol-10-ol (**1l**)

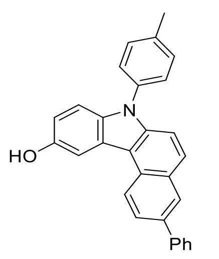

Following the same procedures used as that of **1g**.

1l (61% yield): a white solid.

^{1}**H NMR** (400 MHz, $CDCl_3$): δ 8.77 (d, J = 8.7 Hz, 1H), 8.20 (d, J = 1.8 Hz, 1H), 8.09 (d, J = 2.3 Hz, 1H), 7.98 (dd, J = 8.7, 1.8 Hz, 1H), 7.86 (d, J = 9.2 Hz, 1H), 7.79 (dd, J = 7.3,1.37 Hz, 2H), 7.49-7.55 (m, 3H), 7.43-7.46 (m, 4H), 7.38-7.41 (tt, J = 5.8, 1.15 Hz, 1H), 7.36 (d, J = 8.7 Hz, 1H), 7.01 (dd, J = 8.7, 2.3 Hz, 1H), 4.86 (s, 1H), 2.51 (s, 3H).

^{13}**C NMR** (100 MHz, $CDCl_3$): δ 150.26, 141.42, 139.61, 137.90, 135.78, 135.68, 134.87, 130.63, 129.68, 129.15, 129.00, 127.85, 127.55, 127.40, 127.26, 127.13, 126.48, 124.41, 123.64, 114.90, 113.75, 112.44, 111.22, 107.45, 21.40.

HRMS (APCI): calcd for $C_{29}H_{22}NO$: *m/z* 400.1696 $[M + H]^+$, found 400.1692.

IR (KBr): 3355, 3058, 3032, 2922, 1698, 1588, 1516, 1366, 1167, 802 cm^{-1}.

mp: 112-114 °C (*n*-hexane/EtOAc).

2-7. Synthesis of 1m

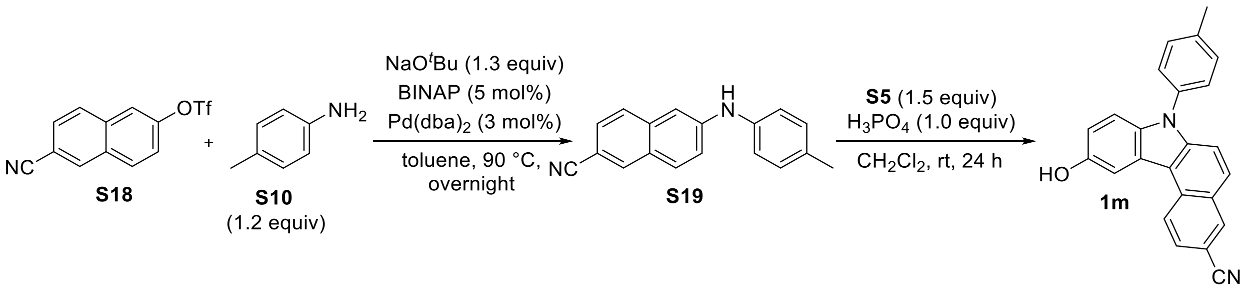

6-(*p*-tolylamino)-2-naphthonitrile (**S19**)

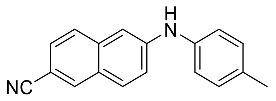

A coupling of **S18** with **S10** was performed following the same procedures as that of **S11**.

S19 (72% yield): a yellow solid.

^{1}**H NMR** (600 MHz, $CDCl_3$): δ 8.05 (s, 1H), 7.73 (d, J = 8.9 Hz, 1H), 7.60 (d, J = 8.2 Hz, 1H), 7.47 (dd, J = 8.6, 1.7 Hz, 1H), 7.28 (d, J = 9.1 Hz, 1H), 7.18-7.22 (m, 3H), 7.13-7.16 (m, 2H), 5.99 (s, 1H), 2.37 (s, 3H).

^{13}**C NMR** (150 MHz, $CDCl_3$): δ 145.27, 138.34, 136.73, 133.91, 133.41, 130.29, 129.99, 127.24, 127.22, 127.16, 121.35, 120.27, 120.12, 107.83, 105.48, 21.01.

HRMS (APCI): calcd for $C_{18}H_{15}N_2$: m/z 259.1230 $[M + H]^+$, found 259.1230.

IR (KBr): 3357, 3060, 3026, 2914, 2857, 2217, 1533, 1407, 1314, 816 cm^{-1}.

mp: 170-172 °C (*n*-hexane/EtOAc).

10-Hydroxy-7-(p-tolyl)-7H-benzo[c]carbazole-3-carbonitrile (**1m**)

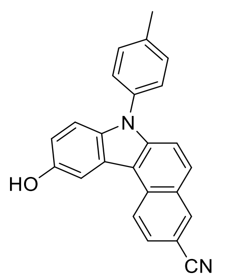

Following the same procedures used as that of **1g**.

1m (52% yield): a yellow solid.

^{1}H NMR (600 MHz, $(CD_3)_2CO$): δ 8.87 (d, J = 8.2 Hz, 1H), 8.54 (s, 1H), 8.34 (s, 1H), 8.09 (s, 1H), 8.00 (d, J = 8.9 Hz, 1H), 7.96 (d, J = 8.2 Hz, 1H), 7.64 (d, J = 8.9 Hz, 1H), 7.51-7.58 (m, 4H), 7.34 (d, J = 8.9 Hz, 1H), 7.11 (d, J = 8.2 Hz, 1H), 2.51 (s, 3H).

^{13}C NMR (150 MHz, $(CD_3)_2CO$): δ 152.80, 140.44, 138.35, 134.82, 134.27, 131.63, 130.76, 128.37, 127.85, 127.39, 124.06, 123.86, 119.37, 114.87, 114.56, 113.71, 112.20, 111.36, 110.60, 106.75, 105.68, 20.41.

HRMS (APCI): calcd for $C_{24}H_{17}N_2O$: m/z 349.1335 $[M + H]^+$, found 349.1323.

IR (KBr): 3375, 3028, 3005, 2975, 2220, 1675, 1534, 1513, 1180, 798 cm^{-1}.

mp: 135-137 °C (*n*-hexane/EtOAc).

2.8. Synthesis of 1p

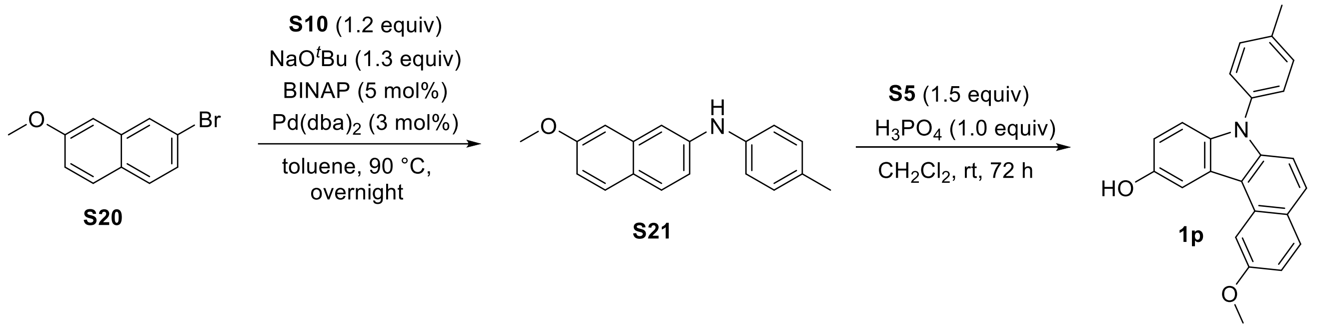

7-Methoxy-*N*-(*p*-tolyl)naphthalen-2-amine (**S21**)

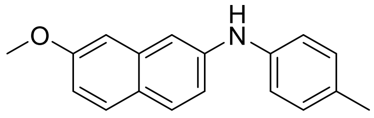

A coupling of **S20** and **S10** was performed following the same procedures used as that of **S11**. (**S21**) was obtained in 72% yield as a white solid.

^{1}H NMR (400 MHz, $CDCl_3$): δ 7.60-7.65 (m, 2H), 7.26 (d, J = 2.3 Hz, 1H), 7.14 (d, J = 8.7 Hz, 2H), 7.10 (d, J = 8.3 Hz, 2H), 7.02 (dd, J = 8.7, 2.3 Hz, 1H), 6.93-6.96 (m, 2H), 5.75 (s, 1H), 3.89 (s, 3H), 2.34 (s, 3H).

^{13}C NMR (100 MHz, $CDCl_3$): δ 158.36, 142.51, 140.18, 136.12, 131.56, 130.07, 129.25, 129.05, 124.38, 119.74, 117.16, 115.86, 109.45, 104.79, 55.35, 20.91.

HRMS (APCI): calcd for $C_{18}H_{18}NO$: m/z 264.1383 $[M + H]^+$, found 264.1378.

IR (KBr): 3387, 3027, 2998, 2922, 2861, 1631, 1514, 1214, 1030, 817 cm^{-1}.

mp: 78-80 °C (*n*-hexane/EtOAc).

2-Methoxy-7-(*p*-tolyl)-*7H*-benzo[*c*]carbazol-10-ol (**1p**)

Following the same procedures used for preparation of compound (**1g**). (**1p**) was obtained in 56% yield as a white solid.

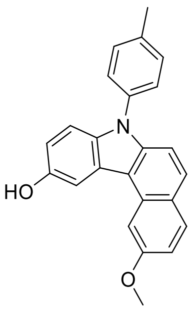

^{1}H NMR (600 MHz, $CDCl_3$): δ 8.01 (d, J = 2.1 Hz, 1H), 7.99 (d, J = 2.1 Hz, 1H), 7.88 (d, J = 8.9 Hz, 1H), 7.73 (d, J = 8.9 Hz, 1H), 7.40-7.44 (m, 4H), 7.36 (d, J = 8.9 Hz, 1H), 7.33 (d, J = 8.3 Hz, 1H), 7.13 (dd, J = 8.6, 1.7 Hz, 1H), 6.98 (dd, J = 8.6, 1.7 Hz, 1H), 5.13 (s, 1H), 4.06 (s, 3H), 2.50 (s, 3H).

^{13}C NMR (150 MHz, $CDCl_3$): δ 158.88, 150.05, 140.09, 137.81, 135.61, 134.92, 131.20, 130.76, 130.59, 127.55, 127.35, 124.52, 124.35, 114.33, 114.22, 113.26, 111.00, 109.47, 107.14, 103.33, 55.61, 21.40.

HRMS (APCI): calcd for $C_{24}H_{20}NO_2$: m/z 354.1489 $[M + H]^+$, found 354.1489.

IR (KBr): 3381, 3035, 2954, 2923, 2853, 1624, 1516, 1228, 1157, 814 cm^{-1}.

mp: 82-84 °C (*n*-hexane/EtOAc).

3. Supplementary Method 3: *optimization of reaction conditions*

Table S1. Screening of different solvents.[a]

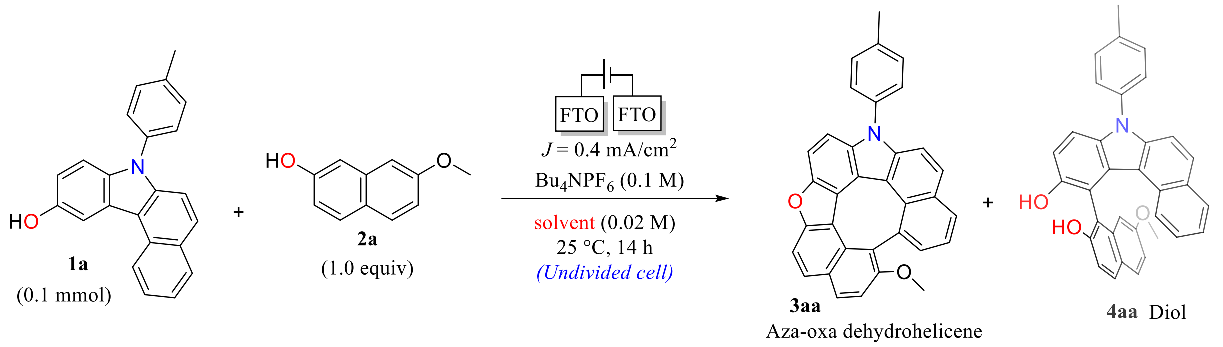

Entry	solvent	**3aa** yield (%)[b]	**4aa** yield (%)[b]
1	CH_2Cl_2	28	68
2	THF	17	70
3	CH_3CN	14	50
4	CH_3OH	12	57

[a] Carried out in different solvents (5 mL) under air (1 atm).

[b] Determined by ^{1}H NMR spectroscopy using 1,3,5-trimethoxybenzene as an internal standard.

Table S2. Screening of electrochemical parameters (electrodes, and electrolytes).[a]

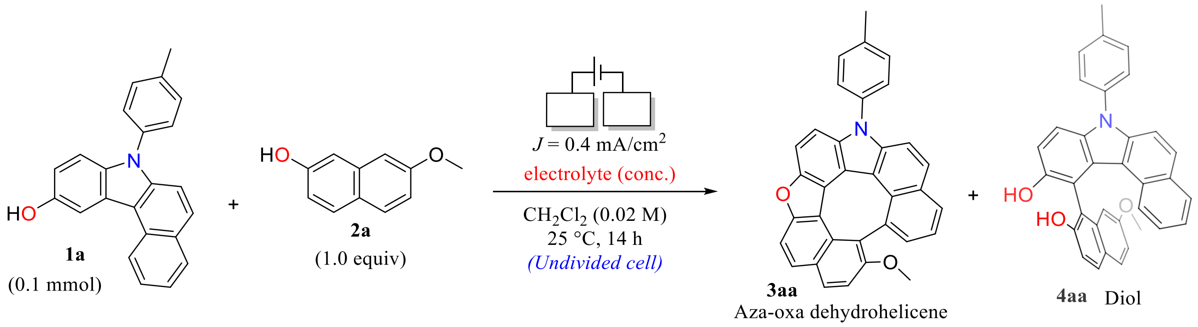

entry	Electrode	electrolyte (conc.)	**3aa** yield (%)[b]	**4aa** yield (%)[b]
1	Pt(+)-Pt(-)	Bu_4NPF_6 (0.1 M)	25	64
2	Pt(+)–C(-)	Bu_4NPF_6 (0.1 M)	22	55
3	Pt(+)–FTO(-)	Bu_4NPF_6 (0.1 M)	24	62
4	FTO(+)–FTO(-)	Bu_4NPF_6 (0.1 M)	28	68
5	FTO(+)–FTO(-)	$LiClO_4$ (0.1 M)	15	50
6	FTO(+)–FTO(-)	Bu_4NClO_4 (0.1 M)	20	54
7	FTO(+)–FTO(-)	Bu_4NPF_6 (0.05 M)	15	60
8	FTO(+)–FTO(-)	Bu_4NPF_6 (0.2 M)	26	68

[a] Carried out in dichloromethane (DCM, 5 mL) under air (1 atm). [b] Determined by ^1H NMR spectroscopy using 1,3,5-trimethoxybenzene as an internal standard.

Table S3. Screening of reaction concentration and current density.[a]

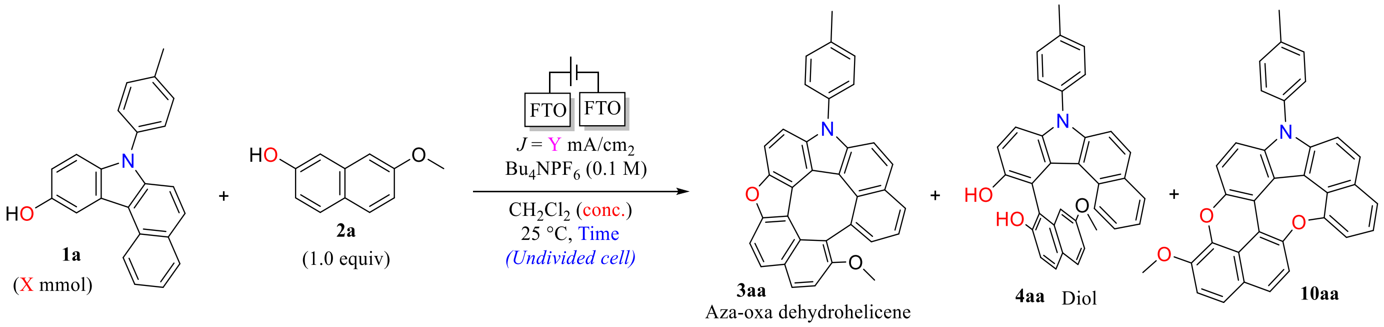

Entry	**1a** (X mmol)	J = Y mA/cm^2	Time (h)	**3aa** yield (%)[b]	**4aa** yield (%)[b]
1	0.025	0.4	3.0	22	63
2	0.05	0.4	6.5	25	64
3	0.1	0.4	14.0	28	68
4	0.1	0.8	12.5	32	64
5	0.1	1.2	10.0	37	60[c]
6	0.1	1.6	7.0	30	55[c]

[a] Carried out in dichloromethane DCM (5 mL) under air (1 atm). [b] Determined by ^1H NMR spectroscopy using 1,3,5-trimethoxybenzene as an internal standard. [c] Trace amounts of **10aa** were identified.

Table S4. Screening of different additives.[a]

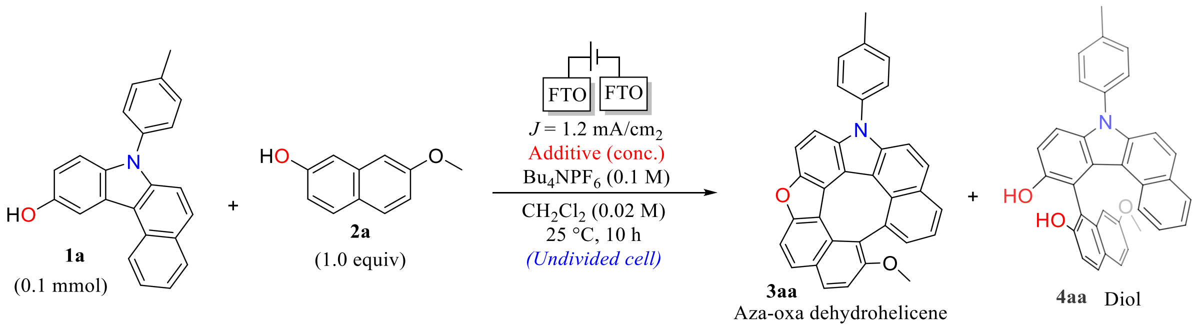

Entry	Additive	additive conc.	**3aa** yield (%)[b]	**4aa** yield (%)[b]
1	acetic acid	0.05 M	50	35
2	TFA	0.05 M	55	21
3	$BF_3.OEt_2$	0.05 M	57	28
4	$BF_3.OEt_2$	0.1 M	68	20
5	$BF_3.OEt_2$	0.2 M	84	0
6	$BF_3.OEt_2$	0.3 M	76	0

[a] Carried out in DCM (5 mL) in presence of additive (conc.) under air (1 atm).

[b] Determined by ^{1}H NMR spectroscopy using 1,3,5-trimethoxybenzene as an IS.

* The intramolecular C-C bond formation of **5aa** proceeded smoothly to afford **3aa** in 95% yield without using $BF_3.OEt_2$.

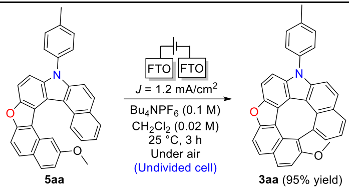

4. Supplementary Method 4: *general procedure for the sequential preparation of dehydro[7]helicenes* ***3***

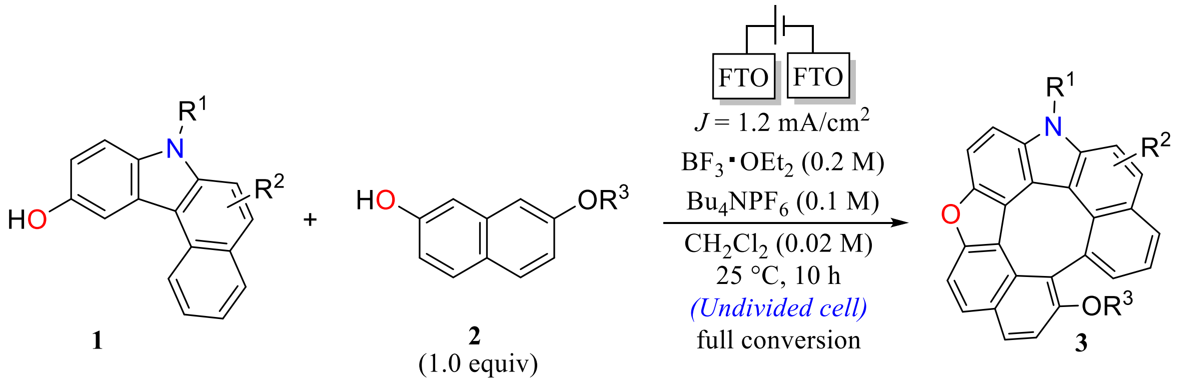

A solution of benzo[*c*]carbazol-10-ol derivatives **1** (0.1 mmol), 2-naphthols **2** (0.1 mmol), tetrabutylammonium hexafluorophosphate(V) (193.7 mg, 0.5 mmol) and $BF_3 \cdot EtO_2$ (0.2 M) in CH_2Cl_2 (5.0 mL) was transferred into an undivided electrolysis cell. This cell is equipped with two FTO electrodes (1.0 × 2.5 cm^2), which are connected to DC power supply. At rt, a constant current electrolysis with a current density of 1.20 mA/cm^2 was applied. After stirring for 10 h, the electrolysis was stopped and purification of the crude products by column chromatography (SiO_2, EtOAc/hexane) provided the desired dehydro[7]helicene **3**.

Dehydro[7]helicene **3aa**

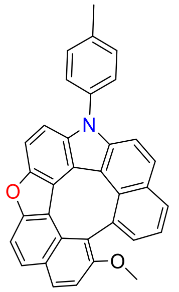

3aa (81% yield): a yellow solid.

^{1}H NMR (400 MHz, $CDCl_3$): δ 7.93 (dd, J = 7.6, 1.1 Hz, 1H), 7.89 (d, J = 8.7 Hz, 1H), 7.84 (d, J = 8.7 Hz, 1H), 7.80 (d, J = 8.7 Hz, 1H), 7.71 (d, J = 8.7 Hz, 1H), 7.53 (d, J = 9.4 Hz, 1H), 7.50 (d, J = 8.7 Hz, 1H), 7.41-7.45 (m, 4H), 7.31-7.35 (m, 3H), 7.25 (d, J = 8.7 Hz, 1H), 3.24 (s, 3H), 2.51 (s, 3H).

^{13}C NMR (100 MHz, $CDCl_3$): δ 161.99, 156.28, 151.31, 140.73, 138.33, 137.32, 136.95, 135.06, 132.66, 130.83, 130.75, 130.70, 129.73, 128.77, 128.40, 128.05, 128.00, 127.73, 126.84, 126.80, 122.24, 120.20, 118.85, 118.17, 117.48, 115.84, 112.23, 111.54, 108.15, 107.99, 58.47, 21.45.

HRMS (APCI): calcd for $C_{34}H_{22}NO_2$: m/z 476.1645 $[M + H]^+$, found 476.1638.

IR (KBr): 2955, 2925, 2853, 2321, 1516, 1457, 1296, 1269, 1020, 819 cm^{-1}.

mp: 310-312 °C (*n*-hexane/EtOAc).

11-(2-Hydroxy-7-methoxynaphthalen-1-yl)-7-(p-tolyl)-7*H*-benzo[c]carbazol-10-ol (**4aa**)

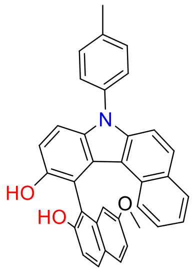

During the optimization of reaction conditions, intermediary diol **4aa** was formed as a yellow solid.

^{1}H NMR (600 MHz, $CDCl_3$): δ 8.01 (d, J = 8.9 Hz, 1H), 7.88 (d, J = 8.9 Hz, 1H), 7.76 (d, J = 6.9 Hz, 1H), 7.72 (d, J = 8.9 Hz, 1H), 7.52 (d, J = 9.3 Hz, 1H), 7.47-7.51 (m, 4H), 7.43 (d, J = 8.9 Hz, 1H), 7.29 (d, J = 8.9 Hz, 1H), 7.20 (d, J = 8.9 Hz, 1H), 7.14 (ddd, J = 7.6, 6.9, 1.4 Hz, 1H), 7.06 (dd, J = 8.9, 2.7 Hz, 1H), 6.88 (d, J = 2.7 Hz, 1H), 6.76 (d, J = 8.9 Hz, 1H), 6.67 (ddd, J = 7.6, 6.9, 1.4 Hz, 1H), 5.25 (s, 1H), 4.95 (s, 1H), 3.61 (s, 3H), 2.55 (s, 3H).

^{13}C NMR (150 MHz, $(CD_3)_2CO$): δ 159.22, 155.27, 150.99, 140.59, 139.05, 137.42, 136.76, 135.63, 131.54, 130.62, 130.53, 130.46, 130.38, 129.32, 128.99, 128.65, 126.34, 126.30, 125.68, 125.33, 122.92, 118.46, 117.37, 116.91, 115.49, 115.44, 114.95, 112.34, 111.84, 105.45, 55.03, 21.26.

HRMS (APCI): calcd for $C_{34}H_{26}NO_3$: m/z 496.1907 $[M + H]^+$, found 496.1904.

IR (KBr): 3498, 3331, 3059, 3032, 2940, 1624, 1516, 1276, 812, 747 cm^{-1}.

mp: 275- 277 °C (*n*-hexane/EtOAc).

2-Methoxy-10-(*p*-tolyl)-10*H*-benzo[c]naphtho[1',2':4,5]furo[3,2-g]carbazole (**5aa**)

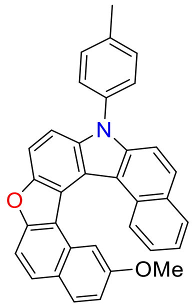

During the optimization of reaction conditions, intermediary helicene **5aa** was formed as a yellow solid.

^{1}H NMR (400 MHz, $CDCl_3$): δ 8.49 (d, J = 8.2 Hz, 1H), 7.94 (d, J = 7.8 Hz, 1H), 7.91 (d, J = 8.7 Hz, 1H), 7.85 (d, J = 8.9 Hz, 1H), 7.84 (d, J = 9.2 Hz, 1H), 7.73 (d, J = 8.7 Hz, 1H), 7.71 (d, J = 8.7 Hz, 1H), 7.68 (d, J = 2.5 Hz, 1H), 7.58 (d, J = 8.7 Hz, 1H), 7.47-7.54 (m, 5H), 7.32 (ddd, J = 7.8, 7.3, 0.9 Hz, 1H), 7.02 (ddd, J = 7.8, 7.6, 1.2 Hz, 1H), 6.97 (dd, J = 8.7, 2.7 Hz, 1H), 2.62 (s, 3H), 2.55 (s, 3H).

^{13}C NMR (100 MHz, $CDCl_3$): δ 154.58, 153.11, 139.16, 138.46, 138.30, 134.85, 130.77, 129.73, 129.50, 129.20, 128.57, 128.27, 128.13, 127.94, 127.91, 127.68, 124.86, 124.55, 124.25, 123.24, 120.17, 117.50, 117.40, 116.14, 112.61, 111.85, 109.29, 109.08, 29.85, 21.46 (Two carbons overlapped).

HRMS (ESI): calcd for $C_{34}H_{24}NO_2$: m/z 478.1802 $[M + H]^+$, found 478.1792.

IR (KBr): 3060, 2996, 2962, 2934, 1726, 1516, 1422, 1104, 1019, 803 cm^{-1}.

mp: 276-278 °C (*n*-hexane/EtOAc).

Dehydro[7]helicene **3ba**

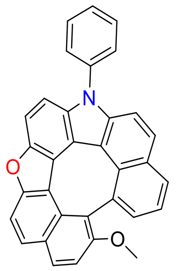

3ba (80% yield): a yellow solid.

^{1}H NMR (400 MHz, $CDCl_3$): δ 7.96 (dd, J = 7.2, 1.9 Hz, 1H), 7.93 (d, J = 8.7 Hz, 1H), 7.88 (d, J = 8.7 Hz, 1H), 7.83 (d, J = 9.2 Hz, 1H), 7.73 (d, J = 8.7 Hz, 1H), 7.65-7.69 (m, 2H), 7.56-7.62 (m, 4H), 7.54 (d, J = 8.7 Hz, 1H), 7.31-7.38 (m, 3H), 7.28 (d, J = 8.7 Hz, 1H), 3.27 (s, 3H).

^{13}C NMR (100 MHz, ($CDCl_3$): δ 161.96, 156.31, 151.35, 140.57, 137.77, 137.35, 136.79, 132.68, 130.86, 130.79, 130.13, 129.75, 128.83, 128.50, 128.38, 128.24, 127.97, 127.71, 126.80, 126.78, 122.32, 120.16, 118.89, 118.25, 117.58, 115.76, 112.16, 111.55, 108.09, 58.44 (One carbon overlapped).

HRMS (APCI): calcd for $C_{33}H_{20}NO_2$: *m/z* 462.1489 $[M + H]^+$, found 462.1483.

IR (KBr): 3054, 2957, 2934, 2835, 1712, 1595, 1501, 1425, 1297, 821 cm^{-1}.

mp: 312-314°C (*n*-hexane/EtOAc).

Dehydro[7]helicene **3ca**

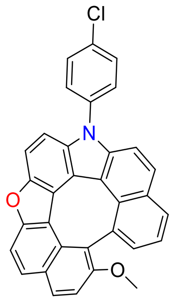

3ca (85% yield): a yellow solid.

^{1}H NMR (600 MHz, $CDCl_3$): 7.95 (dd, J = 7.2, 1.7 Hz, 1H), 7.92 (d, J = 8.2 Hz, 1H), 7.87 (d, J = 8.9 Hz, 1H), 7.83 (d, J = 8.9 Hz, 1H), 7.72 (d, J = 8.9 Hz, 1H), 7.62 (d, J = 8.9 Hz, 2H), 7.52-7.55 (m, 3H), 7.49 (d, J = 8.9 Hz, 1H), 7.32-7.37 (m, 2H), 7.30 (d, J = 8.9 Hz, 1H), 7.27 (d, J = 8.9 Hz, 1H), 3.28 (s, 3H).

^{13}C NMR (150 MHz, $CDCl_3$): δ 161.93, 156.35, 151.41, 140.36, 137.44, 136.60, 136.32, 134.10, 132.73, 130.91, 130.84, 130.40, 129.77, 129.52, 128.94, 128.70, 127.83, 127.65, 126.73, 122.48, 120.04, 118.98, 118.39, 117.81, 115.61, 111.79, 111.54, 108.22, 107.74, 58.35 (One carbon overlapped).

HRMS (APCI): calcd for $C_{33}H_{19}ClNO_2$: *m/z* 496.1099 $[M + H]^+$, found 496.1092.

IR (KBr): 3054, 2955, 2926, 2853, 1606, 1496, 1259, 1091, 819, 785 cm^{-1}.

mp: 311-313 °C (*n*-hexane/EtOAc).

Dehydro[7]helicene **3da**

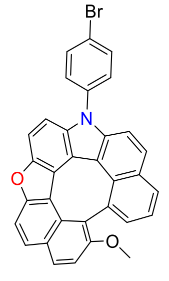

3da (79% yield): a yellow solid.

^{1}H NMR (400 MHz, $CDCl_3$): δ 7.92-7.97 (m, 2H), 7.89 (d, J = 8.7 Hz, 1H), 7.85 (d, J = 9.2 Hz, 1H), 7.80 (d, J = 8.7 Hz, 2H), 7.73 (d, J = 8.7 Hz, 1H), 7.57 (d, J = 9.2 Hz, 1H), 7.49-7.51 (m, 3H), 7.32-7.36 (m, 3H), 7.29 (d, J = 8.7 Hz, 1H), 3.28 (s, 3H).

^{13}C NMR (100 MHz, $CDCl_3$): δ 161.97, 156.38, 151.45, 140.32, 137.46, 136.88, 136.56, 133.40, 132.76, 131.03, 130.94, 130.84, 129.86, 129.78, 128.96, 128.72, 127.87, 127.68, 126.76, 122.50, 122.07, 120.06, 119.02, 118.45, 117.88, 115.67, 111.79, 111.56, 108.25, 107.76, 58.38.

HRMS (APCI): calcd for $C_{33}H_{19}BrNO_2$: *m/z* 540.0594 $[M + H]^+$, found 540.0585.

IR (KBr): 3054, 2957, 2928, 2854, 1731, 1605, 1493, 1259, 1022, 819 cm^{-1}.

mp: 344-346 °C (*n*-hexane/EtOAc).

Dehydro[7]helicene **3ea**

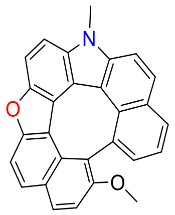

3ea (78% yield): a yellow solid.

^{1}H NMR (400 MHz, $CDCl_3$): δ 7.96 (dd, J = 7.4, 1.7 Hz, 1H), 7.90-7.93 (m, 2H), 7.87 (d, J = 9.2 Hz, 1H), 7.73 (d, J = 7.3 Hz, 1H), 7.70 (d, J = 7.3 Hz, 1H), 7.64 (d, J = 8.7 Hz, 1H), 7.47 (d, J = 8.7 Hz, 1H), 7.28-7.35 (m, 3H), 4.05 (s, 3H), 3.24 (s, 3H).

^{13}C NMR (150 MHz, $CDCl_3$): δ 161.95, 156.22, 150.93, 140.39, 137.30, 136.50, 132.37, 130.72, 130.30, 129.78, 128.77, 128.34, 128.13, 127.76, 126.92, 126.80, 122.02, 120.12, 118.95, 117.82, 117.10, 115.91, 111.53, 110.95, 107.73, 106.71, 58.51, 30.18.

HRMS (APCI): calcd for $C_{28}H_{18}NO_2$: *m/z* 400.1332 [M + H]$^+$, found 400.1333.

IR (KBr): 3039, 2957, 2927, 2853, 1518, 1460, 1259,1018, 819, 782 cm^{-1}.

mp: 300-302 °C (*n*-hexane/EtOAc).

Dehydro[7]helicene **3fa**

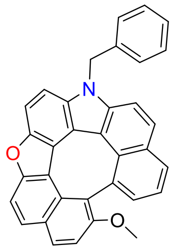

3fa (86% yield): a yellow solid.

^{1}H NMR (400 MHz, $CDCl_3$): δ 7.95 (dd, J = 7.6, 1.6 Hz, 1H), 7.92 (d, J = 8.7 Hz, 1H), 7.87 (d, J = 8.7 Hz, 2H), 7.72 (d, J = 8.7 Hz, 1H), 7.65 (d, J = 9.2 Hz, 1H), 7.59 (d, J = 8.7 Hz, 1H), 7.41 (d, J = 8.7 Hz, 1H), 7.32-7.37 (m, 2H), 7.23-7.30 (m, 4H), 7.11-7.13 (m, 2H), 5.73 (s, 2H), 3.27 (s, 3H).

^{13}C NMR (100 MHz, $CDCl_3$): δ 161.90, 156.30, 151.10, 139.93, 137.33, 137.20, 135.89, 132.47, 130.78, 130.53, 129.78, 128.98, 128.84, 128.56, 128.00, 127.73, 127.69, 126.90, 126.77, 126.37, 122.13, 120.09, 119.05, 118.03, 117.34, 115.62, 111.55, 111.24, 108.01, 107.14, 58.37, 47.18.

HRMS (APCI): calcd for $C_{34}H_{22}NO_2$: *m/z* 476.1645 [M + H]$^+$, found 476.1640.

IR (KBr): 3057, 2956, 2925, 1517, 1376, 1184, 1091, 819, 832, 755 cm^{-1}.

mp: 320-322 °C (*n*-hexane/EtOAc).

Dehydro[7]helicene **3ga**

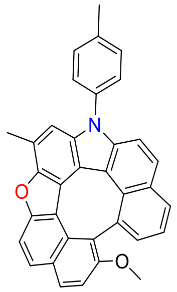

3ga (76% yield): a yellow solid.

^{1}H NMR (600 MHz, $CDCl_3$): δ 7.95 (dd, J = 7.2, 1.7 Hz, 1H), 7.91 (d, J = 8.9 Hz, 1H), 7.86 (d, J = 8.9 Hz, 1H), 7.79 (d, J = 8.9 Hz, 1H), 7.75 (d, J = 8.9 Hz, 1H), 7.45-7.55 (m, 5H), 7.35 (dd, J = 7.2, 1.7 Hz, 1H), 7.33 (d, J = 7.6 Hz, 1H), 7.27 (d, J = 8.2 Hz, 1H), 7.16 (s, 1H), 3.24 (s, 3H), 2.70 (s, 3H), 2.54 (s, 3H).

^{13}C NMR (150 MHz, $CDCl_3$): δ 161.99, 156.09, 150.37, 140.22, 138.23, 137.20, 137.14, 135.21, 132.67, 130.81, 130.69, 129.67, 128.57, 128.10, 128.05, 127.94, 127.66, 126.81, 126.77, 122.16, 120.48, 118.62, 118.23, 117.58, 116.27, 115.89, 112.19, 111.59, 108.66, 58.51, 21.46, 16.11 (One carbon overlapped).

HRMS (APCI): calcd for $C_{35}H_{24}NO_2$: *m/z* 490.1802 [M + H]$^+$, found 490.1801.

IR (KBr): 3036, 2955, 2925, 2854, 1516, 1456, 1292, 1258, 1017, 819 cm^{-1}.

mp: 317-319 °C (*n*-hexane/EtOAc).

Dehydro[7]helicene **3ha**

3ha (79% yield): a yellow solid.

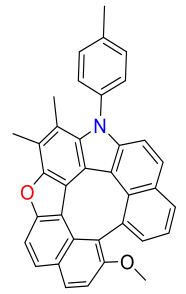

¹H NMR (600 MHz, $CDCl_3$): δ 7.92 (dd, J = 6.9, 2.1 Hz, 1H), 7.89 (d, J = 8.2 Hz, 1H), 7.83 (d, J = 8.9 Hz, 1H), 7.74 (d, J = 6.9 Hz, 1H), 7.72 (d, J = 6.9 Hz, 1H), 7.62-7.45 (m, 1H), 7.29-7.44 (m, 5H), 7.25 (d, J = 8.9 Hz, 1H), 7.23 (d, J = 8.9 Hz, 1H), 3.21 (s, 3H), 2.62 (s, 3H), 2.53 (s, 3H), 2.06 (s, 3H).

¹³C NMR (150 MHz, $CDCl_3$): δ 161.75, 155.57, 150.89, 142.18, 138.57, 137.98, 137.04, 136.27, 132.82, 130.86, 130.57, 130.09, 129.61, 128.12, 127.92, 127.83, 127.55, 126.71, 126.60, 122.15, 120.82, 118.31, 117.76, 117.33, 116.95, 116.00, 115.86, 112.63, 111.59, 58.58, 21.52, 15.58, 12.63 (One carbon overlapped).

HRMS (APCI): calcd for $C_{36}H_{26}NO_2$: m/z 504.1958 $[M + H]^+$, found 504.1957.

IR (KBr): 3043, 2953, 2924, 2854, 1602, 1514, 1167, 1019, 818, 750 cm^{-1}.

mp: 335-337 °C (*n*-hexane/EtOAc).

Dehydro[7]helicene **3ia**

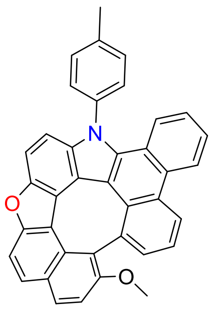

3ia (80% yield): a yellow solid.

¹H NMR (600 MHz, $CDCl_3$): δ 8.67 (d, J = 8.2 Hz, 1H), 8.58 (d, J = 7.6 Hz, 1H), 7.94 (d, J = 8.2 Hz, 1H), 7.87 (d, J = 8.9 Hz, 1H), 7.72-7.74 (m, 2H), 7.54-7.59 (m, 2H), 7.50 (d, J = 8.9 Hz, 1H), 7.42 (dd, J = 8.3, 1.4 Hz, 1H), 7.39 (t, J = 7.9 Hz, 1H), 7.31 (dd, J = 8.3, 2.1 Hz, 1H), 7.27-7.29 (m, 2H), 7.23 (dd, J = 6.9, 1.4 Hz, 1H), 7.06 (d, J = 8.9 Hz, 1H), 6.90 (dd, J = 7.9, 2.4 Hz, 1H), 3.37 (s, 3H), 2.56 (s, 3H).

¹³C NMR (150 MHz, $CDCl_3$): δ 161.34, 156.23, 151.96, 139.14, 138.29, 137.14, 136.87, 135.59, 132.80, 132.27, 131.00, 130.76, 130.56, 129.56, 129.30, 128.81, 128.43, 127.65, 127.59, 127.50, 126.33, 126.27, 125.72, 124.77, 123.73, 123.39, 123.12, 122.54, 120.46, 118.42, 118.35, 116.32, 114.99, 111.55, 108.68, 107.75, 58.20, 21.61.

HRMS (APCI): calcd for $C_{38}H_{24}NO_2$: *m/z* 526.1802 $[M + H]^+$, found 526.1792.

IR (KBr): 3052, 2955, 2925, 2854, 1733, 1595, 1501, 1426, 1296, 821 cm^{-1}.

mp: 325-327 °C (*n*-hexane/EtOAc).

Dehydro[7]helicene **3ja**

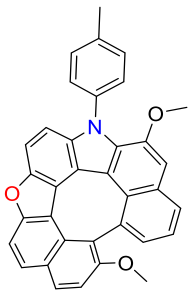

3ja (82% yield): a yellow solid.

¹H NMR (600 MHz, $CDCl_3$): δ 7.91 (d, J = 8.2 Hz, 1H), 7.86 (d, J = 8.9 Hz, 1H), 7.83 (d, J = 7.6 Hz, 1H), 7.71 (d, J = 8.9 Hz, 1H), 7.51 (d, J = 8.9 Hz, 1H), 7.26-7.34 (m, 6H), 7.18 (d, J = 7.6 Hz, 1H), 7.13 (d, J = 8.9 Hz, 1H), 7.11 (s, 1H), 3.79 (s, 3H), 3.27 (s, 3H), 2.51 (s, 3H).

¹³C NMR (150 MHz, $CDCl_3$): δ 161.87, 156.25, 151.44, 147.43, 138.18, 137.77, 137.38, 135.04, 132.70, 132.08, 131.91, 130.59, 129.31, 128.64, 128.53, 128.46, 128.13, 127.96, 127.81, 126.67, 123.17, 122.76, 120.28, 119.60, 118.76, 118.08, 115.85, 111.52, 108.85, 108.25, 105.18, 58.54, 55.53, 21.51.

HRMS (APCI): calcd for $C_{35}H_{24}NO_3$: *m/z* 506.1751 $[M + H]^+$, found 506.1744.

IR (KBr): 3035, 2957, 2925, 2854, 1606, 1514, 1299, 1259, 1038, 819 cm^{-1}.

mp: 319-321 °C (*n*-hexane/EtOAc).

Dehydro[7]helicene **3ka**

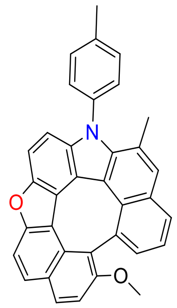

3ka (80% yield): a yellow solid.

^{1}H NMR (600 MHz, $CDCl_3$): δ 7.91 (d, J = 8.2 Hz, 1H), 7.85-7.87 (m, 2H), 7.72 (d, J = 8.2 Hz, 1H), 7.56 (s, 1H), 7.53 (d, J = 7.6 Hz, 1H), 7.50 (d, J = 8.2 Hz, 1H), 7.40 (d, J = 8.2 Hz, 1H), 7.36 (d, J = 8.2 Hz, 1H), 7.27-7.31 (m, 3H), 7.24 (d, J = 8.2 Hz, 1H), 7.03 (d, J = 8.9 Hz, 1H), 3.24 (s, 3H), 2.53 (s, 3H), 2.16 (s, 3H).

^{13}C NMR (150 MHz, $CDCl_3$): δ 161.84, 156.20, 151.47, 140.03, 139.02, 138.51, 137.18, 136.40, 132.29, 130.80, 130.58, 130.03, 129.83, 129.46, 129.41, 128.59, 128.53, 128.12, 127.75, 126.63, 125.98, 122.88, 122.33, 120.34, 118.60, 118.18, 117.78, 115.83, 111.52, 108.67, 107.93, 58.50, 21.53, 20.21.

HRMS (APCI): calcd for $C_{35}H_{24}NO_2$: *m/z* 490.1802 $[M + H]^+$, found 490.1794.

IR (KBr): 3047, 2959, 2935, 2844, 1723, 1585, 1505, 1429, 1298, 827 cm^{-1}.

mp: 311-313 °C (*n*-hexane/EtOAc).

Dehydro[7]helicene **3la**

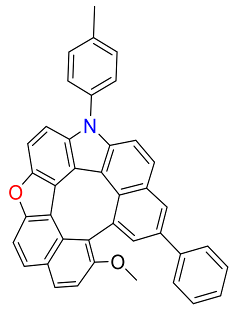

3la (84% yield): a yellow solid.

^{1}H NMR (400 MHz, $CDCl_3$): δ 8.18 (s, 1H), 7.95 (d, J = 8.7 Hz, 1H), 7.89 (dd, J = 8.9, 1.6 Hz, 2H), 7.75 (d, J = 8.7 Hz, 1H), 7.64-7.69 (m, 3H), 7.56 (td, J = 8.3, 0.9 Hz, 2H), 7.41-7.50 (m, 6H), 7.36 (d, J = 9.6 Hz, 1H), 7.29-7.33 (m, 2H), 3.32 (s, 3H), 2.54 (s, 3H).

^{13}C NMR (150 MHz, $CDCl_3$): δ 162.04, 156.38, 151.35, 140.87, 140.80, 138.40, 137.01, 136.89, 135.03, 134.75, 133.23, 131.17, 130.89, 130.74, 128.87, 128.84, 128.73, 128.00, 127.87, 127.75, 127.44, 127.22, 127.03, 126.77, 126.06, 120.09, 118.85, 118.06, 117.41, 115.50, 112.66, 111.59, 108.20, 108.11, 58.36, 21.48.

HRMS (APCI): calcd for $C_{40}H_{26}NO_2$: *m/z* 552.1958 $[M + H]^+$, found 552.1955.

IR (KBr): 3031, 2962, 2925, 2835, 1604, 1515, 1437, 1259, 1022, 796 cm^{-1}.

mp: 338-340 °C (*n*-hexane/EtOAc).

Dehydro[7]helicene **3ma**

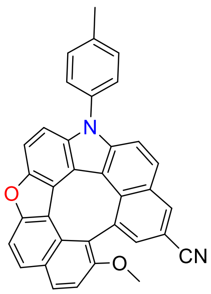

3ma (67% yield): a yellow solid.

^{1}H NMR (400 MHz, $CDCl_3$): δ 8.26 (d, J = 1.4 Hz, 1H), 7.99 (d, J = 8.7 Hz, 1H), 7.89 (d, J = 8.7 Hz, 1H), 7.83 (d, J = 8.7 Hz, 1H), 7.74 (d, J = 8.7 Hz, 1H), 7.60 (d, J = 8.7 Hz, 1H), 7.59 (d, J = 9.2 Hz, 1H), 7.54 (d, J = 1.6 Hz, 1H), 7.47 (d, J = 7.3 Hz, 4H), 7.34 (d, J = 8.7 Hz, 1H), 7.31 (d, J = 8.7 Hz, 1H), 3.54 (s, 3H), 2.54 (s, 3H).

^{13}C NMR (150 MHz, $CDCl_3$): δ 161.48, 156.59, 151.61, 144.08, 141.90, 138.94, 137.74, 137.08, 134.40, 134.05, 134.02, 131.76, 130.88, 129.15, 128.21, 127.24, 126.55, 125.56, 119.80, 119.63, 119.14, 118.91, 117.68, 117.46, 113.98, 113.59, 111.74, 108.90, 108.43, 104.78, 100.05, 57.46, 21.49.

HRMS (APCI): calcd for $C_{35}H_{21}N_2O_2$: *m/z* 501.1598 $[M + H]^+$, found 501.1601.

IR (KBr): 2954, 2925, 2870, 2853, 2224, 1712, 1600, 1516, 1260, 818 cm^{-1}.

mp: 340-342 °C (*n*-hexane/chloroform).

Dehydro[7]helicene **3pa**

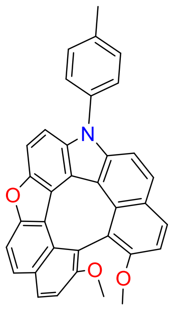

3pa (27% yield): a yellow solid.

^{1}H NMR (400 MHz, $CDCl_3$): *δ* 7.95 (d, *J* = 8.7 Hz, 1H), 7.91 (d, *J* = 8.7 Hz, 1H), 7.88 (d, *J* = 8.7 Hz, 1H), 7.76 (d, *J* = 9.2 Hz, 1H), 7.71 (d, *J* = 9.2 Hz, 1H), 7.54 (d, *J* = 8.7 Hz, 1H), 7.45 (s, 4H), 7.40 (d, *J* = 8.7 Hz, 1H), 7.31 (d, *J* = 8.7 Hz, 1H), 7.22 (d, *J* = 8.7 Hz, 1H), 7.15 (d, *J* = 8.7 Hz, 1H), 3.48 (s, 3H), 3.45 (s, 3H), 2.53 (s, 3H).

^{13}C NMR (150 MHz, $CDCl_3$): *δ* 162.32, 162.09, 156.55, 151.56, 141.07, 138.26, 136.80, 135.15, 131.07, 130.96, 130.67, 128.81, 128.45, 128.19, 128.01, 127.88, 127.15, 126.17, 121.40, 120.50, 119.88, 119.19, 118.20, 117.61, 113.80, 112.09, 111.29, 110.41, 107.69, 107.67, 57.95, 29.85, 21.46.

HRMS (APCI): calcd for $C_{35}H_{24}NO_3$: *m/z* 506.1751 [M + H]$^+$, found 506.1747.

IR (KBr): 2958, 2922, 2851, 1712, 1596, 1516, 1457, 1261, 1024, 815 cm^{-1}.

mp: 278-280 °C (*n*-hexane/EtOAc).

Dehydro[7]helicene **3ab**

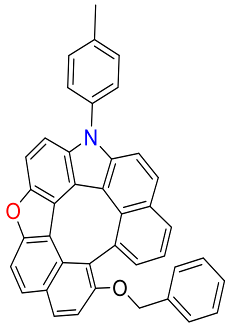

3ab (79% yield): a yellow solid.

^{1}H NMR (600 MHz, $CDCl_3$): *δ* 7.98 (d, *J* = 7.6 Hz, 1H), 7.91 (d, *J* = 8.9 Hz, 1H), 7.88 (d, *J* = 8.9 Hz, 1H), 7.84 (d, *J* = 8.9 Hz, 1H), 7.75 (d, *J* = 8.9 Hz, 1H), 7.57 (d, *J* = 8.9 Hz, 1H), 7.54 (d, *J* = 8.9 Hz, 1H), 7.45-7.48 (m, 5H), 7.34-7.36 (m, 2H), 7.33 (d, *J* = 8.9 Hz, 1H), 7.18-7.21 (m, 3H), 6.93 (dd, *J* = 7.6, 2.1 Hz, 2H), 4.46 (d, *J* = 11.7 Hz, 1H), 4.07 (d, *J* = 11.7 Hz, 1H), 2.54 (s, 3H).

^{13}C NMR (150 MHz, $CDCl_3$): *δ* 160.94, 156.26, 151.34, 140.77, 138.40, 137.66, 137.39, 136.97, 135.04, 132.47, 130.97, 130.82, 130.74, 129.85, 129.19, 128.78, 128.38, 128.21, 128.03, 127.74, 127.65, 127.48, 127.23, 126.93, 122.50, 120.33, 118.85, 118.17, 117.93, 117.46, 112.34, 111.81, 108.24, 108.07, 73.50, 21.48.

HRMS (APCI): calcd for $C_{40}H_{26}NO_2$: *m/z* 552.1958 [M + H]$^+$, found 552.1956.

IR (KBr): 3044, 2947, 2944, 2837, 1718, 1597, 1505, 1427, 1299, 823 cm^{-1}.

mp: 332-334 °C (*n*-hexane/EtOAc).

Dehydro[7]helicene **3ac**

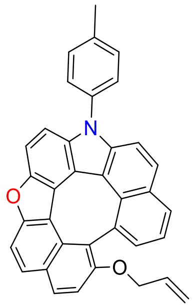

3ac (80% yield): a yellow solid.

^{1}H NMR (600 MHz, $CDCl_3$): *δ* 7.95 (d, *J* = 6.9 Hz, 1H), 7.91 (d, *J* = 8.2 Hz, 1H), 7.88 (d, *J* = 8.9 Hz, 1H), 7.83 (d, *J* = 8.9 Hz, 1H), 7.74 (d, *J* = 8.9 Hz, 1H), 7.56 (d, *J* = 8.9 Hz, 1H), 7.53 (d, *J* = 8.9 Hz, 1H), 7.45-7.50 (m, 4H), 7.40 (dd, *J* = 6.9, 1.4 Hz, 1H), 7.32-7.35 (m, 2H), 7.29 (d, *J* = 8.2 Hz, 1H), 5.58-5.64 (m, 1H), 4.96 (dq, *J* = 10.7, 1.6 Hz, 1H), 4.89 (dq, *J* = 17.2, 1.6 Hz, 1H), 3.95-3.98 (m, 1H), 3.67-3.70 (m, 1H), 2.54 (s, 3H).

^{13}C NMR (150 MHz, $CDCl_3$): *δ* 160.79, 156.25, 151.33, 140.73, 138.37, 137.53, 136.95, 135.06, 133.53, 132.47, 130.87, 130.72, 129.77, 128.93, 128.76, 128.39, 128.02, 127.71, 127.07, 126.86, 122.36, 120.28, 118.84, 118.15, 117.46, 117.35, 116.82, 112.25, 111.72, 108.19, 108.02, 72.16, 21.47 (One carbon overlapped).

HRMS (APCI): calcd for $C_{36}H_{24}NO_2$: *m/z* 502.1802 [M + H]$^+$, found 502.1794.

IR (KBr): 3038, 2961, 2924, 2855, 1725, 1604, 1516, 1260, 1018, 820 cm^{-1}.

mp: 346-348 °C (*n*-hexane/EtOAc).

Dehydro[7]helicene **3bb**

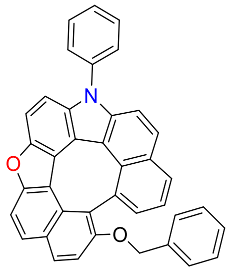

3bb (82% yield): a yellow solid.

^{1}H NMR (400 MHz, $CDCl_3$): δ 7.98 (d, J = 6.9 Hz, 1H), 7.91 (d, J = 8.7 Hz, 1H), 7.89 (d, J = 9.2 Hz, 1H), 7.85 (d, J = 9.2 Hz, 1H), 7.75 (d, J = 8.7 Hz, 1H), 7.54-7.69 (m, 7H), 7.48 (dd, J = 6.9, 0.9 Hz, 1H), 7.35-7.38 (m, 2H), 7.33 (d, J = 8.2 Hz, 1H), 7.18-7.20 (m, 3H), 6.92-6.94 (m, 2H), 4.47 (d, J = 11.4 Hz, 1H), 4.09 (d, J = 11.9 Hz, 1H).

^{13}C NMR (150 MHz, $CDCl_3$): δ 160.94, 156.29, 151.40, 140.63, 137.77, 137.69, 137.38, 136.84, 132.52, 131.03, 130.83, 130.15, 129.85, 129.15, 128.82, 128.45, 128.41, 128.26, 128.20, 127.74, 127.65, 127.46, 127.22, 126.92, 122.56, 120.31, 118.91, 118.29, 117.86, 117.61, 112.24, 111.81, 108.14, 73.47 (One carbon overlapped).

HRMS (APCI): calcd for $C_{39}H_{24}NO_2$: m/z 538.1802 $[M + H]^+$, found 538.1794.

IR (KBr): 3060, 3034, 2925, 2859, 1595, 1500, 1296, 1020, 820, 744 cm^{-1}.

mp: 301-303 °C (*n*-hexane/EtOAc).

Dehydro[7]helicene **3bc**

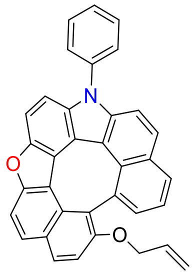

3bc (81% yield): a yellow solid.

^{1}H NMR (600 MHz, $CDCl_3$): δ 7.96 (d, J = 8.2 Hz, 1H), 7.92 (d, J = 8.9 Hz, 1H), 7.89 (d, J = 8.9 Hz, 1H), 7.84 (d, J = 8.9 Hz, 1H), 7.75 (d, J = 8.9 Hz, 1H), 7.67 (t, J = 7.9 Hz, 2H), 7.60-7.63 (m, 2H), 7.56-7.58 (m, 2H), 7.54 (d, J = 8.9 Hz, 1H), 7.41 (dd, J = 6.9, 1.4 Hz, 1H), 7.37 (d, J = 8.9 Hz, 1H), 7.34 (t, J = 7.2 Hz, 1H), 7.29 (d, J = 8.9 Hz, 1H), 5.61 (qd, J = 10.9, 5.2 Hz, 1H), 4.96 (dd, J = 11.7, 1.4 Hz, 1H), 4.89 (dd, J = 17.2, 1.4 Hz, 1H), 3.97 (dd, J = 12.7, 5.2 Hz, 1H), 3.70 (dd, J = 12.7, 5.2 Hz, 1H).

^{13}C NMR (150 MHz, $CDCl_3$) δ 160.79, 156.28, 151.38, 140.57, 137.77, 137.56, 136.80, 133.51, 132.51, 130.92, 130.74, 130.15, 129.79, 128.87, 128.82, 128.48, 128.40, 128.26, 127.70, 127.05, 126.84, 122.44, 120.27, 118.89, 118.25, 117.58, 117.29, 116.84, 112.17, 111.72, 108.12, 108.11, 72.13.

HRMS (APCI): calcd for $C_{35}H_{22}NO_2$: m/z 488.1645 $[M + H]^+$, found 488.1645.

IR (KBr): 3051, 2955, 2925, 2853, 1595, 1501, 1425, 1296, 821, 734 cm^{-1}.

mp: 317-319 °C (*n*-hexane/EtOAc).

Dehydro[7]helicene **3cb**

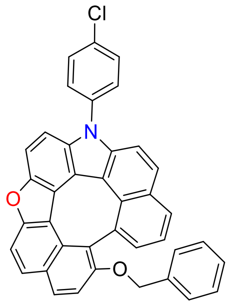

3cb (84% yield): a yellow solid.

^{1}H NMR (600 MHz, $CDCl_3$): δ 7.98 (dd, J = 8.3, 1.4 Hz, 1H), 7.91 (d, J = 8.2 Hz, 1H), 7.88 (d, J = 8.9 Hz, 1H), 7.86 (d, J = 9.6 Hz, 1H), 7.75 (d, J = 8.9 Hz, 1H), 7.64 (d, J = 8.9 Hz, 2H), 7.54-7.58 (m, 3H), 7.51 (d, J = 8.9 Hz, 1H), 7.47 (dd, J = 6.9, 1.4 Hz, 1H), 7.36 (t, J = 7.6 Hz, 1H), 7.32-7.33 (m, 2H), 7.18-7.21 (m, 3H), 6.92-6.93 (m, 2H), 4.48 (d, J = 11.7 Hz, 1H), 4.11 (d, J = 11.7 Hz, 1H).

^{13}C NMR (150 MHz, $CDCl_3$): δ 160.93, 156.34, 151.46, 140.43, 137.78, 137.32, 136.66, 136.31, 134.17, 132.57, 131.07, 130.88, 130.43, 129.88, 129.55, 128.99, 128.95, 128.66, 128.22, 127.70, 127.68, 127.42, 127.18, 126.84, 122.74, 120.20, 119.01, 118.44, 117.83, 117.75, 111.90, 111.81, 108.31, 107.85, 73.39.

HRMS (APCI): calcd for $C_{39}H_{23}ClNO_2$: m/z 572.1412 $[M + H]^+$, found 572.1413.

IR (KBr): 3033, 2955, 2925, 2855, 1605, 1495, 1292, 1091, 1017, 819 cm^{-1}.

mp: 324-326 °C (*n*-hexane/EtOAc).

Dehydro[7]helicene **3cc**

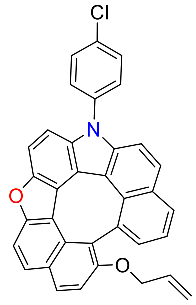

3cc (75% yield): a yellow solid.

^{1}H NMR (600 MHz, $CDCl_3$): δ 7.95 (d, *J* = 6.9 Hz, 1H), 7.91 (d, *J* = 8.9 Hz, 1H), 7.88 (d, *J* = 8.9 Hz, 1H), 7.85 (d, *J* = 8.9 Hz, 1H), 7.74 (d, *J* = 8.9 Hz, 1H), 7.64 (d, *J* = 8.9 Hz, 2H), 7.54-7.57 (m, 3H), 7.50 (d, *J* = 8.9 Hz, 1H), 7.40 (dd, *J* = 7.6, 1.4 Hz, 1H), 7.35 (d, *J* = 7.6 Hz, 1H), 7.31 (d, *J* = 8.9 Hz, 1H), 7.28 (d, *J* = 8.9 Hz, 1H), 5.58-5.64 (m, 1H), 4.96 (dd, *J* = 10.7, 1.7 Hz, 1H), 4.88 (dd, *J* = 17.2, 1.4 Hz, 1H), 3.98 (dq, *J* = 13.6, 1.9 Hz, 1H), 3.72 (qd, *J* = 12.0, 4.8 Hz, 1H).

^{13}C NMR (150 MHz, $CDCl_3$): δ 160.77, 156.33, 151.44, 140.37, 137.65, 136.63, 136.33, 134.15, 133.45, 132.57, 130.97, 130.80, 130.41, 129.81, 129.54, 128.93, 128.72, 128.68, 127.65, 127.02, 126.77, 122.60, 120.15, 119.00, 118.40, 117.81, 117.15, 116.84, 111.82, 111.72, 108.26, 107.80, 72.04.

HRMS (APCI): calcd for $C_{35}H_{21}ClNO_2$: *m/z* 522.1255 $[M + H]^+$, found 522.1247.

IR (KBr): 3049, 2955, 2925, 2855, 1496, 1425, 1298, 1091, 1016, 819 cm^{-1}.

mp: 314-316 °C (*n*-hexane/EtOAc).

Dehydro[7]helicene **3db**

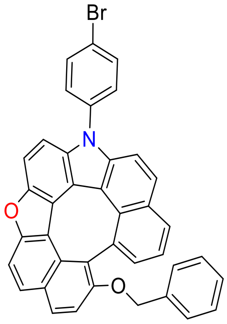

3db (81% yield): a yellow solid.

^{1}H NMR (600 MHz, $CDCl_3$): δ 7.98 (dd, *J* = 7.6, 1.4 Hz, 1H), 7.91 (d, *J* = 8.9 Hz, 1H), 7.87 (d, *J* = 8.2 Hz, 1H), 7.86 (d, *J* = 8.9 Hz, 1H), 7.78 (d, *J* = 8.9 Hz, 2H), 7.74 (d, *J* = 8.9 Hz, 1H), 7.56 (d, *J* = 7.9 Hz, 1H), 7.46-7.51 (m, 4H), 7.36 (t, *J* = 7.6 Hz, 1H), 7.31-7.33 (m, 2H), 7.18-7.21 (m, 3H), 6.92 (dd, *J* = 7.2, 2.4 Hz, 2H), 4.48 (d, *J* = 11.7 Hz, 1H), 4.11 (d, *J* = 11.7 Hz, 1H).

^{13}C NMR (150 MHz, $CDCl_3$) δ 160.90, 156.33, 151.45, 140.32, 137.77, 137.31, 136.83, 136.54, 133.40, 132.57, 131.07, 130.87, 129.87, 128.94, 128.65, 128.21, 127.67, 127.40, 127.15, 126.82, 122.74, 122.08, 120.17, 119.00, 118.45, 117.85, 117.70, 111.88, 111.79, 108.30, 107.82, 73.36 (Three carbons overlapped).

HRMS (APCI): calcd for $C_{39}H_{23}BrNO_2$: *m/z* 616.0907 $[M + H]^+$, found 616.0909.

IR (KBr): 3061, 3034, 2925, 2856, 1605, 1496, 1247, 1091, 1017, 819 cm^{-1}.

mp: 348-350 °C (*n*-hexane/EtOAc).

Dehydro[7]helicene **3dc**

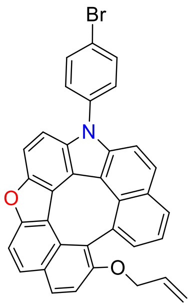

3dc (72% yield): a yellow solid.

^{1}H NMR (400 MHz, $CDCl_3$): δ 7.96 (dd, *J* = 7.6, 1.1 Hz, 1H), 7.92 (d, *J* = 8.2 Hz, 1H), 7.89 (d, *J* = 8.7 Hz, 1H), 7.85 (d, *J* = 8.7 Hz, 1H), 7.80 (d, *J* = 8.7 Hz, 2H), 7.74 (d, *J* = 9.2 Hz, 1H), 7.57 (d, *J* = 8.7 Hz, 1H), 7.49-7.52 (m, 3H), 7.41 (dd, *J* = 7.3, 1.4 Hz, 1H), 7.32-7.36 (m, 2H), 7.29 (d, *J* = 8.7 Hz, 1H), 5.56-5.66 (m, 1H), 4.96 (dq, *J* = 10.5, 1.5 Hz, 1H), 4.88 (dq, *J* = 17.3, 1.7 Hz, 1H), 3.98 (dtd, *J* = 12.8, 3.3, 1.7 Hz, 1H), 3.71 (dtd, *J* = 12.8, 3.4, 1.7 Hz, 1H).

^{13}C NMR (175 MHz, $CDCl_3$): δ 160.77, 156.33, 151.44, 140.30, 137.65, 136.85, 136.54, 133.41, 132.56, 131.06, 130.97, 130.80, 129.87, 129.82, 128.95, 128.70, 127.64, 127.01, 126.75, 122.62, 122.09, 120.14, 119.00, 118.43, 118.25, 117.84, 117.17, 116.85, 111.82, 111.72, 108.28, 107.80, 72.05.

HRMS (APCI): calcd for $C_{35}H_{21}BrNO_2$: *m/z* 566.0750 $[M + H]^+$, found 566.0751.

IR (KBr): 3050, 3017, 2925, 2854, 1605, 1496, 1298, 1091, 820, 733 cm^{-1}.

mp: 342-344 °C (*n*-hexane/EtOAc).

Dehydro[7]helicene **3eb**

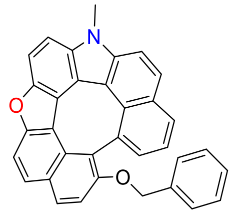

3eb (71% yield): a yellow solid.

^{1}H NMR (400 MHz, $CDCl_3$): δ 7.99 (dd, *J* = 7.6, 1.1 Hz, 1H), 7.94 (d, *J* = 8.7 Hz, 1H), 7.86-7.91 (m, 2H), 7.74 (d, *J* = 6.9 Hz, 1H), 7.72 (d, *J* = 6.9 Hz, 1H), 7.66 (d, *J* = 8.7 Hz, 1H), 7.49 (d, *J* = 8.7 Hz, 1H), 7.45 (dd, *J* = 7.3, 1.4 Hz, 1H), 7.34 (d, *J* = 7.8 Hz, 1H), 7.31 (d, *J* = 8.7 Hz, 1H), 7.17-7.21 (m, 3H), 6.91 (dd, *J* = 6.6, 2.5 Hz, 2H), 4.41 (d, *J* = 11.4 Hz, 1H), 4.06 (s, 3H), 4.04 (d, *J* = 11.9 Hz, 1H).

^{13}C NMR (150 MHz, $CDCl_3$): δ 160.91, 158.74, 156.20, 151.26, 150.99, 140.47, 137.65, 137.39, 136.72, 136.53, 132.21, 130.76, 130.13, 129.89, 129.16, 128.80, 128.30, 128.20, 127.93, 127.65, 127.50, 122.28, 118.08, 116.98, 114.77, 111.79, 111.04, 107.81, 106.78, 105.90, 73.52, 70.30.

HRMS (APCI): calcd for $C_{34}H_{22}NO_2$: *m/z* 476.1645 $[M + H]^+$, found 476.1639.

IR (KBr): 3062, 3033, 2926, 2854, 1702, 1625, 1515, 1455, 1018, 820 cm^{-1}.

mp: 335-337 °C (*n*-hexane/EtOAc).

Dehydro[7]helicene **3ec**

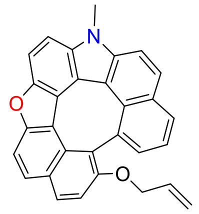

3ec (62% yield): a yellow solid.

^{1}H NMR (600 MHz, $CDCl_3$): δ 7.96 (dd, *J* = 7.6, 1.4 Hz, 1H), 7.92 (d, *J* = 8.9 Hz, 1H), 7.90 (d, *J* = 8.9 Hz, 1H), 7.87 (d, *J* = 8.9 Hz, 1H), 7.73 (d, *J* = 8.2 Hz, 1H), 7.71 (d, *J* = 8.9 Hz, 1H), 7.64 (d, *J* = 8.9 Hz, 1H), 7.46 (d, *J* = 8.9 Hz, 1H), 7.38 (dd, *J* = 6.9, 1.4 Hz, 1H), 7.31 (t, *J* = 7.6 Hz, 1H), 7.27 (d, *J* = 8.2 Hz, 1H), 5.56-5.62 (m, 1H), 4.94 (dq, *J* = 10.3, 1.4 Hz, 1H), 4.86 (dq, *J* = 17.2, 1.8 Hz, 1H), 4.04 (s, 3H), 3.91 (dtd, *J* = 12.7, 3.4, 1.7 Hz, 1H), 3.64-3.67 (dtd, *J* = 12.8, 3.3, 1.7 Hz, 1H).

^{13}C NMR (150 MHz, $CDCl_3$): δ 160.75, 156.19, 150.96, 140.36, 137.51, 136.49, 133.54, 132.21, 130.67, 130.37, 129.82, 129.06, 128.75, 128.30, 127.75, 127.09, 126.96, 122.14, 120.24, 118.96, 117.82, 117.52, 117.10, 116.88, 111.70, 110.96, 107.76, 106.74, 72.22, 30.17.

HRMS (APCI): calcd for $C_{30}H_{20}NO_2$: *m/z* 426.1489 $[M + H]^+$, found 426.1488.

IR (KBr): 3036, 2925, 2888, 2855, 1604, 1517, 1257, 1144, 816, 782 cm^{-1}.

mp: 323-325 °C (*n*-hexane/EtOAc).

Dehydro[7]helicene **3fb**

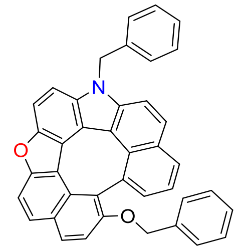

3fb (77% yield): a yellow solid.

^{1}H NMR (600 MHz, $CDCl_3$): δ 7.98 (dd, J = 7.6, 1.4 Hz, 1H), 7.91 (d, J = 8.9 Hz, 1H), 7.88 (t, J = 8.9 Hz, 2H), 7.74 (d, J = 8.9 Hz, 1H), 7.66 (d, J = 8.9 Hz, 1H), 7.59 (d, J = 8.9 Hz, 1H), 7.47 (dd, J = 6.9, 1.4 Hz, 1H), 7.42 (d, J = 8.9 Hz, 1H), 7.35 (d, J = 7.6 Hz, 1H), 7.32 (d, J = 8.2 Hz, 1H), 7.23-7.27 (m, 3H), 7.18-7.19 (m, 3H), 7.13 (d, J = 6.2 Hz, 2H), 6.92 (dd, J = 7.2, 2.4 Hz, 2H), 5.73 (s, 2H), 4.46 (d, J = 11.7 Hz, 1H), 4.10 (d, J = 11.7 Hz, 1H).

^{13}C NMR (150 MHz, $CDCl_3$): δ 160.85, 156.28, 151.14, 139.98, 137.64, 137.34, 137.18, 135.93, 132.32, 130.82, 130.68, 129.88, 129.19, 129.00, 128.83, 128.80, 128.50, 128.19, 127.71, 127.65, 127.48, 127.22, 127.04, 126.39, 122.38, 120.26, 119.07, 118.08, 117.80, 117.37, 111.79, 111.31, 108.07, 107.22, 73.39, 47.23.

HRMS (APCI): calcd for $C_{40}H_{26}NO_2$: *m/z* 552.1958 $[M + H]^+$, found 552.1951.

IR (KBr): 3060, 3031, 2925, 1624, 1516, 1378, 1183, 1001, 831, 754 cm^{-1}.

mp: 309-311 °C (*n*-hexane/EtOAc).

Dehydro[7]helicene **3fc**

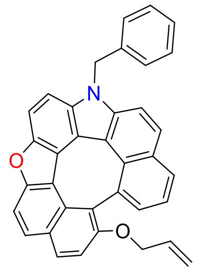

3fc (79% yield): a yellow solid.

^{1}H NMR (600 MHz, $CDCl_3$): δ 7.95 (dd, J = 7.6, 1.4 Hz, 1H), 7.91 (d, J = 8.2 Hz, 1H), 7.88 (d, J = 8.9 Hz, 1H), 7.87 (d, J = 9.3 Hz, 1H), 7.73 (d, J = 8.2 Hz, 1H), 7.66 (d, J = 8.9 Hz, 1H), 7.59 (d, J = 8.2 Hz, 1H), 7.42 (d, J = 8.9 Hz, 1H), 7.40 (dd, J = 7.4, 1.7 Hz, 1H), 7.32 (t, J = 7.6 Hz, 2H), 7.25-7.29 (m, 3H), 7.13 (d, J = 6.9 Hz, 2H), 5.75 (s, 2H), 5.57-5.64 (m, 1H), 4.96 (dq, J = 10.3, 1.4 Hz, 1H), 4.88 (dq, J = 17.2, 1.6 Hz, 1H), 3.94-3.98 (m, 1H), 3.69-3.72 (m, 1H).

^{13}C NMR (175 MHz, $CDCl_3$): δ 160.69, 156.25, 151.10, 139.92, 137.51, 137.18, 135.89, 133.47, 132.28, 130.73, 130.56, 129.82, 129.00, 128.87, 128.81, 128.53, 127.70, 127.03, 126.94, 126.37, 122.25, 120.18, 119.04, 118.03, 117.34, 117.19, 116.87, 111.71, 111.24, 108.03, 107.18, 72.04, 47.22 (One carbon overlapped).

HRMS (APCI): calcd for $C_{36}H_{24}NO_2$: *m/z* 502.1802 $[M + H]^+$, found 502.1802.

IR (KBr): 3060, 3032, 2925, 2855, 1604, 1518, 1453, 1216, 819, 781 cm^{-1}.

mp: 322-324 °C (*n*-hexane/EtOAc).

5. **Supplementary Method 5:** *general procedure for the two-pot synthesis of dehydro[7]helicene* ***3ba***

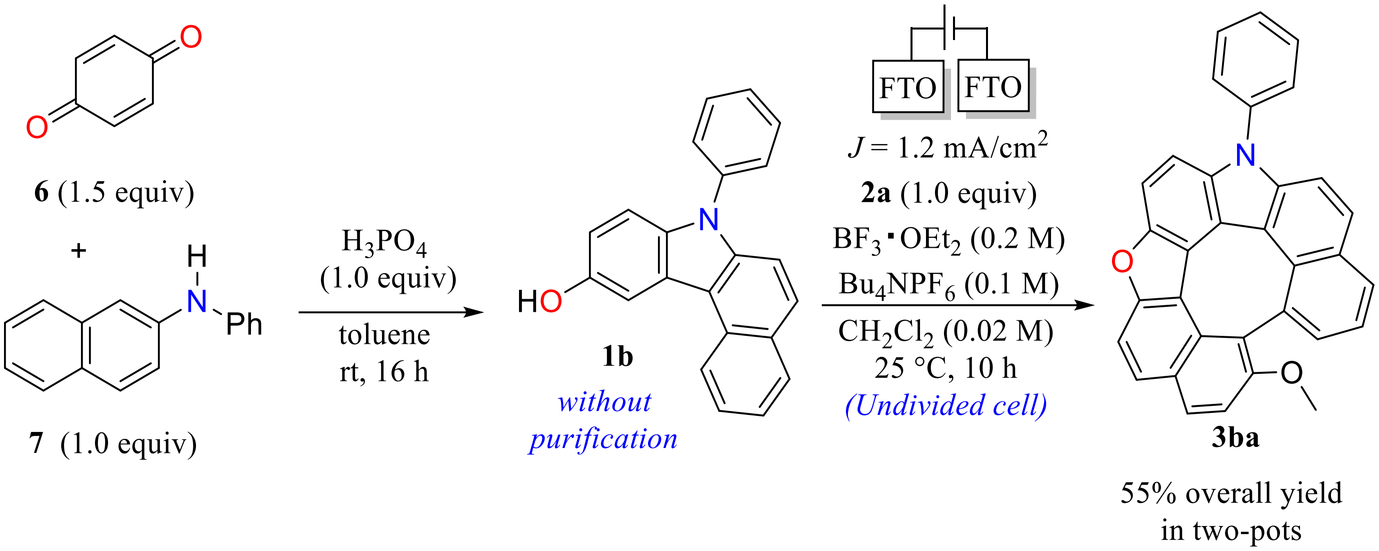

A mixture of **6** (23.36 mg, 0.216 mmol), and **7** (31.56 mg, 0.144 mmol) were dissolved in dry toluene (0.8 mL). To this mixture, orthophosphoric acid (7.6 µL, 14.12 mg, 0.144 mmol) dissolved in toluene (0.64 mL) was added dropwise. After stirring for 16 h at 25 °C under N_2 atmosphere, the reaction was quenched by water and extracted with EtOAc. The combined organic extracts were washed with water, dried over Na_2SO_4, filtered, and evaporated *in vacuo* to afford benzo[*c*]carbazol-10-ol derivative **1b** as a white solid. The crude was used directly without any further purification with 7-MeO-2-naphthol derivative **2a** (17.4 mg, 0.1 mmol), and tetrabutylammonium hexafluorophosphate(V) (193.7 mg, 0.5 mmol) in the presence of $BF_3 \cdot OEt_2$ (0.2 M) CH_2Cl_2 (5 mL). The reaction mixture was transferred into an undivided electrolysis cell equipped with two FTO electrodes, which are connected to DC power supply. At rt, a constant current electrolysis with a current density of 1.2 mA/cm^2 was performed. After stirring for 10 h, the electrolysis was stopped and purification of the crude products by column chromatography (SiO_2, EtOAc/hexane) provided **3ba** as a yellow solid in 55% overall yield in two steps.

6. Supplementary Method 6: *derivatization of dehydro[7]helicenes 3*

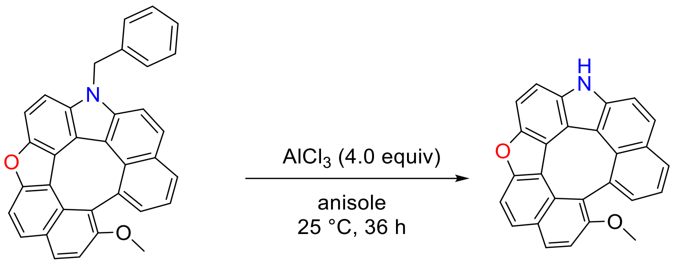

3fa **3na**, 67%

A solution of **3fa** (22.38 mg, 0.05 mmol) in anisole (1 mL) was added to a suspension of $AlCl_3$ (26.67 mg, 0.2 mmol) in anisole (0.5 mL) at 0 °C under nitrogen atmosphere. After stirring at 25 °C for 72 h, the mixture was poured into water and extracted with ethyl acetate. The organic layer was washed successively with 5% $NaHCO_3$, brine, dried over Na_2SO_4 and evaporated to dryness *in vacuo*. The residue was purified on column chromatography (SiO_2, EtOAc/ hexane) to give **3na** (67% yield) as a yellow solid.

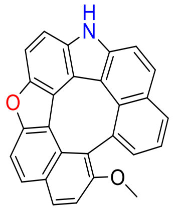

^{1}H NMR (600 MHz, $CDCl_3$): δ 8.44 (s, 1H), 7.92 (d, J = 6.9 Hz, 1H), 7.89 (d, J = 8.2 Hz, 1H), 7.84 (d, J = 8.9 Hz, 1H), 7.78 (d, J = 8.9 Hz, 1H), 7.70 (d, J = 8.9 Hz, 1H), 7.55 (d, J = 8.9 Hz, 1H), 7.52 (d, J = 8.9 Hz, 1H), 7.36 (d, J = 8.2 Hz, 1H), 7.29-7.33 (m, 2H), 7.25 (d, J = 8.9 Hz, 1H), 3.24 (s, 3H).

^{13}C NMR (150 MHz, $CDCl_3$): δ 161.88, 156.13, 150.93, 139.34, 137.29, 134.88, 132.59, 130.77, 130.62, 129.77, 128.76, 128.40, 127.91, 127.59, 126.78, 126.76, 122.12, 120.02, 118.78, 118.50, 117.82, 115.63, 112.86, 111.52, 108.66, 107.94, 58.38.

HRMS (APCI): calcd for $C_{27}H_{16}NO_2$: m/z 386.1176 $[M + H]^+$, found 386.1170.

IR (KBr): 3409, 3045, 2960, 2934, 2836, 1605, 1518, 1257, 1020, 820 cm^{-1}.

mp: 313-315 °C (*n*-hexane/EtOAc).

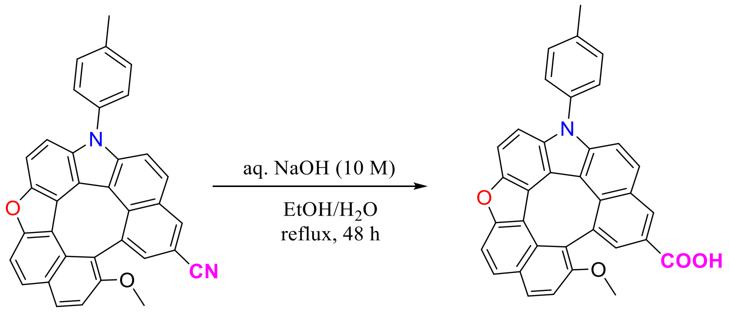

3ma **3oa** (65%)

An aqueous solution of NaOH (10 M, 1 mL) was added to ethanol solution (1 mL) of **3ma** (25.03 mg, 0.05 mmol). After refluxing for 48 h, the solution was acidified by (1 M) HCl, and extracted with EtOAc. The organic layer was dried over Na_2SO_4, filtered, and concentrated *in vacuo*. The residue was purified by column chromatography (SiO_2, EtOAc/hexane) to give **3oa** (65% yield) as a yellow solid.

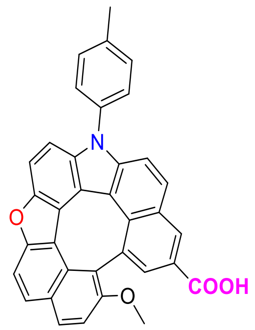

^{1}H NMR (400 MHz, $CDCl_3$): δ 8.51 (d, J = 1.8 Hz, 1H), 7.96 (d, J = 8.7 Hz, 1H), 7.90 (d, J = 9.2 Hz, 1H), 7.89 (d, J = 9.4 Hz, 1H), 7.74 (d, J = 8.7 Hz, 1H), 7.71 (d, J = 1.8 Hz, 1H), 7.58 (d, J = 8.7 Hz, 1H), 7.57 (d, J = 8.9 Hz, 1H), 7.45-7.49 (m, 4H), 7.35 (d, J = 8.7 Hz, 1H), 7.30 (d, J = 8.7 Hz, 1H), 3.40 (s, 3H), 2.54 (s, 3H).

^{13}C NMR (175 MHz, $CDCl_3$) δ 169.25, 161.73, 156.47, 151.47, 141.65, 138.69, 136.98, 134.69, 134.63, 133.07, 131.22, 130.81, 130.25, 129.73, 129.20, 129.03, 128.97, 127.93, 127.48, 127.06, 126.64, 126.14, 119.82, 118.83, 117.86, 117.37, 114.82, 113.30, 111.68, 108.49, 108.33, 58.05, 21.50.

HRMS (APCI): calcd for $C_{34}H_{22}NO_4$: m/z 520.1543 $[M + H]^+$, found 520.1533.

IR (KBr): 3368, 2955, 2925, 2853, 1657, 1603, 1515, 1022, 817, 782 cm^{-1}.

mp: 319-321°C (n-hexane/EtOAc).

c- Synthesis of aza-dioxa[8]circulene 8

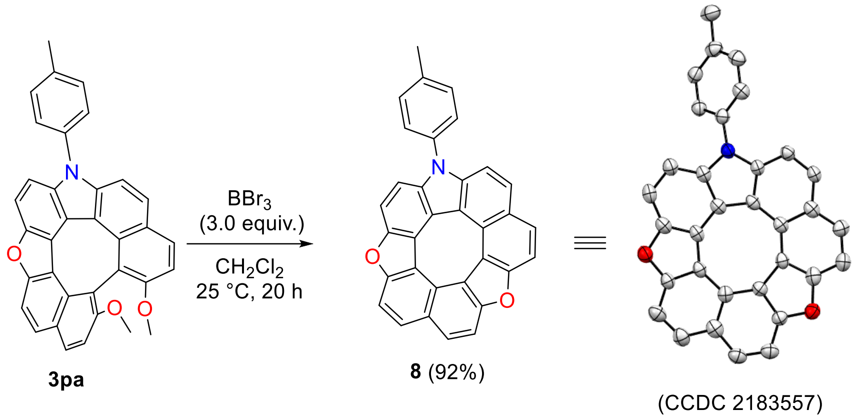

To a dry round bottom flask, **3pa** (50.5 mg, 0.1 mmol) was charged. The reaction flask was evacuated and purged with argon three times. After **3pa** was dissolved in dry dichloromethane (40 mL), 1.0 M boron tribromide solution in dichloromethane (0.3 mL, 0.3 mmol, 3.0 equiv.) was added via syringe at 0 °C, and the reaction mixture was stirred at room temperature for 20 h. After quenched with saturated aqueous sodium bicarbonate solution, the mixture was extracted with THF three times and the combined organic layers were washed with water, brine and dried over anhydrous sodium sulfate. After removal of the solvents *in vacuo*, the residue was purified by column chromatography on silica with ethyl acetate/n-hexane as eluent to give product **8** as a yellowish white solid in 92% yield.

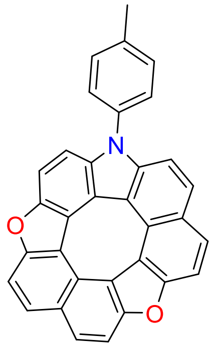

^{1}H NMR (700 MHz, $CDCl_3$): δ 8.31 (m, 3H), 8.21 (d, J = 9.0 Hz, 1H), 8.06 (d, J = 8.6 Hz, 1H), 8.03 (d, J = 8.4 Hz, 1H), 8.02 (d, J = 8.6 Hz, 1H), 7.93 (d, J = 8.6 Hz, 1H), 7.72 (d, J = 8.6 Hz, 1H), 7.69 (d, J = 8.6 Hz, 1H), 7.60 (d, J = 7.7 Hz, 2H), 7.54 (d, J = 8.2 Hz, 2H), 2.59 (s, 3H).

^{13}C NMR (175 MHz, $CDCl_3$) δ 155.96, 155.87, 155.56, 151.15, 141.15, 138.76, 136.72, 134.88, 131.00, 130.84, 130.75, 130.42, 130.40, 128.68, 127.39, 126.51, 123.02, 122.14, 117.61, 117.17, 116.82, 115.69, 114.58, 114.52, 110.86, 110.60, 110.02, 109.59, 108.71, 108.46, 21.56.

HRMS (APCI): calcd for $C_{33}H_{18}NO_2$: m/z 460.1332 $[M + H]^+$, found 460.1328.

IR (KBr): 2956, 2923, 2869, 2850, 1604, 1516, 1262, 1099, 1026, 817 cm^{-1}.

mp: 295-297 °C (n-hexane/chloroform).

7. Supplementary Method 7: *stepwise enantioselective synthesis*

7-1. Effects of vanadium complexes

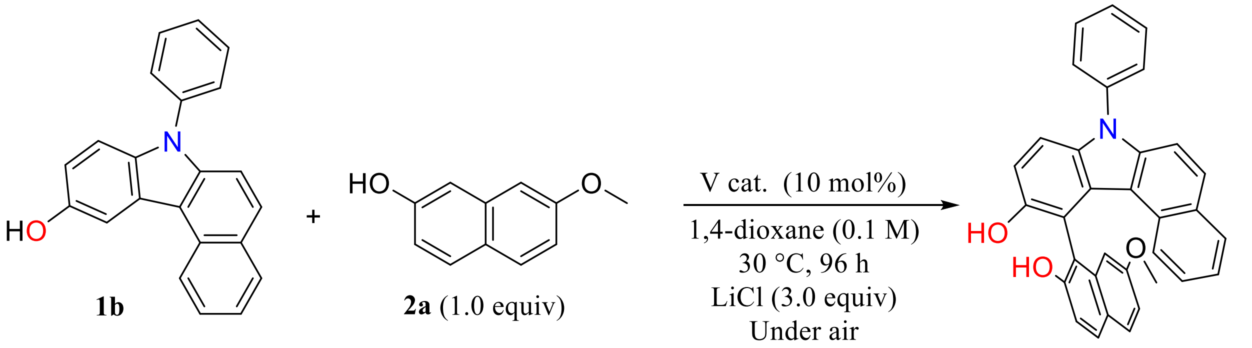

1b + **2a** (1.0 equiv) → (*R*)-**4ba**

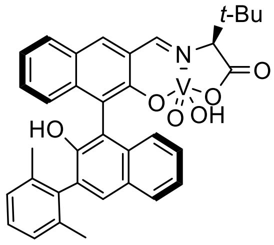

(*R*$_a$,*S*)-**9a**

85% yield, 50%ee

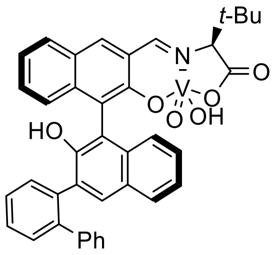

(*R*$_a$,*S*)-**9b**

74% yield, 35%ee

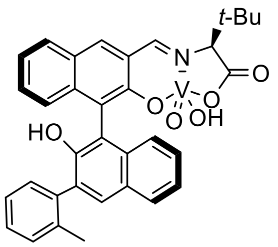

(*R*$_a$,*S*)-**9c**

77% yield, 32%ee

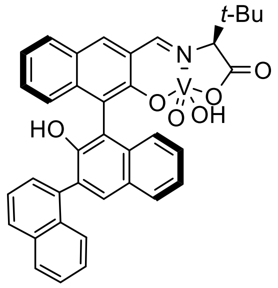

(*R*$_a$,*S*)-**9d**

83% yield, 34%ee

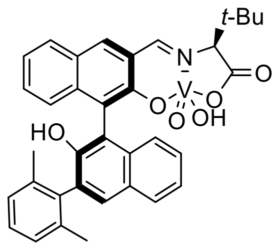

(*S*$_a$,*S*)-**9a**

72% yield, 33%ee

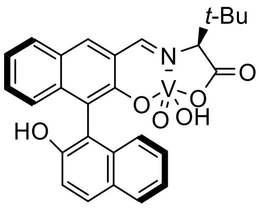

(*R*$_a$,*S*)-**9e**

81% yield, 11%ee

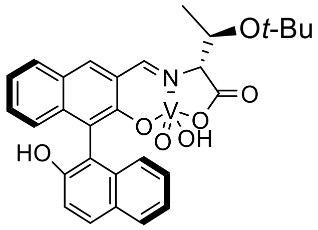

(*R*$_a$,*R*,*R*)-**9f**

69% yield, 9%ee

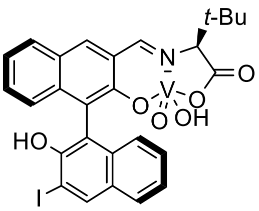

(*R*$_a$,*S*)-**9g**

75% yield, 16%ee

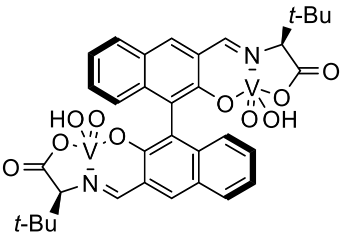

(*R*$_a$,*S*,*S*)-**9h**

80% yield, 20%ee

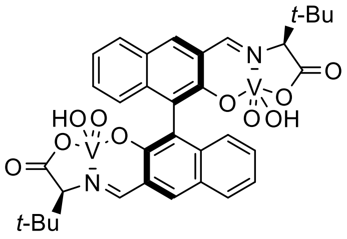

(*S*$_a$,*S*,*S*)-**9h**

82% yield, 25%ee

7-2. Effects of temperatures and solvents

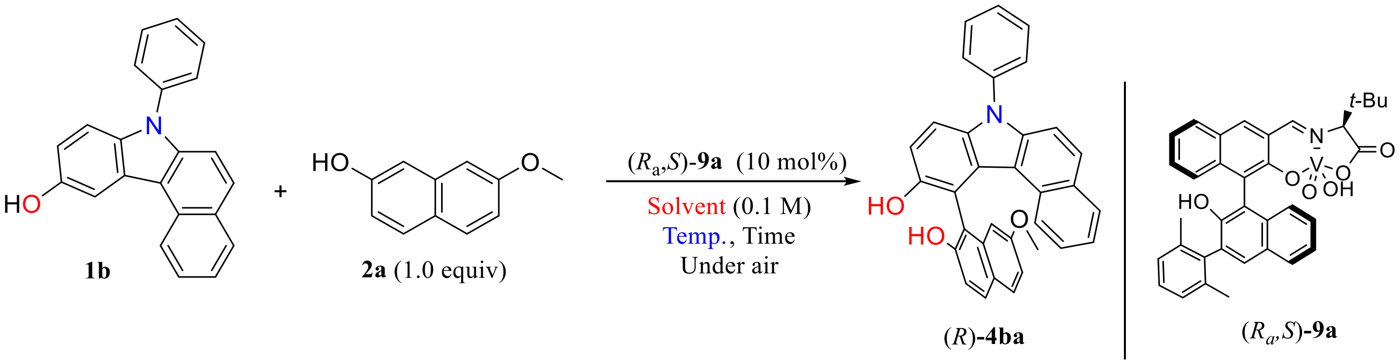

Entry	Temp.	Solvent	Time	(*R*)-**4ba** yield (%)[b]	(*R*)-**4ba** ee (%)[c]
1	10 °C	$ClCH_2CH_2Cl$	24 h	45	36
2	20 °C	$ClCH_2CH_2Cl$	24 h	56	34
3	30 °C	$ClCH_2CH_2Cl$	24 h	60	33
4	30 °C	$ClCH_2CH_2Cl$	48 h	84	33
5	40 °C	$ClCH_2CH_2Cl$	24 h	71	30
6[d]	50 °C	$ClCH_2CH_2Cl$	24 h	75	25
7	30 °C	$Cl_2C{=}CCl_2$	48 h	75	39
8	30 °C	PhCl	48 h	81	39
9	30 °C	1,4-dioxane	48 h	85	44
10	30 °C	toluene	48 h	80	43
11	30 °C	*o*-xylene	48 h	80	Rac.
12	30 °C	*p*-xylene	48 h	81	10
13	30 °C	THF	48 h	65	35
14	30 °C	Et_2O	48 h	60	29
15	30 °C	EtOAc	48 h	75	42
16	30 °C	CH_2Cl_2	48 h	90	36
17	30 °C	$CHCl_3$	48 h	87	36
18[d]	30 °C	CCl_4	48 h	83	59
19[e]	0 °C	CCl_4	48 h	57	59
20	-10 °C	CCl_4	96 h	45	61

[a] Determined by ^{1}H NMR spectroscopy using 1,3,5-trimethoxybenzene as an internal standard. [b] Determined by HPLC (Daicel Chiralpak IB, hexane/EtOH = 2/1, flow rate 1.0 mL/min, T = 25 ºC, 280 nm): t_{maj} = 5.6 min, t_{min} = 6.9 min. [c] Trace amount of helicene **5ba** was formed.

Channel Name 280.0nm

#	Peak Name	CH	tR [min]	Area [μV·sec]	Height [μV]	Area%	Height%	Quantity	NTP	Resolution	Symmetry Factor	Warning
1	Unknown	9	5.628	496949	38252	50.016	54.410	N/A	5069	3.897	1.490	
2	Unknown	9	6.983	496625	32051	49.984	45.590	N/A	5372	N/A	1.573	

Chromatogram Name Ik 1186 N-Ph 7MeO diol H EtOH 2 1 IB-CH9

Sample Name

Channel Name 280.0nm

#	Peak Name	CH	tR [min]	Area [μV·sec]	Height [μV]	Area%	Height%	Quantity	NTP	Resolution	Symmetry Factor	Warning
1	Unknown	9	5.682	18599043	1377129	79.709	82.620	N/A	4660	3.893	1.774	
2	Unknown	9	7.113	4734579	289685	20.291	17.380	N/A	4948	N/A	1.719	

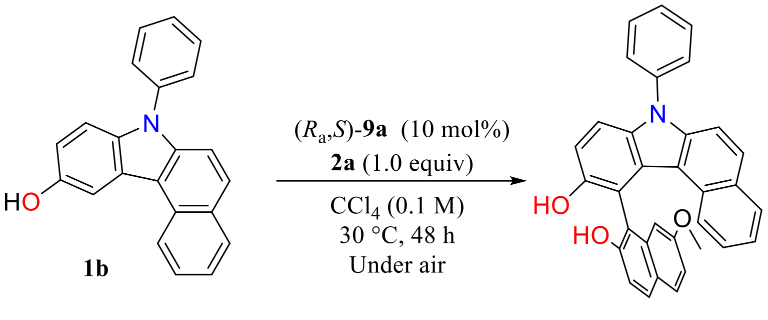

(R)-**4ba**
83%, 59% ee

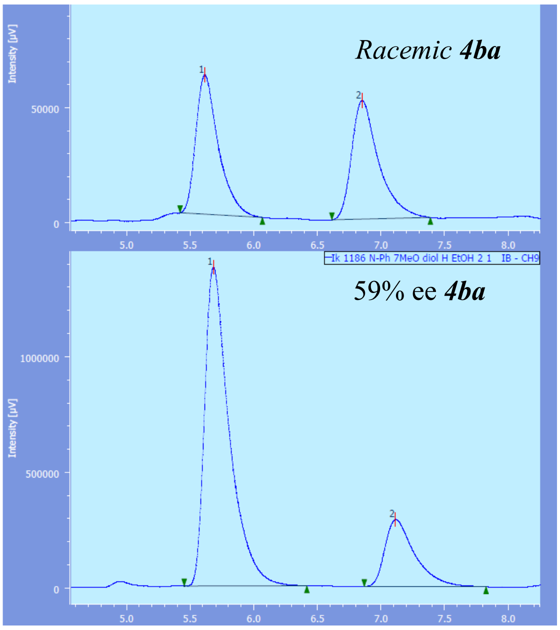

Determined by HPLC (Daicel Chiralpak IB, hexane/EtOH = 2/1, flow rate 1.0 mL/min, T = 25 ºC, 280 nm): t_{maj} = 5.6 min, t_{min} = 6.9 min

7-3. General procedures for the asymmetric synthesis 3

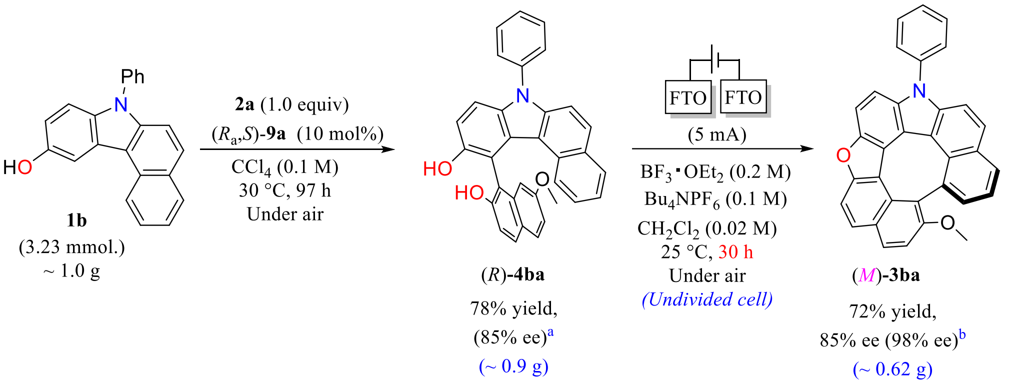

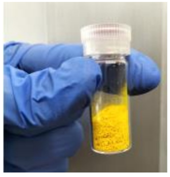

First step:

A test tube was charged with carbazole **1b** (1.0 g, 3.23 mmol, 1.0 equiv), 7-methoxy-2-naphthol **2a** (563 mg, 3.23 mmol, 1.0 equiv), mononuclear vanadium catalyst (R_a,S)-**9a** (10 mol %), and CCl_4 (0.1 M) under air at 30 °C. After stirring for 97 h, The reaction mixture was then filtered through a short pad of silica-gel and the solvent was evaporated. The residue was purified by silica-gel column chromatography to afford diol (R)-**4ba** in 78% isolated yield and 59% ee. [(R)-**4ba** in 85%ee after recrystallization from hexane/$CHCl_3$].

11-(2-Hydroxy-7-methoxynaphthalen-1-yl)-7-phenyl-7H-benzo[c]carbazol-10-ol (**4ba**)

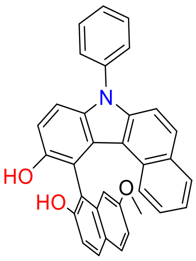

^{1}H NMR (400 MHz, $(CD_3)_2CO$): δ 7.98 (d, J = 8.7 Hz, 1H), 7.85 (d, J = 8.7 Hz, 1H), 7.74-7.79 (m, 4H), 7.62-7.69 (m, 3H), 7.43 (d, J = 7.8 Hz, 1H), 7.41 (d, J = 7.3 Hz, 1H), 7.25 (d, J = 8.7 Hz, 1H), 7.22 (d, J = 8.7 Hz, 1H), 7.07 (ddd, J = 7.6, 7.3, 1.2 Hz, 1H), 6.92 (dd, J = 8.9, 2.5 Hz, 2H), 6.74 (d, J = 2.7 Hz, 1H), 6.61 (ddd, J = 7.8, 7.6, 1.6 Hz, 1H), 3.42 (s, 3H).

^{13}C NMR (100 MHz, $(CD_3)_2CO$): δ 159.23, 155.25, 151.06, 140.47, 138.32, 137.42, 136.65, 131.05, 130.64, 130.51, 130.47, 130.43, 129.33, 129.22, 129.16, 128.73, 126.37, 126.32, 125.80, 125.33, 122.98, 118.44, 117.51, 116.89, 115.54, 115.45, 115.02, 112.26, 111.78, 105.44, 55.03.

HRMS (APCI): calcd for $C_{33}H_{24}NO_3$: *m/z* 482.1751 $[M + H]^+$, found 482.1750.

IR (KBr): 3537, 3481, 3053, 3038, 2952, 1620, 1511, 1160, 1030, 882 cm^{-1}.

mp: 265-267 °C.

Second step:

A solution of (*R*)-**4ba** (0.9 g, 1.87 mmol), tetrabutylammonium hexafluorophosphate(V) (3.68 g, 9.5 mmol), and BF_3・OEt_2 (0.2 M) in CH_2Cl_2 (95 mL, 0.02 M) was transferred into an undivided electrolysis cell. This cell is equipped with two FTO electrodes (5*4 cm), which are connected to DC power supply. At rt, constant current electrolysis with a current intensity of 5.0 mA and a current density of 0.25 mA/cm^2 was performed. After 30 h., the electrolysis was stopped and purification of the crude products by column chromatography (SiO_2, EtOAc/hexane) provided 0.62 g of dehydrohelicene (*M*)-**3ba** as yellow solid in 72% yield and 85% ee [(*M*)-**3ba** in 98%ee after recrystallization from hexane/EtOAc].

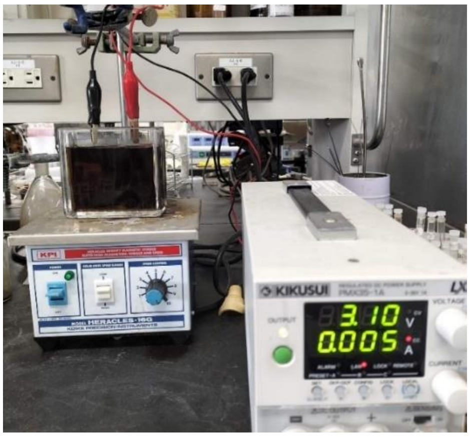

7-4. Second step (0.1 mmol scale) without using BF_3・OEt_2

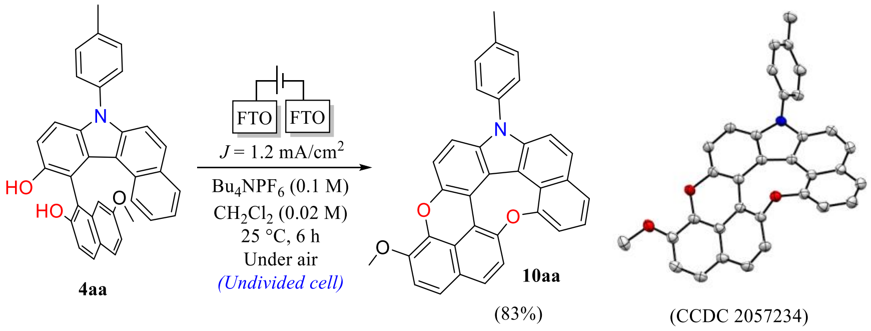

A solution of **4aa** (0.1 mmol), tetrabutylammonium hexafluorophosphate(V) (193.7 mg, 0.5 mmol) in CH_2Cl_2 (0.02 M) was transferred into an undivided electrolysis cell. This cell is equipped with two FTO electrodes, which are connected to DC power supply. At 25 °C temperature, a constant current electrolysis with a current density of 1.2 mA/cm^2 was performed. After 6 h, the electrolysis was stopped and a purification of the crude products by column chromatography (SiO_2, EtOAc/hexane) provided **10aa** (83% yield) as a yellow solid.

8-Methoxy-17-(*p*-tolyl)-7,13-dioxa-17-aza-3,4-methanobenzo[*jk*]naphtho[1',8':6,7,8]cycloocta[1,2,3,4-def]phenanthrene (**10aa**)

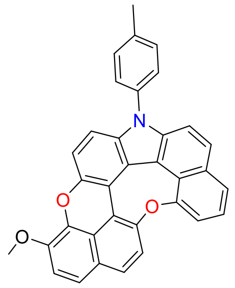

^{1}H NMR (400 MHz, $CDCl_3$): δ 7.81 (d, J = 7.8 Hz, 1H), 7.72-7.74 (m, 2H), 7.65 (d, J = 9.2 Hz, 1H), 7.56 (d, J = 8.7 Hz, 1H), 7.32-7.48 (m, 8H), 7.30 (d, J = 8.7 Hz, 1H), 7.18 (d, J = 9.2 Hz, 1H), 4.02 (s, 3H), 2.52 (s, 3H).

^{13}C NMR (100 MHz, $CDCl_3$): δ 152.78, 151.28, 149.45, 141.32, 140.00, 138.99, 138.82, 138.73, 134.15, 131.53, 130.79, 127.45, 127.04, 126.61, 125.79, 125.61, 123.31, 121.36, 120.94, 119.80, 119.20, 118.43, 116.39, 115.16, 114.57, 114.38, 112.48, 112.33, 57.43, 21.45 (Two carbons overlaped)

HRMS (APCI): calcd for $C_{34}H_{22}NO_3$: *m/z* 492.1594 $[M + H]^+$, found 492.1593.

IR (KBr): 3060, 2954, 2925, 2853, 1724, 1604, 1515, 1249, 822, 806 cm^{-1}.

mp: 310-312 °C (*n*-hexane/EtOAc).

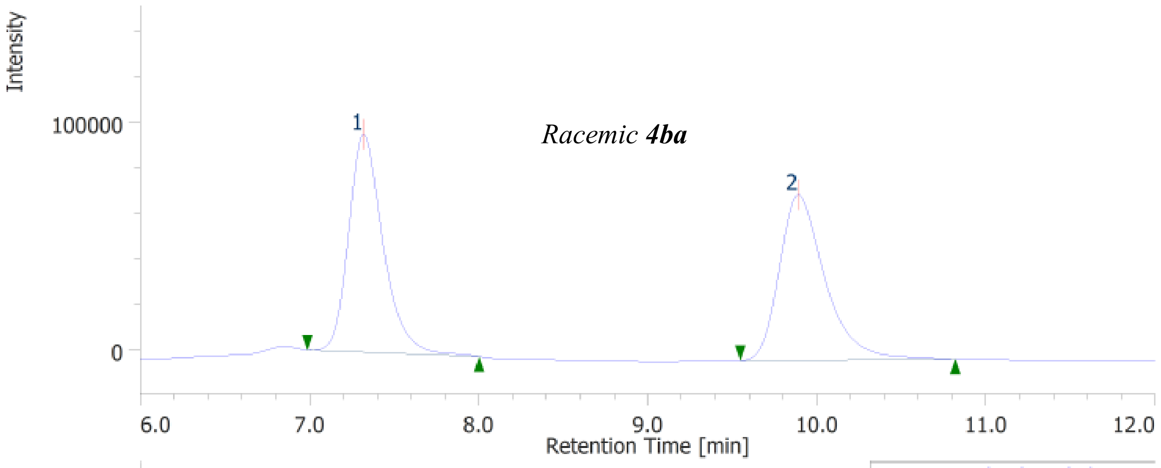

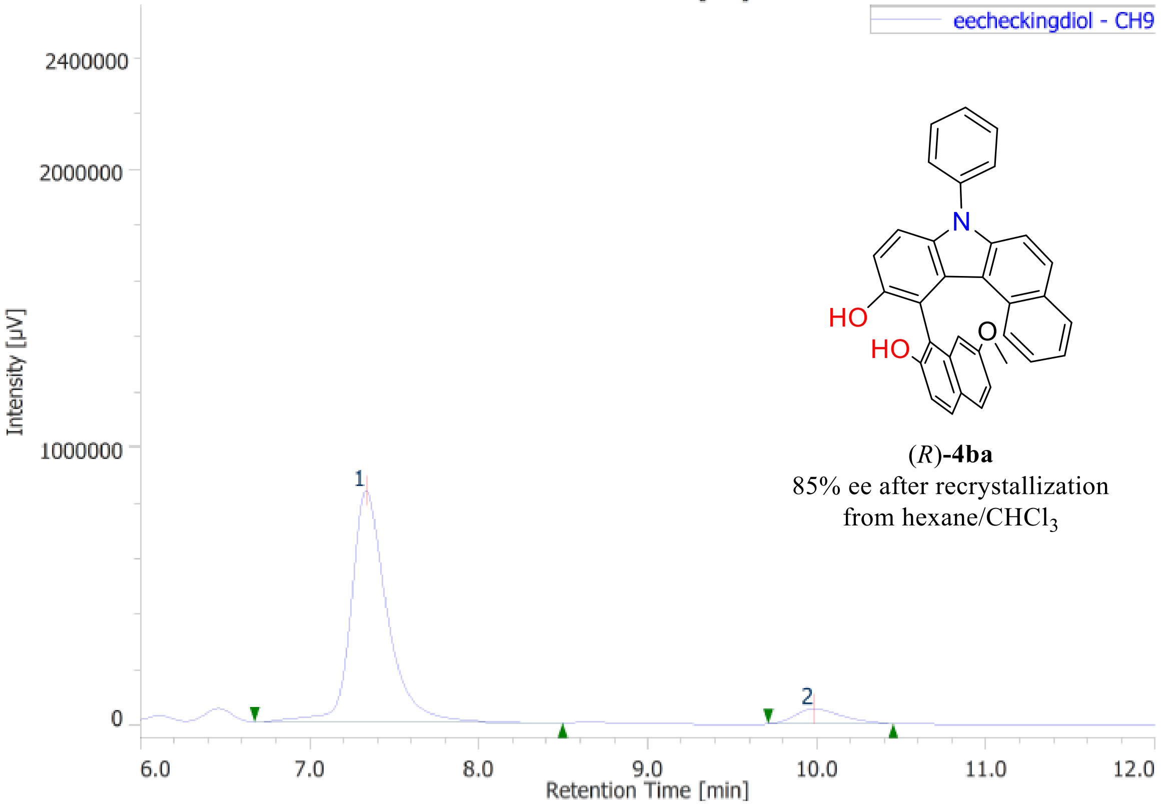

Channel Name 235.0nm

#	Peak Name	CH	tR [min]	Area [µV·sec]	Height [µV]	Area%	Height%	Quantity	NTP	Resolution	Symmetry Factor
1	Unknown	9	7.320	1356777	95771	49.950	56.893	N/A	6720	6.228	1.289
2	Unknown	9	9.890	1359477	72564	50.050	43.107	N/A	7073	N/A	1.401

Chromatogram Name eecheckingdiol-CH9
Sample Name
Channel Name 235.0nm

#	Peak Name	CH	tR [min]	Area [µV·sec]	Height [µV]	Area%	Height%	Quantity	NTP	Resolution	Symmetry Factor
1	Unknown	9	7.333	12376068	832388	92.342	93.952	N/A	6910	6.146	1.391
2	Unknown	9	9.983	1026375	53579	7.658	6.048	N/A	6088	N/A	1.345

Determined by HPLC (Daicel Chiralpak IB, hexane/EtOH = 3/1, flow rate 1.0 mL/min, T = 25 ºC, 235 nm): t_{maj} = 7.3 min, t_{min} = 9.9 min.

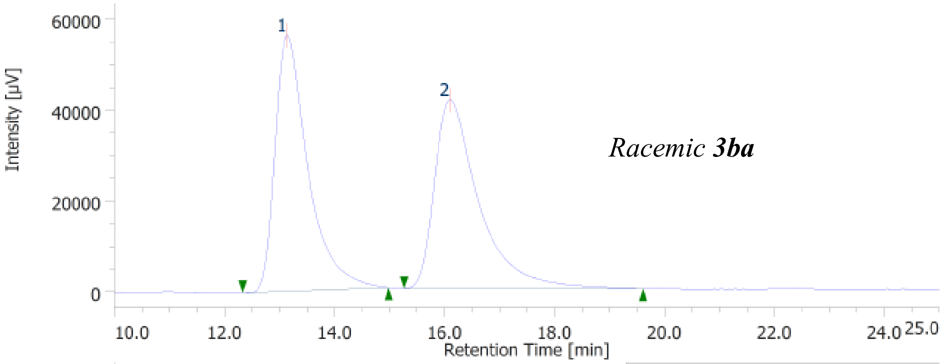

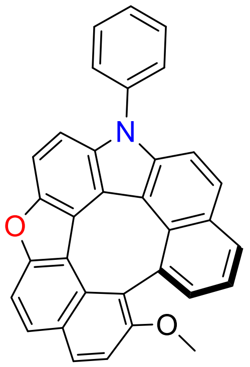

(*M*)-**3ba**

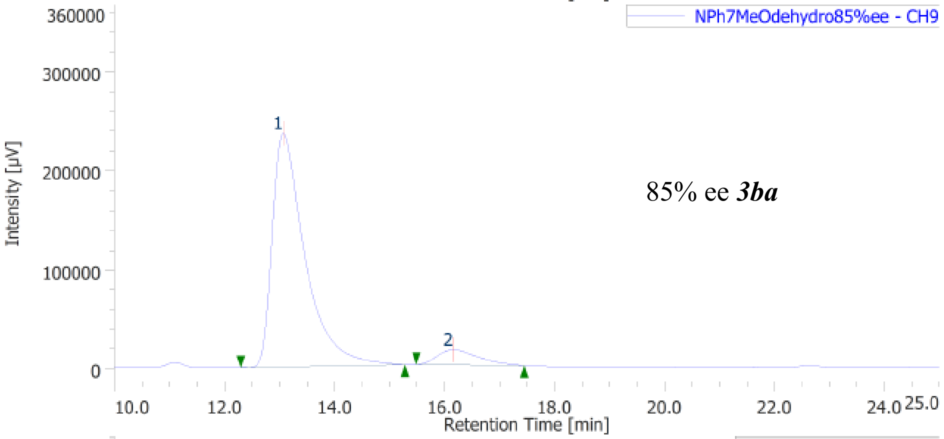

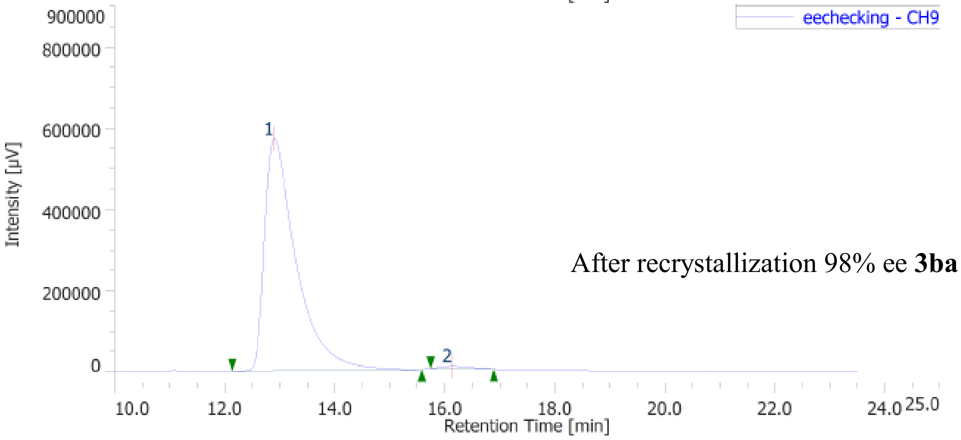

Channel & Peak Information Table

Chromatogram Name N-Ph7MeOdehydrorcIC3HIPA5011mlmin-CH9
Sample Name
Channel Name 230.0nm

#	Peak Name	CH	tR [min]	Area [μV·sec]	Height [μV]	Area%	Height%	Quantity	NTP	Resolution	Symmetry Factor	Warning
1	Unknown	9	13.137	1363113	33117	49.946	57.330	N/A	2714	2.531	1.878	
2	Unknown	9	16.100	1366059	24649	50.054	42.670	N/A	2312	N/A	1.849	

Chromatogram Name NPh7MeOdehydro85%ee-CH9
Sample Name
Channel Name 230.0nm

#	Peak Name	CH	tR [min]	Area [μV·sec]	Height [μV]	Area%	Height%	Quantity	NTP	Resolution	Symmetry Factor	Warning
1	Unknown	9	13.067	8131582	199192	92.619	93.970	N/A	2699	2.640	1.918	
2	Unknown	9	16.167	647976	12783	7.381	6.030	N/A	2300	N/A	1.491	

Chromatogram Name eechecking-CH9
Sample Name
Channel Name 230.0nm

#	Peak Name	CH	tR [min]	Area [μV·sec]	Height [μV]	Area%	Height%	Quantity	NTP	Resolution	Symmetry Factor	Warning
1	Unknown	9	12.903	22846310	571669	98.920	98.847	N/A	2831	3.202	2.183	
2	Unknown	9	16.143	249540	6671	1.080	1.153	N/A	3716	N/A	1.403	

Determined by HPLC (Daicel Chiralpak IC-3, hexane/*i*-PrOH = 50/1, flow rate 1.0 mL/min, T = 25 ºC, 230 nm): t_{maj} = 12.9 min, t_{min} = 16.1 min.

8. Supplementary Note 1: *determination of the absolute configuration of **3ba***

The configuration of **3ba** was determined as (*M*) after comparing the CD spectrum of **3ba** with that of previously assigned (*M*)-**3aa** and (*P*)-**3aa** *via* X-ray crystallographic analysis, and found the pattern of **3ba** is matching with the (*M*) configuration. Hence, **4ba** has an (*R*) configuration (i.e. (*R*)-**4ba** will give exclusively (*M*)-**3ba** and vice versa). These results were also matching with our previous result[1)] in which similar substrates gave (*R*)-diols using this vanadium complex (R_a,*S*)-**9a**

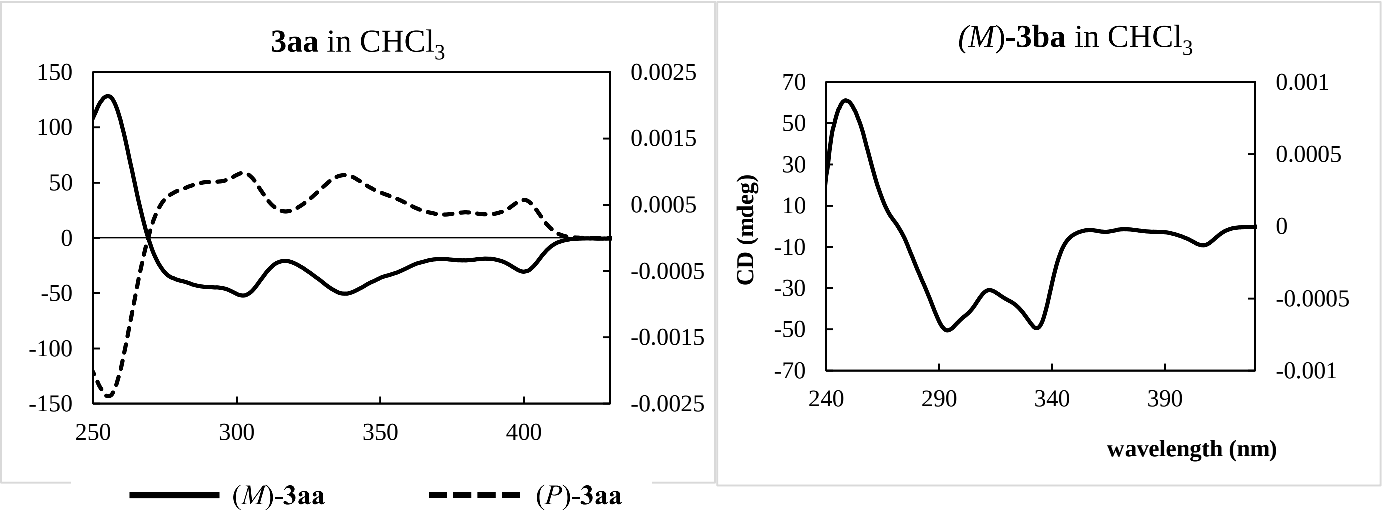

9. Supplementary Note 2: *racemization barrier of dehydro[7]helicenes **3***

Since racemization is a first-order process, we could monitor the change in the enantiomeric excess ratio (ee) with time at different temperatures 100- 150 °C.

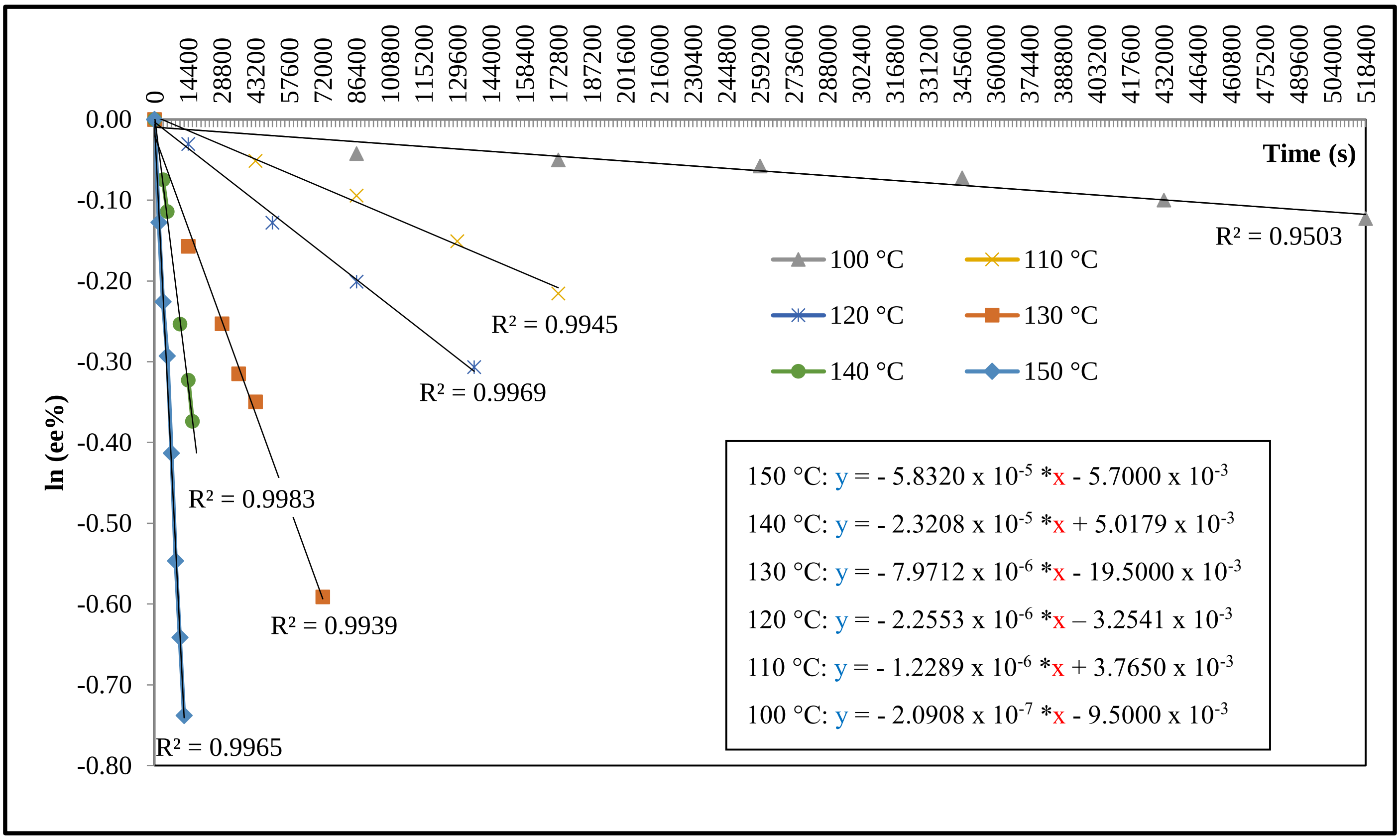

Figure S2: A plot showing ln(ee) versus time in seconds to show racemization rate of (+) **3aa** as it was heated at 100, 110, 120, 130, 140 and 150 °C, at 1 mg/mL concentration in DMF

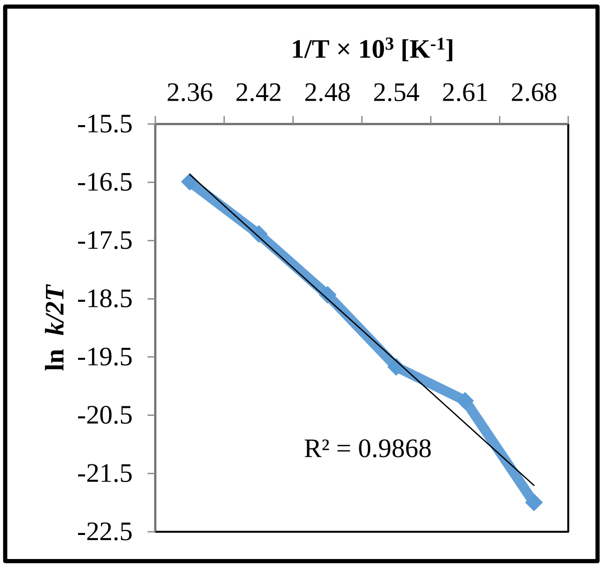

Figure S3: Eyring plot of **3aa** showing the change in ln(*k*/2T) versus 1/T

Eyring plot: $y = -1.6880 \times 10^{4} * x + 23.4356$

$\ln(k/2T) = -1.6880 \times 10^{4} * (1/T) + 24.7568$

$-\Delta H^{\ddagger}/R = -1.6880 \times 10^{4}$ & $\ln(kb/h) + \Delta S^{\ddagger}/R = 24.7568$

$\Delta H^{\ddagger}$ = 140.345 kJ.mol^{-1} as (R = 8.31 J/mol.K)

$\Delta S^{\ddagger}$ = -2.6880 J.mol^{-1} K^{-1} as kB (Boltzmann const. = 1.38×10^{-23} J/K) & h (Planck's const. = 6.63×10^{-34} J.s)

$\Delta G^{\ddagger} = \Delta H^{\ddagger} - T\,\Delta S^{\ddagger}$

$\Delta G^{\ddagger}$ = 141.415 ± 0.05

$\Delta G^{\ddagger}$ at 100 °C = 141.3481 kJ.mol^{-1}

$\Delta G^{\ddagger}$ at 110 °C = 141.3750 kJ.mol^{-1}

$\Delta G^{\ddagger}$ at 120 °C = 141.4022 kJ.mol^{-1}

$\Delta G^{\ddagger}$ at 130 °C = 141.4291 kJ.mol^{-1}

$\Delta G^{\ddagger}$ at 140 °C = 141.4560 kJ.mol^{-1}

$\Delta G^{\ddagger}$ at 150 °C = 141.4819 kJ.mol^{-1}

$t_{1/2}$ at 25 °C = ln 2 /*k*

k at 25 °C = 2.3233×10^{-12}

$t_{1/2}$ at 25 °C = 9.5×10^{3} years

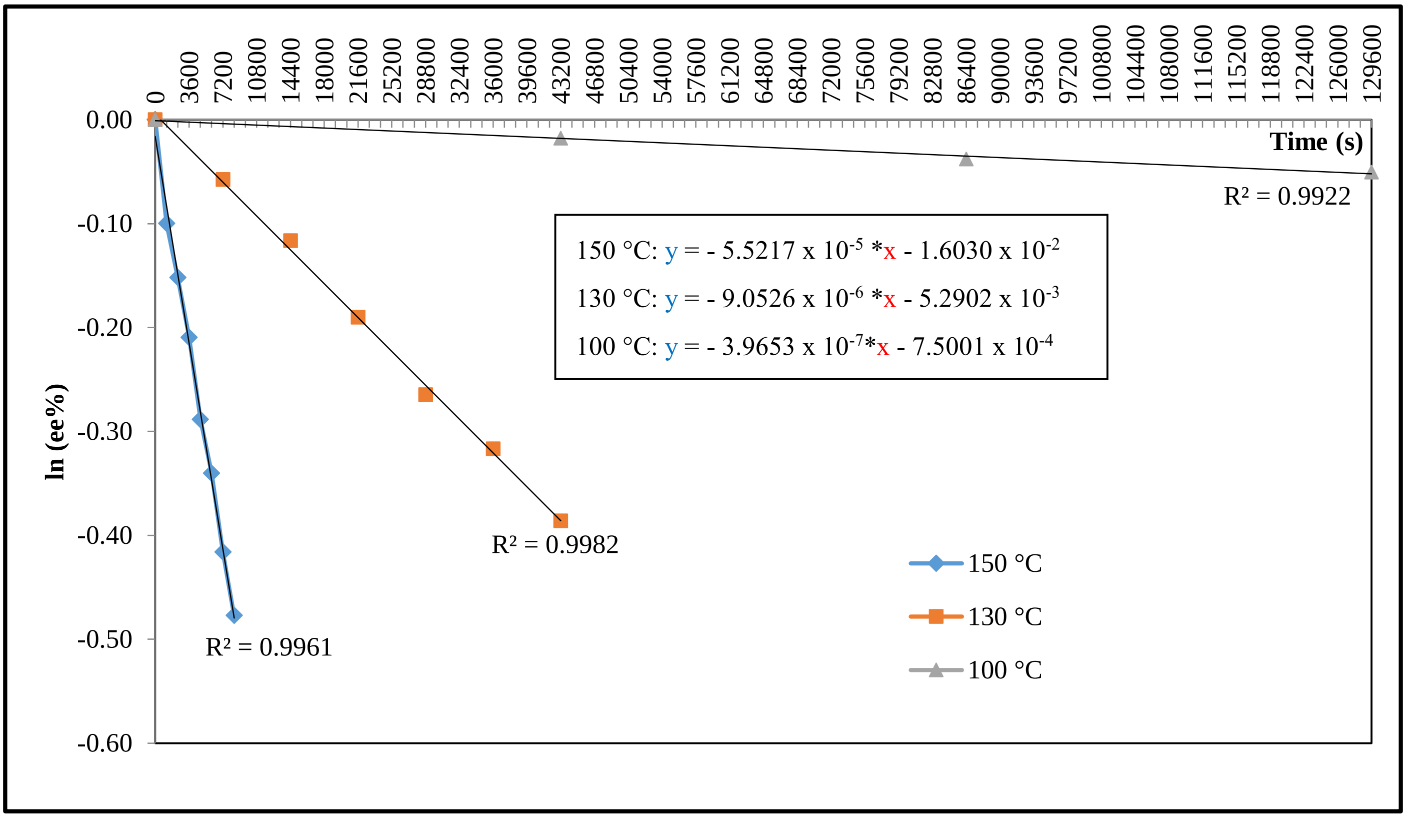

Figure S4: A plot showing ln(ee) versus time in seconds to show racemization rate of (+) **3ca** as it was heated at 100, 130, and 150 °C, at 1 mg/mL concentration in DMF.

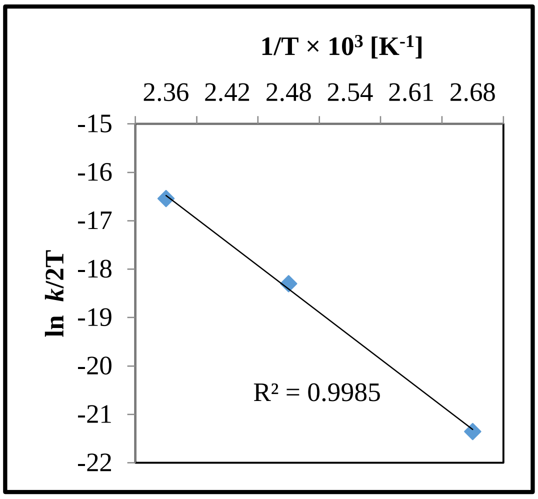

$y = -1.5203 \times 10^4 * x + 19.3914$

$\ln(k/2T) = -1.5203 \times 10^4 * (1/T) + 19.3914$

$-\Delta H^‡/R = -1.5203 \times 10^4$ & $\ln(kb/h) + \Delta S^‡/R = 19.3914$

ΔH‡ = 126.4041 kJ.mol^{-1} as (R = 8.31 J/mol.K)

ΔS‡ = -36.3130 J.mol^{-1} K^{-1} as kB (Boltzmann const. = 1.38×10^{-23} J/K) & h (Planck's const. = 6.63×10^{-34} J.s)

ΔG‡ = ΔH‡ -T ΔS‡

ΔG‡ = 140.9 ± 0.914

ΔG‡ at 100 °C = 140.0 kJ.mol^{-1}

ΔG‡ at 130 °C = 141.0 kJ.mol^{-1}

ΔG‡ at 150 °C = 141.8 kJ.mol^{-1}

Figure S5: Eyring plot of **3ca** showing the change in ln(*k*/2T) versus 1/T.

$t_{1/2}$ at 25 °C = ln 2 /*k*

k at 25 °C = 1.1278×10^{-11}

$t_{1/2}$ at 25 °C = 1.9 x 10^3 years

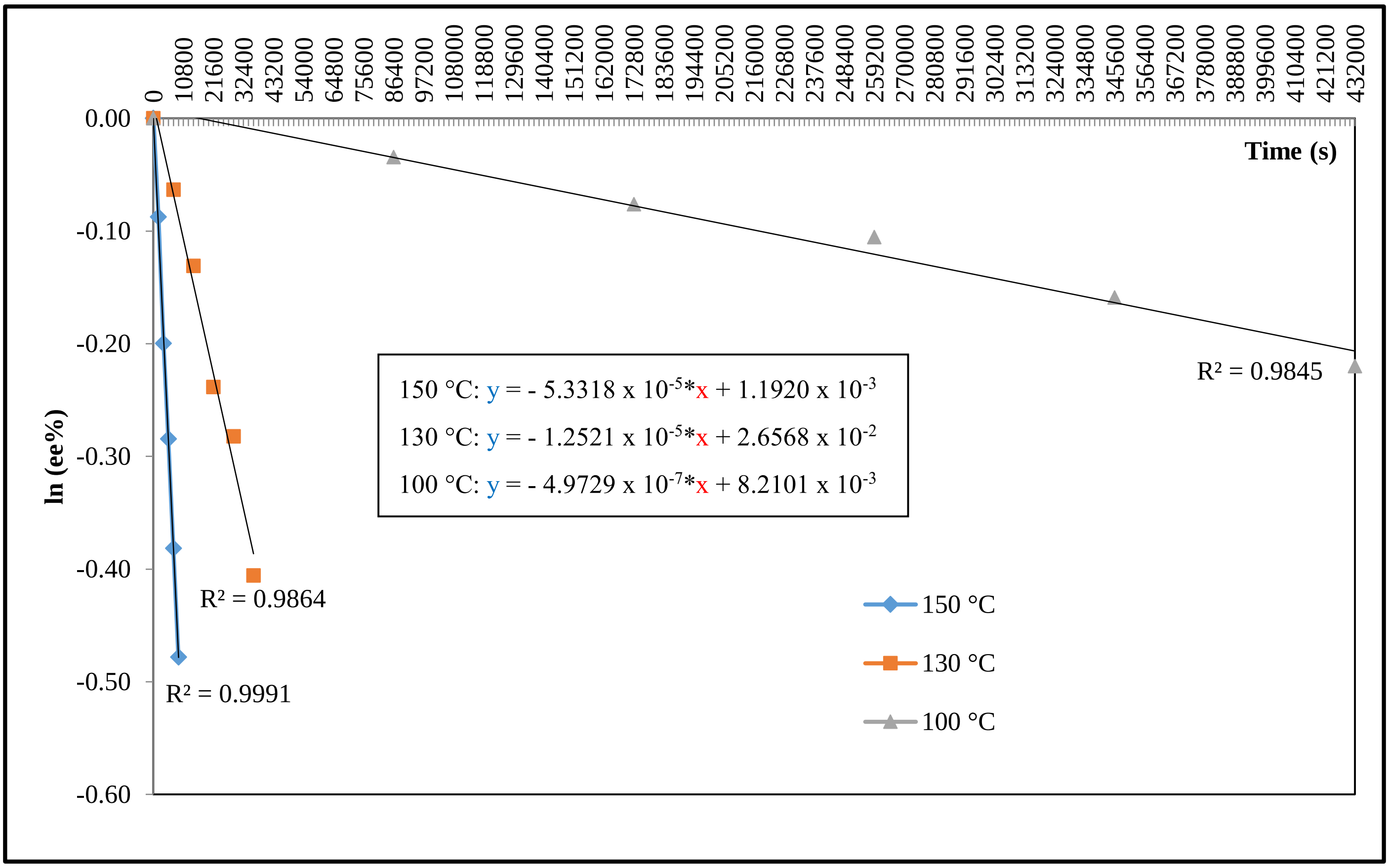

Figure S6: A plot showing ln(ee) versus time in seconds to show racemization rate of (+) **3fa** as it was heated at 100, 130, and 150 °C, at 1 mg/mL concentration in DMF.

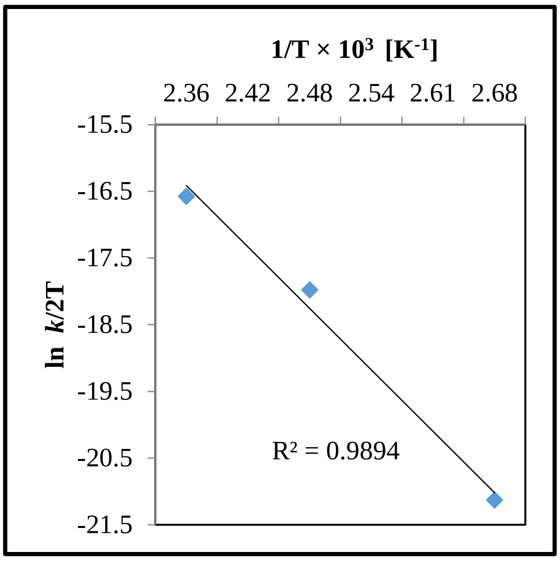

Figure S7: Eyring plot of **3fa** showing the change in ln(k/2T) versus 1/T.

$y = -1.4518 \times 10^4 * x + 17.8447$

$\ln(k/2T) = -1.4518 \times 10^4 * (1/T) + 17.8447$

$-\Delta H^‡/R = -1.4518 \times 10^4$ & $\ln(k_B/h) + \Delta S^‡/R = 17.8447$

$\Delta H^‡$ = 120.7060 kJ.mol^{-1} as (R = 8.31 J/mol.K)

$\Delta S^‡$ = -49.1741 J.mol^{-1} K^{-1} as kB (Boltzmann const. = 1.38×10^{-23} J/K) & h (Planck's const. = 6.63×10^{-34} J.s)

$$\Delta G^‡ = \Delta H^‡ - T\,\Delta S^‡$$

$$\Delta G^‡ = 140.4 \pm 1.24$$

$\Delta G^‡$ at 100 °C = 139.1 kJ.mol^{-1}

$\Delta G^‡$ at 130 °C = 140.5 kJ.mol^{-1}

$\Delta G^‡$ at 150 °C = 141.5 kJ.mol^{-1}

$t_{1/2}$ at 25 °C = ln 2 /k

k at 25 °C = 2.3918×10^{-11}

$t_{1/2}$ at 25 °C = 9.2×10^2 years

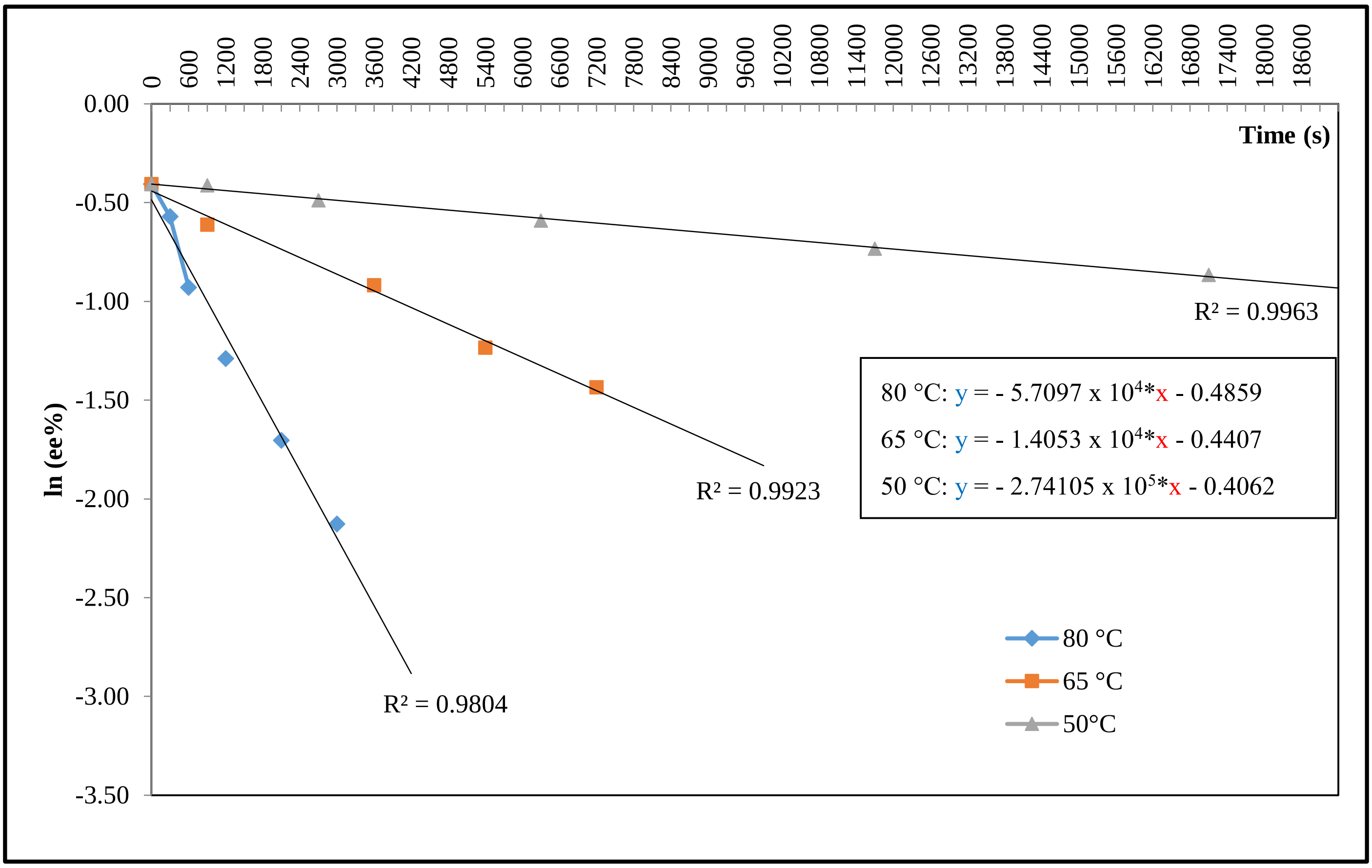

Figure S8: A plot showing ln(ee) versus time in seconds to show racemization rate of (+) **5aa** as it was heated at 50, 65, and 80 °C, at 1 mg/mL concentration in toluene.

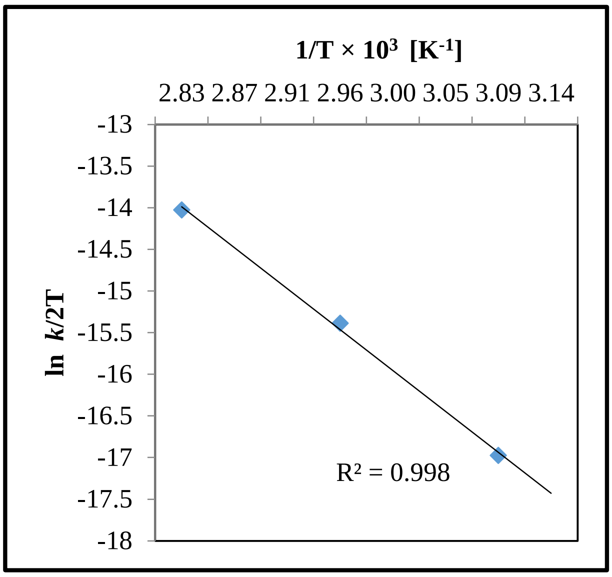

Figure S9: Eyring plot of **5aa** showing the change in ln(k/2T) versus 1/T.

$y = -1.1222 \times 10^4 * x + 17.7663$

$\ln(k/2T) = -1.1222 \times 10^4 * (1/T) + 17.7663$

$-\Delta H^{\ddagger}/R = -1.1222 \times 10^4$ & $\ln(kb/h) + \Delta S^{\ddagger}/R = 17.7663$

$\Delta H^{\ddagger}$ = 93.3043 kJ.mol^{-1} as (R = 8.31 J/mol.K)

$\Delta S^{\ddagger}$ = -49.8260 J.mol^{-1} K^{-1} as kB (Boltzmann const. = 1.38×10^{-23} J/K) & h (Planck's const. = 6.63×10^{-34} J.s)

$\Delta G^{\ddagger} = \Delta H^{\ddagger} - T\,\Delta S^{\ddagger}$

$\Delta G^{\ddagger}$ = 110.2 ± 0.74

$\Delta G^{\ddagger}$ at 50 °C = 109.4 kJ.mol^{-1}

$\Delta G^{\ddagger}$ at 65 °C = 110.3 kJ.mol^{-1}

$\Delta G^{\ddagger}$ at 80 °C = 110.9 kJ.mol^{-1}

$t_{1/2}$ at 25 °C = ln 2 /*k*

(Using **Eq. 17**) k at 25 °C = 1.3970×10^{-6}

$t_{1/2}$ at 25 °C = 5.7 days

After comparing the thermodynamic parameters of dehydro[7]helicenes **3aa**, **3ca**, and **3fa**, we realized that our scaffold can exhibit a high racemization barrier > 140 kJ mol^{-1} (~ 33.5 kcal mol^{-1}) opening the gate for wide applications that require significantly high chiral stability and sustained racemization half-life time. The $t_{1/2}$ of compound **3aa** at 25 °C was more than 9.5 x 10^3 years which reflects the high chiral stability and low entropy ($\Delta S^{\ddagger}$). Although changing the *N*-substituents of dehydrohelicene **3**, has no great effect on $\Delta G^{\ddagger}$ values, other thermodynamic parameters showed considerable variations which revealed on the lower racemization half-life times (**Figure S10**). To show the superior chiral stability of our dehydrohelicene **3aa**, we wanted to compare with the corresponding helicene **5aa**; hence we isolated that compound and separated its two enantiomers by HPLC. After checking the racemization rate at different temperatures, and drawing the Eyring plot, we observed the lower racemization barrier of **5aa** ~110 kJ mol^{-1} (~ 26.3 kcal mol^{-1}) and the dramatically shorter $t_{1/2}$ at 25 °C (only 5.74 days). In other words, compound **5aa** racemizes more than million times faster compared to the corresponding dehydrohelicene **3aa**.

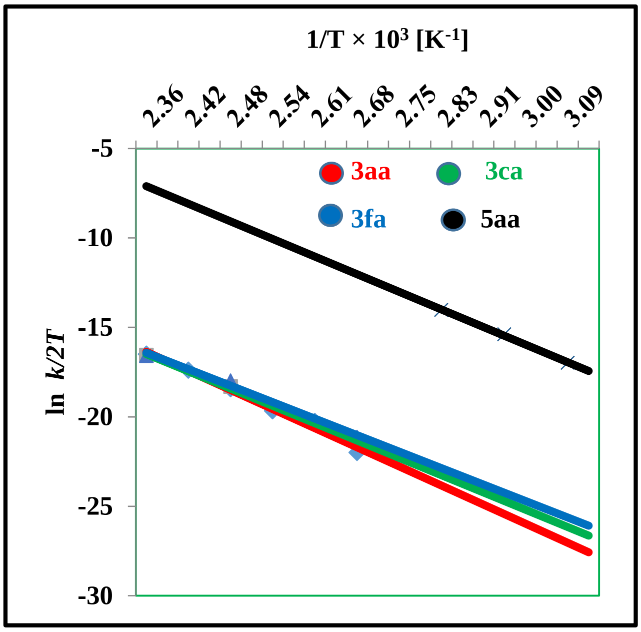

	3aa	5aa
R^2	0.999	0.998
Slope	-1.6880×10^4	-1.1222×10^4
intercept	23.4356	17.7663
k/s^{-1} at 25°C	2.32×10^{-12}	1.40×10^{-6}

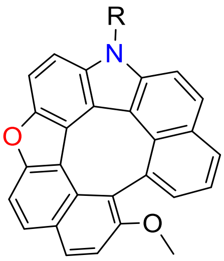

3

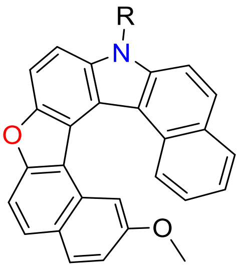

5aa

	R	ΔH≠	ΔS≠	ΔG≠	$t_{1/2}$ at 25 °C
**3aa**	*p*-tolyl	**140.345 kJ.mol^{-1}**	**-2.688 J.mol^{-1}K^{-1}**	**141.4 ± 0.05 kJ.mol^{-1}**	**9.5 x 10^3 years**
**3ca**	*p*-ClC_6H_4	**126.404 kJ.mol^{-1}**	**-36.313 J.mol^{-1}K^{-1}**	**140.9 ± 0.91 kJ.mol^{-1}**	**1.9 x 10^3 years**
**3fa**	Bn	**120.706 kJ.mol^{-1}**	**-49.174 J.mol^{-1}K^{-1}**	**140.4 ± 1.24 kJ.mol^{-1}**	**9.2 x 10^2 years**
**5aa**	*p*-tolyl	**93.304 kJ.mol^{-1}**	**-49.826 J.mol^{-1}K^{-1}**	**110.2 ± 0.75 kJ.mol^{-1}**	**5.7 days**

Figure S10. Eyring plot for the racemization of dehydro[7]helicenes **3aa, 3ca, 3fa,** and helicene **5aa.**

10. Supplementary Note 3: *plausible reaction mechanism for the electrochemical sequential reaction*

The anodic oxidation of **1a** would occur first to generate the electrophilic radical species (intermediate **I**), because **1a** is more easily oxidized than **2a** (from CV studies; Figure S18). After that, single electron transfer (SET) would occur to generate the electrophilic radical species **II** at the anode that undergo radical-anion coupling with the more nucleophilic **2a**. Further anodic oxidations can afford intermediate **IV** that can either tautomerize to generate diol **4aa** or readily undergoes dehydrative cyclization to afford [7]helicene **5aa**. In the presence of acid additives like ($BF_3.OEt_2$) as demonstrated by some previous studies,[10,11)] diol **4aa** would undergo dehydrative cyclization to afford [7]helicene **5aa** as well. Finally, this [7]helicene **5aa** will undergo two successive anodic oxidation to afford the corresponding dehydro[7]helicene *via* intramolecular C-C bond formation (**Scheme S1**).

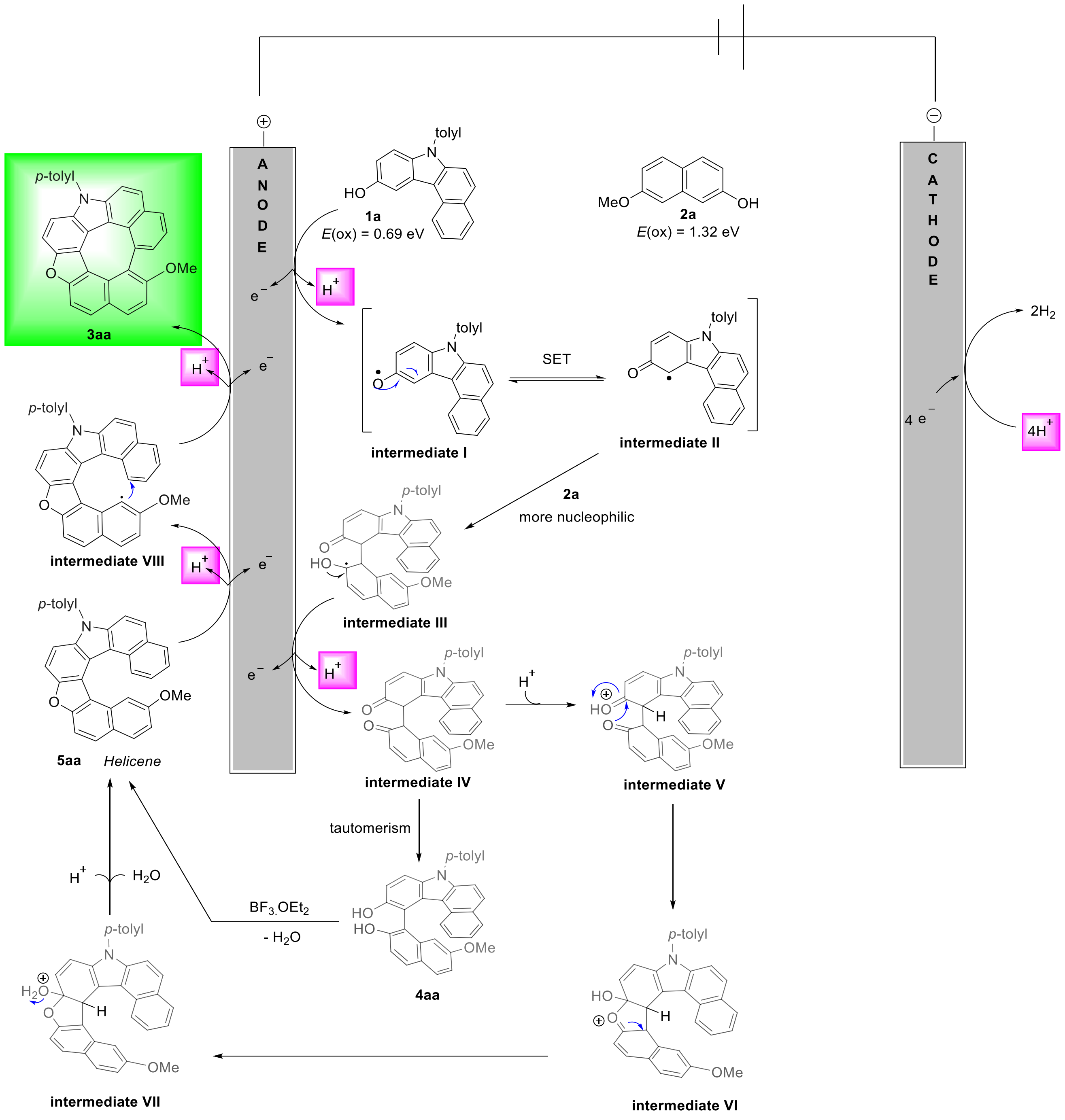

Scheme S1. Plausible reaction mechanism

11. Supplementary Note 4: *photophysical properties (UV and PL)*

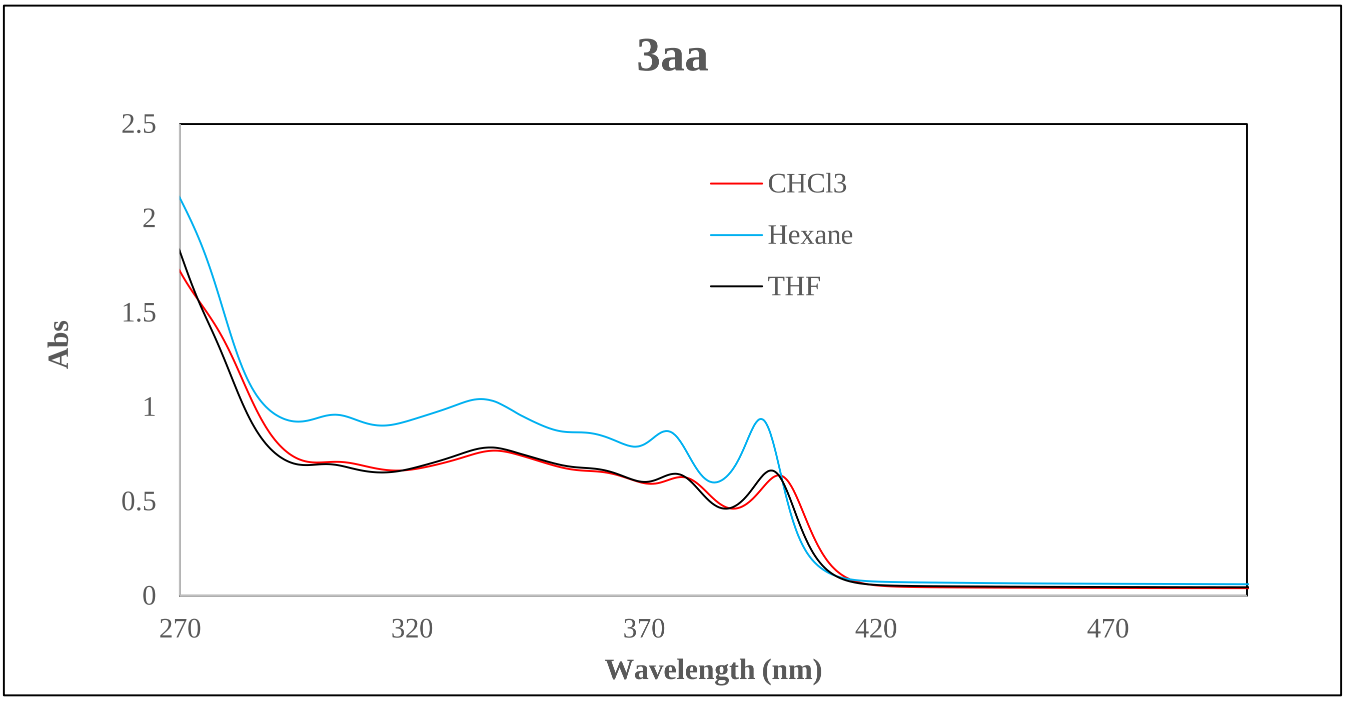

Figure S11. UV spectra of compound **3aa** in ($CHCl_3$, hexane, and THF) 20 µM solutions.

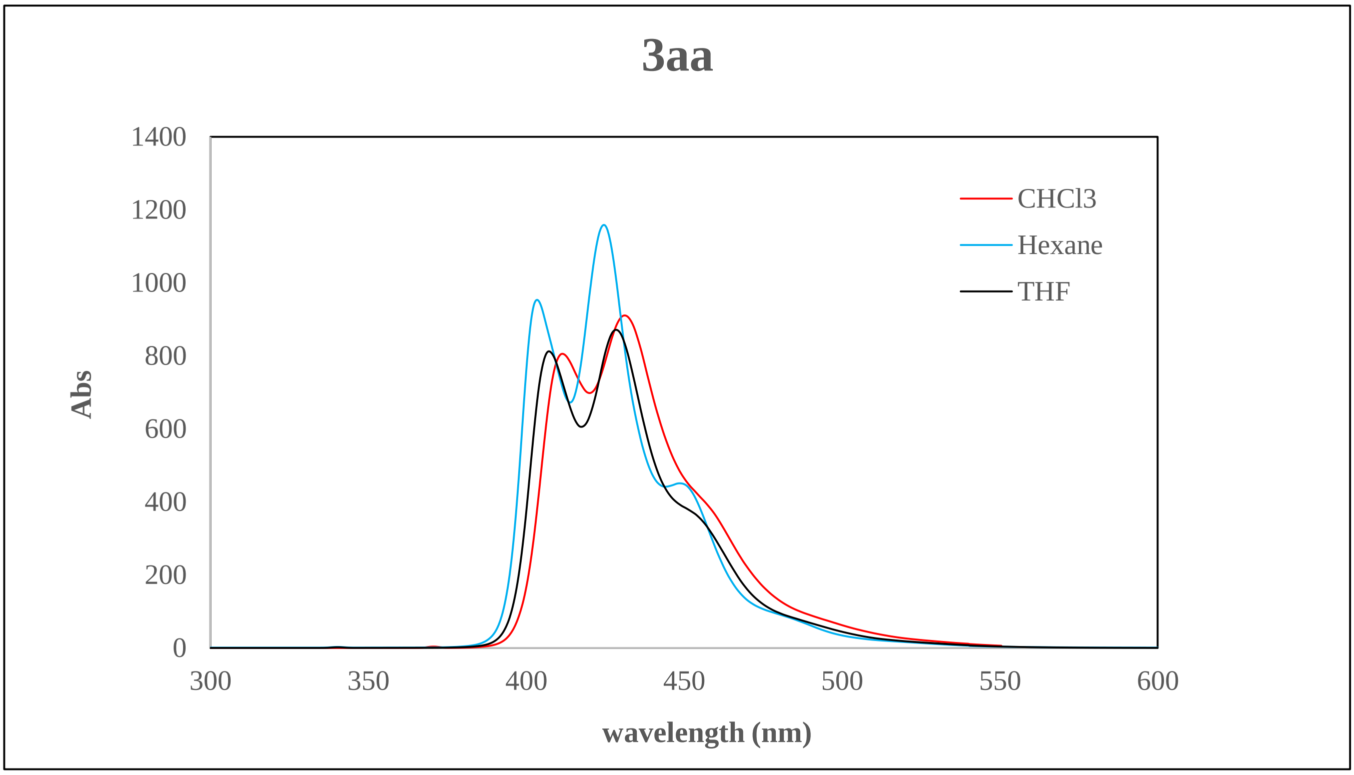

Figure S12. PL spectra of compound **3aa** measured at excitation wavelength of 340 nm, in ($CHCl_3$, hexane, and THF) 20 µM solutions.

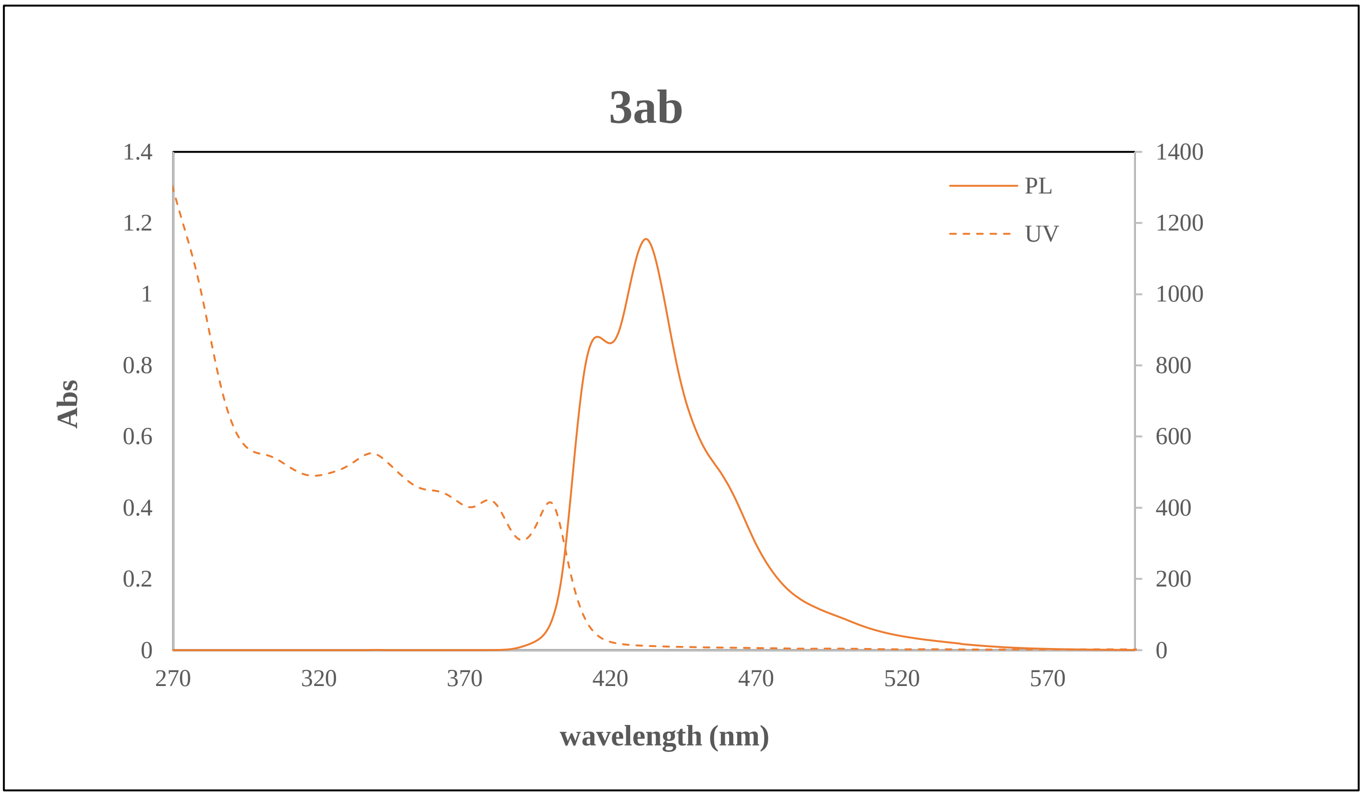

Figure S13. UV and PL spectra (measured at excitation wavelength of 339 nm) of compound **3ab** in $CHCl_3$ (20 µM) solutions.

These good photophysical properties can open the gate for various optical applications that depends on the absorption and emission characteristics of different materials. For example, the emissive layers in OLEDs, optical fibers that can be used in transfer of information. Other material-based applications like fluorescence sensors, and OFETs.

12. Supplementary Note 5: *chiroptical properties (CD and CPL)*

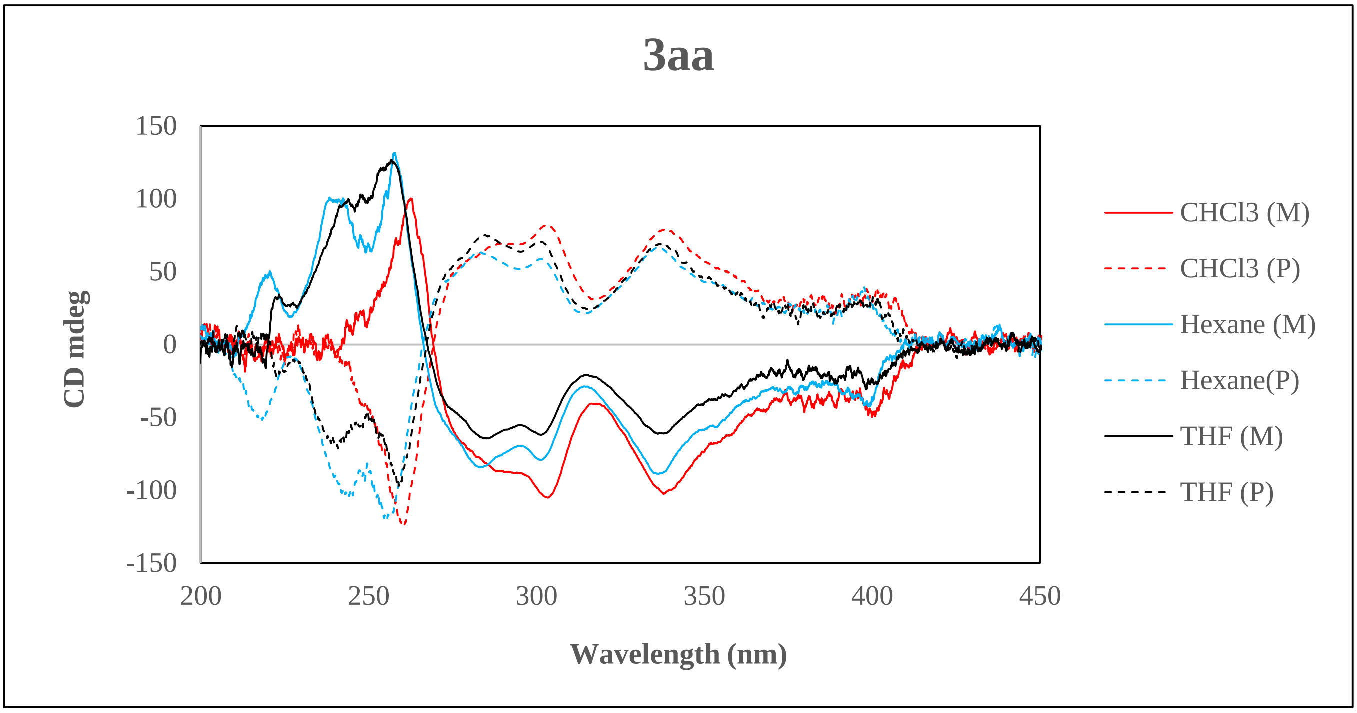

Figure S14. CD spectra of compound **3aa** in ($CHCl_3$, hexane, and THF) 20 µM solutions.

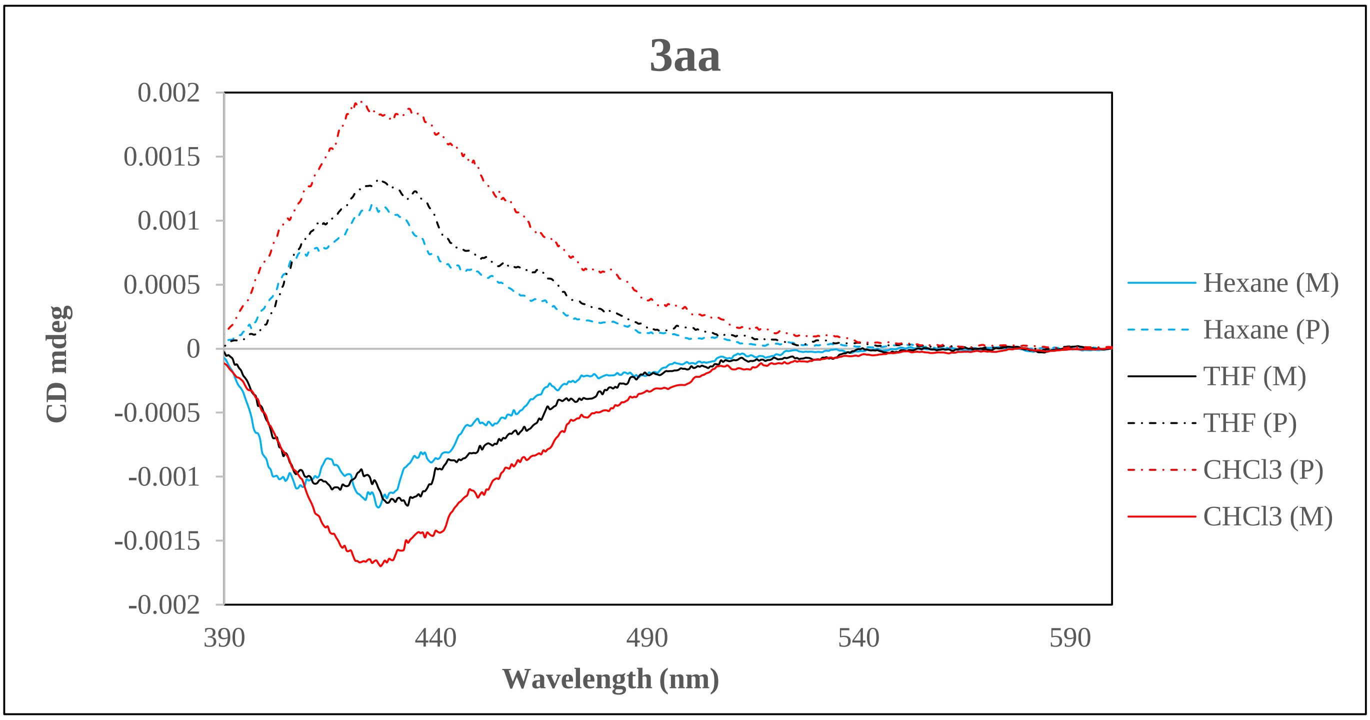

Figure S15. CPL spectra of compound **3aa** in ($CHCl_3$, hexane, and THF) 20 µM solutions. g_{lum} = (2.5×10^{-3}, 2.25×10^{-3}, and 2.2×10^{-3} in $CHCl_3$, hexane, and THF respectively).

PLQY of **3aa** = 0.25, measured with integrated sphere in $CHCl_3$ at room temperature.

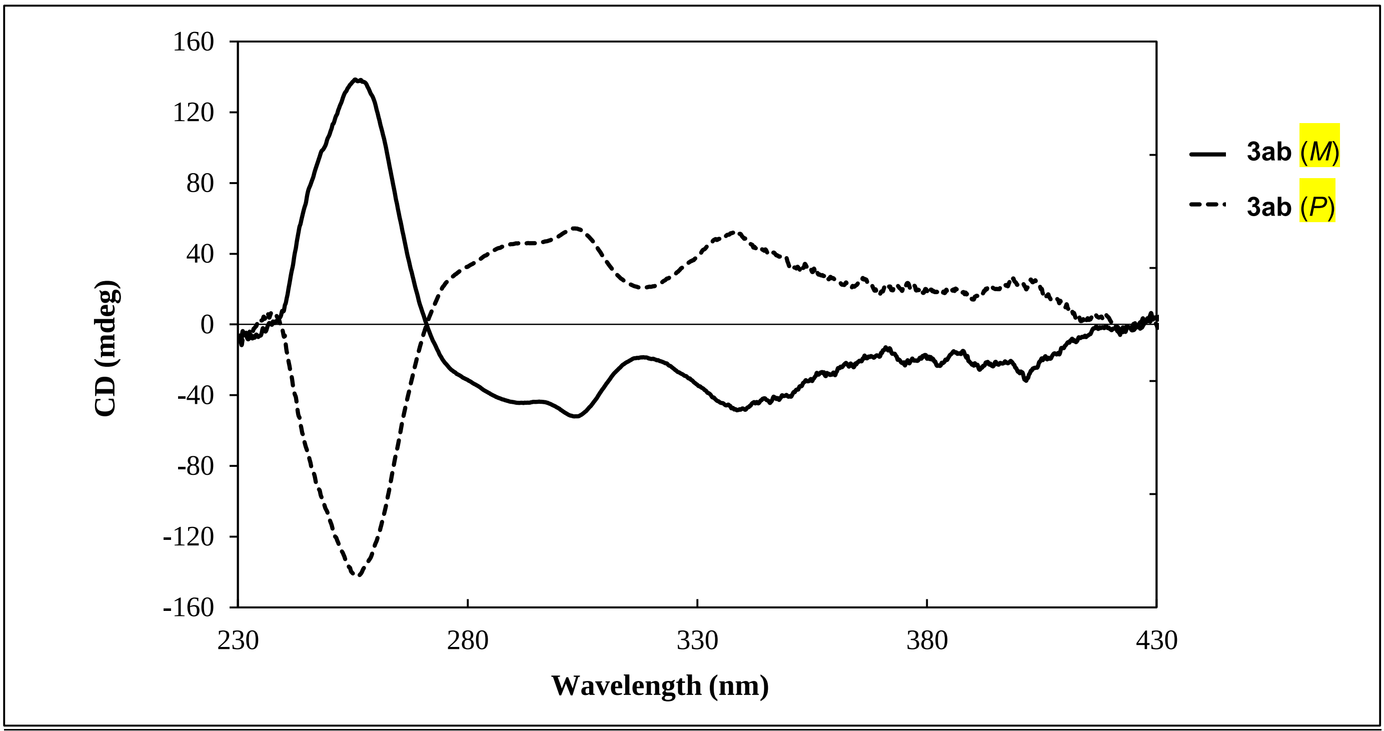

Figure S16. CD spectra of compound (*P* or *M*) **3ab** in $CHCl_3$ (20 μM solutions).

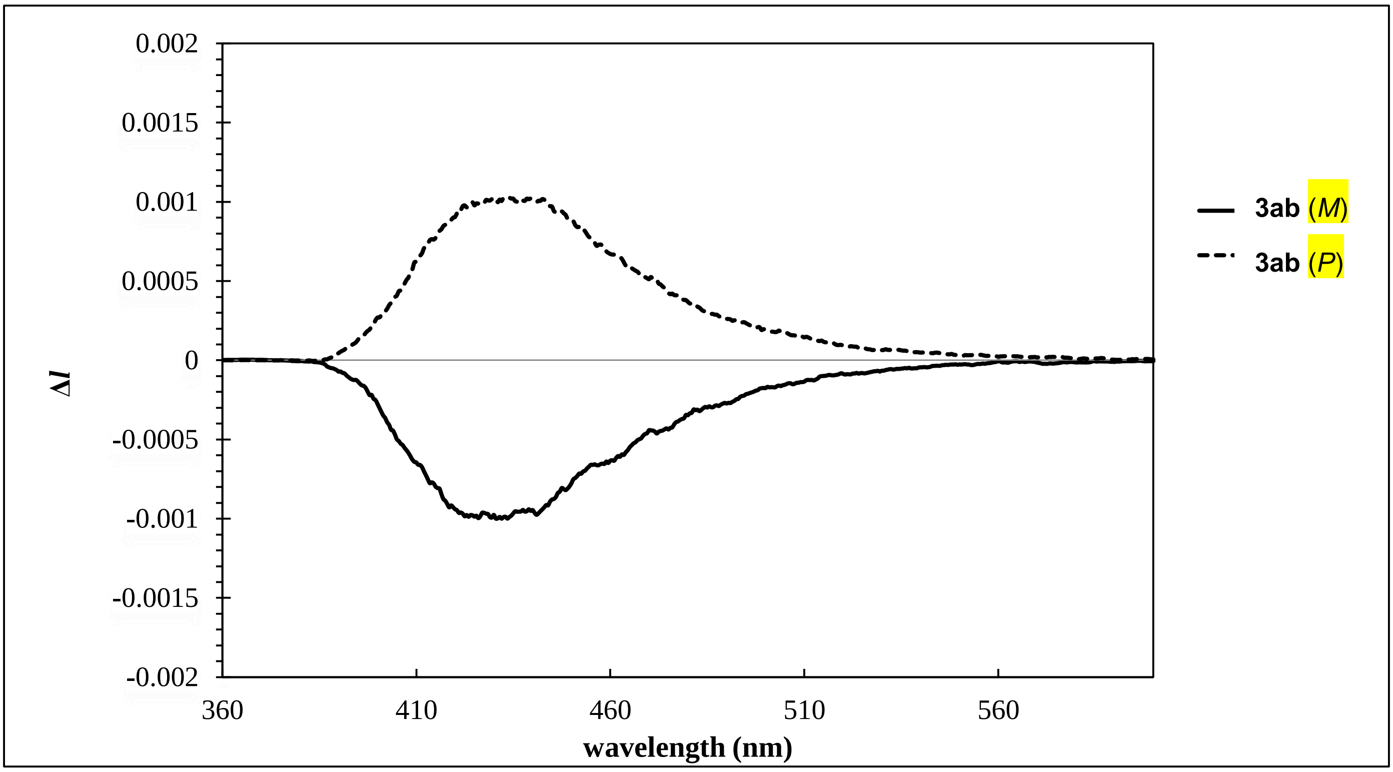

Figure S17. CPL spectra of compound (*P* or *M*) **3ab** in $CHCl_3$ (20 μM solutions). g_{lum} = (2.4×10^{-3})

PLQY of **3ab** = 0.16, measured with integrated sphere in $CHCl_3$ at room temperature.

13. Supplementary Note 6: *CV charts*

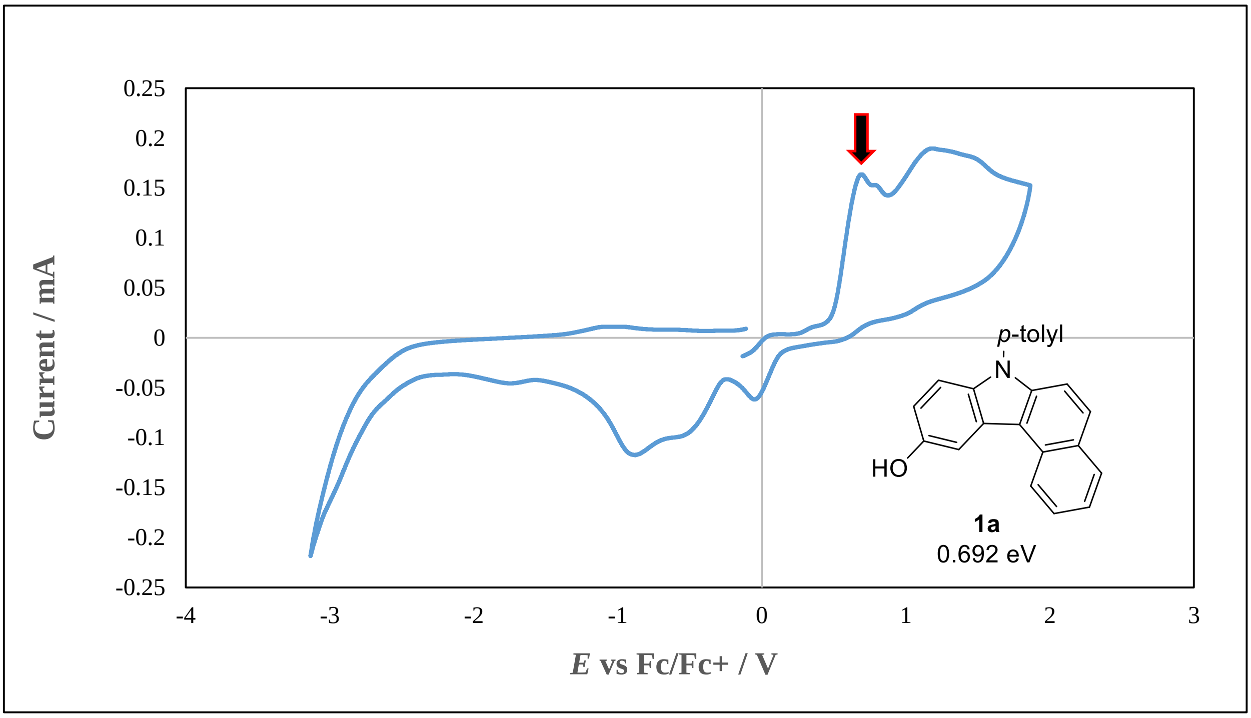

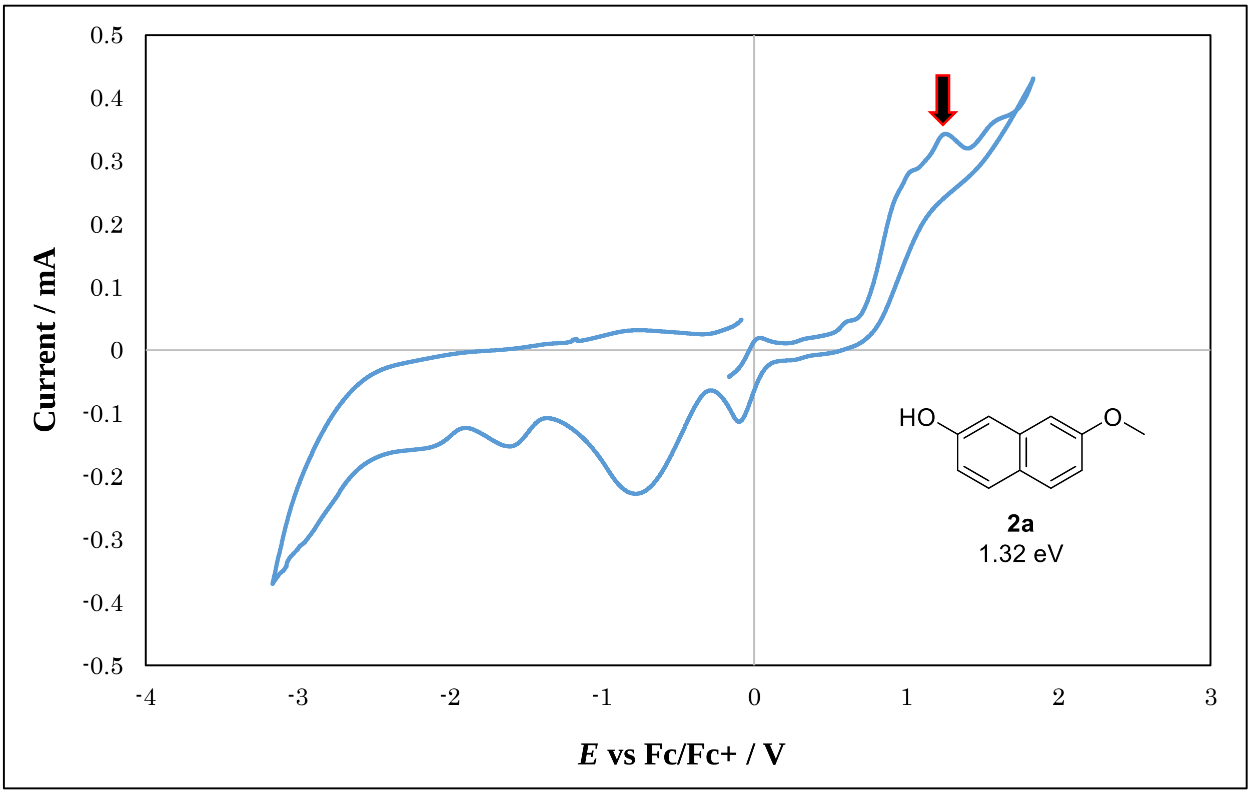

Figure S18. CV experiments (MeCN) as a solvent with Bu4NPF6 (0.1 M) as electrolyte. Compound **1a** decomposes upon oxidation.

14. Supplementary Note 7: *X-ray crystallographic analysis*

3aa (CCDC 2091483) with ellipsoids at 30% probability. (H atoms were omitted for clarity).

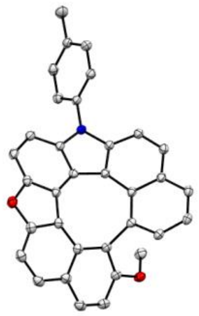

Empirical formula	$C_{34}H_{21}NO_2$
Formula weight	475.551
Temperature/K	173
Crystal system	monoclinic
Space group	$P2_1$
a/Å	9.1897(1)
b/Å	23.1326(3)
c/Å	10.5732(2)
α/°	90
β/°	91.282(1)
γ/°	90
Volume/Å^3	2247.11(6)
Z	4
ρ_{calc}g/cm^3	1.406
μ/mm^{-1}	0.686
F(000)	995.2
Crystal size/mm^3	0.082 × 0.042 × 0.022
Radiation	CuKα (λ = 1.54184)
2Θ range for data collection/°	7.64 to 161.2
Index ranges	$-11 \leq h \leq 11$, $-29 \leq k \leq 28$, $-13 \leq l \leq 13$
Reflections collected	59505
Independent reflections	9585 [R_{int} = 0.0383, R_{sigma} = 0.0254]
Data/restraints/parameters	9585/1/671
Goodness-of-fit on F^2	1.051
Final R indexes [I>=2σ (I)]	R_1 = 0.0358, wR_2 = 0.0866
Final R indexes [all data]	R_1 = 0.0406, wR_2 = 0.0900
Largest diff. peak/hole / e Å^{-3}	0.16/-0.18
Flack parameter	-0.02(6)

Datablock: 200514lm-6

Bond precision: C-C = 0.0027 A Wavelength=1.54184

Cell:	a=9.1897(1)	b=23.1326(3)	c=10.5732(2)
	alpha=90	beta=91.282(1)	gamma=90
Temperature:	173 K		

	Calculated	Reported
Volume	2247.11(6)	2247.11(6)
Space group	P 21	P 1 21 1
Hall group	P 2yb	P 2yb
Moiety formula	C34 H21 N O2	C34 H21 N O2
Sum formula	C34 H21 N O2	C34 H21 N O2
Mr	475.52	475.55
Dx,g cm-3	1.406	1.406
Z	4	4
Mu (mm-1)	0.686	0.686
F000	992.0	995.2
F000'	994.84	
h,k,lmax	11,29,13	11,29,13
Nref	9866[5058]	9585
Tmin,Tmax	0.966,0.985	0.862,1.000
Tmin'	0.945	

Correction method= # Reported T Limits: Tmin=0.862 Tmax=1.000
AbsCorr = MULTI-SCAN

Data completeness= 1.90/0.97 Theta(max)= 80.600

R(reflections)= 0.0358(8733) wR2(reflections)= 0.0900(9585)

S = 1.051 Npar= 671

The following ALERTS were generated. Each ALERT has the format
test-name_ALERT_alert-type_alert-level.
Click on the hyperlinks for more details of the test.

Alert level G

```
PLAT005_ALERT_5_G No Embedded Refinement Details Found  in the CIF     Please Do !
PLAT068_ALERT_1_G Reported F000 Differs from Calcd (or Missing)...     Please Check
PLAT073_ALERT_1_G H-atoms ref, but _hydrogen_treatment Reported as     constr Check
PLAT398_ALERT_2_G Deviating  C-O-C   Angle From 120 for O13       .     105.6 Degree
PLAT398_ALERT_2_G Deviating  C-O-C   Angle From 120 for O50       .     105.5 Degree
PLAT769_ALERT_4_G CIF Embedded explicitly supplied scattering data     Please Note
PLAT912_ALERT_4_G Missing # of FCF Reflections Above STh/L=  0.600         68 Note
PLAT960_ALERT_3_G Number of Intensities with I  <   - 2*sig(I) ...          1 Check
PLAT978_ALERT_2_G Number C-C Bonds with Positive Residual Density.          2 Info
PLAT982_ALERT_1_G The  C-f'=    0.0192 Deviates from  IT-value =       0.0181 Check
PLAT982_ALERT_1_G The  N-f'=    0.0326 Deviates from  IT-value =       0.0311 Check
PLAT982_ALERT_1_G The  O-f'=    0.0524 Deviates from  IT-value =       0.0492 Check
PLAT983_ALERT_1_G The  O-f"=    0.0338 Deviates from  IT-Value =       0.0322 Check
```

---

0 **ALERT level A** = Most likely a serious problem - resolve or explain
0 **ALERT level B** = A potentially serious problem, consider carefully
0 **ALERT level C** = Check. Ensure it is not caused by an omission or oversight
13 **ALERT level G** = General information/check it is not something unexpected

6 ALERT type 1 CIF construction/syntax error, inconsistent or missing data
3 ALERT type 2 Indicator that the structure model may be wrong or deficient
1 ALERT type 3 Indicator that the structure quality may be low
2 ALERT type 4 Improvement, methodology, query or suggestion
1 ALERT type 5 Informative message, check

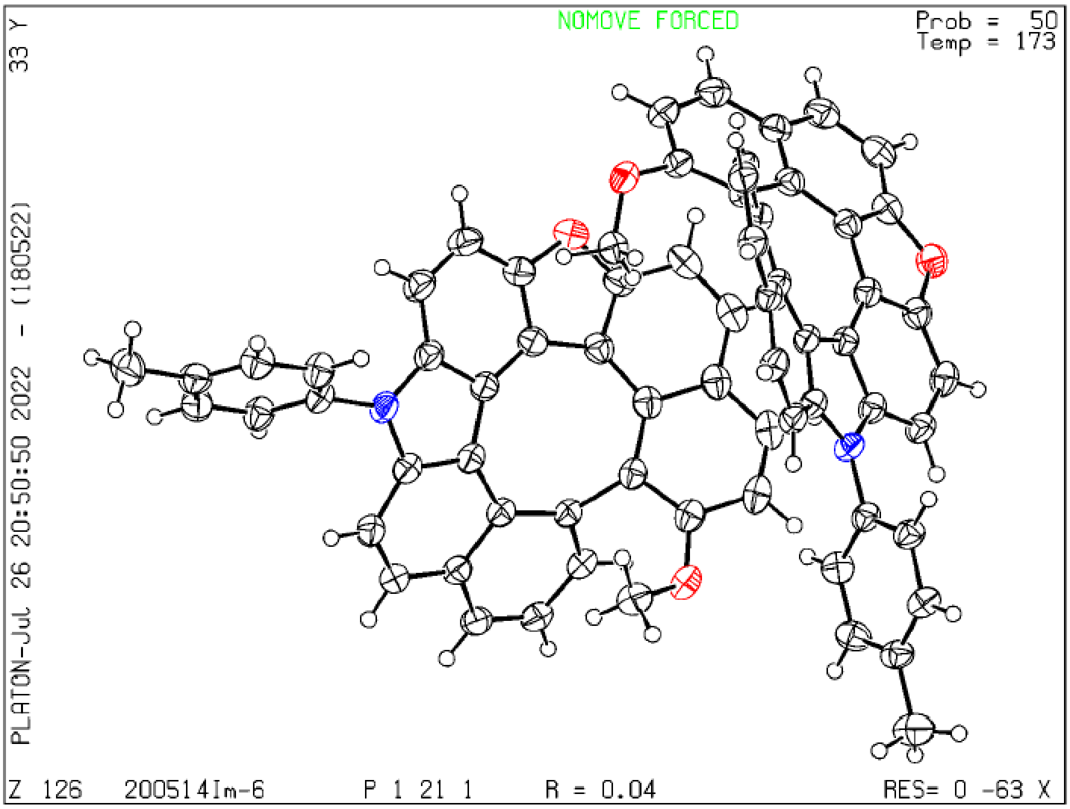

5aa (CCDC 2128351) with ellipsoids at 30% probability. (H atoms were omitted for clarity).

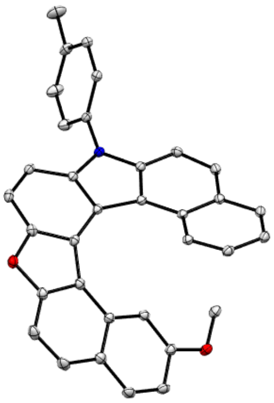

Empirical formula	$C_{34}H_{23}NO_2$
Formula weight	477.567
Temperature/K	100
Crystal system	triclinic
Space group	P-1
a/Å	10.2477(5)
b/Å	11.5049(6)
c/Å	11.5650(5)
α/°	66.563(5)
β/°	76.852(4)
γ/°	68.746(4)
Volume/Å^3	1160.37(11)
Z	2
ρ_{calc}g/cm^3	1.367
μ/mm^{-1}	0.664
F(000)	501.6
Crystal size/mm^3	0.12 × 0.04 × 0.03
Radiation	CuKα (λ = 1.54184)
2Θ range for data collection/°	8.38 to 151.94
Index ranges	-12 ≤ h ≤ 12, -12 ≤ k ≤ 14, -14 ≤ l ≤ 14
Reflections collected	11350
Independent reflections	4657 [R_{int} = 0.0313, R_{sigma} = 0.0423]
Data/restraints/parameters	4657/0/337
Goodness-of-fit on F^2	1.023
Final R indexes [I>=2σ (I)]	R_1 = 0.0419, wR_2 = 0.0977
Final R indexes [all data]	R_1 = 0.0532, wR_2 = 0.1027
Largest diff. peak/hole / e Å^{-3}	0.27/-0.23

Datablock: Req143-1

Bond precision: C-C = 0.0023 A Wavelength=1.54184

Cell:	a=10.2477(5)	b=11.5049(6)	c=11.5650(5)
	alpha=66.563(5)	beta=76.852(4)	gamma=68.746(4)
Temperature:	100 K		

	Calculated	Reported
Volume	1160.37(11)	1160.37(11)
Space group	P -1	P -1
Hall group	-P 1	-P 1
Moiety formula	C34 H23 N O2	C34 H23 N O2
Sum formula	C34 H23 N O2	C34 H23 N O2
Mr	477.53	477.57
Dx,g cm-3	1.367	1.367
Z	2	2
Mu (mm-1)	0.664	0.664
F000	500.0	501.6
F000'	501.42	
h,k,lmax	12,14,14	12,14,14
Nref	4846	4657
Tmin,Tmax	0.969,0.980	0.925,1.000
Tmin'	0.923	

Correction method= # Reported T Limits: Tmin=0.925 Tmax=1.000
AbsCorr = MULTI-SCAN

Data completeness= 0.961 Theta(max)= 75.970

R(reflections)= 0.0419(3861) wR2(reflections)= 0.1027(4657)

S = 1.023 Npar= 337

The following ALERTS were generated. Each ALERT has the format
test-name_ALERT_alert-type_alert-level.
Click on the hyperlinks for more details of the test.

Alert level C

PLAT906_ALERT_3_C Large K Value in the Analysis of Variance ...... 4.801 Check
PLAT911_ALERT_3_C Missing FCF Refl Between Thmin & STh/L= 0.600 21 Report

---

Alert level G

PLAT005_ALERT_5_G No Embedded Refinement Details Found in the CIF Please Do !
PLAT068_ALERT_1_G Reported F000 Differs from Calcd (or Missing)... Please Check
PLAT073_ALERT_1_G H-atoms ref, but _hydrogen_treatment Reported as constr Check
PLAT398_ALERT_2_G Deviating C-O-C Angle From 120 for O002 . 105.4 Degree
PLAT720_ALERT_4_G Number of Unusual/Non-Standard Labels .... 60 Note
PLAT769_ALERT_4_G CIF Embedded explicitly supplied scattering data Please Note
PLAT912_ALERT_4_G Missing # of FCF Reflections Above STh/L= 0.600 169 Note
PLAT978_ALERT_2_G Number C-C Bonds with Positive Residual Density. 11 Info
PLAT982_ALERT_1_G The C-f'= 0.0192 Deviates from IT-value = 0.0181 Check
PLAT982_ALERT_1_G The N-f'= 0.0326 Deviates from IT-value = 0.0311 Check
PLAT982_ALERT_1_G The O-f'= 0.0524 Deviates from IT-value = 0.0492 Check
PLAT983_ALERT_1_G The O-f"= 0.0338 Deviates from IT-Value = 0.0322 Check

---

0 **ALERT level A** = Most likely a serious problem - resolve or explain
0 **ALERT level B** = A potentially serious problem, consider carefully
2 **ALERT level C** = Check. Ensure it is not caused by an omission or oversight
12 **ALERT level G** = General information/check it is not something unexpected

6 ALERT type 1 CIF construction/syntax error, inconsistent or missing data
2 ALERT type 2 Indicator that the structure model may be wrong or deficient
2 ALERT type 3 Indicator that the structure quality may be low
3 ALERT type 4 Improvement, methodology, query or suggestion
1 ALERT type 5 Informative message, check

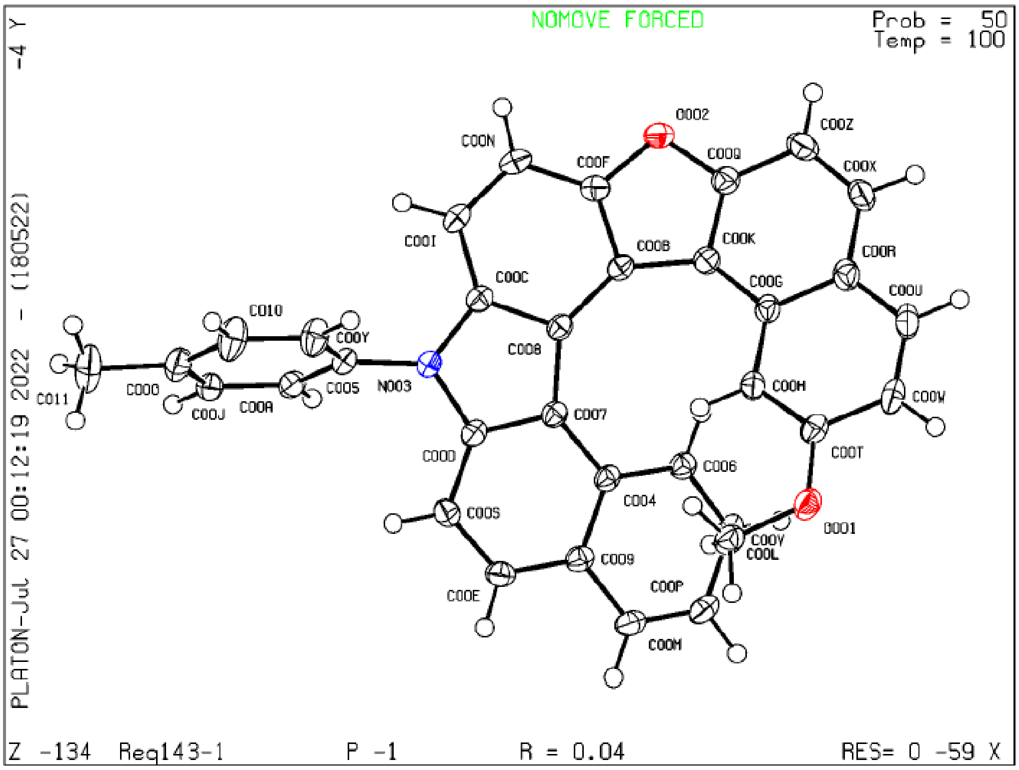

3ma (CCDC 2158892) with ellipsoids at 30% probability. (H atoms were omitted for clarity).

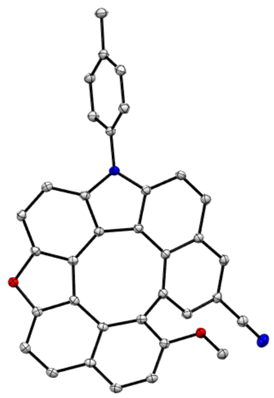

Empirical formula	$C_{46.67}H_{26.67}N_{2.67}O_{2.67}$
Formula weight	667.415
Temperature/K	100
Crystal system	monoclinic
Space group	$P2_1/c$
a/Å	12.7952(2)
b/Å	13.3698(2)
c/Å	14.2802(2)
α/°	90
β/°	104.075(2)
γ/°	90
Volume/Å^3	2369.56(6)
Z	3
ρ_{calc}g/cm^3	1.403
μ/mm^{-1}	0.694
F(000)	1043.4
Crystal size/mm^3	0.06 × 0.05 × 0.04
Radiation	CuKα (λ = 1.54184)
2Θ range for data collection/°	7.12 to 151.88
Index ranges	$-12 \le h \le 15$, $-13 \le k \le 16$, $-17 \le l \le 17$
Reflections collected	13617
Independent reflections	4808 [R_{int} = 0.0262, R_{sigma} = 0.0326]
Data/restraints/parameters	4808/0/355
Goodness-of-fit on F^2	1.017
Final R indexes [I>=2σ (I)]	R_1 = 0.0375, wR_2 = 0.0903
Final R indexes [all data]	R_1 = 0.0484, wR_2 = 0.0967
Largest diff. peak/hole / e Å^{-3}	0.24/-0.21

Datablock: Req148-1

Bond precision:	C-C = 0.0020 A		Wavelength=1.54184
Cell:	a=12.7952(2)	b=13.3698(2)	c=14.2802(2)
	alpha=90	beta=104.075(2)	gamma=90
Temperature:	100 K		

	Calculated	Reported
Volume	2369.56(6)	2369.56(6)
Space group	P 21/c	P 1 21/c 1
Hall group	-P 2ybc	-P 2ybc
Moiety formula	C35 H20 N2 O2	1.333(C35 H20 N2 O2)
Sum formula	C35 H20 N2 O2	C46.667 H26.667 N2.667 O2.667
Mr	500.53	667.41
Dx,g cm-3	1.403	1.403
Z	4	3
Mu (mm-1)	0.694	0.694
F000	1040.0	1043.4
F000'	1043.01	
h,k,lmax	16,16,17	15,16,17
Nref	4935	4808
Tmin,Tmax	0.959,0.973	0.692,1.000
Tmin'	0.959	

Correction method= # Reported T Limits: Tmin=0.692 Tmax=1.000
AbsCorr = MULTI-SCAN

Data completeness= 0.974 Theta(max)= 75.940

R(reflections)= 0.0375(3942) wR2(reflections)= 0.0967(4808)

S = 1.017 Npar= 355

The following ALERTS were generated. Each ALERT has the format
test-name_ALERT_alert-type_alert-level.
Click on the hyperlinks for more details of the test.

Alert level C

```
PLAT911_ALERT_3_C Missing FCF Refl Between Thmin & STh/L=    0.600         14 Report
```

Alert level G

```
FORMU01_ALERT_1_G  There is a discrepancy between the atom counts in the
            _chemical_formula_sum and _chemical_formula_moiety. This is
            usually due to the moiety formula being in the wrong format.
            Atom count from _chemical_formula_sum:   C46.66699 H26.66699 N2.667 O2
            Atom count from _chemical_formula_moiety:C46.65499 H26.66 N2.666 O2.66
PLAT005_ALERT_5_G No Embedded Refinement Details Found  in the CIF     Please Do !
PLAT045_ALERT_1_G Calculated and Reported Z Differ by a Factor ...      1.333 Check
PLAT068_ALERT_1_G Reported F000 Differs from Calcd (or Missing)...     Please Check
PLAT073_ALERT_1_G H-atoms ref, but _hydrogen_treatment Reported as     constr Check
PLAT230_ALERT_2_G Hirshfeld Test Diff for    C015      --C025     .       5.4 s.u.
PLAT398_ALERT_2_G Deviating  C-O-C   Angle From 120 for O001      .     105.4 Degree
PLAT720_ALERT_4_G Number of Unusual/Non-Standard Labels ..........        59 Note
PLAT769_ALERT_4_G CIF Embedded explicitly supplied scattering data     Please Note
PLAT912_ALERT_4_G Missing # of FCF Reflections Above STh/L=  0.600       109 Note
PLAT960_ALERT_3_G Number of Intensities with I  <   - 2*sig(I) ...         2 Check
PLAT978_ALERT_2_G Number C-C Bonds with Positive Residual Density.         8 Info
PLAT982_ALERT_1_G The  C-f'=    0.0192 Deviates from  IT-value =      0.0181 Check
PLAT982_ALERT_1_G The  N-f'=    0.0326 Deviates from  IT-value =      0.0311 Check
PLAT982_ALERT_1_G The  O-f'=    0.0524 Deviates from  IT-value =      0.0492 Check
PLAT983_ALERT_1_G The  O-f"=    0.0338 Deviates from  IT-Value =      0.0322 Check
```

---

0 **ALERT level A** = Most likely a serious problem - resolve or explain
0 **ALERT level B** = A potentially serious problem, consider carefully
1 **ALERT level C** = Check. Ensure it is not caused by an omission or oversight
16 **ALERT level G** = General information/check it is not something unexpected

8 ALERT type 1 CIF construction/syntax error, inconsistent or missing data
3 ALERT type 2 Indicator that the structure model may be wrong or deficient
2 ALERT type 3 Indicator that the structure quality may be low
3 ALERT type 4 Improvement, methodology, query or suggestion
1 ALERT type 5 Informative message, check

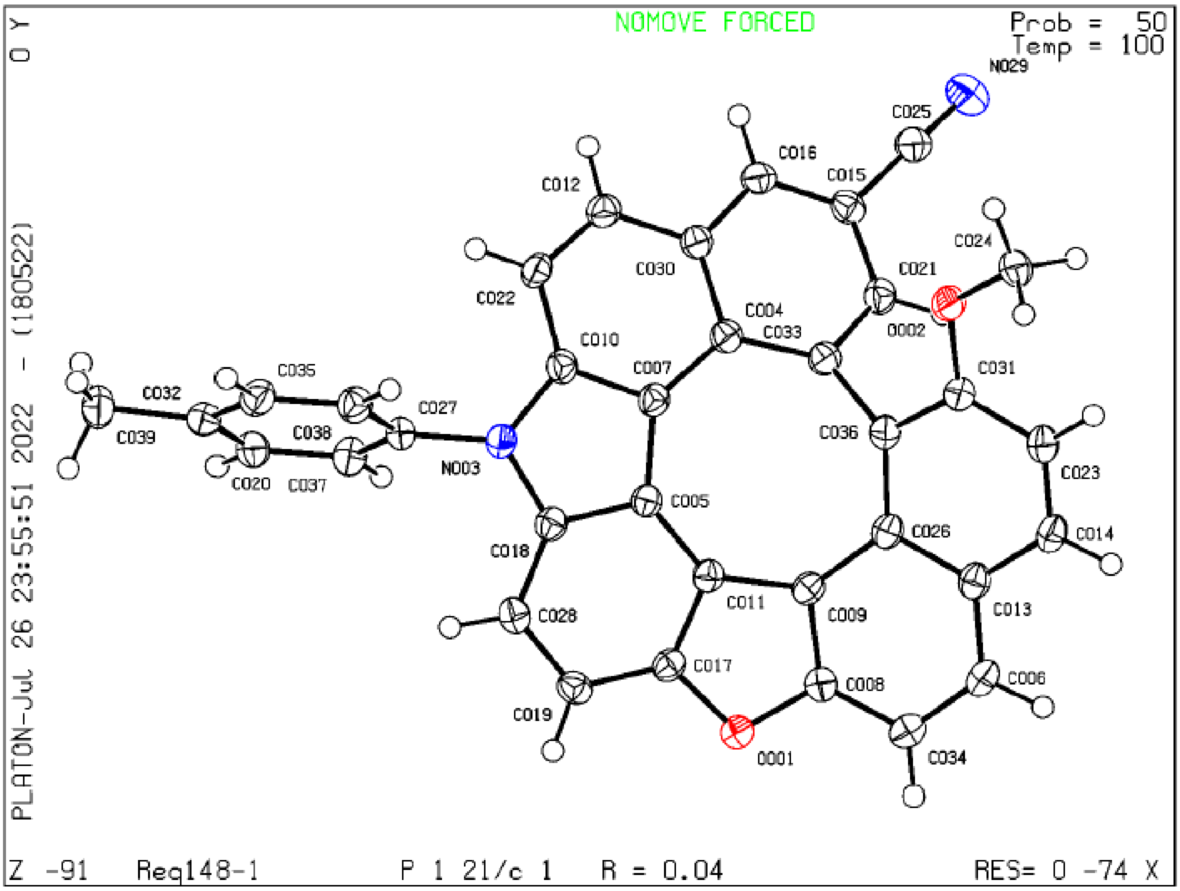
NOMOVE FORCED
Prob = 50
Temp = 100
0 Y
PLATON-Jul 26 23:55:51 2022 - (180522)
N029
C025
C016
C015
C012
C024
C030
C021
C022
C004
C033
O002
C010
C007
C031
C035
C032
C027
C036
C038
C039
N003
C023
C020
C037
C005
C026
C014
C018
C011
C009
C013
C028
C017
C006
C008
C019
O001
C034
Z -91
Req148-1
P 1 21/c 1
R = 0.04
RES= 0 -74 X

8 (CCDC 2183557) with ellipsoids at 30% probability. (H atoms were omitted for clarity).

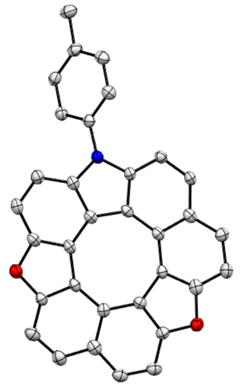

Empirical formula	$C_{33}H_{17}NO_2$
Formula weight	459.508
Temperature/K	100
Crystal system	triclinic
Space group	P-1
a/Å	8.0973(4)
b/Å	11.6061(5)
c/Å	11.6897(5)
α/°	77.674(3)
β/°	75.721(4)
γ/°	76.630(4)
Volume/Å^3	1021.42(8)
Z	2
ρ_{calc}g/cm^3	1.494
μ/mm^{-1}	0.736
F(000)	477.5
Crystal size/mm^3	0.13 × 0.03 × 0.02
Radiation	CuKα (λ = 1.54184)
2Θ range for data collection/°	7.92 to 152.68
Index ranges	$-10 \leq h \leq 9$, $-11 \leq k \leq 14$, $-14 \leq l \leq 14$
Reflections collected	10181
Independent reflections	4043 [R_{int} = 0.0342, R_{sigma} = 0.0524]
Data/restraints/parameters	4043/0/326
Goodness-of-fit on F^2	1.053
Final R indexes [I>=2σ (I)]	R_1 = 0.0670, wR_2 = 0.1839
Final R indexes [all data]	R_1 = 0.0831, wR_2 = 0.1994
Largest diff. peak/hole / e Å^{-3}	0.80/-0.34

Datablock: req214-1

Bond precision:	C-C = 0.0043 A		Wavelength=1.54184
Cell:	a=8.0973(4)	b=11.6061(5)	c=11.6897(5)
	alpha=77.674(3)	beta=75.721(4)	gamma=76.630(4)
Temperature: 100 K			

	Calculated	Reported
Volume	1021.42(8)	1021.42(8)
Space group	P -1	P -1
Hall group	-P 1	-P 1
Moiety formula	C33 H17 N O2	C33 H17 N O2
Sum formula	C33 H17 N O2	C33 H17 N O2
Mr	459.48	459.48
Dx,g cm-3	1.494	1.494
Z	2	2
Mu (mm-1)	0.736	0.736
F000	476.0	476.0
F000'	477.39	
h,k,lmax	10,14,14	10,14,14
Nref	4287	4043
Tmin,Tmax	0.974,0.985	0.751,1.000
Tmin'	0.909	

Correction method= # Reported T Limits: Tmin=0.751 Tmax=1.000 AbsCorr = MULTI-SCAN

Data completeness= 0.943 Theta(max)= 76.342

R(reflections)= 0.0675(3171) wR2(reflections)= 0.2246(4043)

S = 0.798 Npar= 326

The following ALERTS were generated. Each ALERT has the format
test-name_ALERT_alert-type_alert-level.
Click on the hyperlinks for more details of the test.

Alert level C

DIFMX02_ALERT_1_C The maximum difference density is > 0.1*ZMAX*0.75
The relevant atom site should be identified.
GOODF01_ALERT_2_C The least squares goodness of fit parameter lies
outside the range 0.80 <> 2.00
Goodness of fit given = 0.798
PLAT094_ALERT_2_C Ratio of Maximum / Minimum Residual Density .... 2.32 Report
PLAT097_ALERT_2_C Large Reported Max. (Positive) Residual Density 0.72 eA-3
PLAT230_ALERT_2_C Hirshfeld Test Diff for O1 --C019 . 6.9 s.u.

PLAT230_ALERT_2_C Hirshfeld Test Diff for C021 --C023 . 5.5 s.u.
PLAT340_ALERT_3_C Low Bond Precision on C-C Bonds .... 0.00429 Ang.
PLAT906_ALERT_3_C Large K Value in the Analysis of Variance ...... 4.054 Check
PLAT911_ALERT_3_C Missing FCF Refl Between Thmin & STh/L= 0.600 56 Report

Alert level G

PLAT005_ALERT_5_G No Embedded Refinement Details Found in the CIF Please Do !
PLAT072_ALERT_2_G SHELXL First Parameter in WGHT Unusually Large 0.17 Report
PLAT398_ALERT_2_G Deviating C-O-C Angle From 120 for O1 . 104.2 Degree
PLAT398_ALERT_2_G Deviating C-O-C Angle From 120 for O2 . 105.6 Degree
PLAT720_ALERT_4_G Number of Unusual/Non-Standard Labels .... 50 Note
PLAT912_ALERT_4_G Missing # of FCF Reflections Above STh/L= 0.600 189 Note
PLAT978_ALERT_2_G Number C-C Bonds with Positive Residual Density. 13 Info

0 **ALERT level A** = Most likely a serious problem - resolve or explain
0 **ALERT level B** = A potentially serious problem, consider carefully
9 **ALERT level C** = Check. Ensure it is not caused by an omission or oversight
7 **ALERT level G** = General information/check it is not something unexpected

1 ALERT type 1 CIF construction/syntax error, inconsistent or missing data
9 ALERT type 2 Indicator that the structure model may be wrong or deficient
3 ALERT type 3 Indicator that the structure quality may be low
2 ALERT type 4 Improvement, methodology, query or suggestion
1 ALERT type 5 Informative message, check

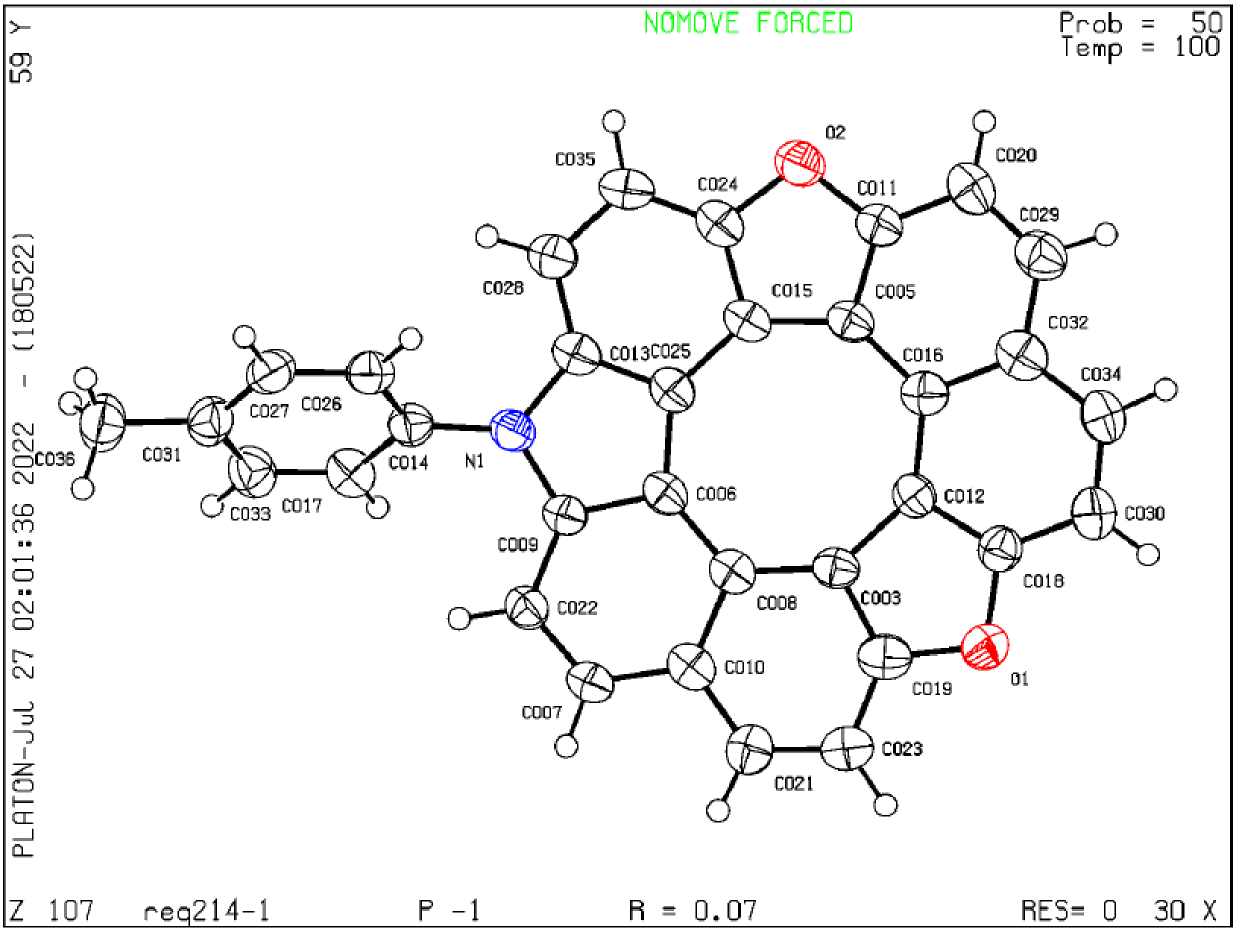

10aa (CCDC 2057234) with ellipsoids at 30% probability. (H atoms were omitted for clarity).

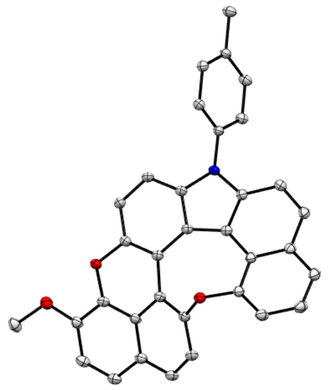

Empirical formula	$C_{34}H_{21}NO_3$
Formula weight	491.550
Temperature/K	100
Crystal system	triclinic
Space group	P-1
a/Å	7.8183(2)
b/Å	10.4498(3)
c/Å	14.6113(4)
α/°	90.337(2)
β/°	102.930(2)
γ/°	99.636(2)
Volume/Å^3	1145.89(6)
Z	2
ρ_{calc}g/cm^3	1.425
μ/mm^{-1}	0.725
F(000)	513.7
Crystal size/mm^3	0.106 × 0.073 × 0.039
Radiation	CuKα (λ = 1.54184)
2Θ range for data collection/°	6.22 to 151.76
Index ranges	$-9 \leq h \leq 9$, $-13 \leq k \leq 13$, $-18 \leq l \leq 18$
Reflections collected	24136
Independent reflections	4643 [R_{int} = 0.0476, R_{sigma} = 0.0348]
Data/restraints/parameters	4643/0/345
Goodness-of-fit on F^2	1.041
Final R indexes [I>=2σ (I)]	R_1 = 0.0414, wR_2 = 0.1052
Final R indexes [all data]	R_1 = 0.0503, wR_2 = 0.1110
Largest diff. peak/hole / e Å^{-3}	0.21/-0.23

Datablock: No2020-7-3

Bond precision: C-C = 0.0020 A Wavelength=1.54184

Cell:	a=7.8183(2)	b=10.4498(3)	c=14.6113(4)
	alpha=90.337(2)	beta=102.930(2)	gamma=99.636(2)
Temperature:	100 K		

	Calculated	Reported
Volume	1145.89(6)	1145.89(6)
Space group	P -1	P -1
Hall group	-P 1	-P 1
Moiety formula	C34 H21 N O3	C34 H21 N O3
Sum formula	C34 H21 N O3	C34 H21 N O3
Mr	491.52	491.55
Dx,g cm-3	1.425	1.425
Z	2	2
Mu (mm-1)	0.725	0.725
F000	512.0	513.7
F000'	513.52	
h,k,lmax	9,13,18	9,13,18
Nref	4768	4643
Tmin,Tmax	0.938,0.972	0.862,1.000
Tmin'	0.926	

Correction method= # Reported T Limits: Tmin=0.862 Tmax=1.000
AbsCorr = MULTI-SCAN

Data completeness= 0.974 Theta(max)= 75.880

R(reflections)= 0.0414(3881) wR2(reflections)= 0.1110(4643)

S = 1.041 Npar= 345

```
The following ALERTS were generated. Each ALERT has the format
       test-name_ALERT_alert-type_alert-level.
Click on the hyperlinks for more details of the test.
```

Alert level C

```
PLAT906_ALERT_3_C Large K Value in the Analysis of Variance ......      2.296 Check
PLAT911_ALERT_3_C Missing FCF Refl Between Thmin & STh/L=     0.600        15 Report
```

Alert level G

```
PLAT005_ALERT_5_G No Embedded Refinement Details Found  in the CIF     Please Do !
PLAT068_ALERT_1_G Reported F000 Differs from Calcd (or Missing)...     Please Check
PLAT073_ALERT_1_G H-atoms ref, but _hydrogen_treatment Reported as     constr Check
PLAT154_ALERT_1_G The s.u.'s on the Cell Angles are Equal ..(Note)      0.002 Degree
PLAT720_ALERT_4_G Number of Unusual/Non-Standard Labels ..........         59 Note
PLAT769_ALERT_4_G CIF Embedded explicitly supplied scattering data     Please Note
PLAT912_ALERT_4_G Missing # of FCF Reflections Above STh/L=  0.600        111 Note
PLAT978_ALERT_2_G Number C-C Bonds with Positive Residual Density.          7 Info
PLAT982_ALERT_1_G The  C-f'=     0.0192 Deviates from  IT-value =      0.0181 Check
PLAT982_ALERT_1_G The  N-f'=     0.0326 Deviates from  IT-value =      0.0311 Check
PLAT982_ALERT_1_G The  O-f'=     0.0524 Deviates from  IT-value =      0.0492 Check
PLAT983_ALERT_1_G The  O-f"=     0.0338 Deviates from  IT-Value =      0.0322 Check
```

```
   0 ALERT level A = Most likely a serious problem - resolve or explain
   0 ALERT level B = A potentially serious problem, consider carefully
   2 ALERT level C = Check. Ensure it is not caused by an omission or oversight
  12 ALERT level G = General information/check it is not something unexpected

   7 ALERT type 1 CIF construction/syntax error, inconsistent or missing data
   1 ALERT type 2 Indicator that the structure model may be wrong or deficient
   2 ALERT type 3 Indicator that the structure quality may be low
   3 ALERT type 4 Improvement, methodology, query or suggestion
   1 ALERT type 5 Informative message, check
```

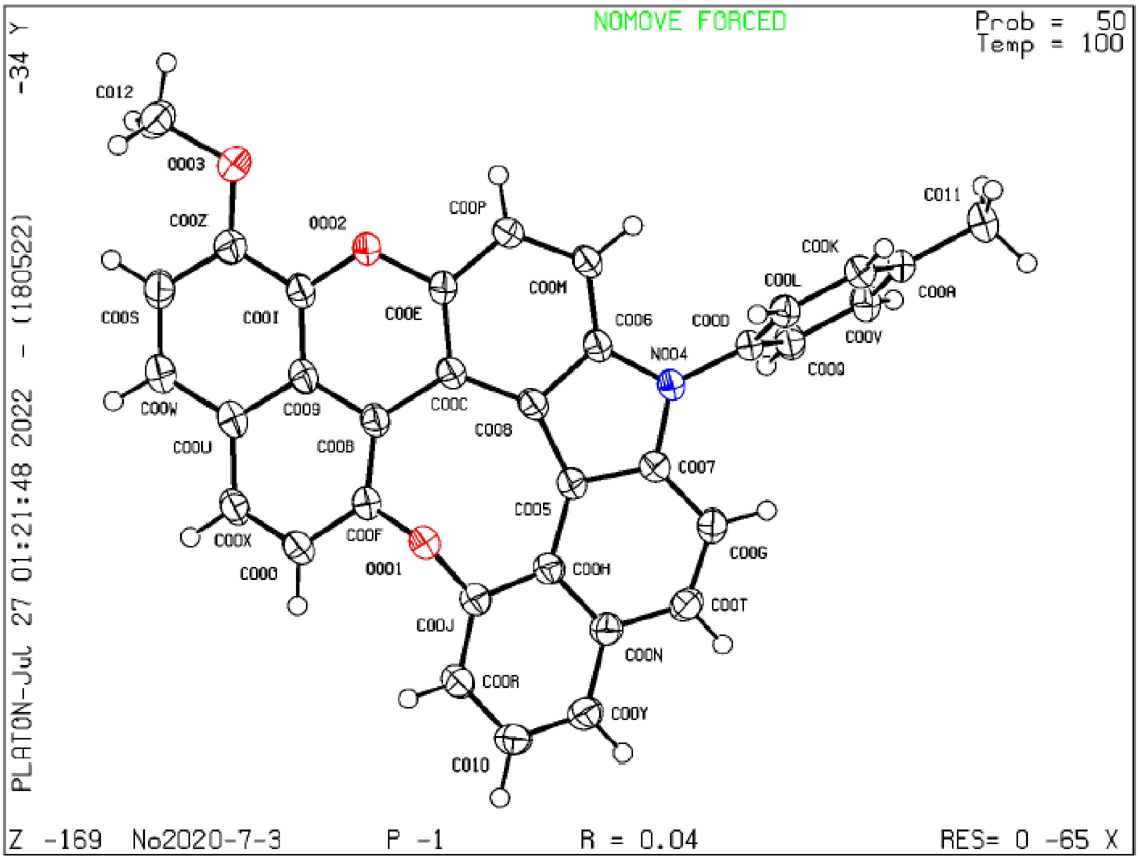

15. Supplementary References

1) Sako, M.; Higashida, K.; Kamble, G. T.; Kaut, K.; Kumar, A.; Hirose, Y.; Zhou, D.; Suzuki, T.; Rueping, M.; Maegawa, T.; Takizawa, S.; Sasai, H. *Org. Chem. Front.* **2021**, *8*, 4878–4885.
2) Kamble, G. T.; Salem, M. S. H.; Abe, T.; Park, H.; Sako, M.; Takizawa, S.; Sasai, H. *Chem. Lett.* **2021**, *50*, 1755–1757.
3) Sako, M.; Takeuchi, Y.; Tsujihara, T.; Kodera, J.; Kawano, T.; Takizawa, S.; Sasai, H. *J. Am. Chem. Soc.* **2016**, *138*, 11481–11484.
4) Liang, W.; Yang, Y.; Yang, M.; Zhang, M.; Li, C.; Ran, Y.; Lan, J.; Bin, Z.; You, J. *Angew. Chem. Int. Ed.* **2021**, *60*, 3493–3497.
5) Taniguchi, T.; Fukuba, T. A.; Nakatsuka, S.; Hayase, S.; Kawatsura, M.; Uno, H.; Itoh, T. *J. Org. Chem.* **2008**, *73*, 3875–3884.
6) Kamble, S. B.; Maliekal, P. J.; Dharpure, P. D.; Badani, P. M.; Karnik, A. V. *J. Org. Chem.* **2020**, *85*, 7739–7747.
7) Mishra, A. K.; Biswas, S. *J. Org. Chem.* **2016**, *81*, 2355–2363.
8) Qiu, H.; Shuai, B.; Wang, Y. Z.; Liu, D.; Chen, Y. G.; Gao, P. S.; Ma, H. X.; Chen, S. Mei, T. S. *J. Am. Chem. Soc.* **2020**, *142*, 9872–9878.
9) Holmes, A. B.; Park, T. Electroactive polyarylamine-type compositions **2002**, WO2002051958A1.
10) Akter, A.; Hossain, M. S.; Kurihara, T.; Iimura, K.; Karikomi, M. *Synlett* **2022**, *33*, 952-958.
11) Hossain, M. S.; Akter, M.; Shahabuddin, M.; Salim, M.; Iimura, K. I.; Karikomi, M. Synlett **2022**, *33*, 277-282.
