# Supplementary material for: Electrochemical synthesis of heterodehydro[7]helicenes
Source: Commun Chem. 2022 Dec 3;5:166. doi: 10.1038/s42004-022-00780-7 (PMC9814689; doi:10.1038/s42004-022-00780-7)
Supplement: Supplementary file 3 — Description of Additional Supplementary Files [file 42004_2022_780_MOESM3_ESM.pdf]

Description of Additional Supplementary Files

File name: Supplementary Data 1

Description: NMR Spectra

File name: Supplementary Data 2

Description: CIF of compound 3aa

File name: Supplementary Data 3

Description: CIF of compound 3ma

File name: Supplementary Data 4

Description: CIF of compound 5aa

File name: Supplementary Data 5

Description: CIF of compound 8

File name: Supplementary Data 6

Description: CIF of compound 10aa
