# Supplementary material for: Electrochemical synthesis of heterodehydro[7]helicenes
Source: Commun Chem. 2022 Dec 3;5:166. doi: 10.1038/s42004-022-00780-7 (PMC9814689; doi:10.1038/s42004-022-00780-7)
Supplement: Supplementary file 4 — Supplementary data 1 [file 42004_2022_780_MOESM4_ESM.pdf]

NMR Spectra

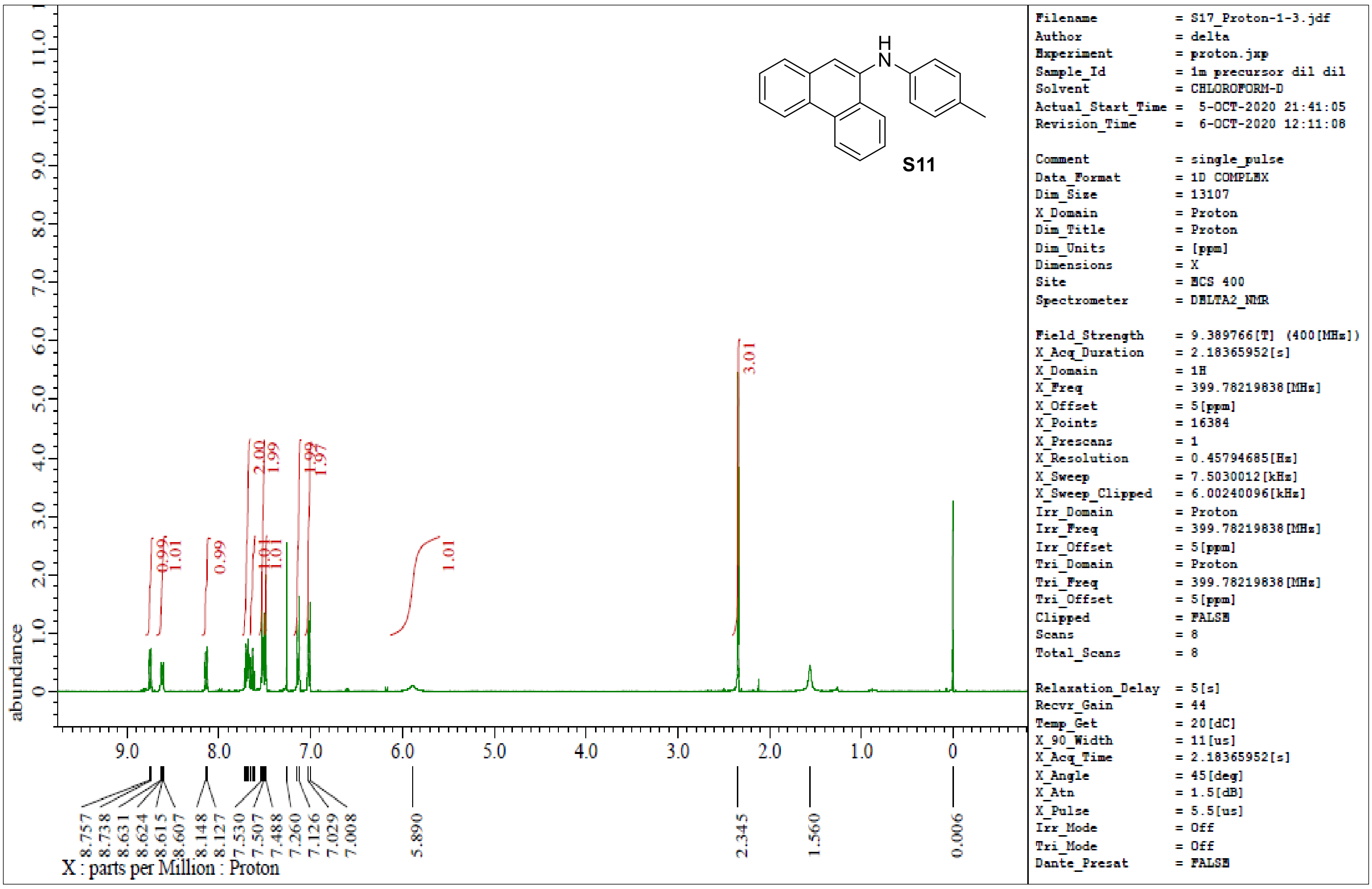

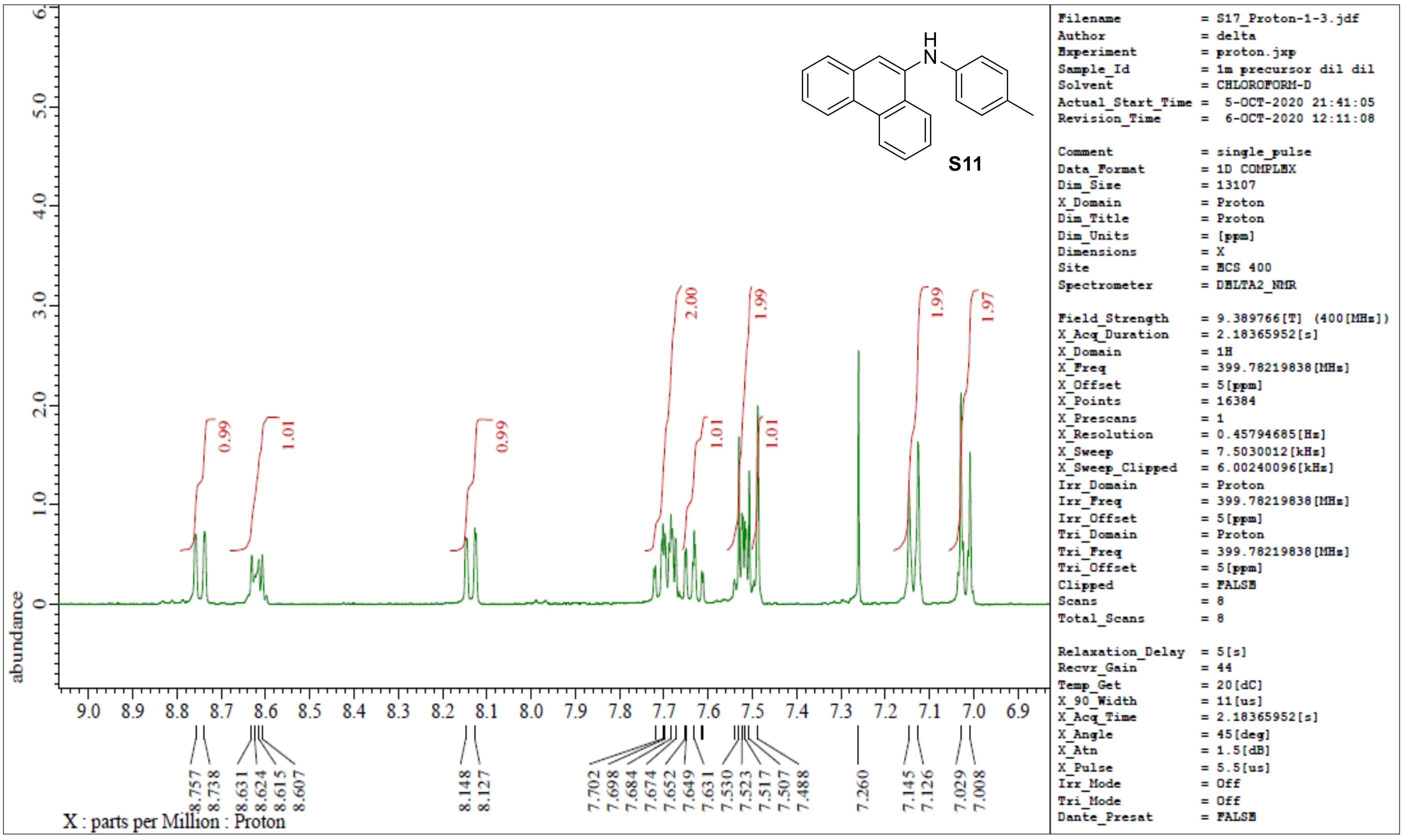

Compound **S11** (^{1}H NMR, 400 MHz, $CDCl_3$).

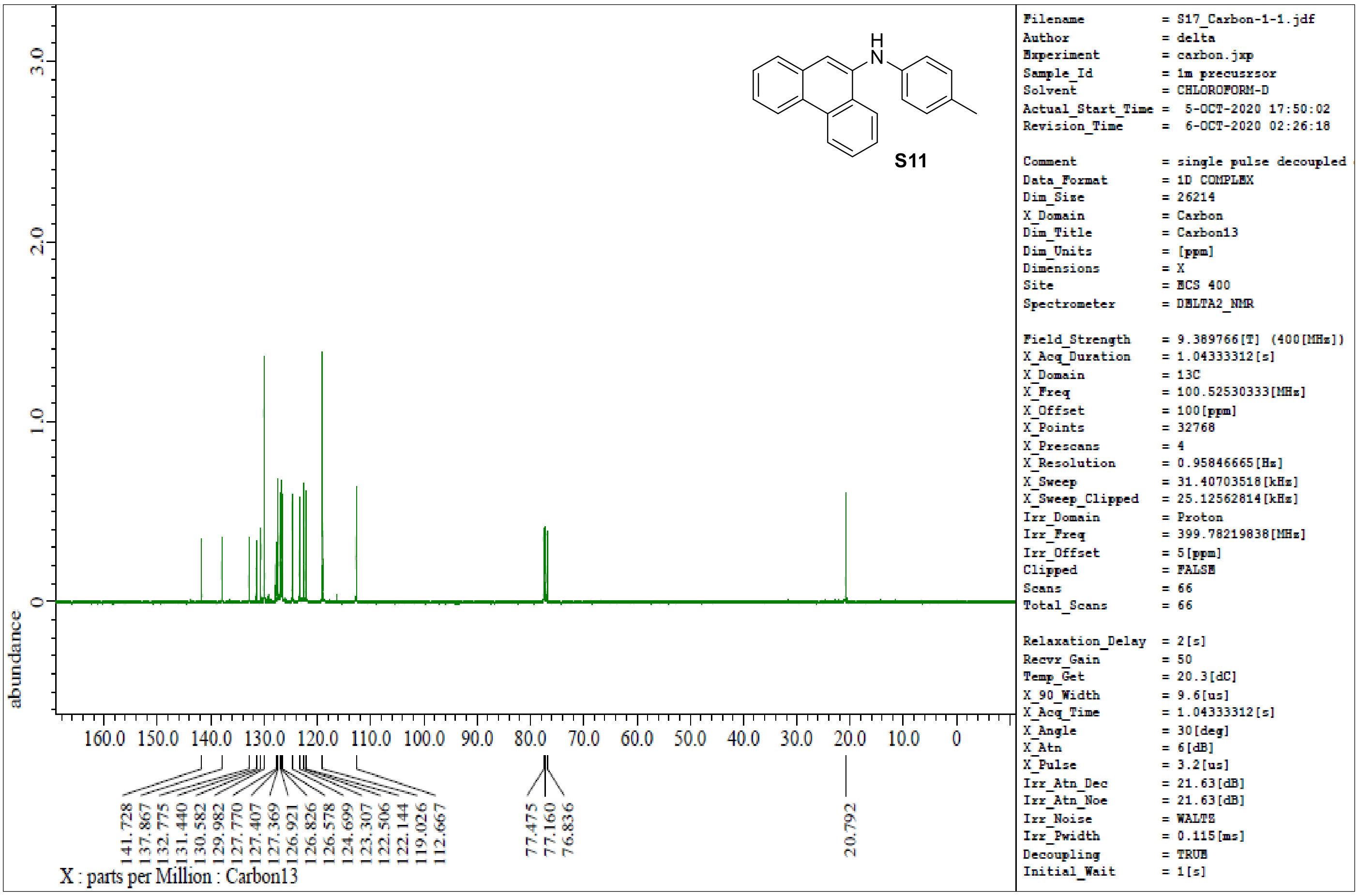

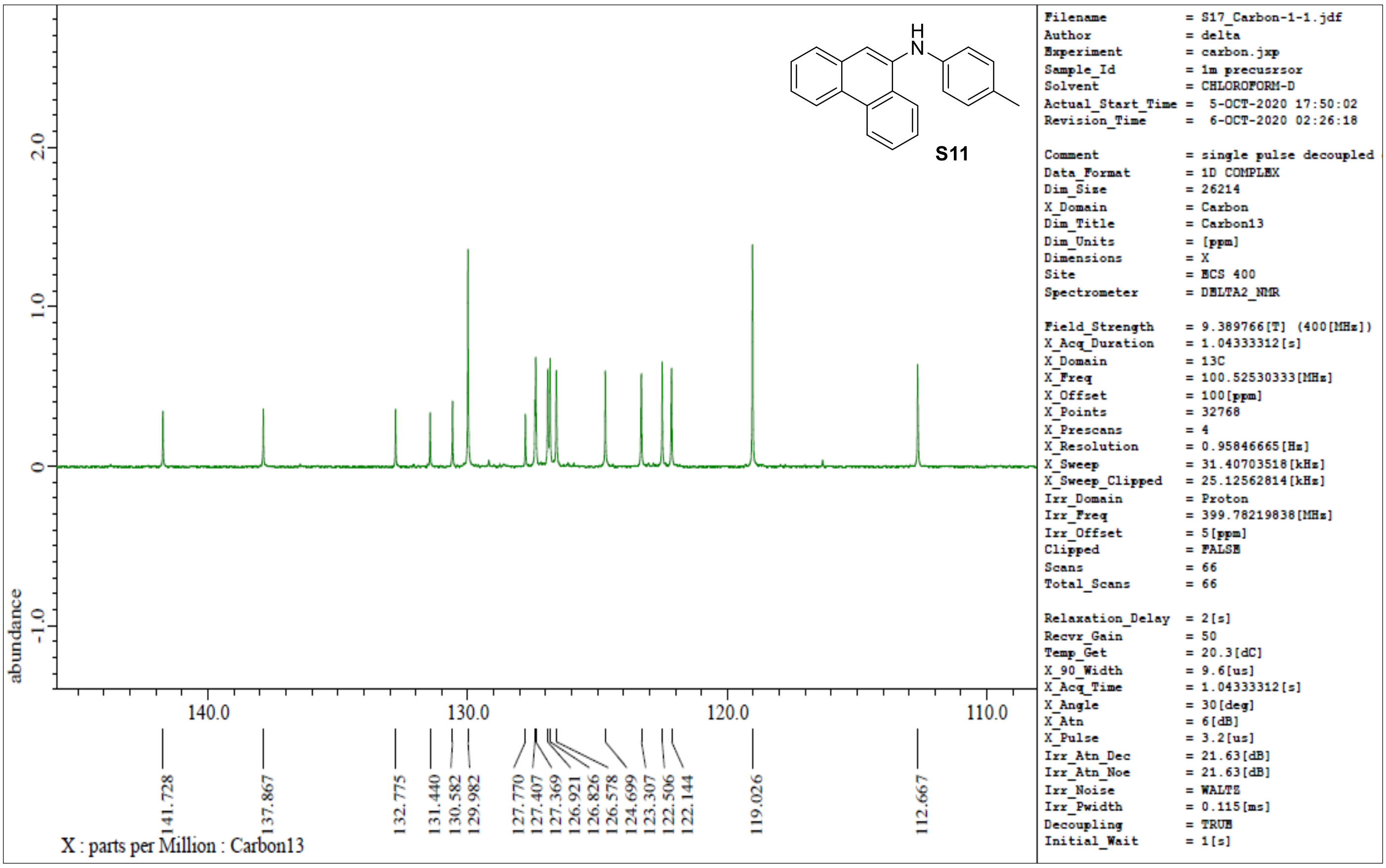

Compound **S11** (^{13}C NMR, 100 MHz, $CDCl_3$).

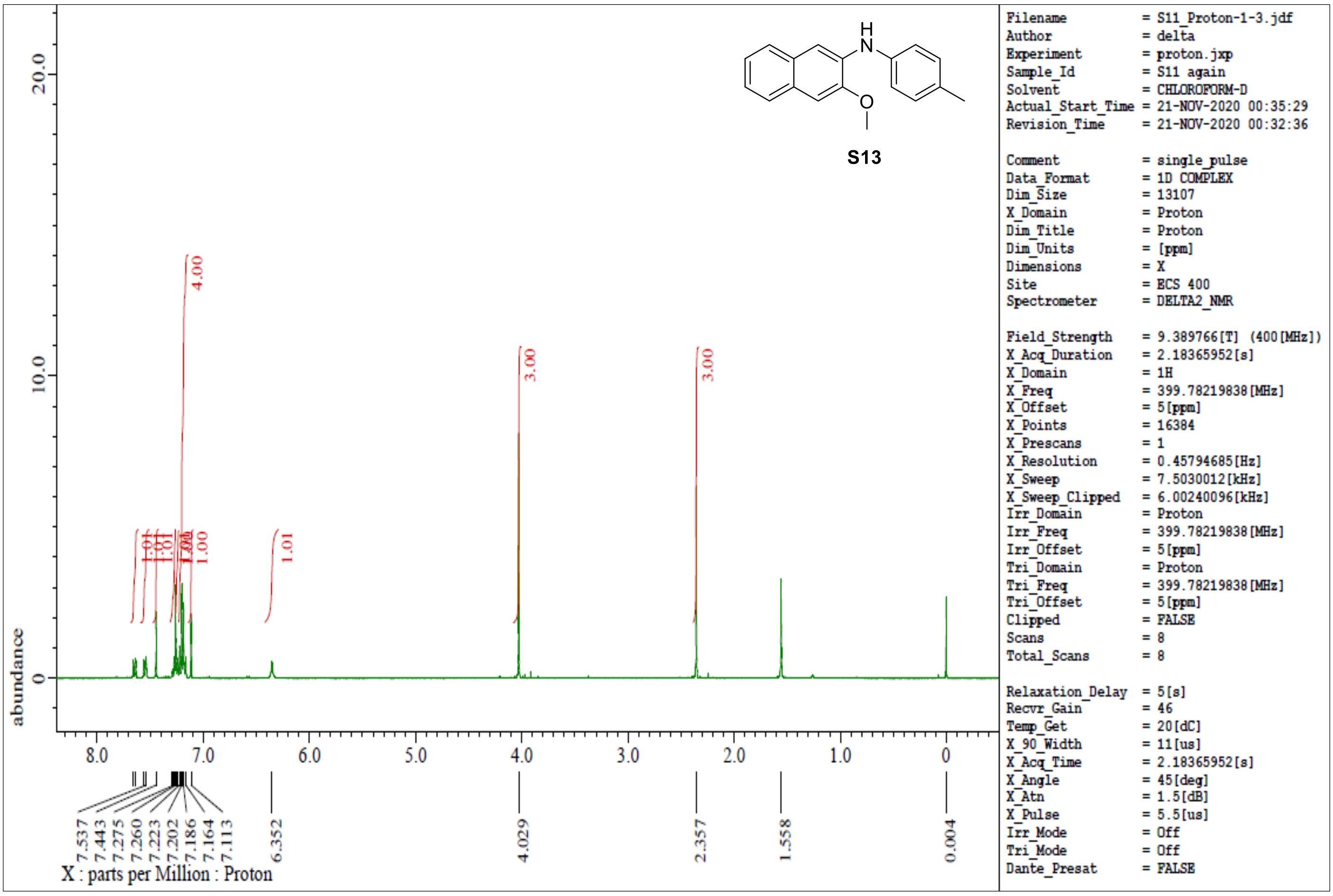

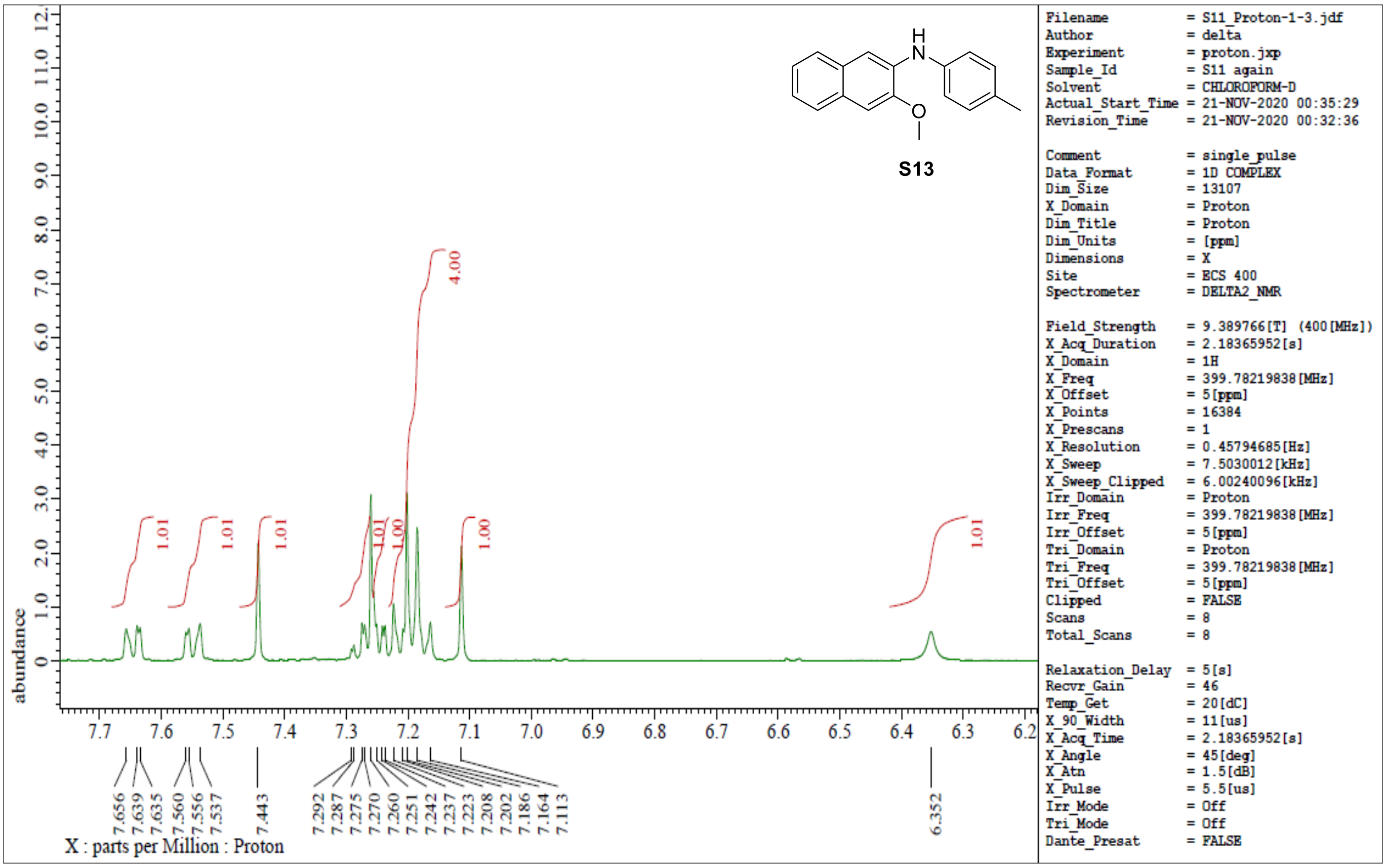

Compound **S13** (^{1}H NMR, 400 MHz, $CDCl_3$).

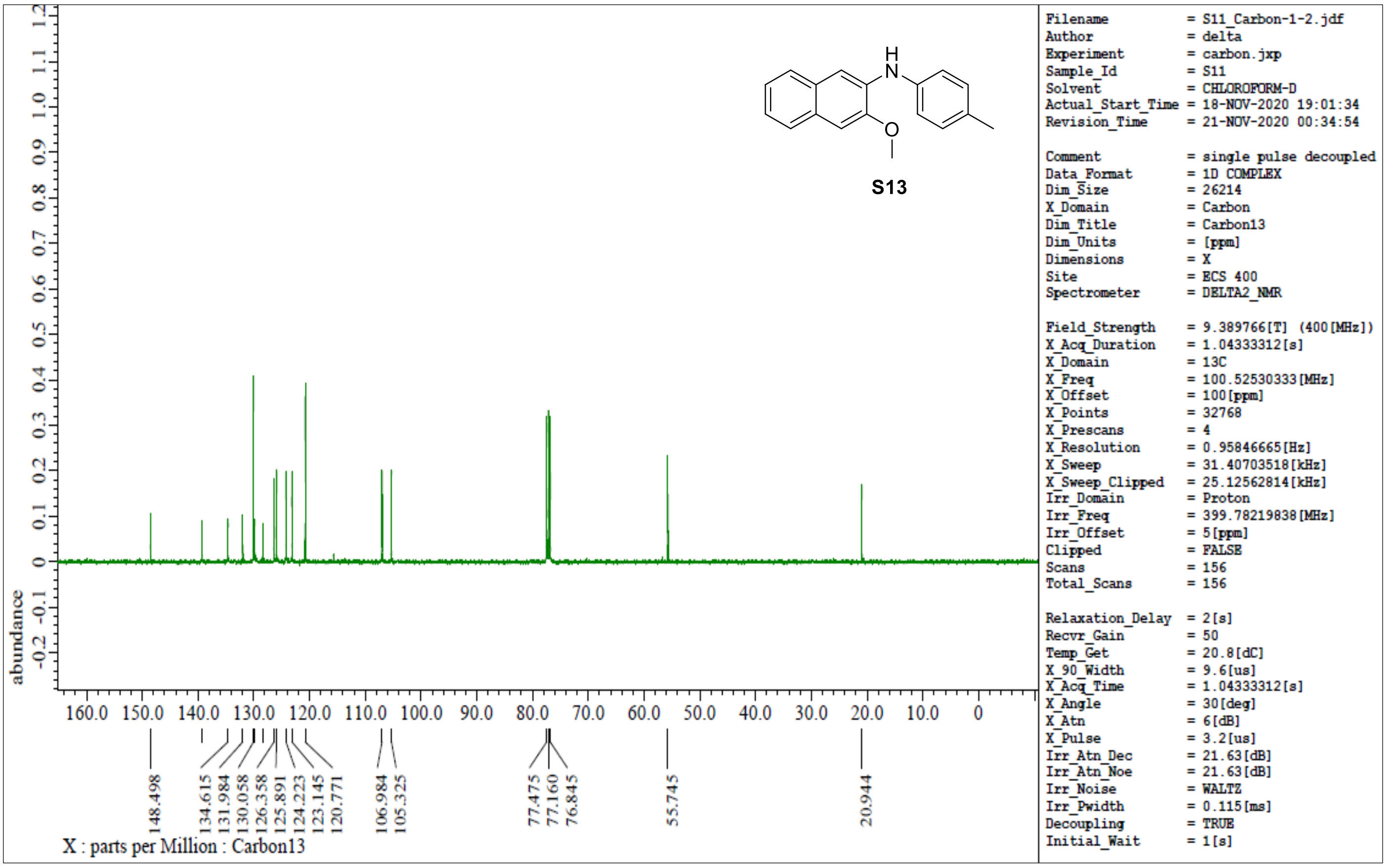

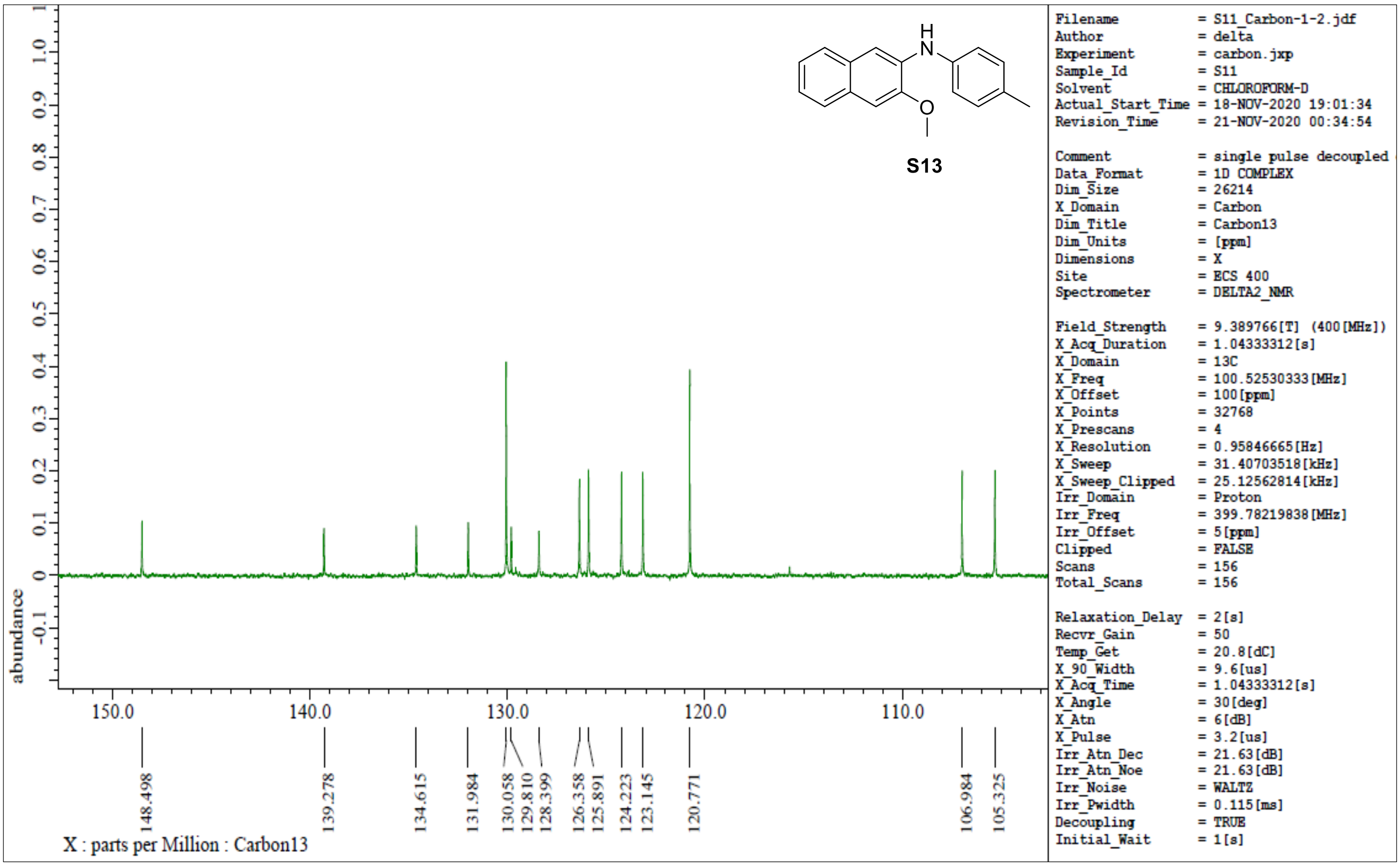

Compound **S13** (^{13}C NMR, 100 MHz, $CDCl_3$).

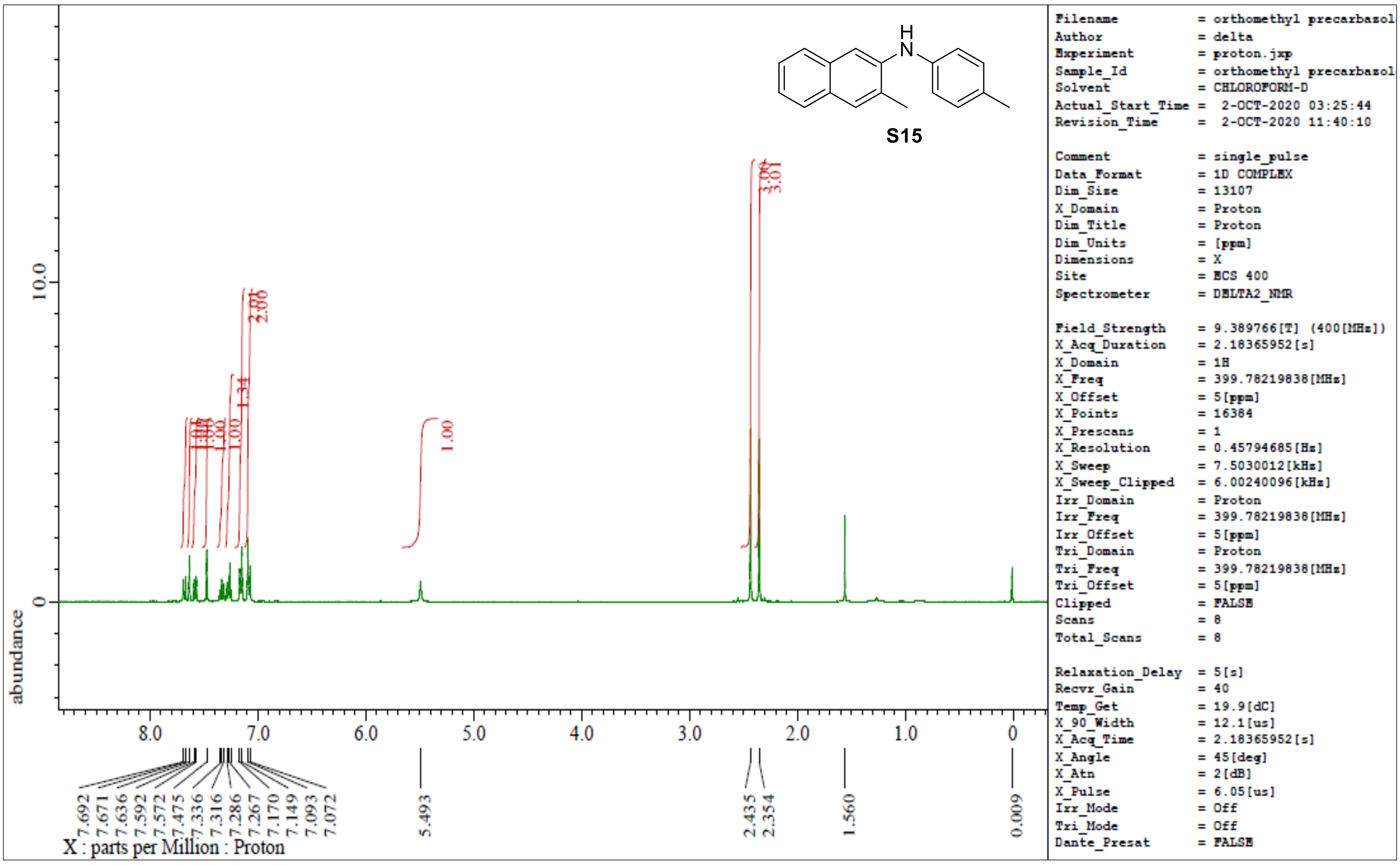

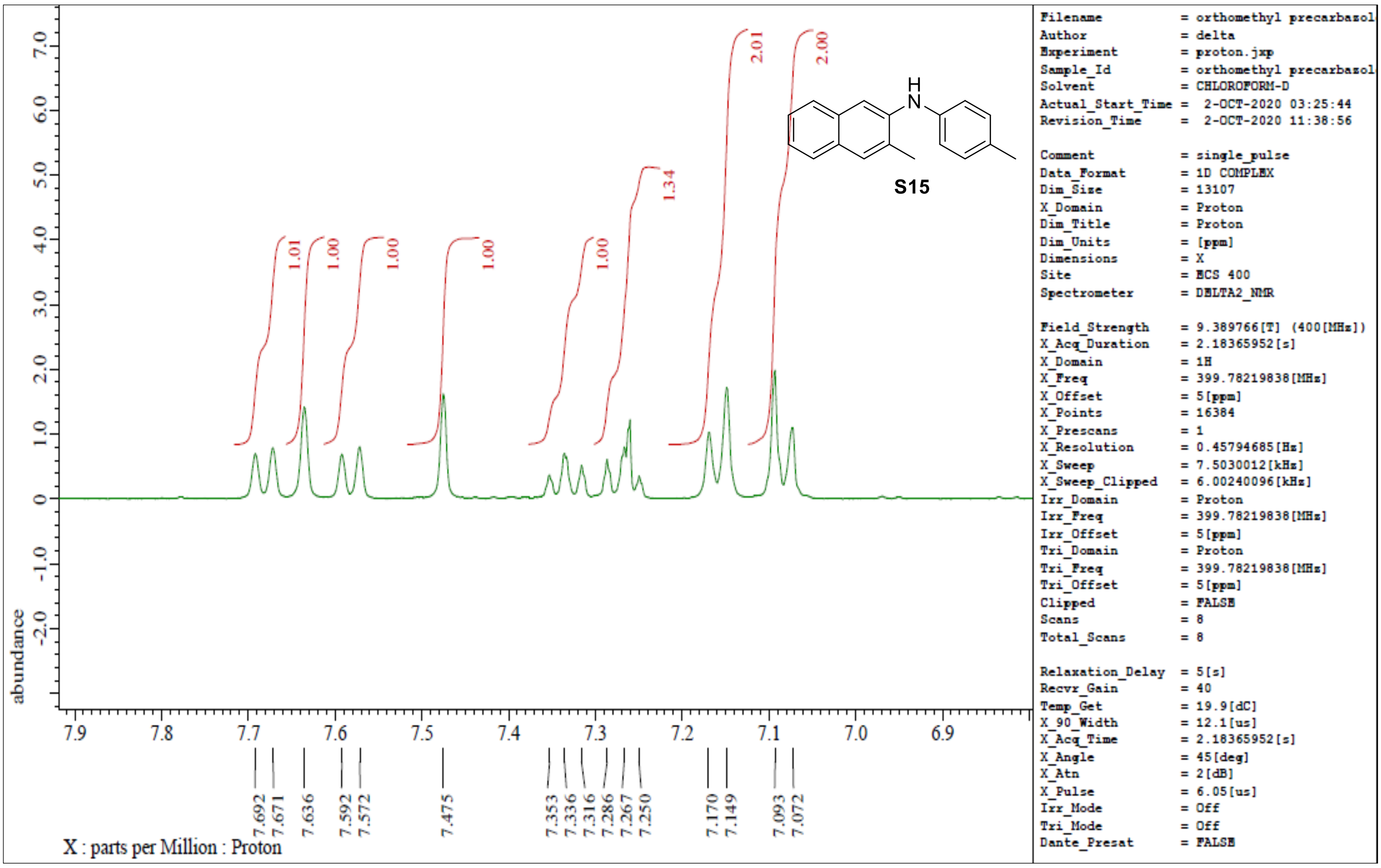

Compound **S15** (^{1}H NMR, 400 MHz, $CDCl_3$).

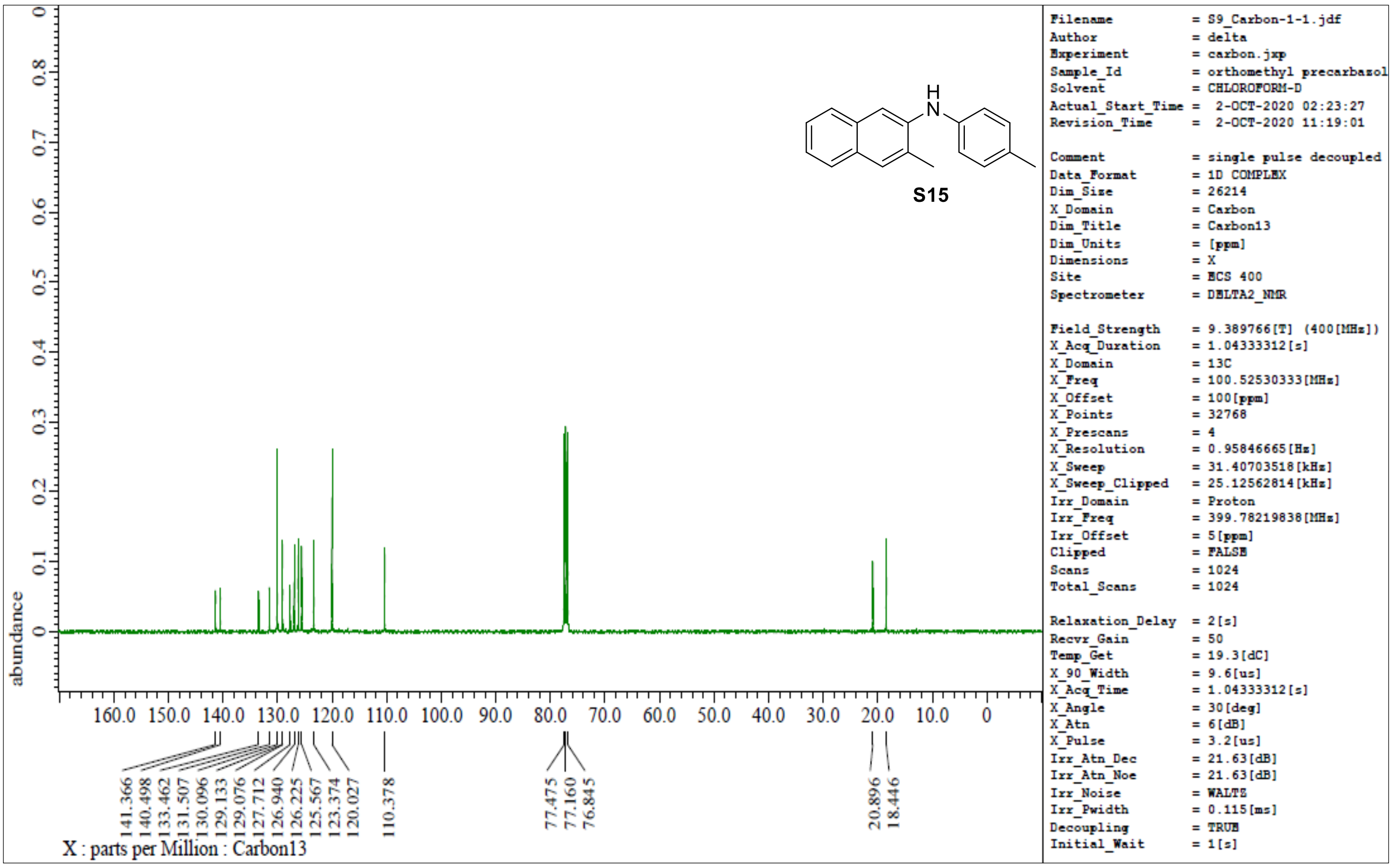

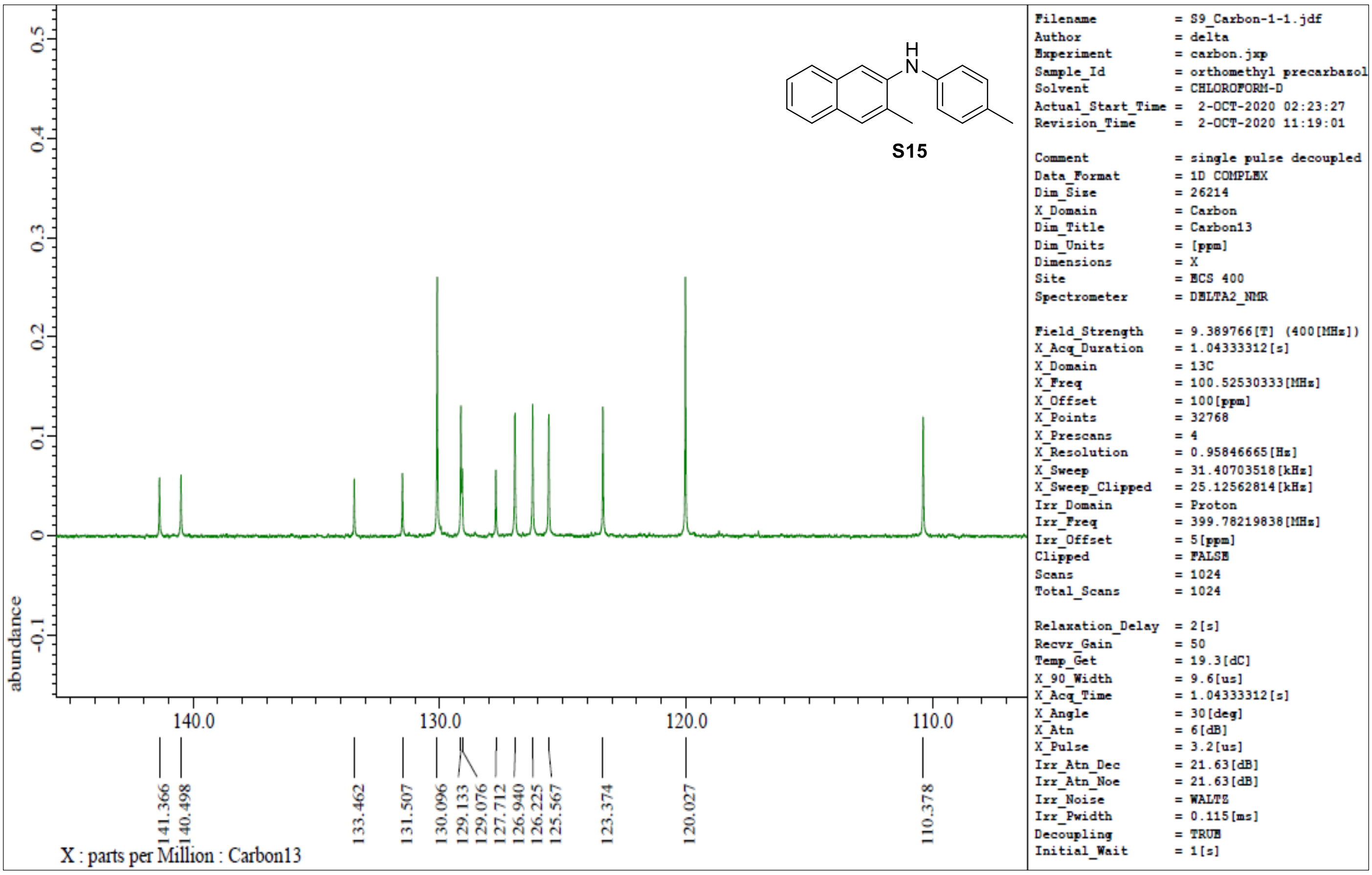

Compound **S15** (^{13}C NMR, 100 MHz, $CDCl_3$).

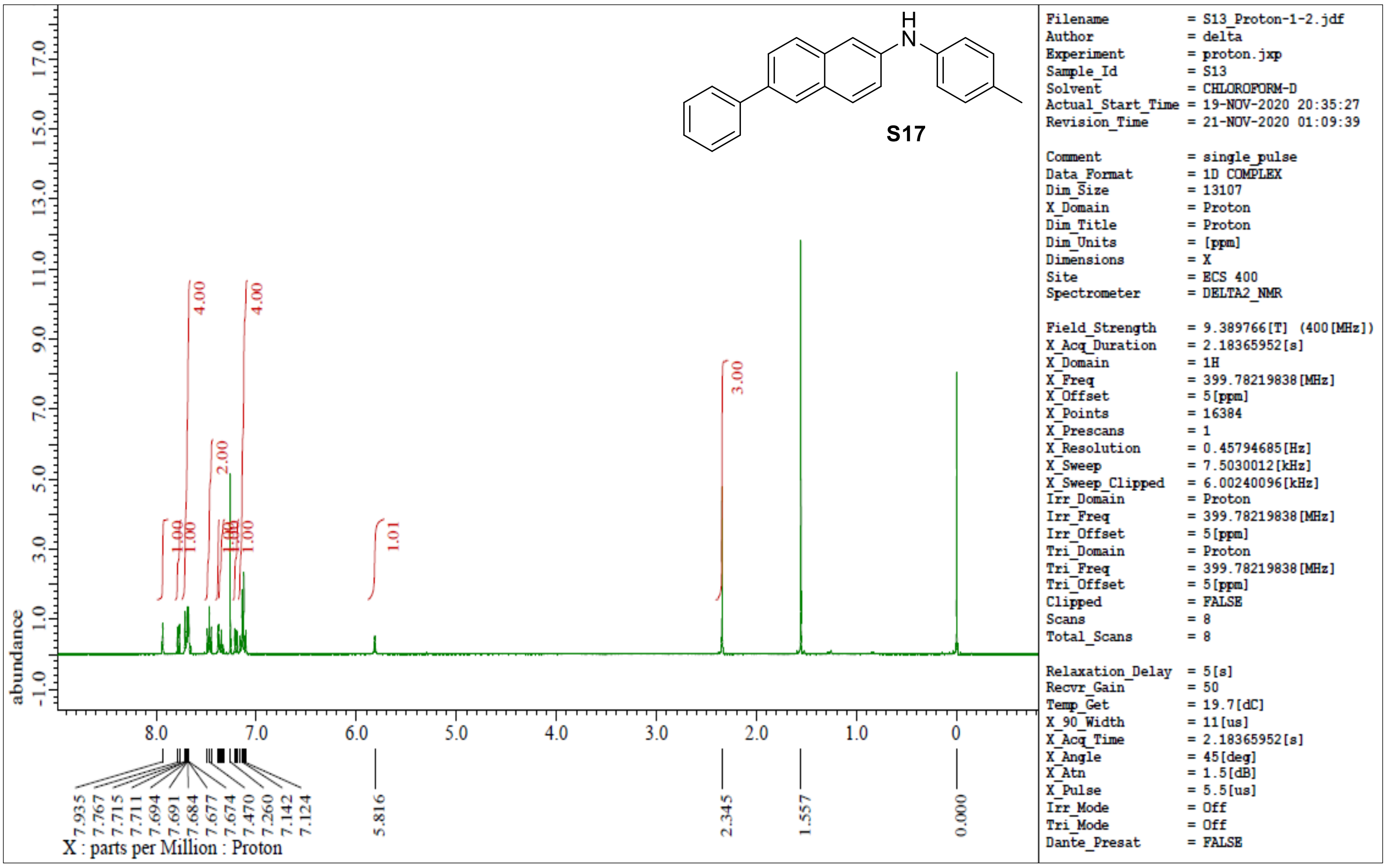

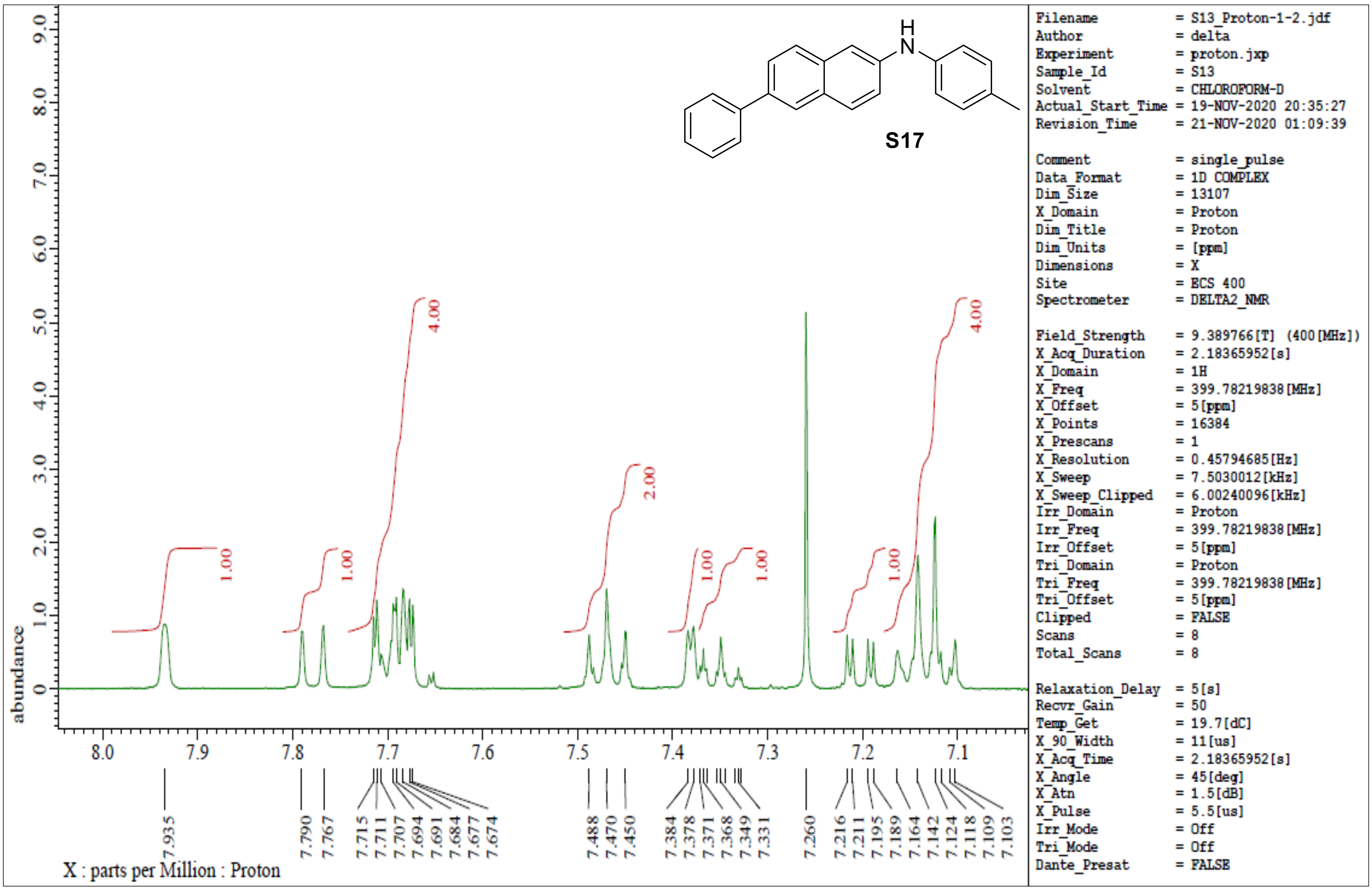

Compound **S17** (^{1}H NMR, 400 MHz, $CDCl_3$).

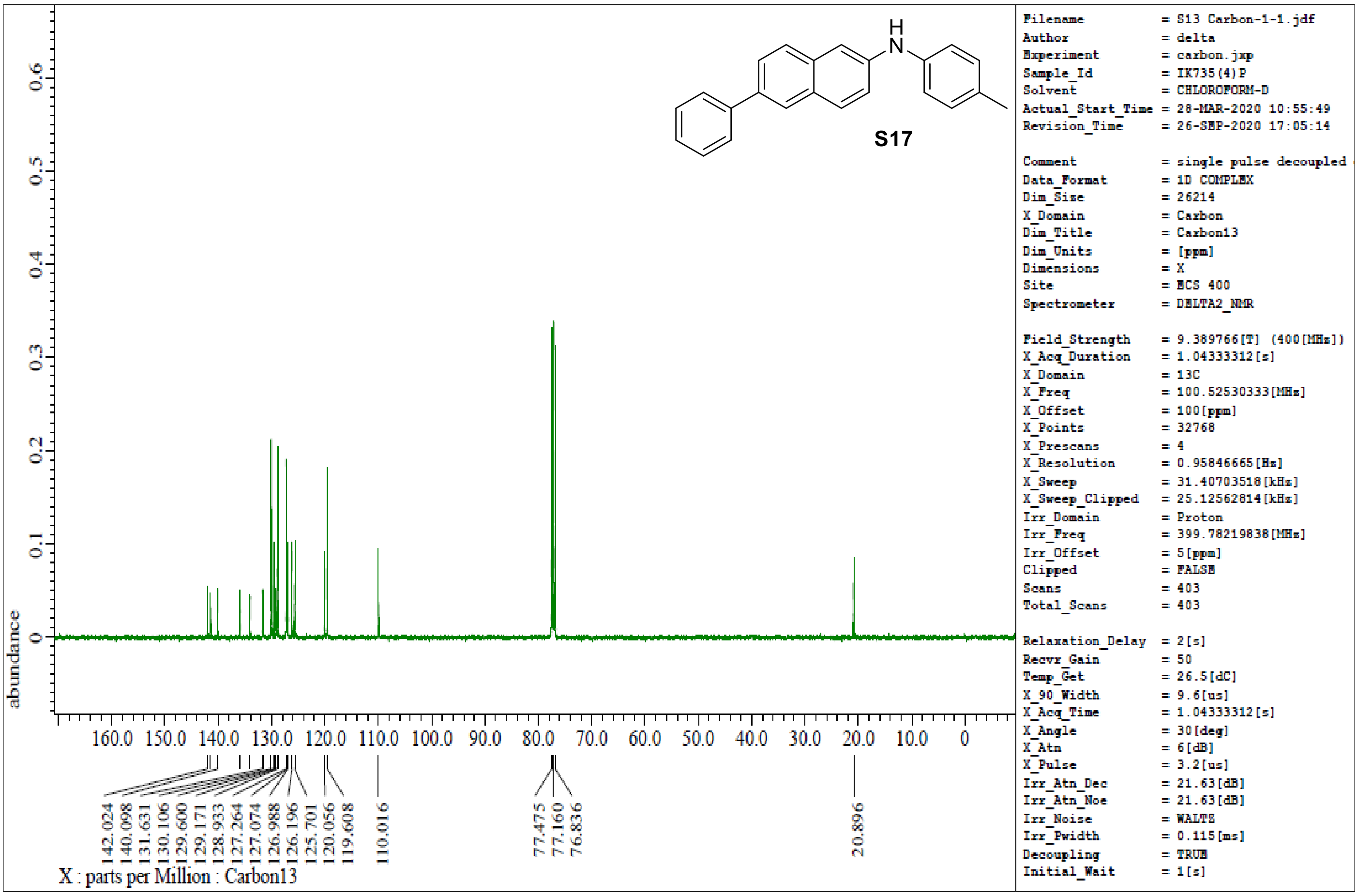

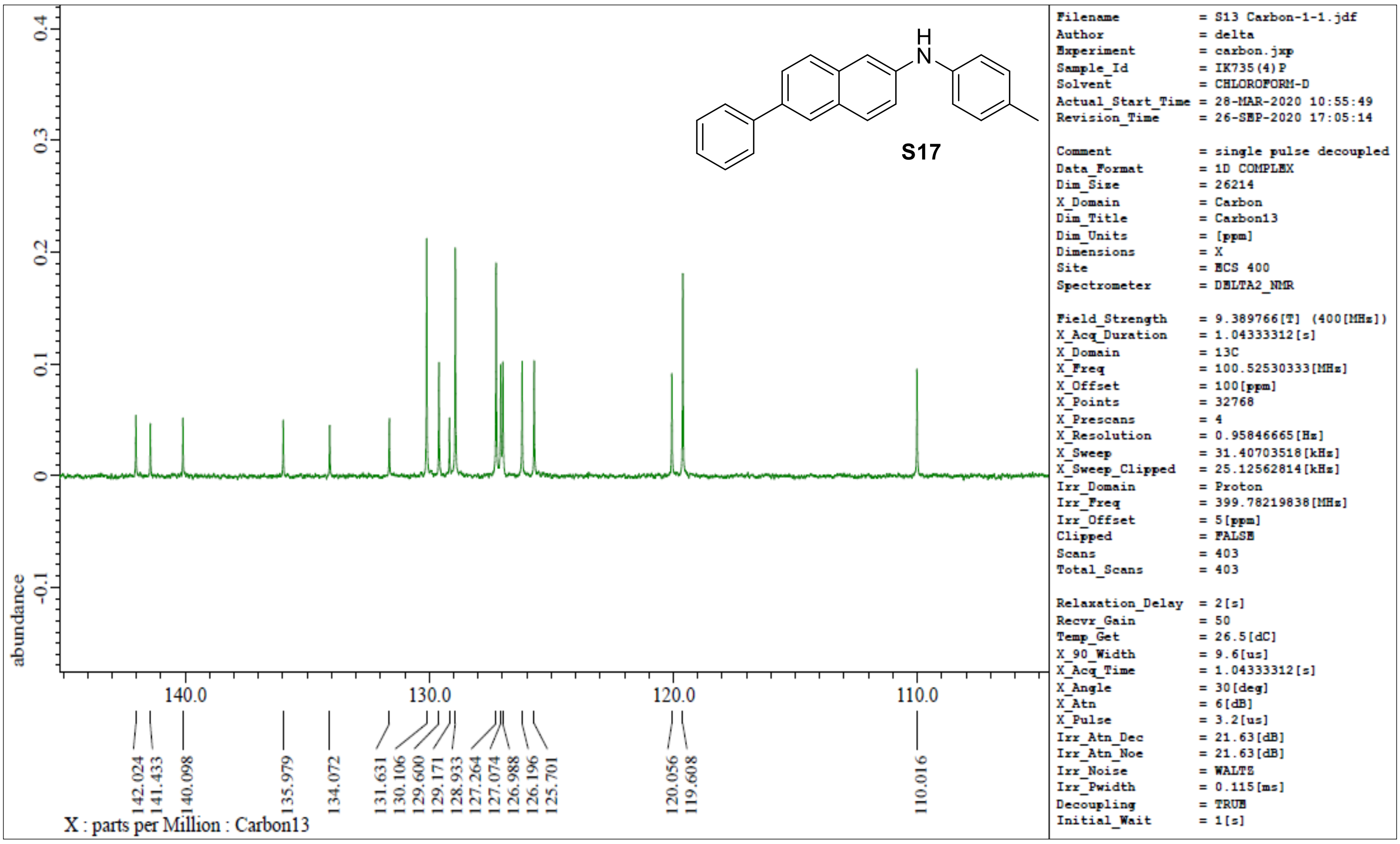

Compound **S17** (^{13}C NMR, 100 MHz, $CDCl_3$).

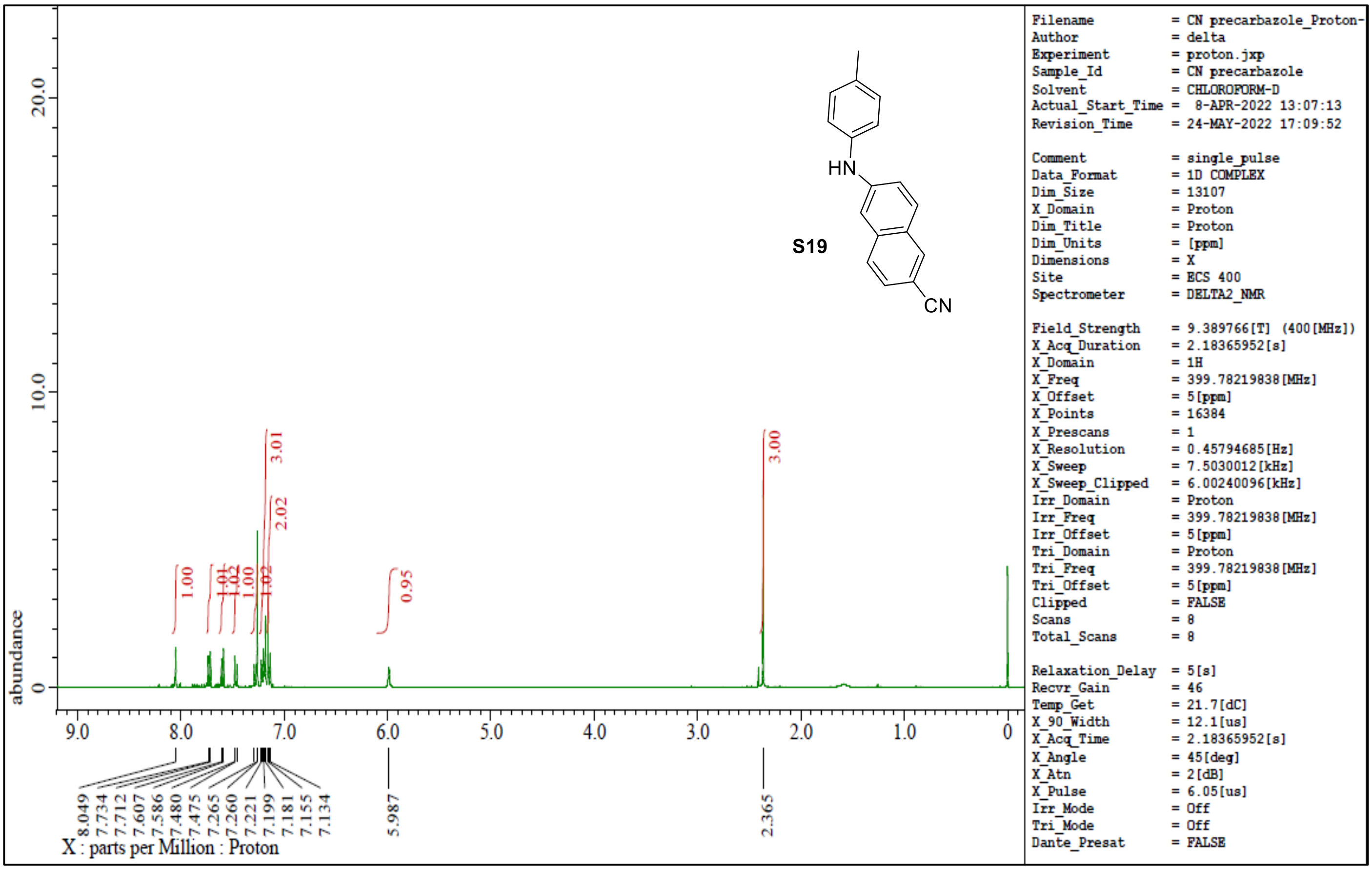

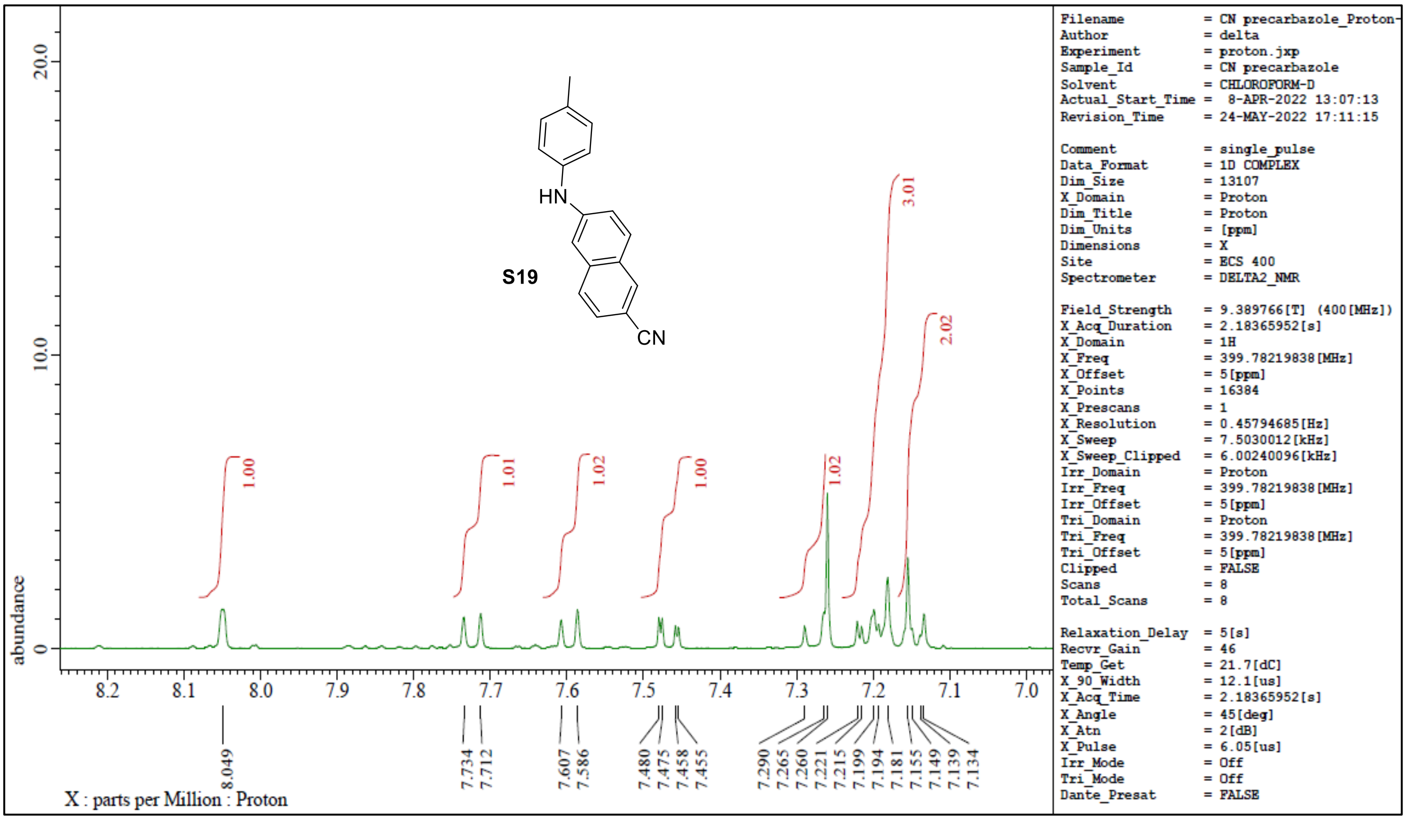

Compound **S19** (^{1}H NMR, 400 MHz, $CDCl_3$).

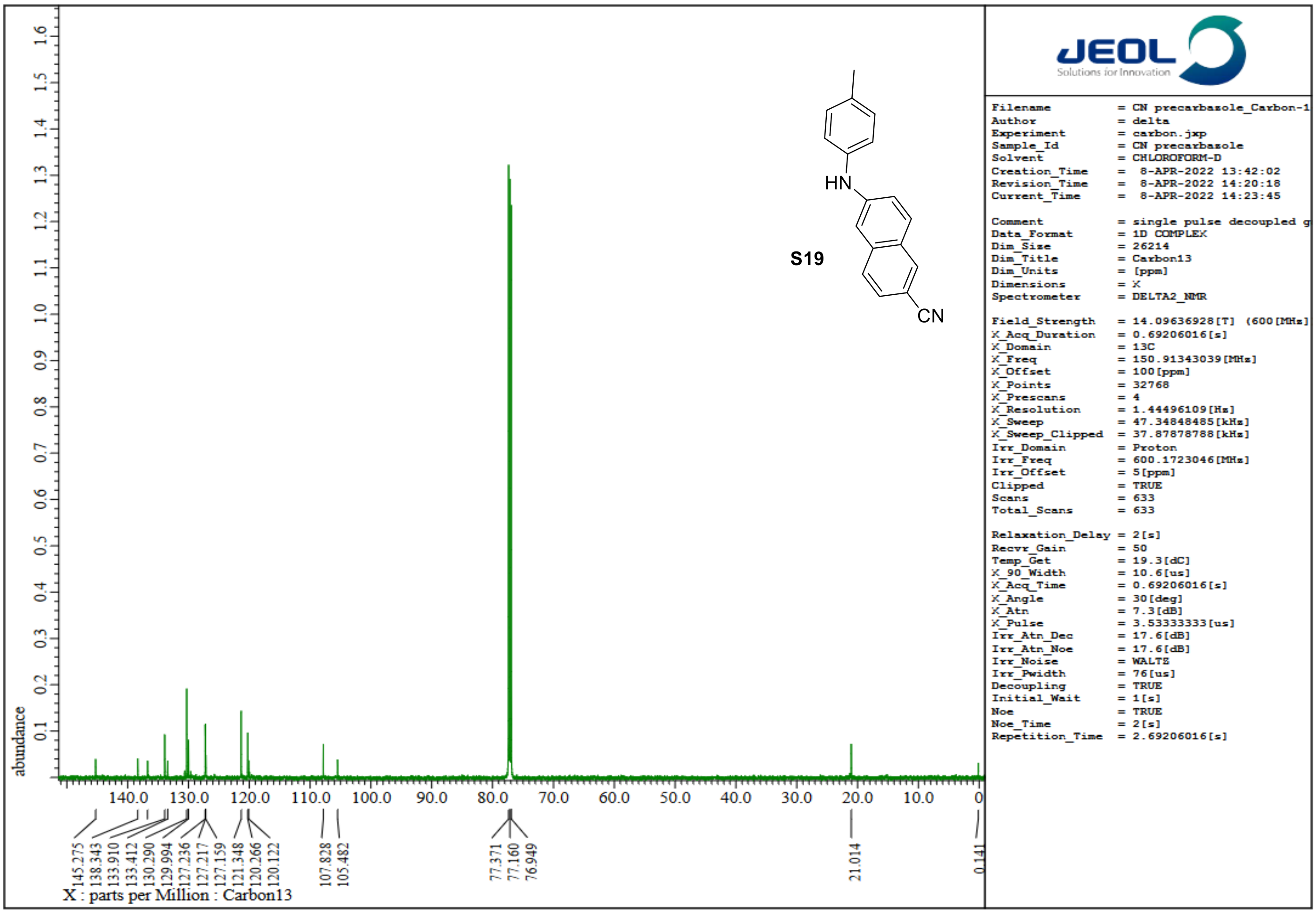

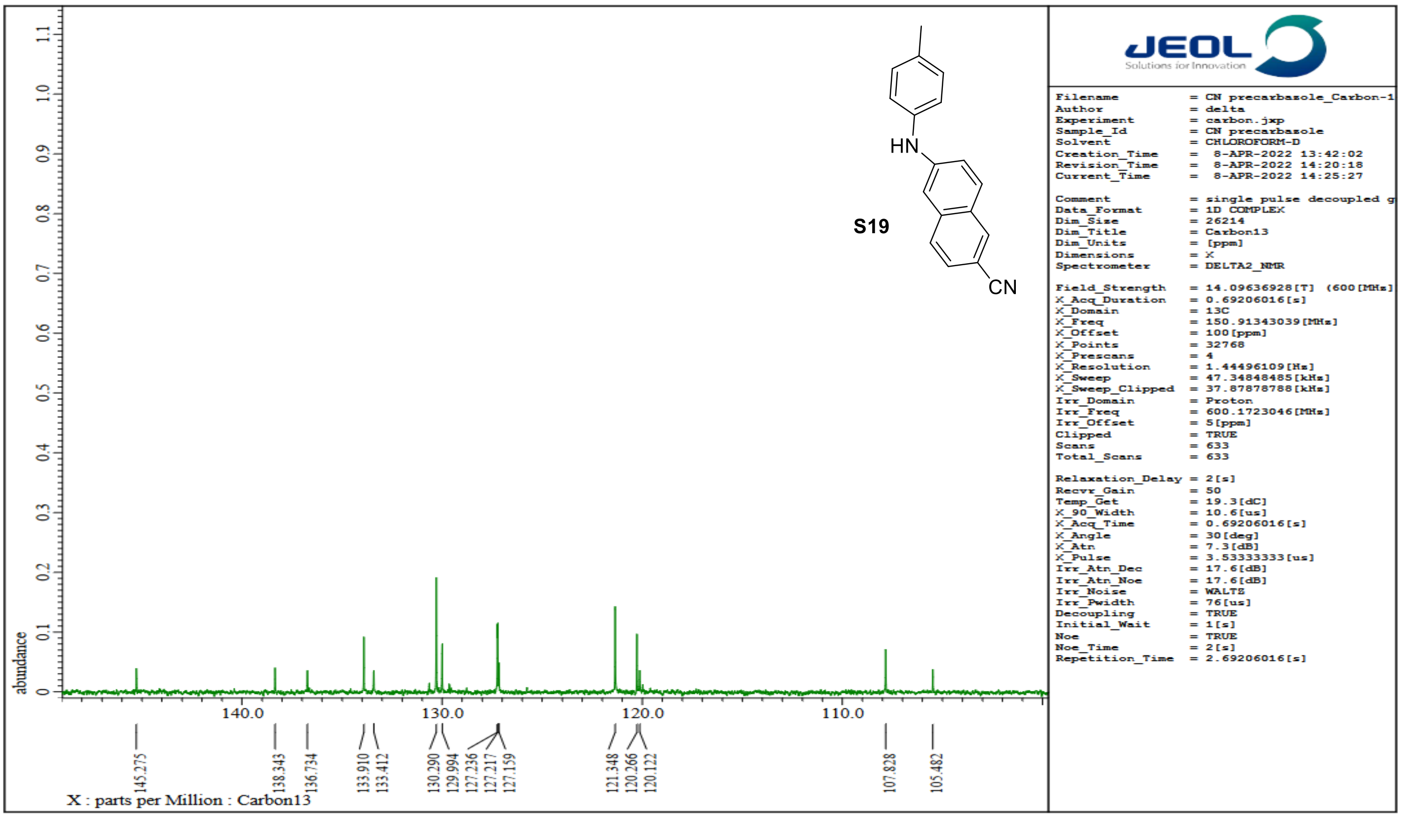

Compound **S19** (^{13}C NMR, 100 MHz, $CDCl_3$).

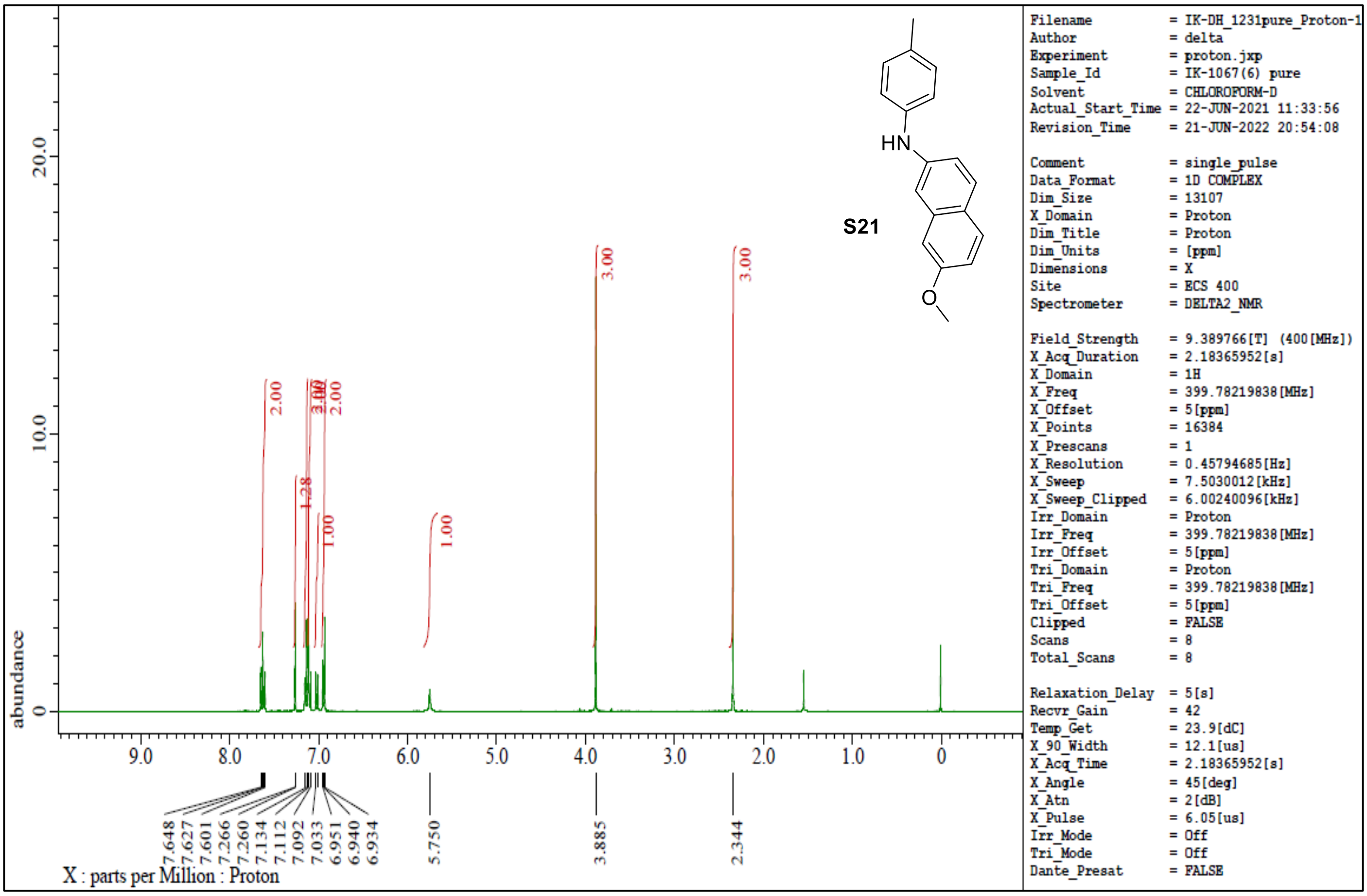

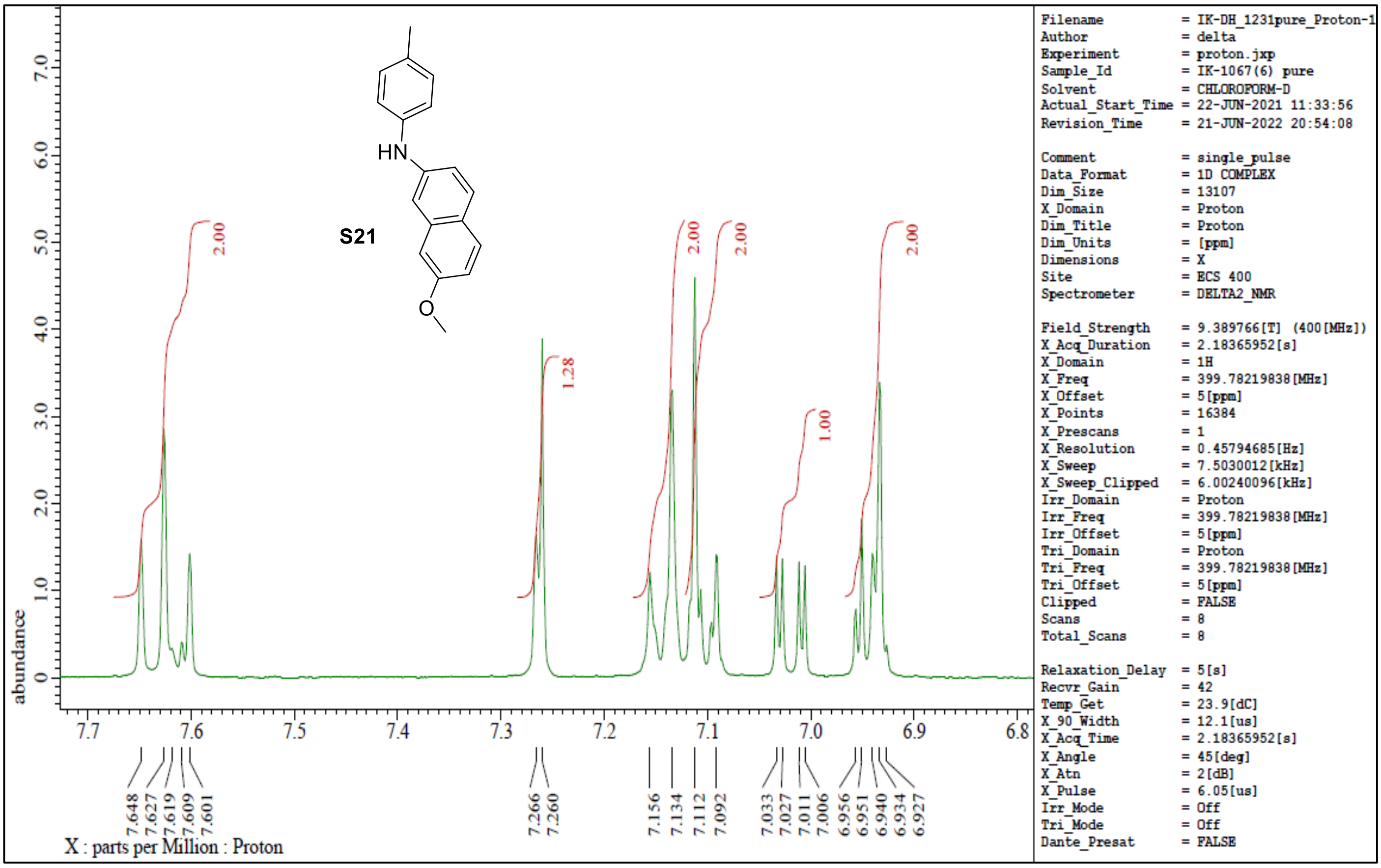

Compound **S21** (^{1}H NMR, 400 MHz, $CDCl_3$).

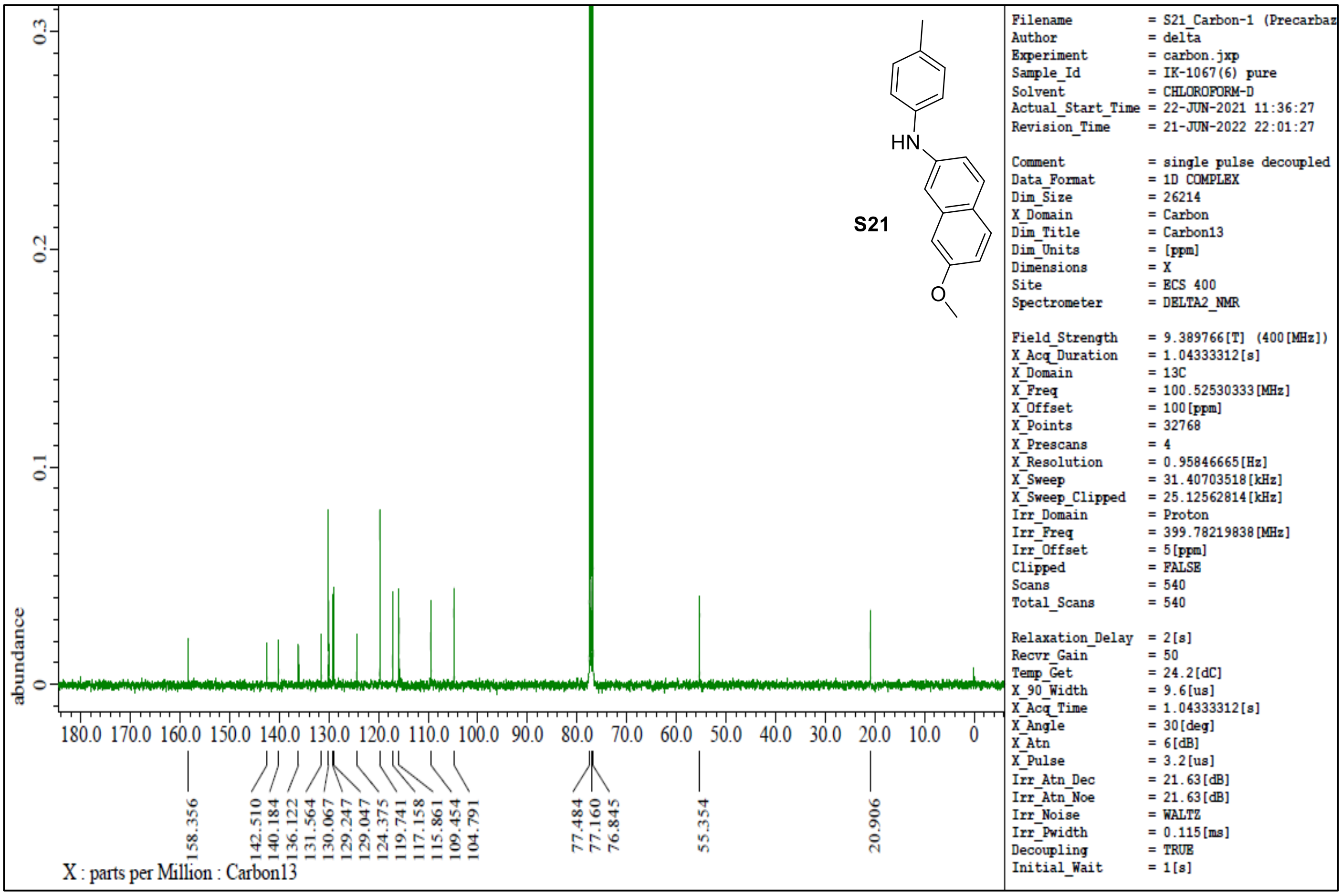

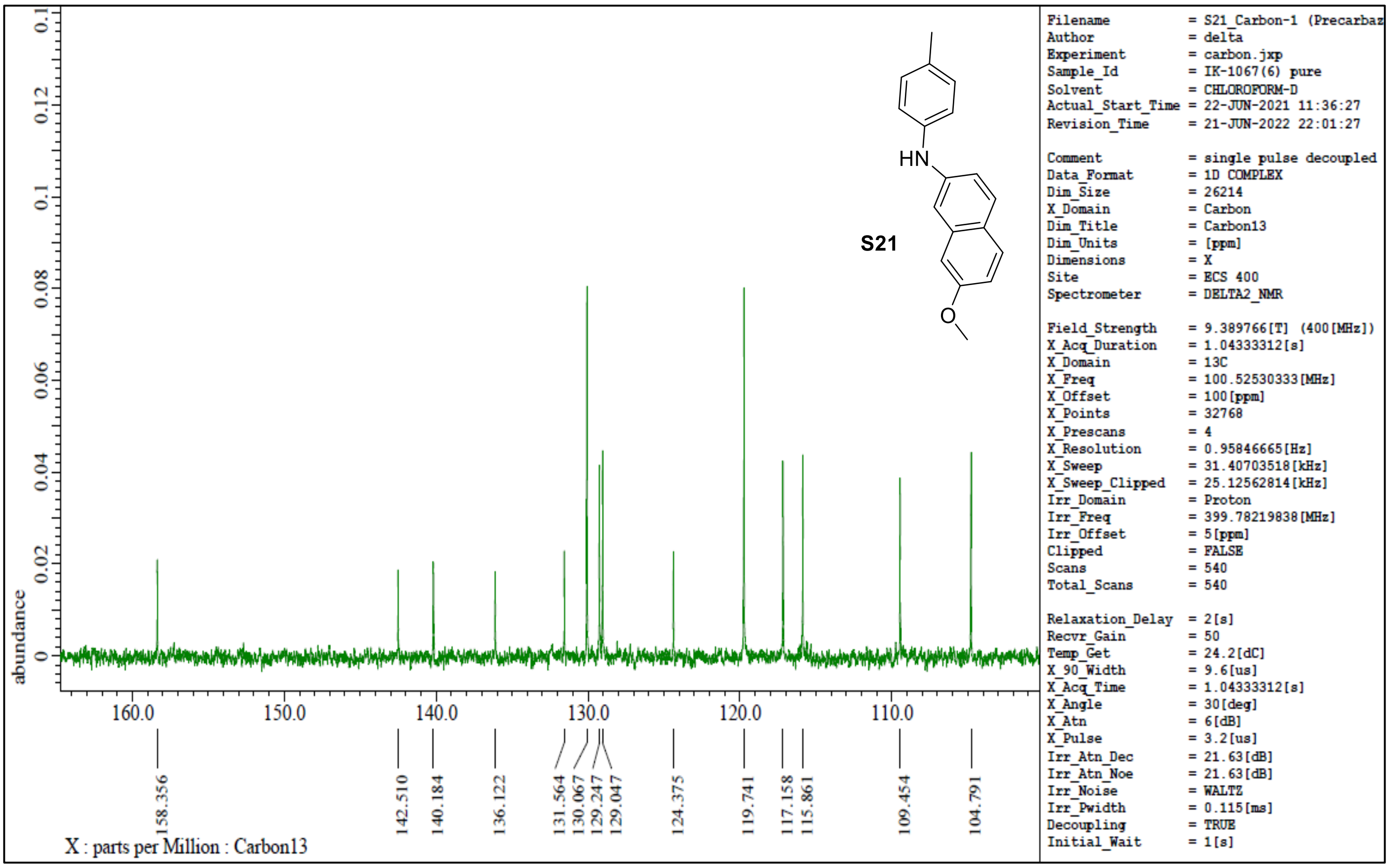

Compound **S21** (^{13}C NMR, 100 MHz, $CDCl_3$).

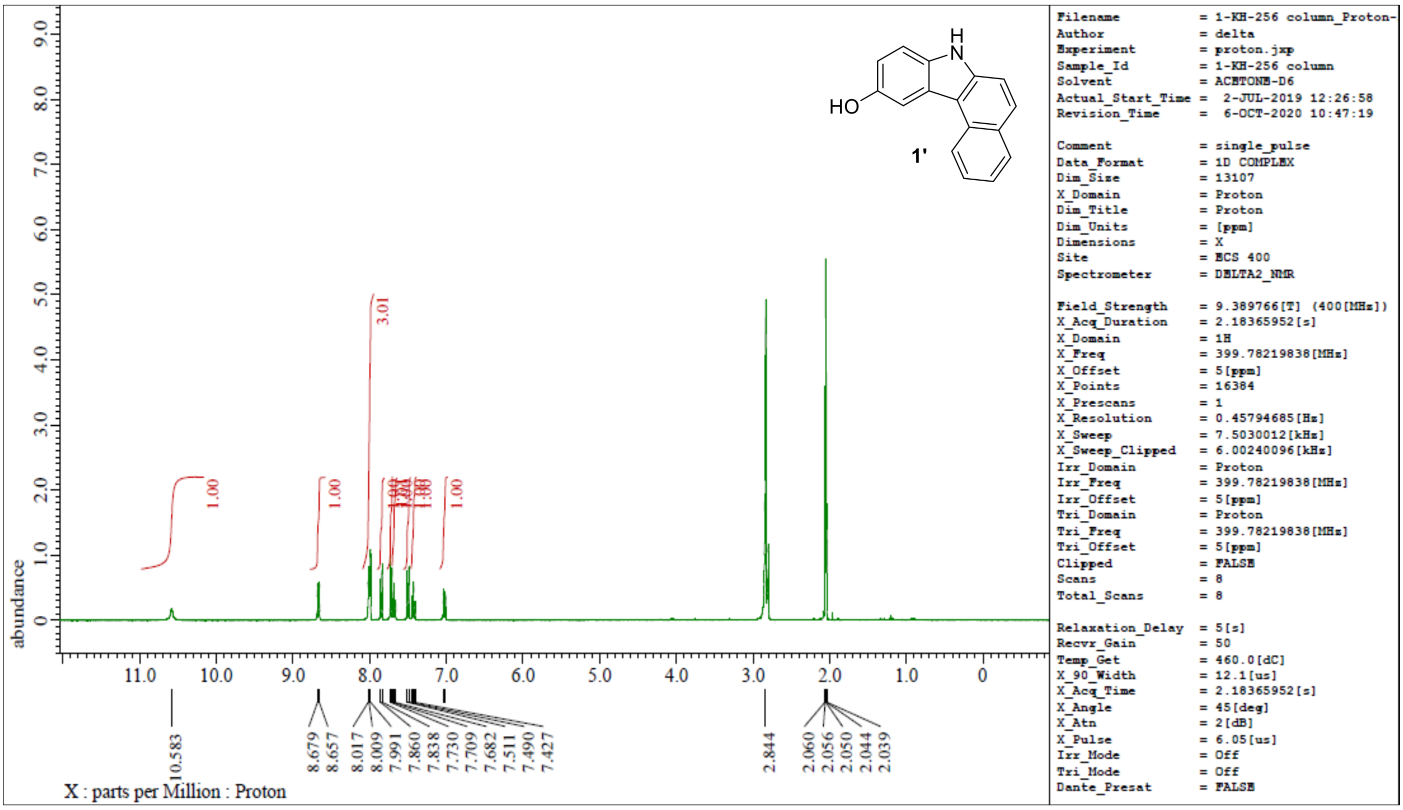

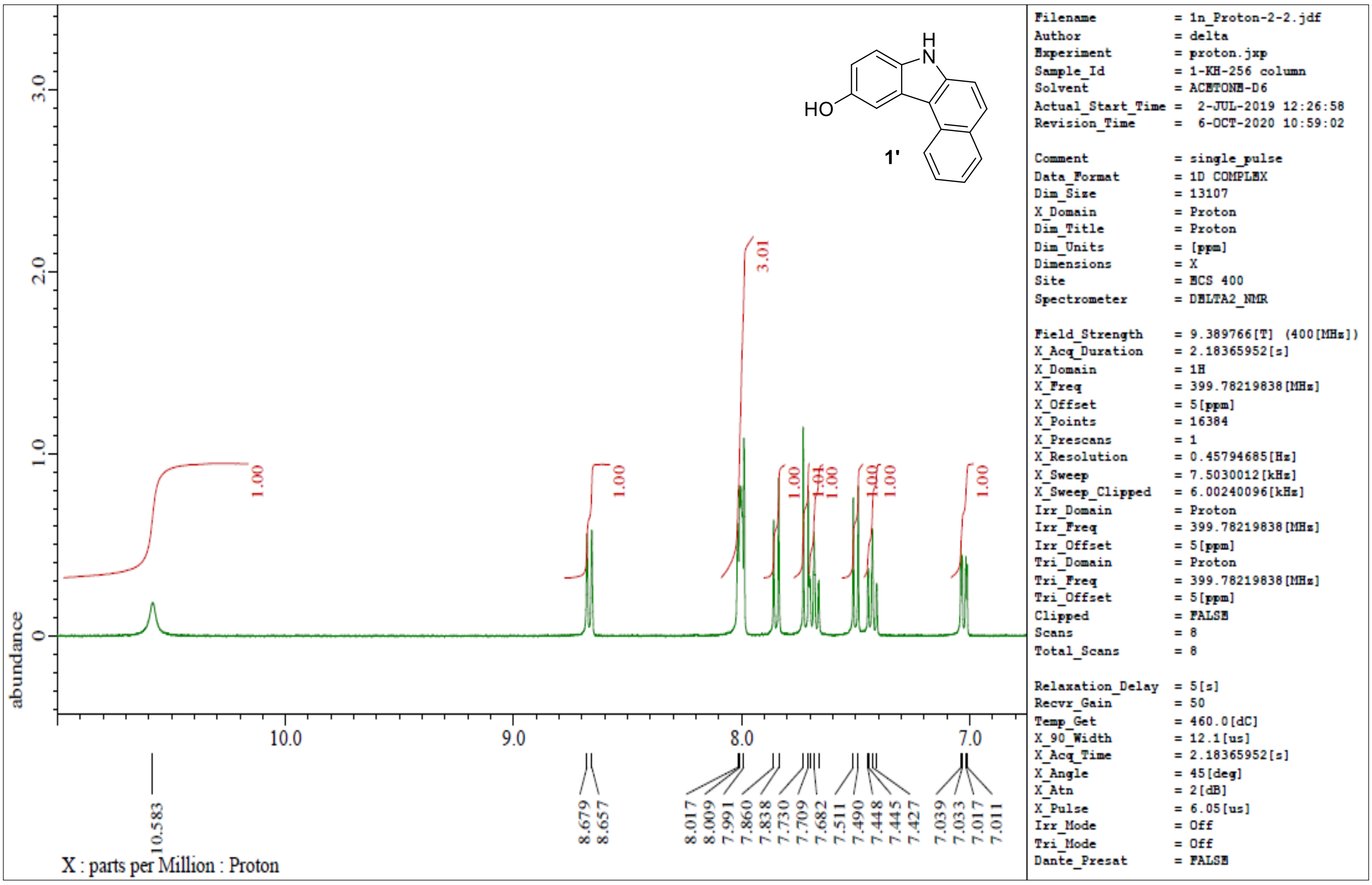

Compound **1’** (^{1}H NMR, 400 MHz, $(CD_3)_2CO$).

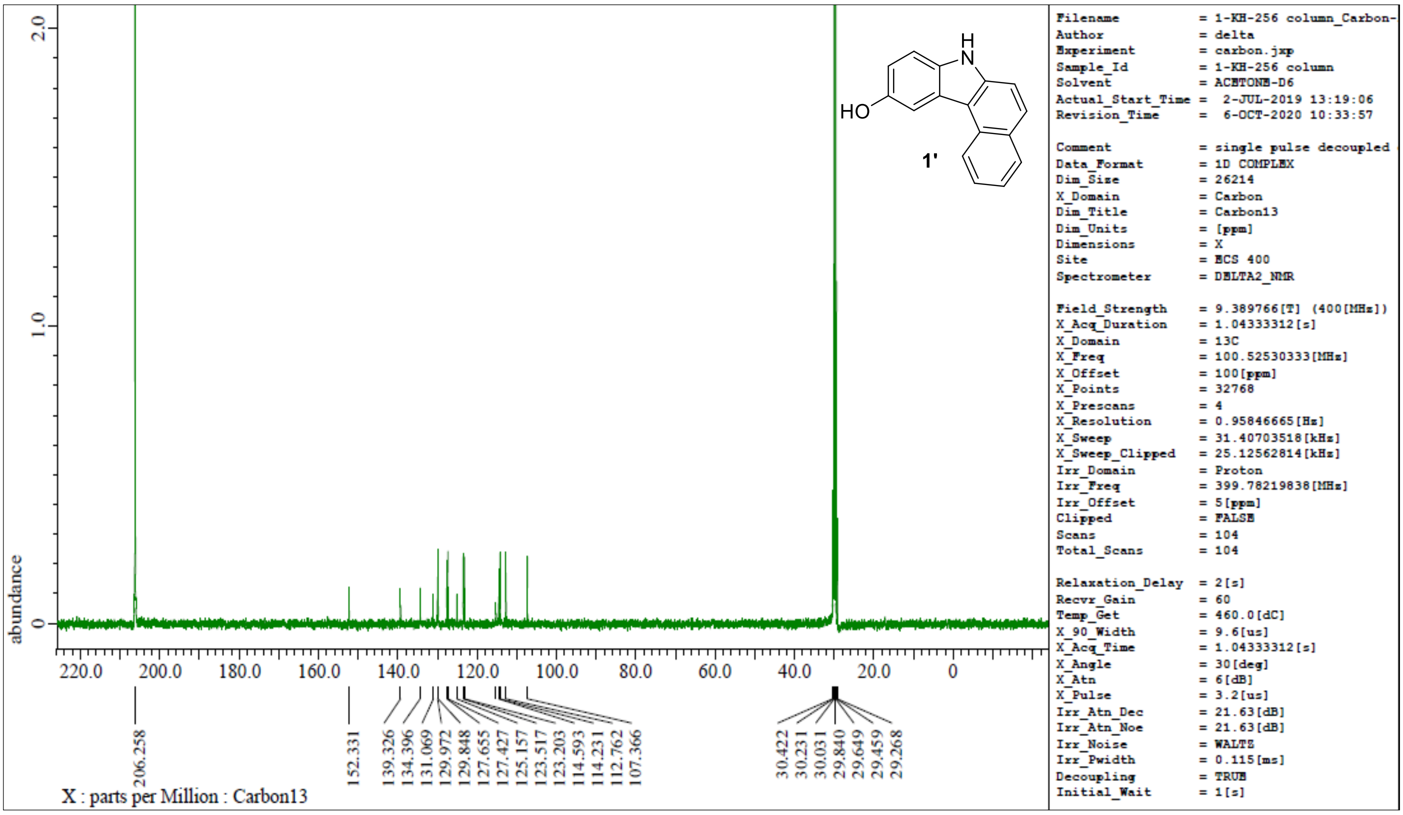

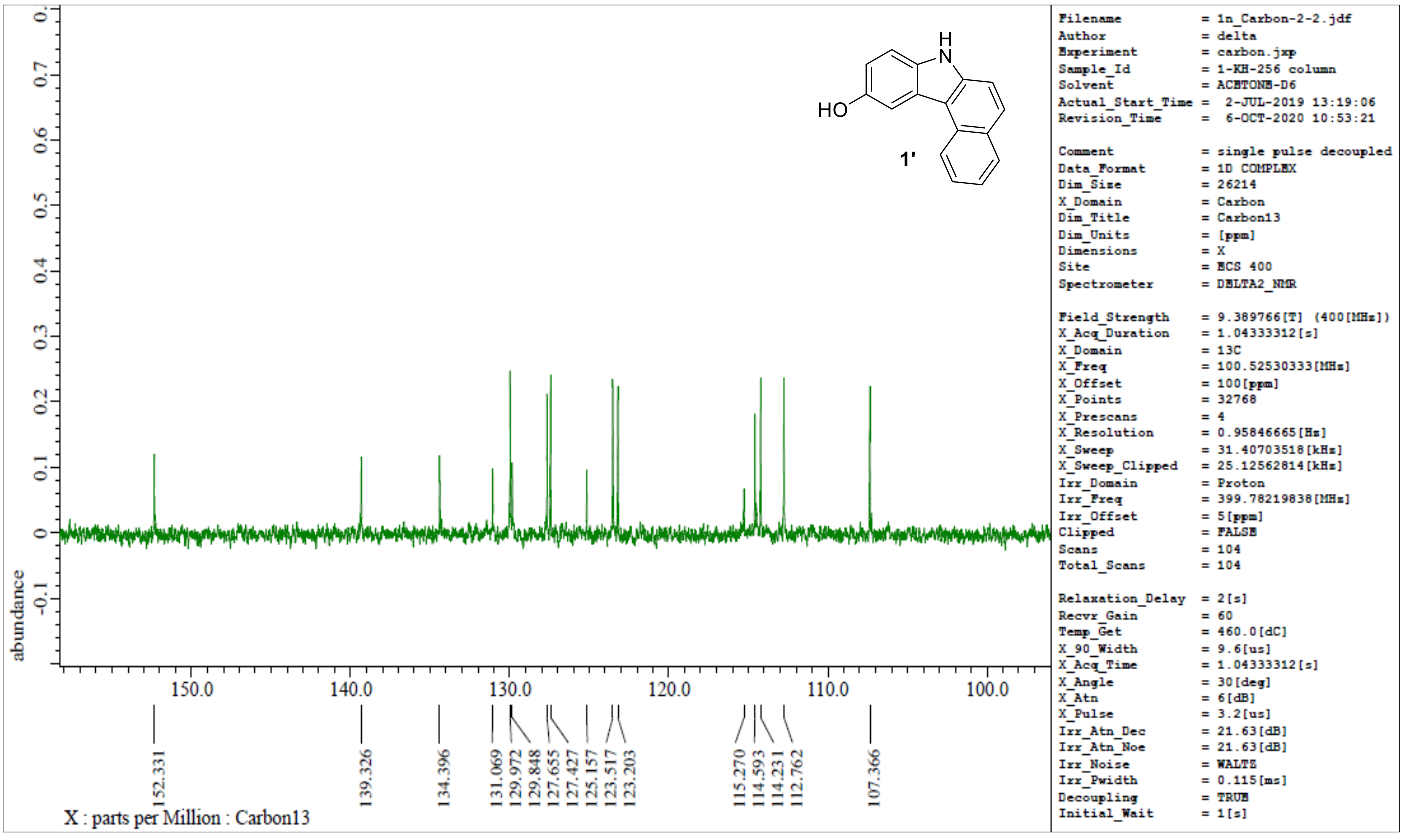

Compound **1’** (^{13}C NMR, 100 MHz, $(CD_3)_2CO$).

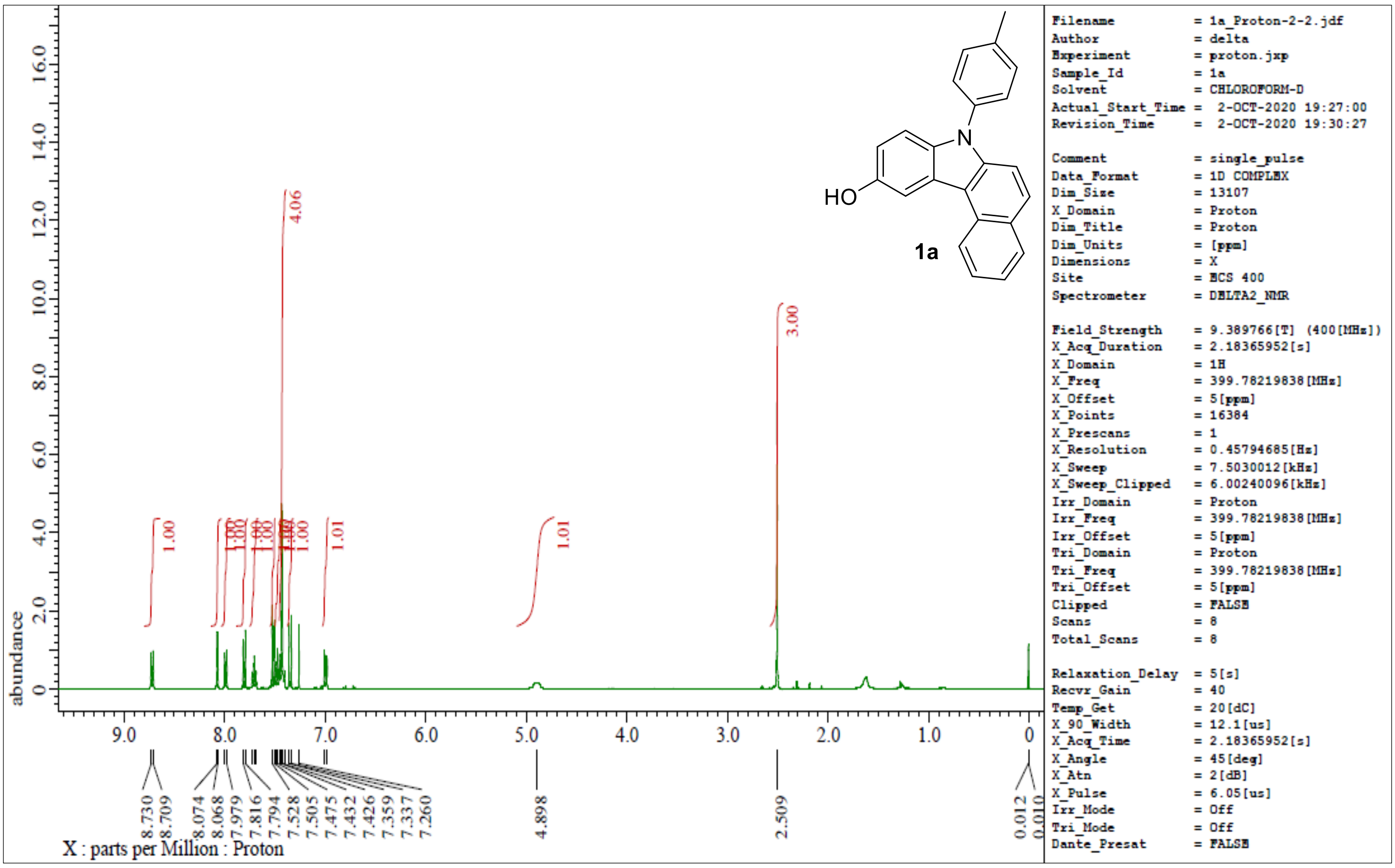

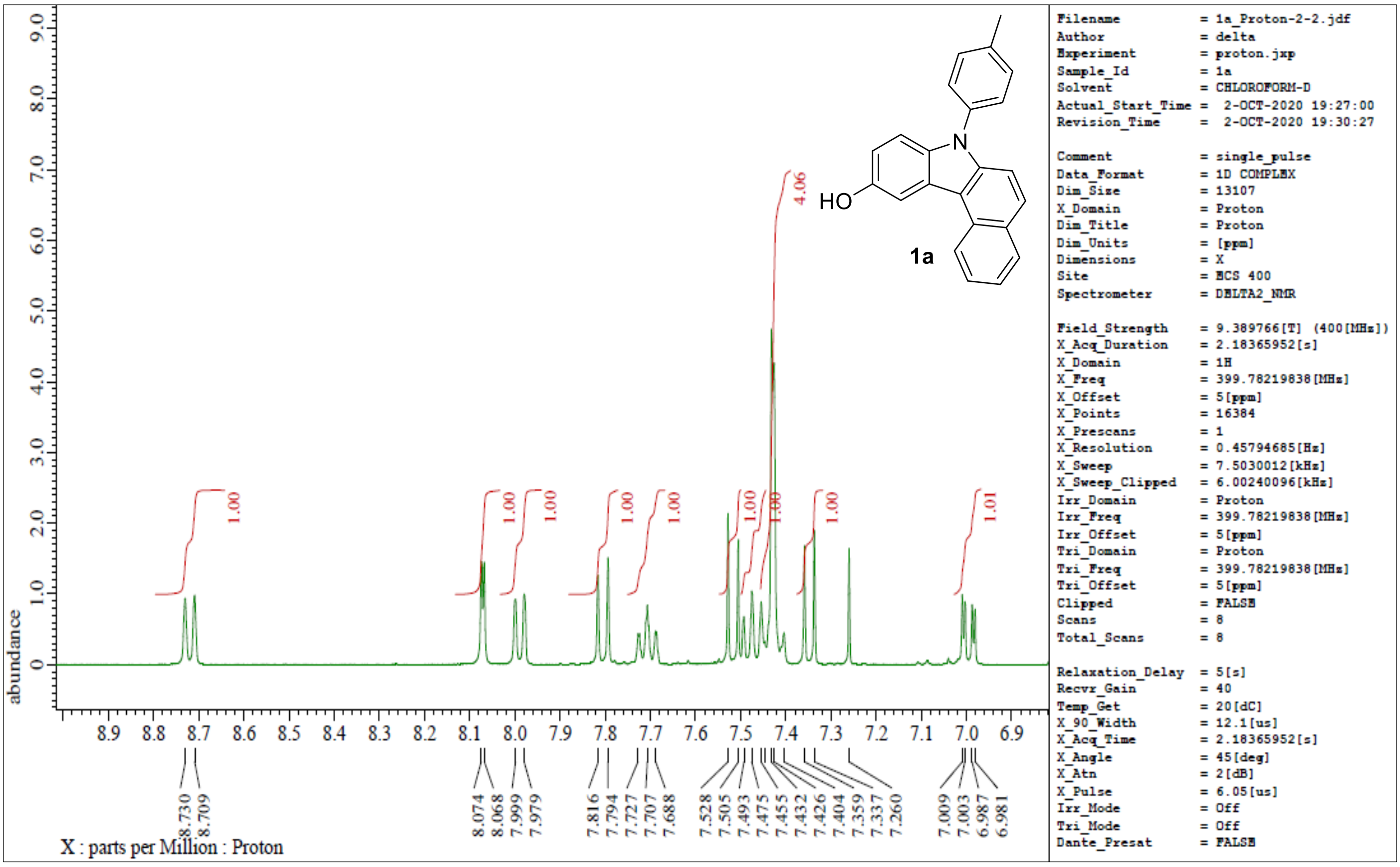

Compound **1a** ([1] H NMR, 400 MHz, $CDCl_3$).

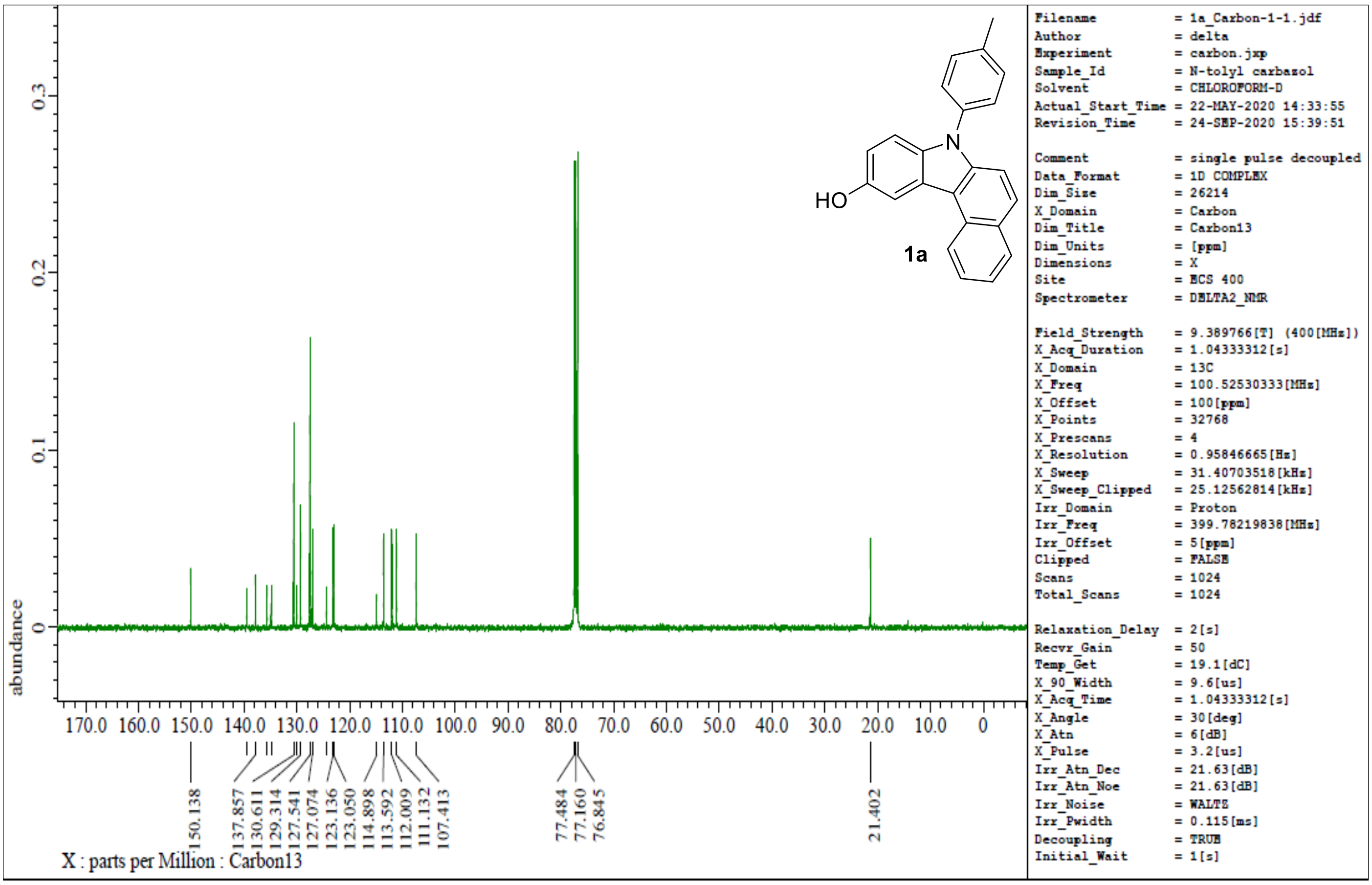

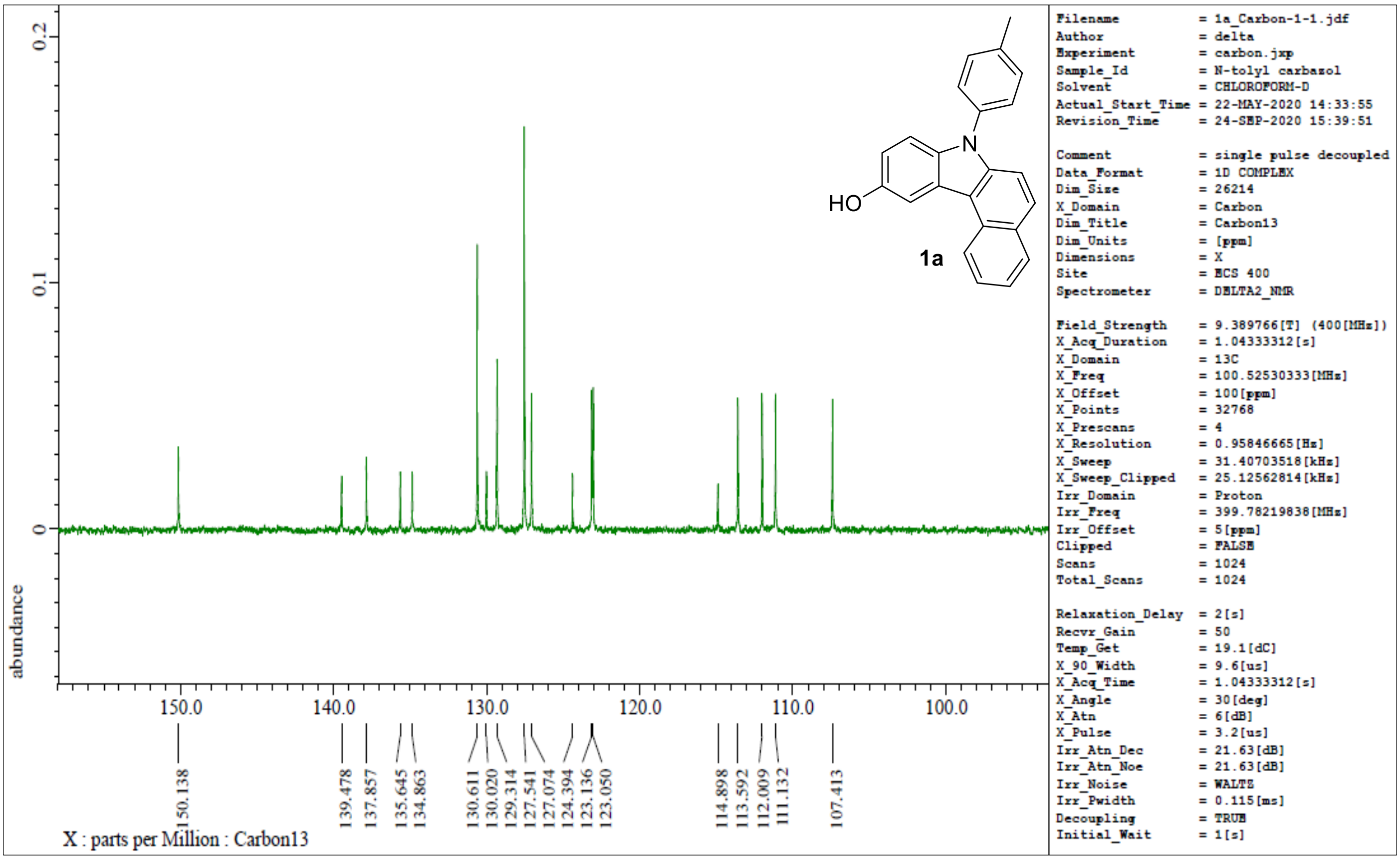

Compound **1a** (^{13}C NMR, 100 MHz, $CDCl_3$).

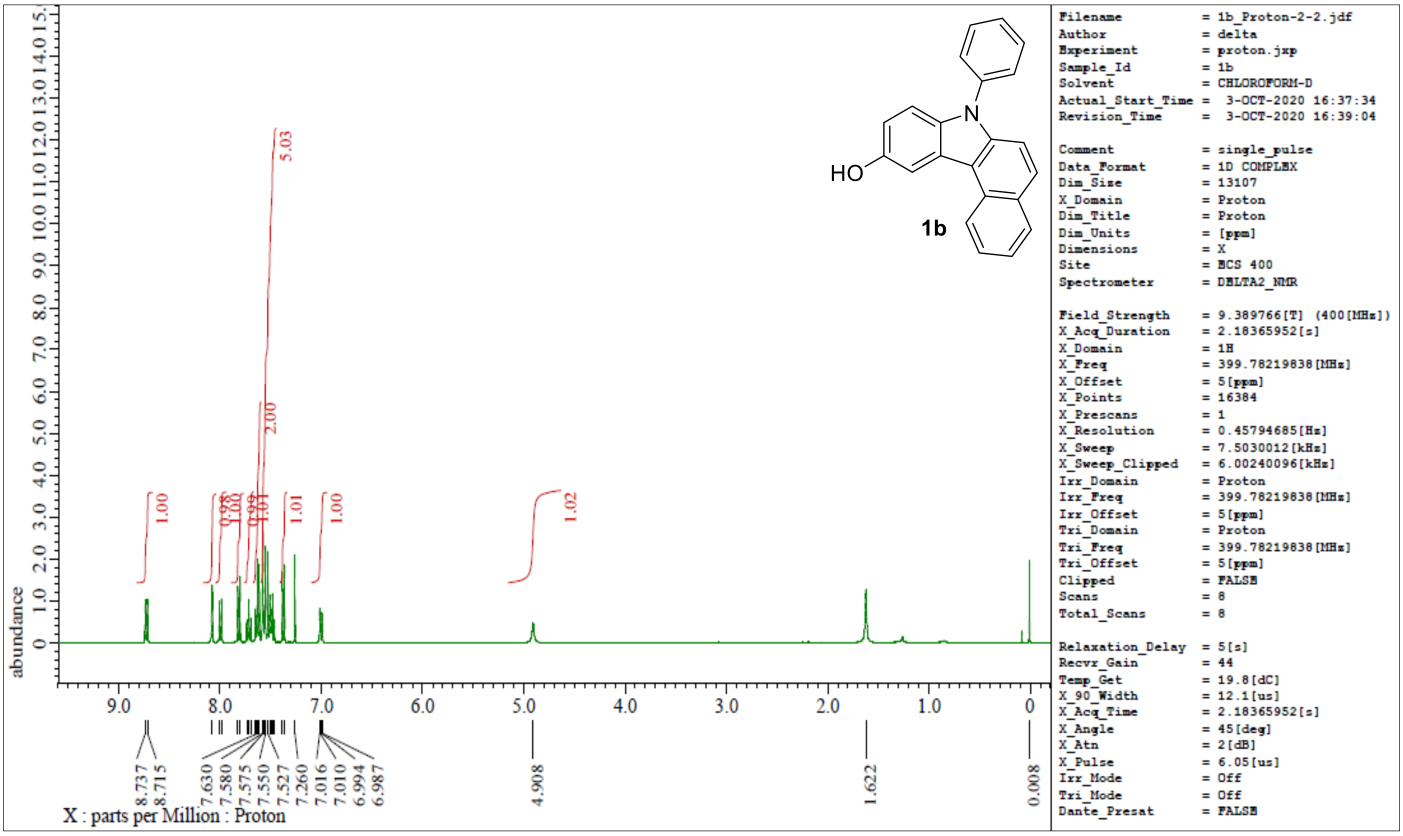

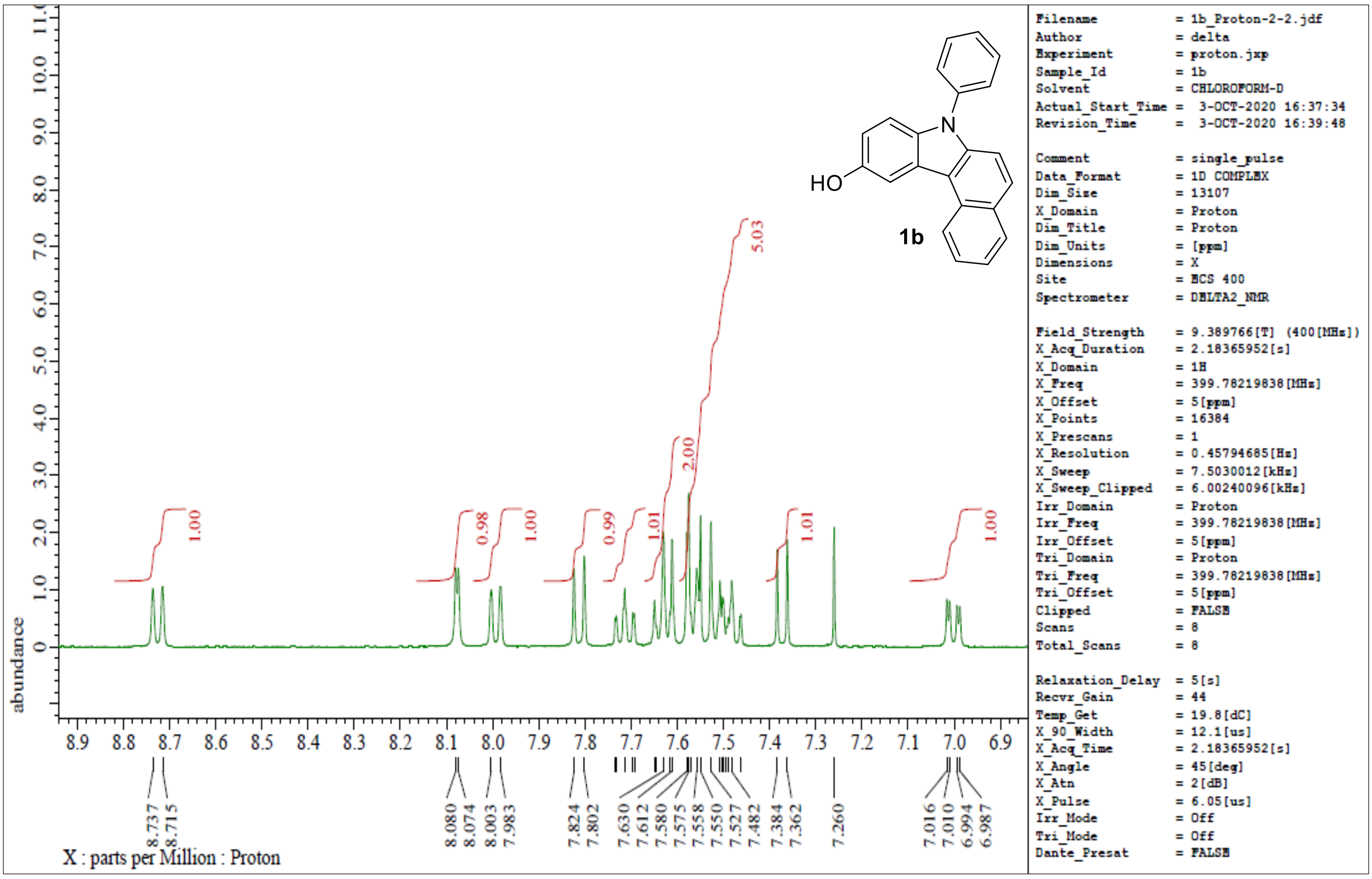

Compound **1b** (^{1}H NMR, 400 MHz, $CDCl_3$).

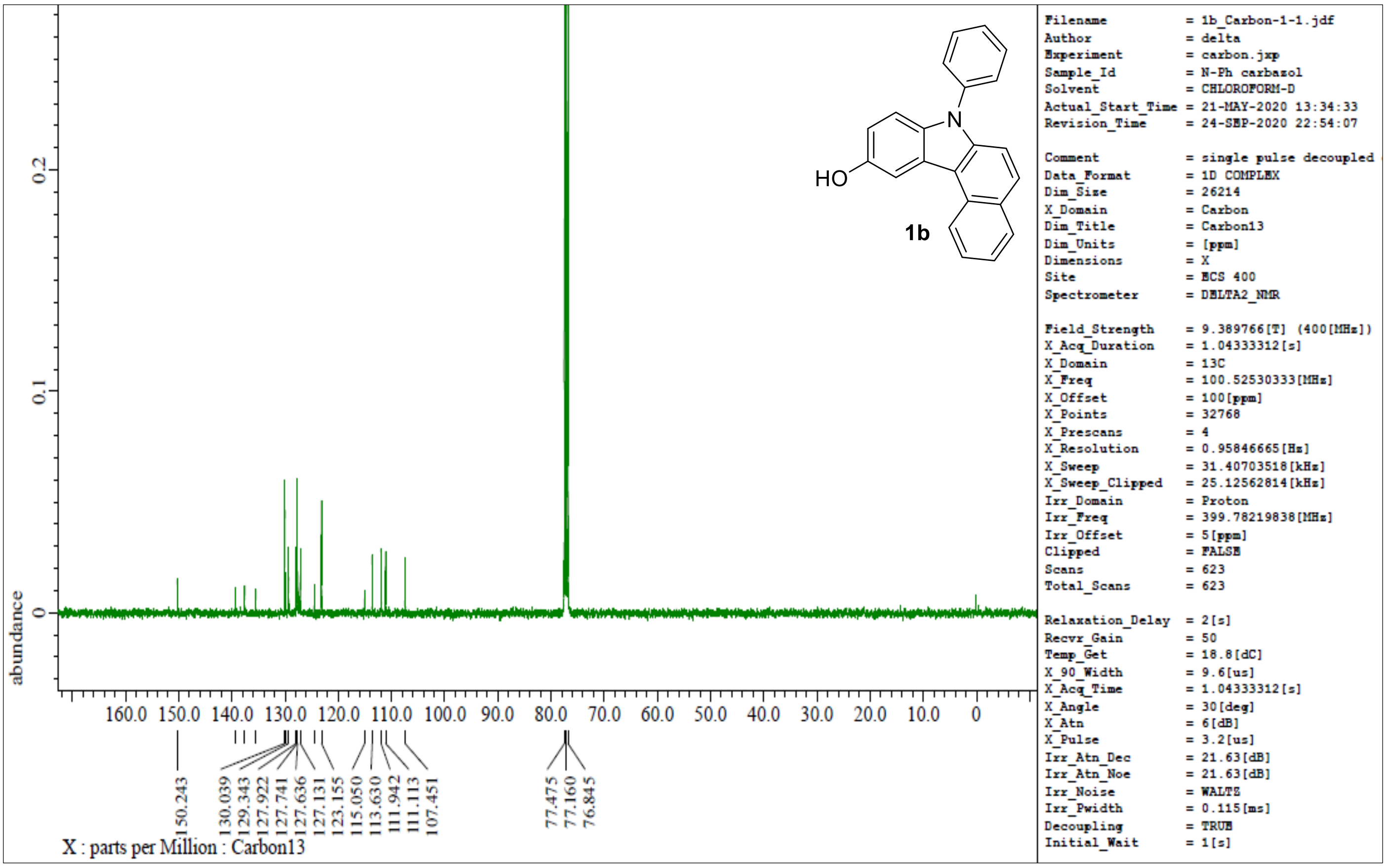

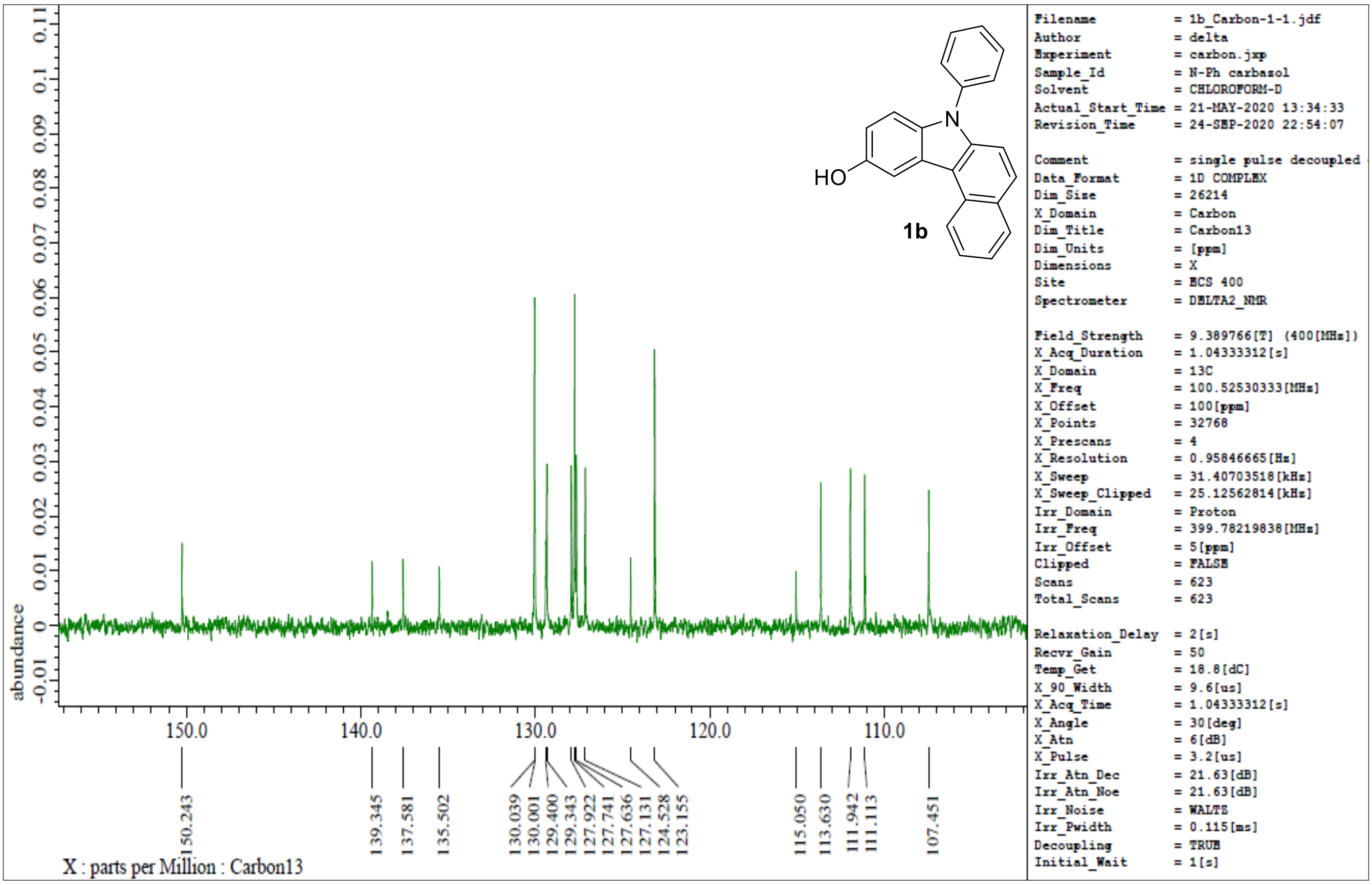

Compound **1b** (^{13}C NMR, 100 MHz, $CDCl_3$).

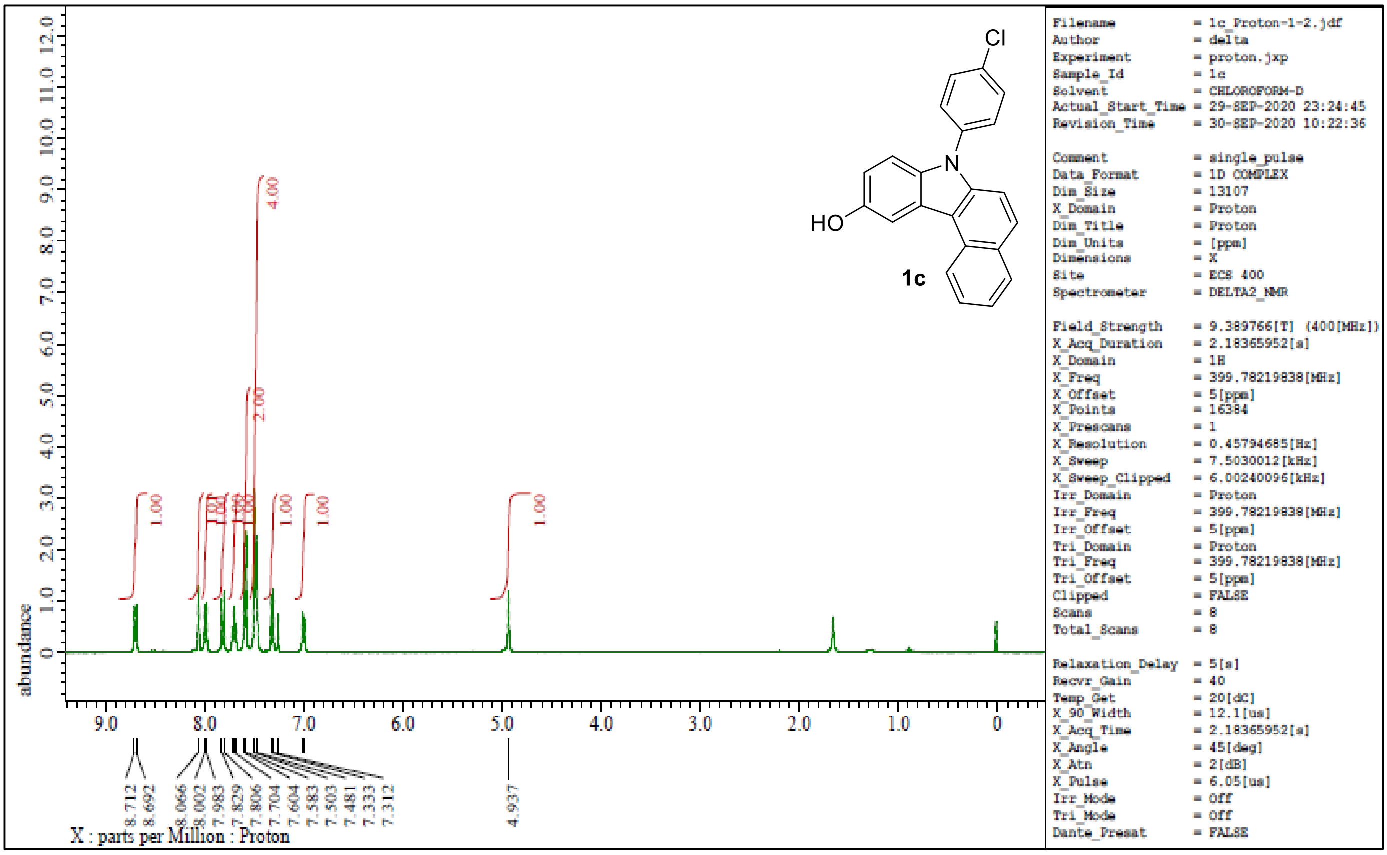

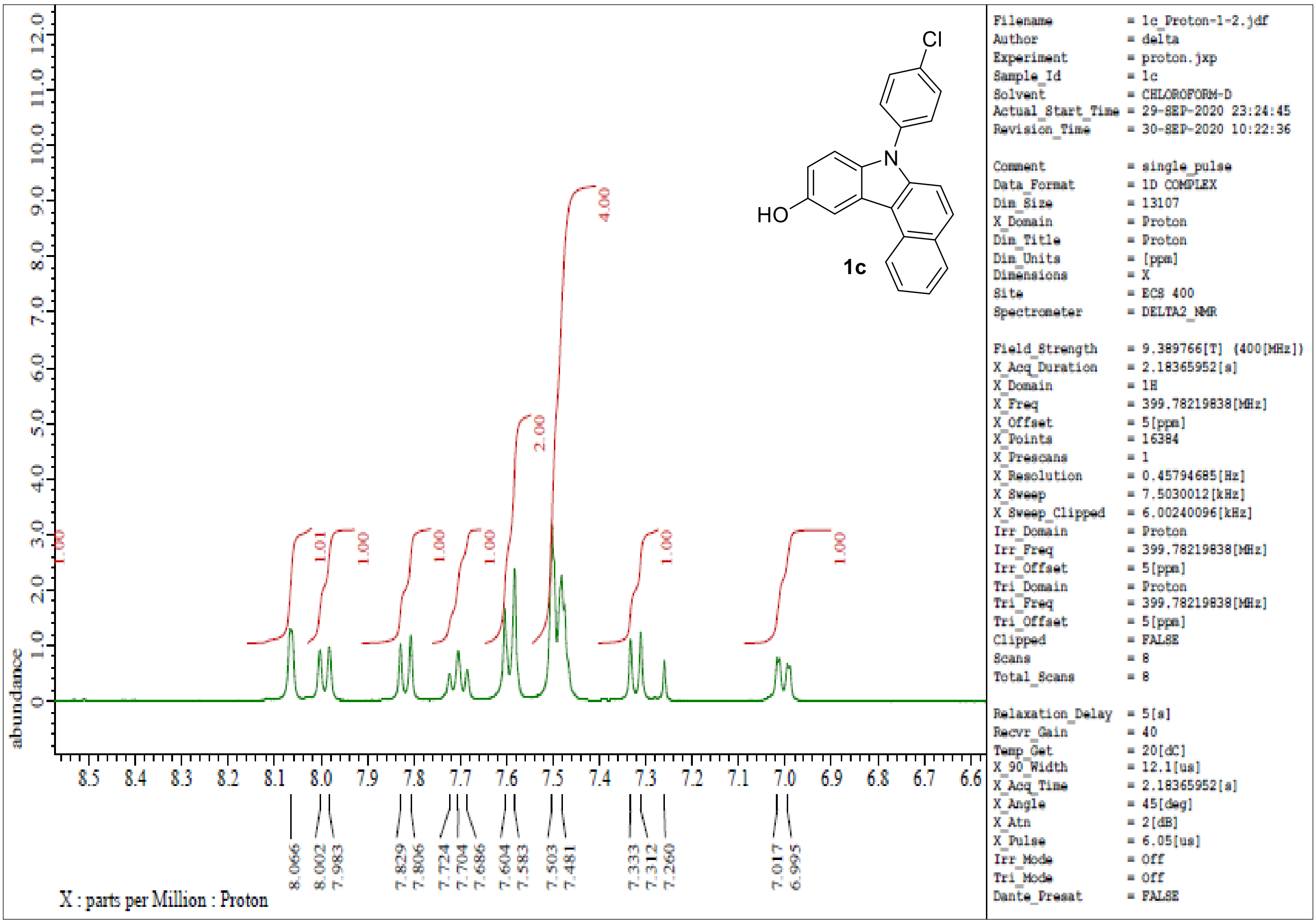

Compound **1c** ([1] H NMR, 400 MHz, $CDCl_3$).

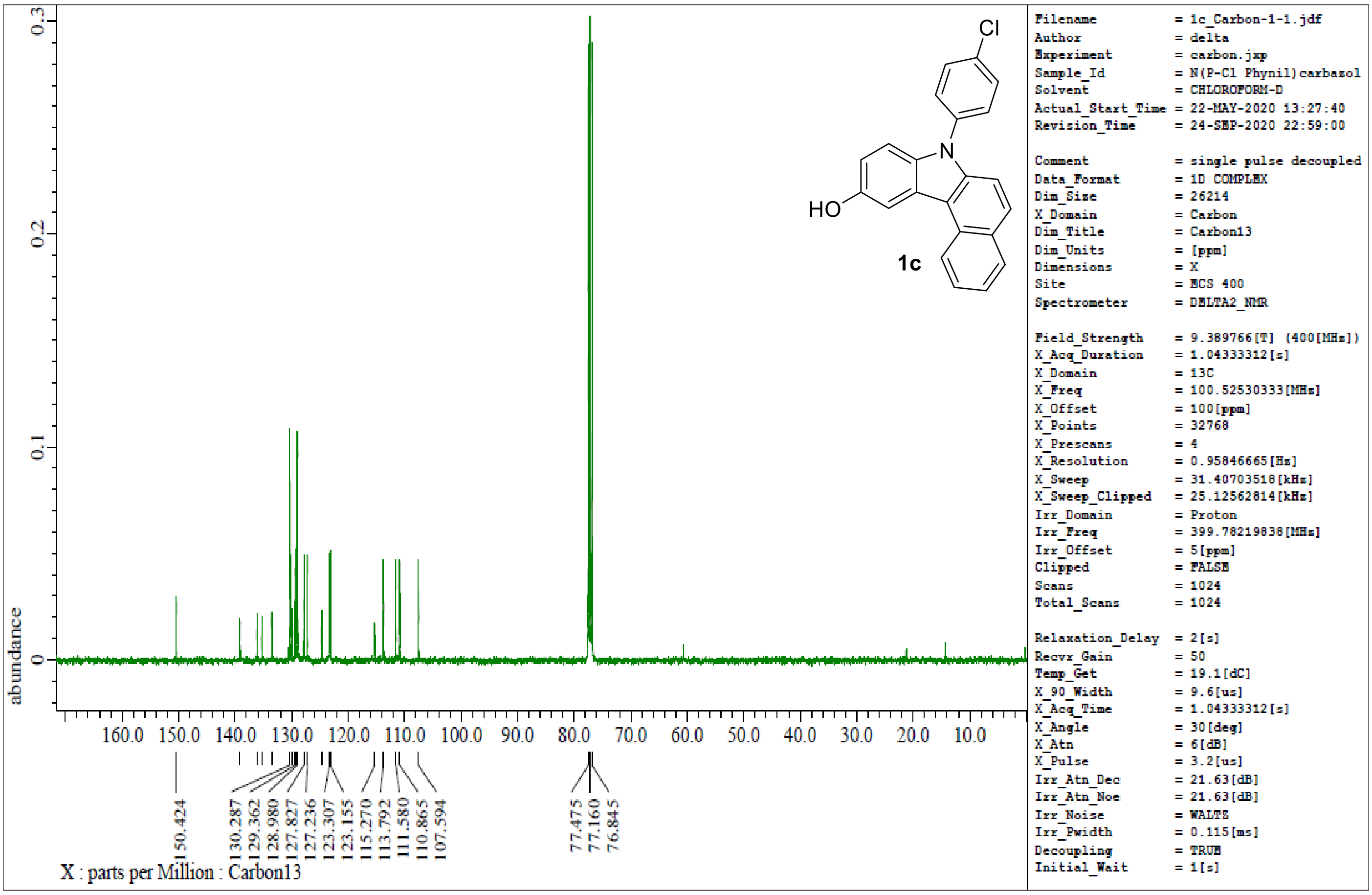

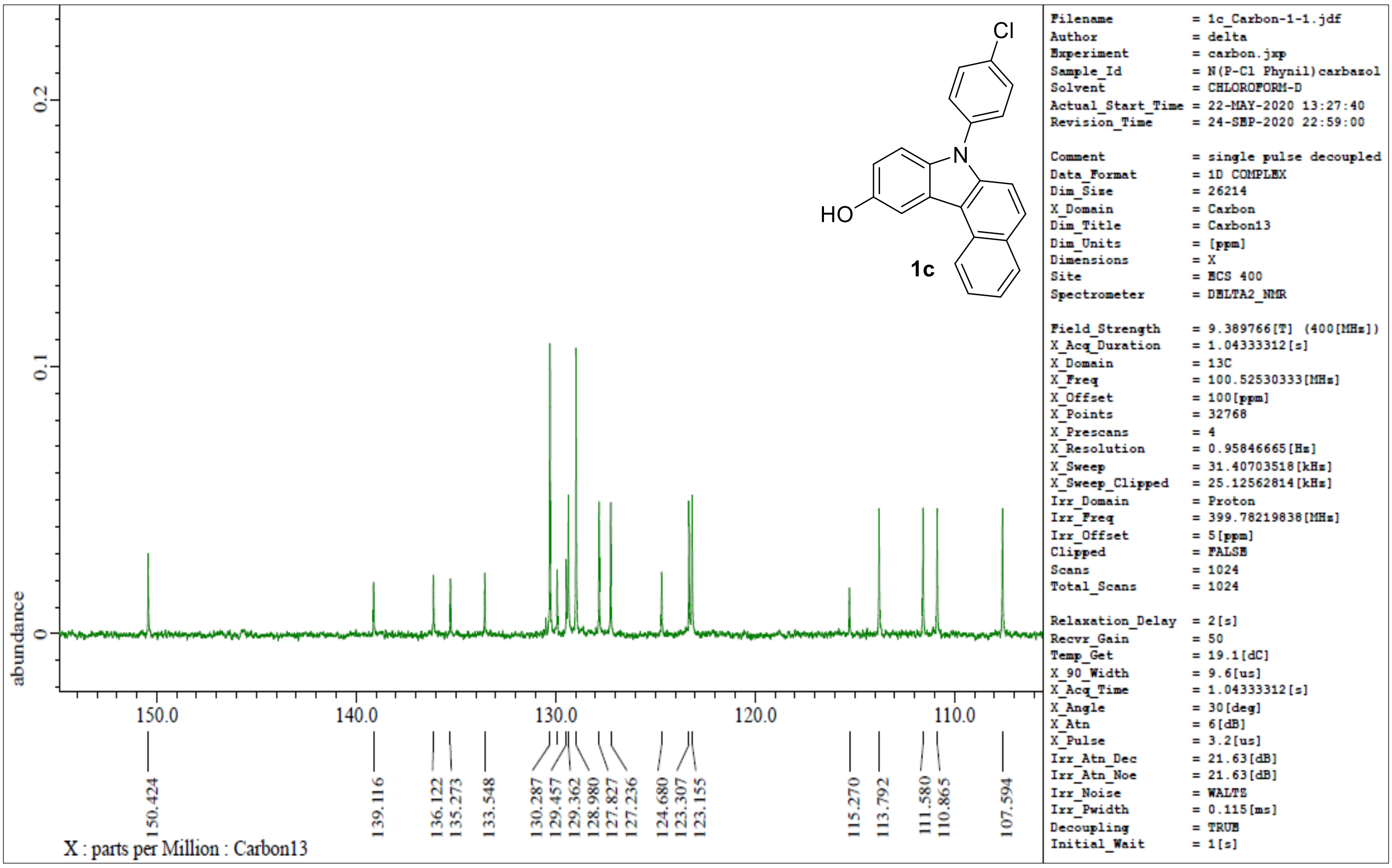

Compound **1c** (^{13}C NMR, 100 MHz, $CDCl_3$).

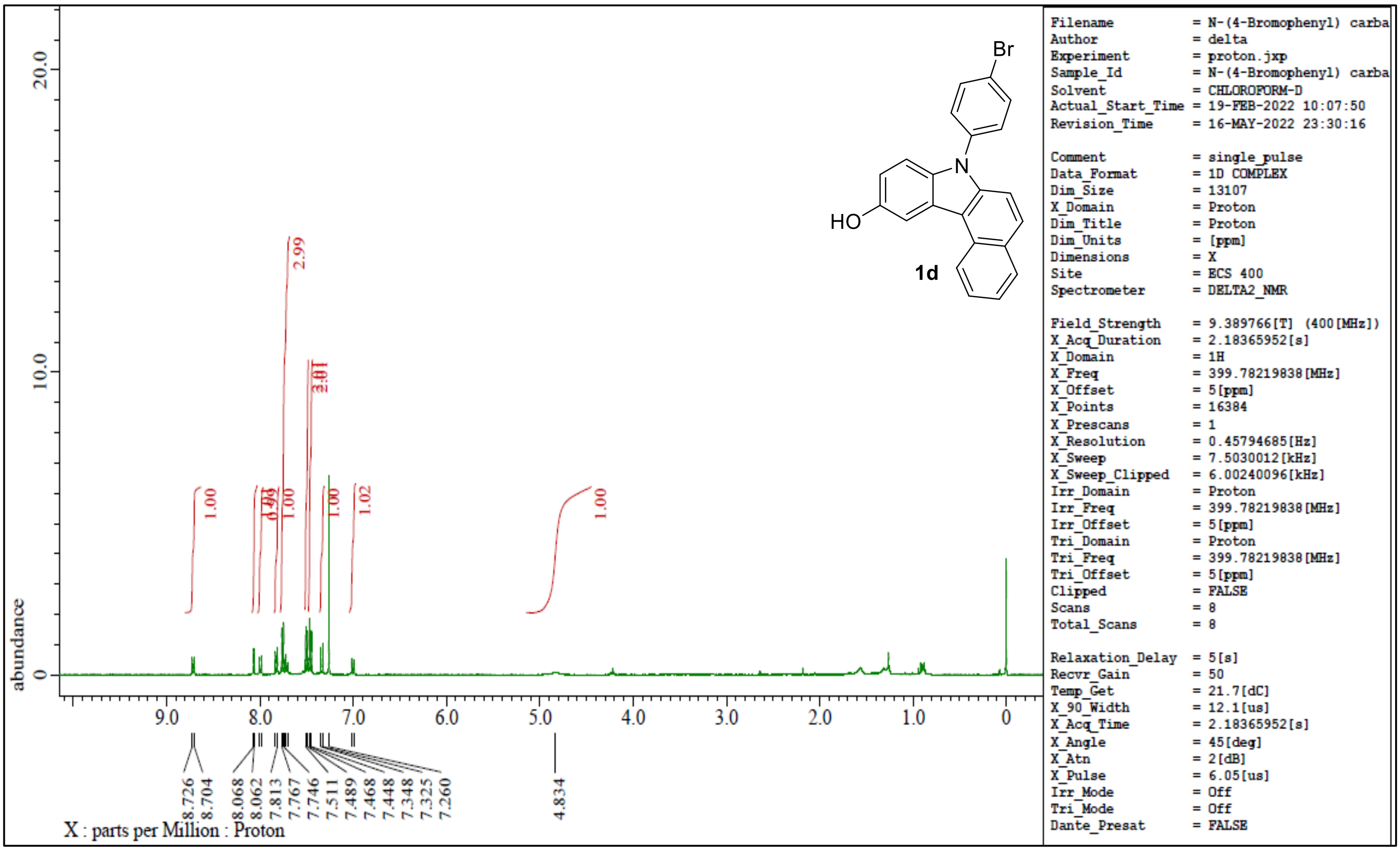

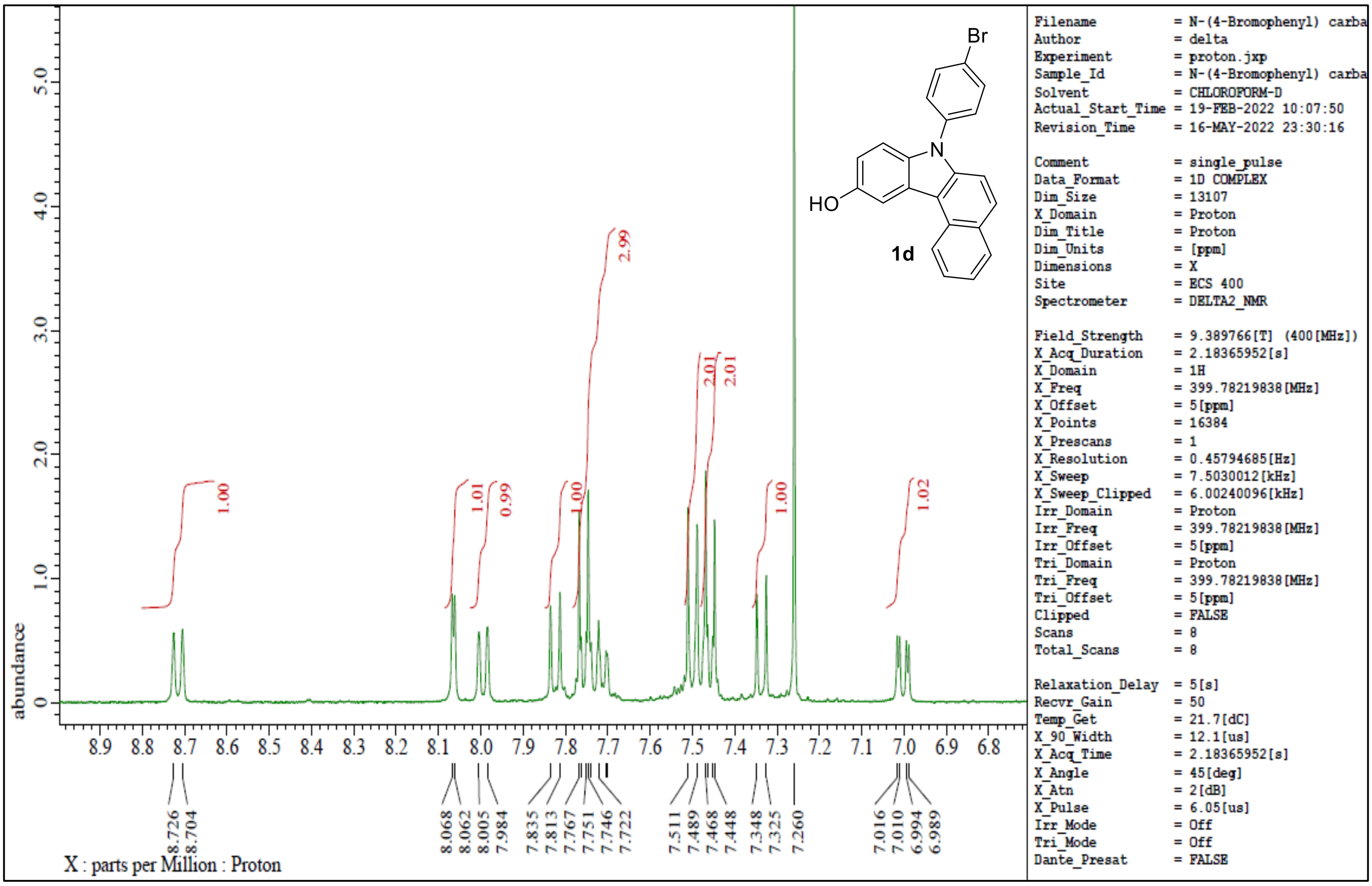

Compound **1d** (^{1}H NMR, 100 MHz, $CDCl_3$).

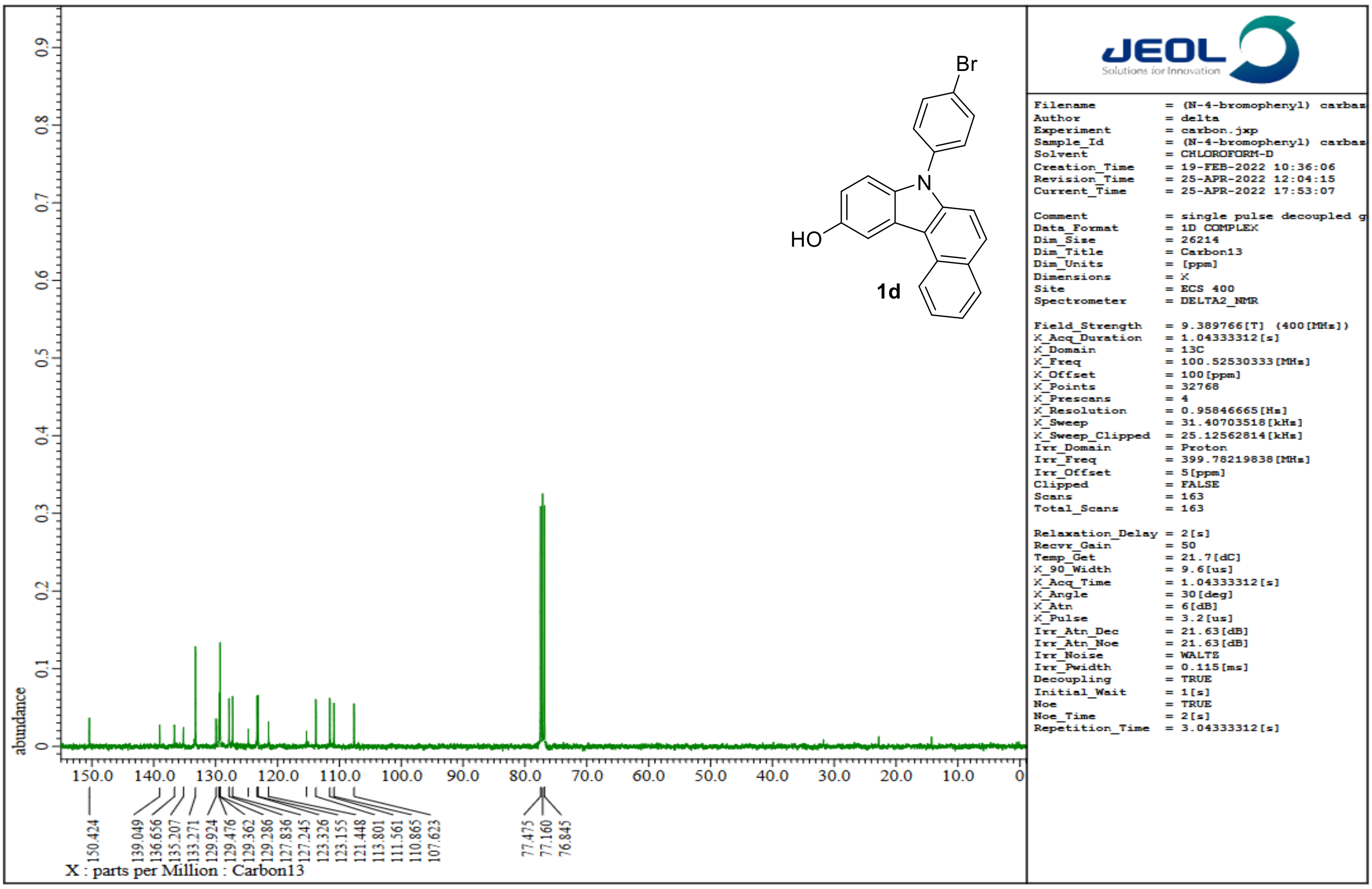

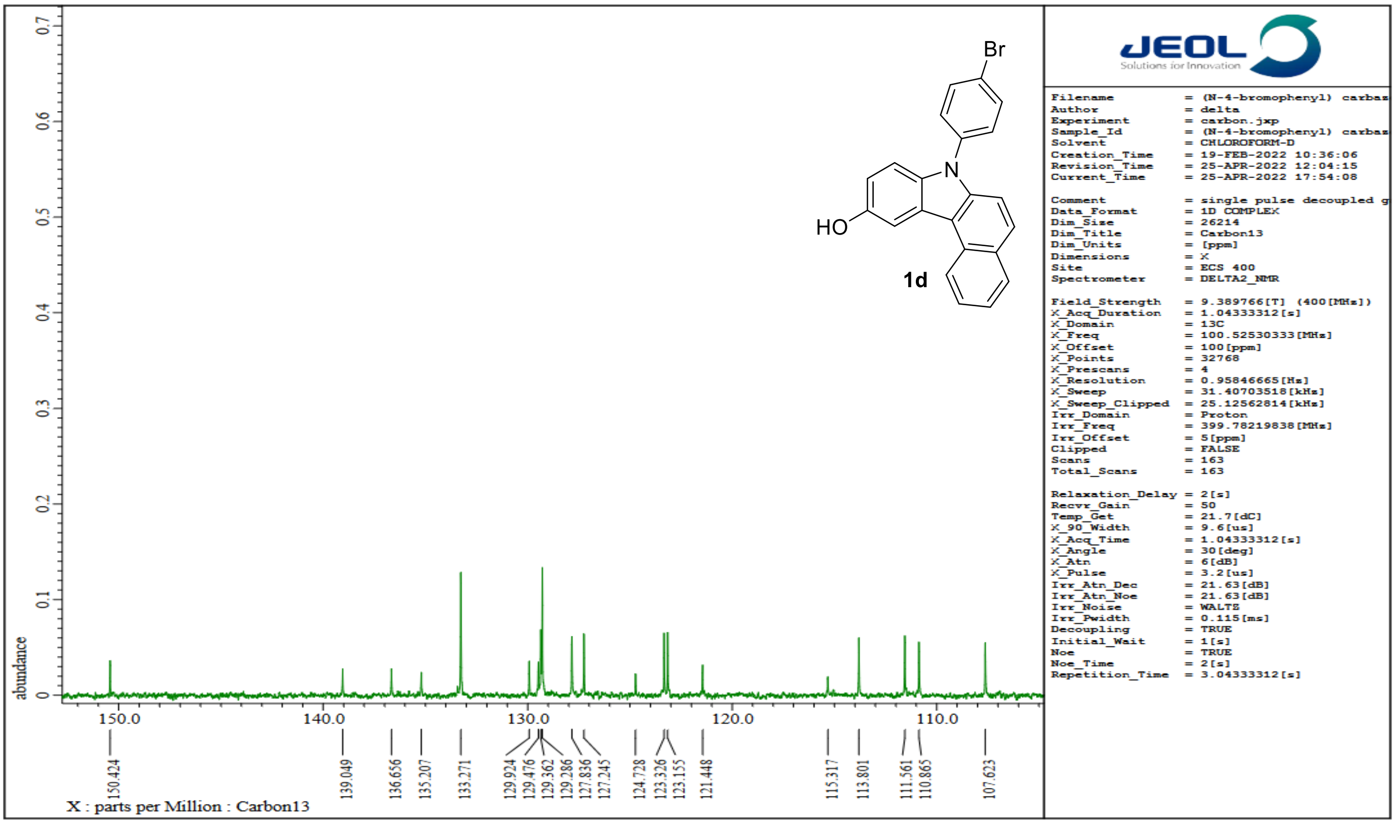

Compound **1d** (^{1}3C NMR, 100 MHz, $CDCl_3$).

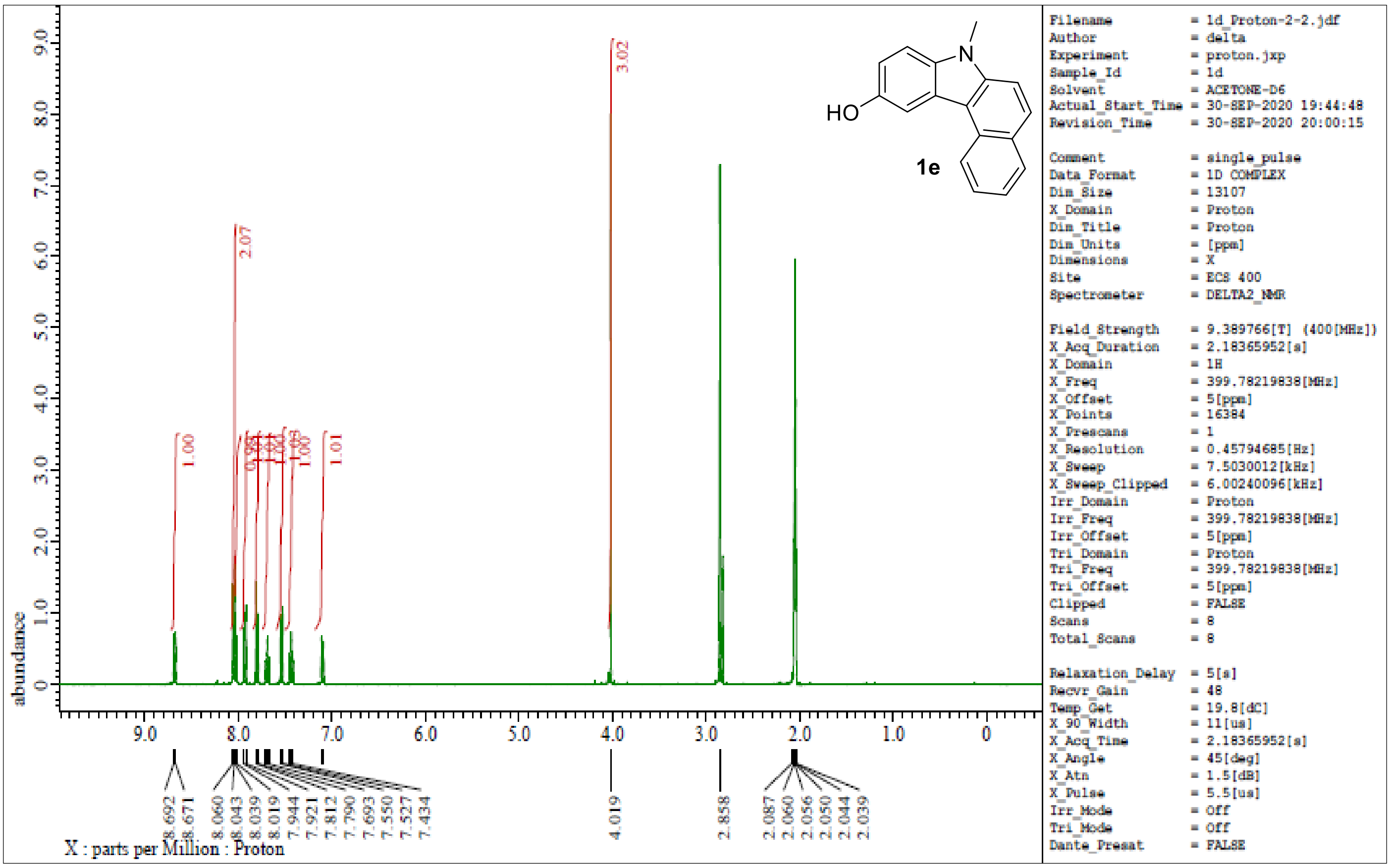

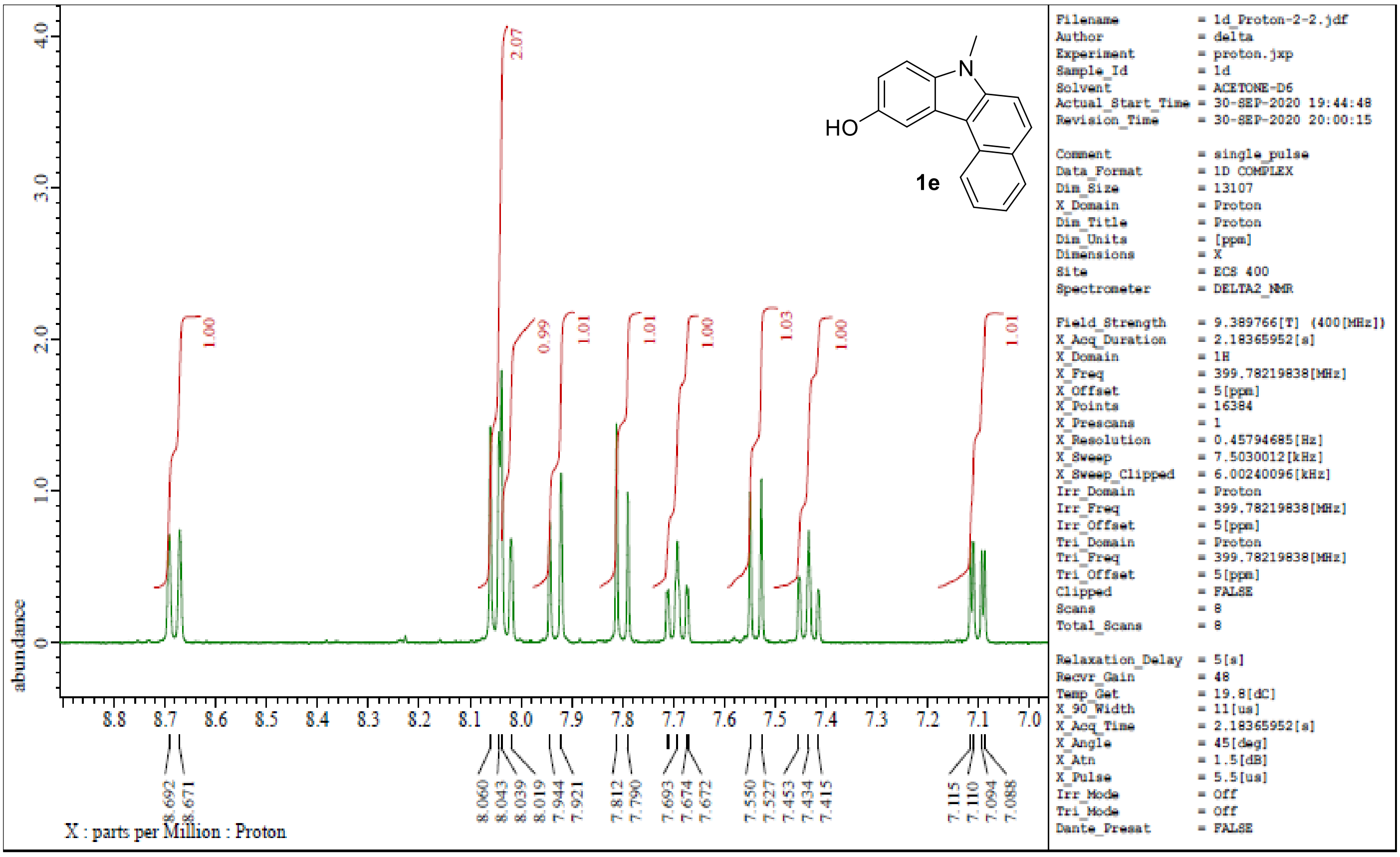

Compound **1e** (^{1}H NMR, 400 MHz, $(CD_3)_2CO$).

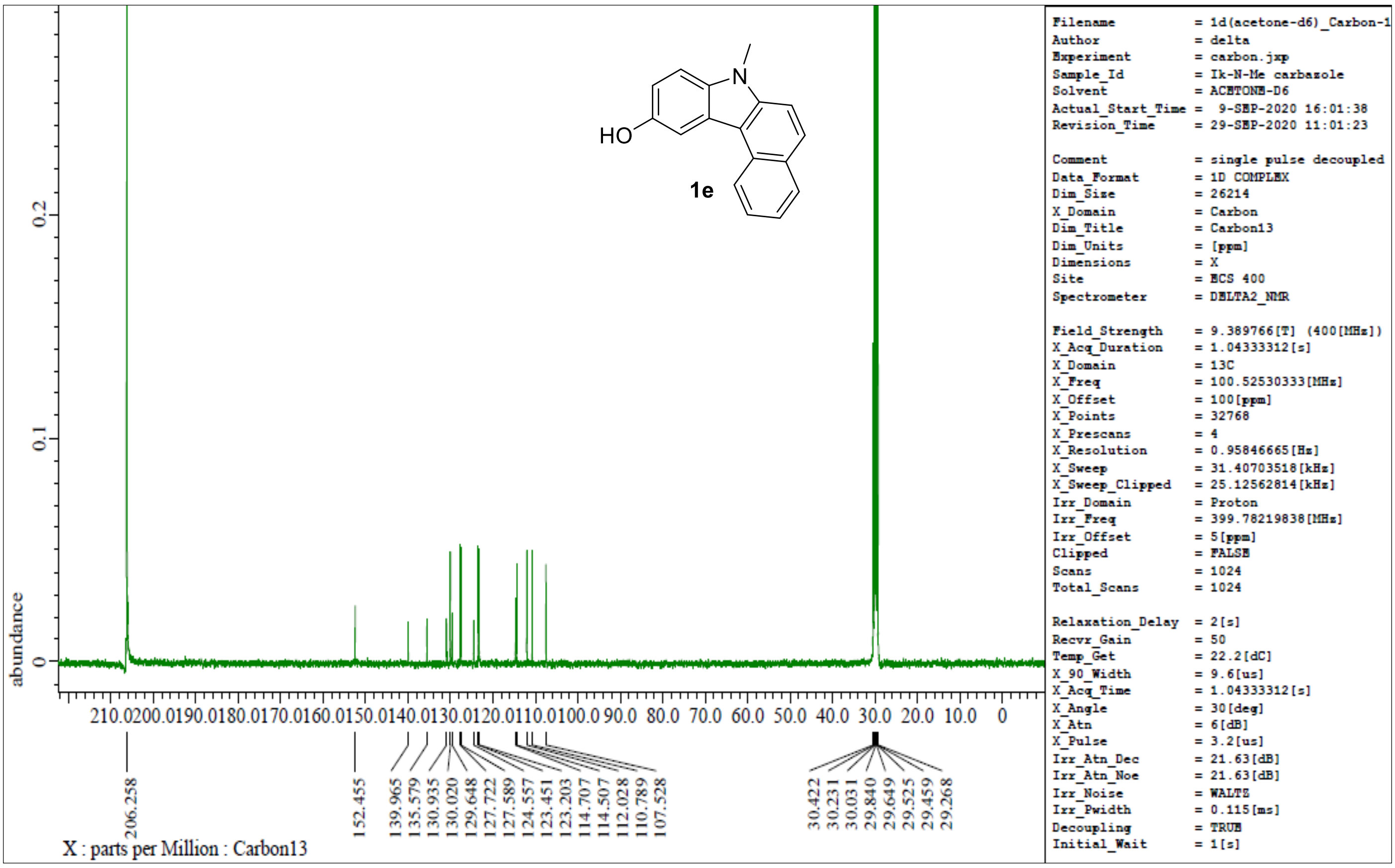

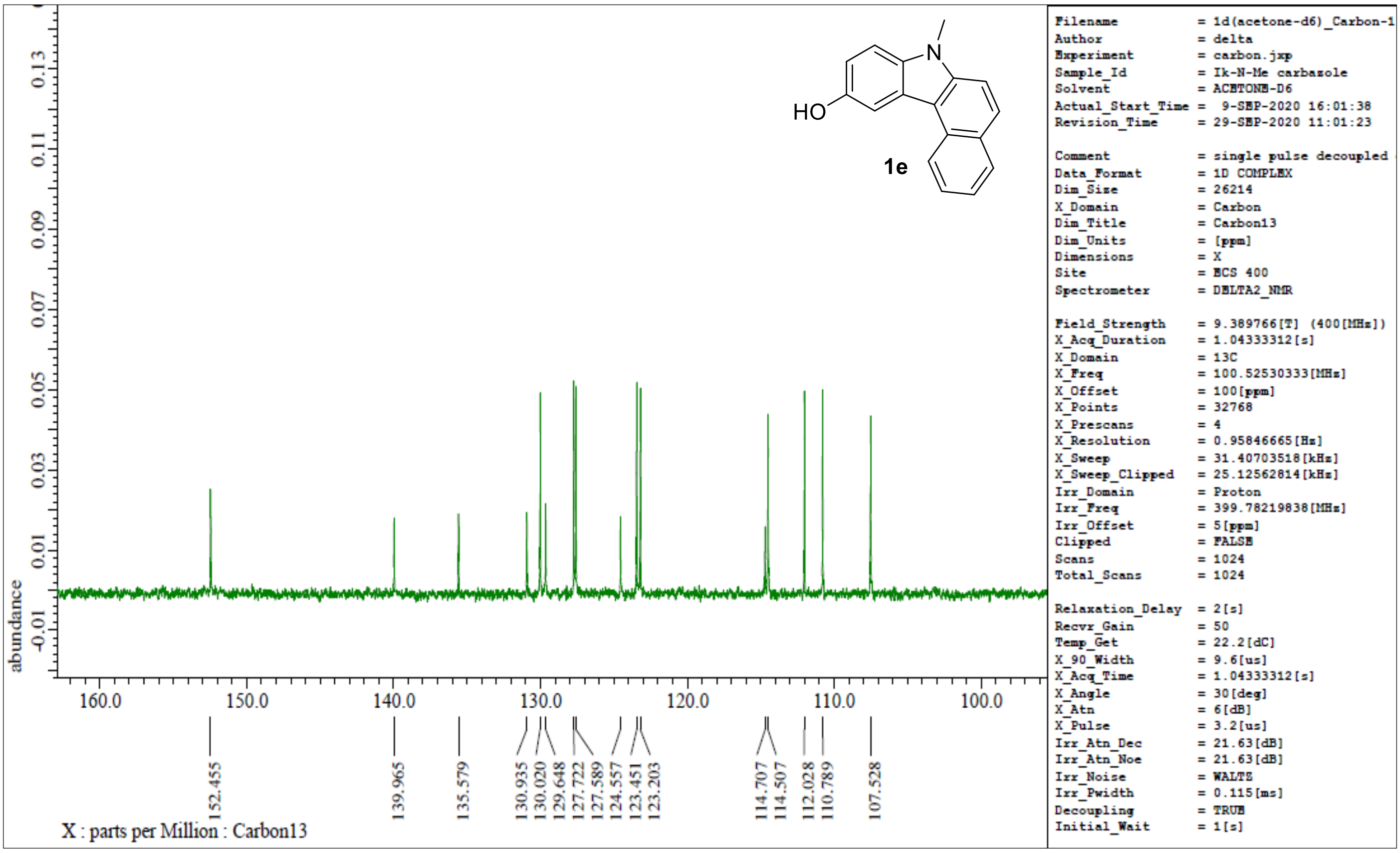

Compound **1e** (^{13}C NMR, 100 MHz, $(CD_3)_2CO$).

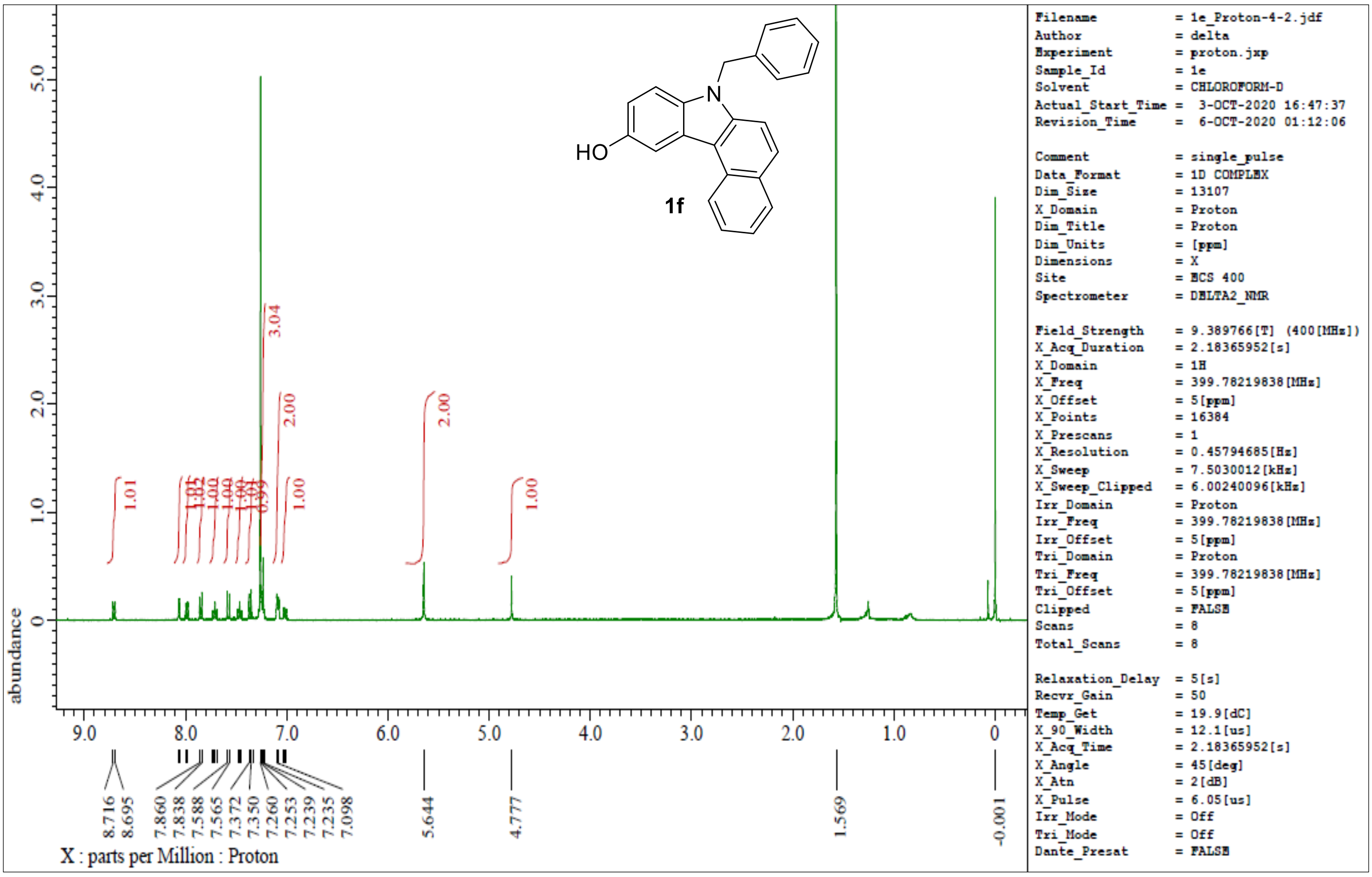

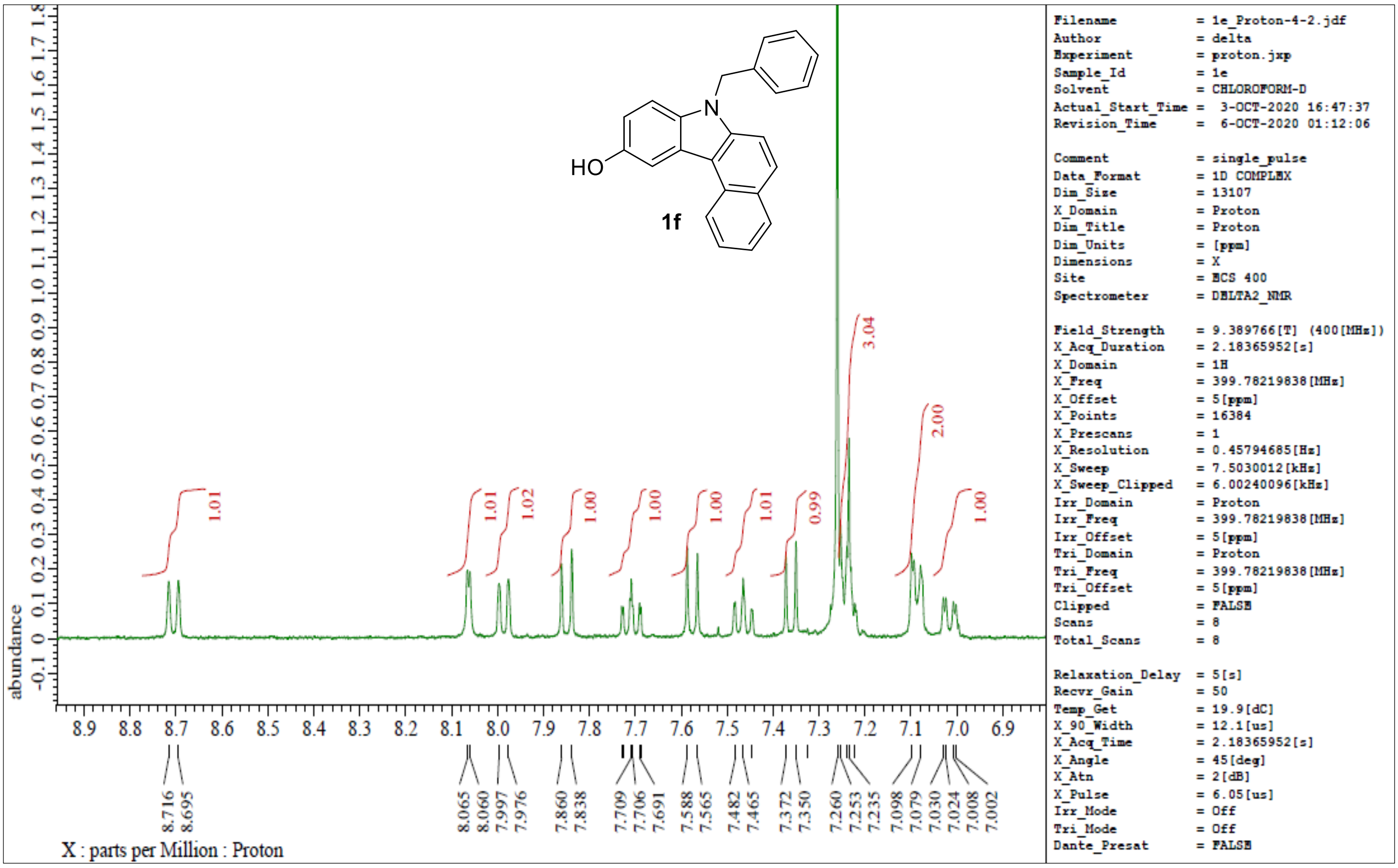

Compound **1f** (^{1}H NMR, 400 MHz, $CDCl_3$).

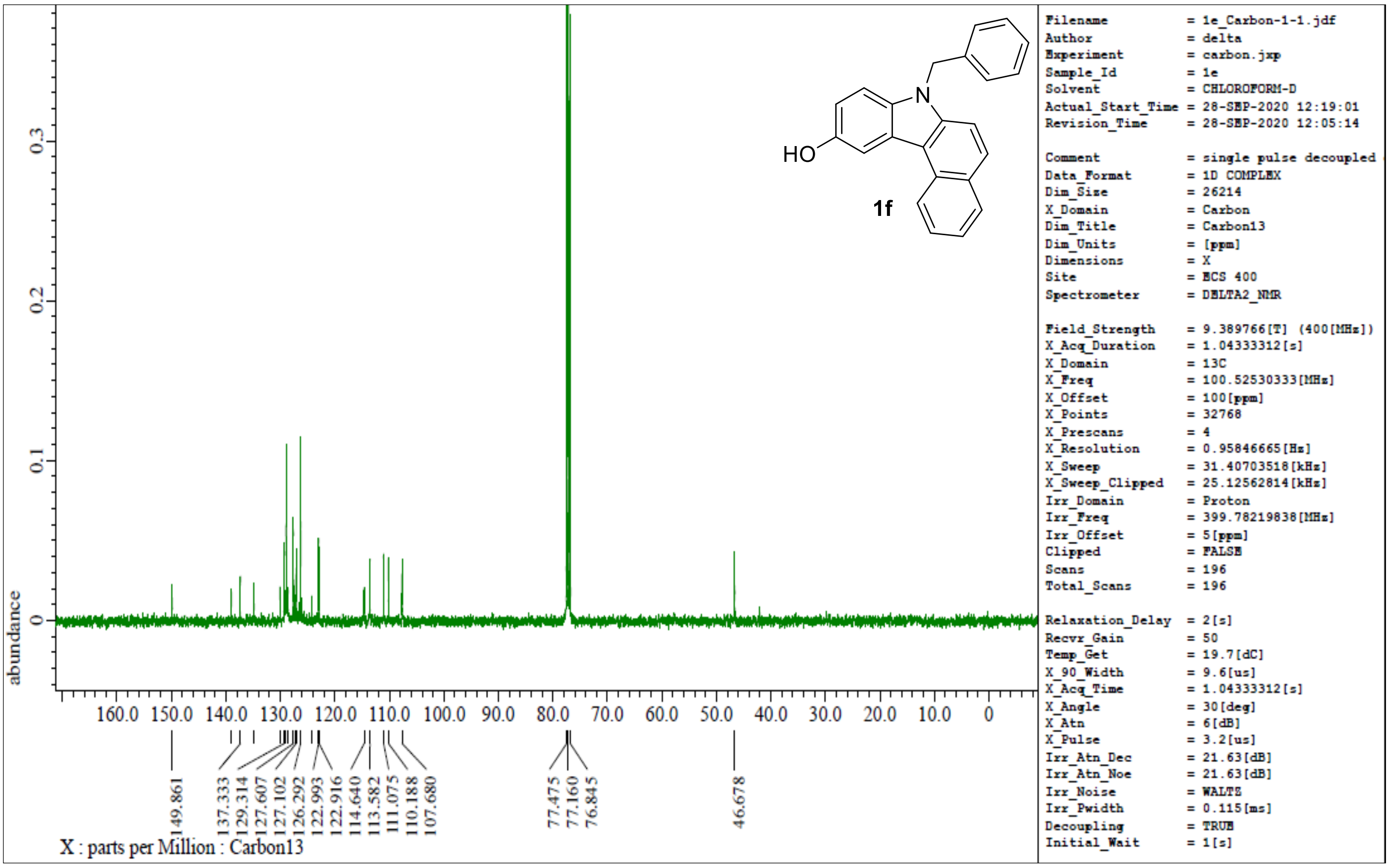

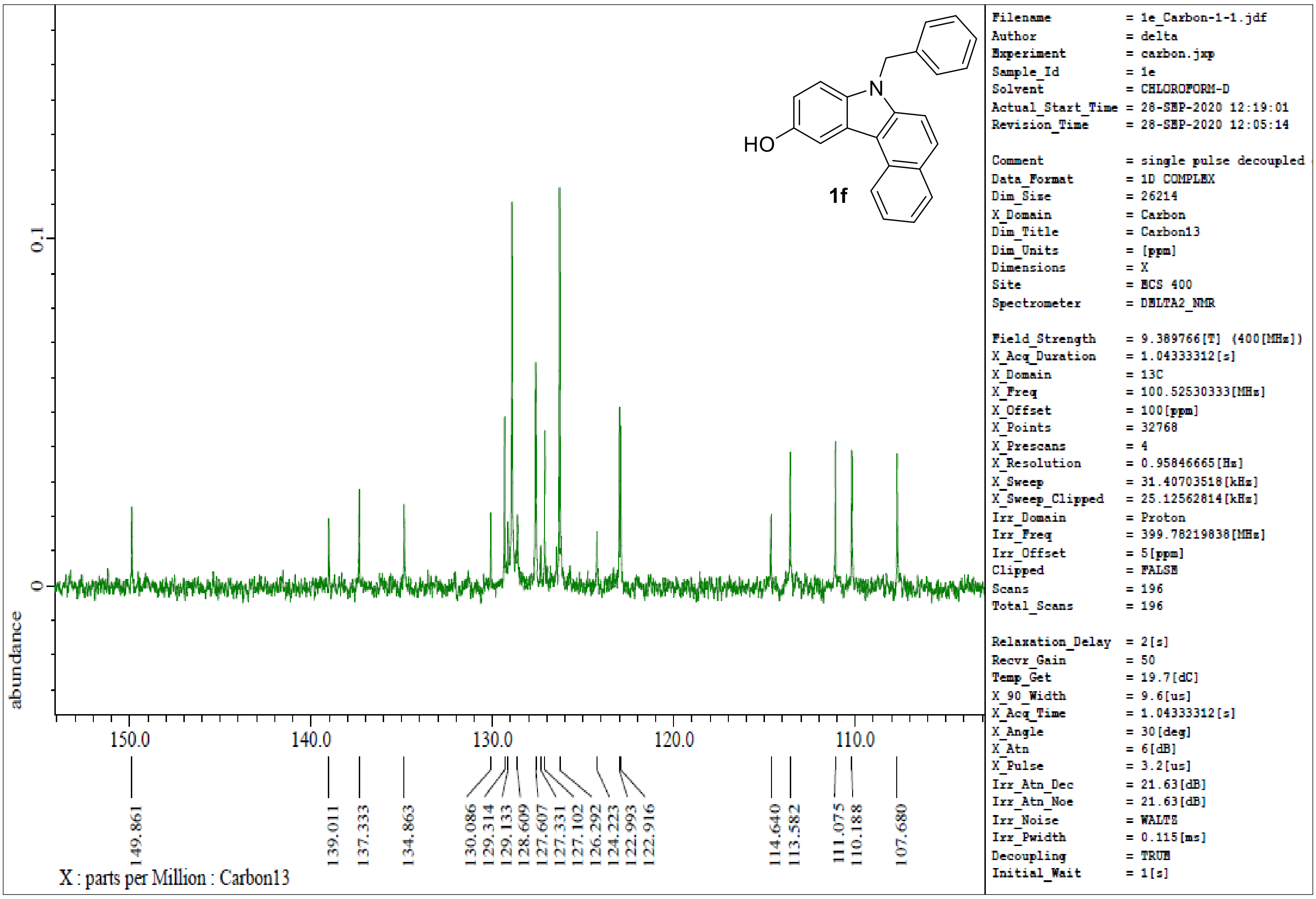

Compound **1f** (^{13}C NMR, 100 MHz, $CDCl_3$).

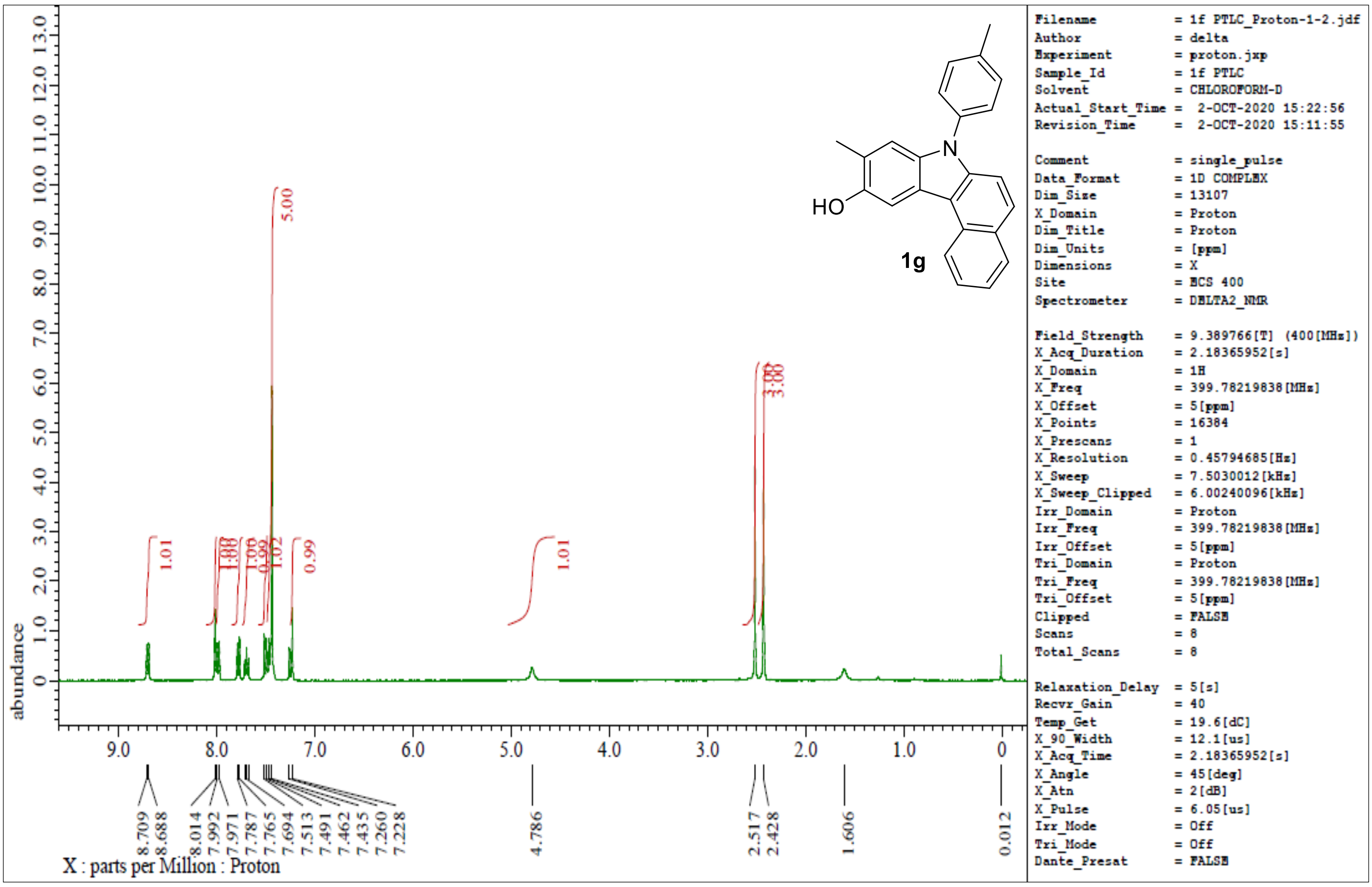

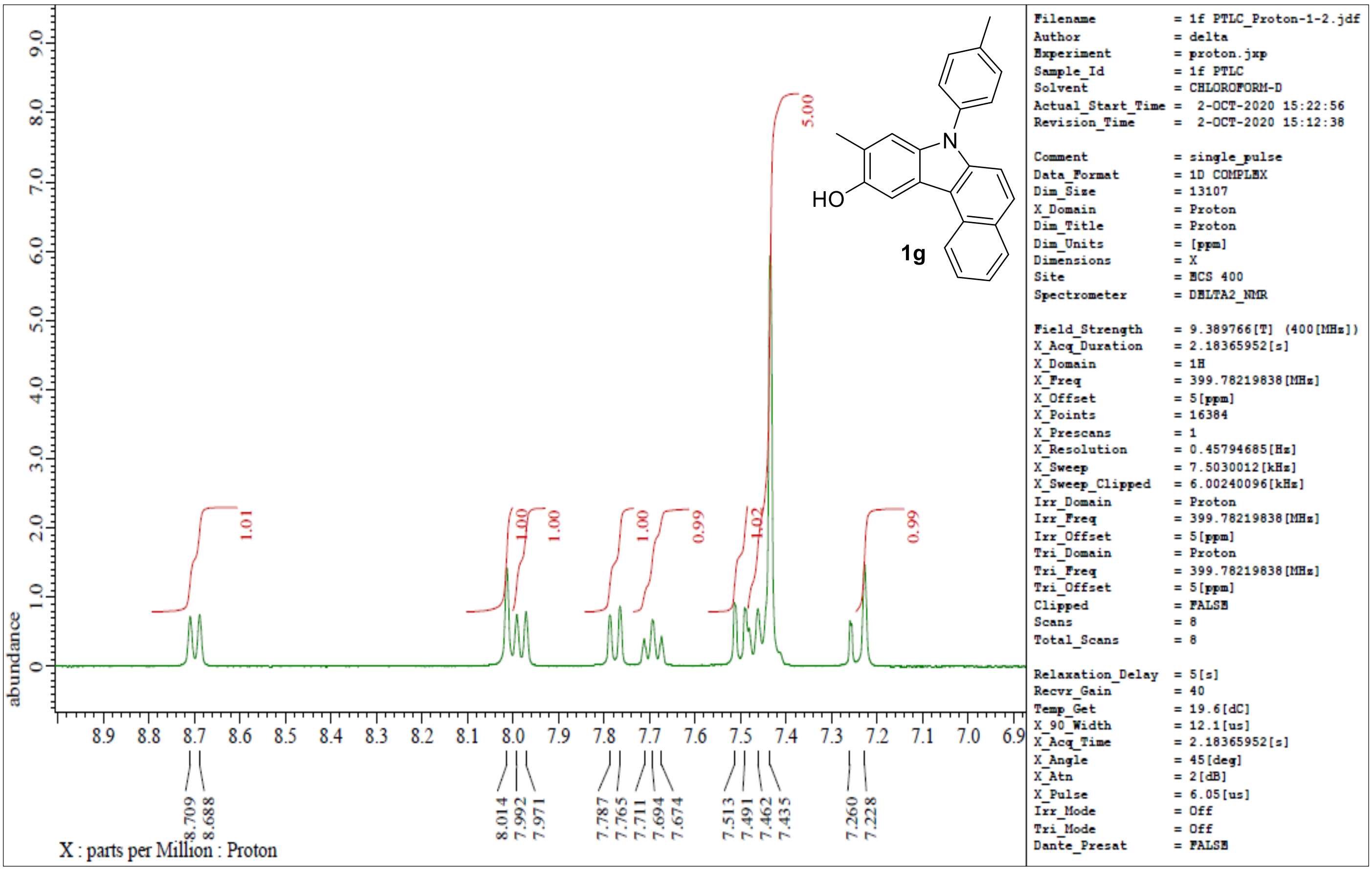

Compound **1g** (^{1}H NMR, 400 MHz, $CDCl_3$).

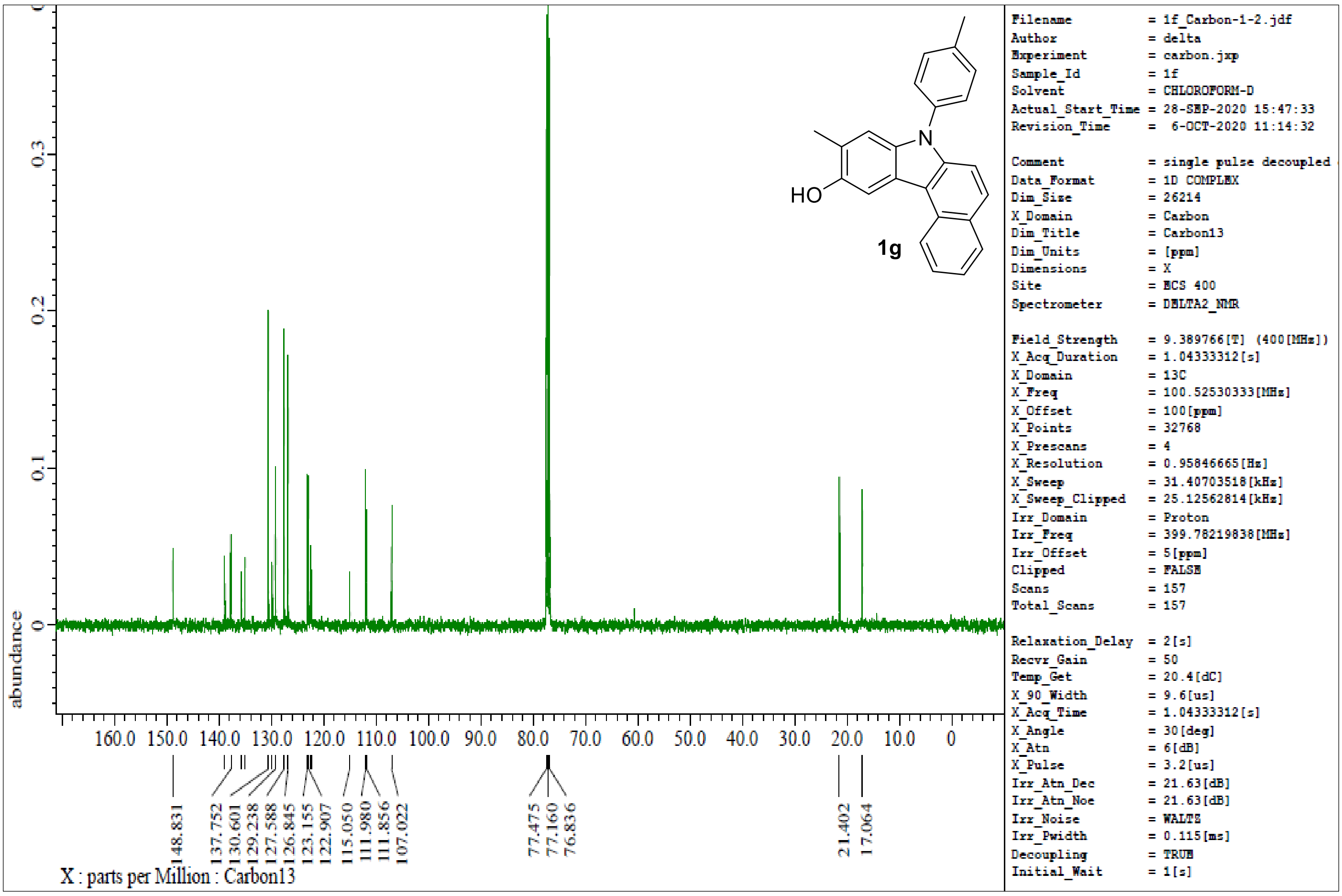

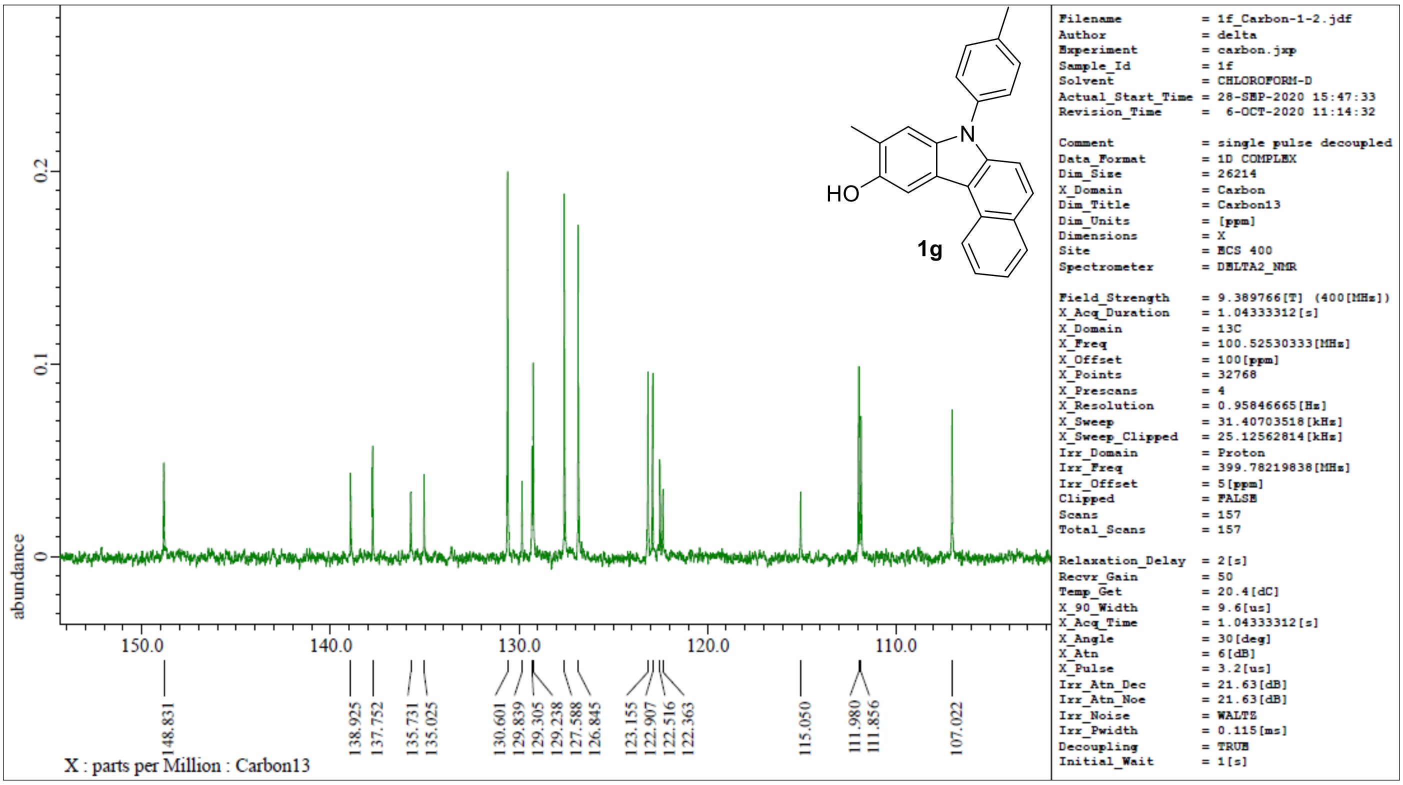

Compound **1g** (^{13}C NMR, 100 MHz, $CDCl_3$).

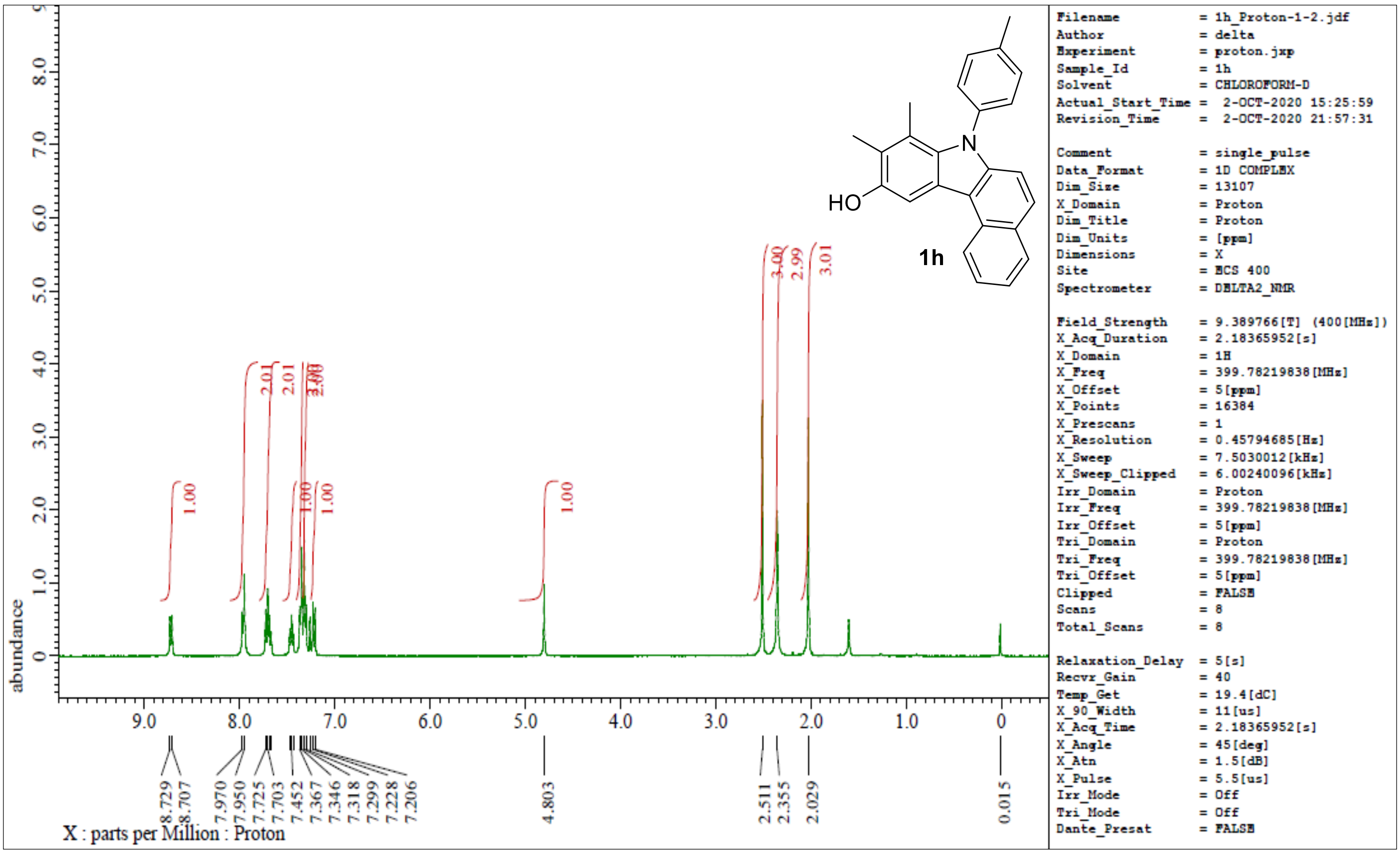

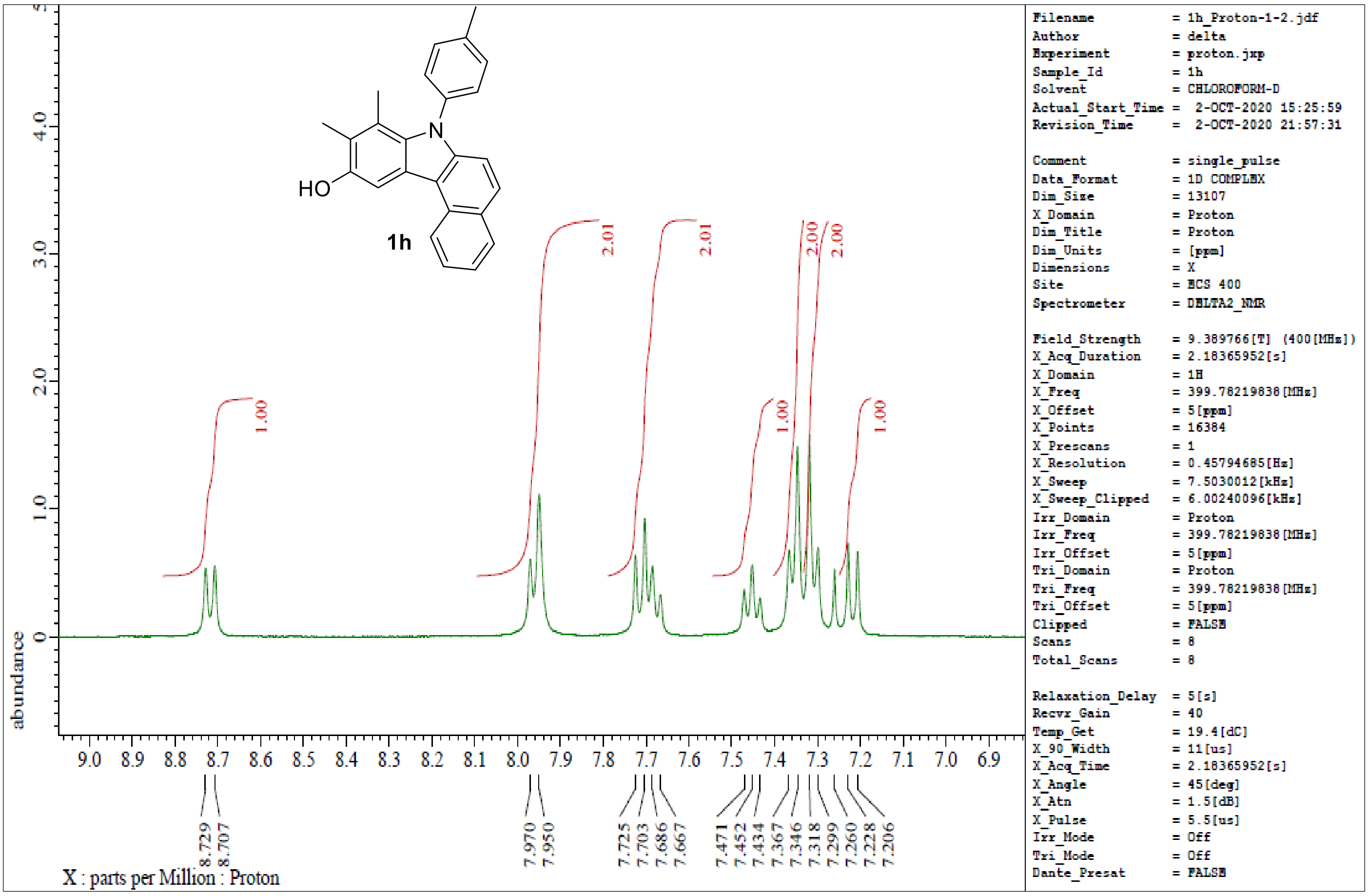

Compound **1h** (^{1}H NMR, 400 MHz, $CDCl_3$).

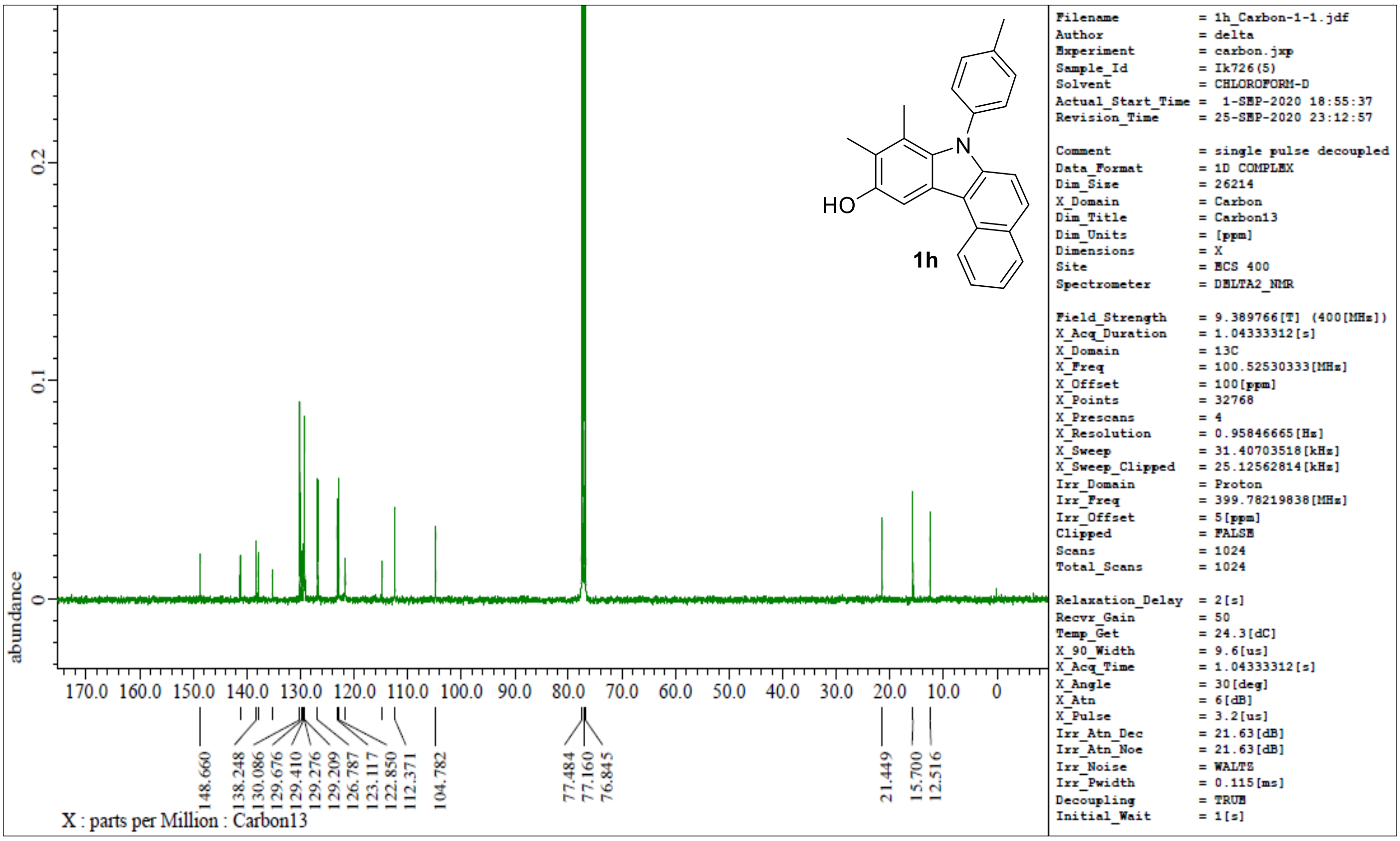

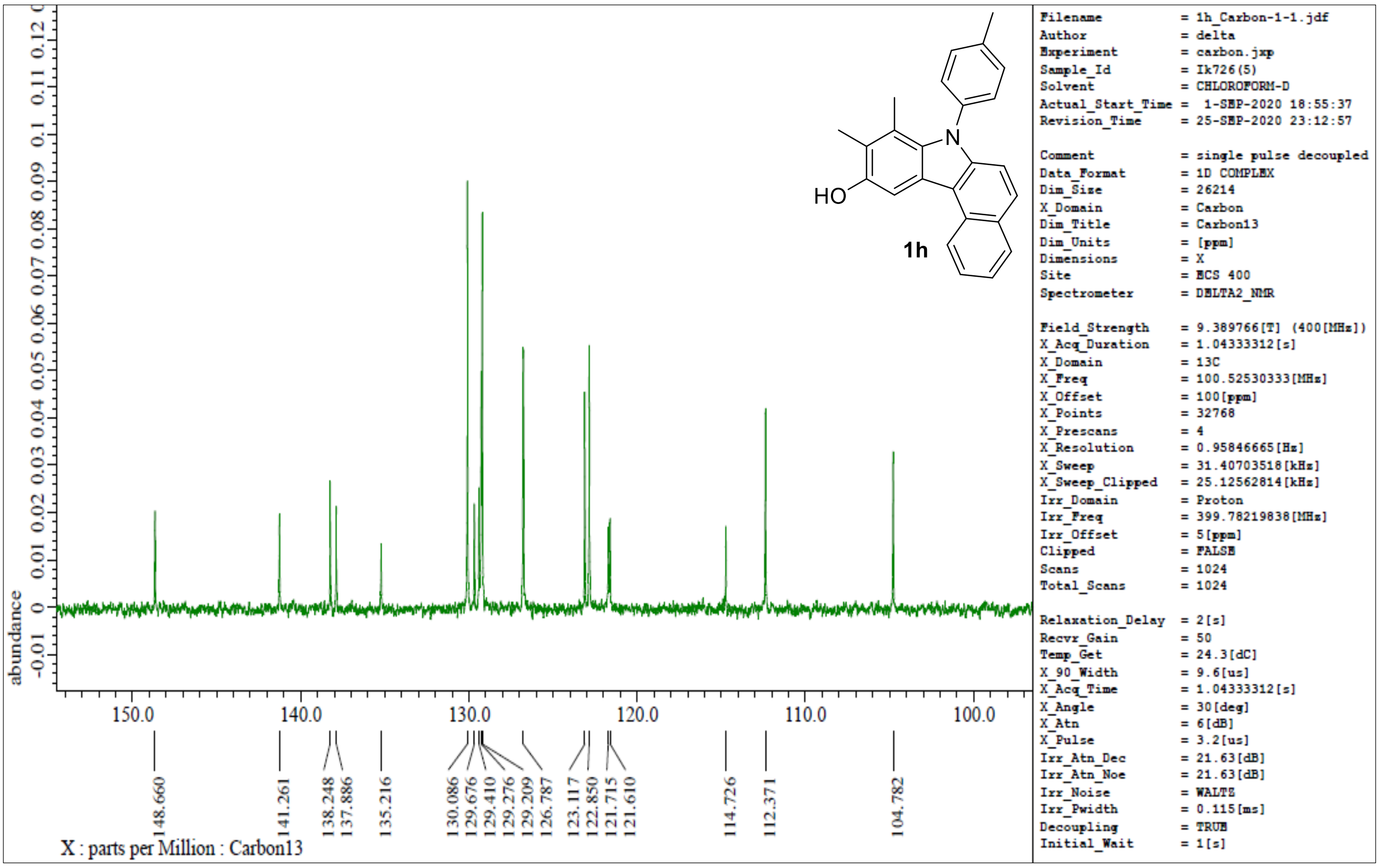

Compound **1h** (^{13}C NMR, 100 MHz, $CDCl_3$).

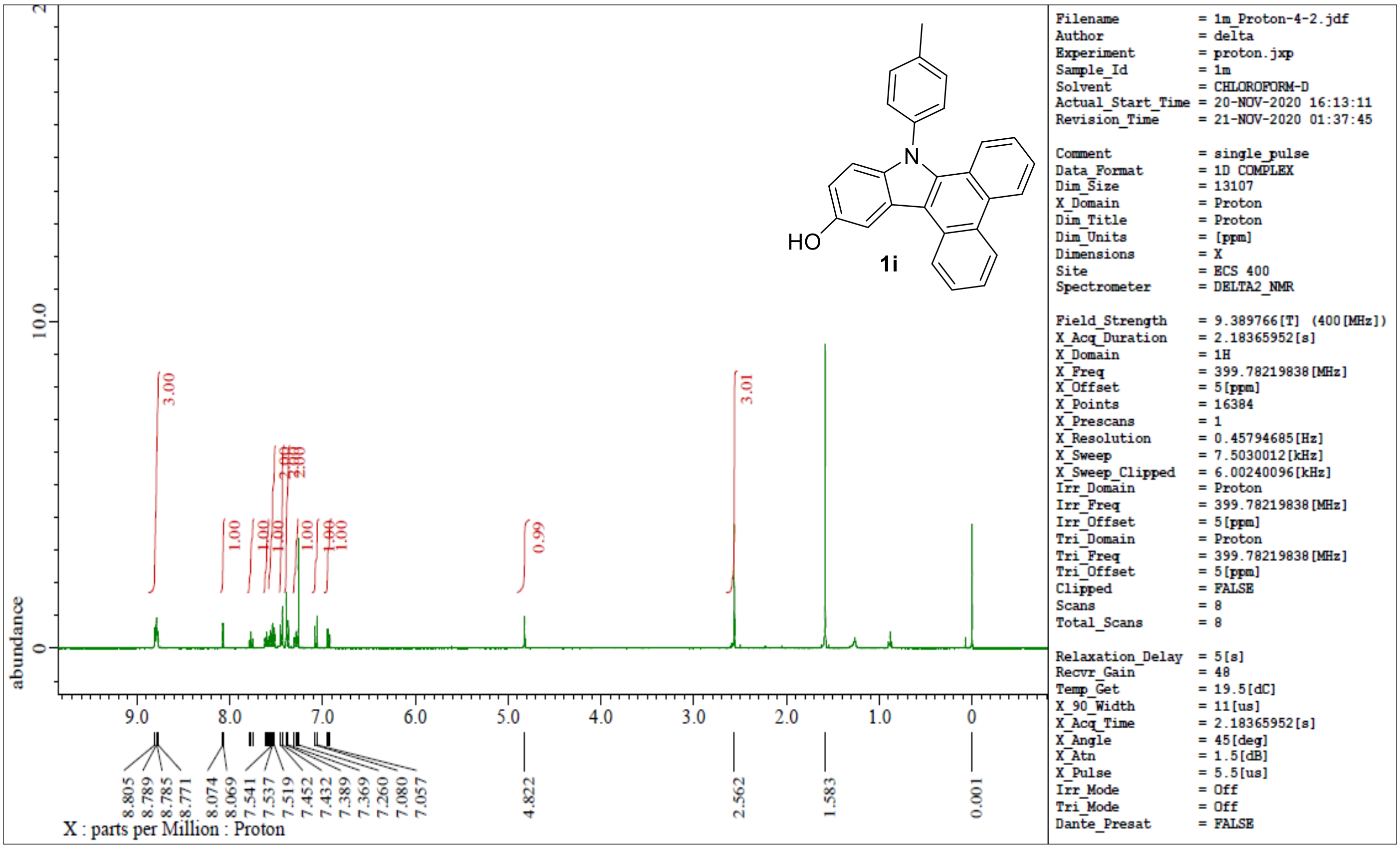

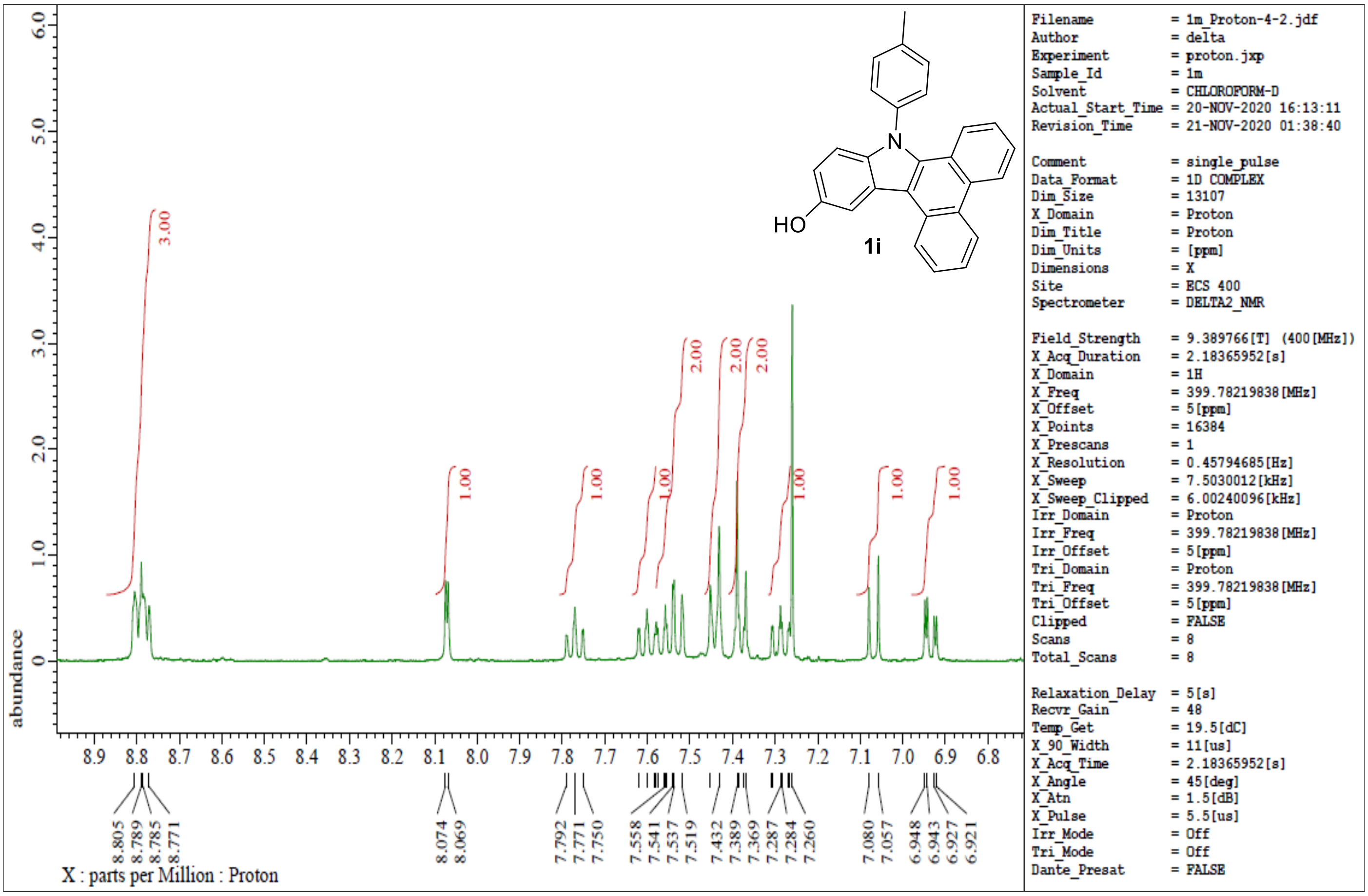

Compound **1i** (^{1}H NMR, 400 MHz, $CDCl_3$).

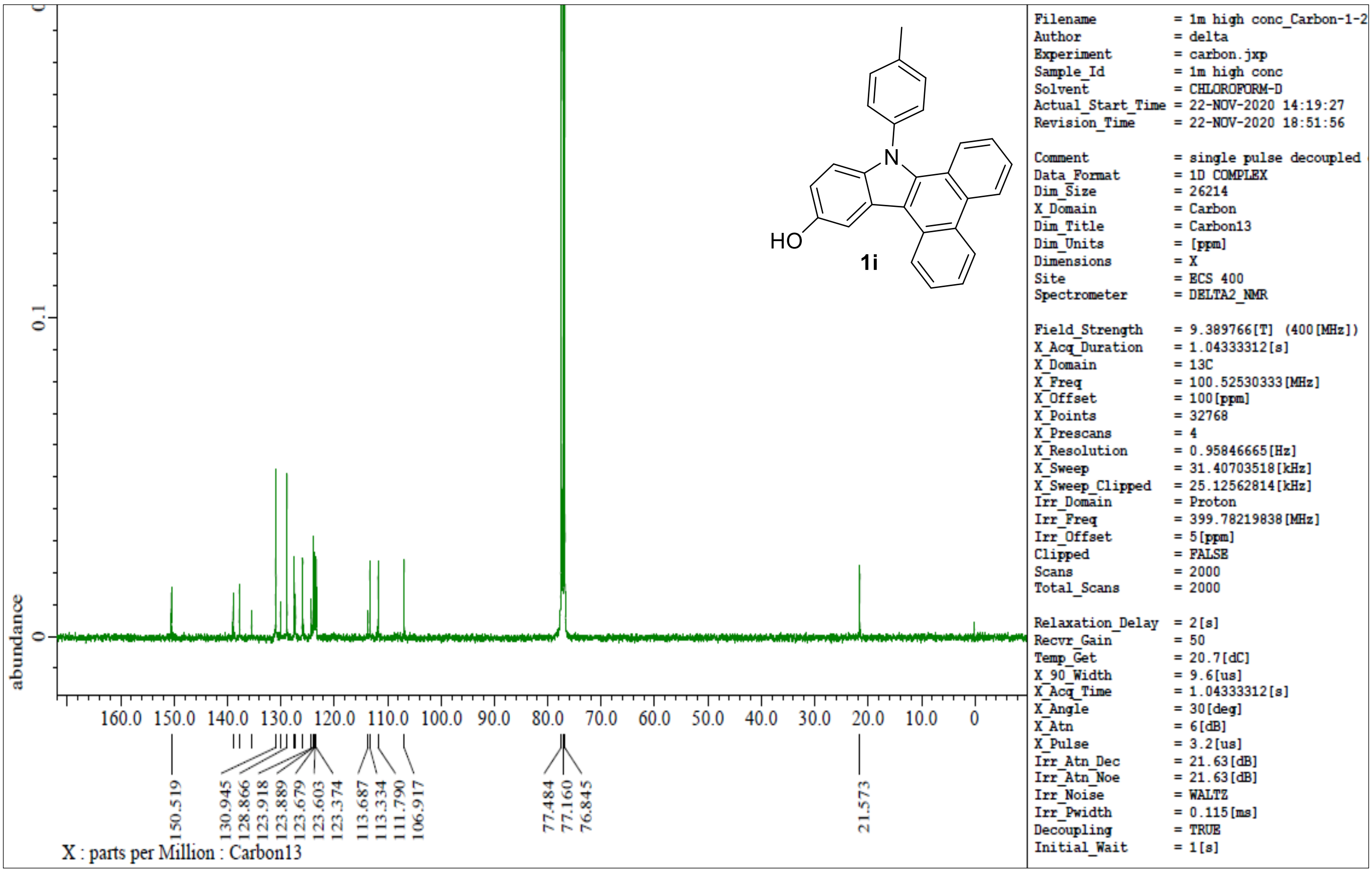

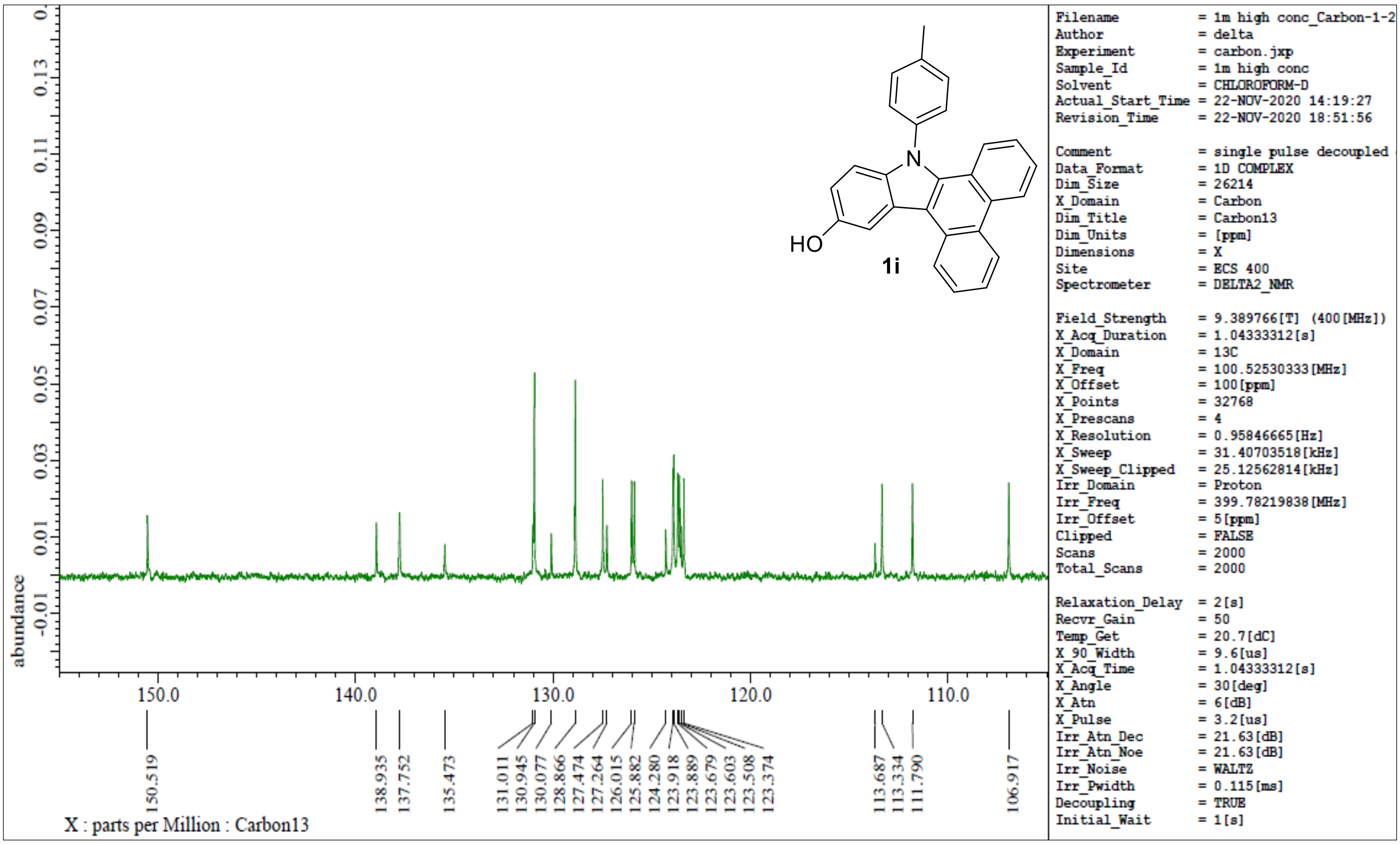

Compound **1i** (^{13}C NMR, 100 MHz, $CDCl_3$).

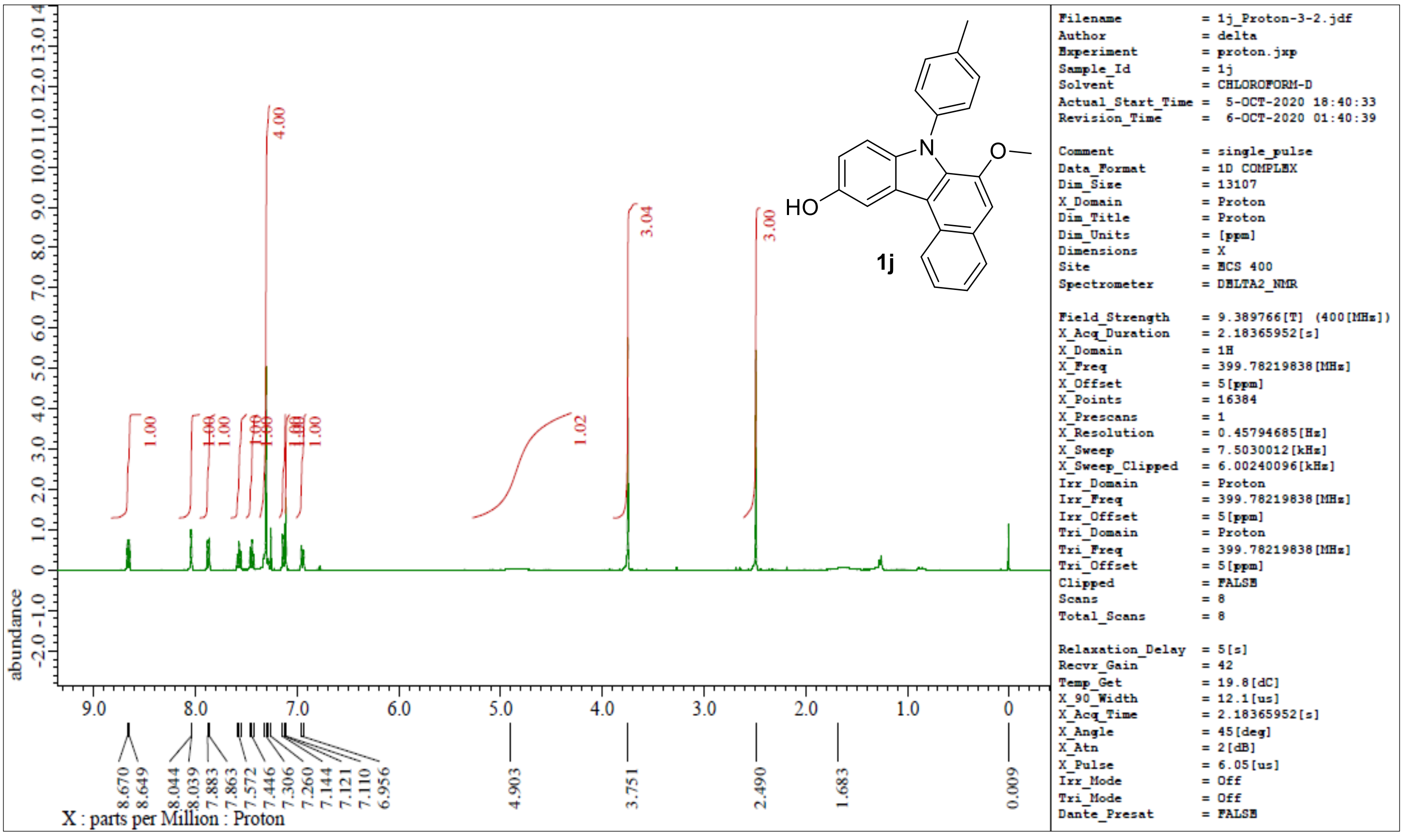

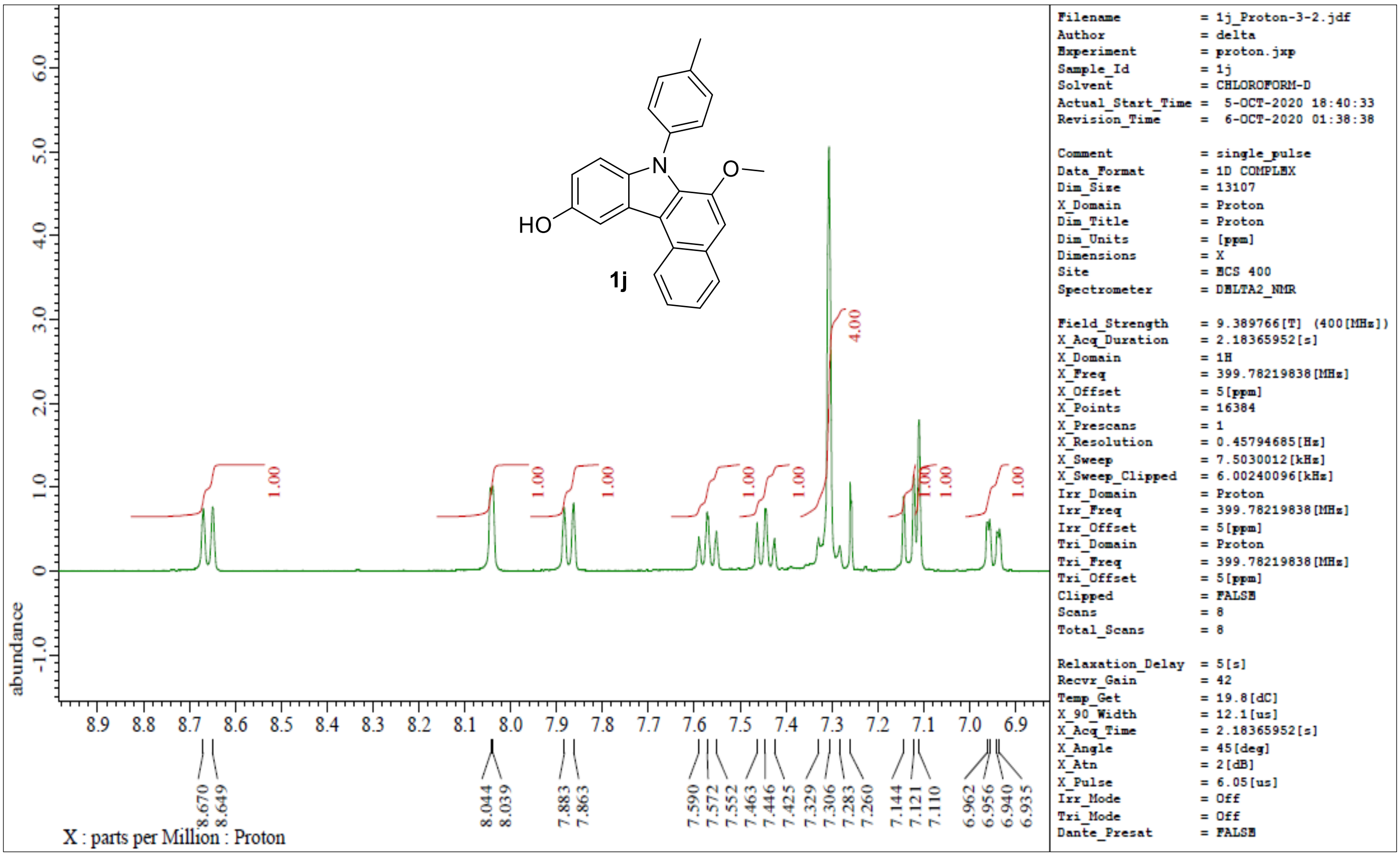

Compound **1j** (^{1}H NMR, 400 MHz, $CDCl_3$).

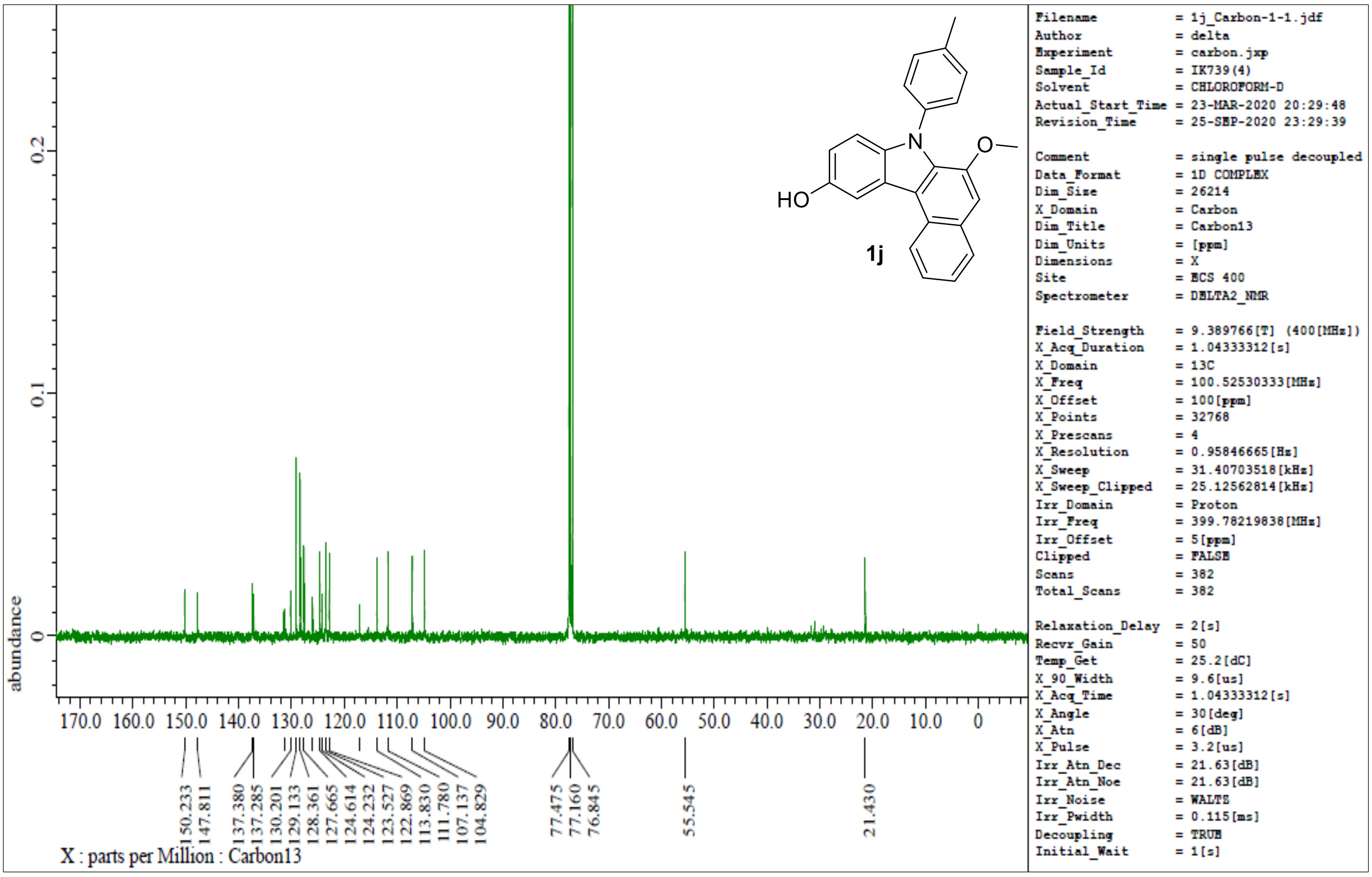

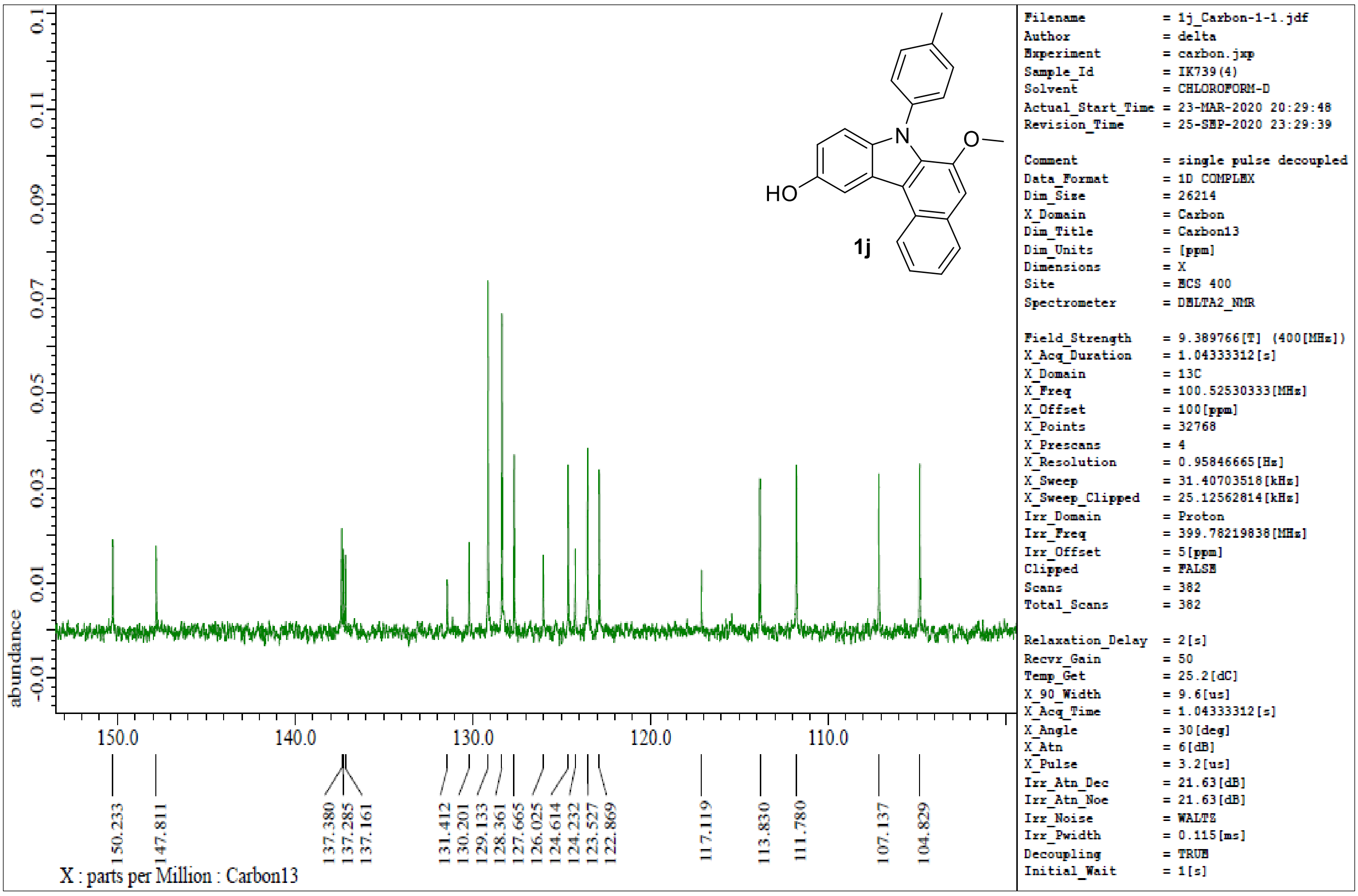

Compound **1j** (^{13}C NMR, 100 MHz, $CDCl_3$).

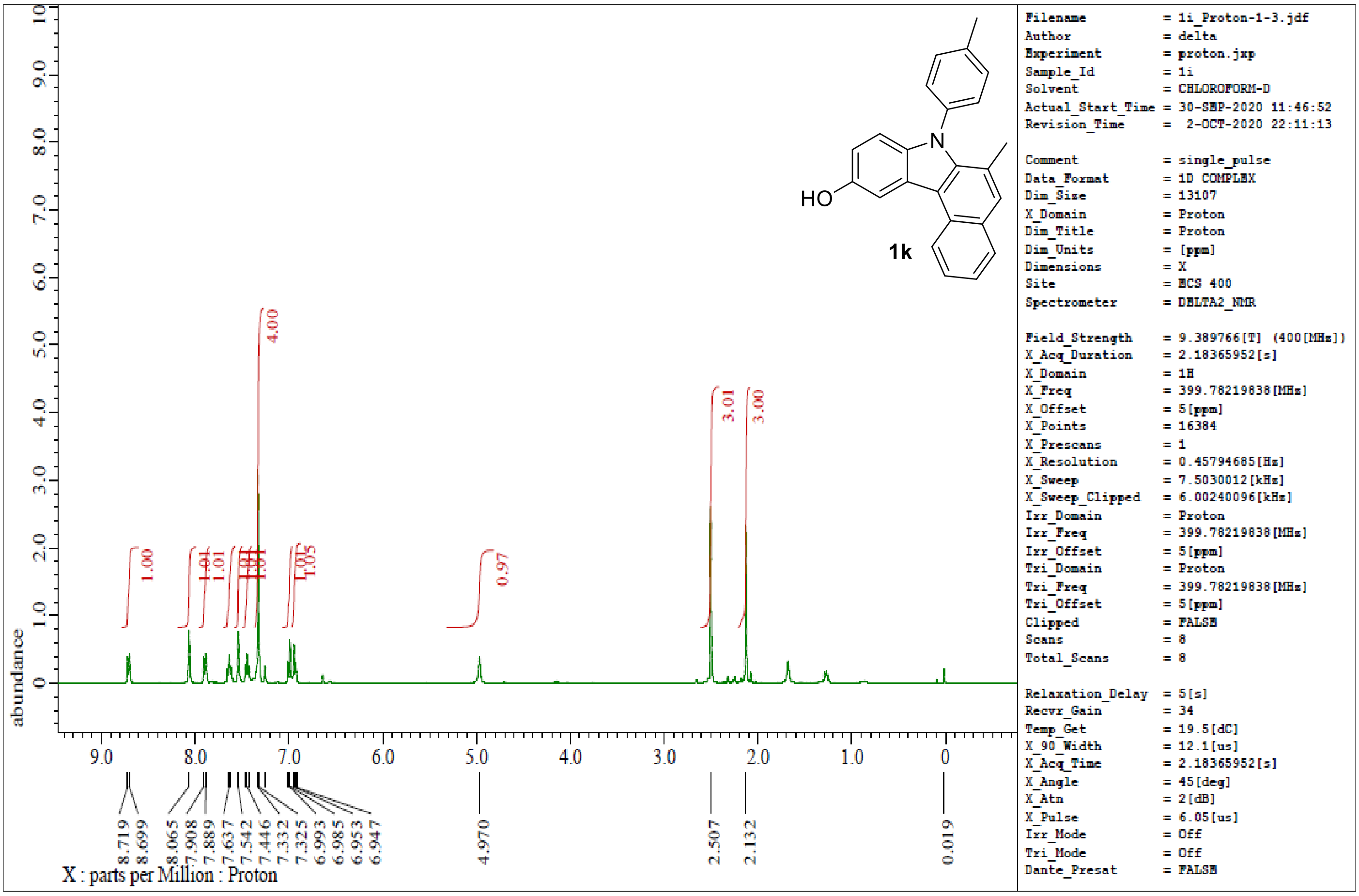

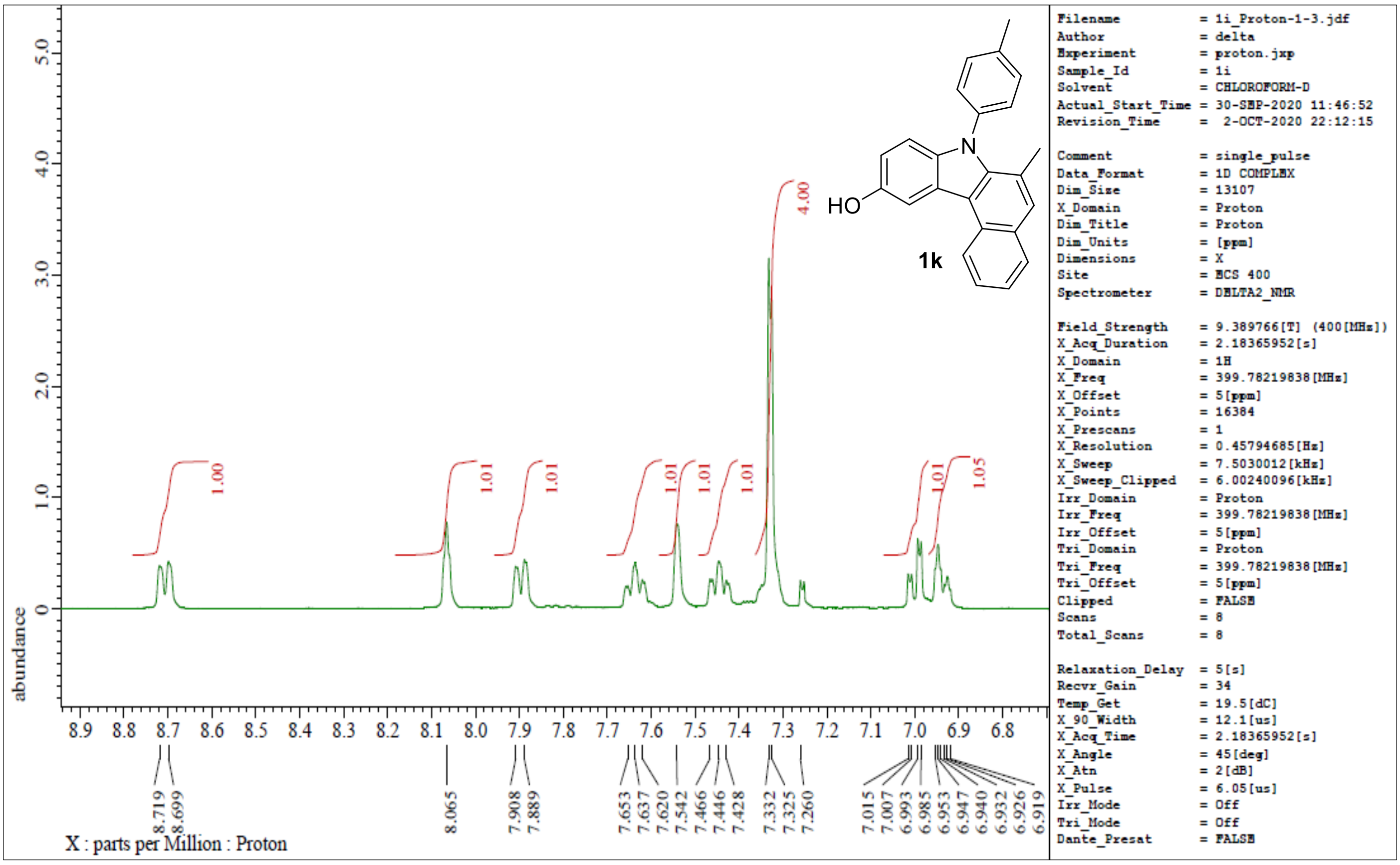

Compound **1k** (^{1}H NMR, 400 MHz, $CDCl_3$).

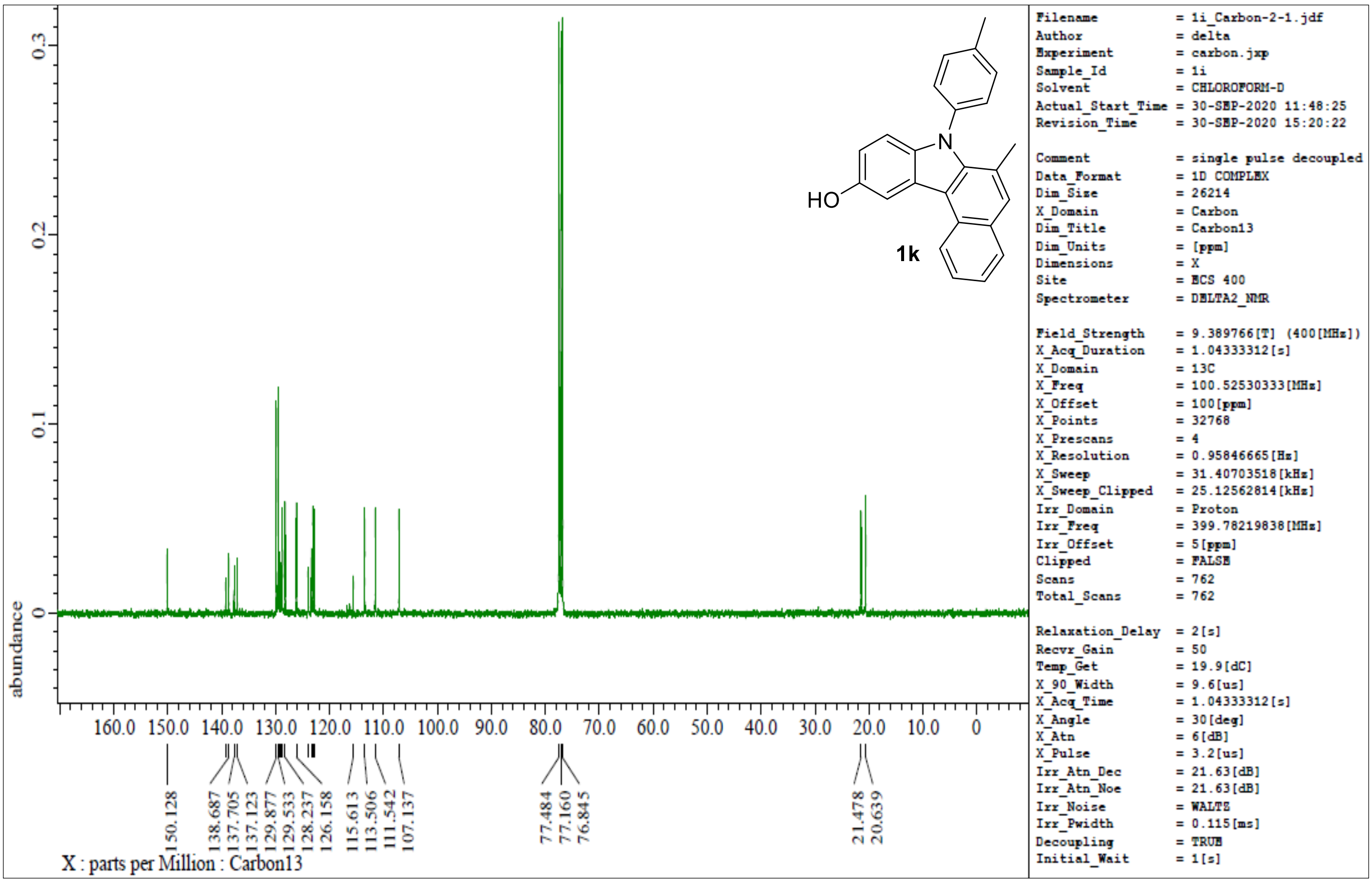

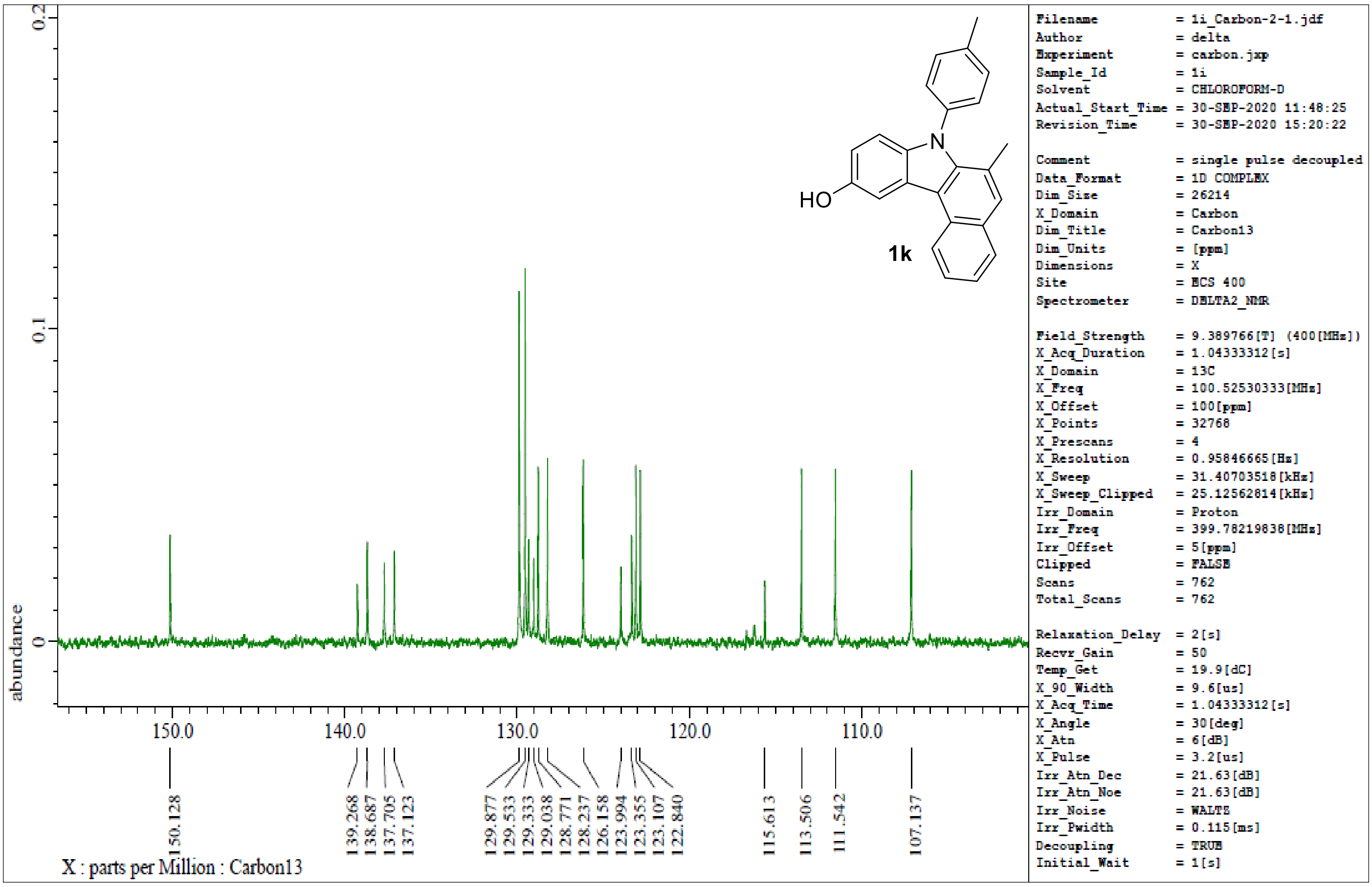

Compound **1k** (^{13}C NMR, 100 MHz, $CDCl_3$).

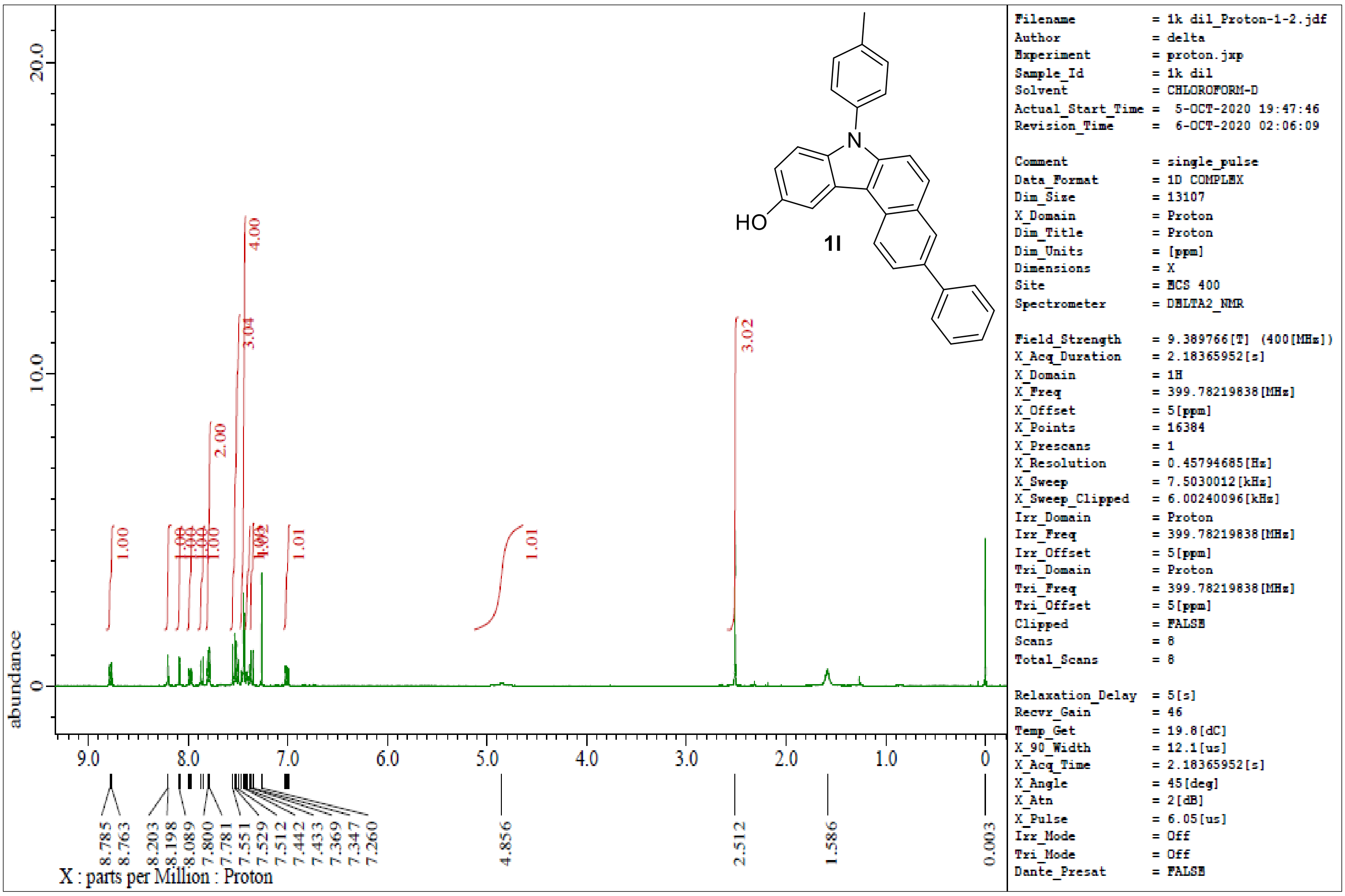

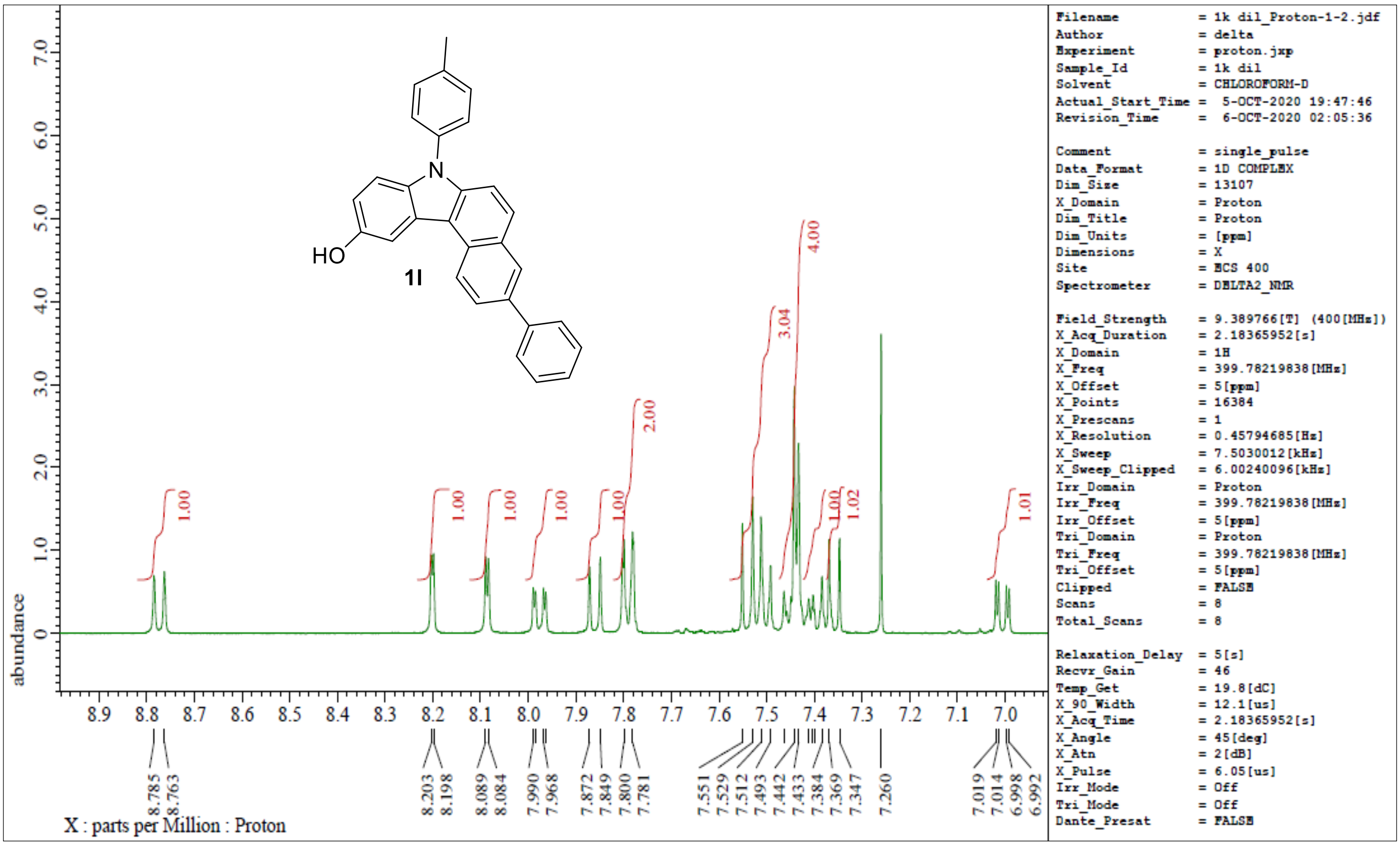

Compound **1l** (^{1}H NMR, 400 MHz, $CDCl_3$).

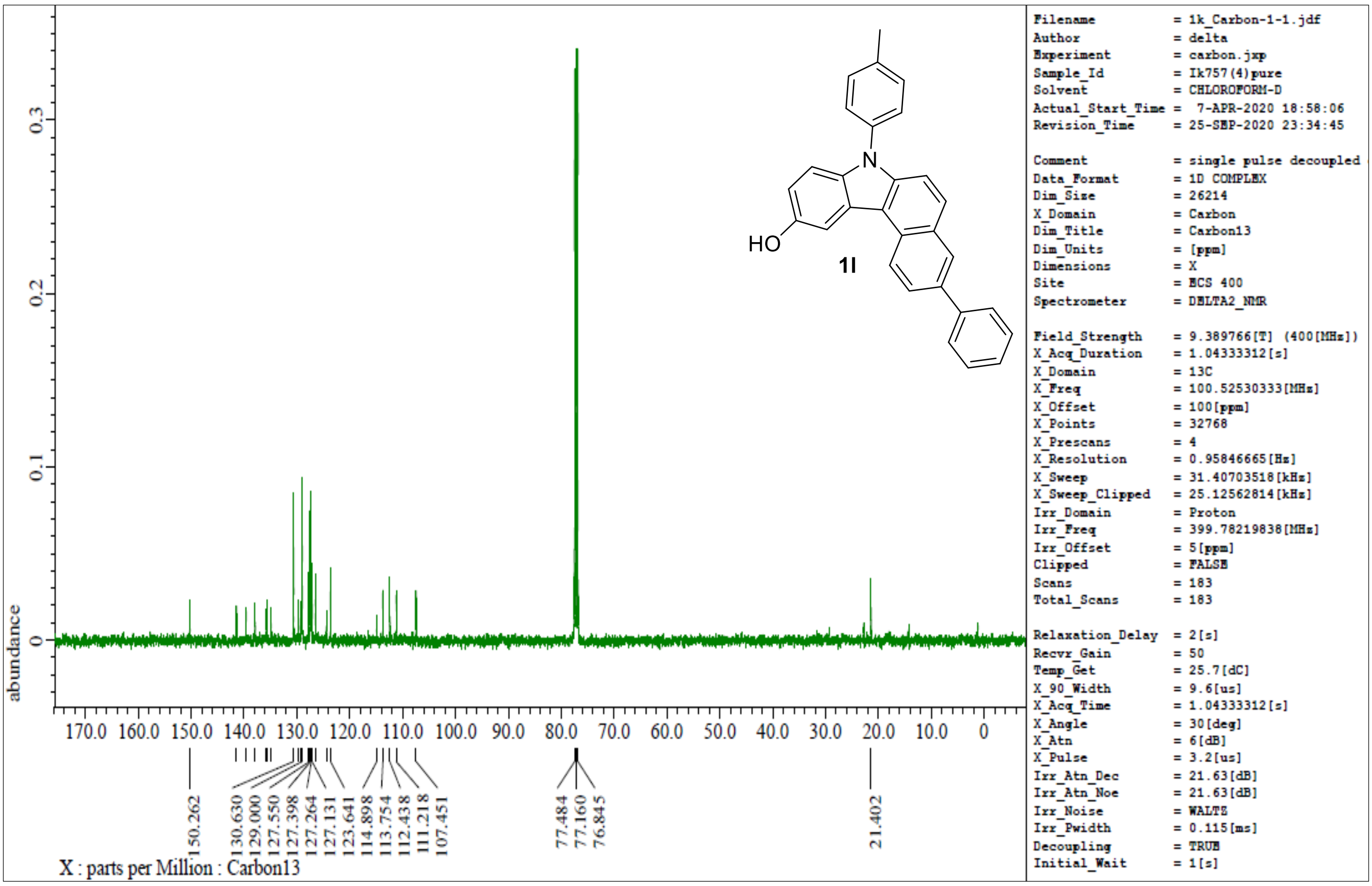

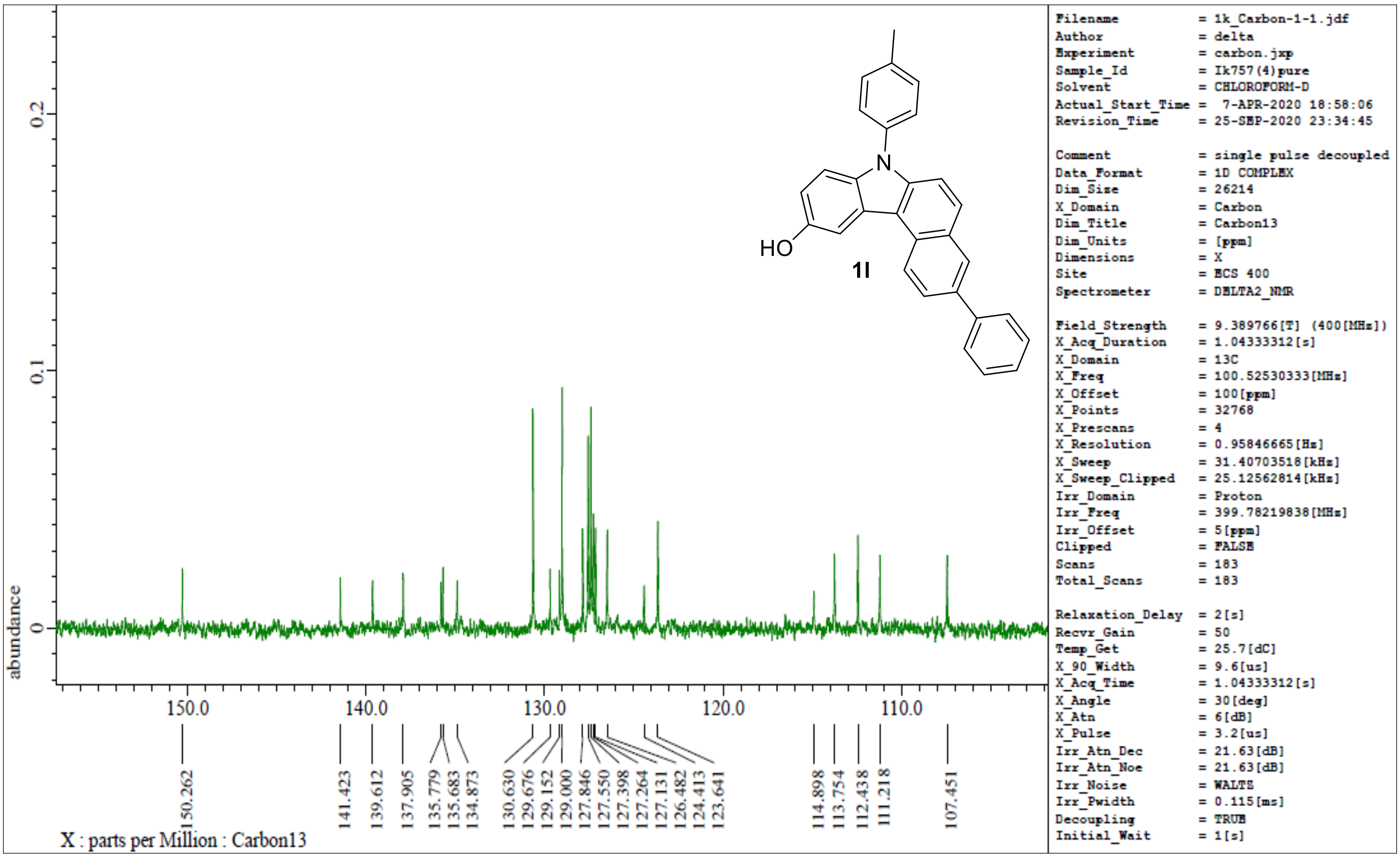

Compound **1l** (^{13}C NMR, 100 MHz, $CDCl_3$).

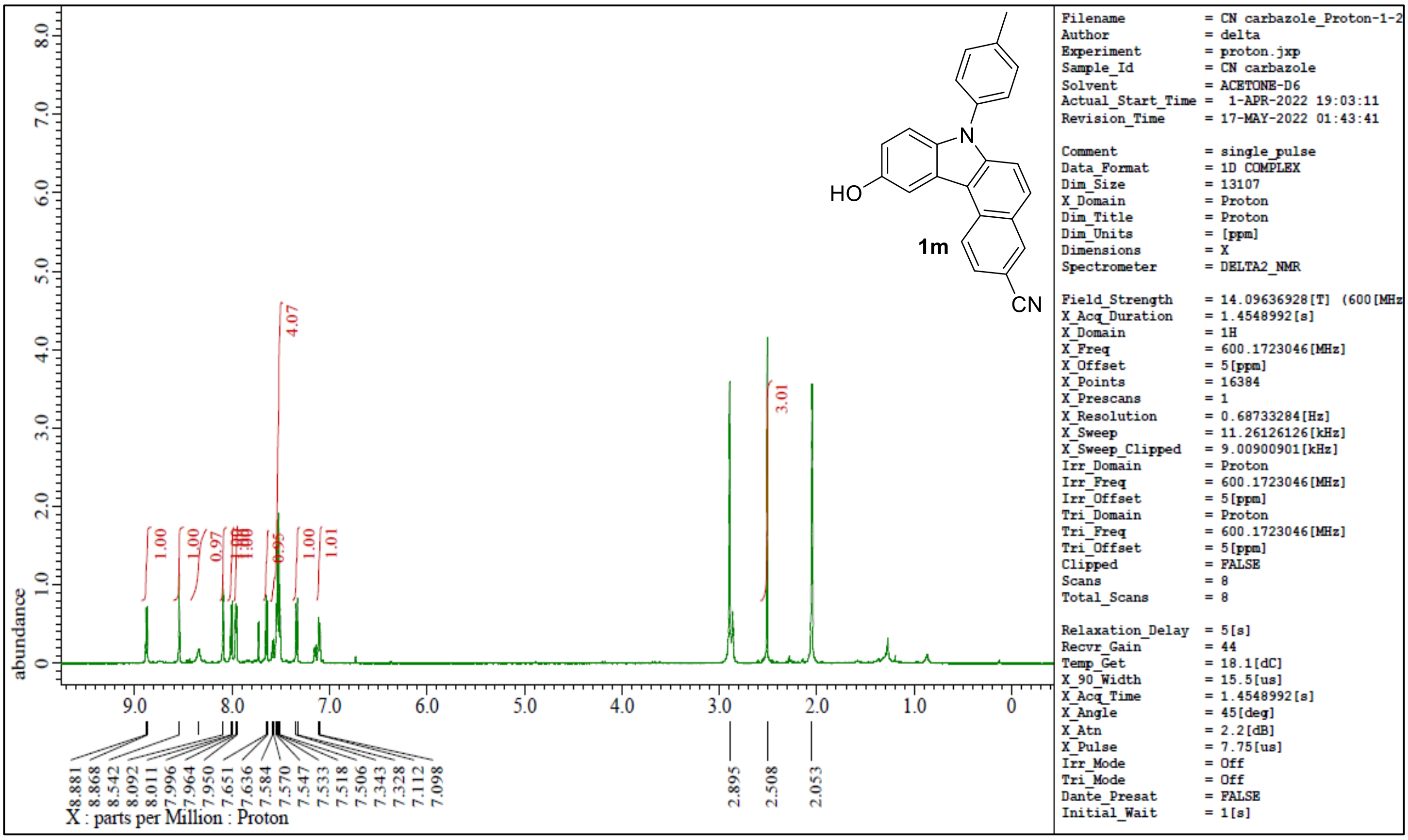

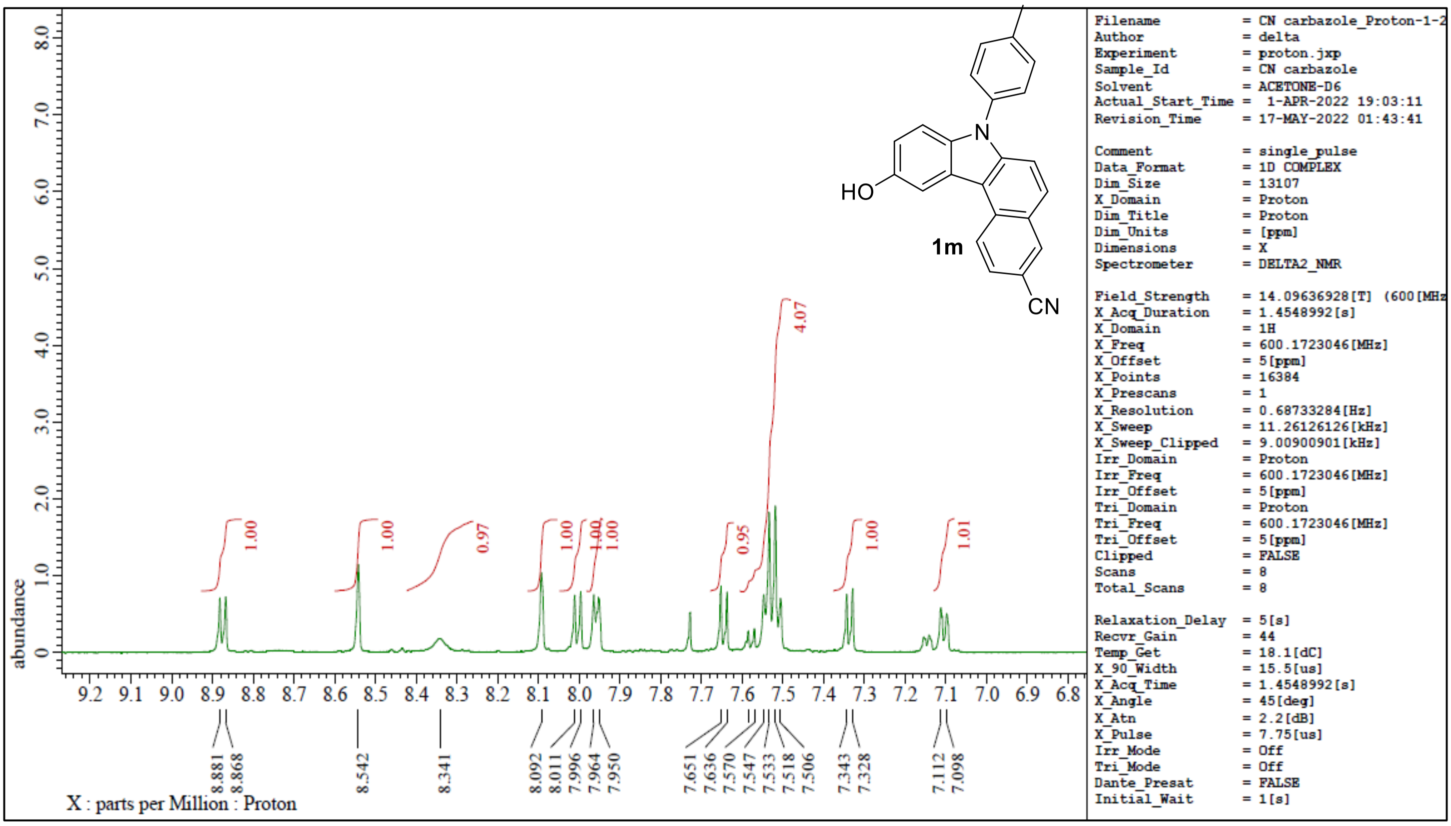

Compound **1m** (^{1}H NMR, 600 MHz, $(CD_3)_2CO$).

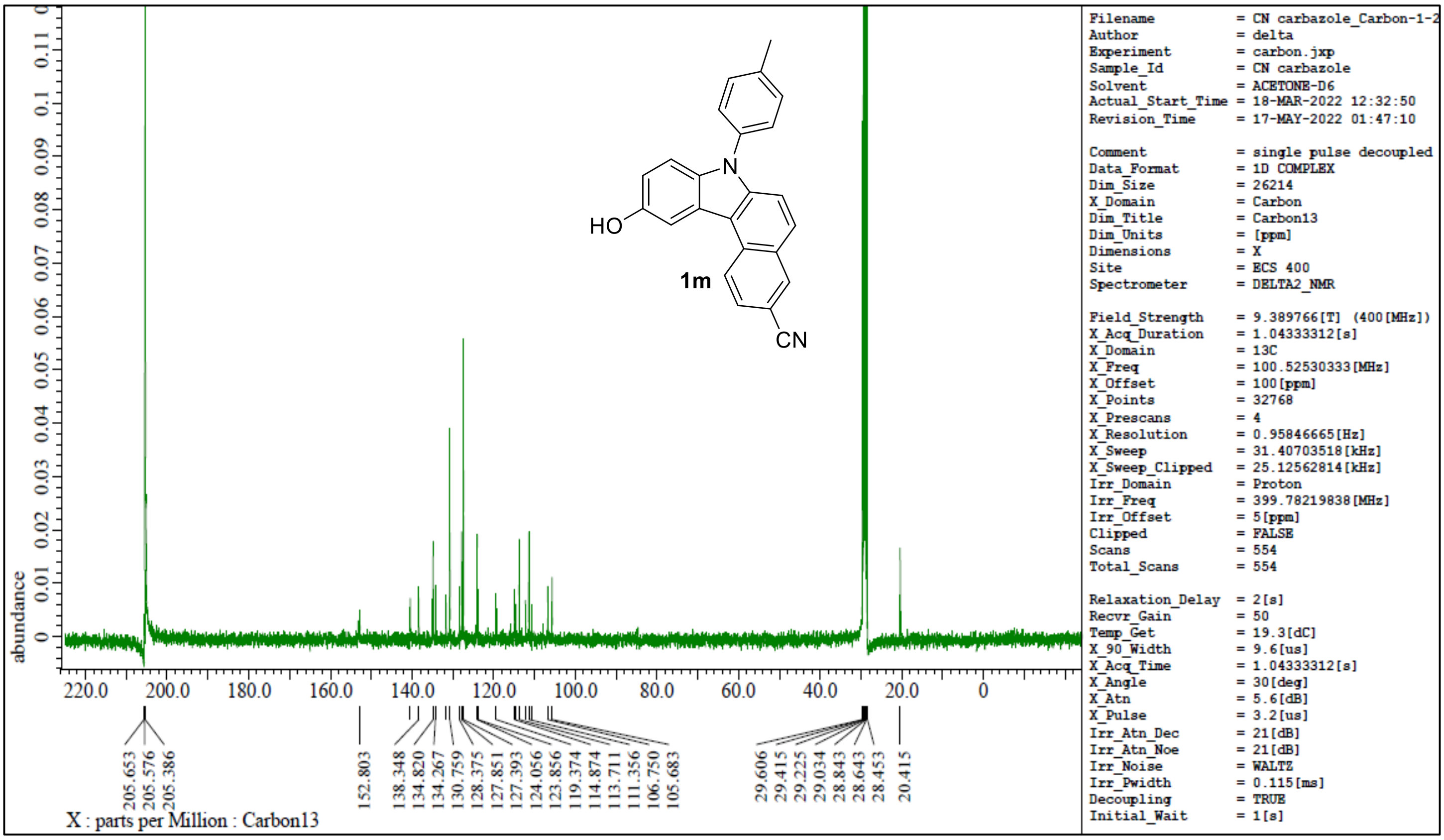

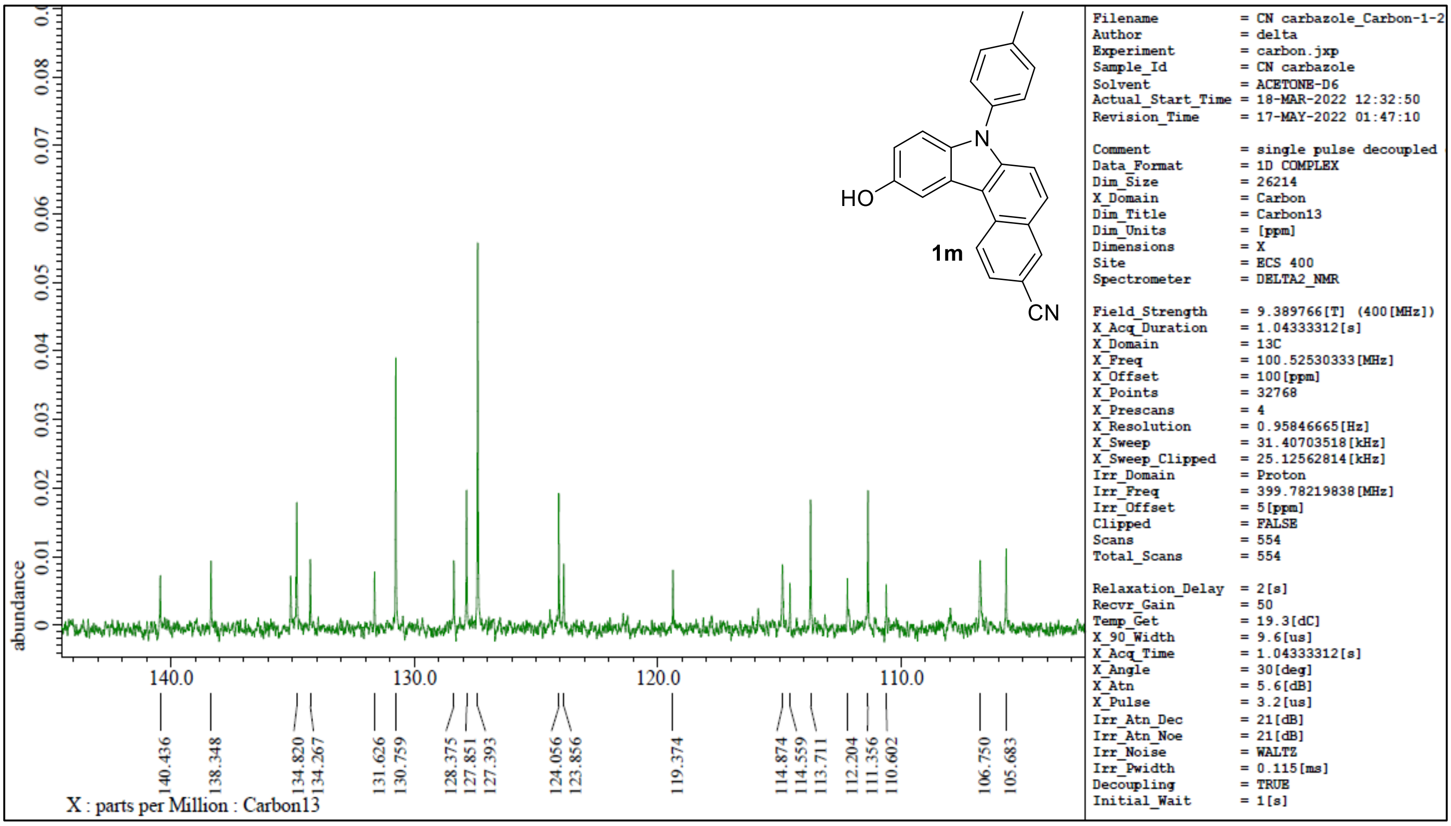

Compound **1m** (^{13}C NMR, 100 MHz, $(CD_3)_2CO$).

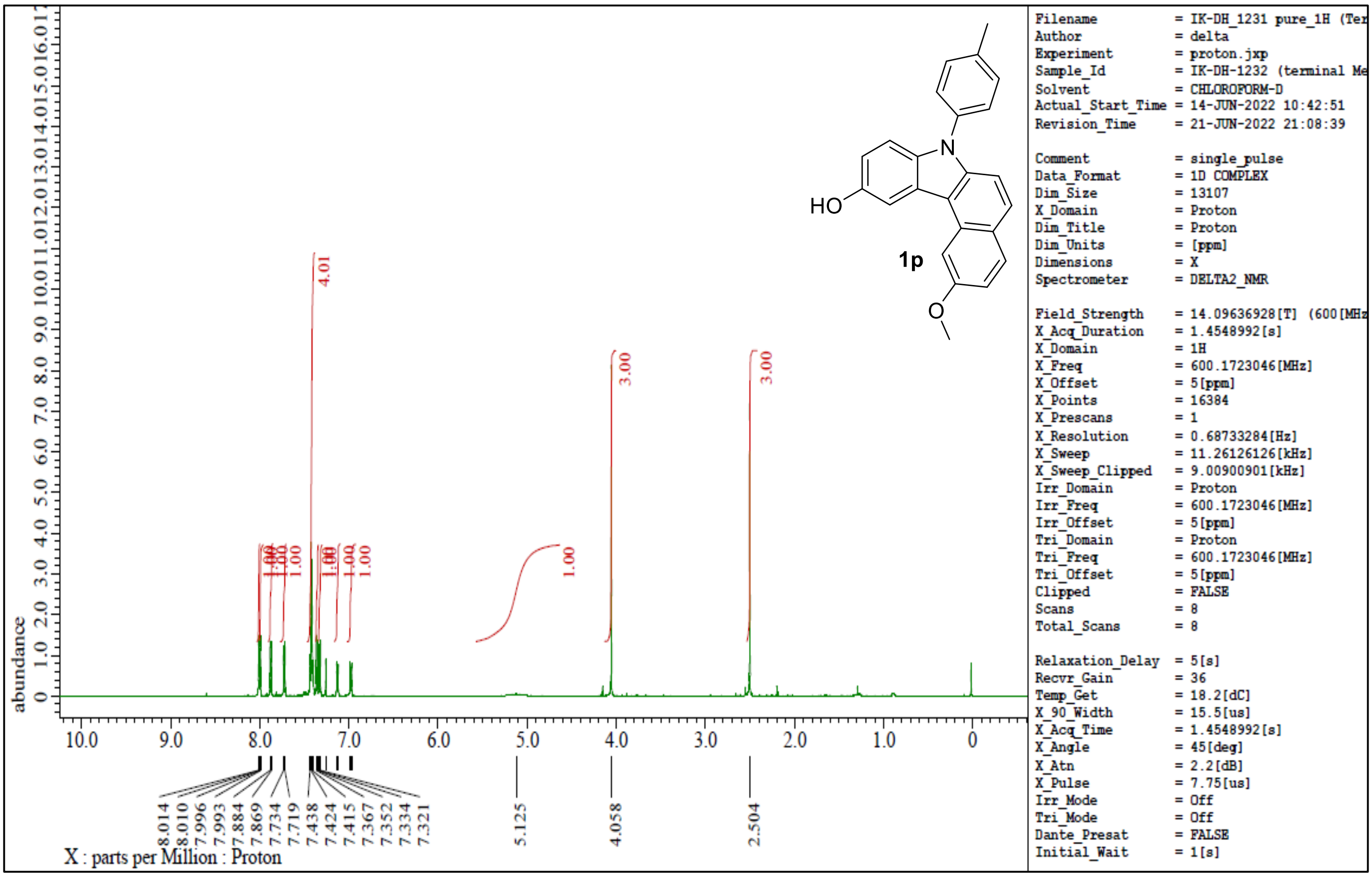

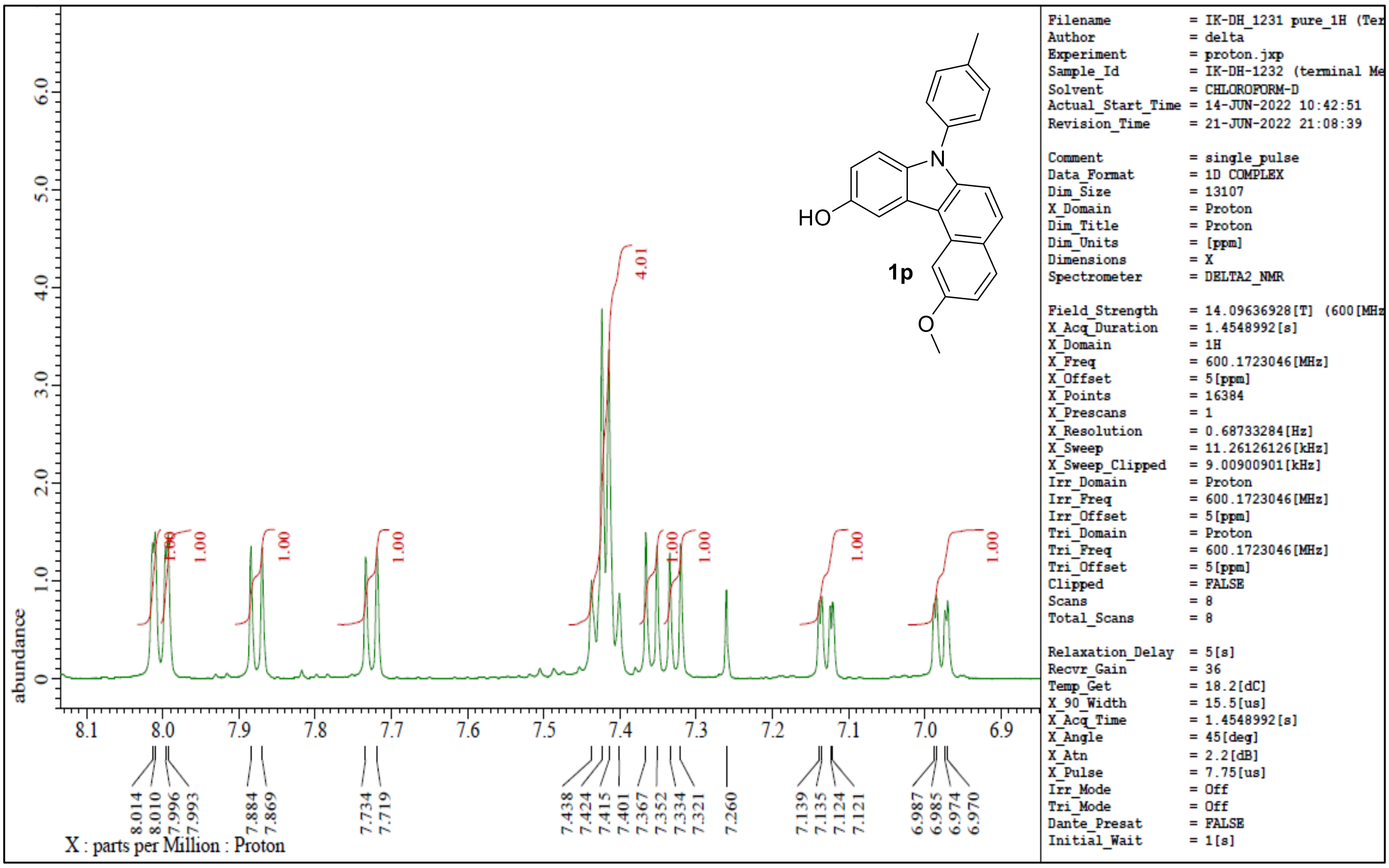

Compound **1p** (^{1}H NMR, 600 MHz, $CDCl_3$).

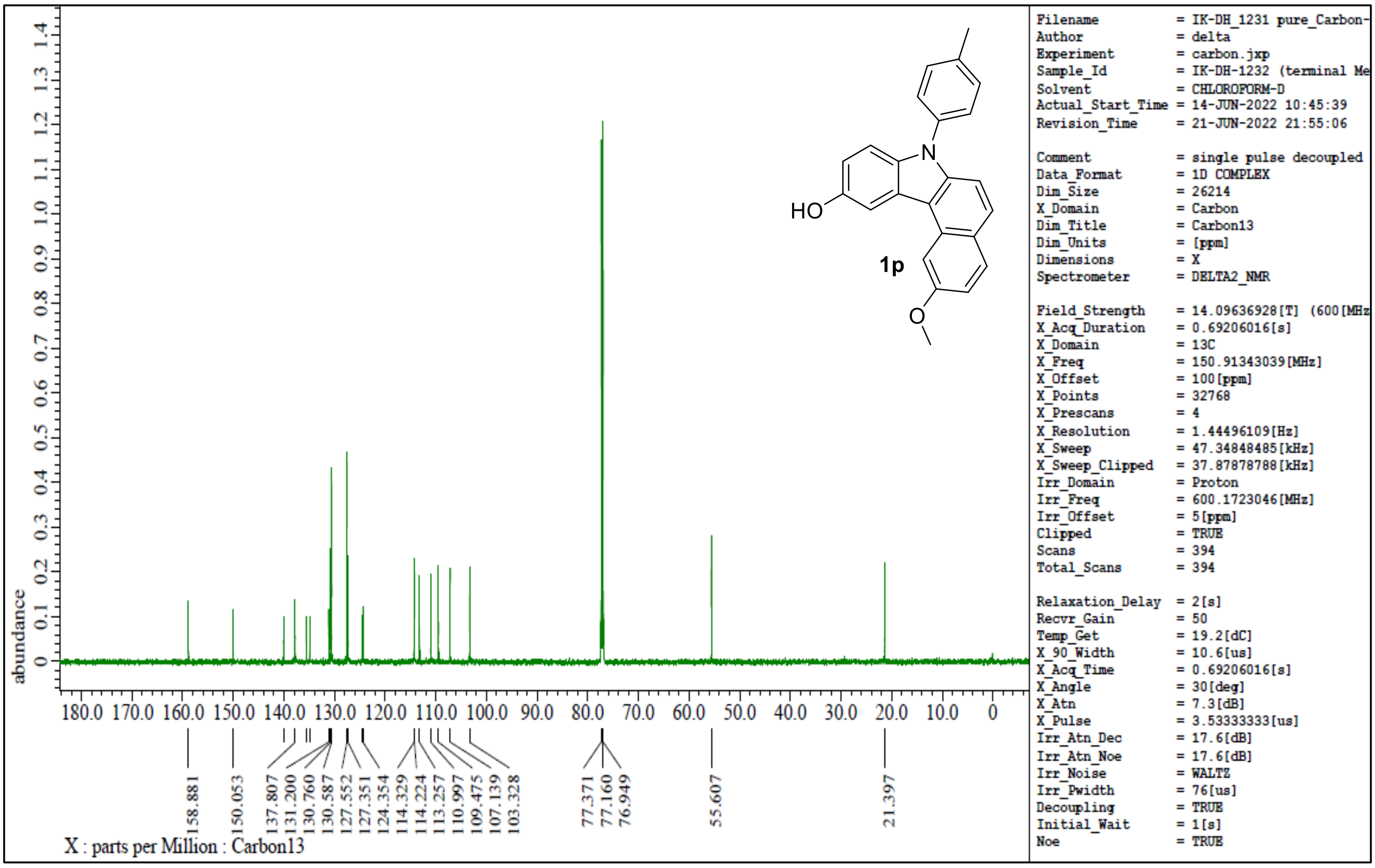

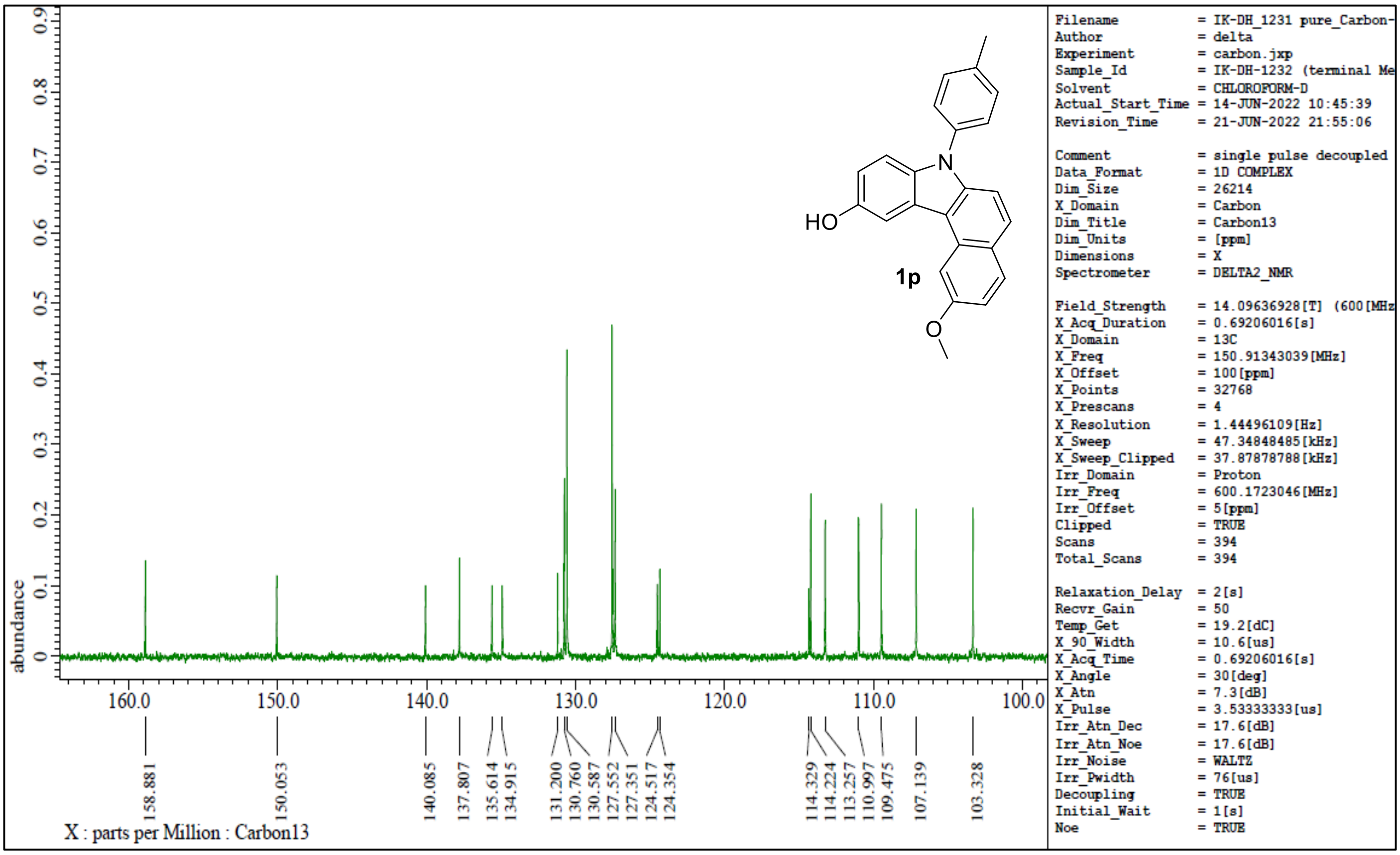

Compound **1p** (^{13}C NMR, 150 MHz, $CDCl_3$).

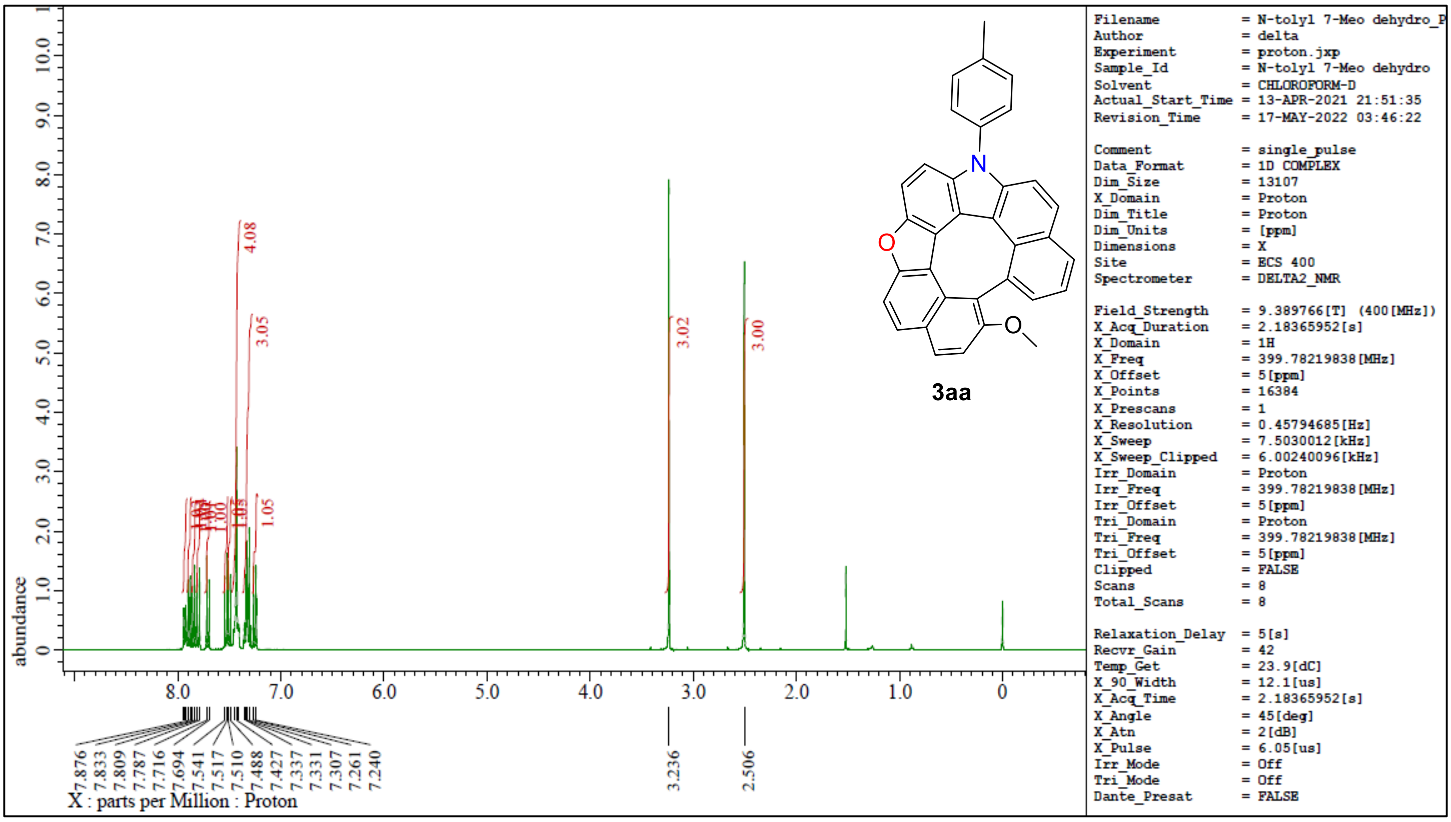

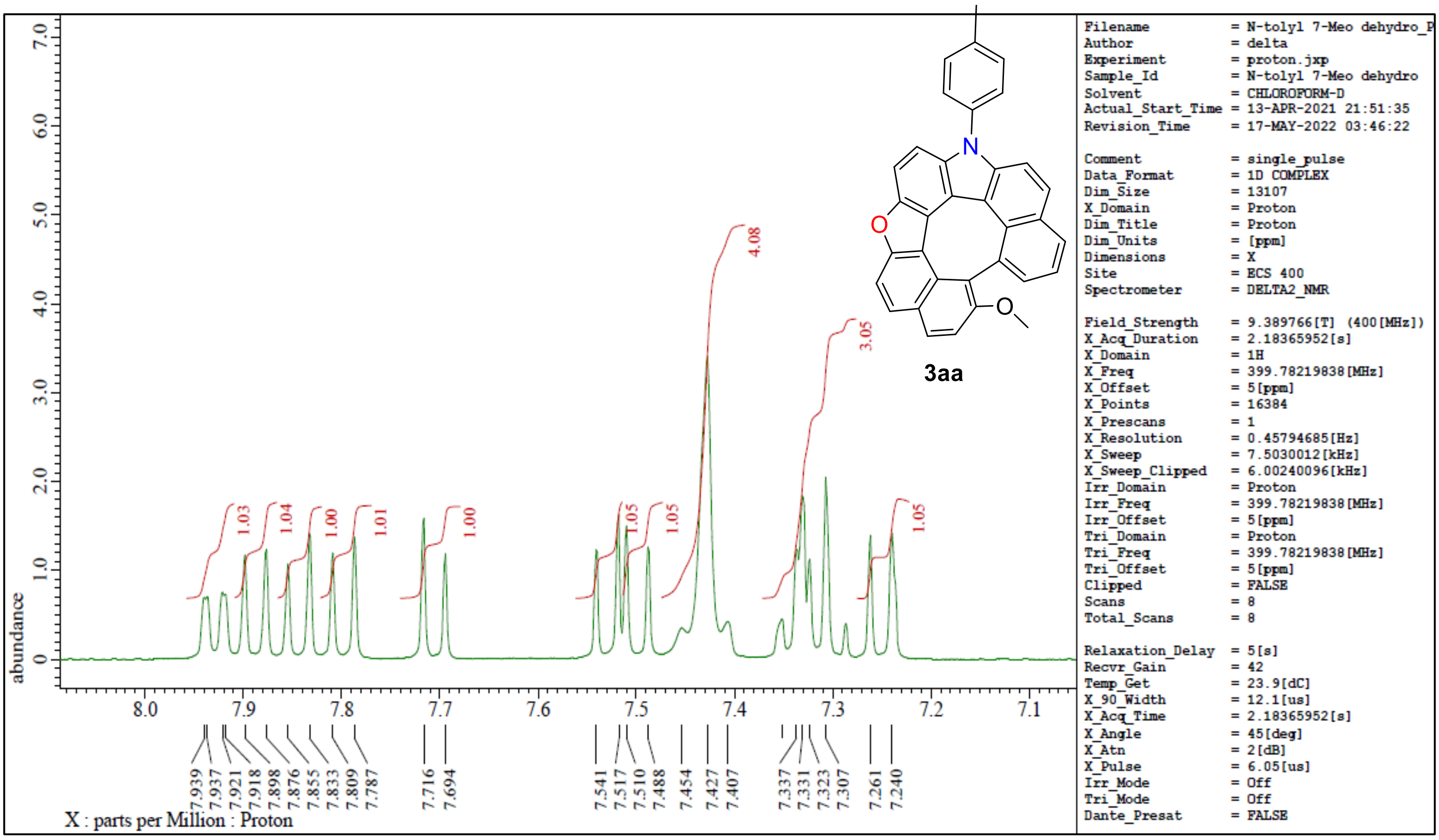

Compound **3aa** (^{1}H NMR, 400 MHz, $CDCl_3$).

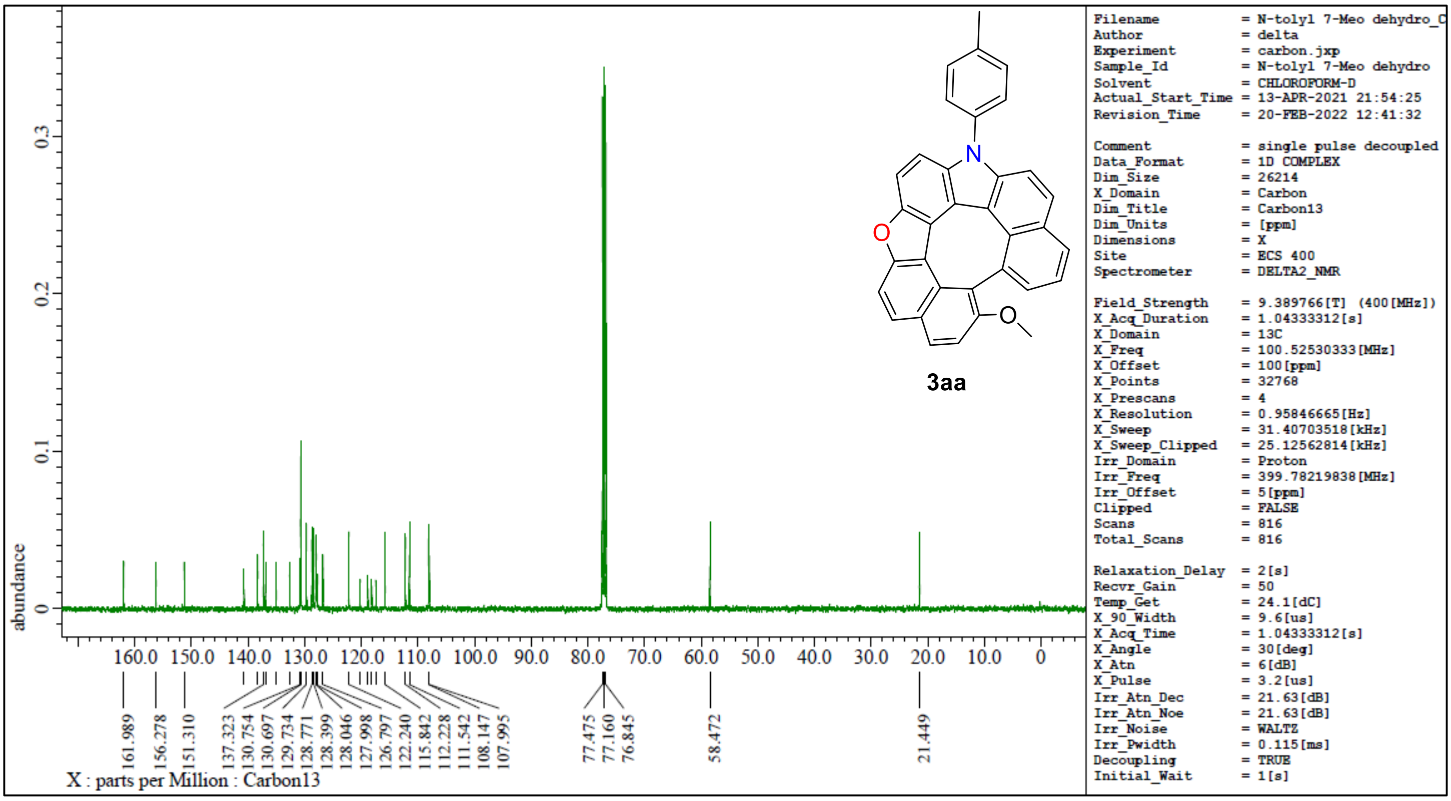

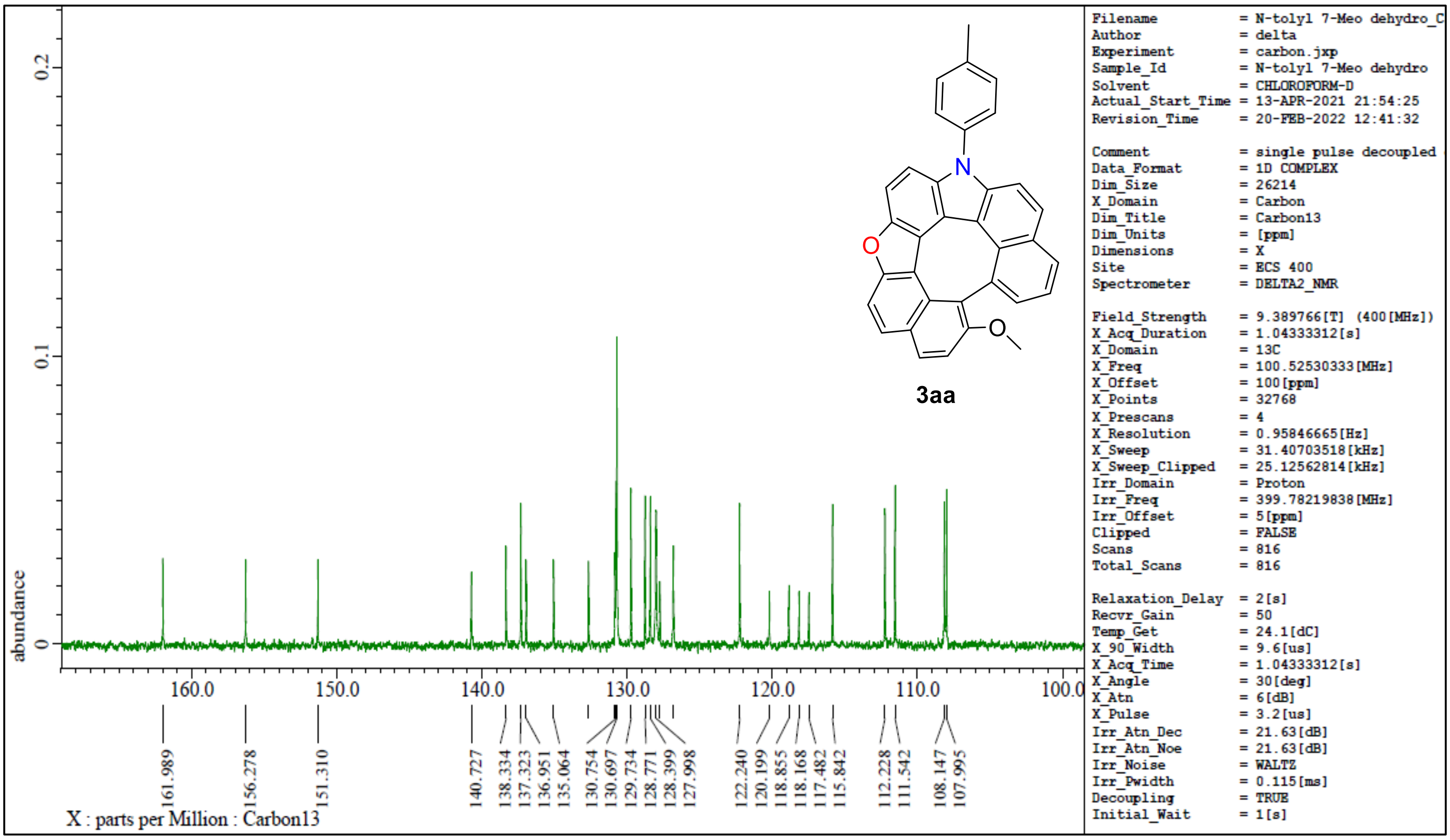

Compound **3aa** (^{13}C NMR, 100 MHz, $CDCl_3$).

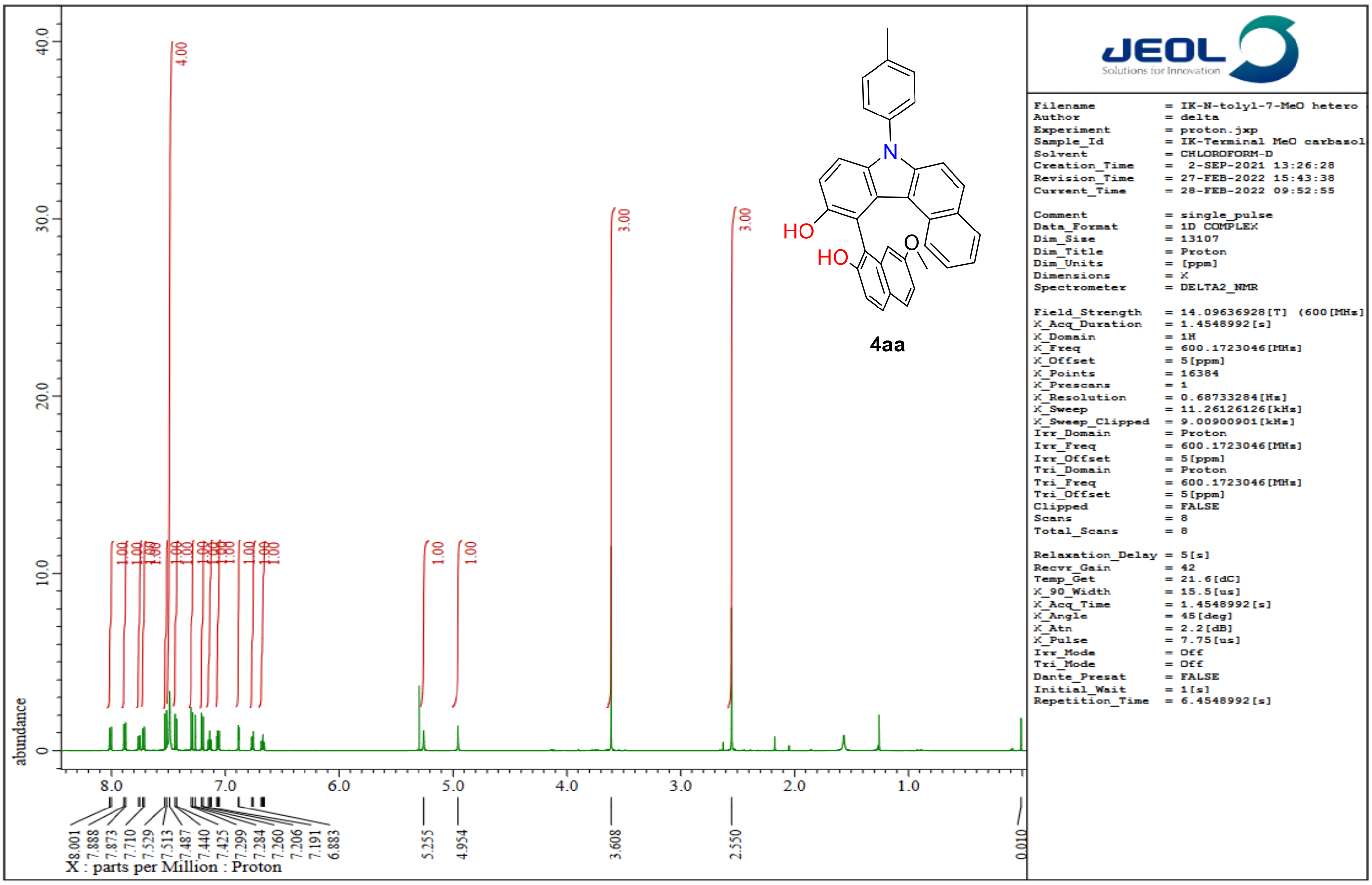

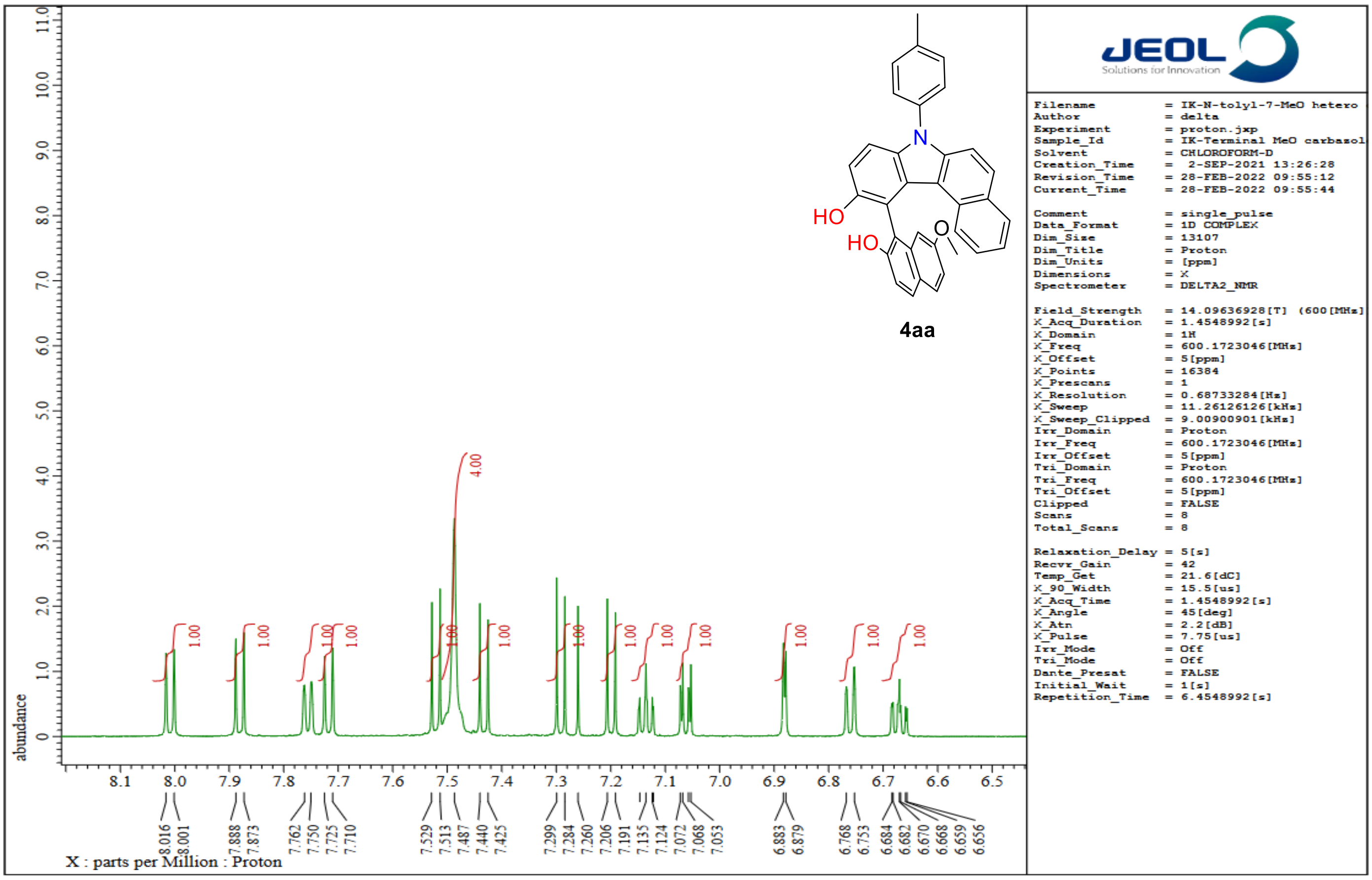

Compound **4aa** (^{1}H NMR, 600 MHz, $CDCl_3$).

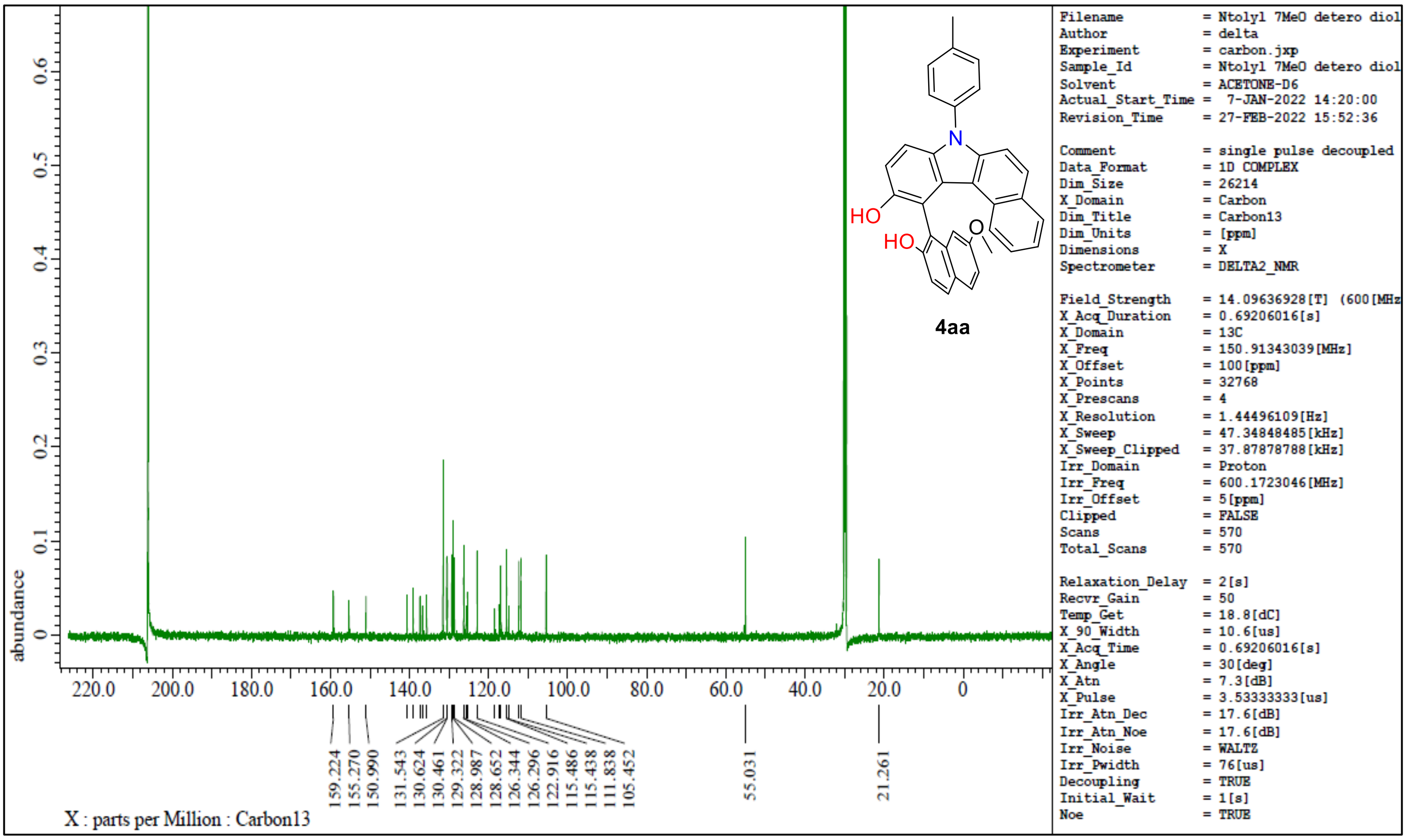

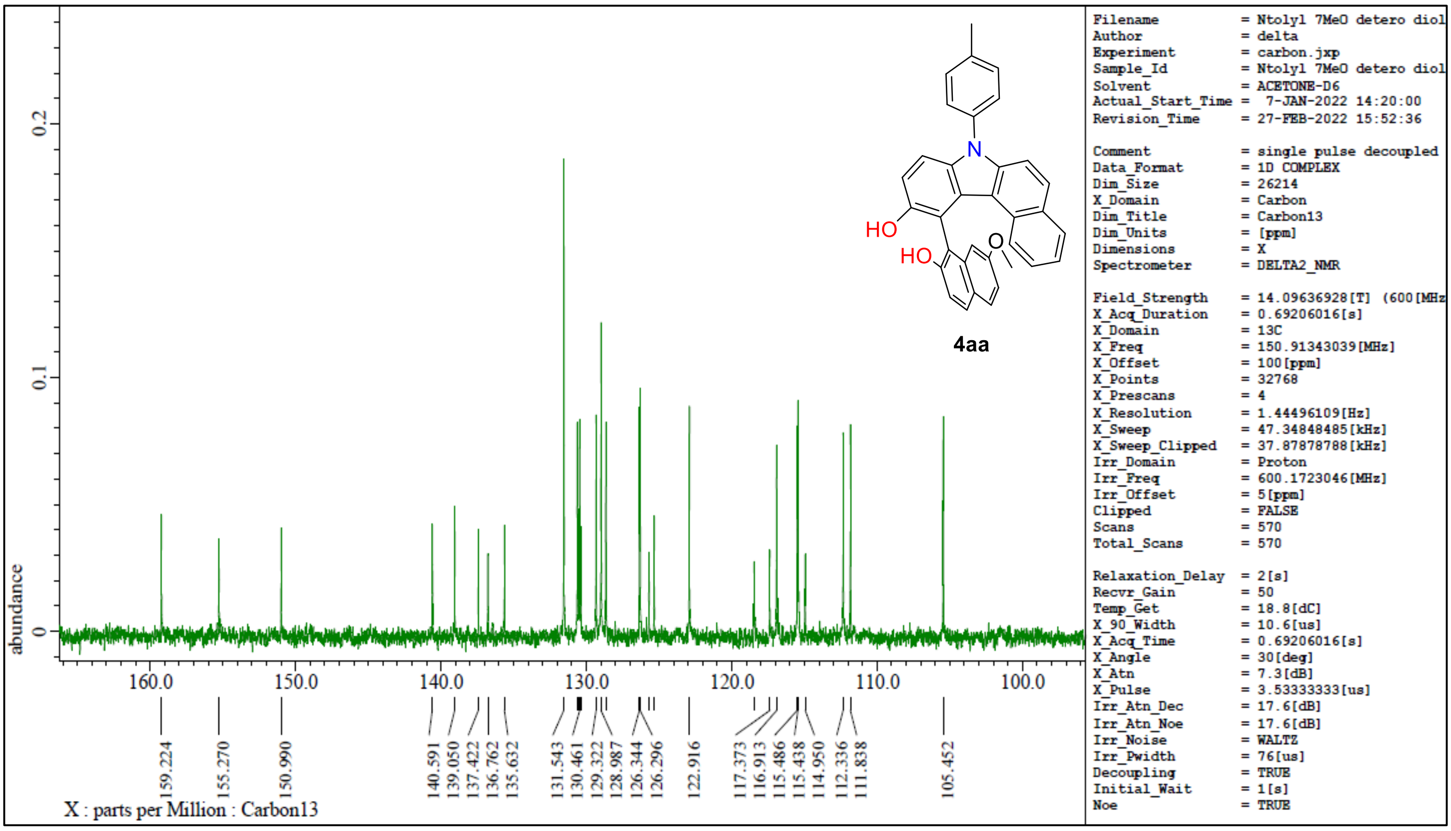

Compound **4aa** (^{13}C NMR, 100 MHz, $(CD_3)_2CO$).

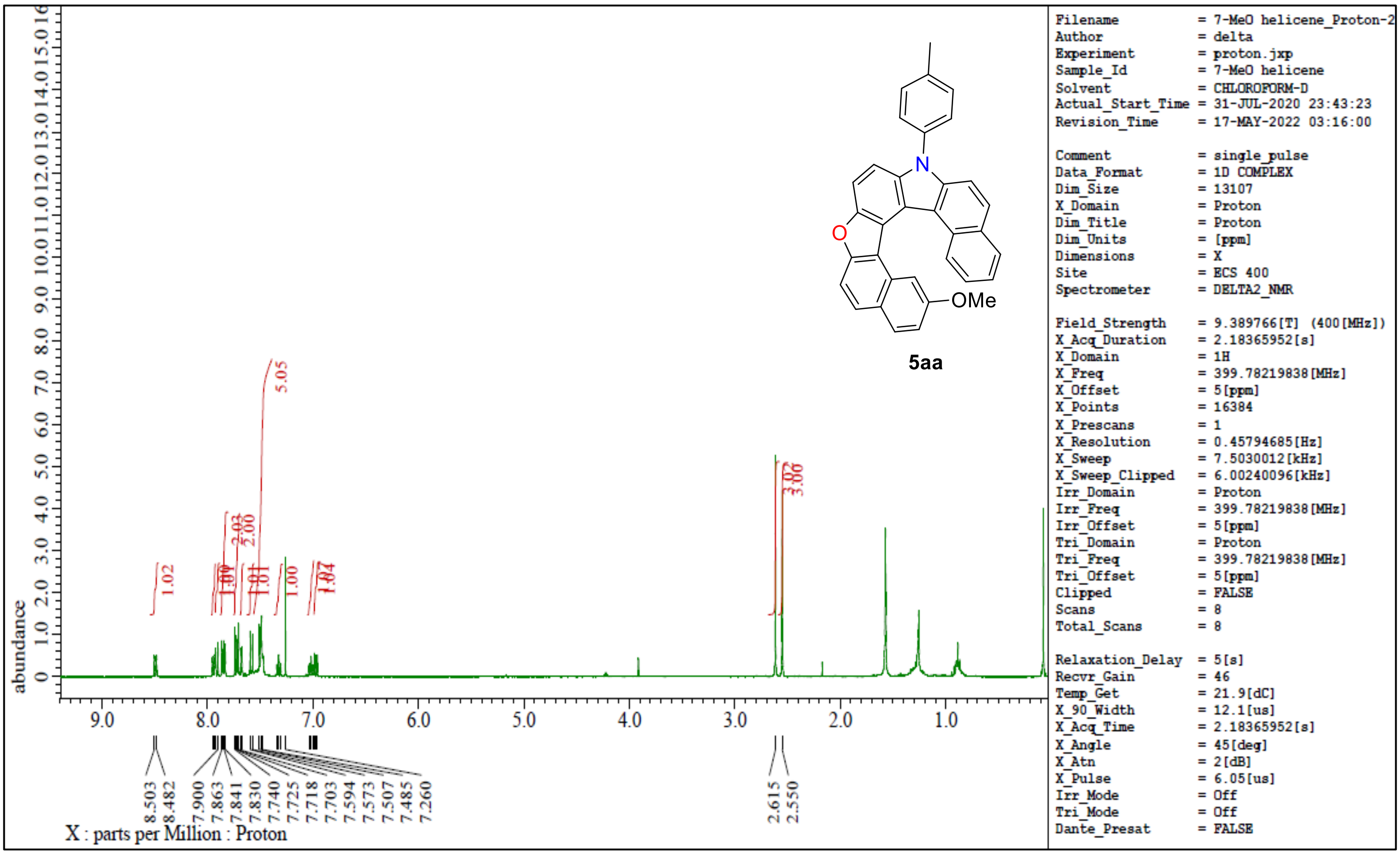

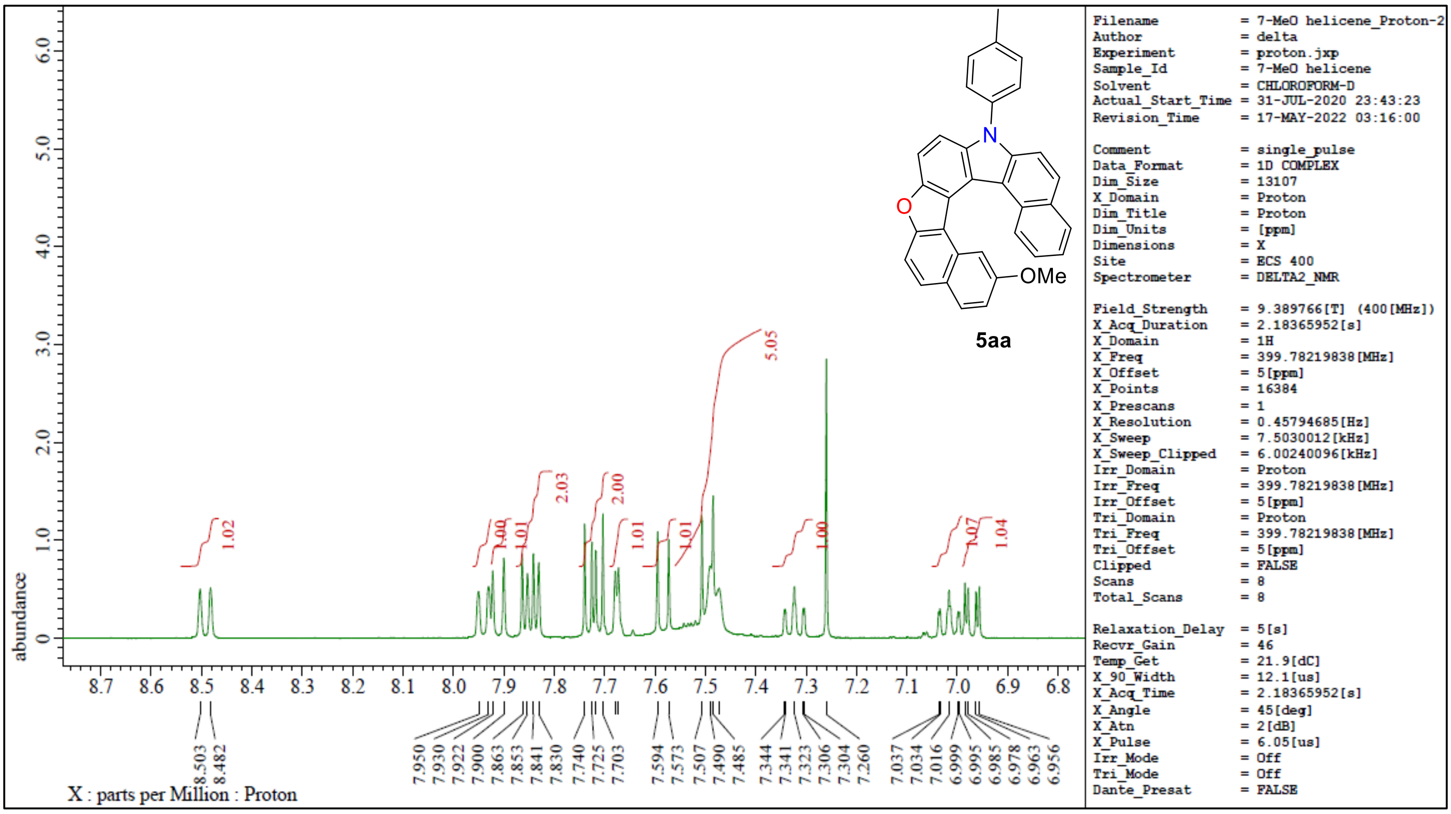

Compound **5aa** (^{1}H NMR, 400 MHz, $CDCl_3$).

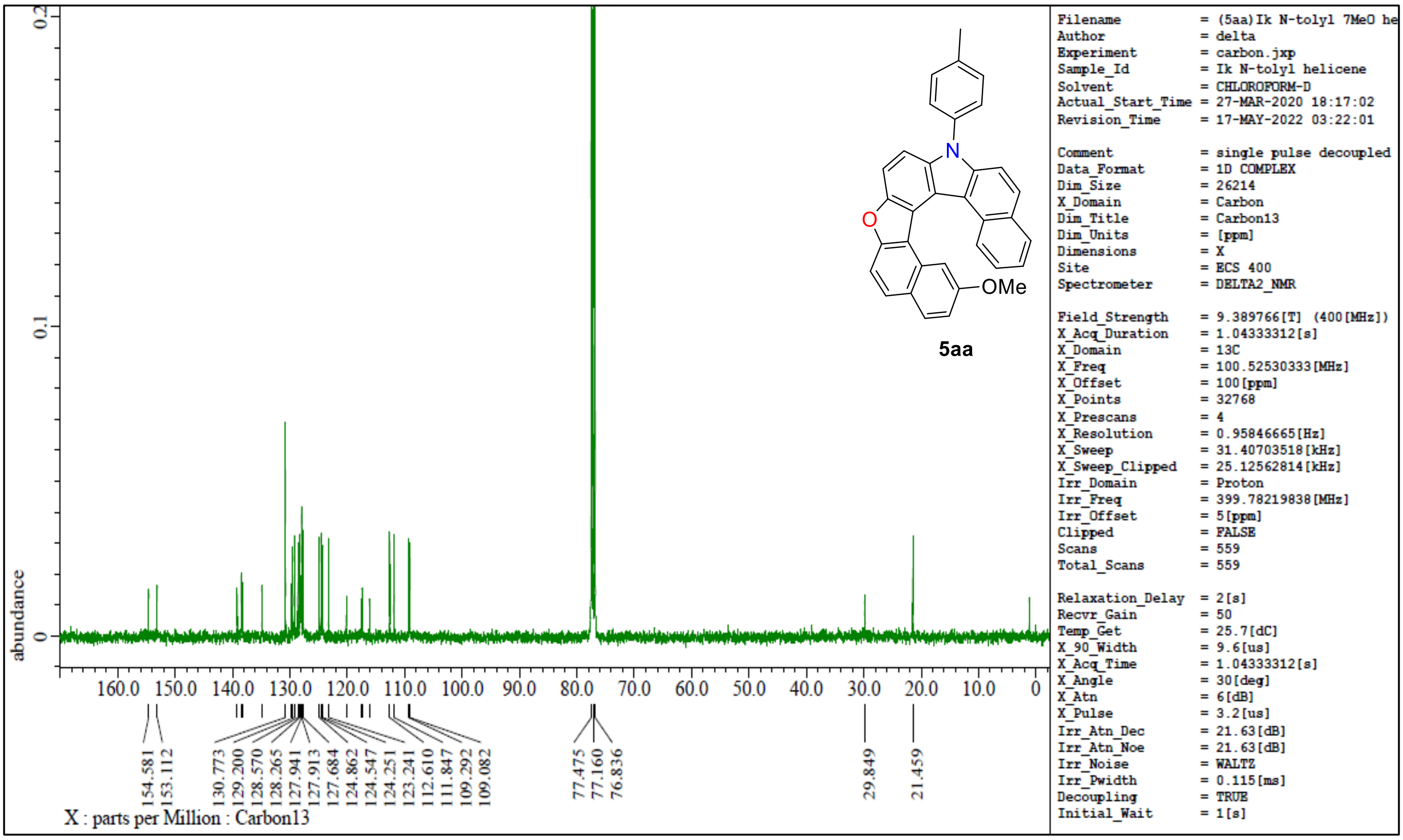

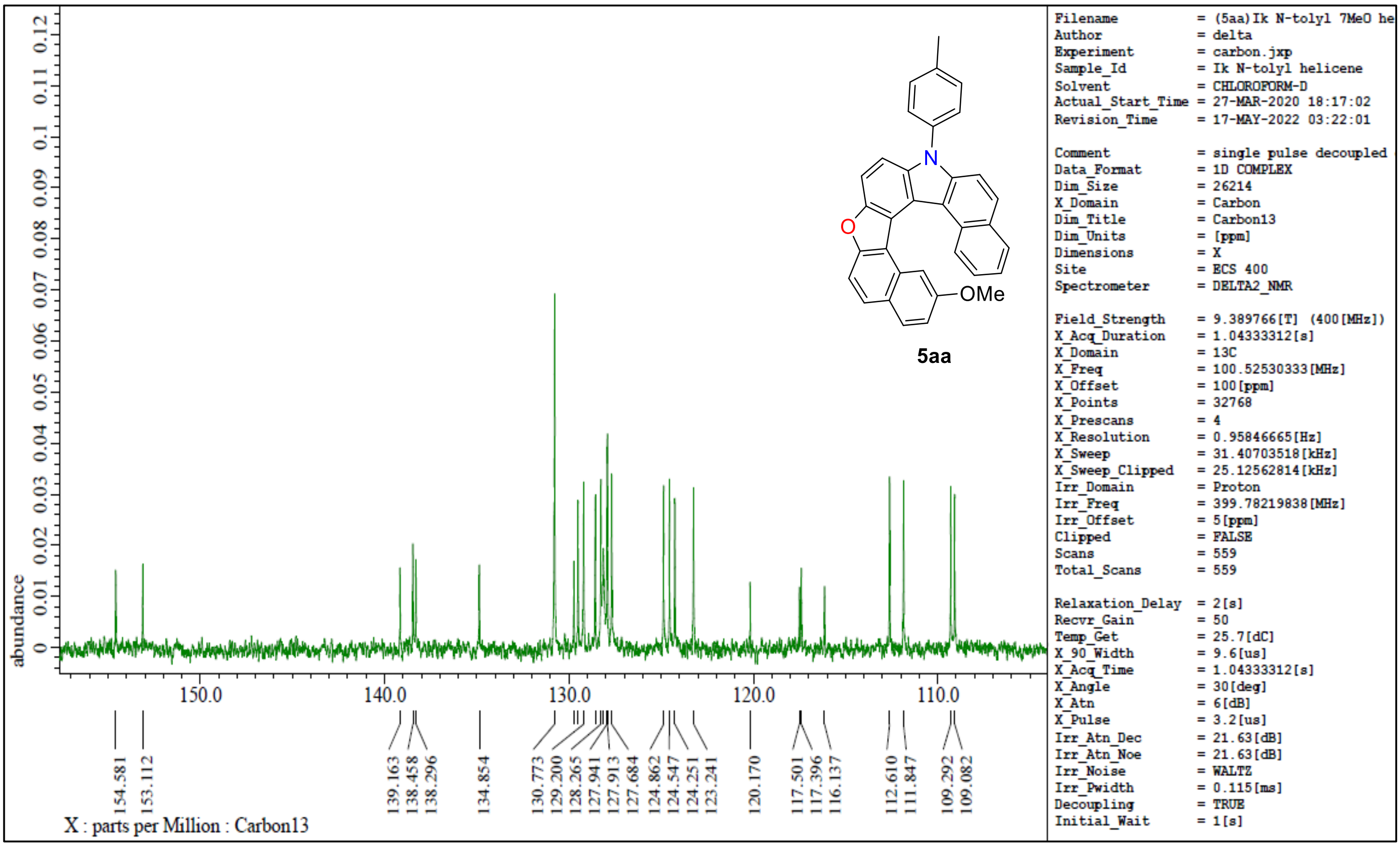

Compound **5aa** (^{13}C NMR, 100 MHz, $CDCl_3$).

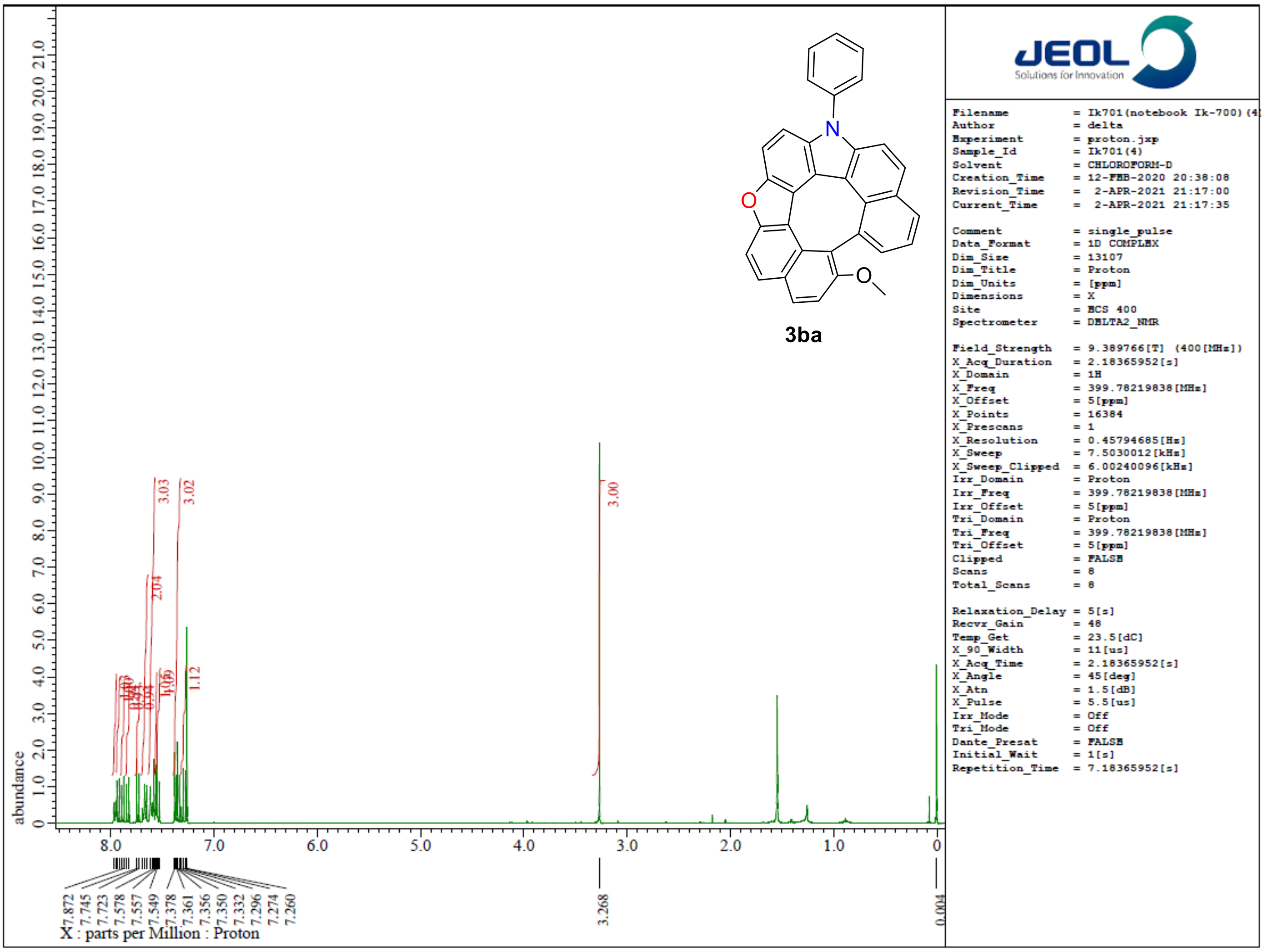

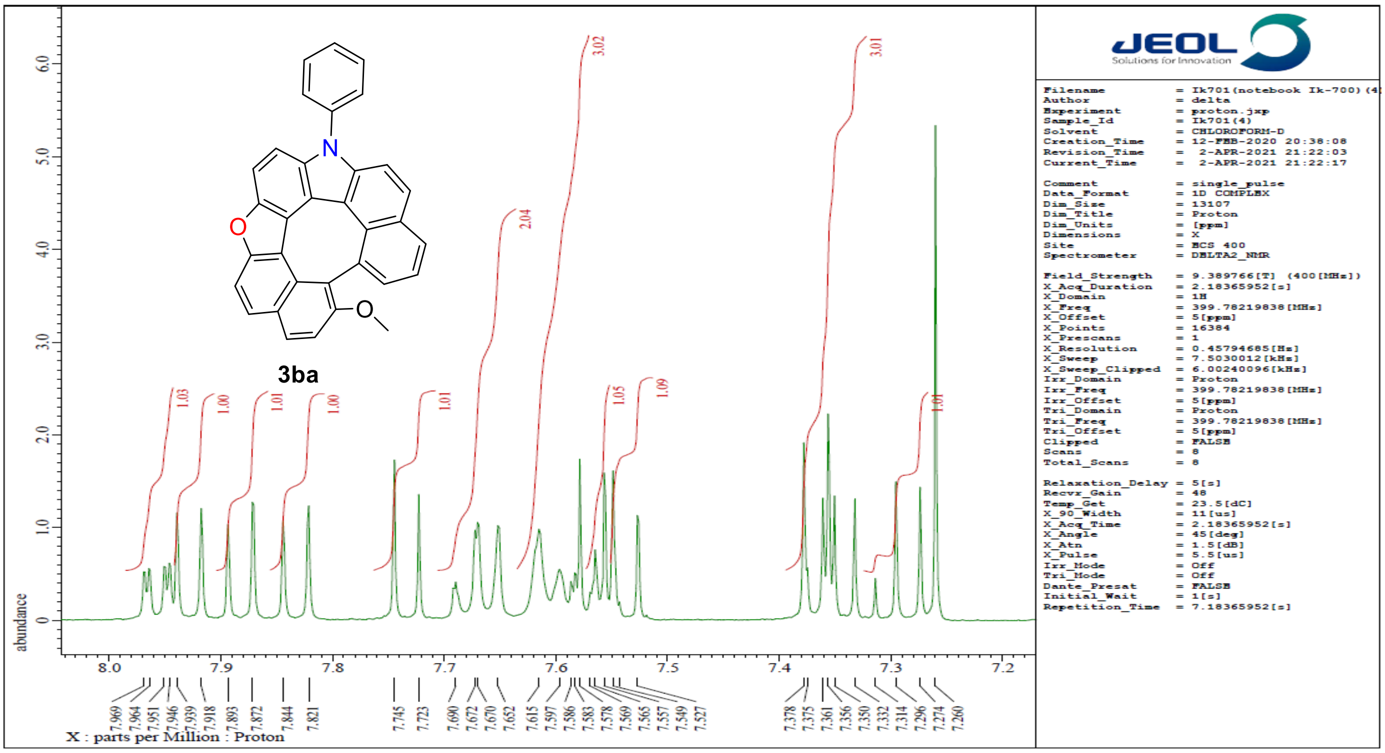

Compound **3ba** (^{1}H NMR, 400 MHz, $CDCl_3$).

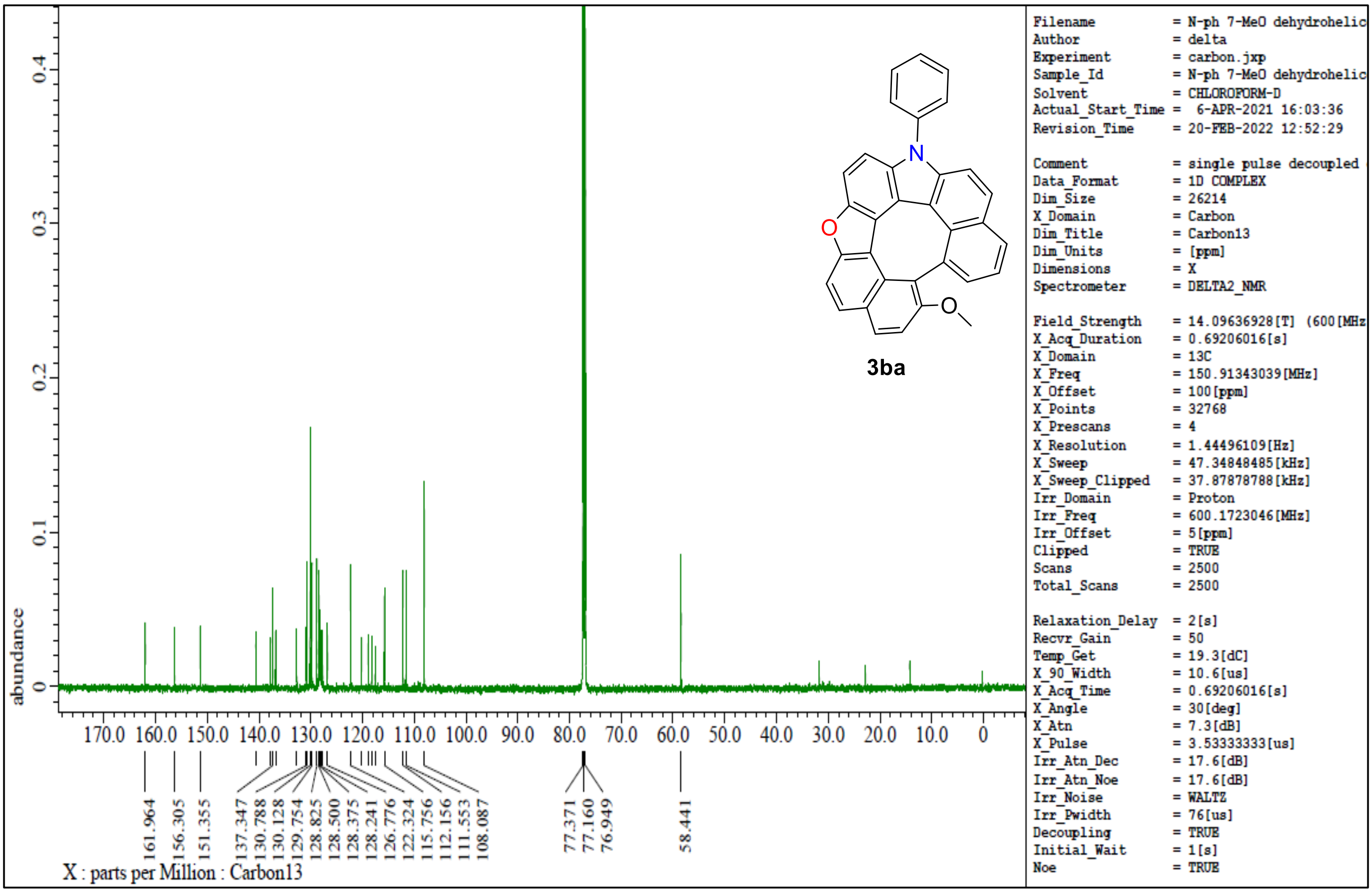

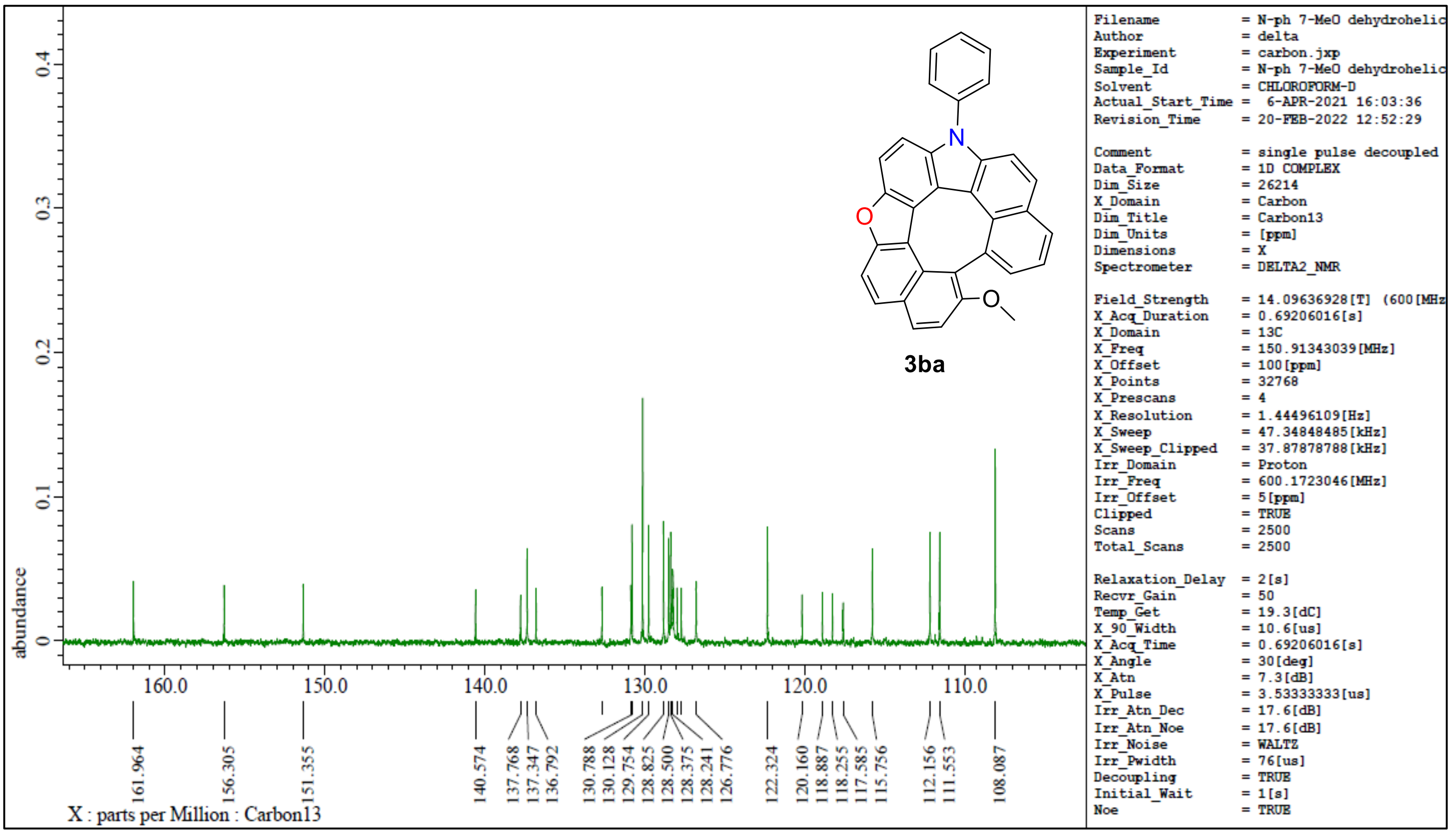

Compound **3ba** (^{13}C NMR, 150 MHz, $CDCl_3$).

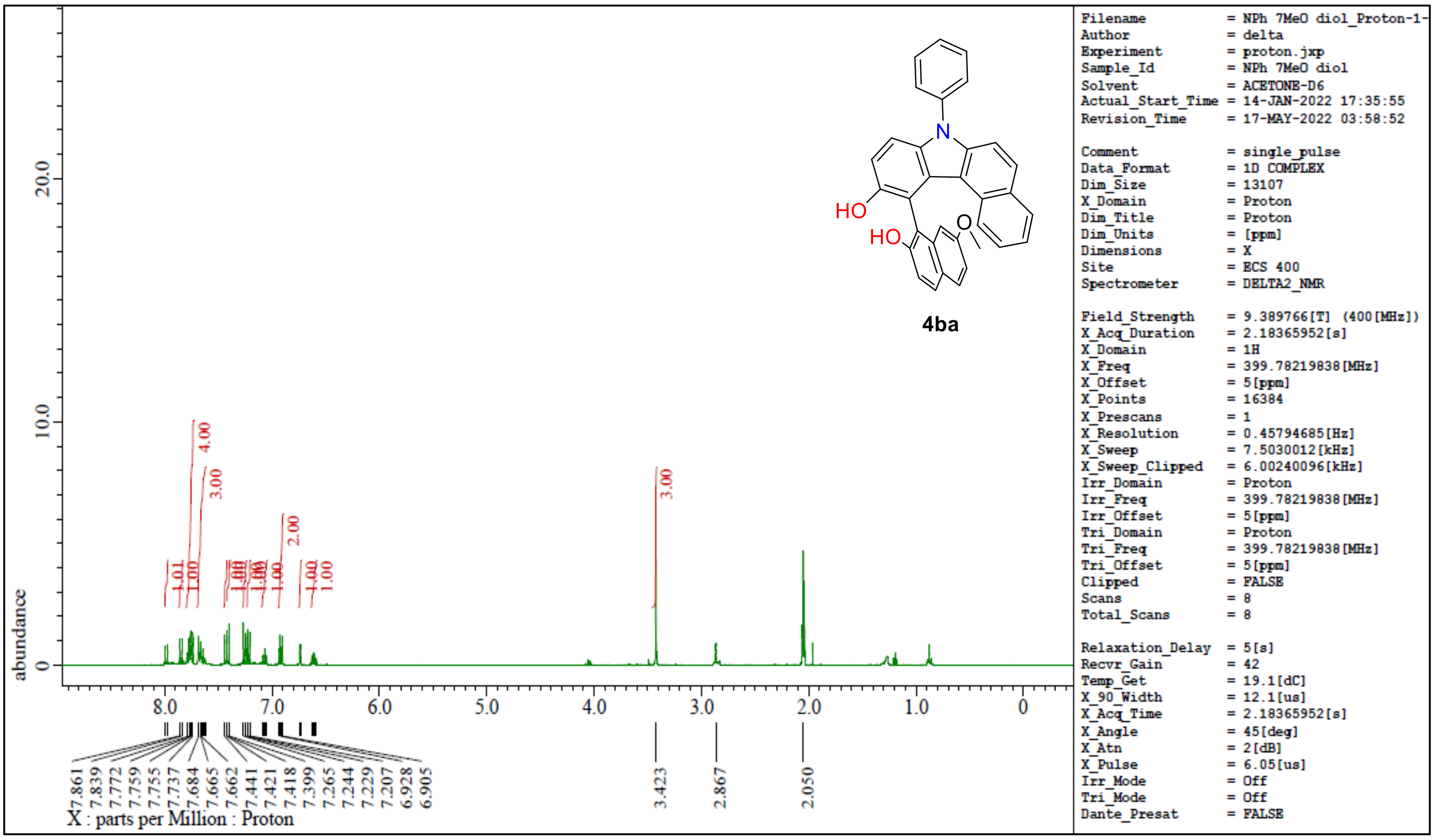

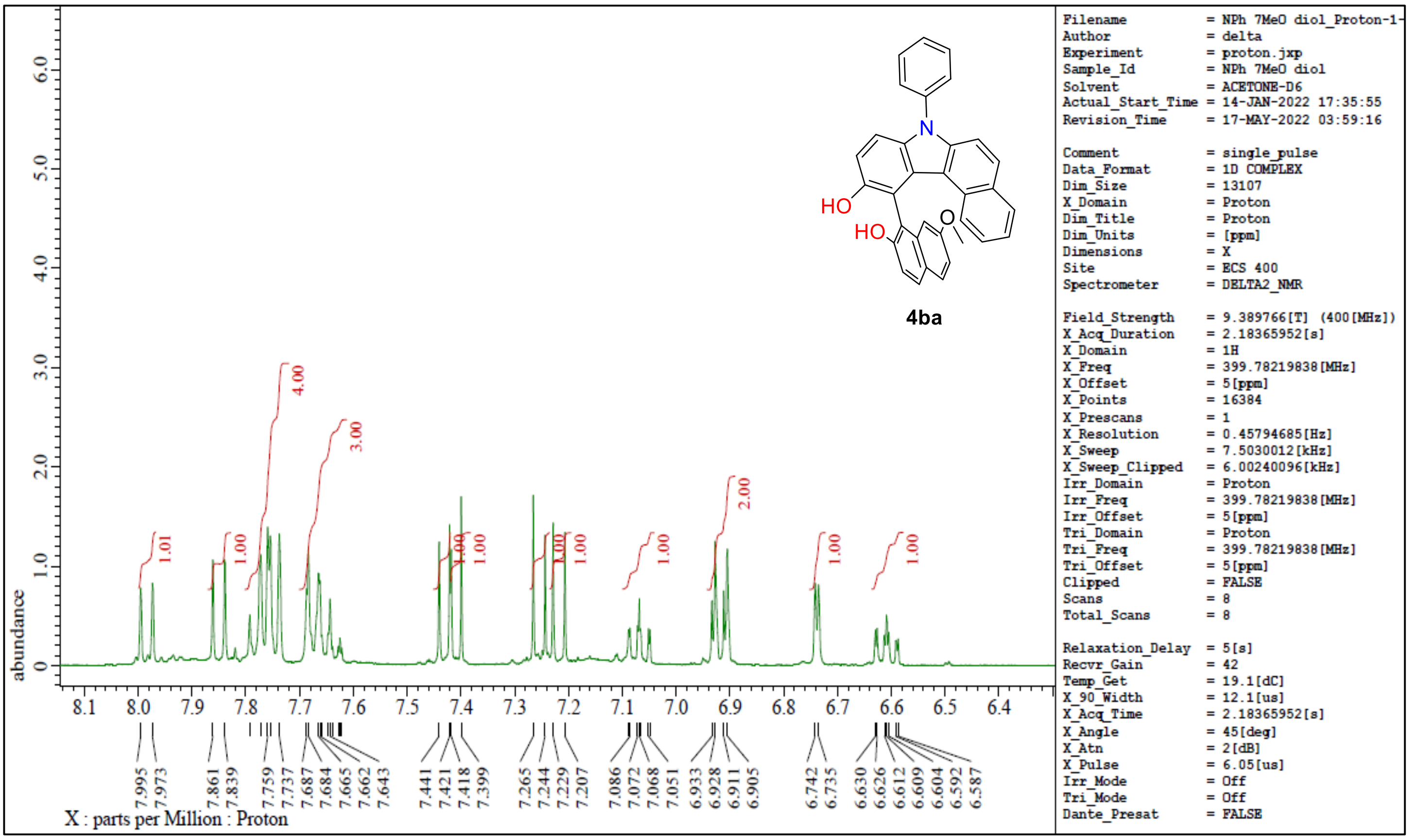

Compound **4ba** (^{1}H NMR, 400 MHz, $(CD_3)_2CO$).

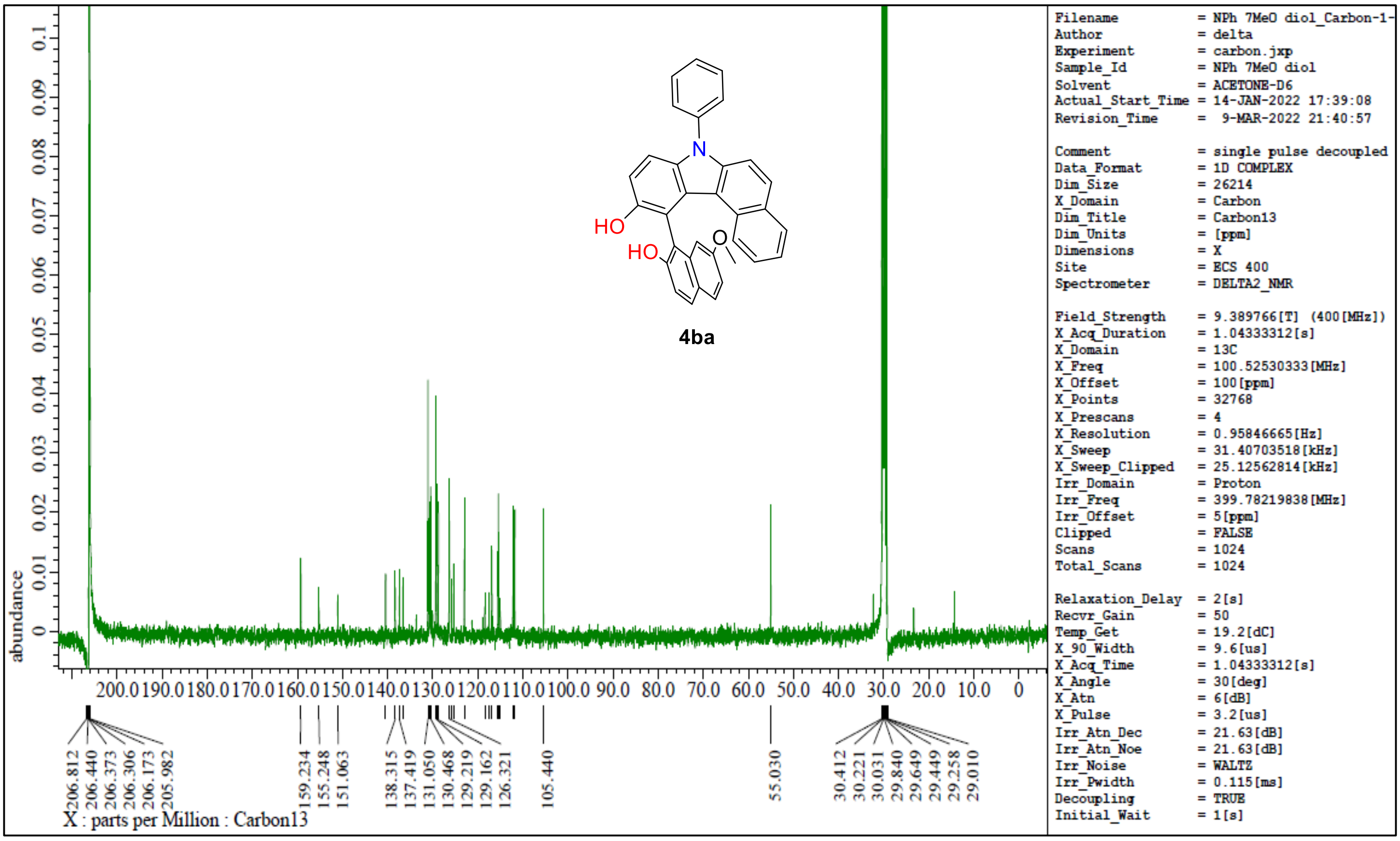

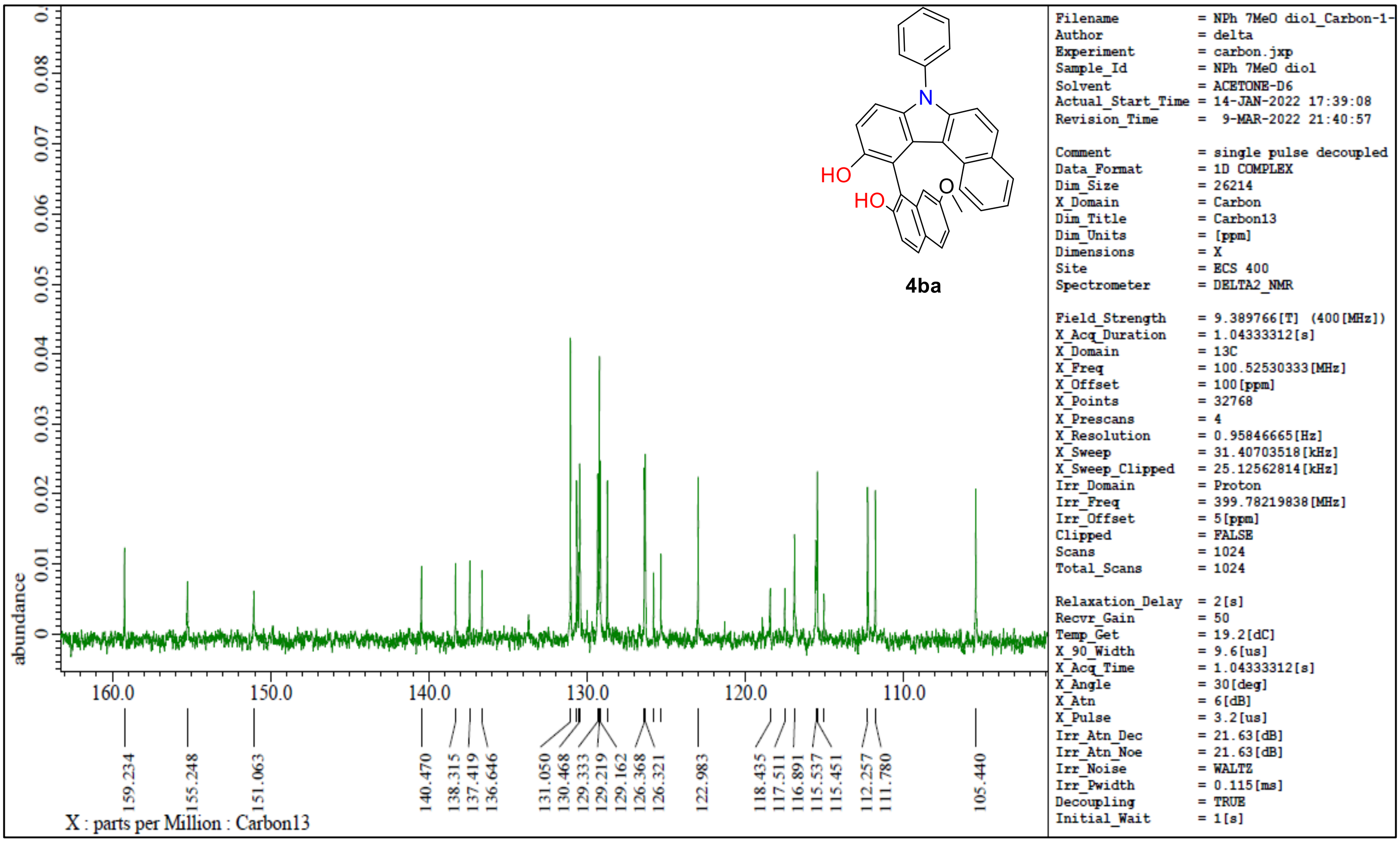

Compound **4ba** (^{13}C NMR, 100 MHz, $(CD_3)_2CO$).

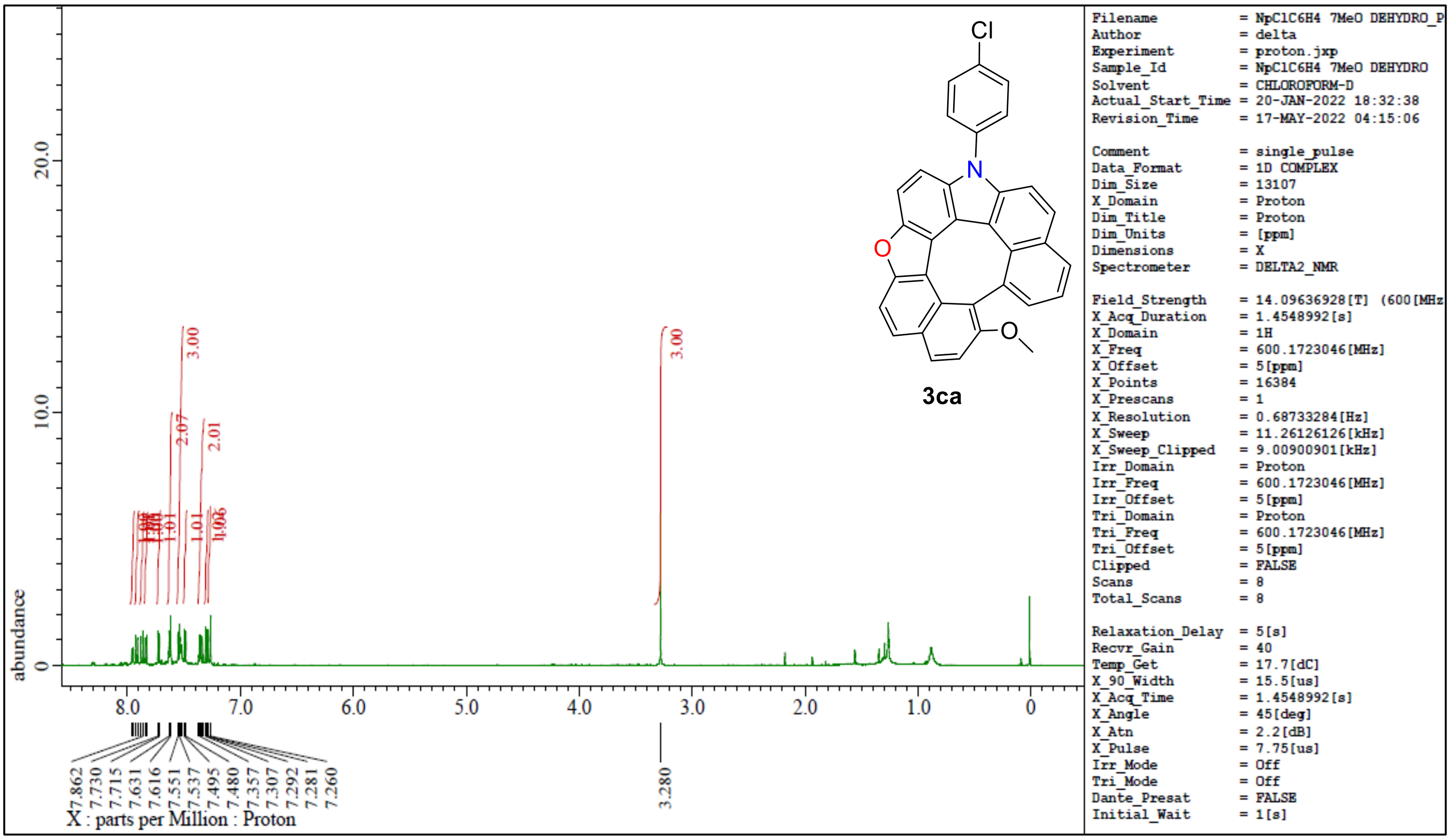

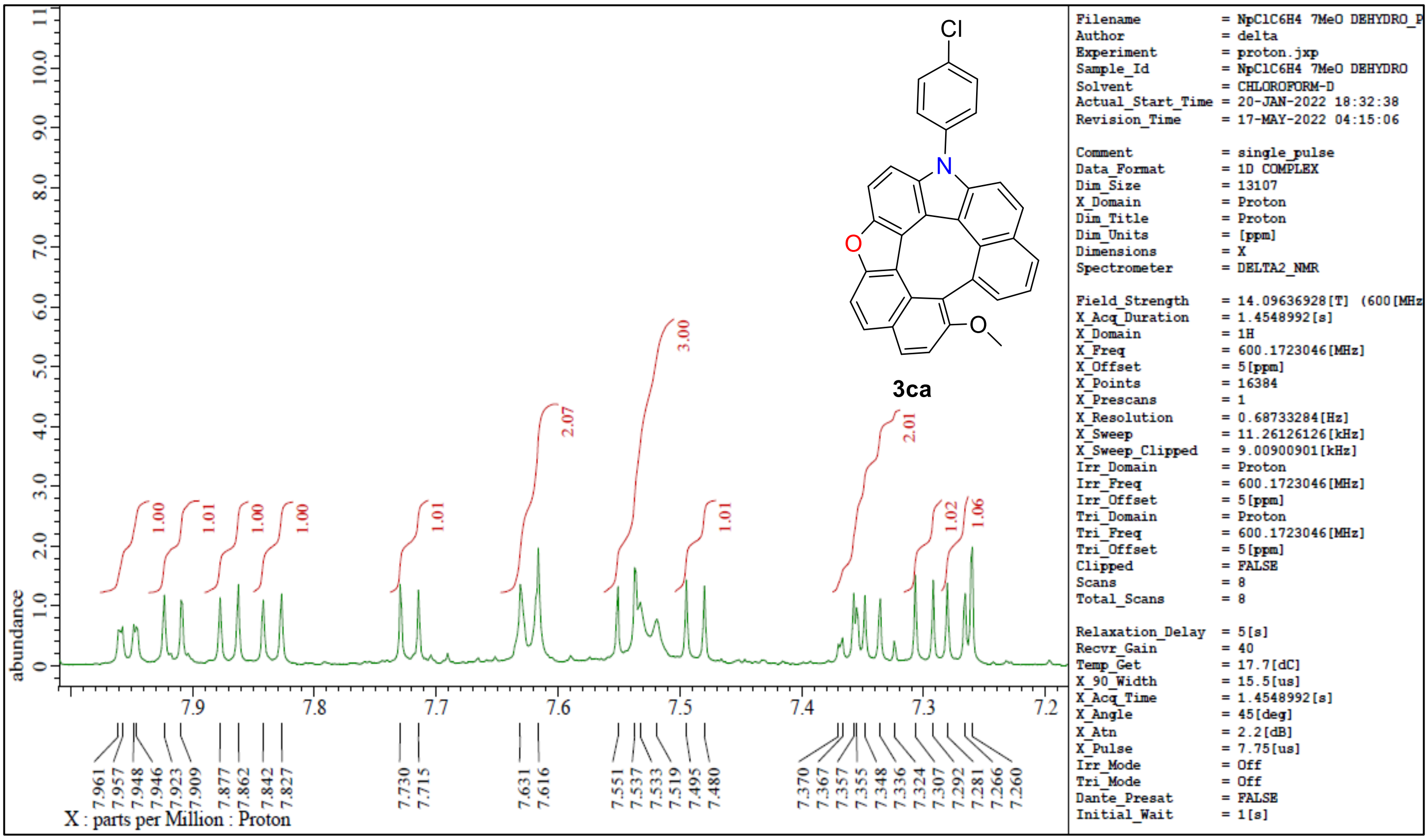

Compound **3ca** (^{1}H NMR, 600 MHz, $CDCl_3$).

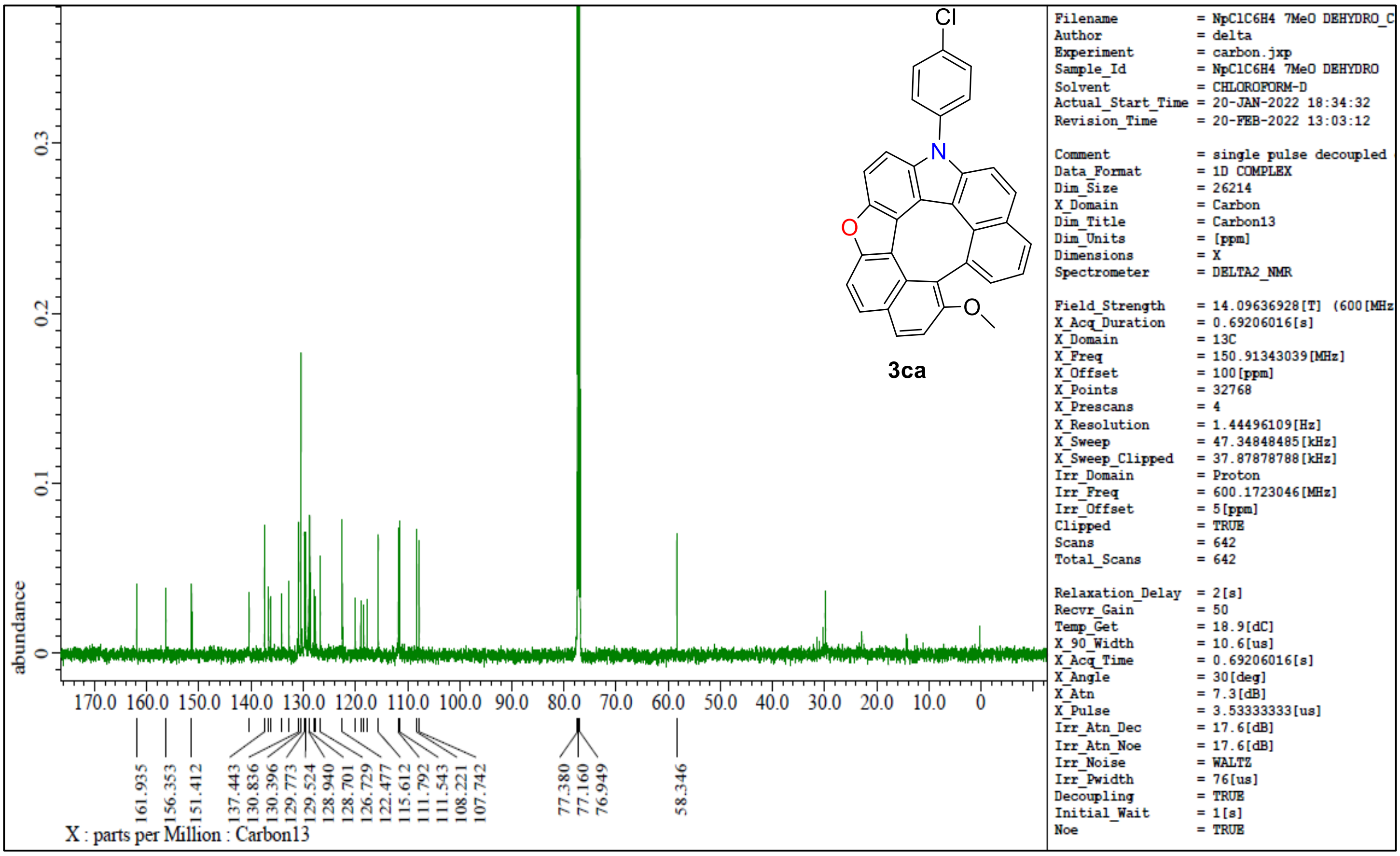

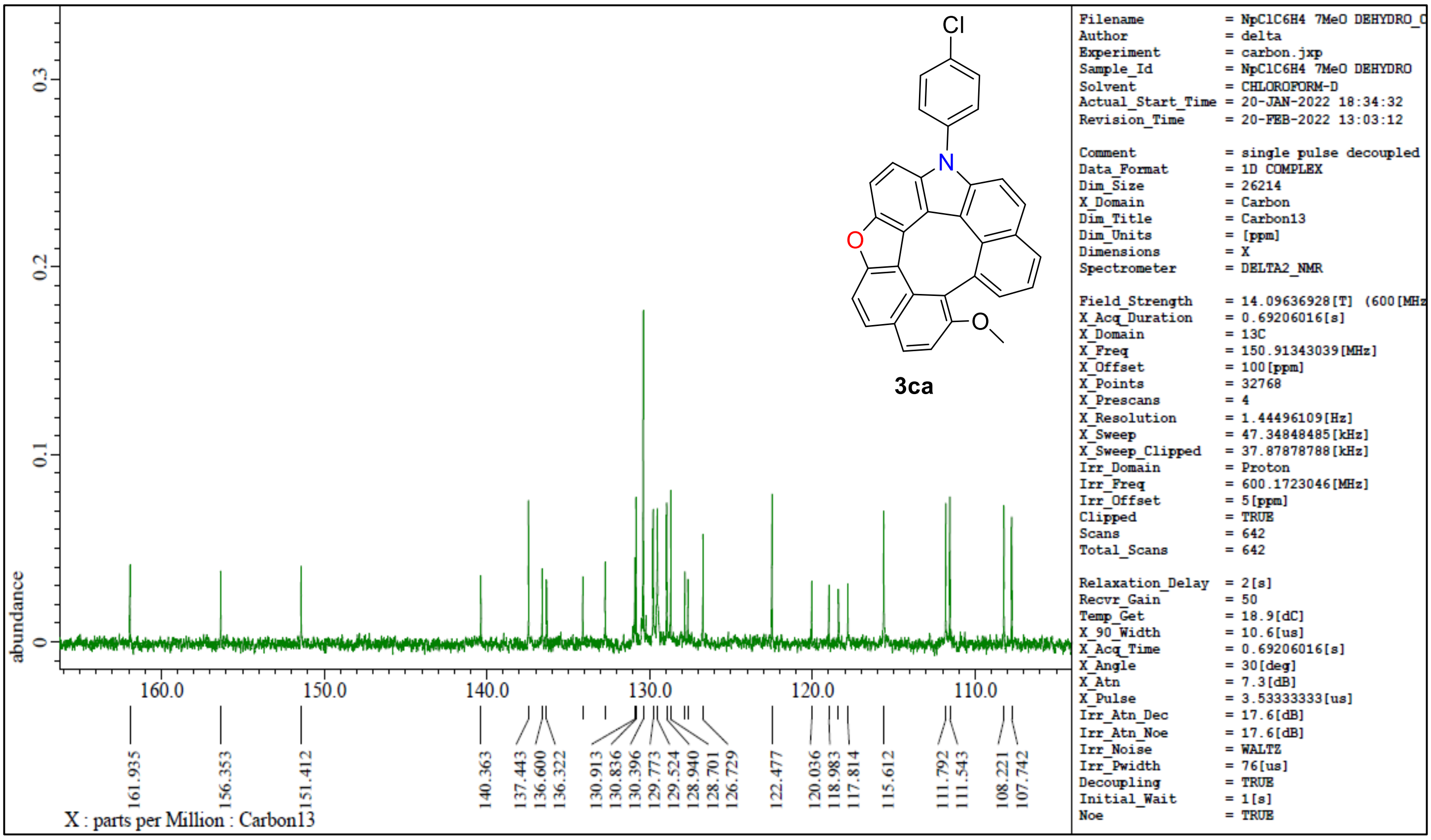

Compound **3ca** (^{13}C NMR, 150 MHz, $CDCl_3$).

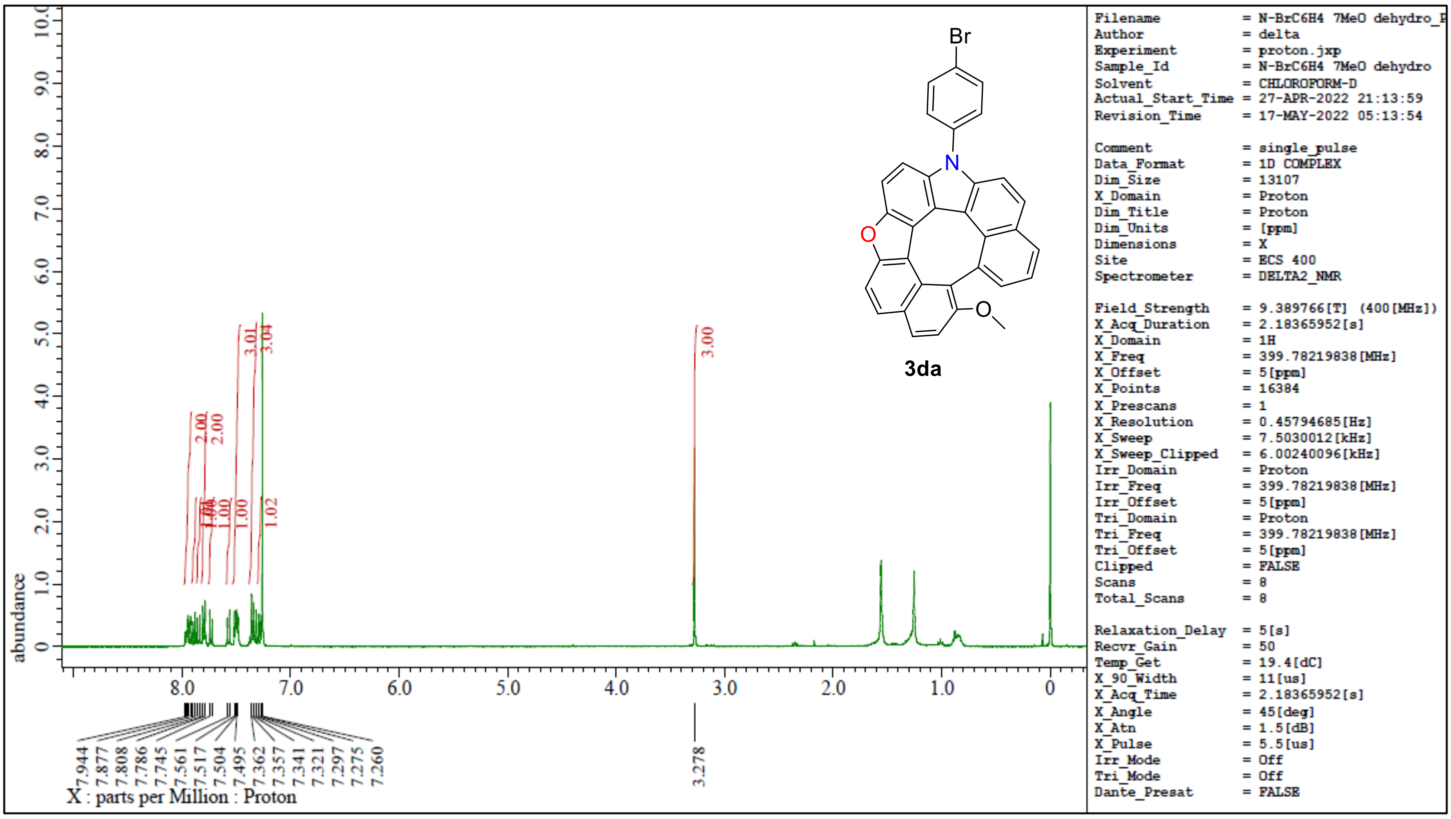

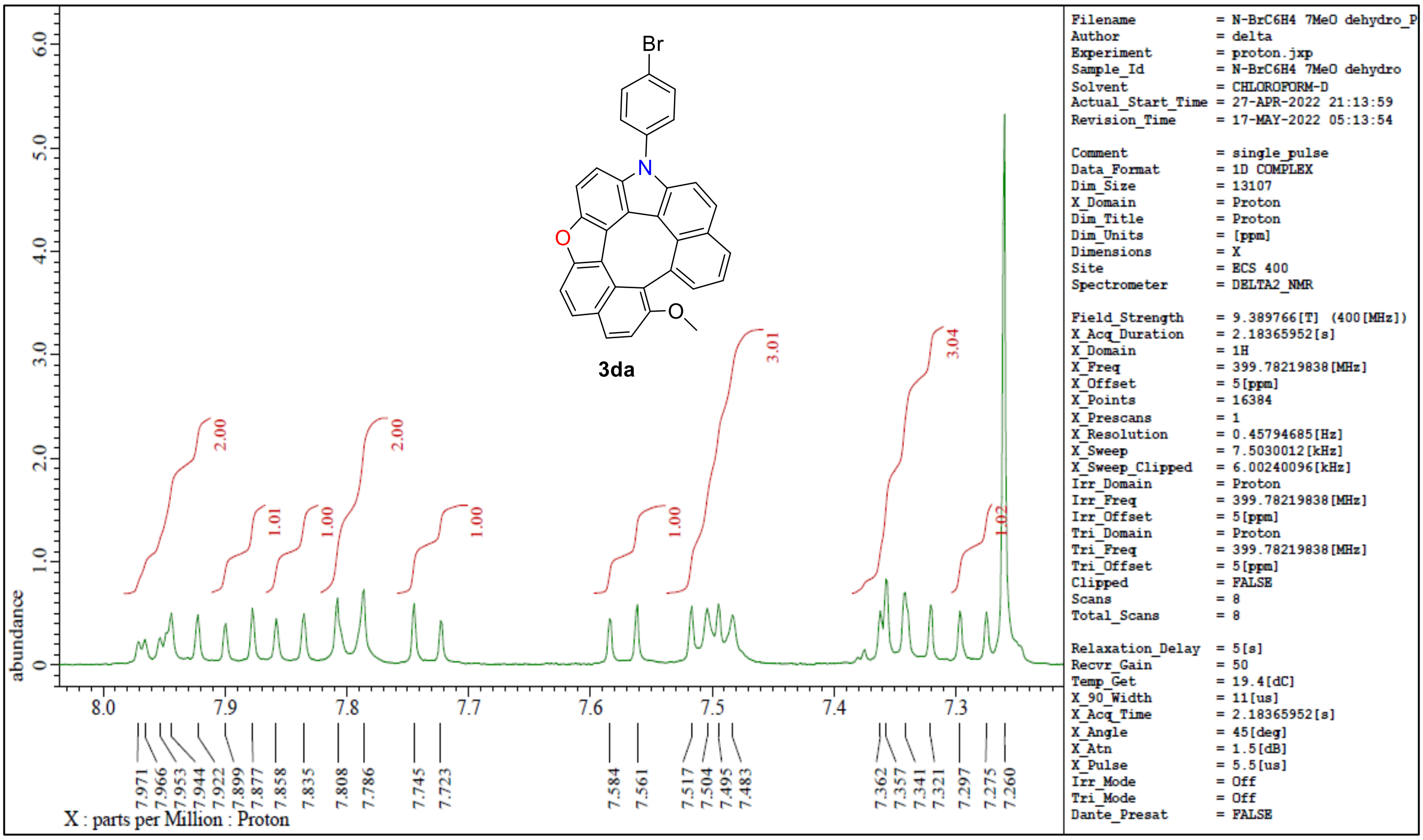

Compound **3da** (^{1}H NMR, 400 MHz, $CDCl_3$).

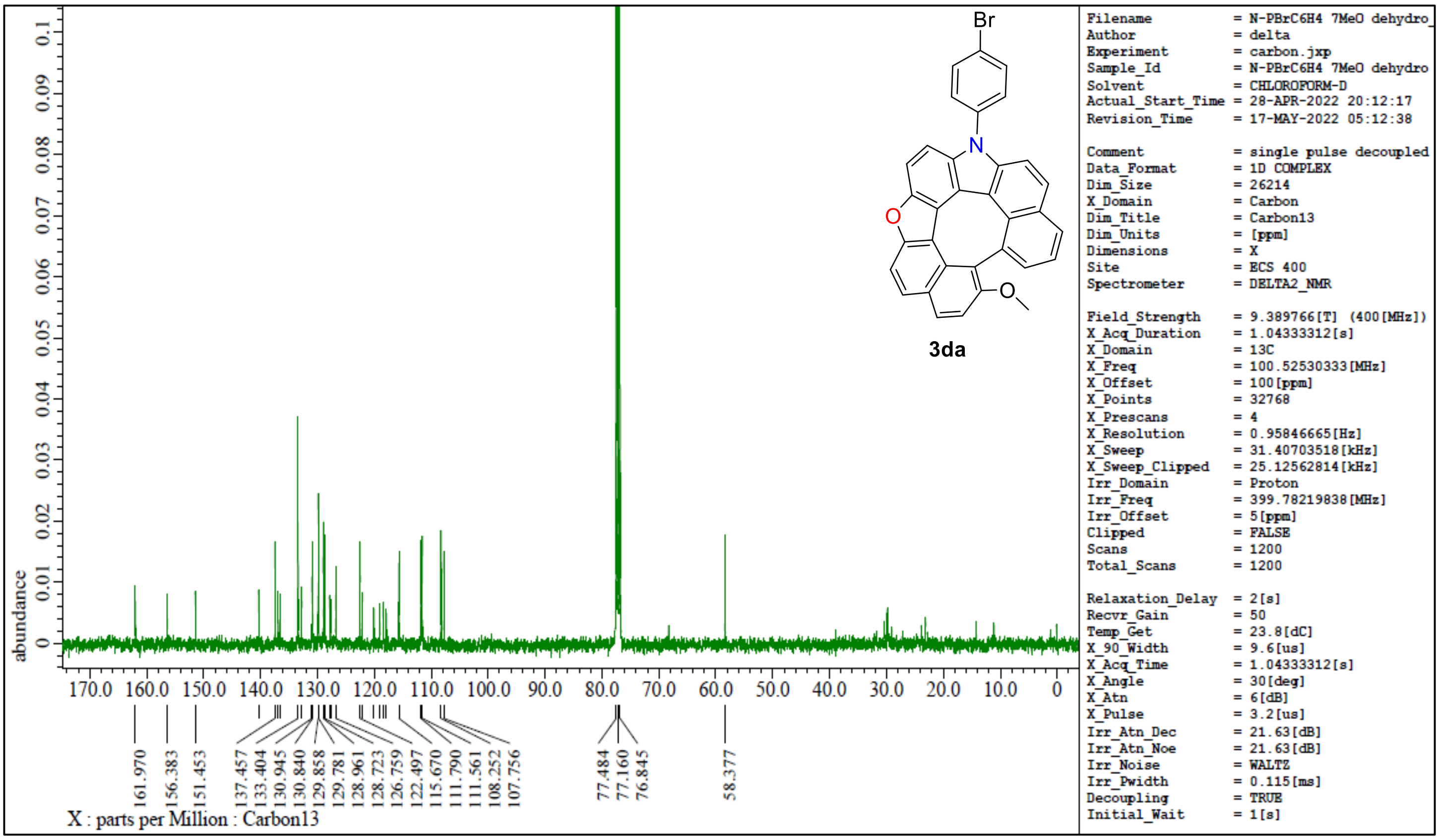

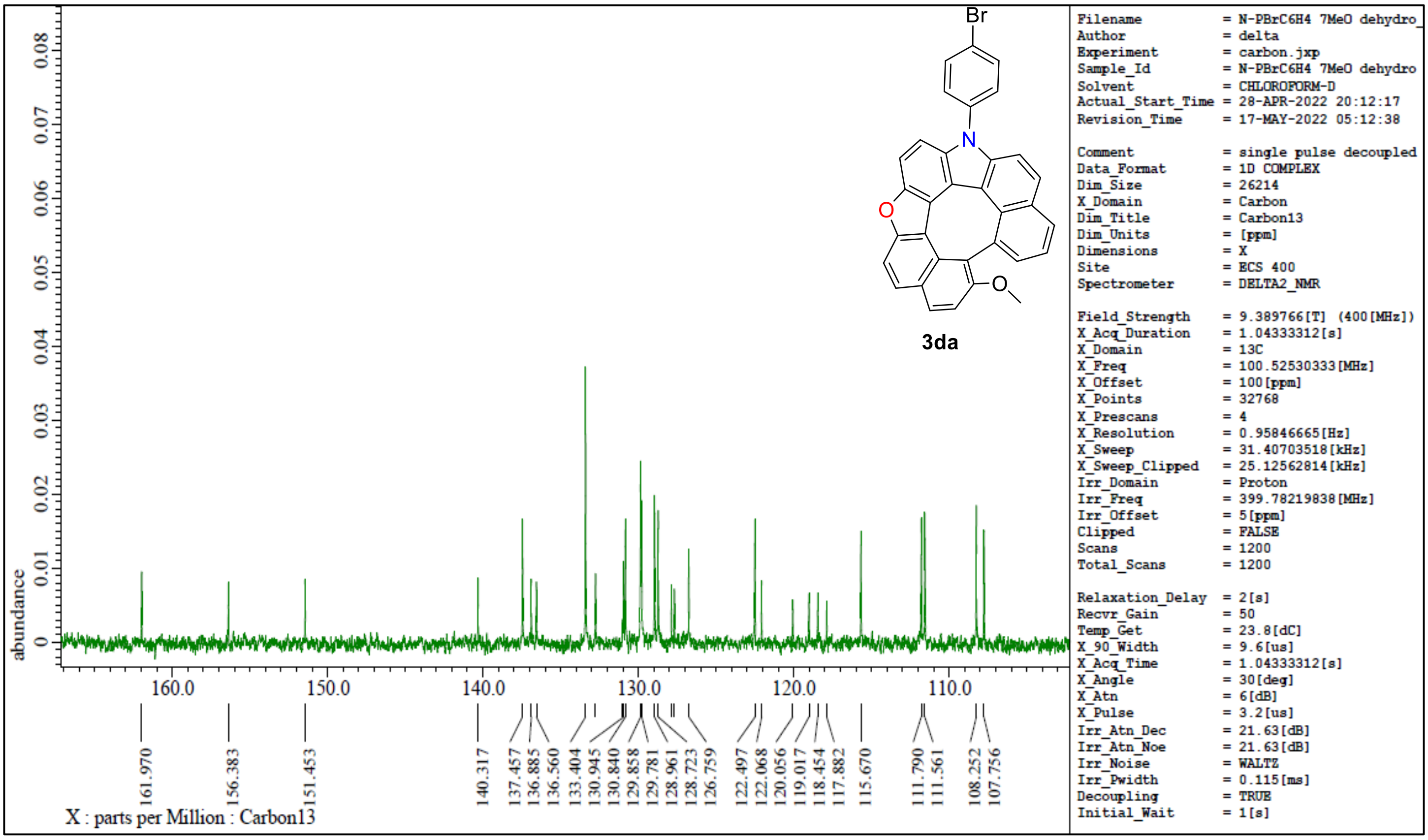

Compound **3da** (^{13}C NMR, 100 MHz, $CDCl_3$).

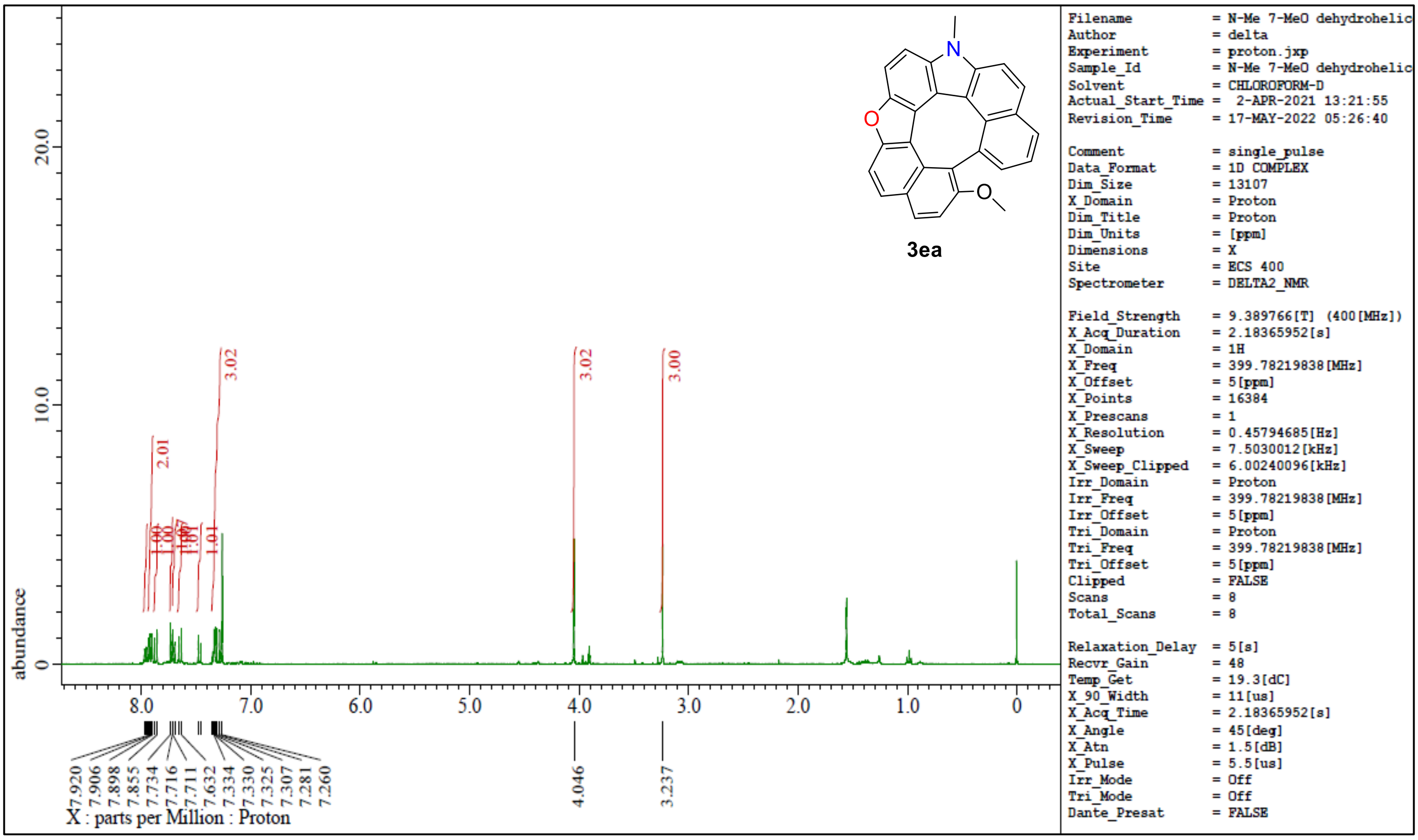

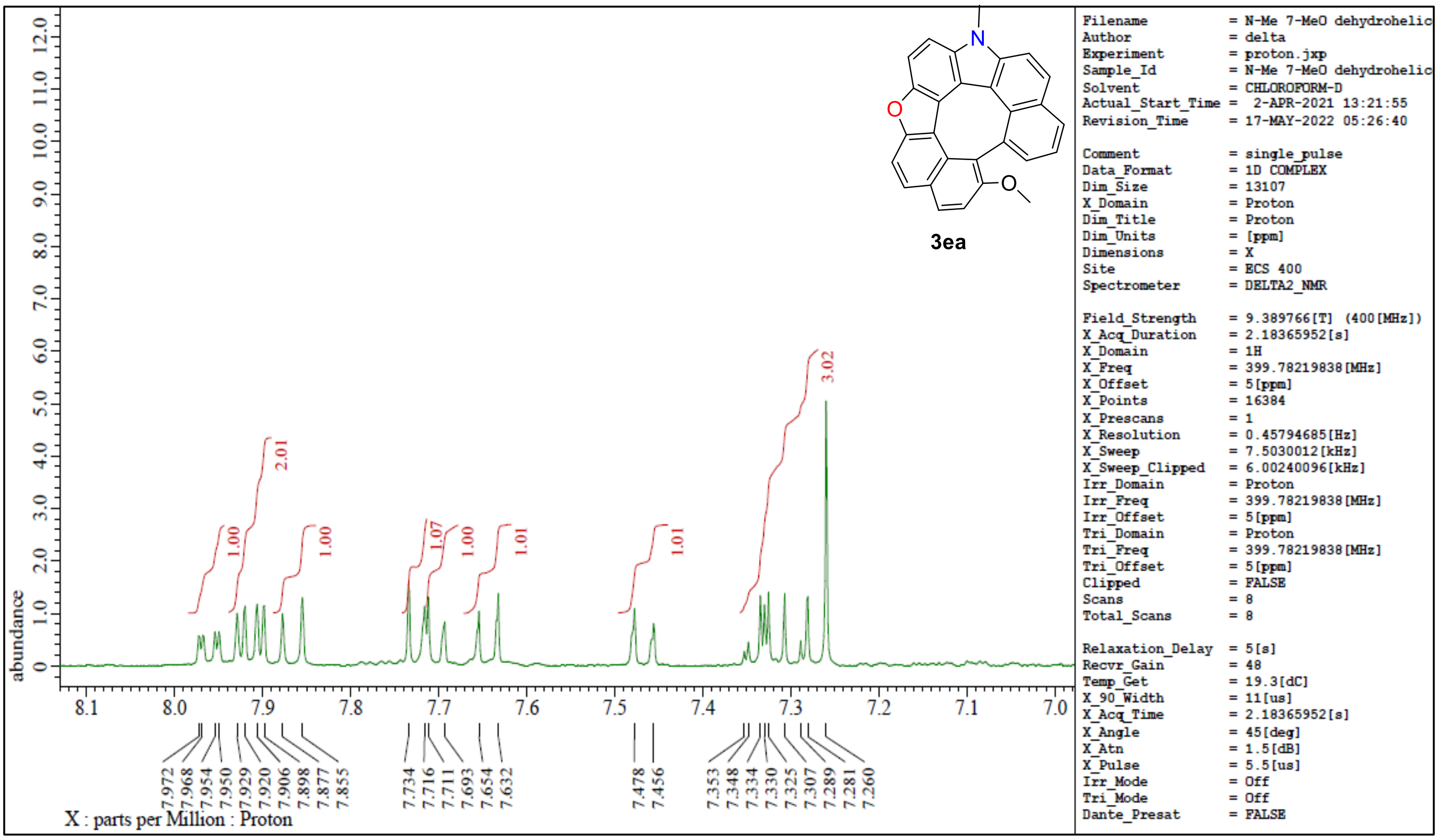

Compound **3ea** (^{1}H NMR, 400 MHz, $CDCl_3$).

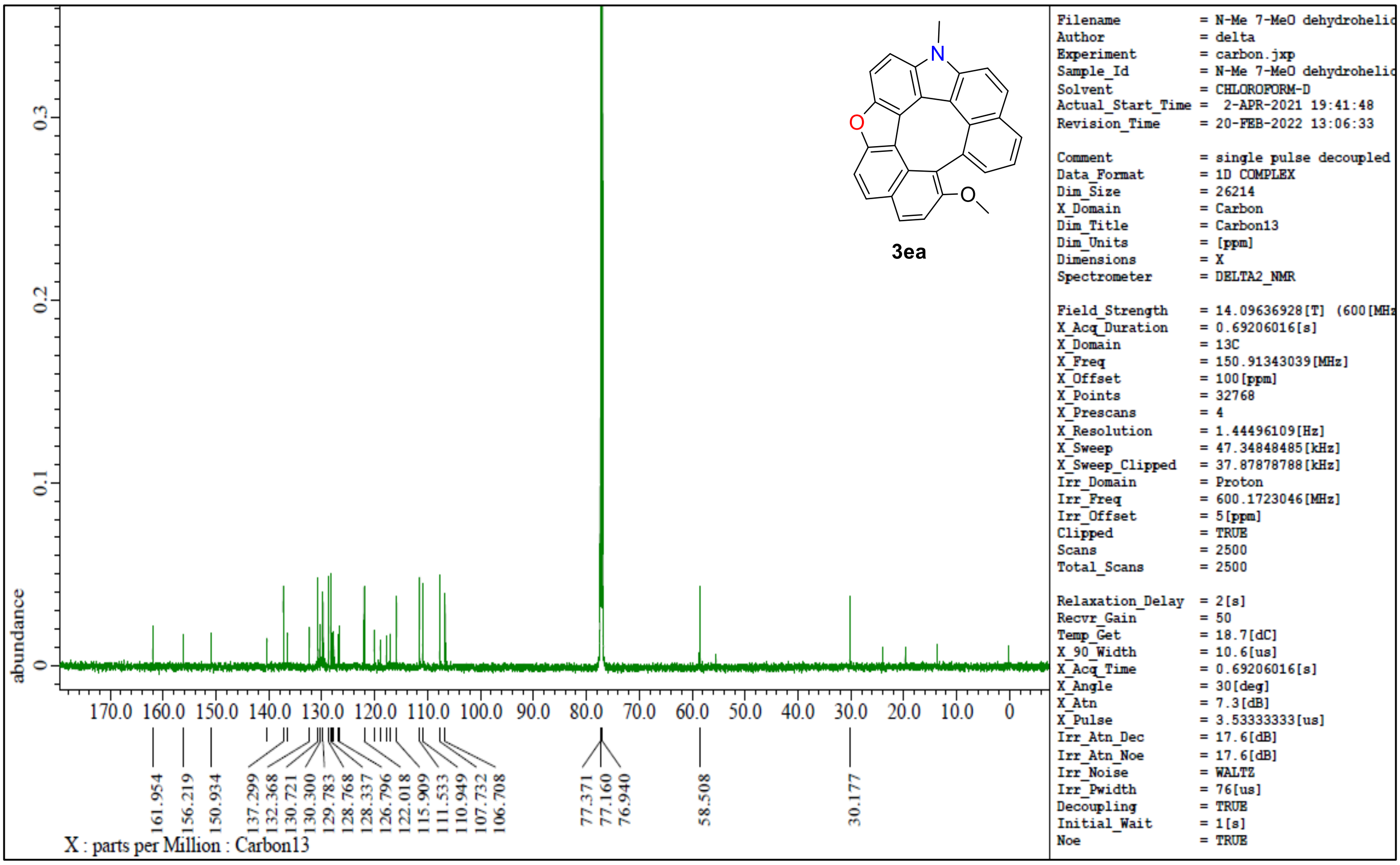

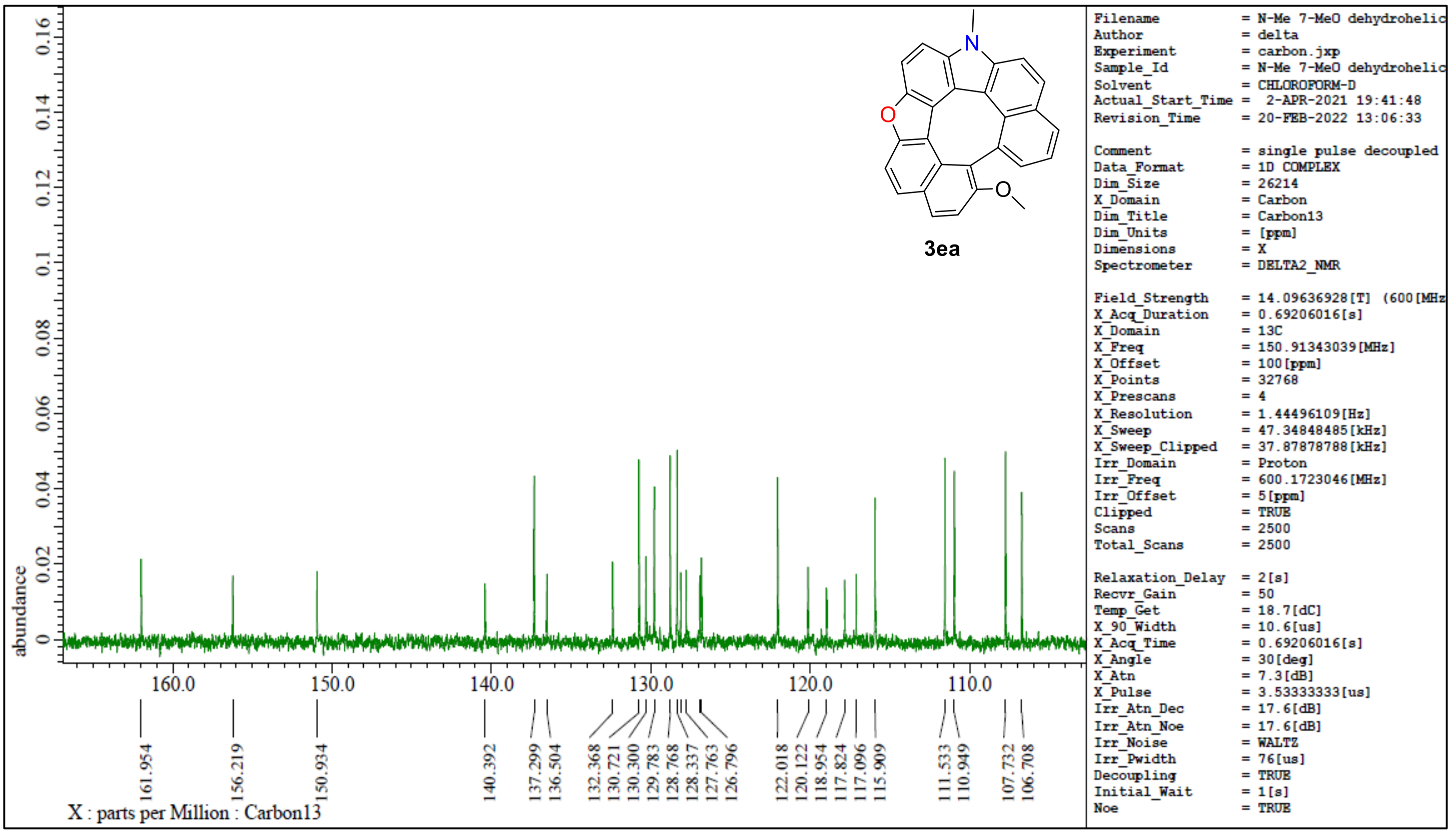

Compound **3ea** (^{13}C NMR, 150 MHz, $CDCl_3$).

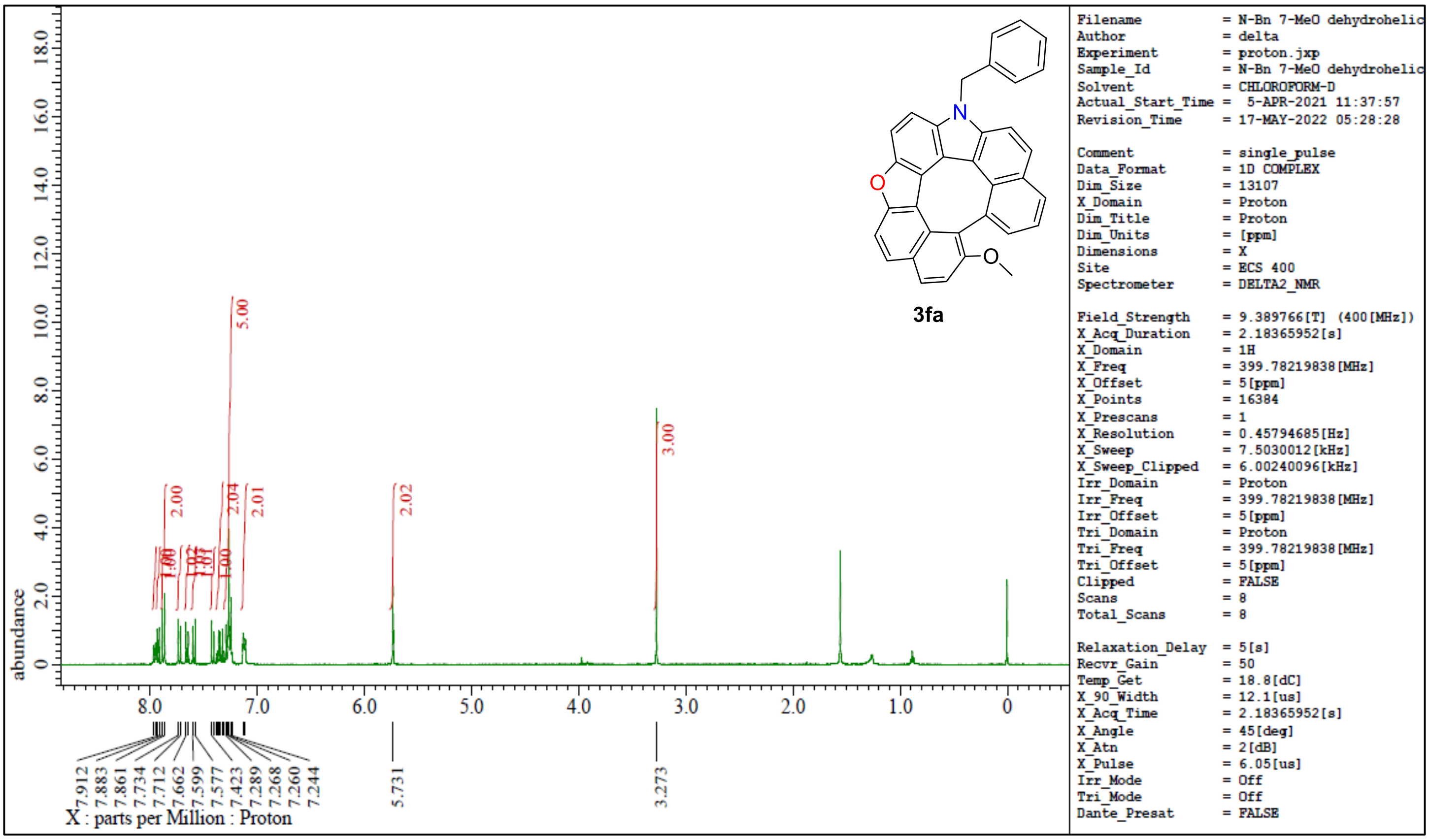

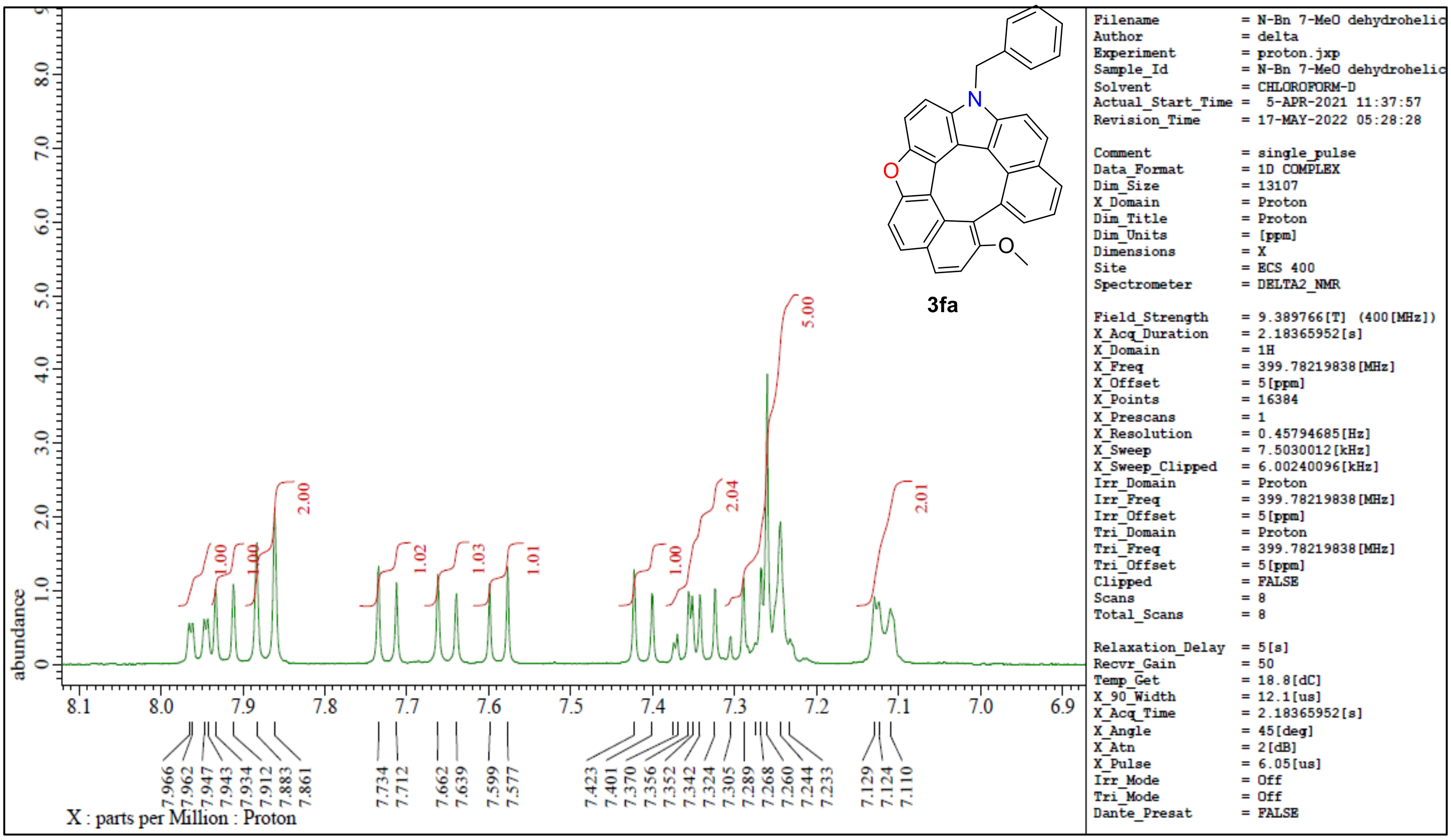

Compound **3fa** (^{1}H NMR, 400 MHz, $CDCl_3$).

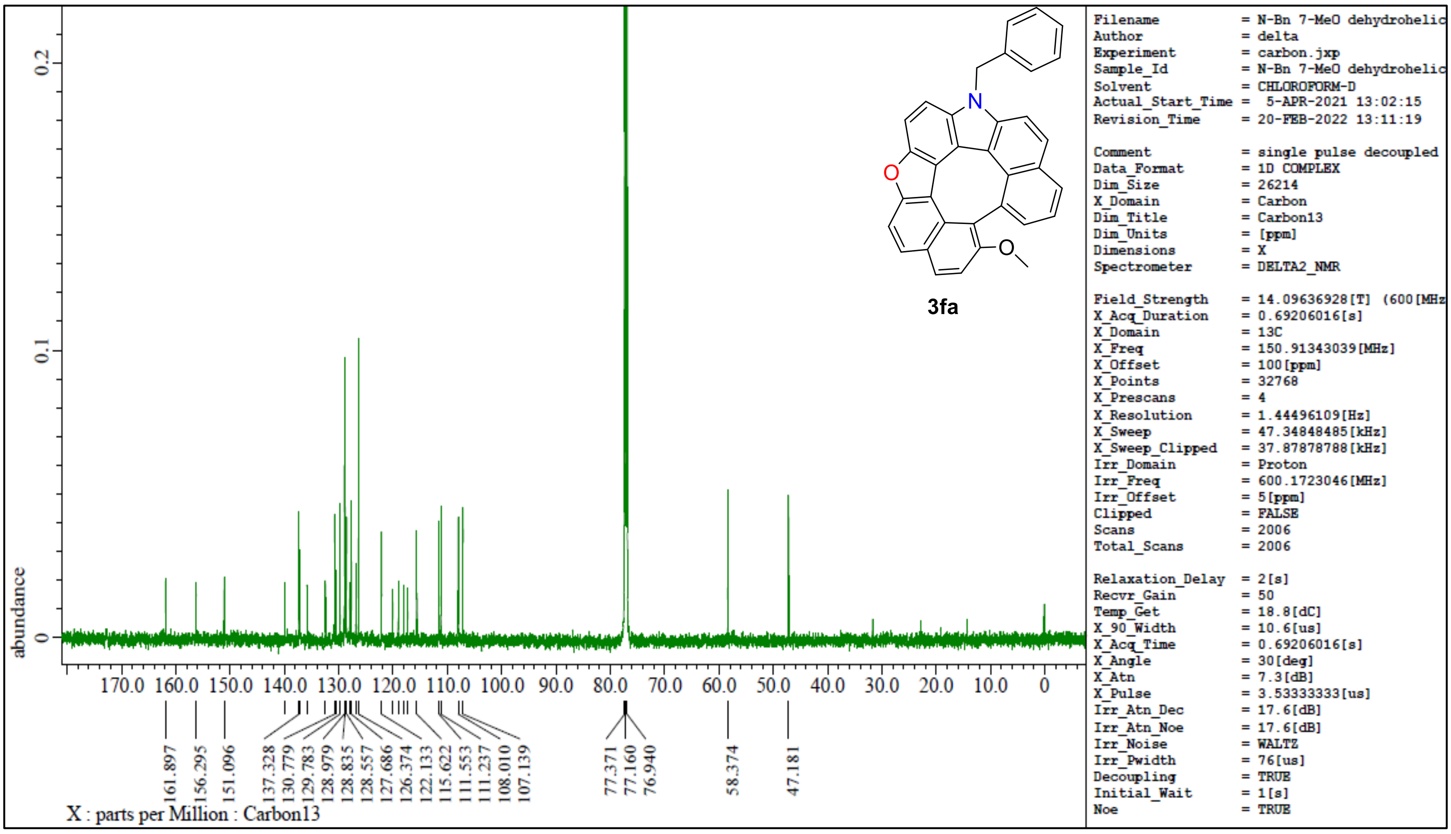

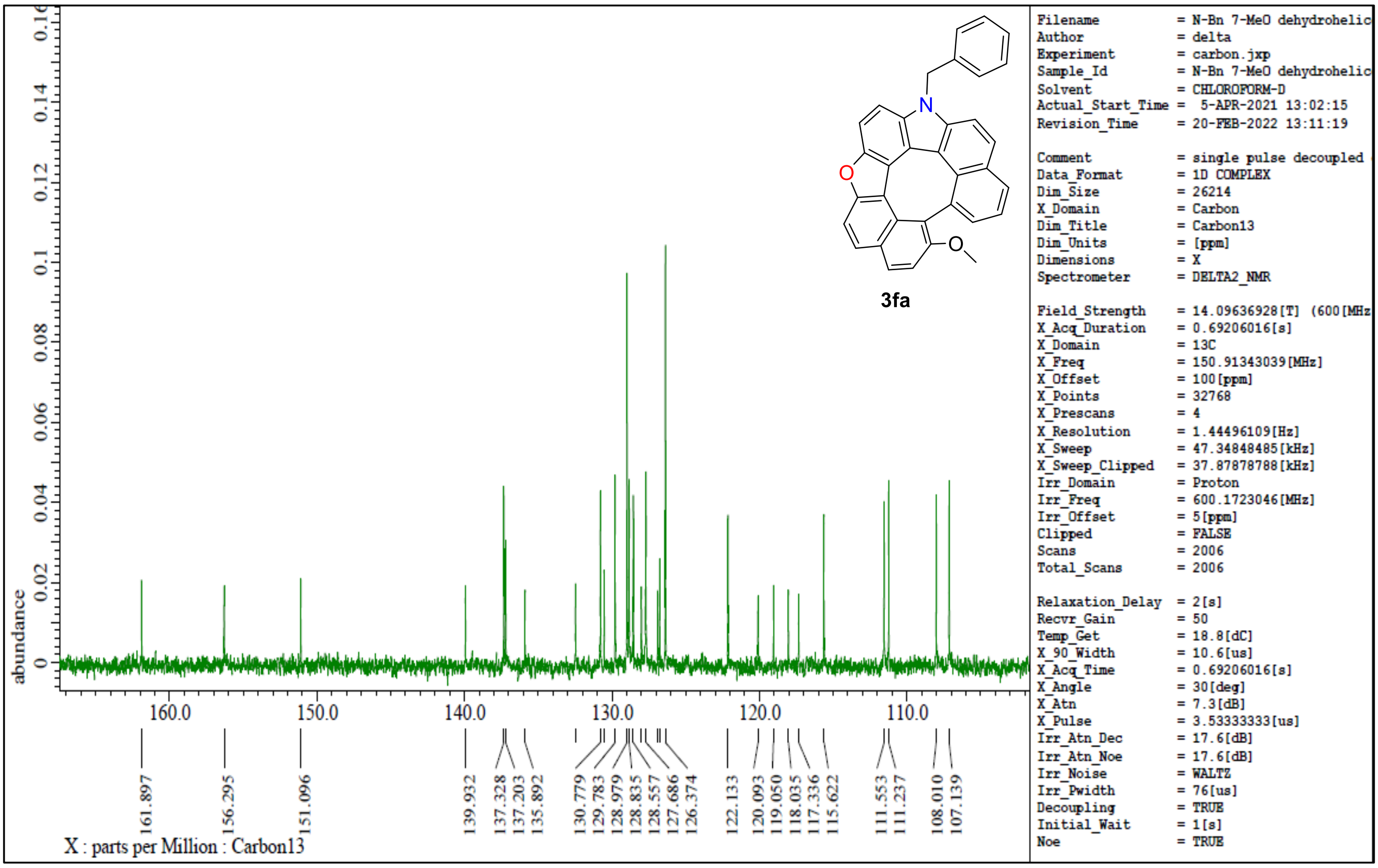

Compound **3fa** (^{13}C NMR, 150 MHz, $CDCl_3$).

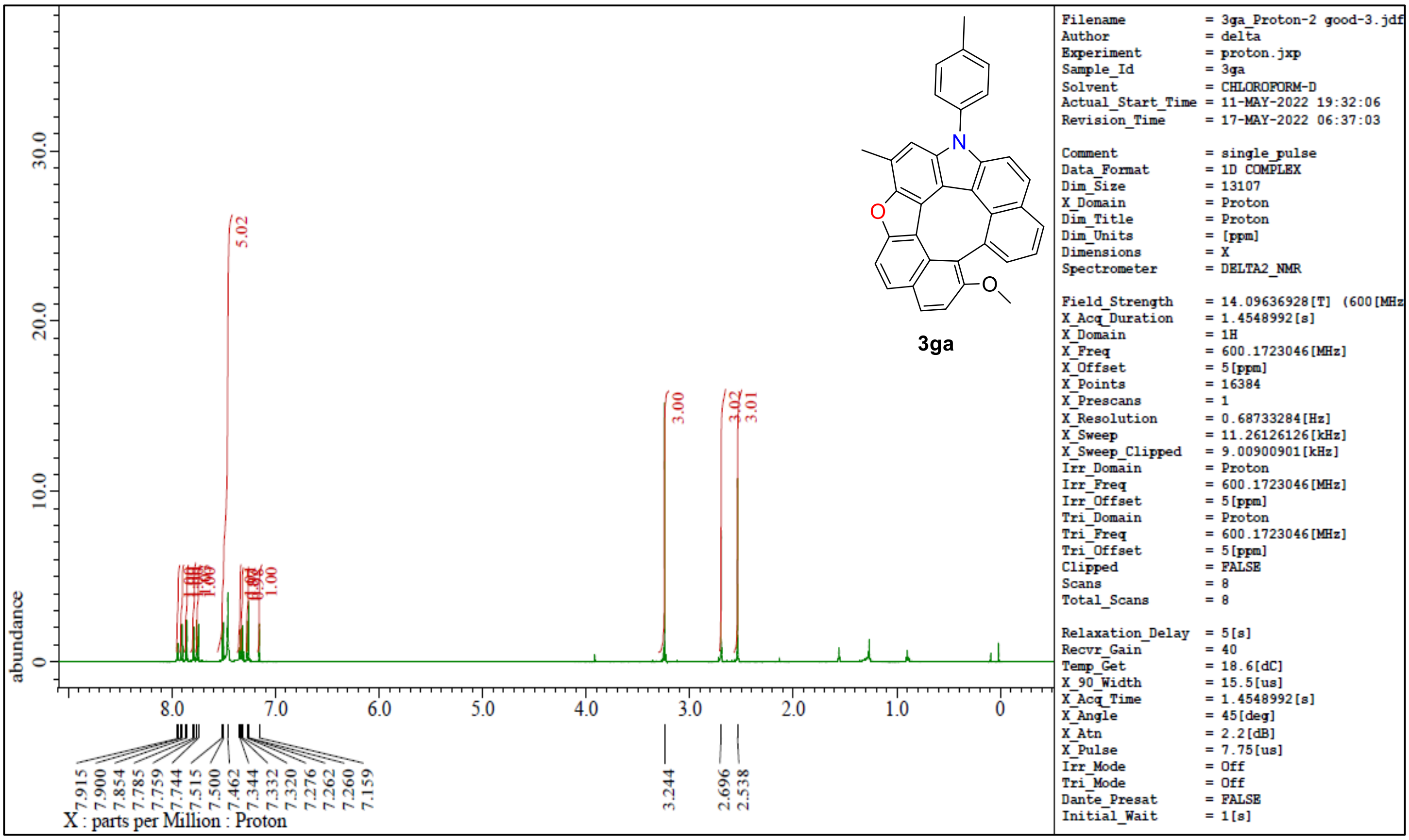

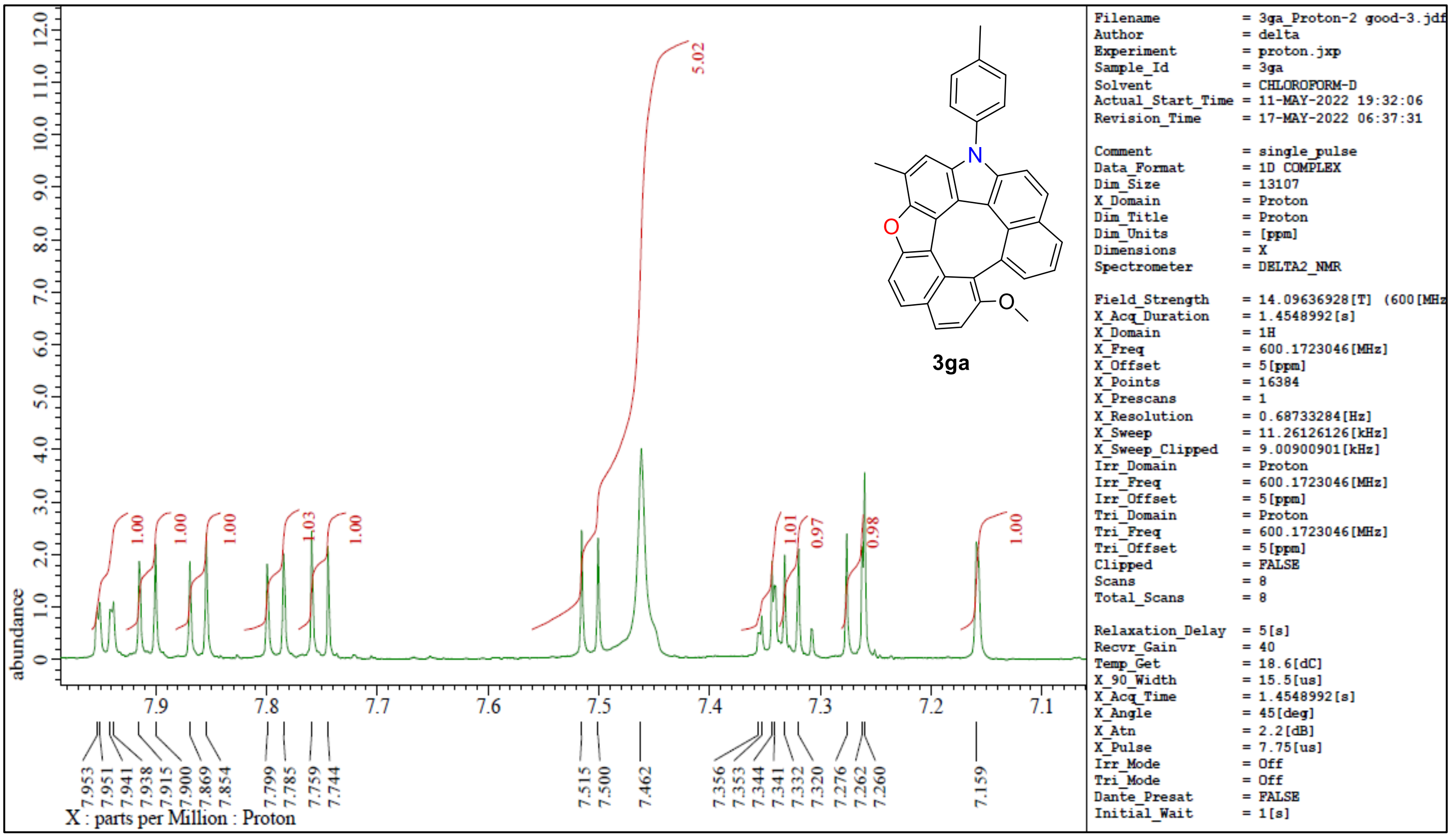

Compound **3ga** (^{1}H NMR, 600 MHz, $CDCl_3$).

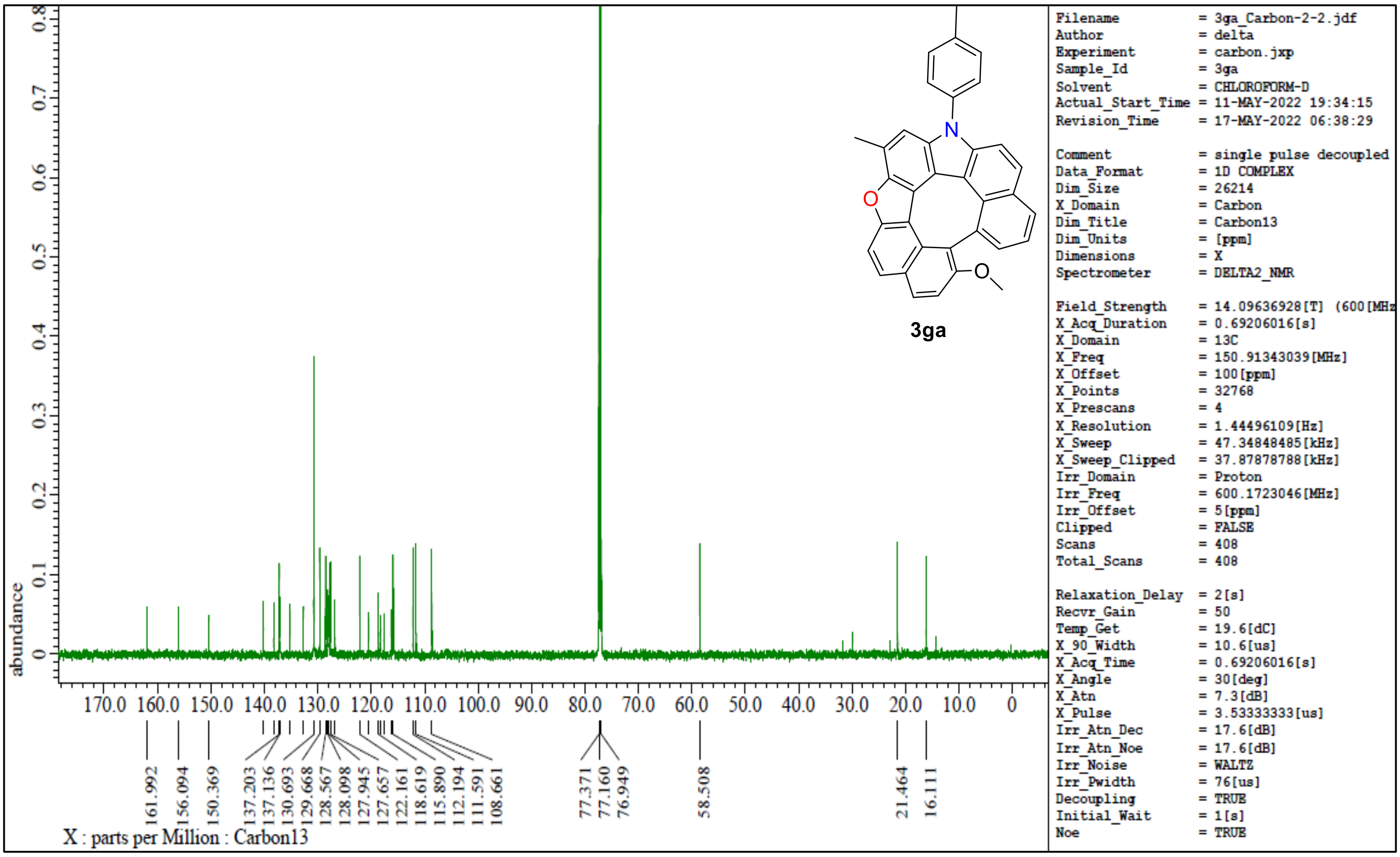

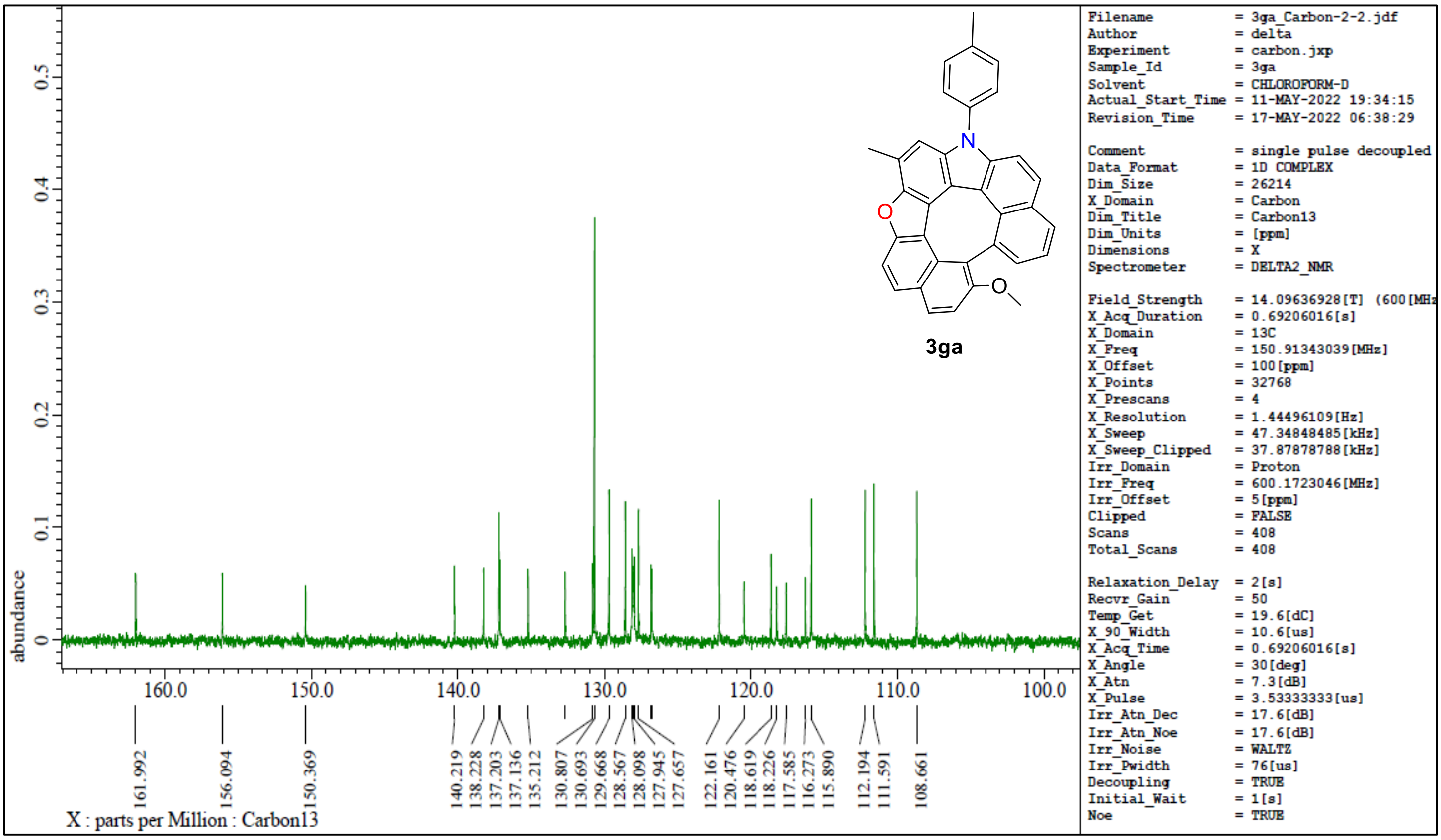

Compound **3ga** (^{13}C NMR, 150 MHz, $CDCl_3$).

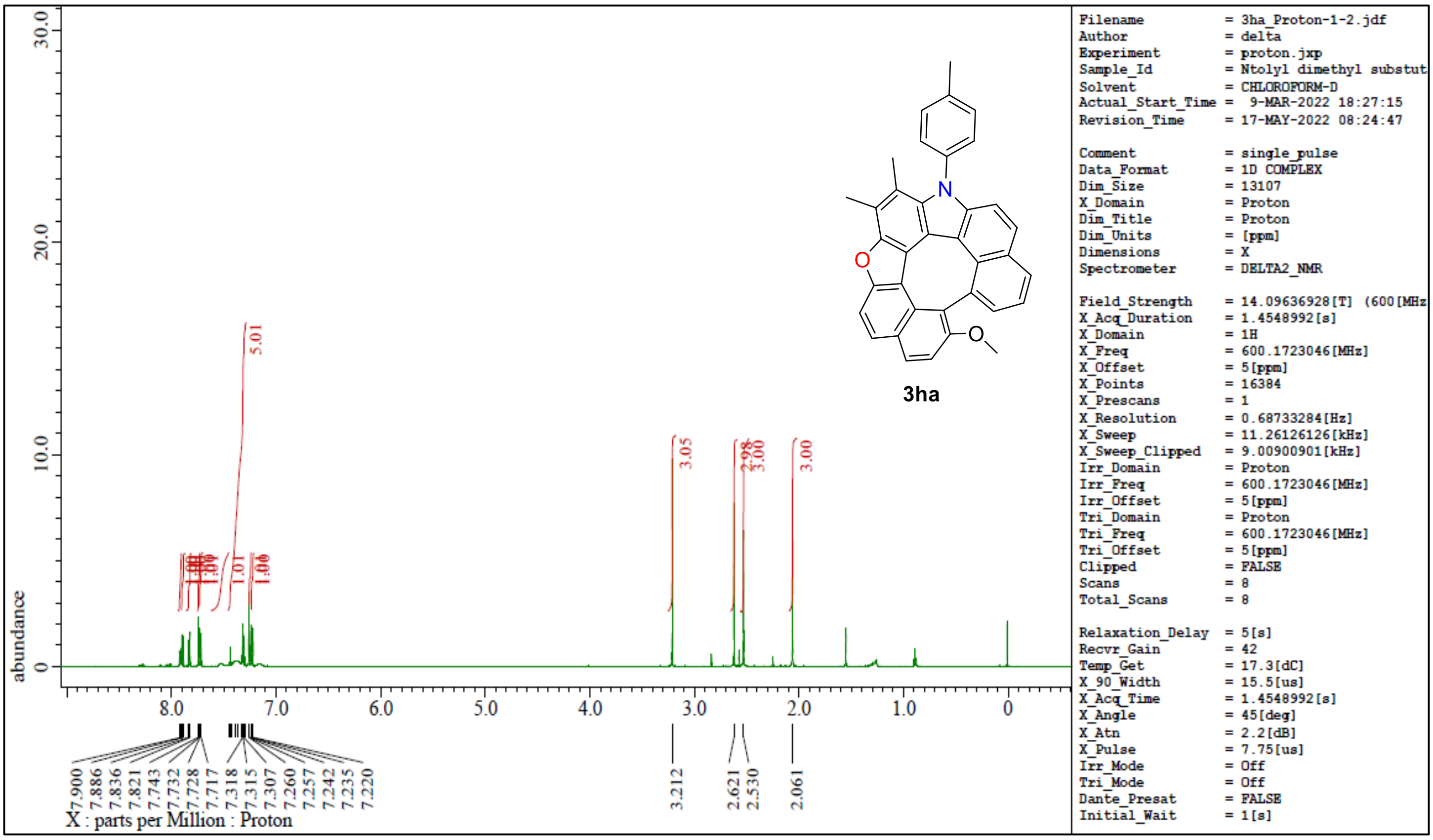

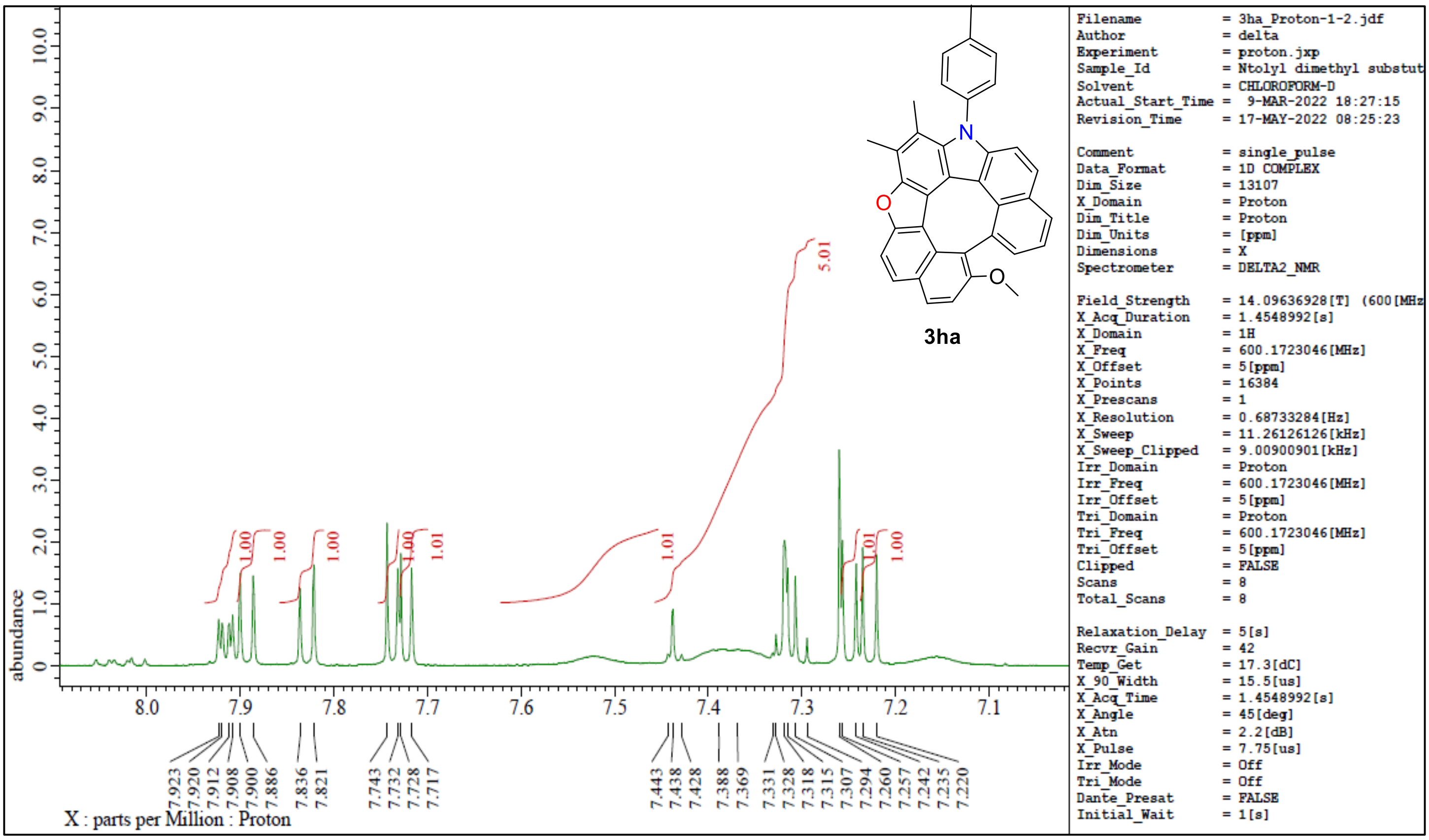

Compound **3ha** (^{1}H NMR, 600 MHz, $CDCl_3$).

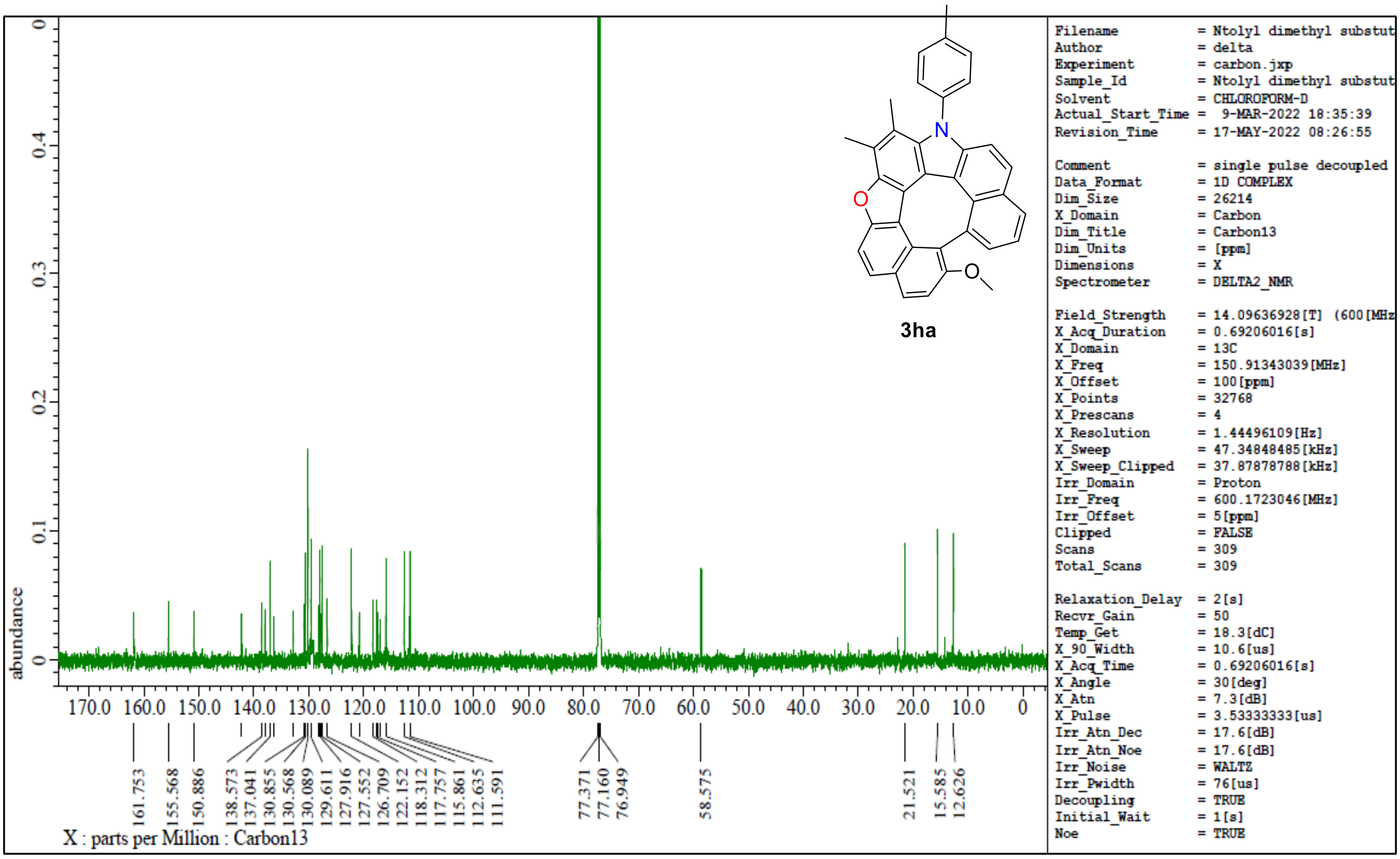

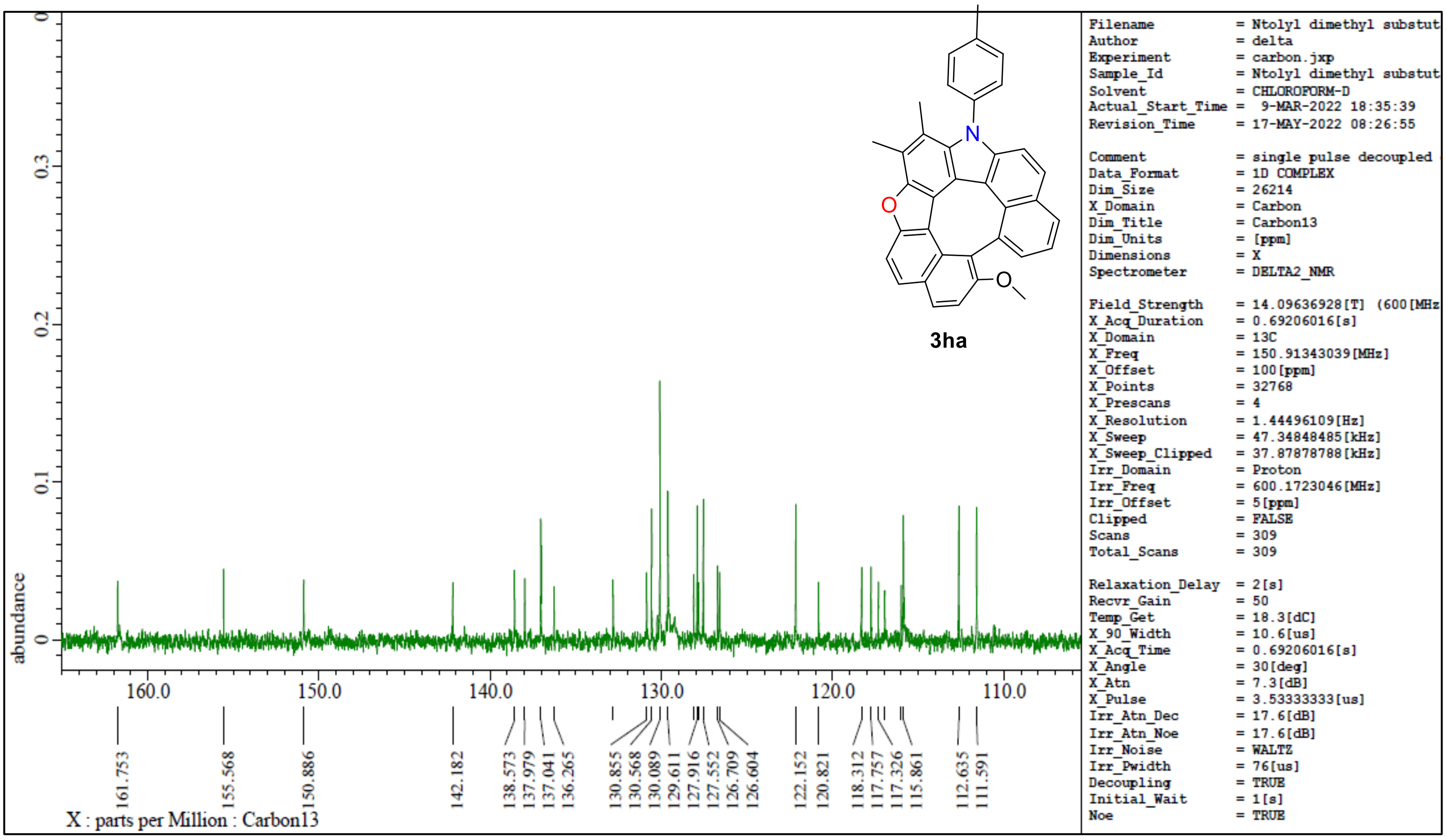

Compound **3ha** (^{13}C NMR, 150 MHz, $CDCl_3$).

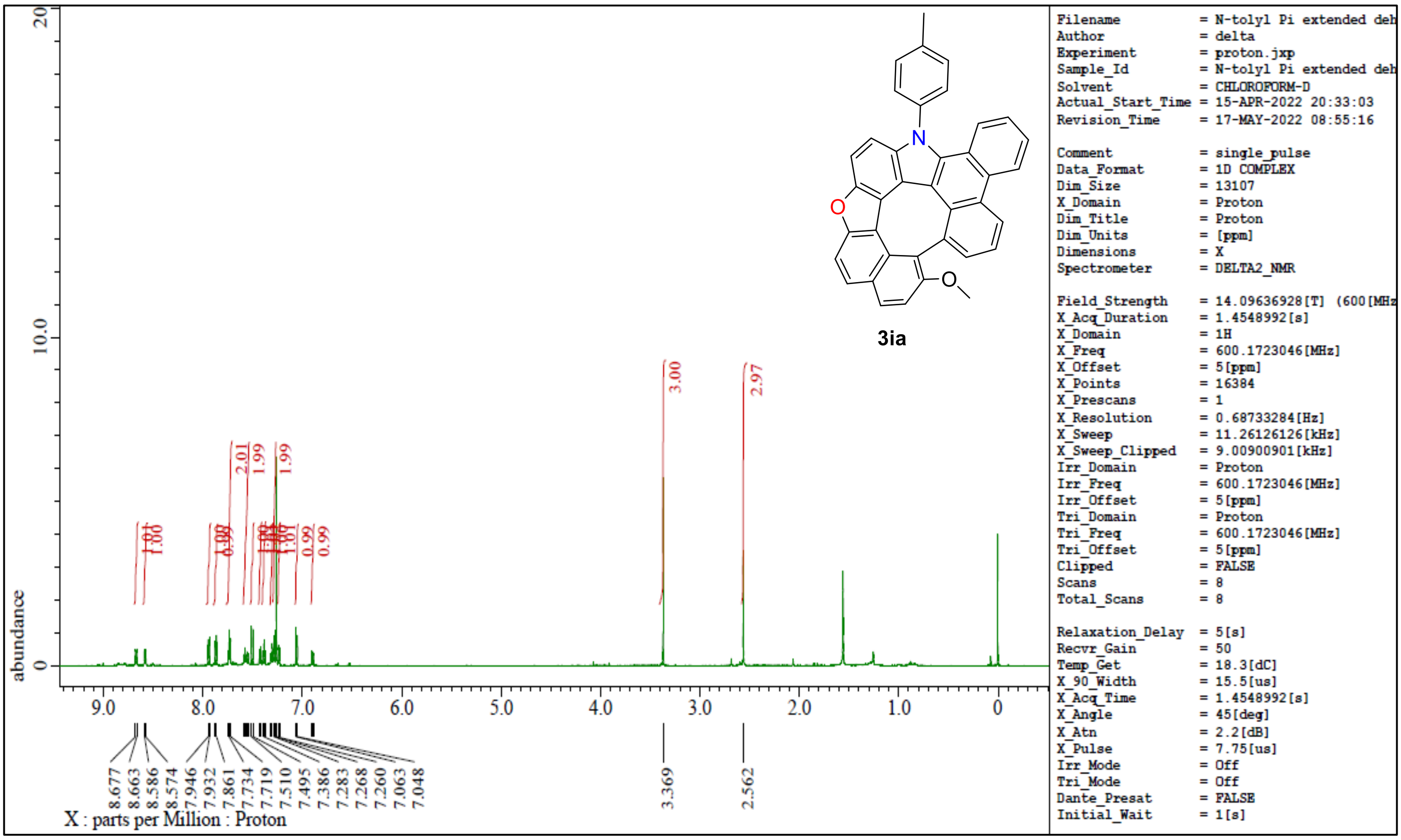

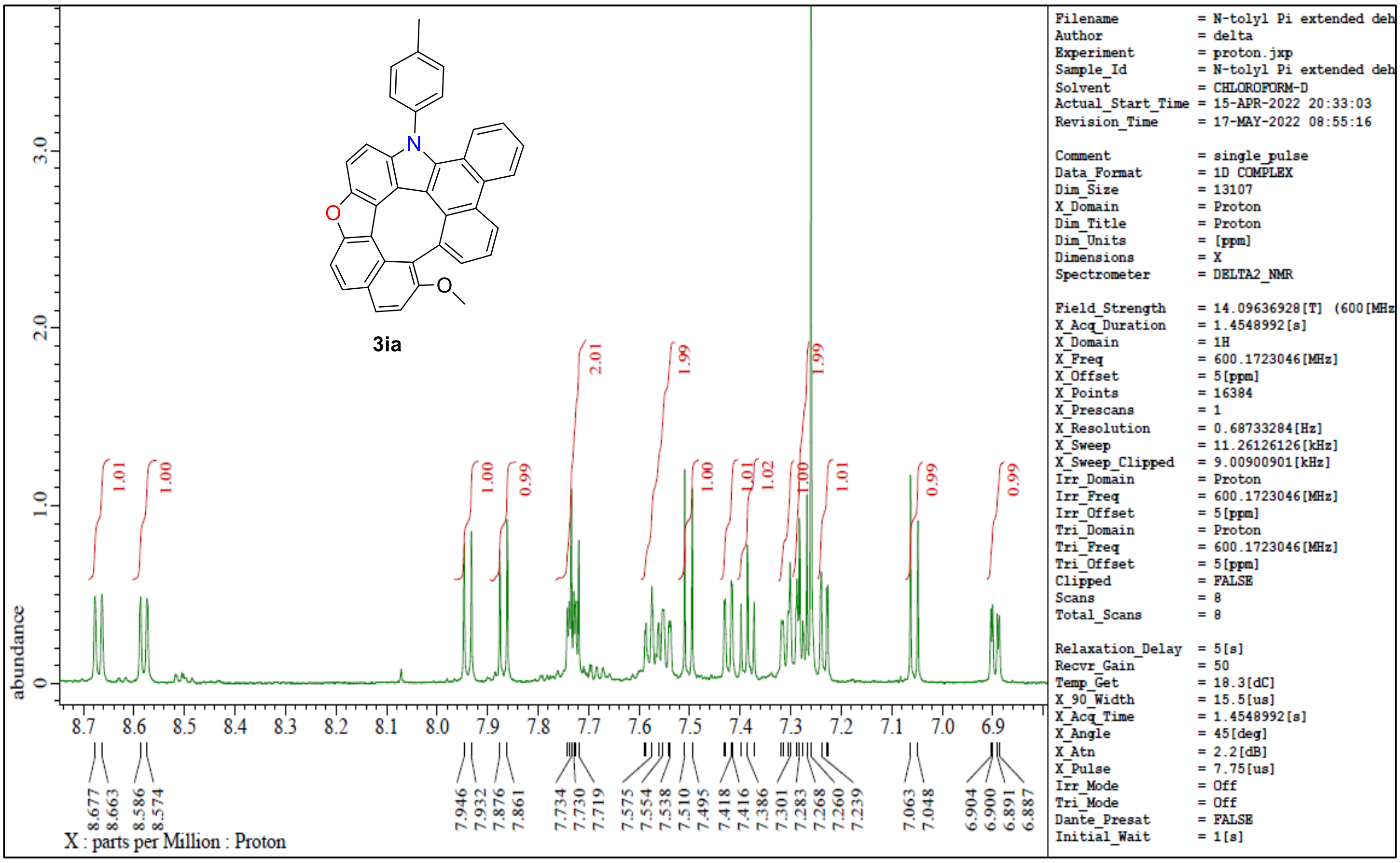

Compound **3ia** (^{1}H NMR, 600 MHz, $CDCl_3$).

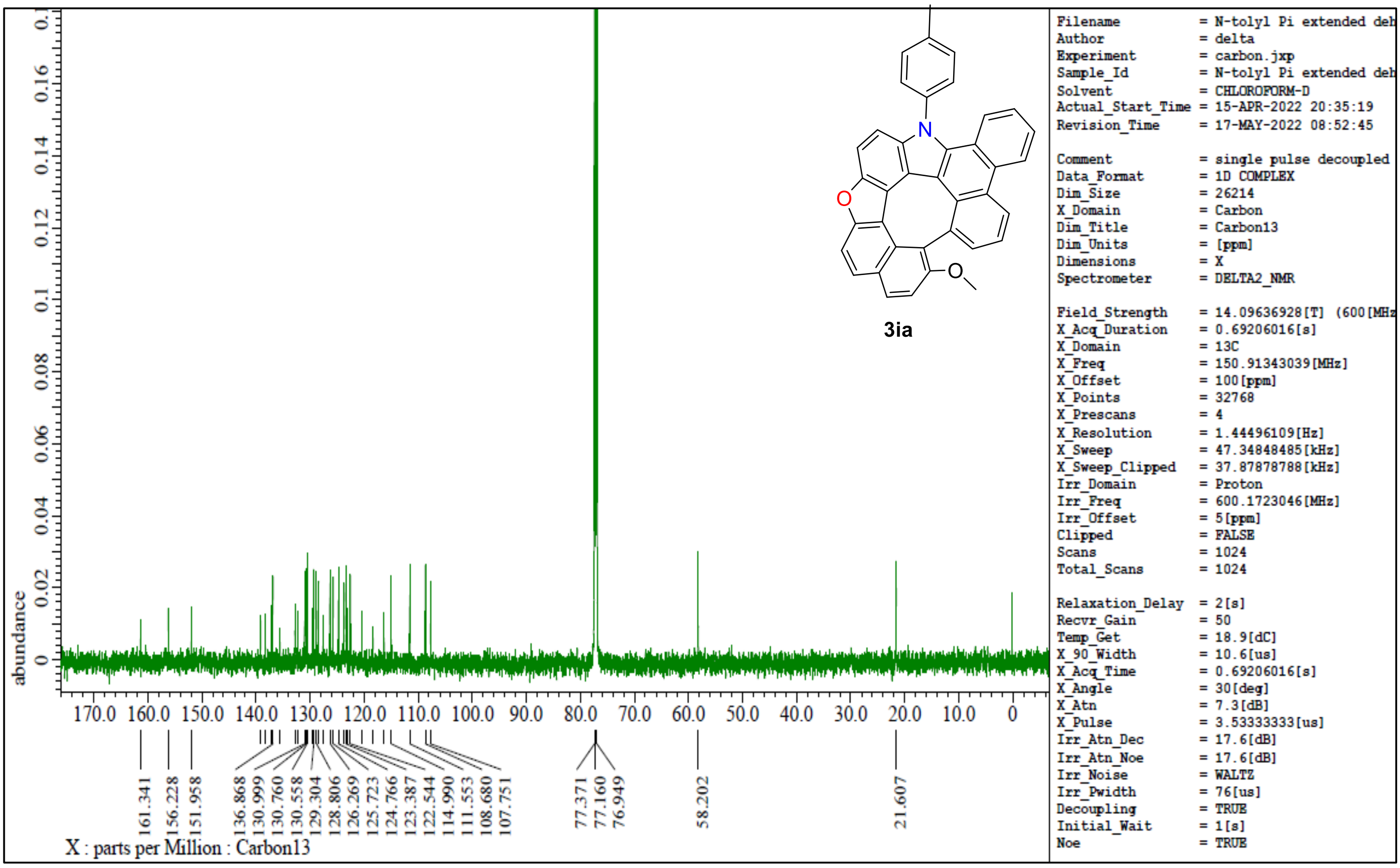

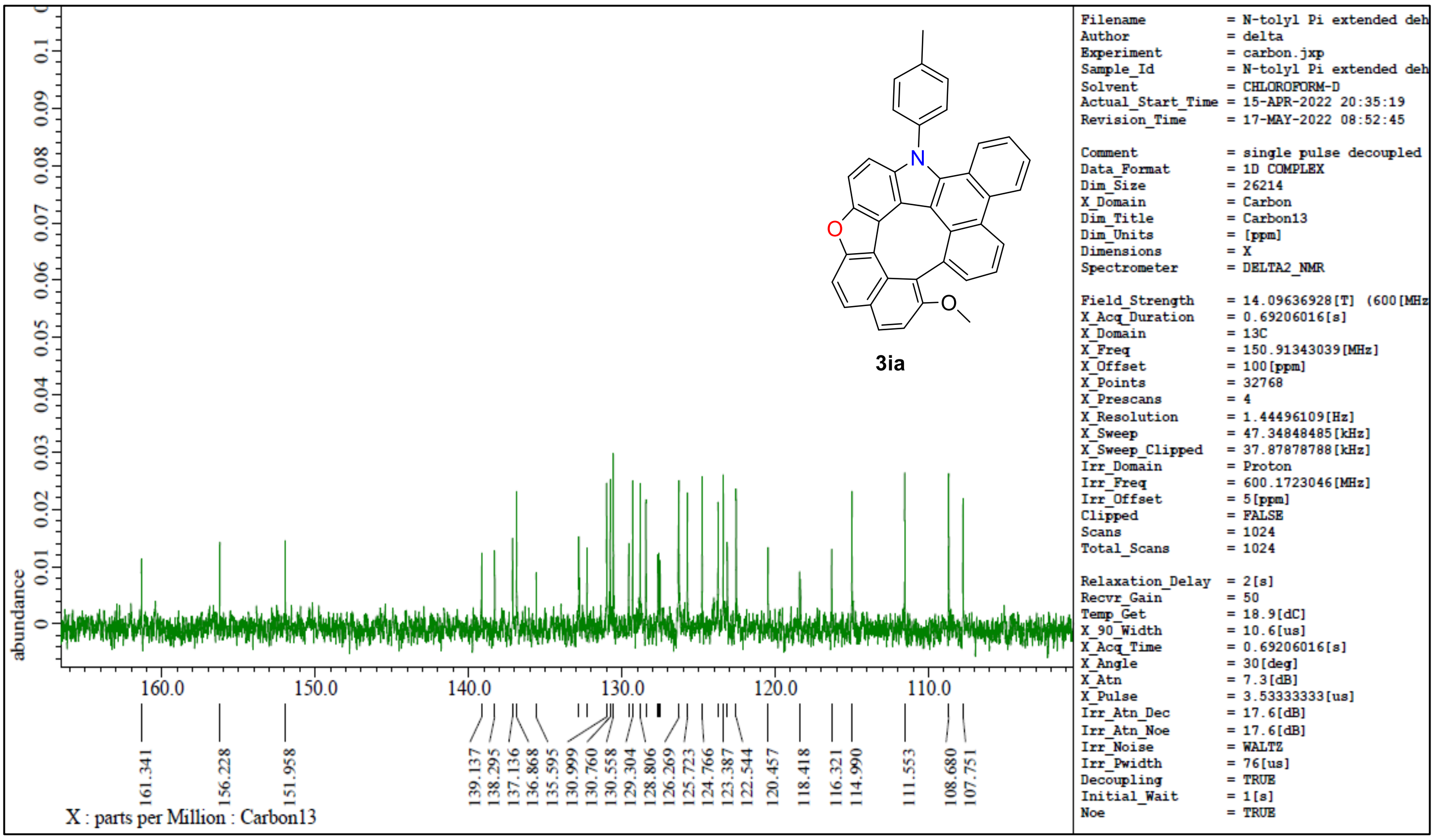

Compound **3ia** (^{13}C NMR, 150 MHz, $CDCl_3$).

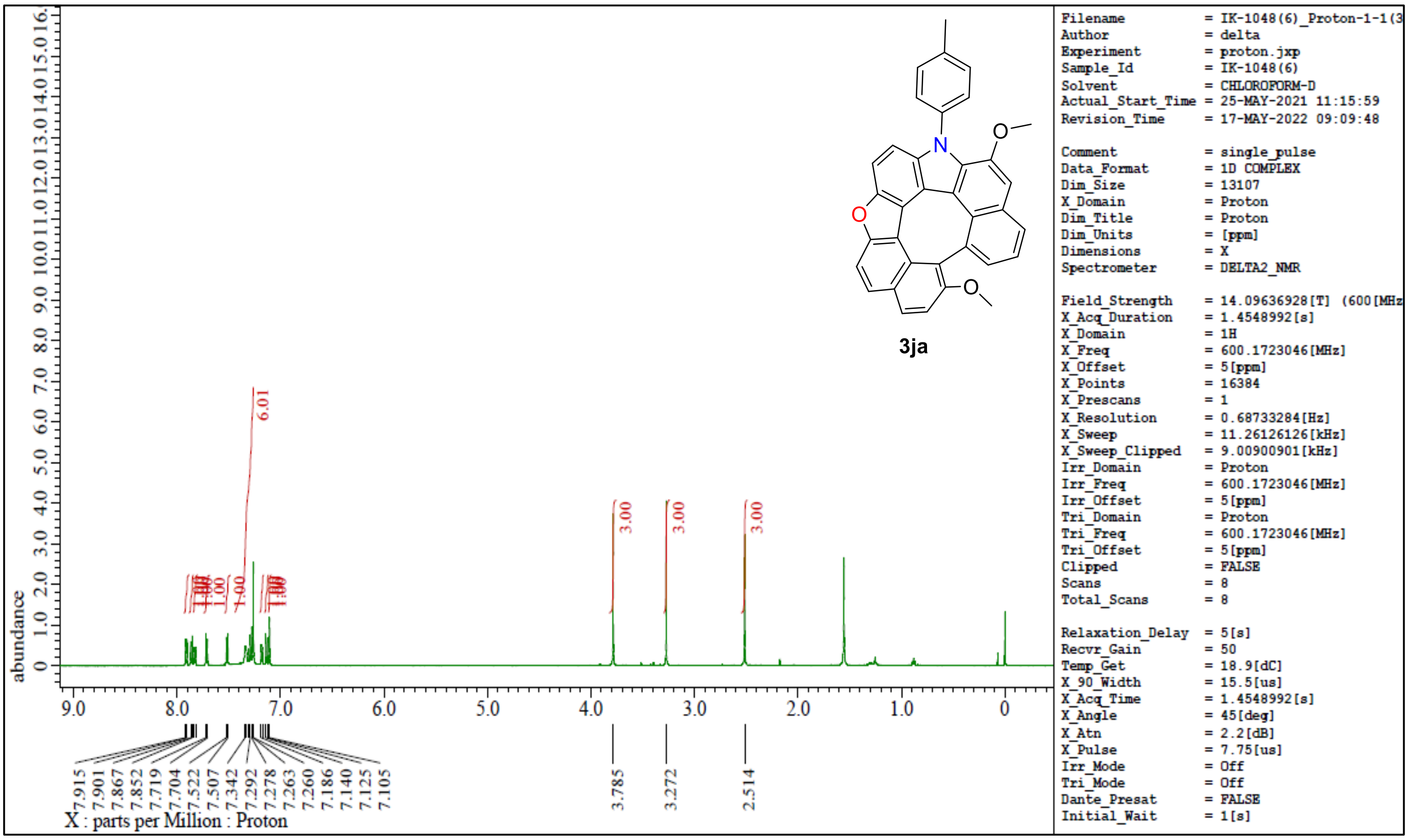

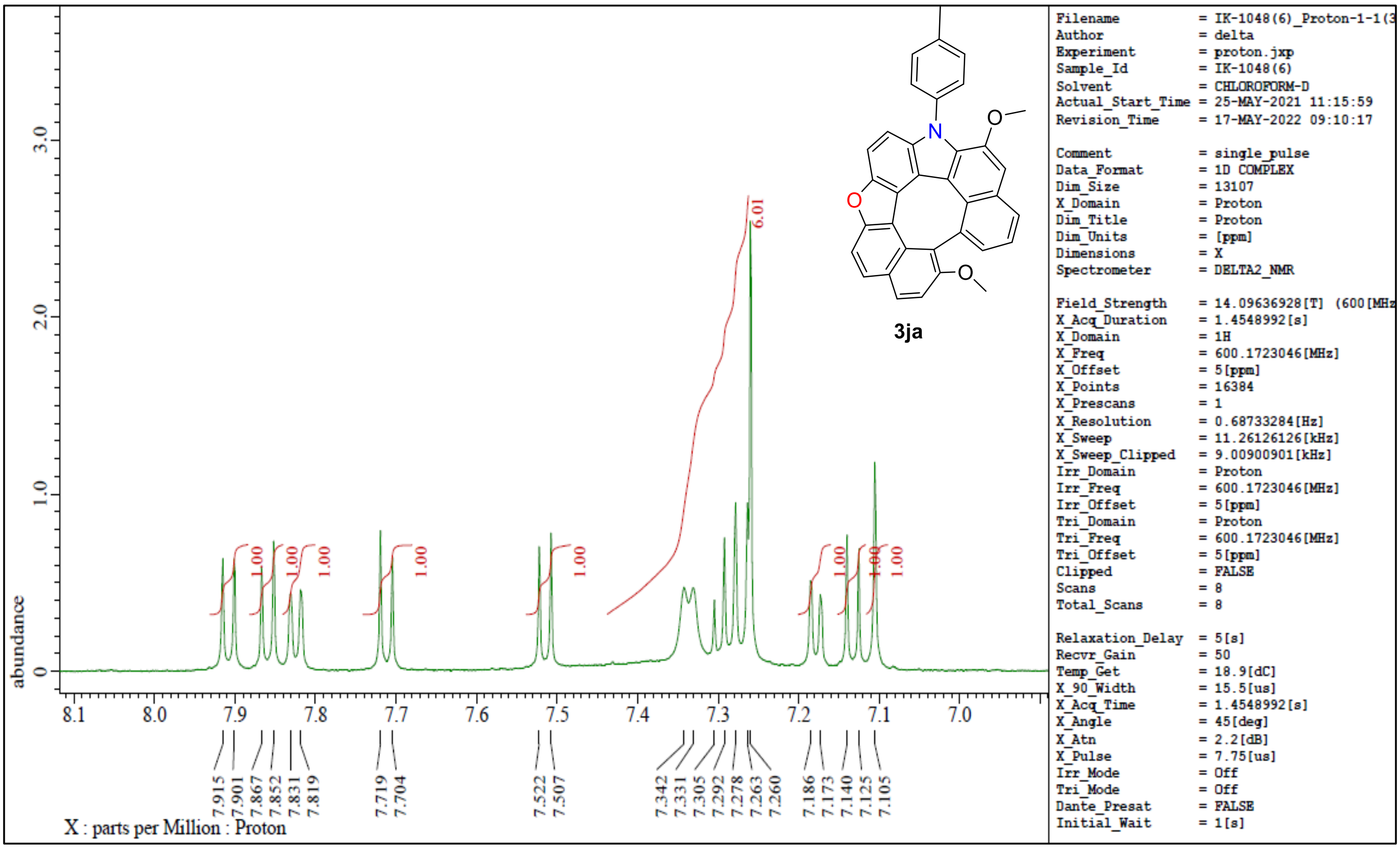

Compound **3ja** (^{1}H NMR, 600 MHz, $CDCl_3$).

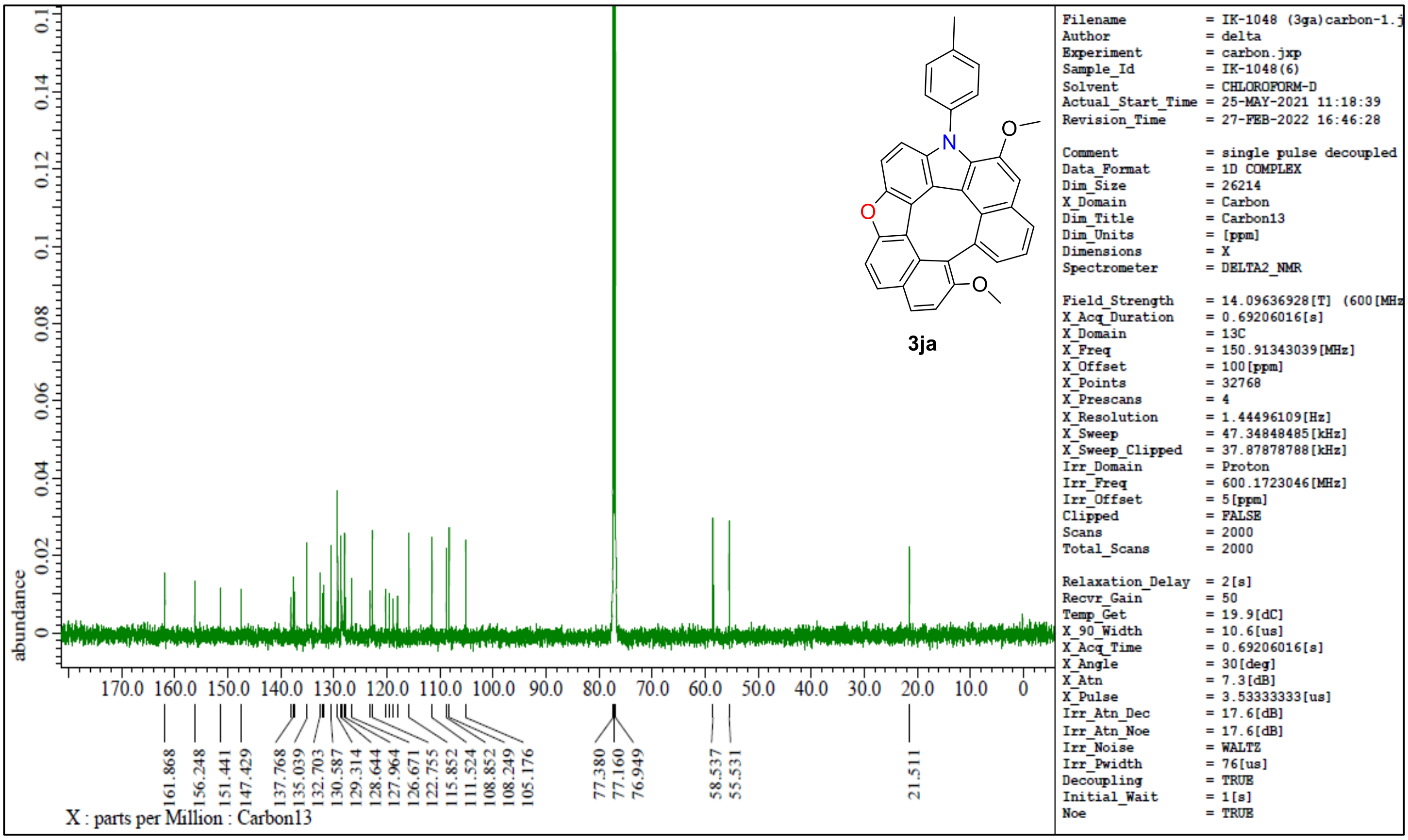

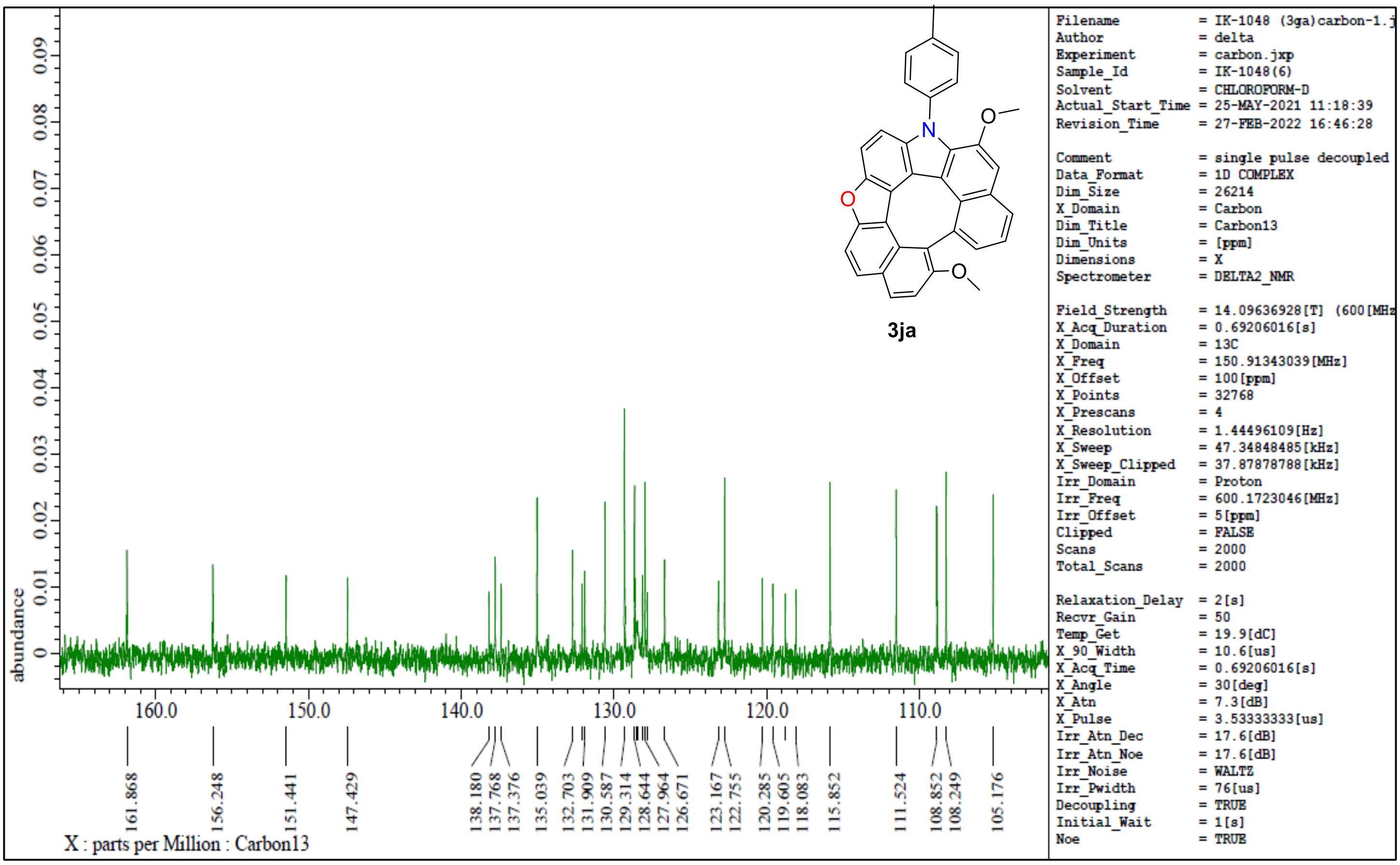

Compound **3ja** (^{13}C NMR, 150 MHz, $CDCl_3$).

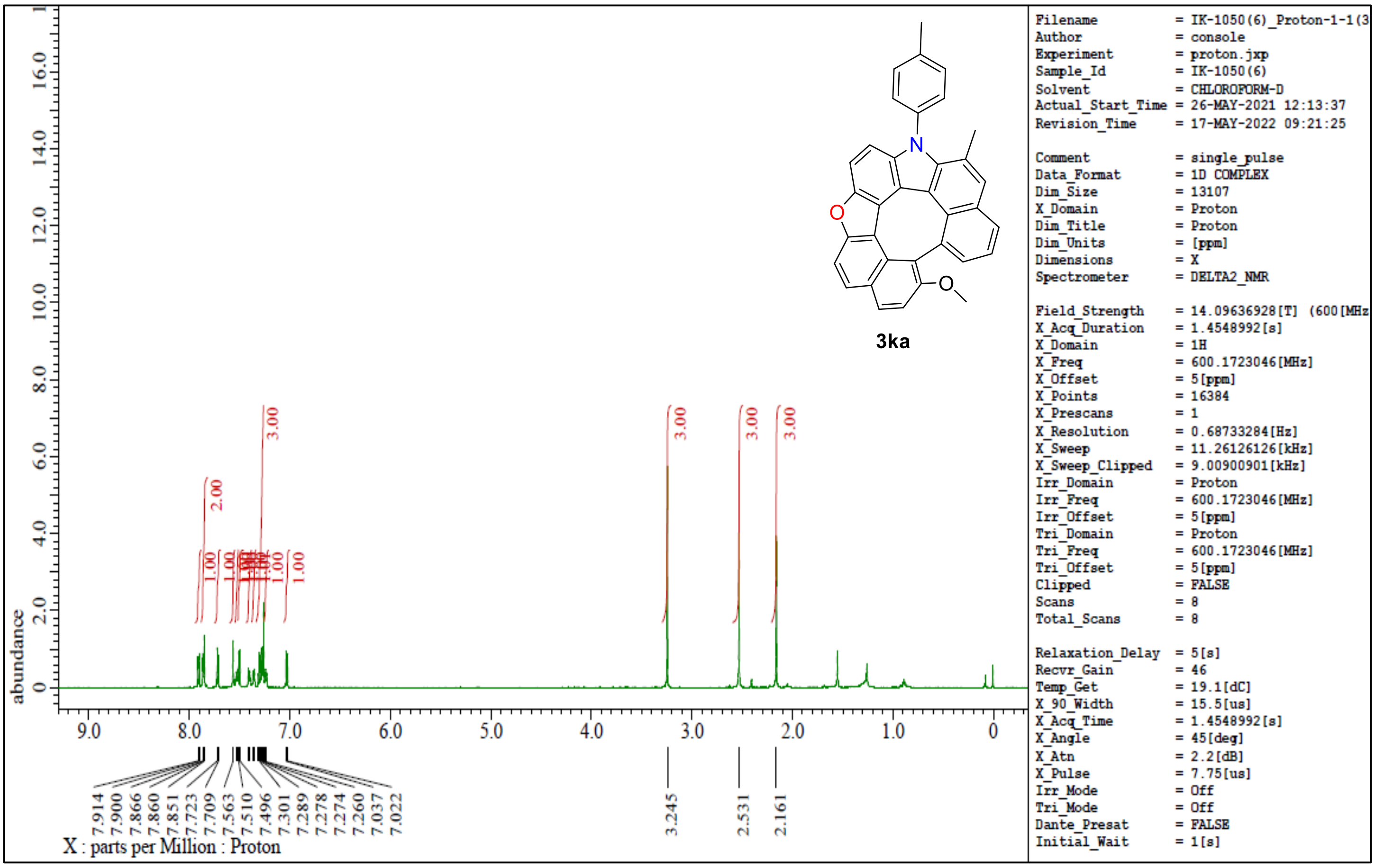

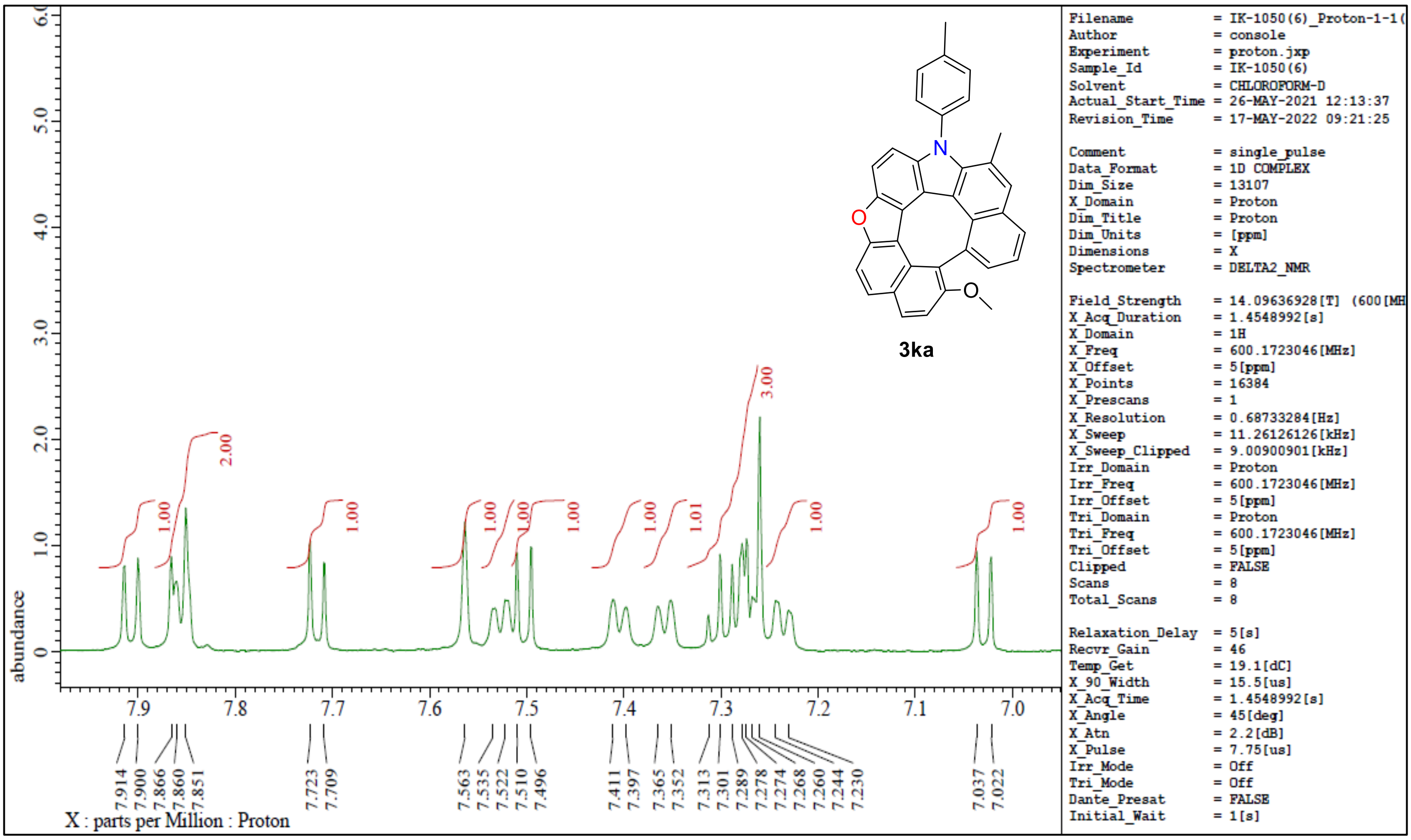

Compound **3ka** (^{1}H NMR, 600 MHz, $CDCl_3$).

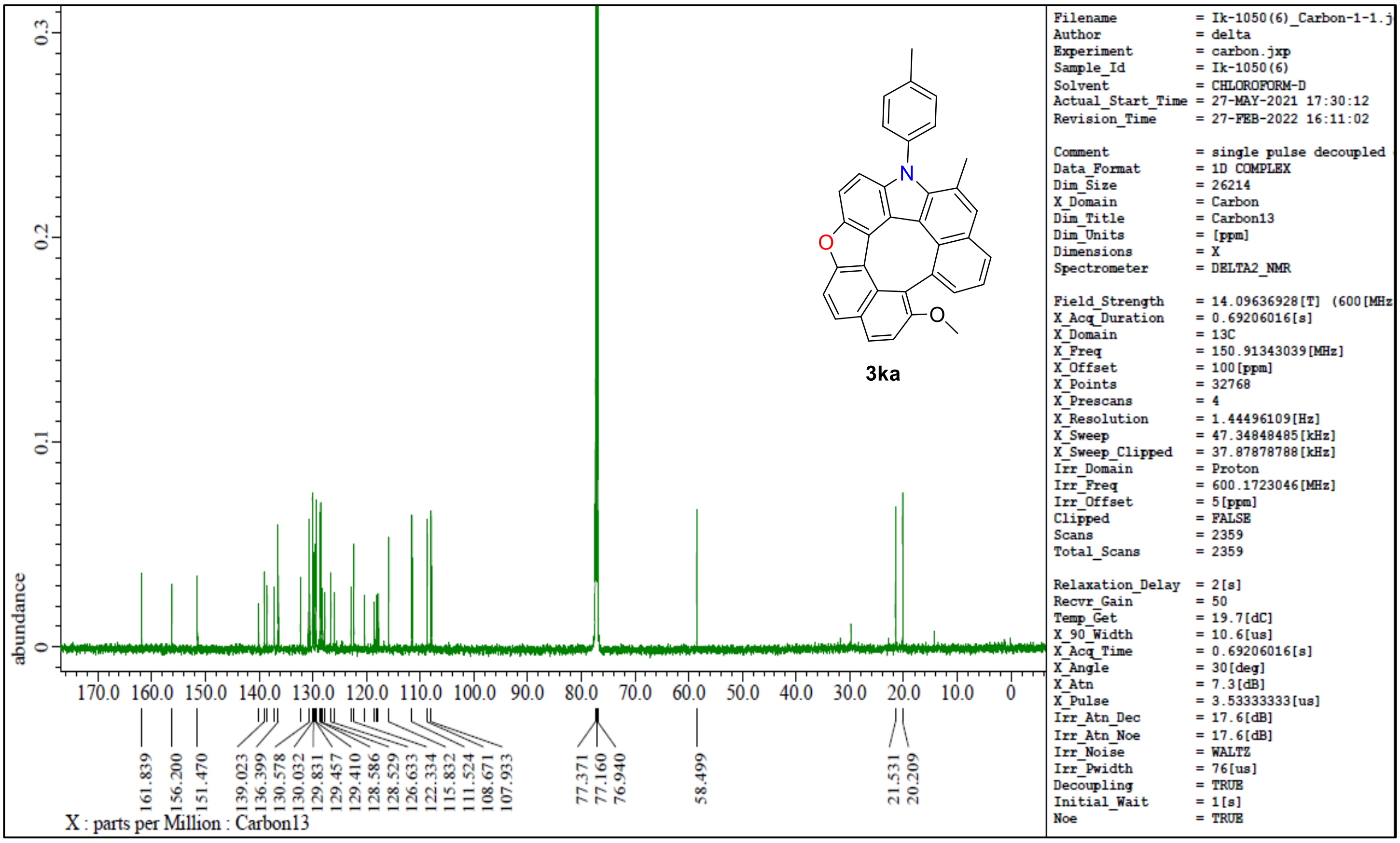

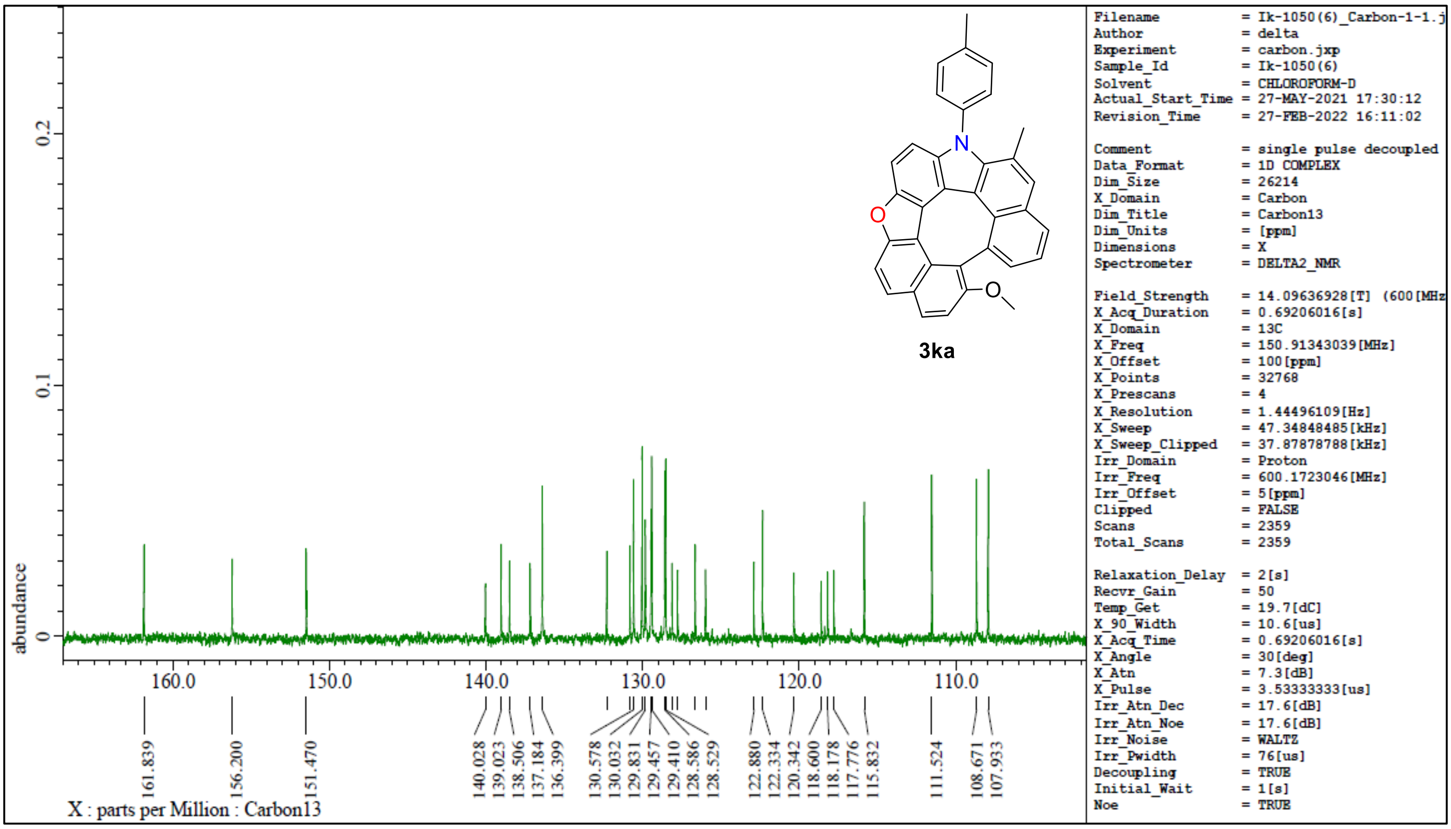

Compound **3ka** (^{13}C NMR, 150 MHz, $CDCl_3$).

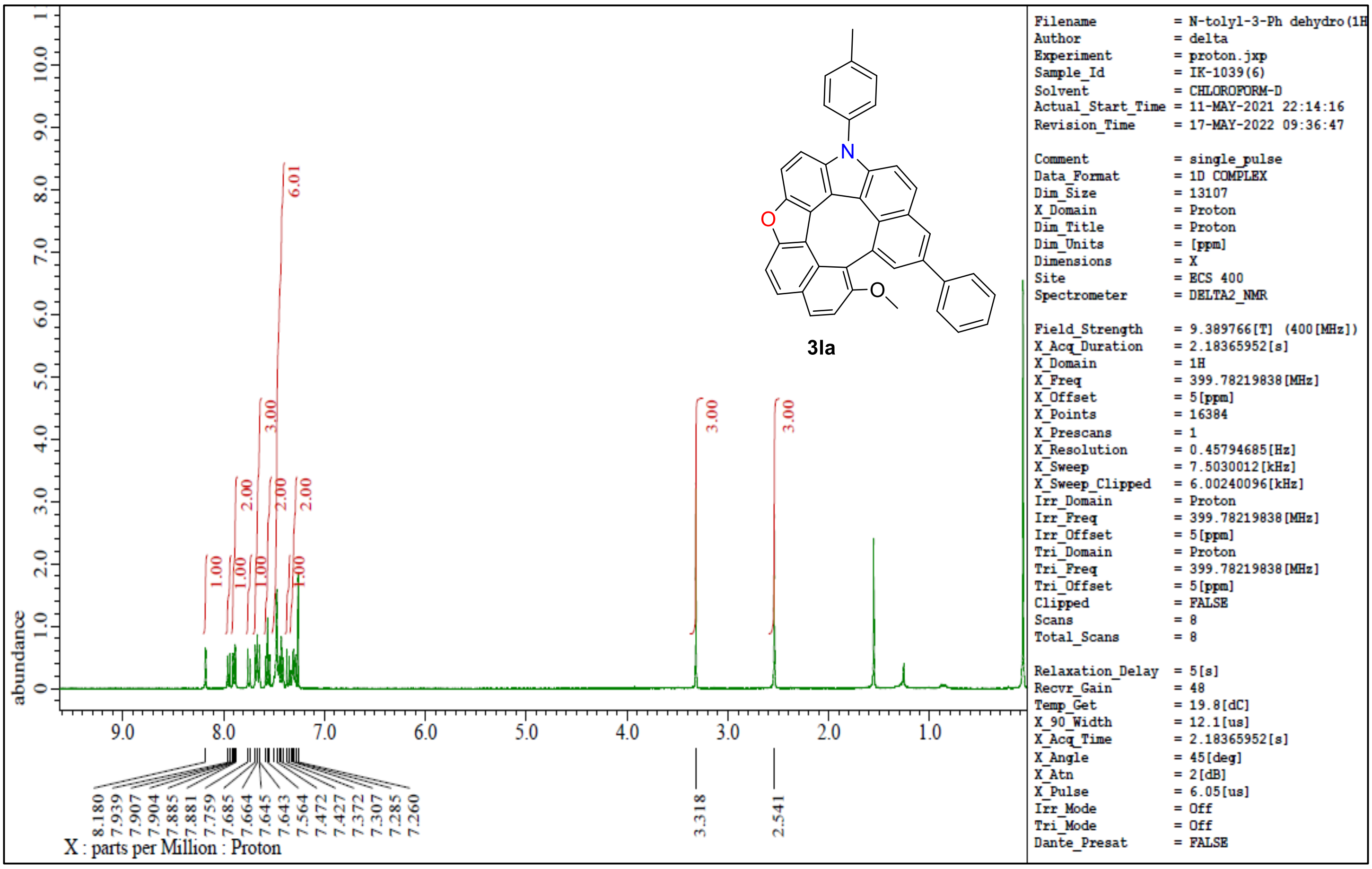

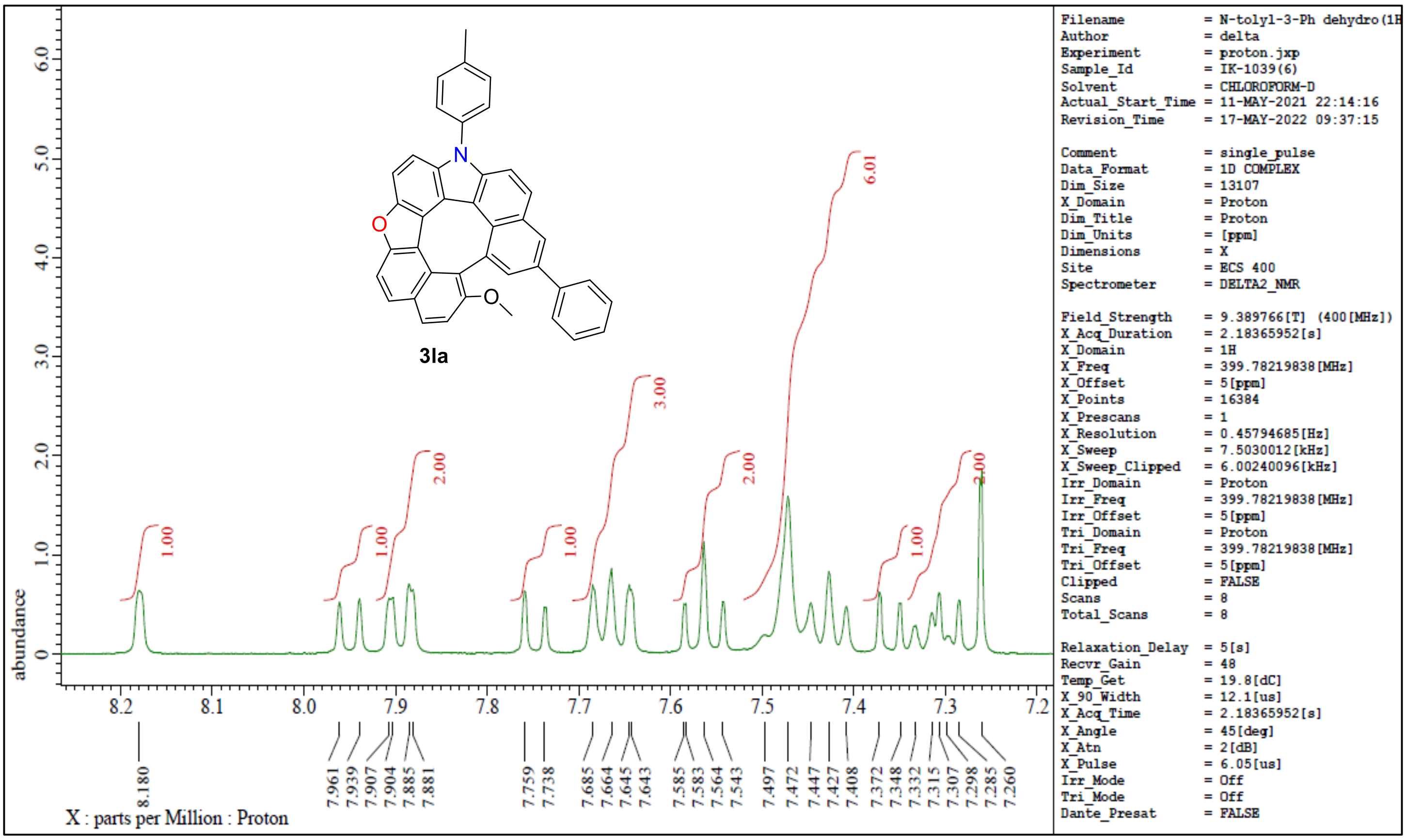

Compound **3la** (^{1}H NMR, 400 MHz, $CDCl_3$).

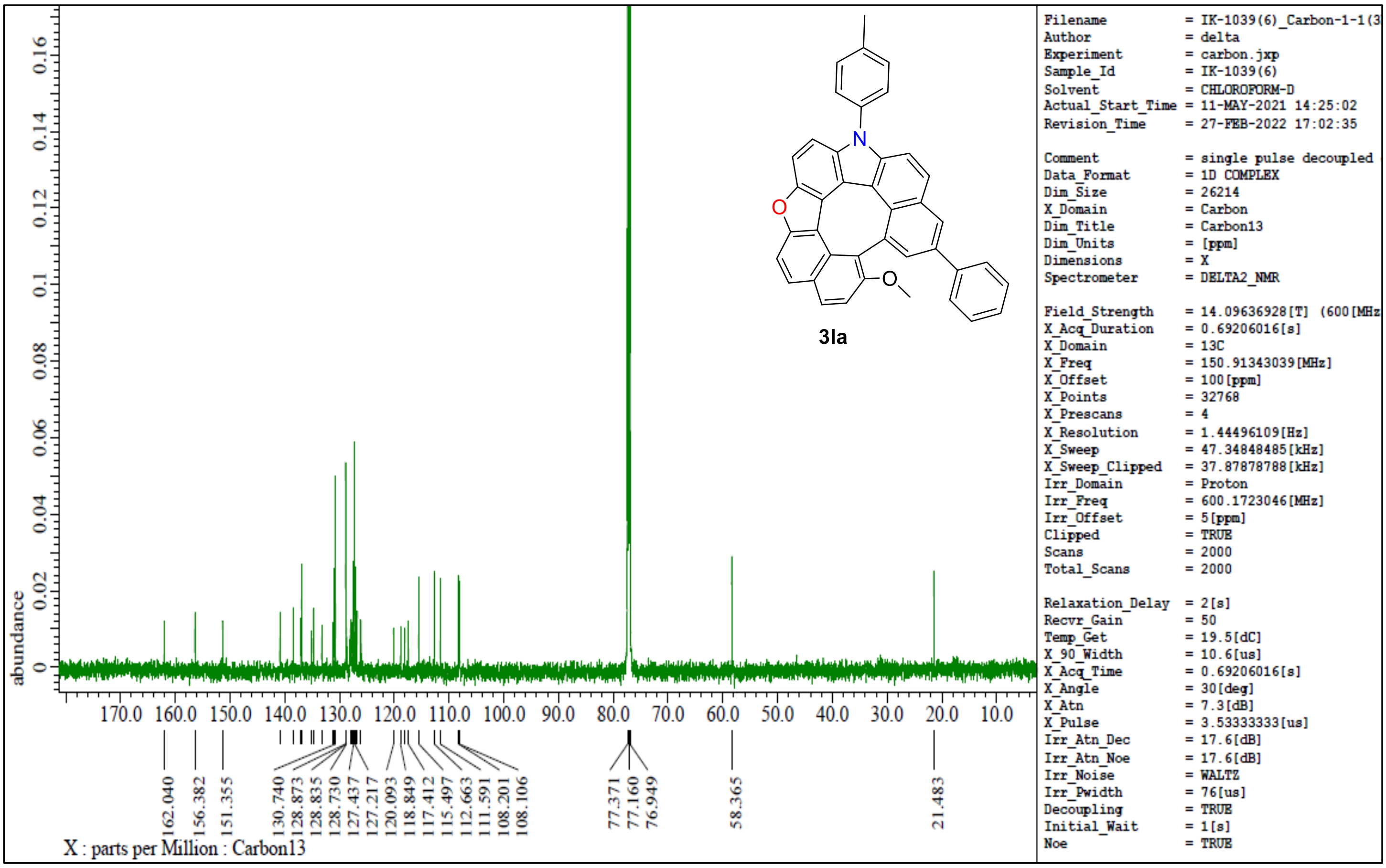

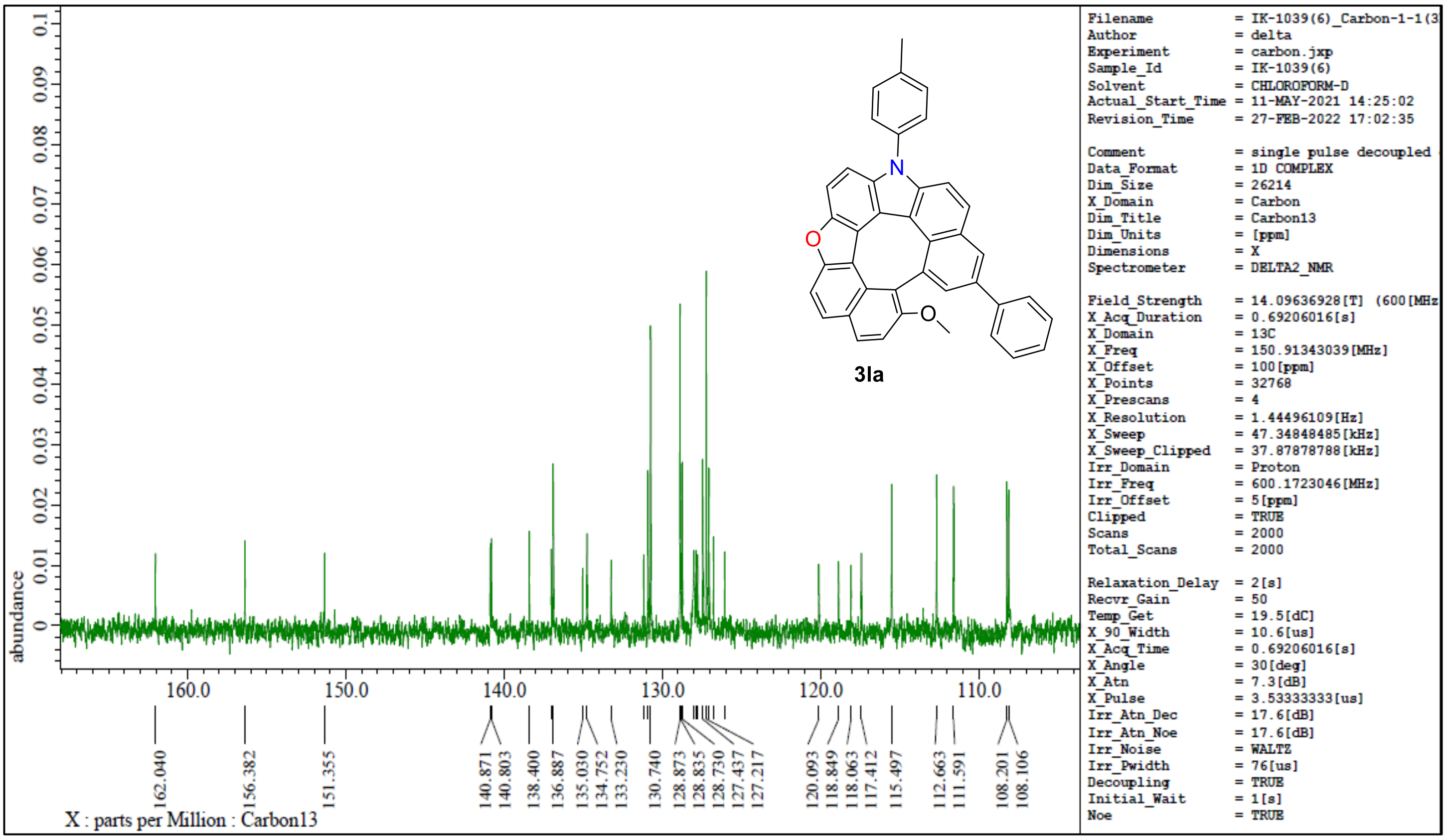

Compound **3la** (^{13}C NMR, 150 MHz, $CDCl_3$).

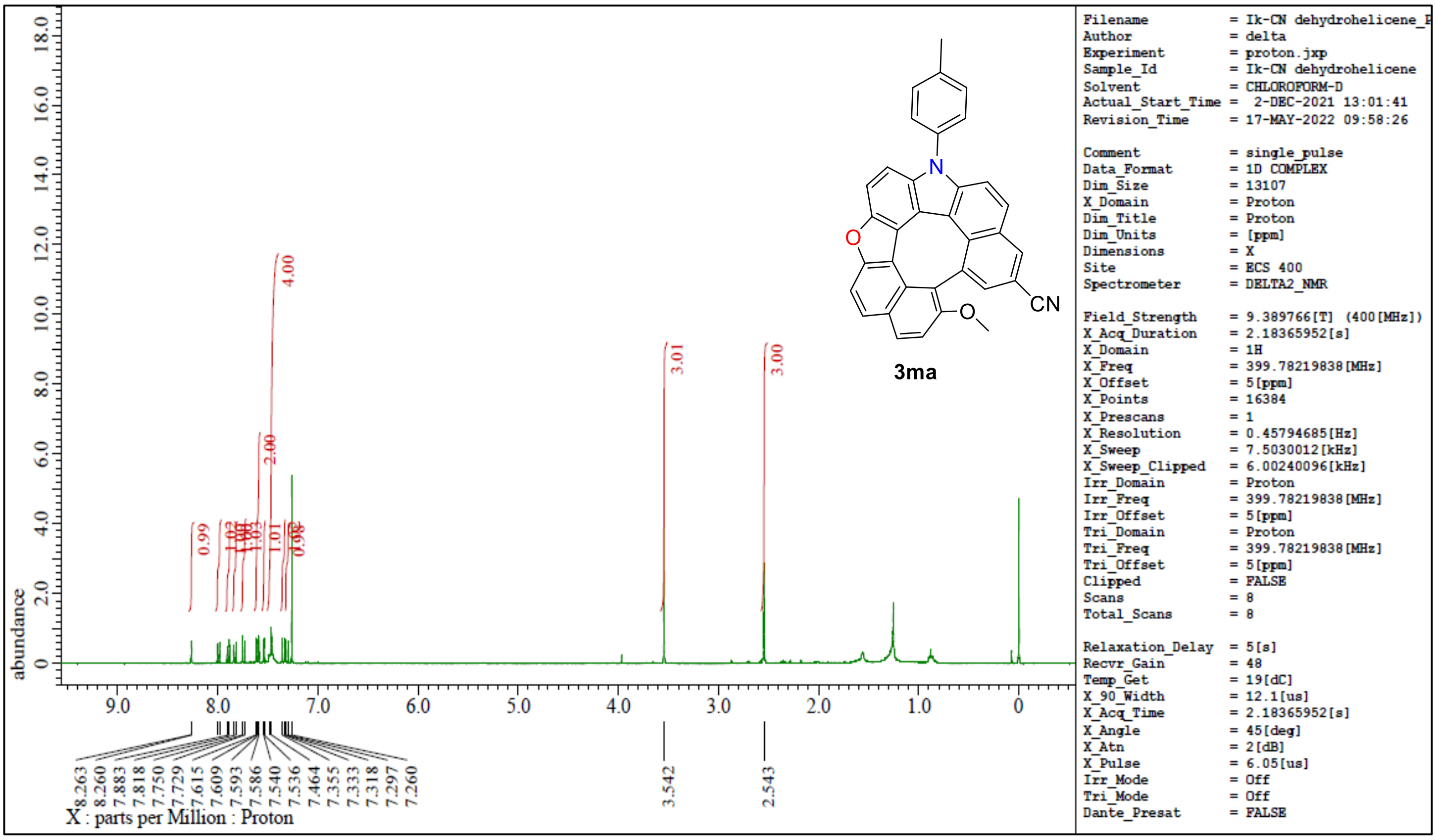

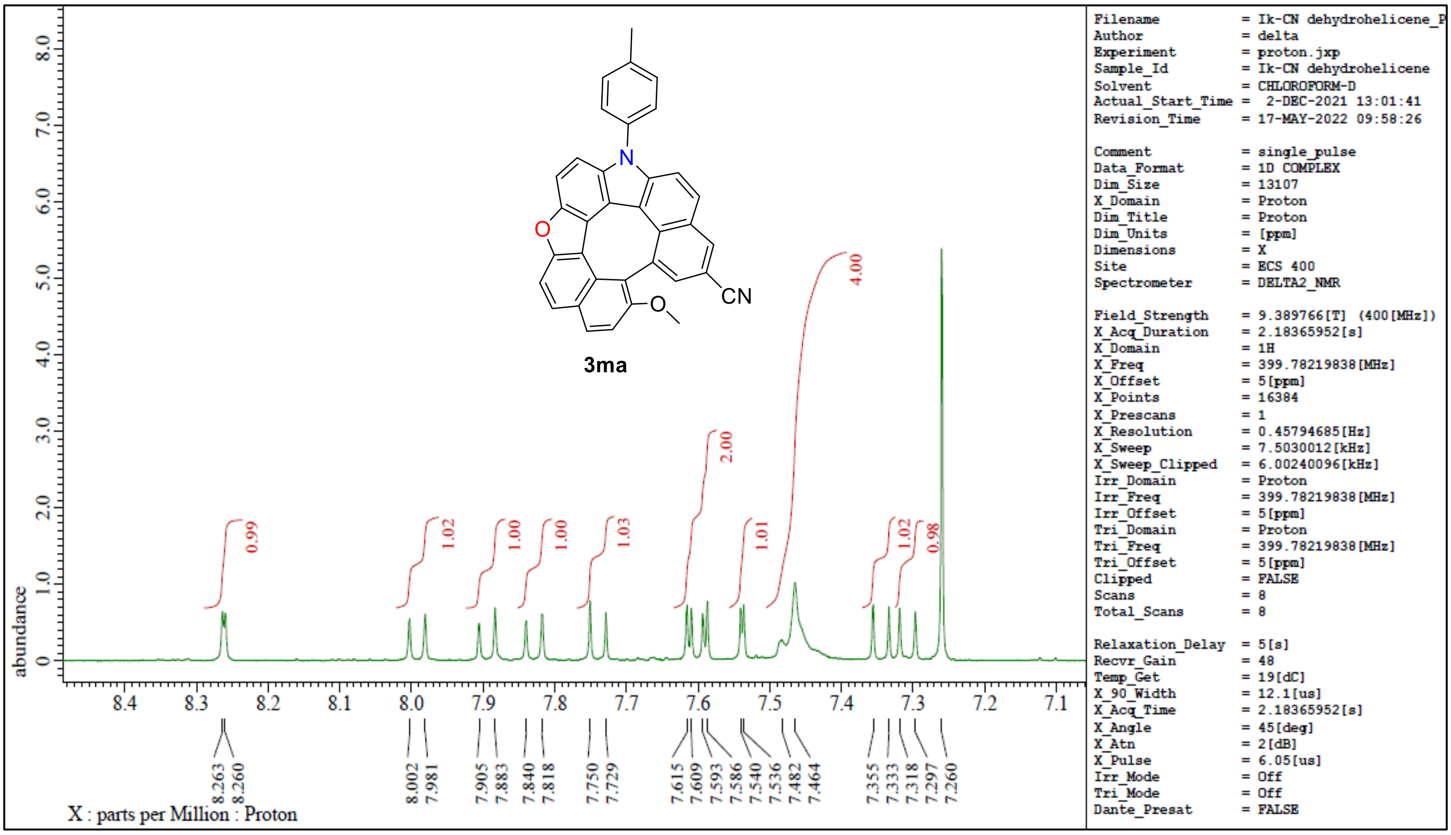

Compound **3ma** (^{1}H NMR, 400 MHz, $CDCl_3$).

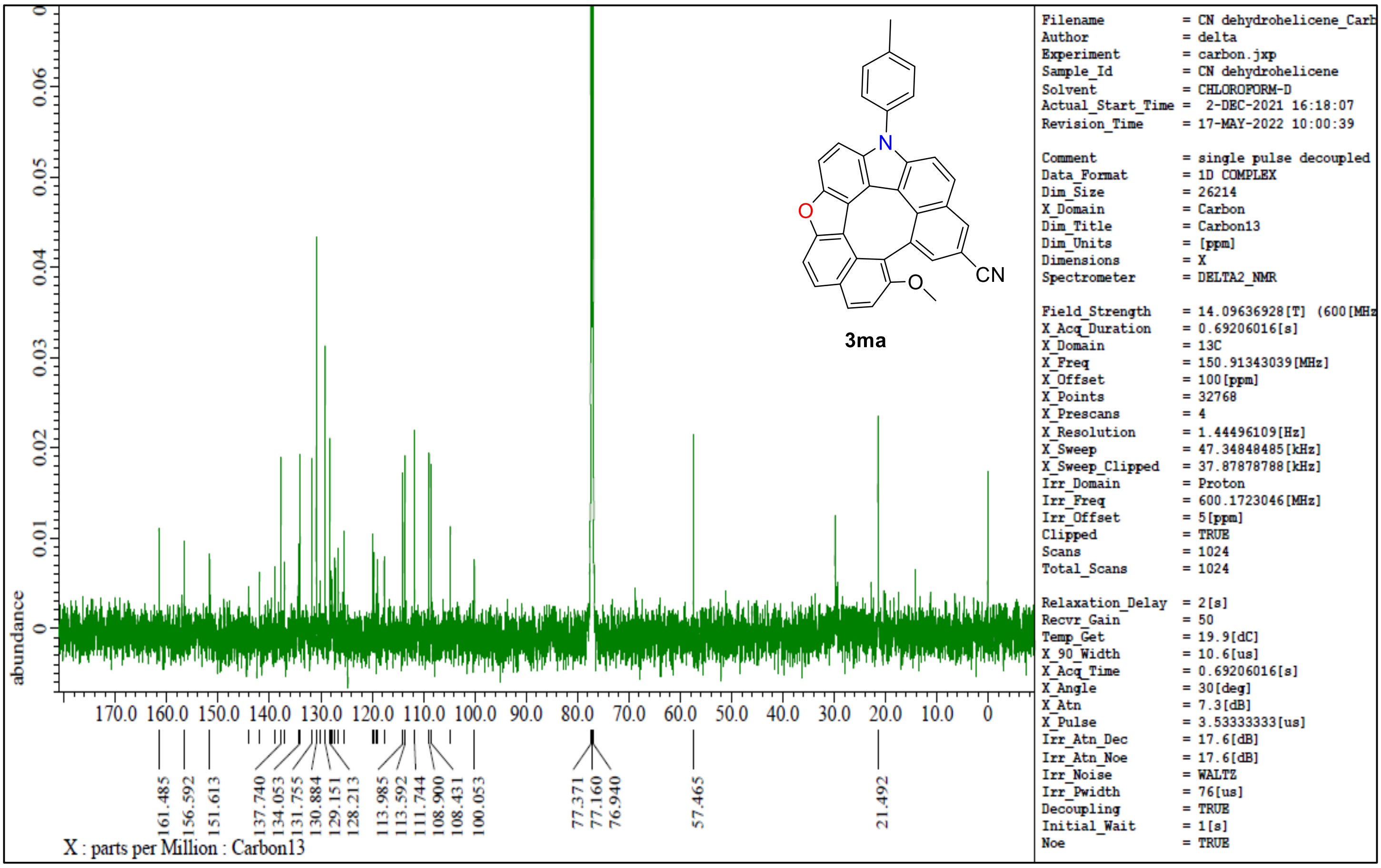

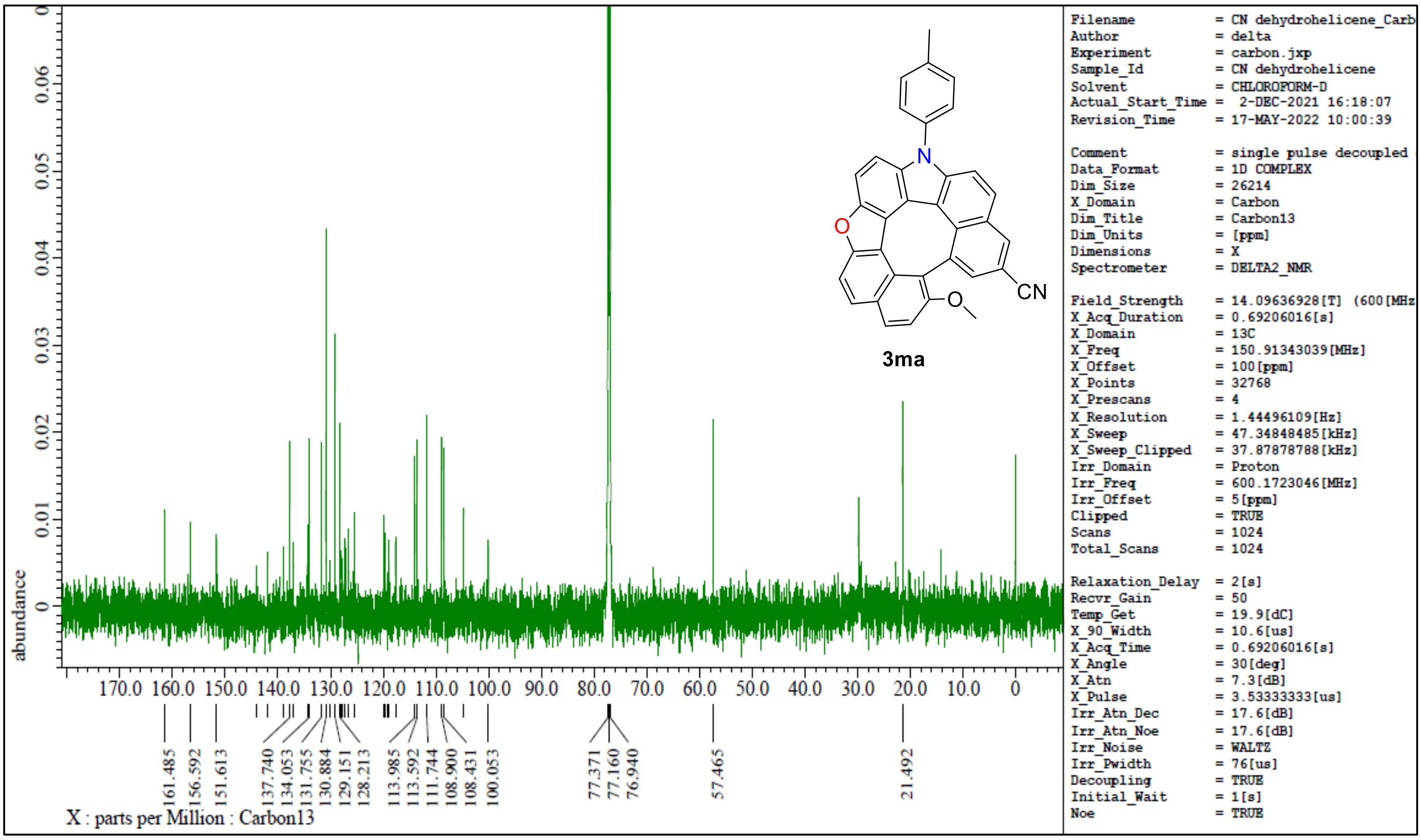

Compound **3ma** (^{13}C NMR, 150 MHz, $CDCl_3$).

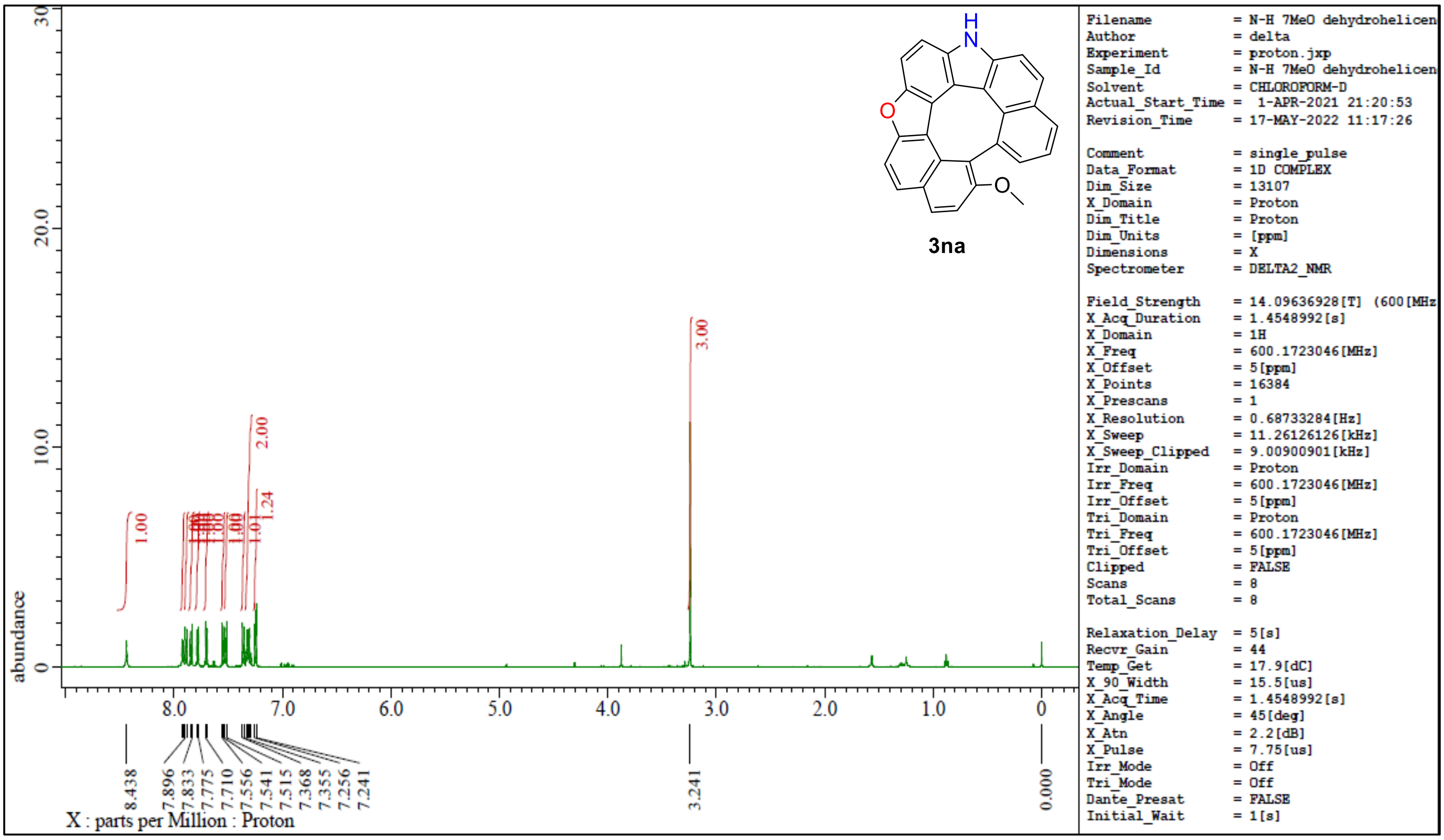

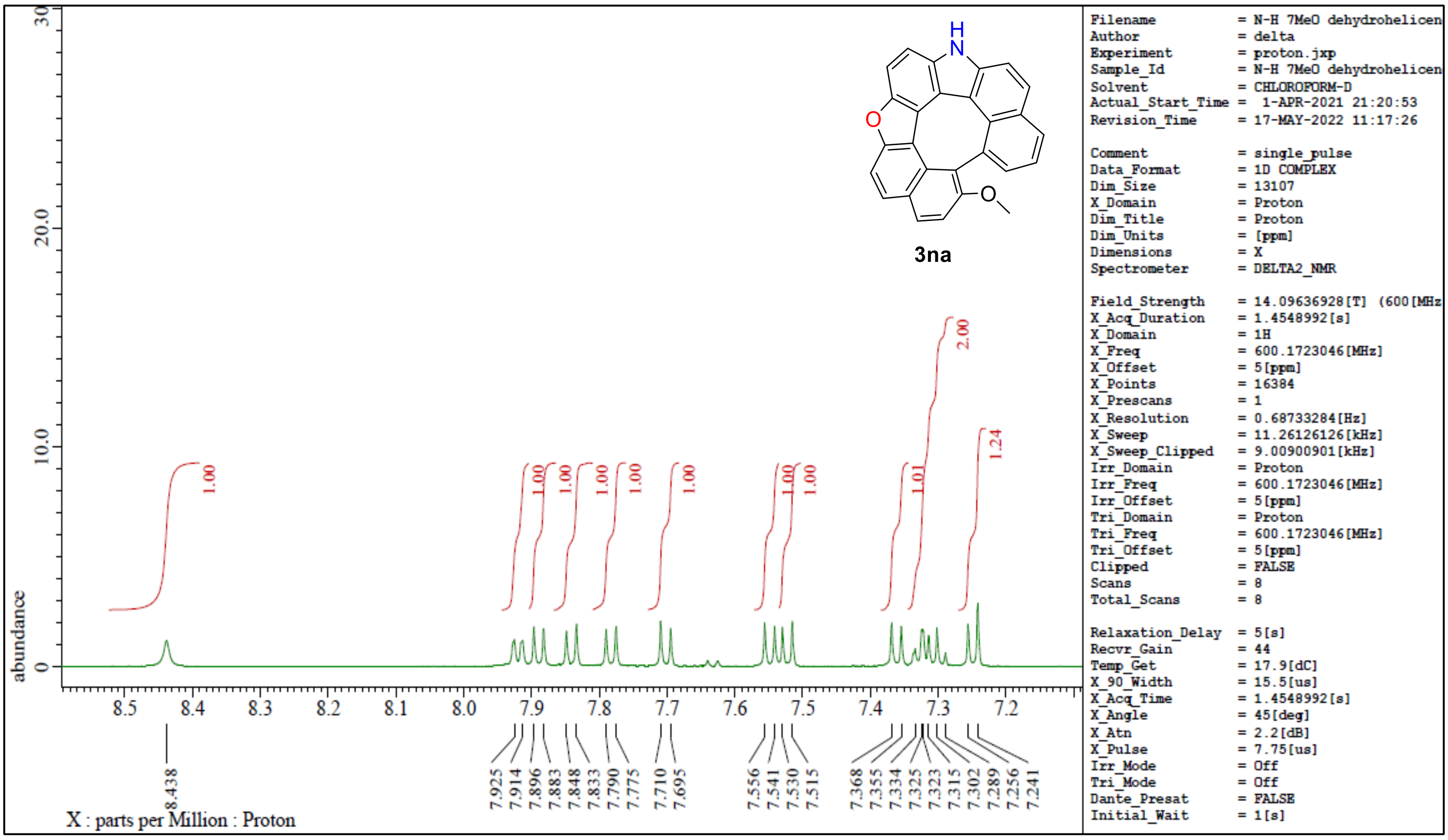

Compound **3na** (^{1}H NMR, 600 MHz, $CDCl_3$).

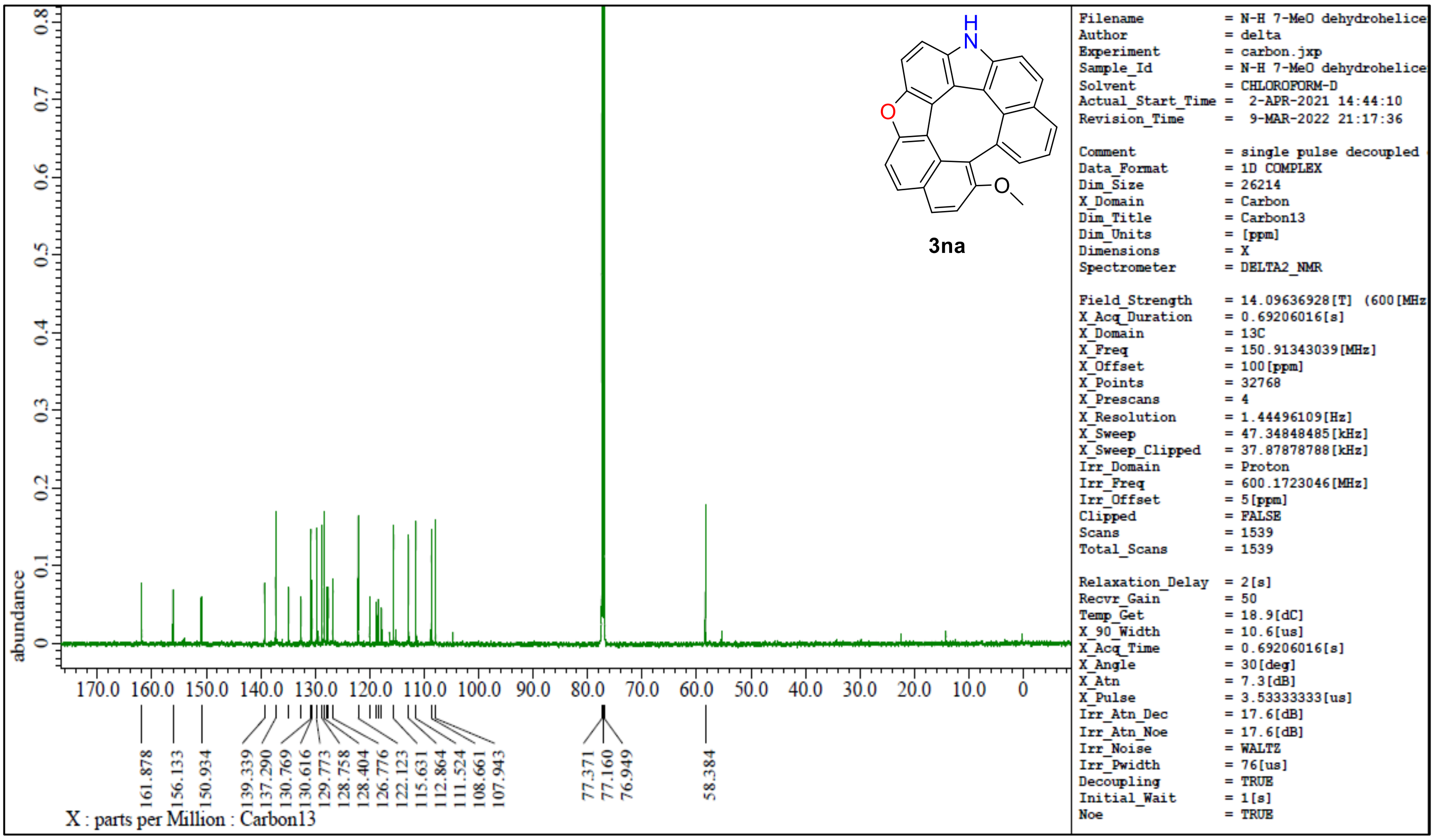

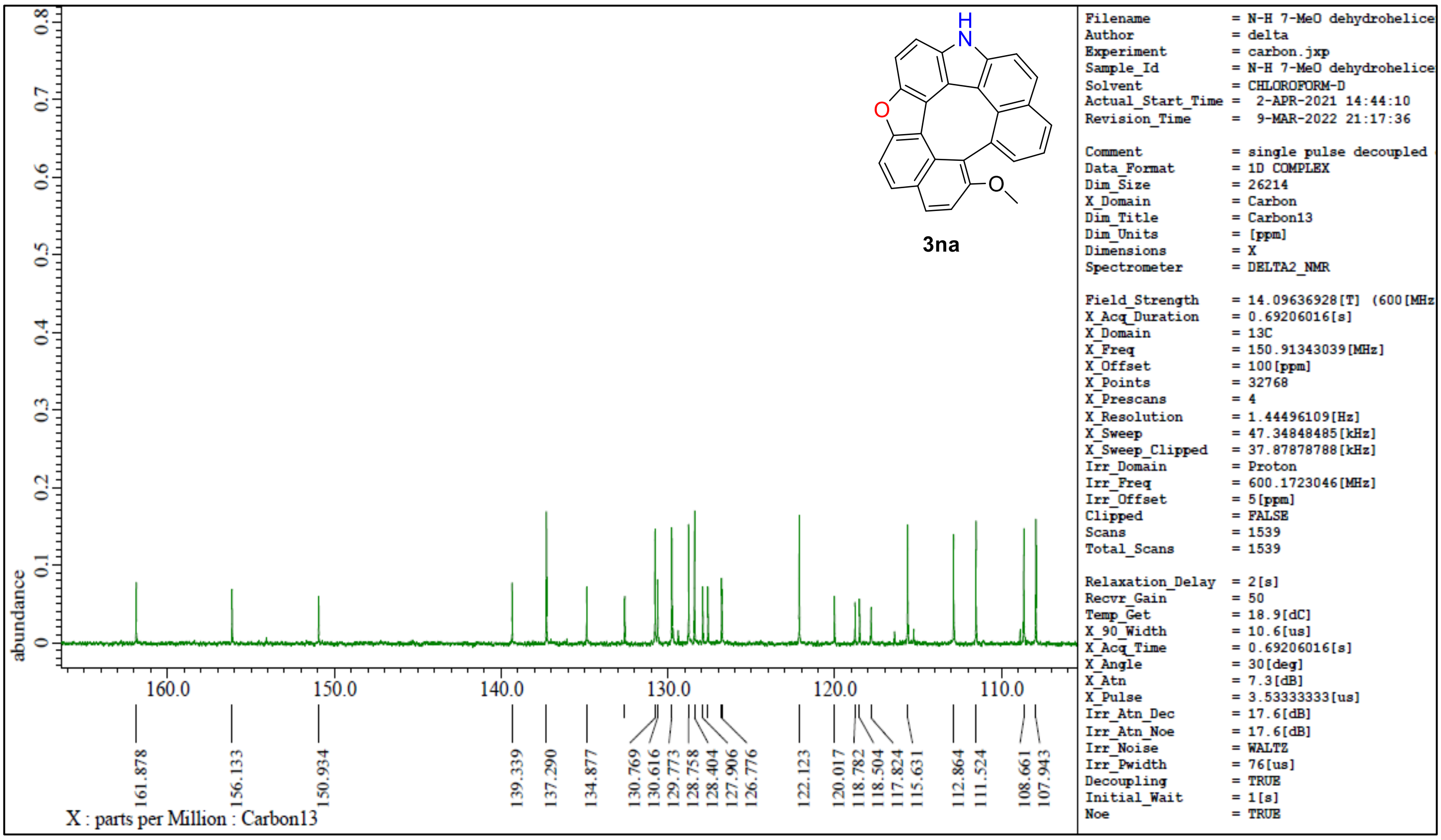

Compound **3na** (^{13}C NMR, 150 MHz, $CDCl_3$).

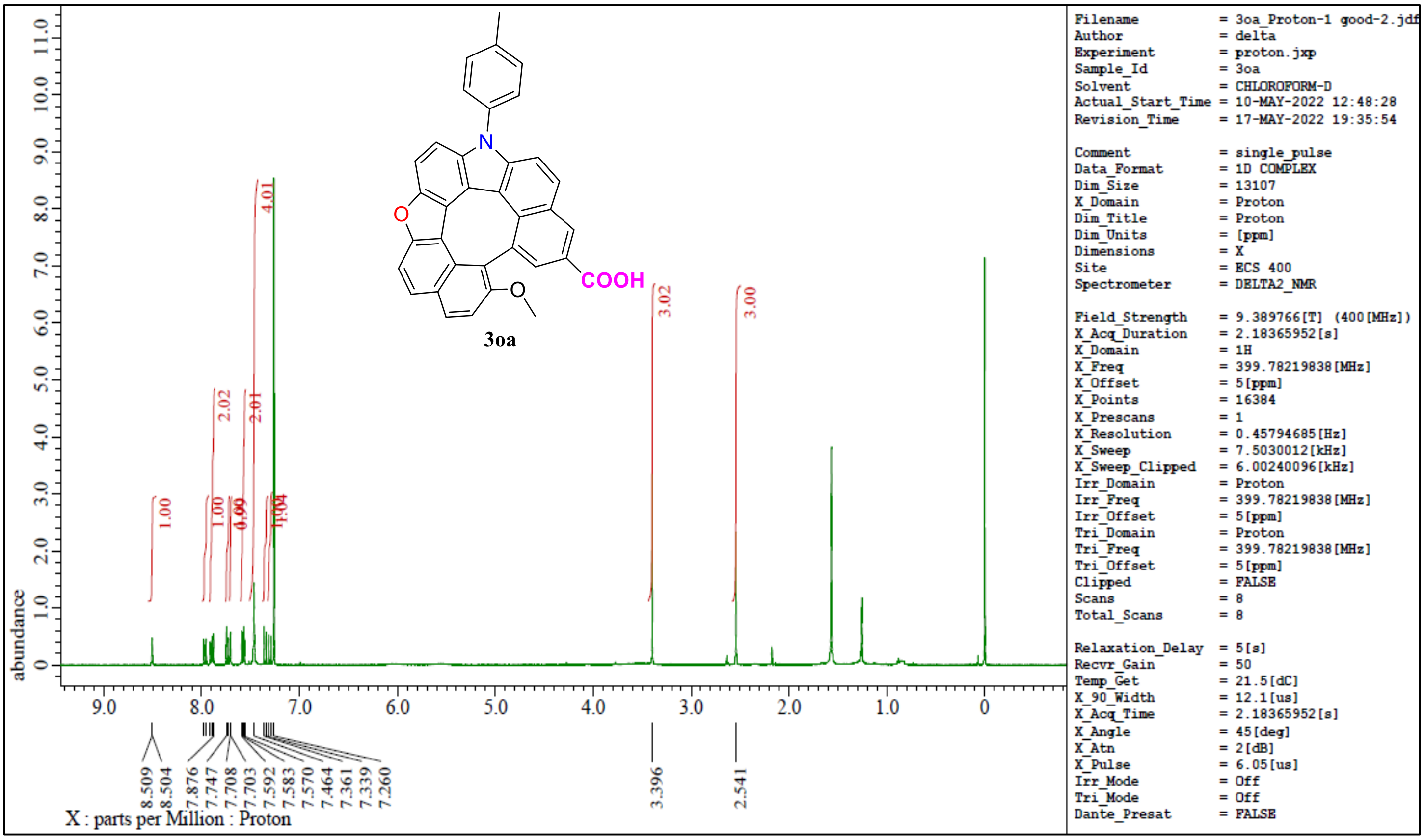

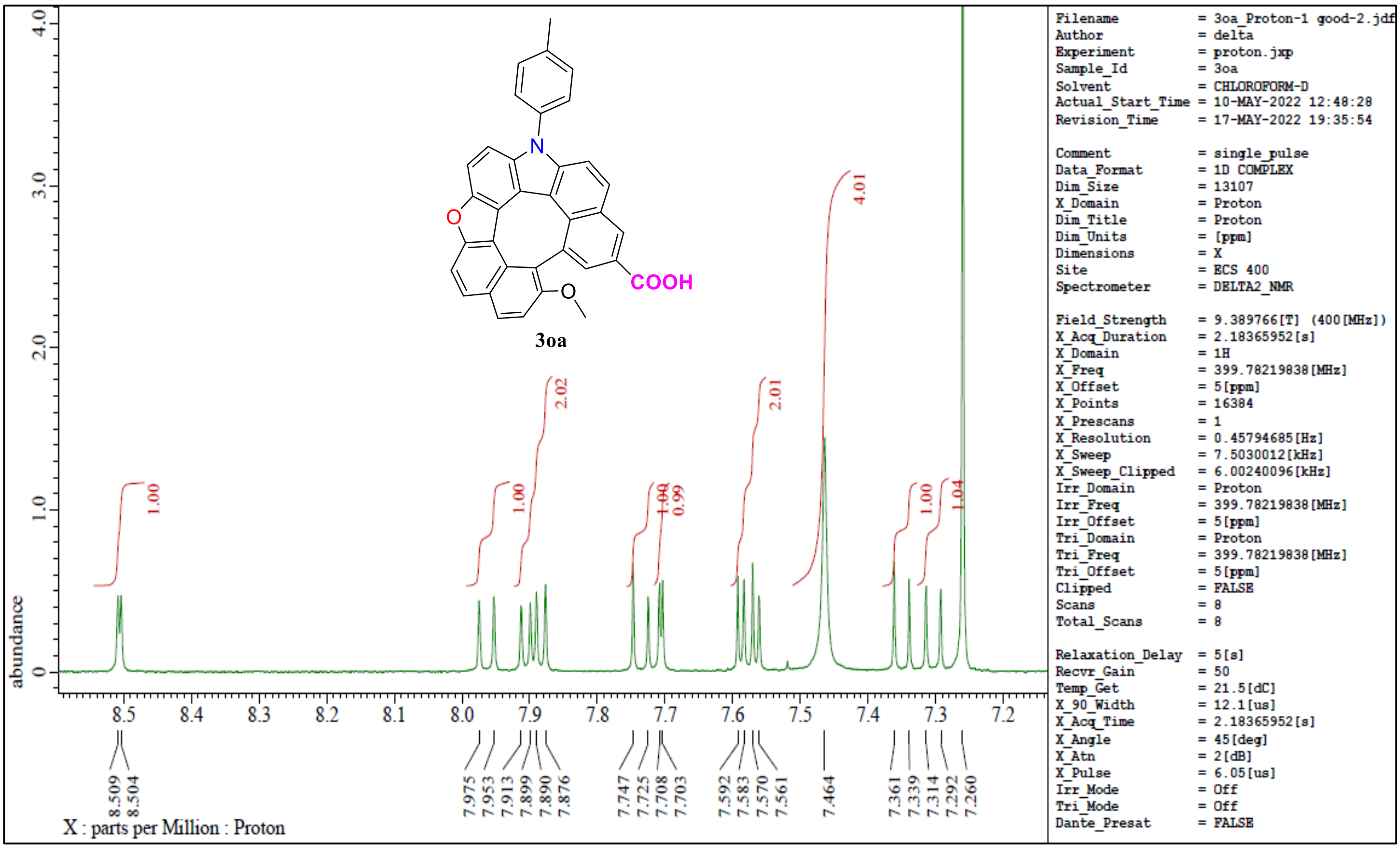

Compound **3oa** (^{1}H NMR, 400 MHz, $CDCl_3$).

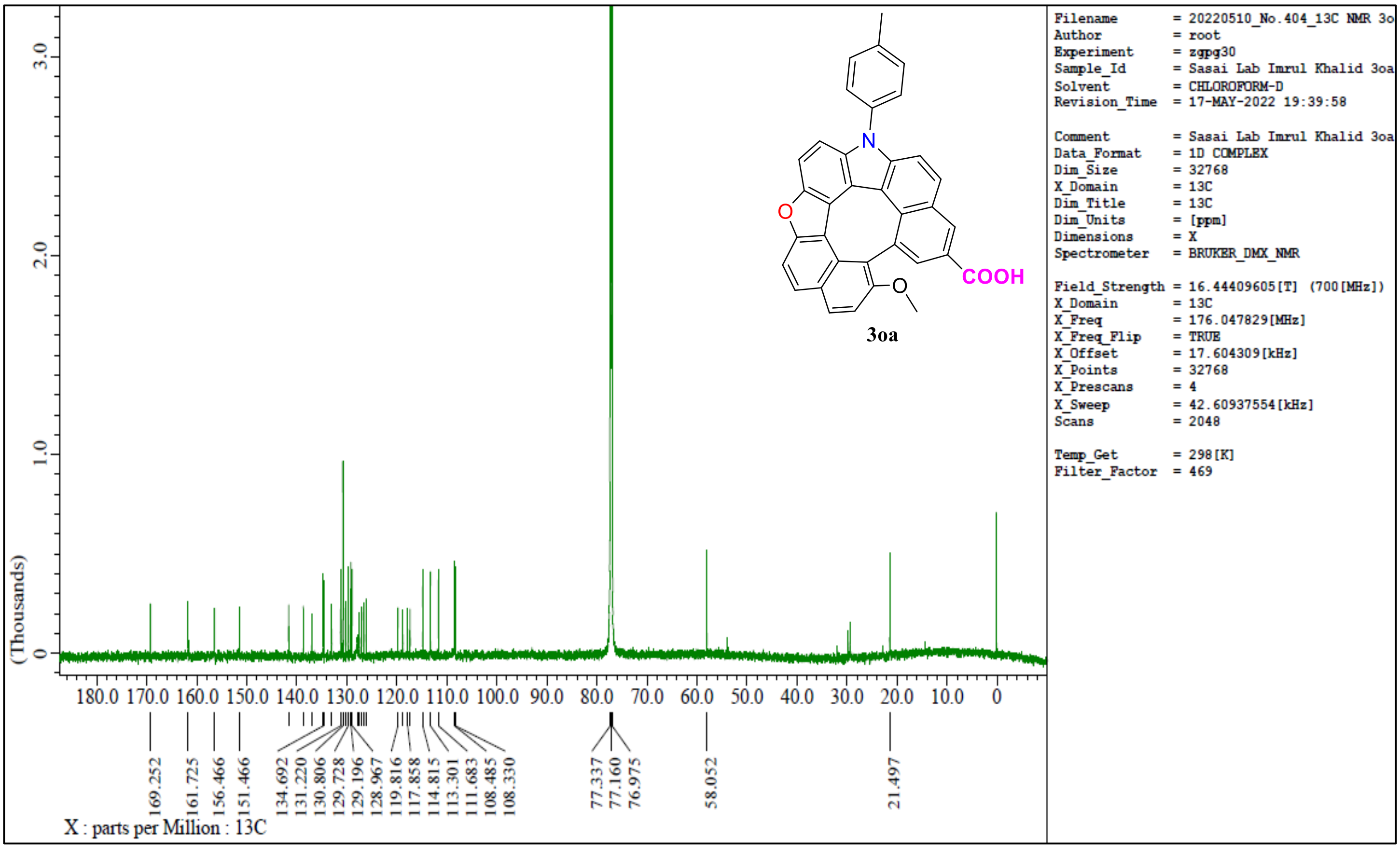

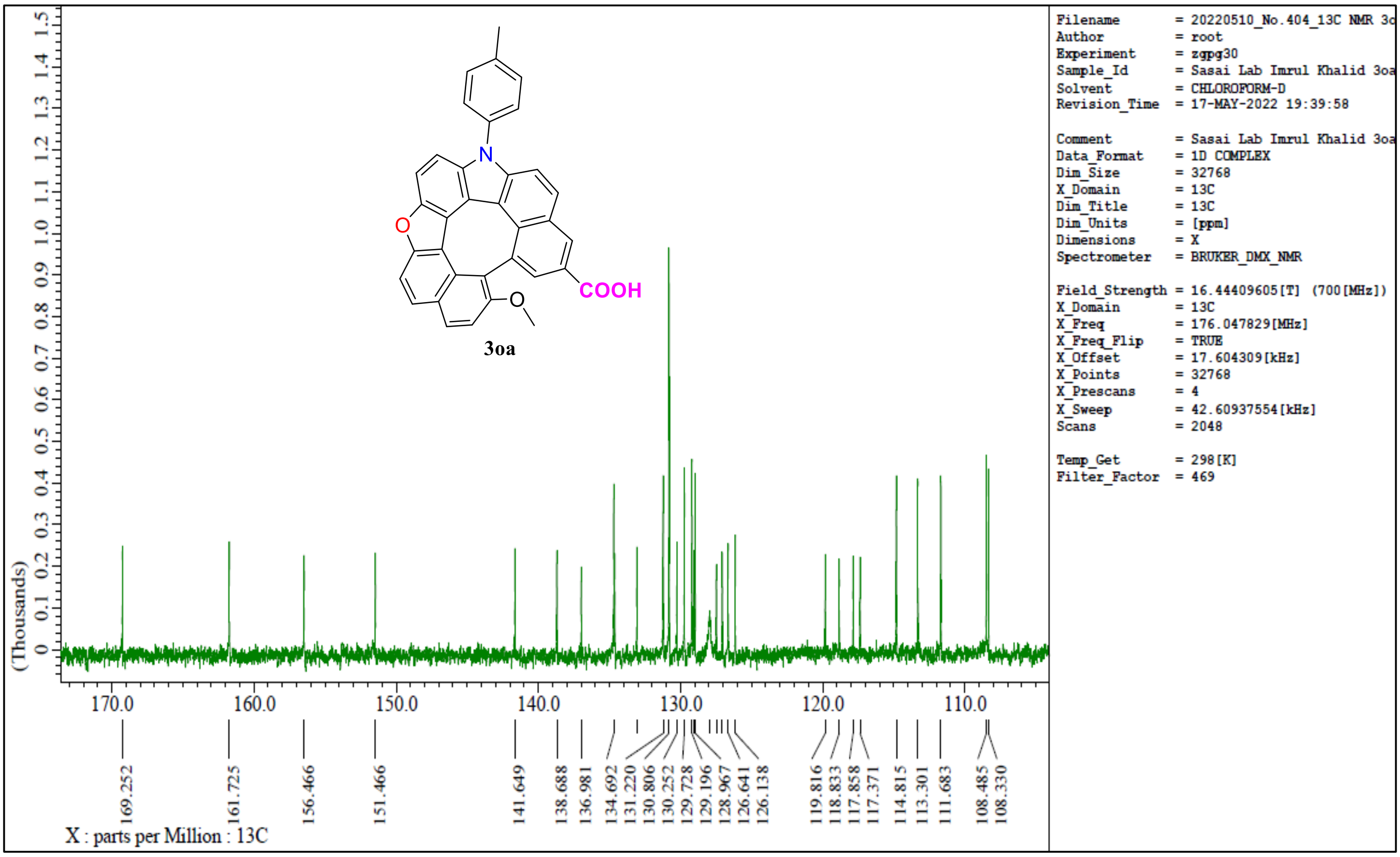

Compound **3oa** (^{13}C NMR, 175 MHz, $CDCl_3$).

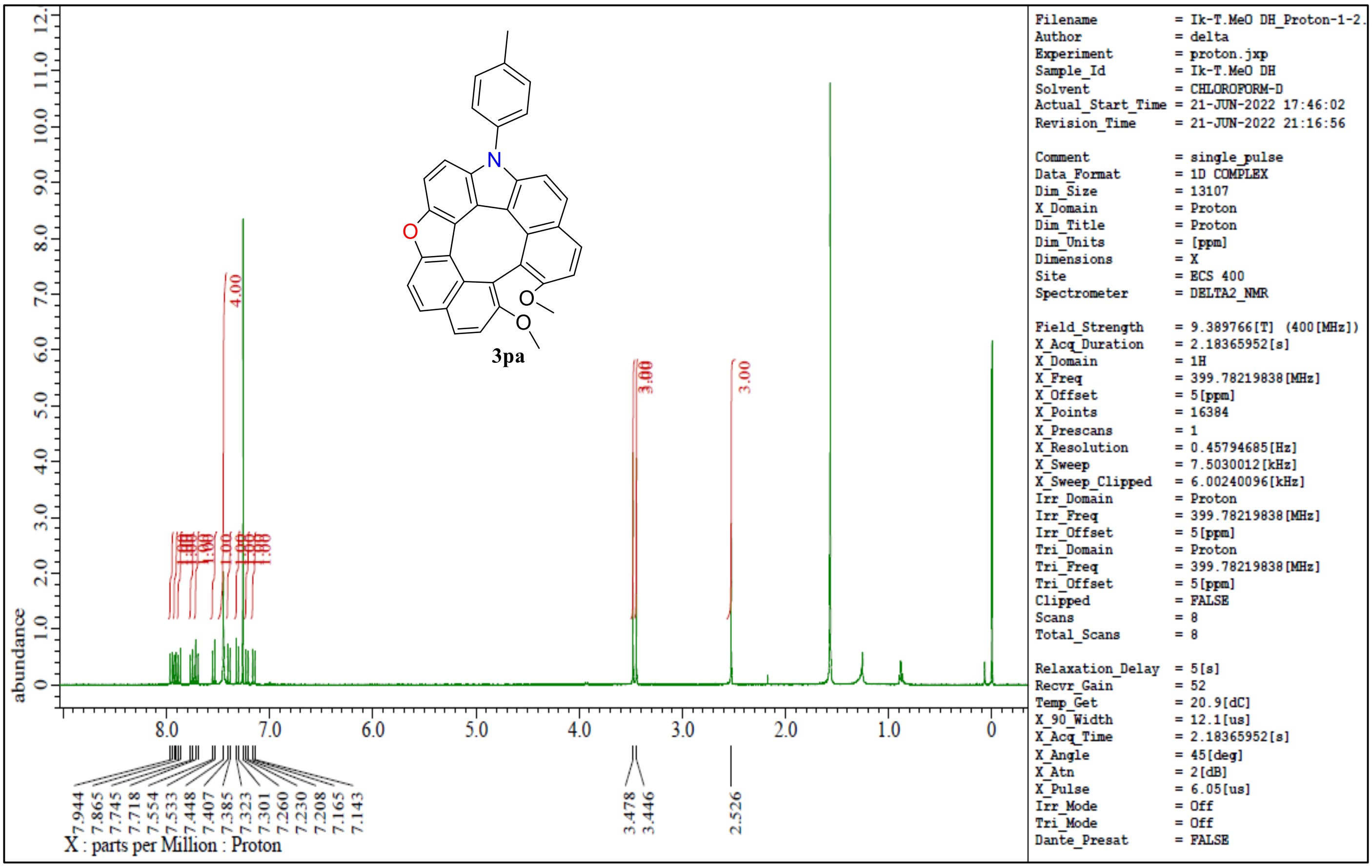

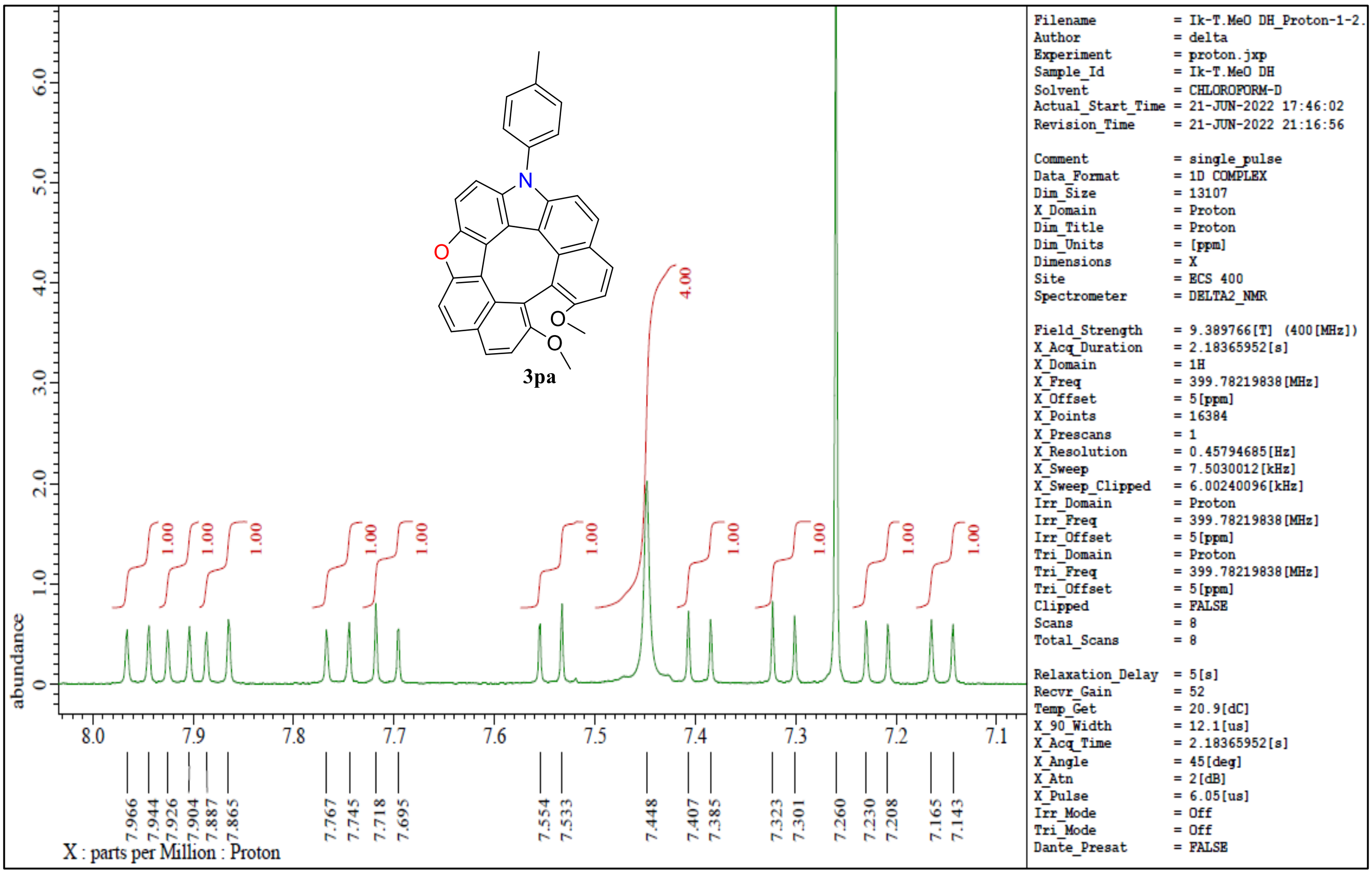

Compound **3pa** (^{1}H NMR, 400 MHz, $CDCl_3$).

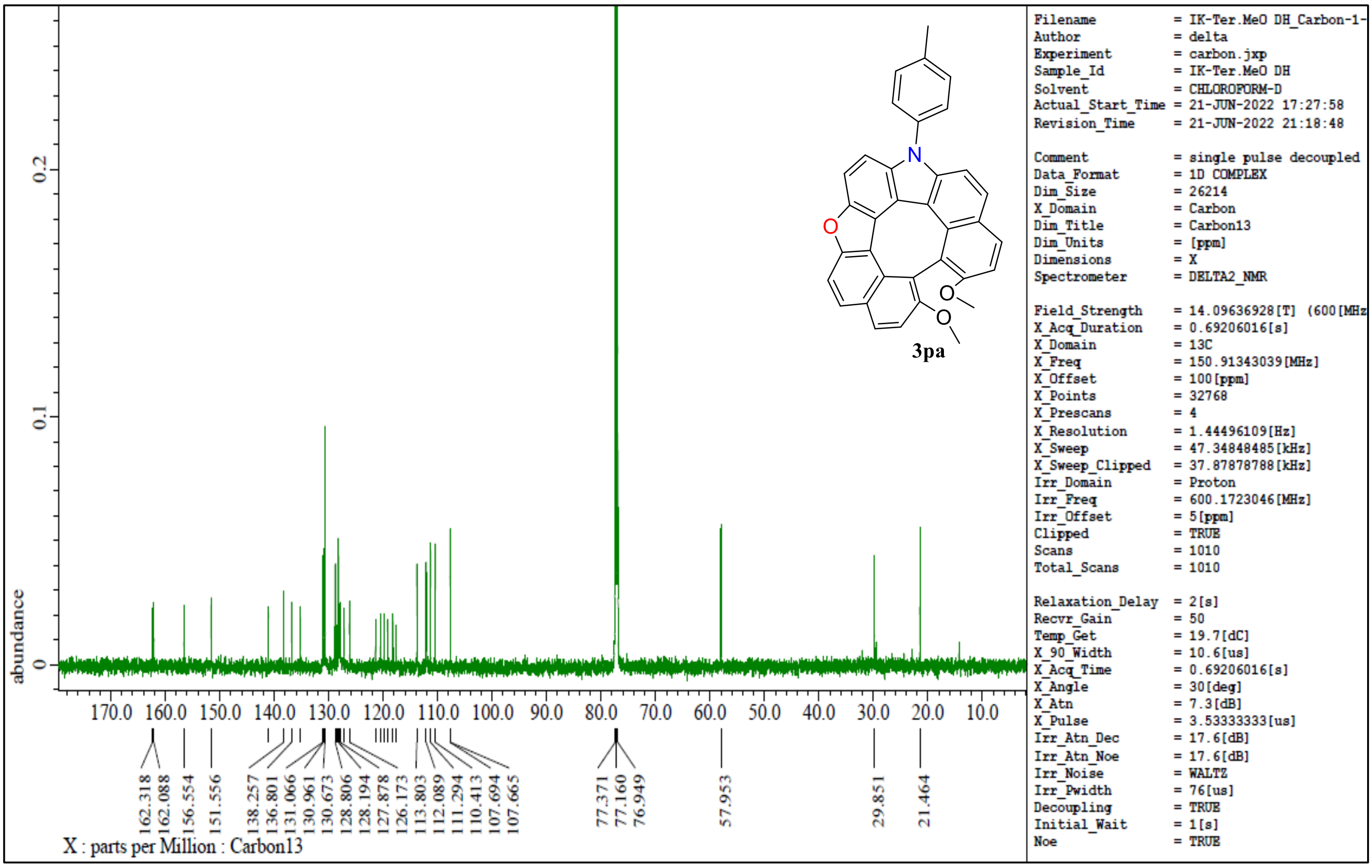

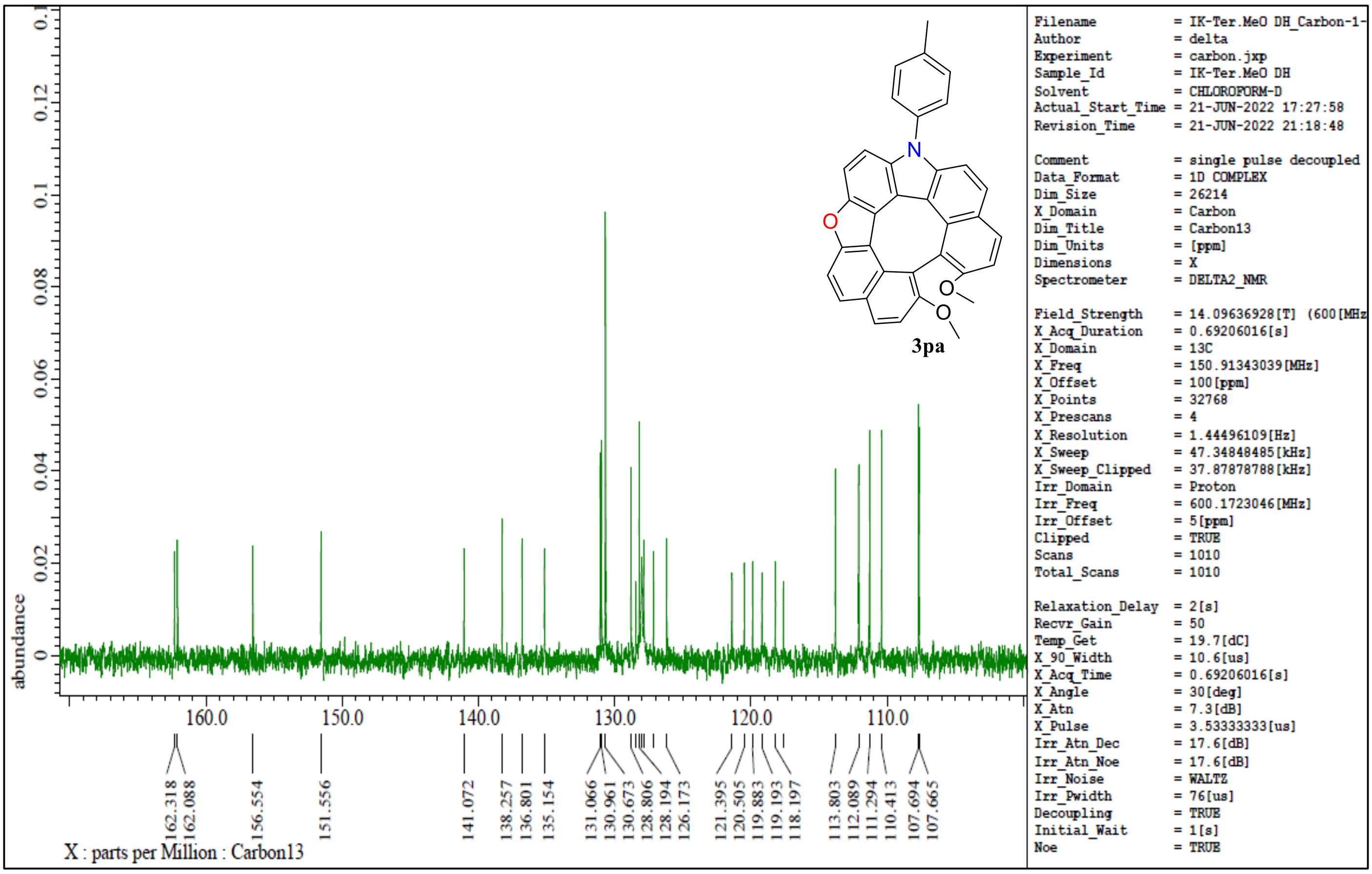

Compound **3pa** (^{13}C NMR, 150 MHz, $CDCl_3$).

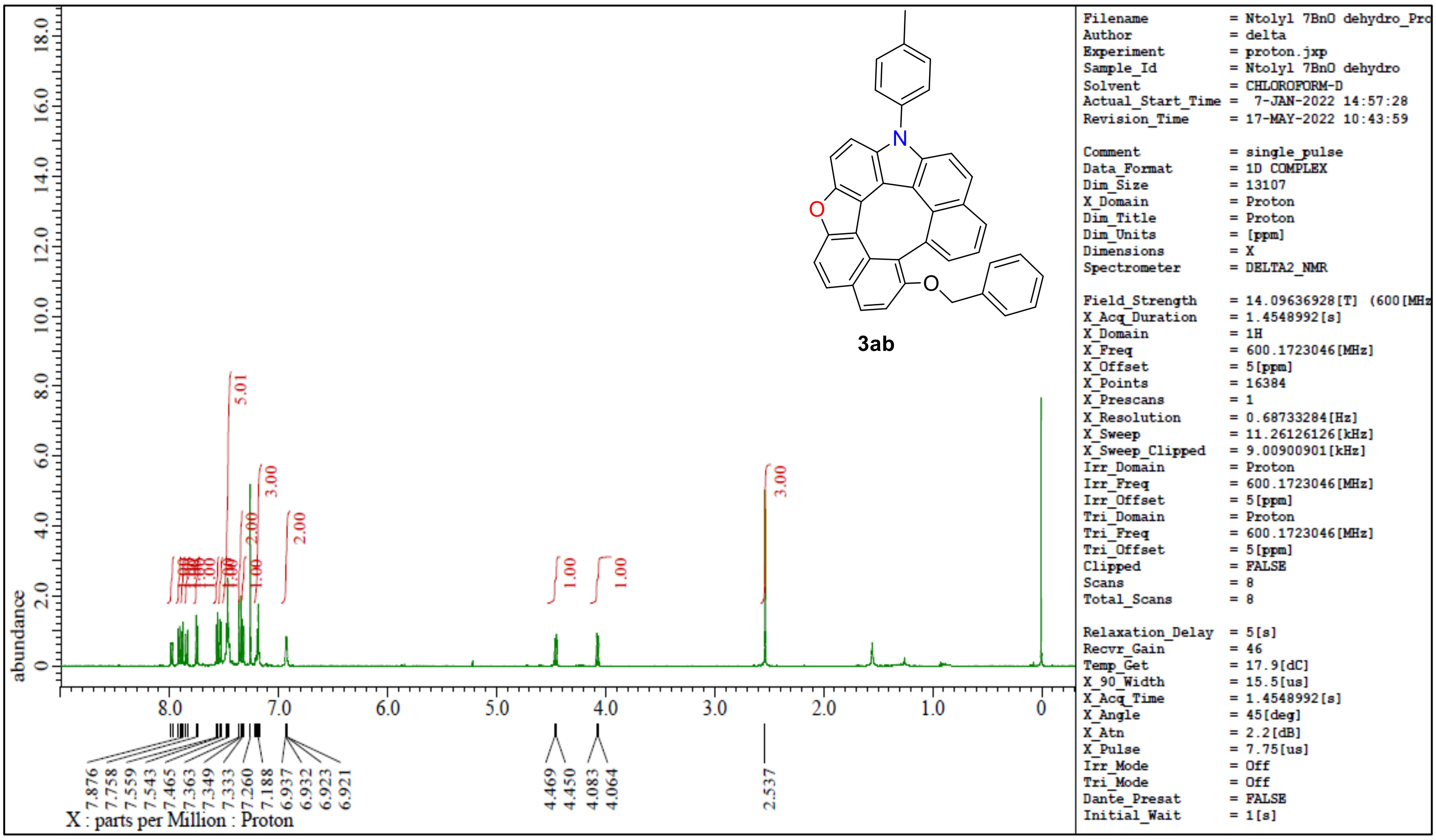

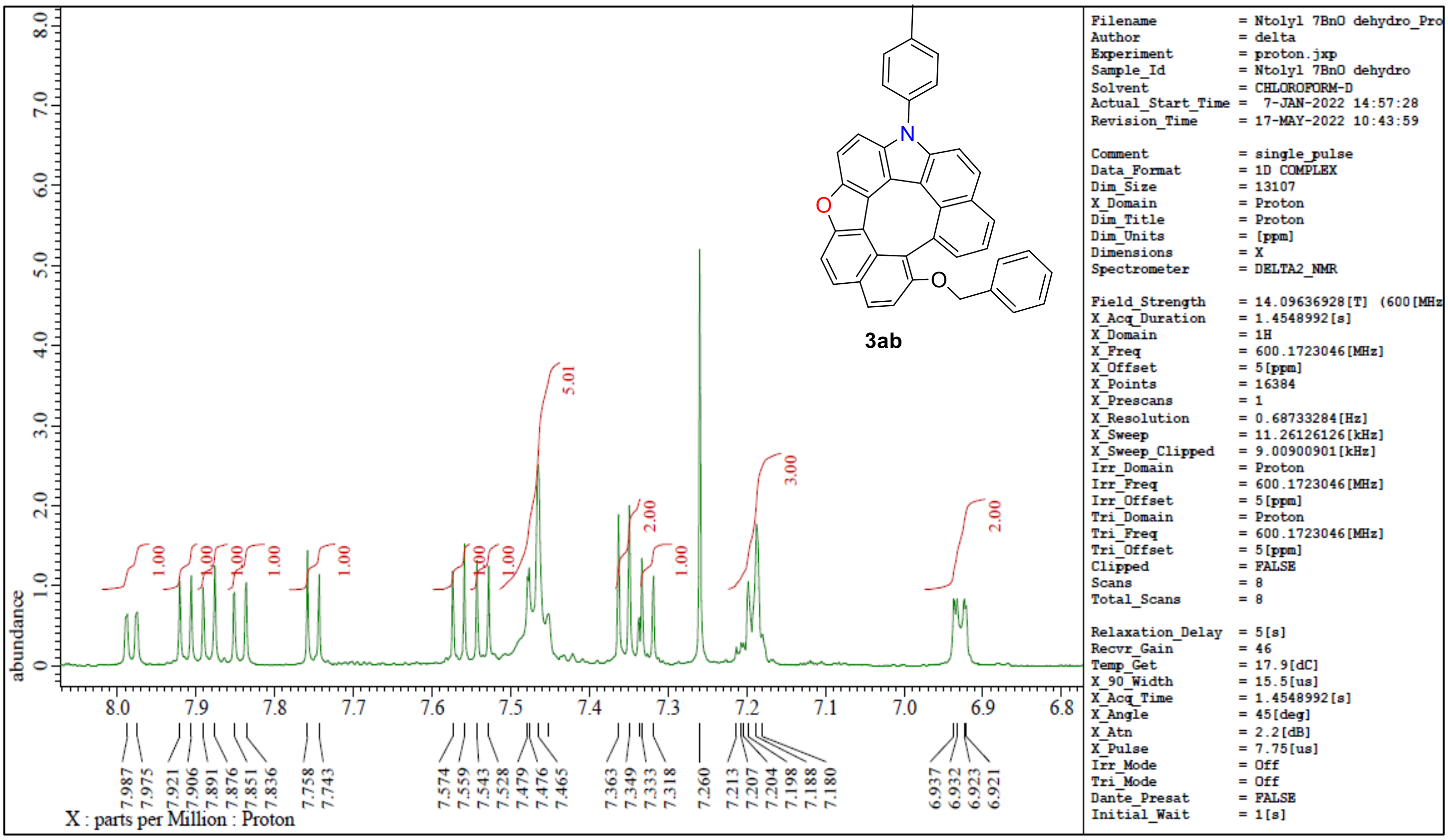

Compound **3ab** (^{1}H NMR, 600 MHz, $CDCl_3$).

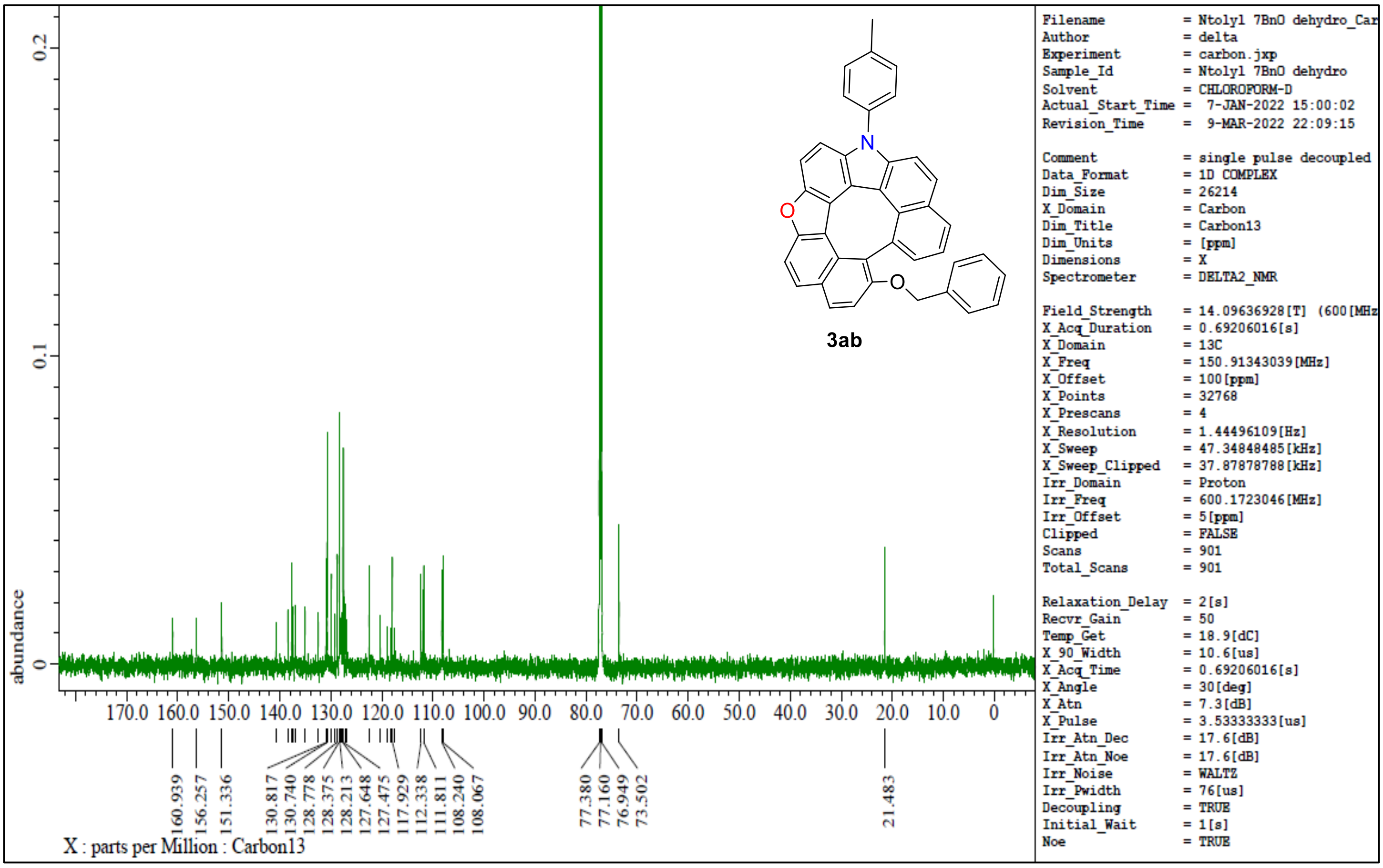

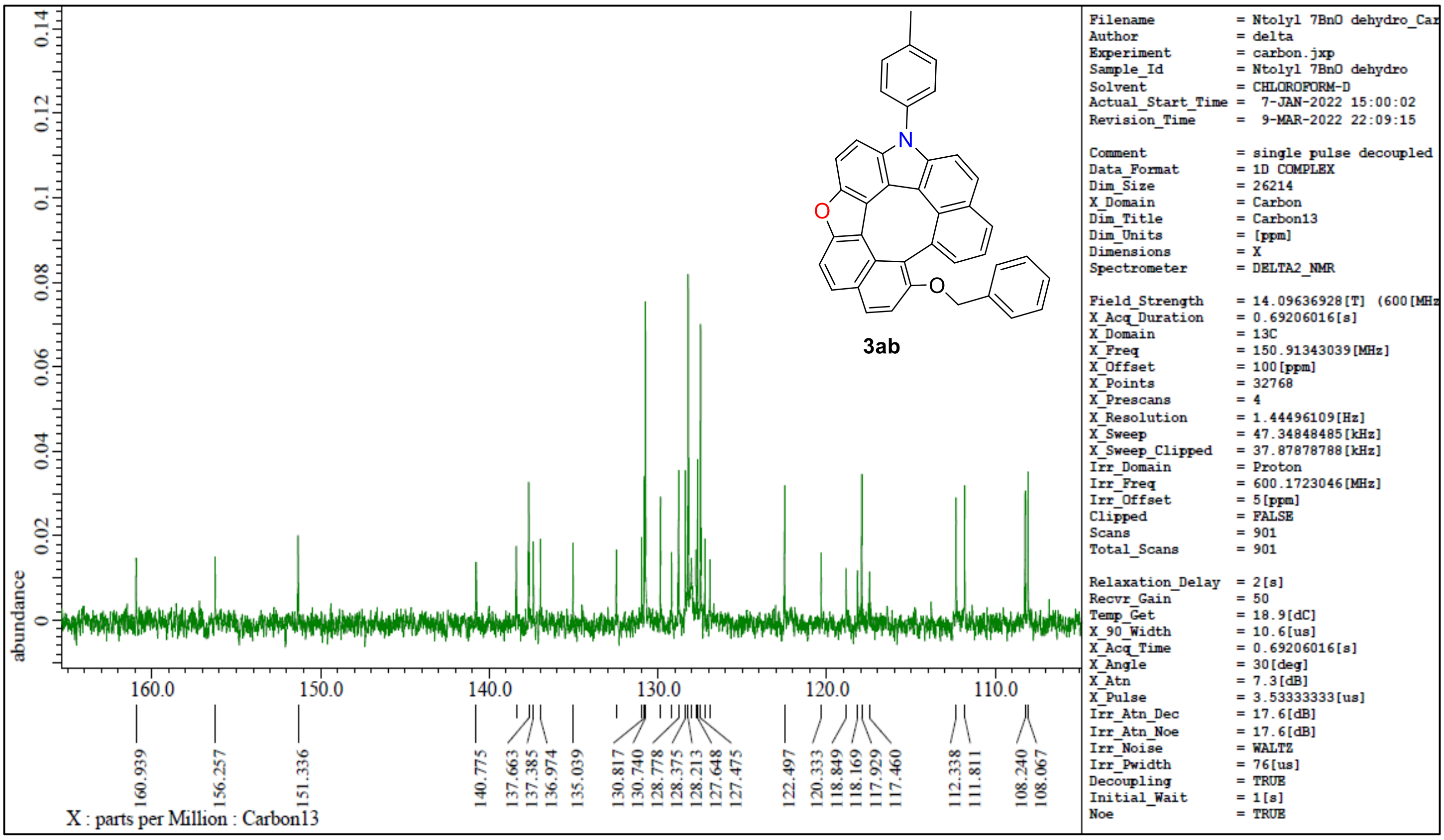

Compound **3ab** (^{13}C NMR, 150 MHz, $CDCl_3$).

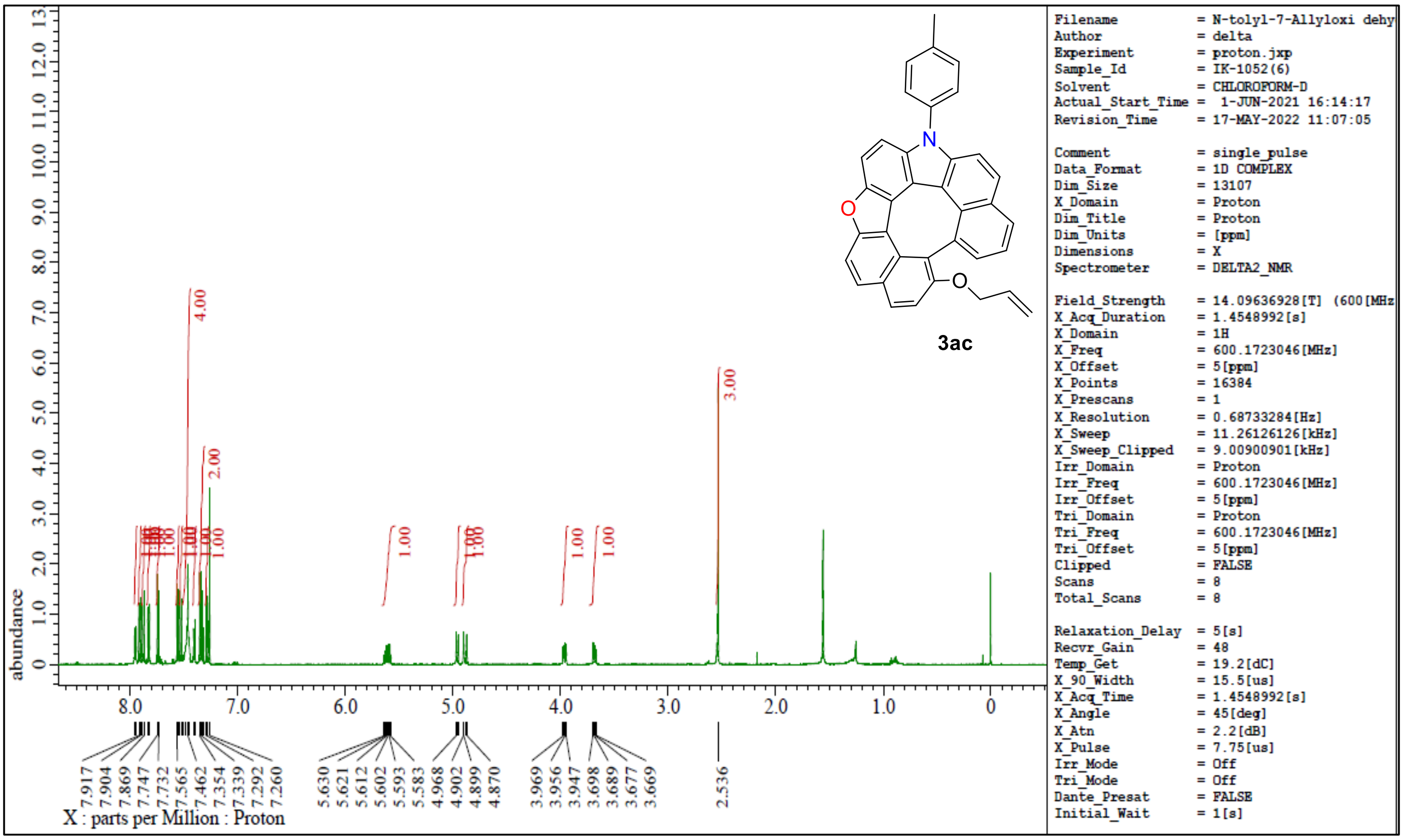

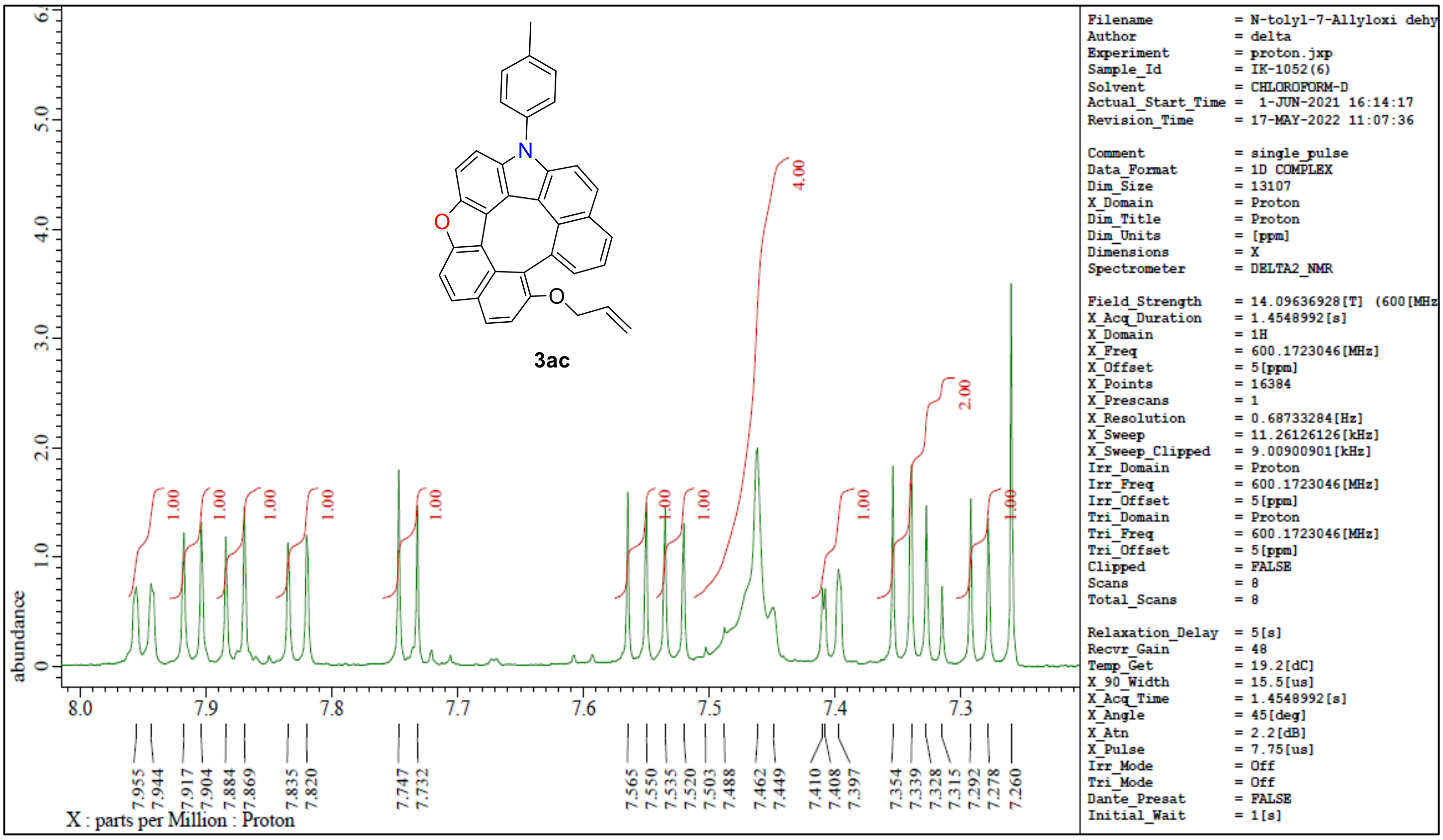

Compound **3ac** (^{1}H NMR, 600 MHz, $CDCl_3$).

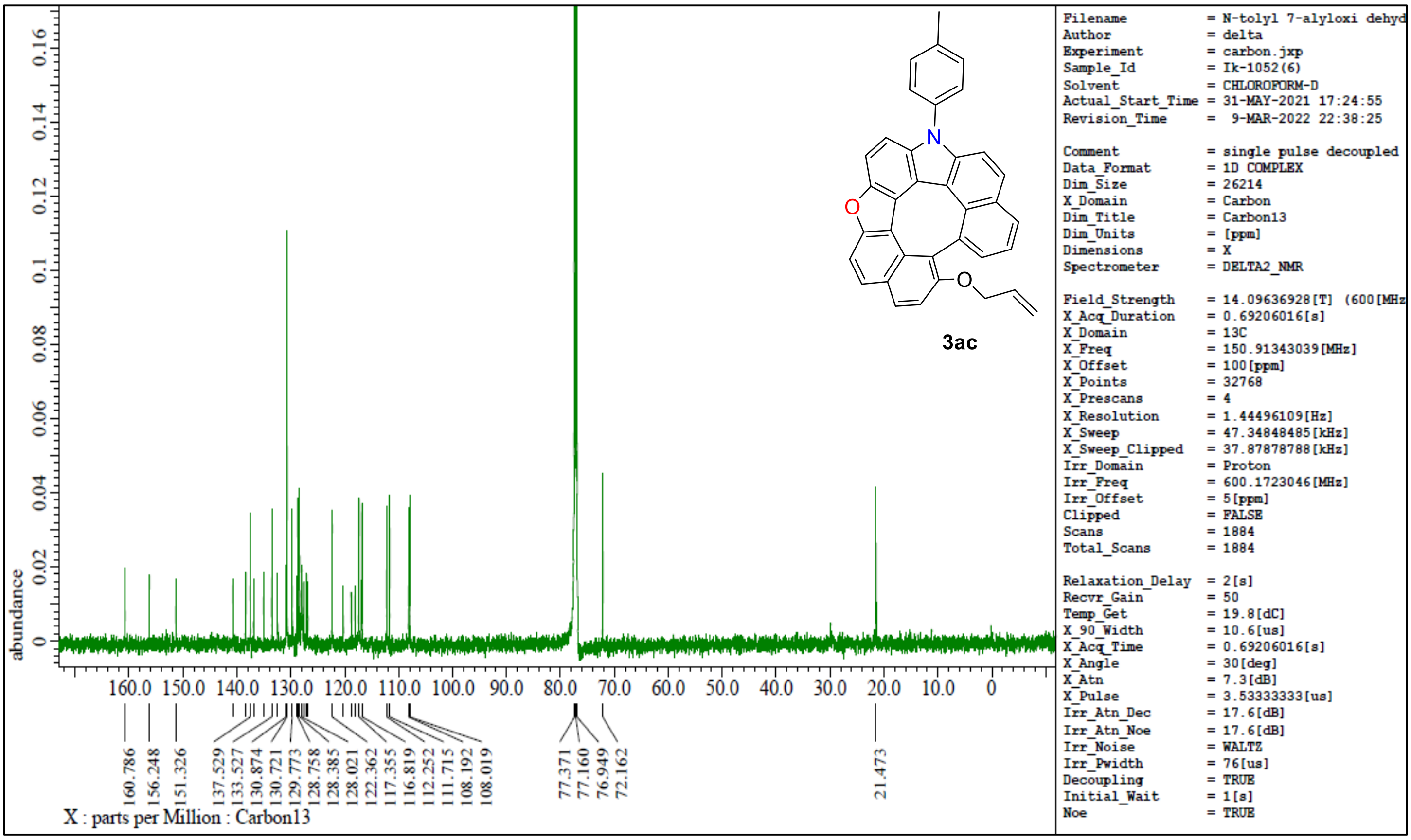

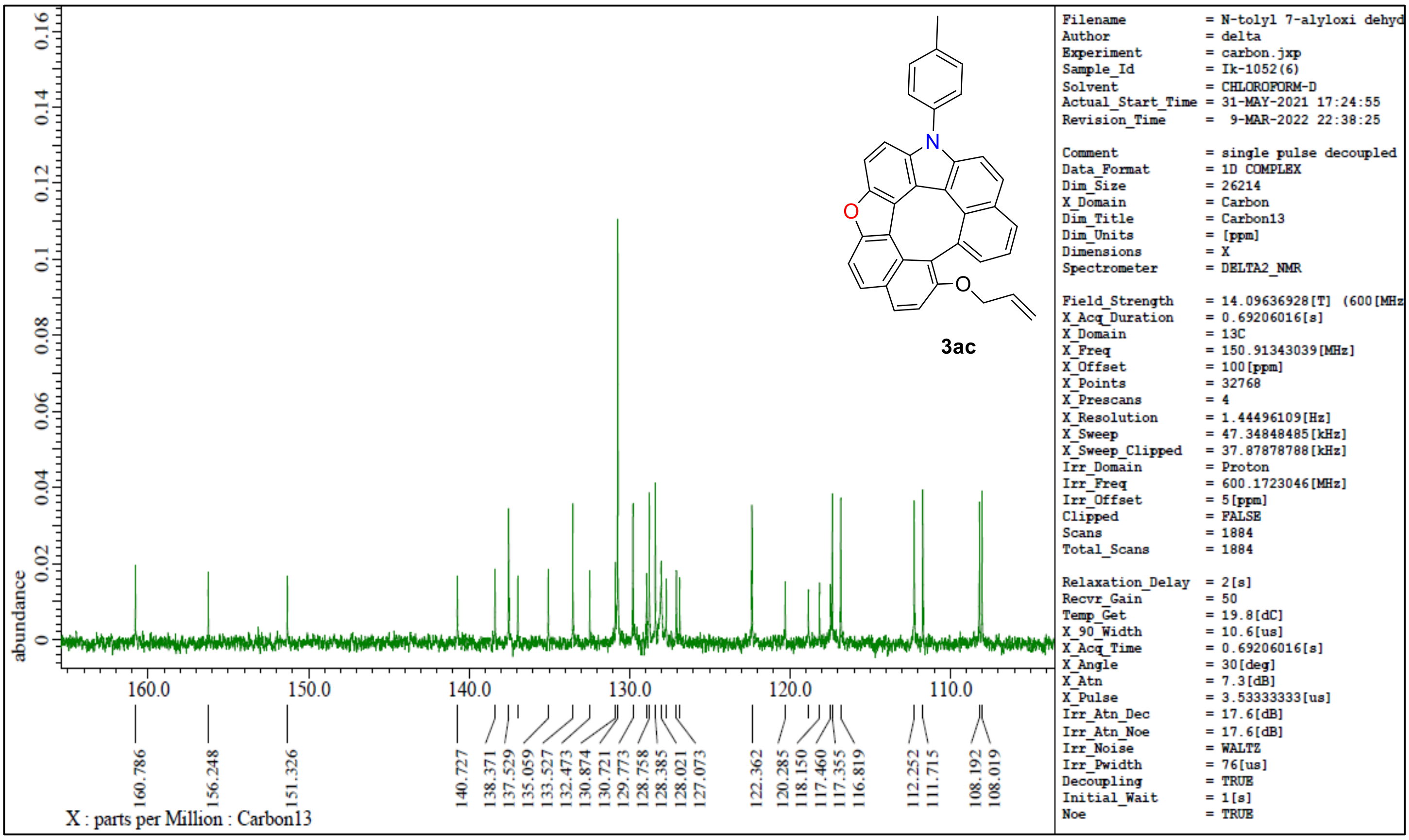

Compound **3ac** (^{13}C NMR, 150 MHz, $CDCl_3$).

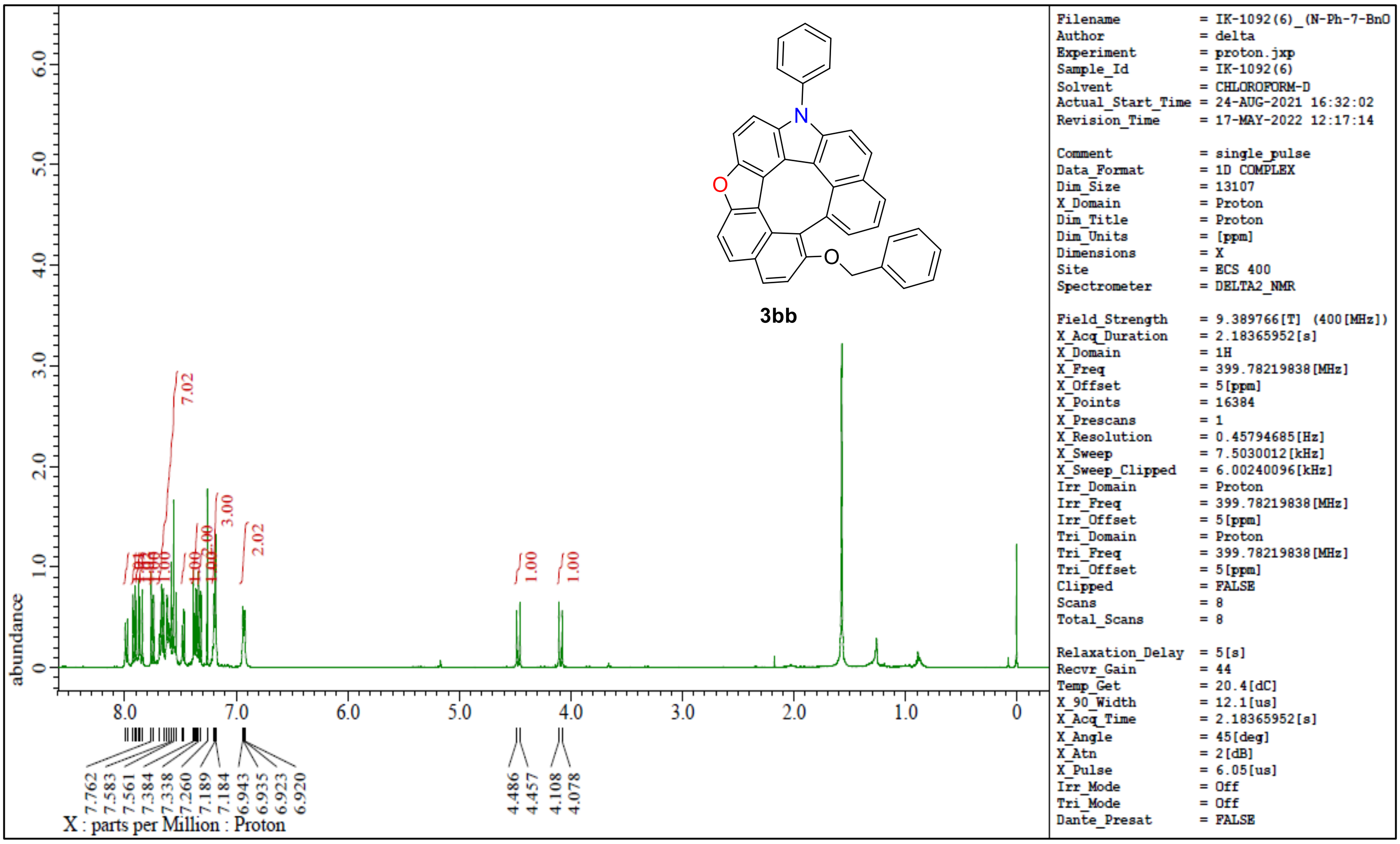

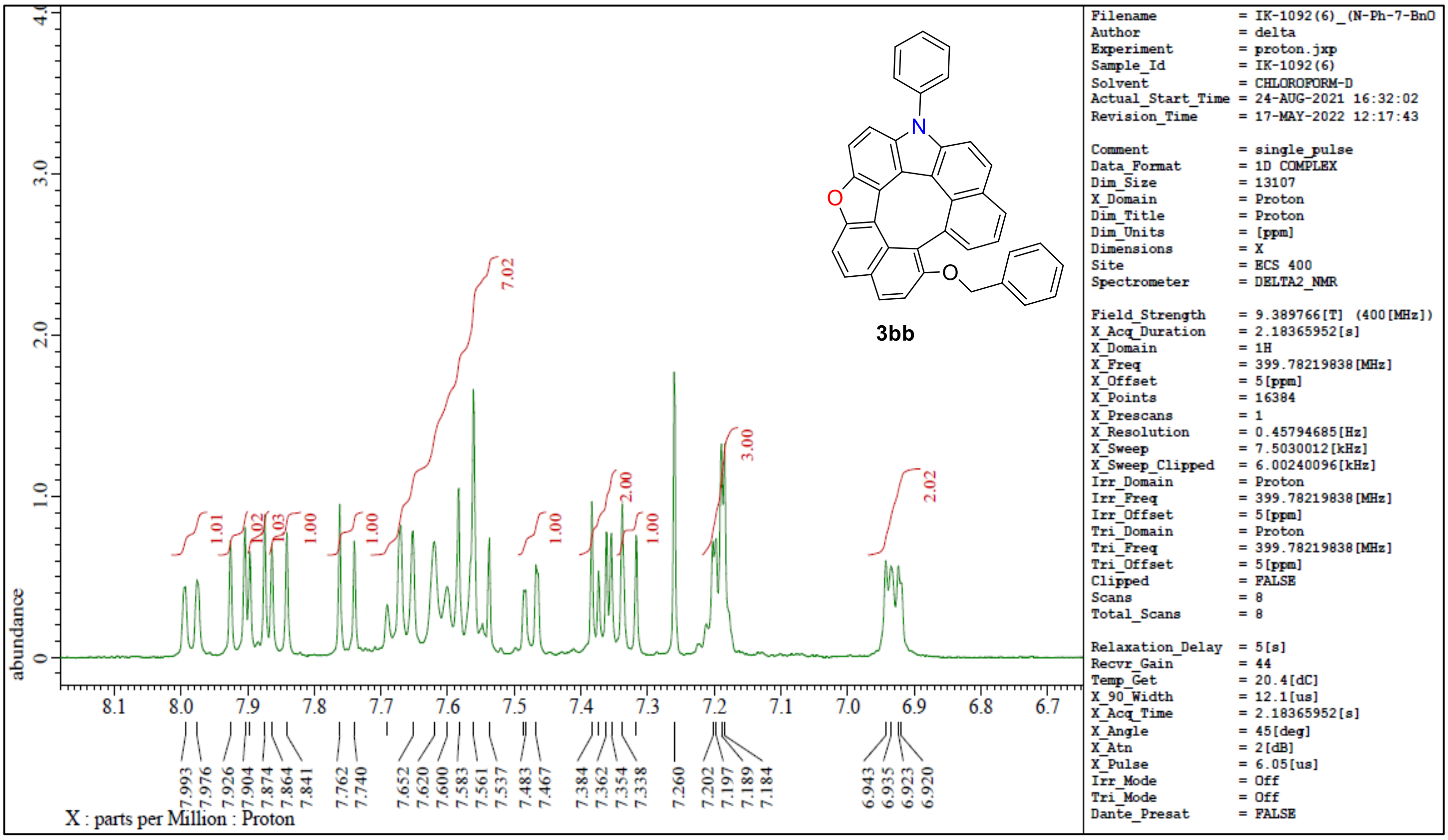

Compound **3bb** (^{1}H NMR, 400 MHz, $CDCl_3$).

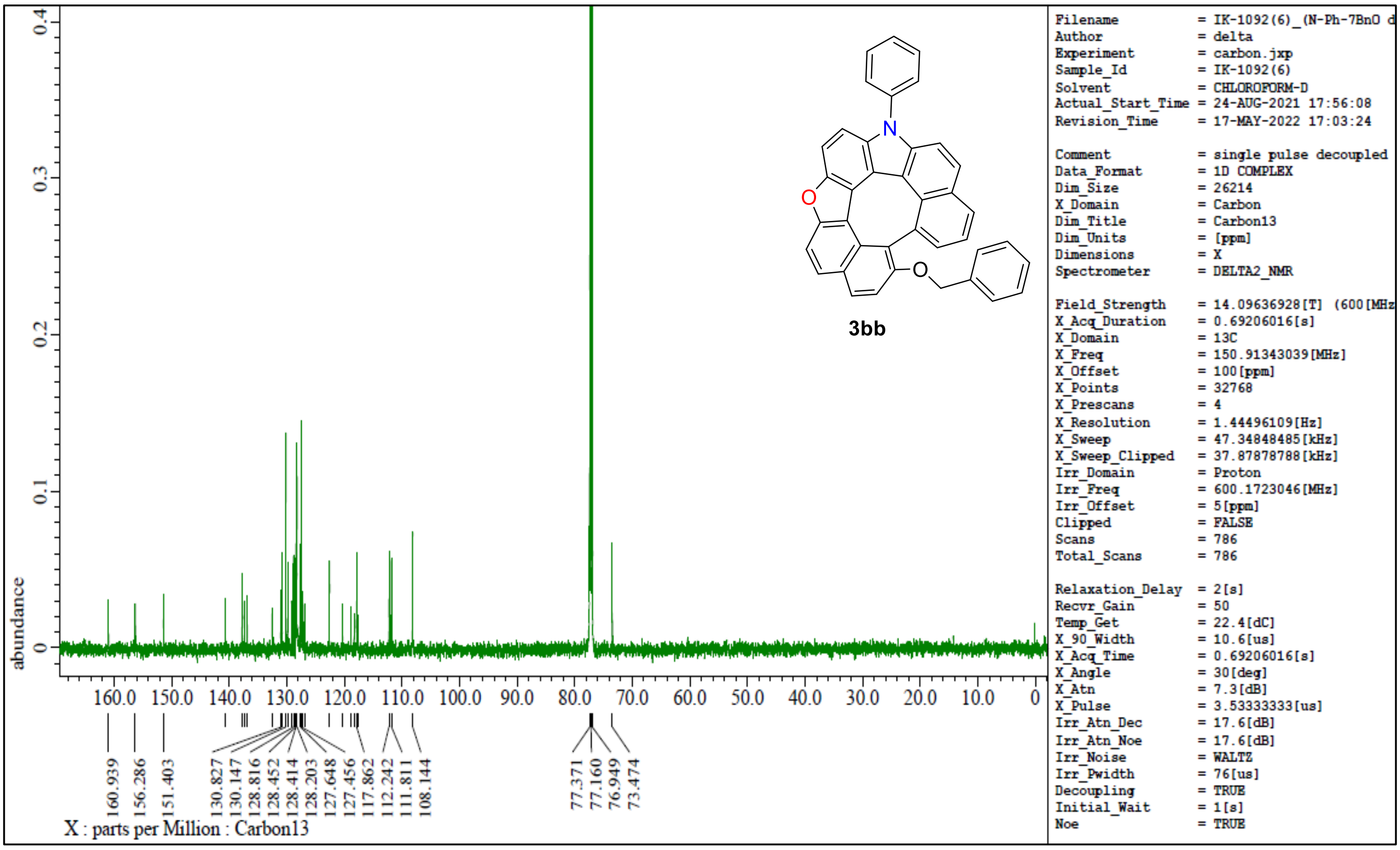

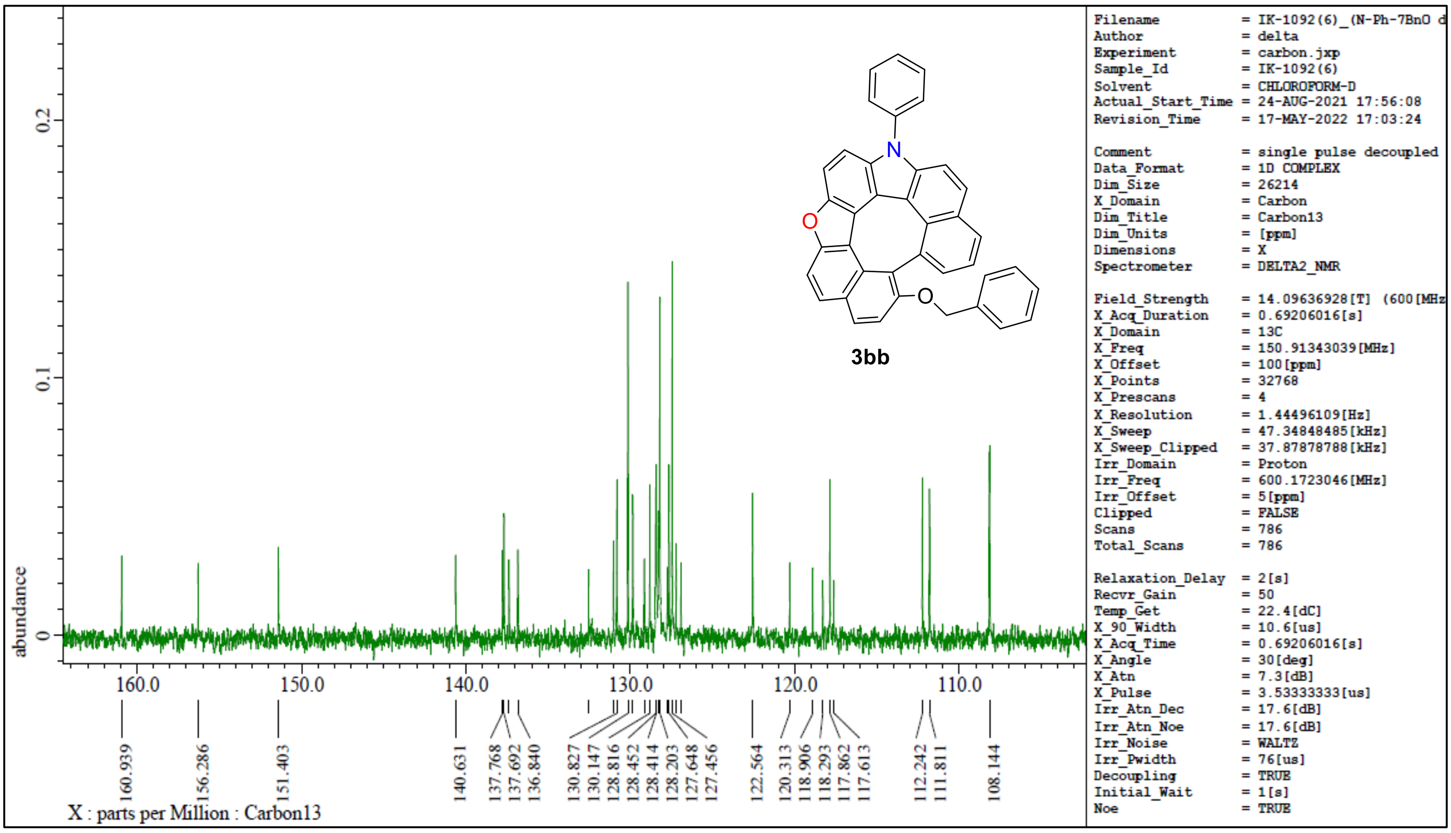

Compound **3bb** (^{13}C NMR, 150 MHz, $CDCl_3$).

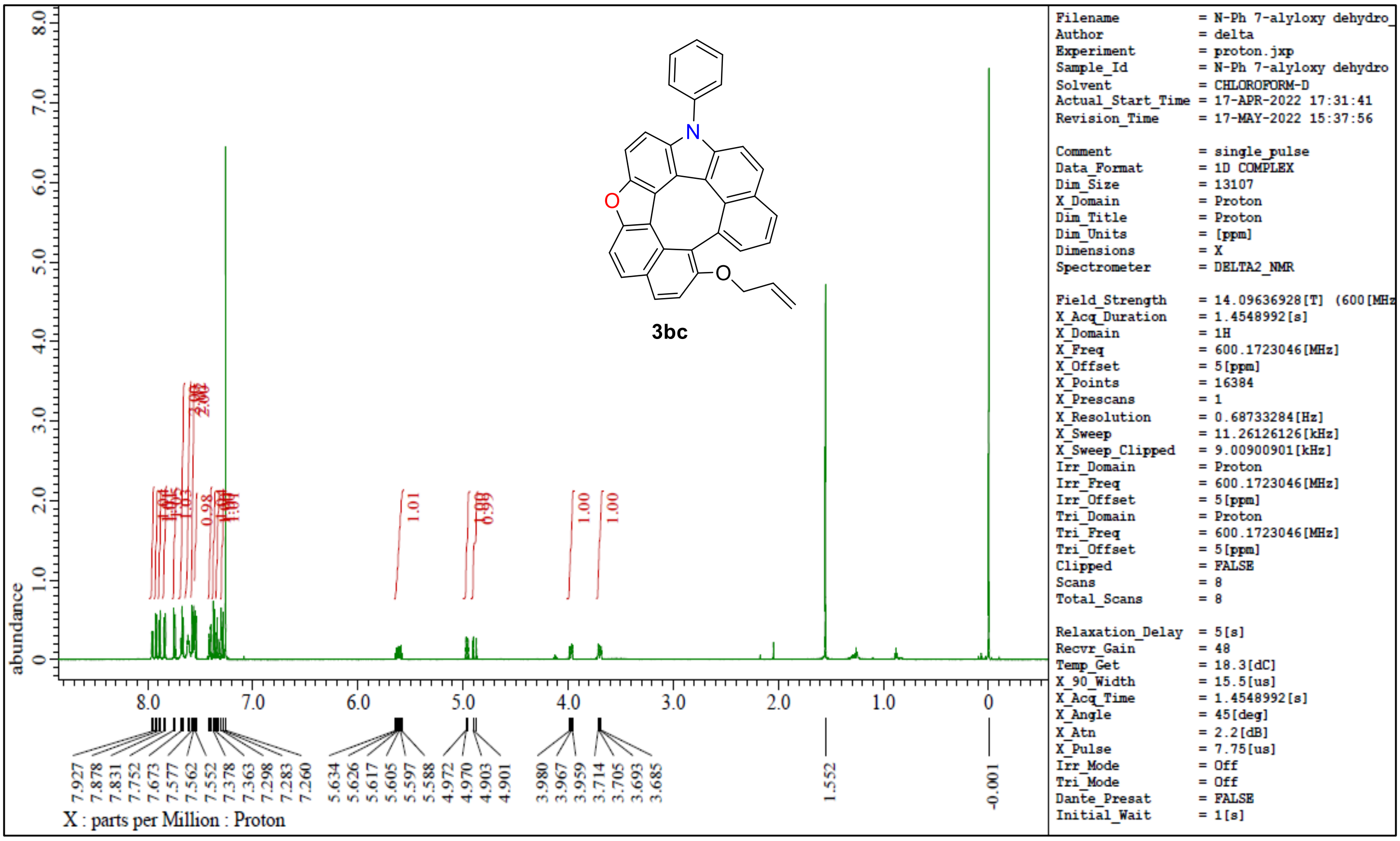

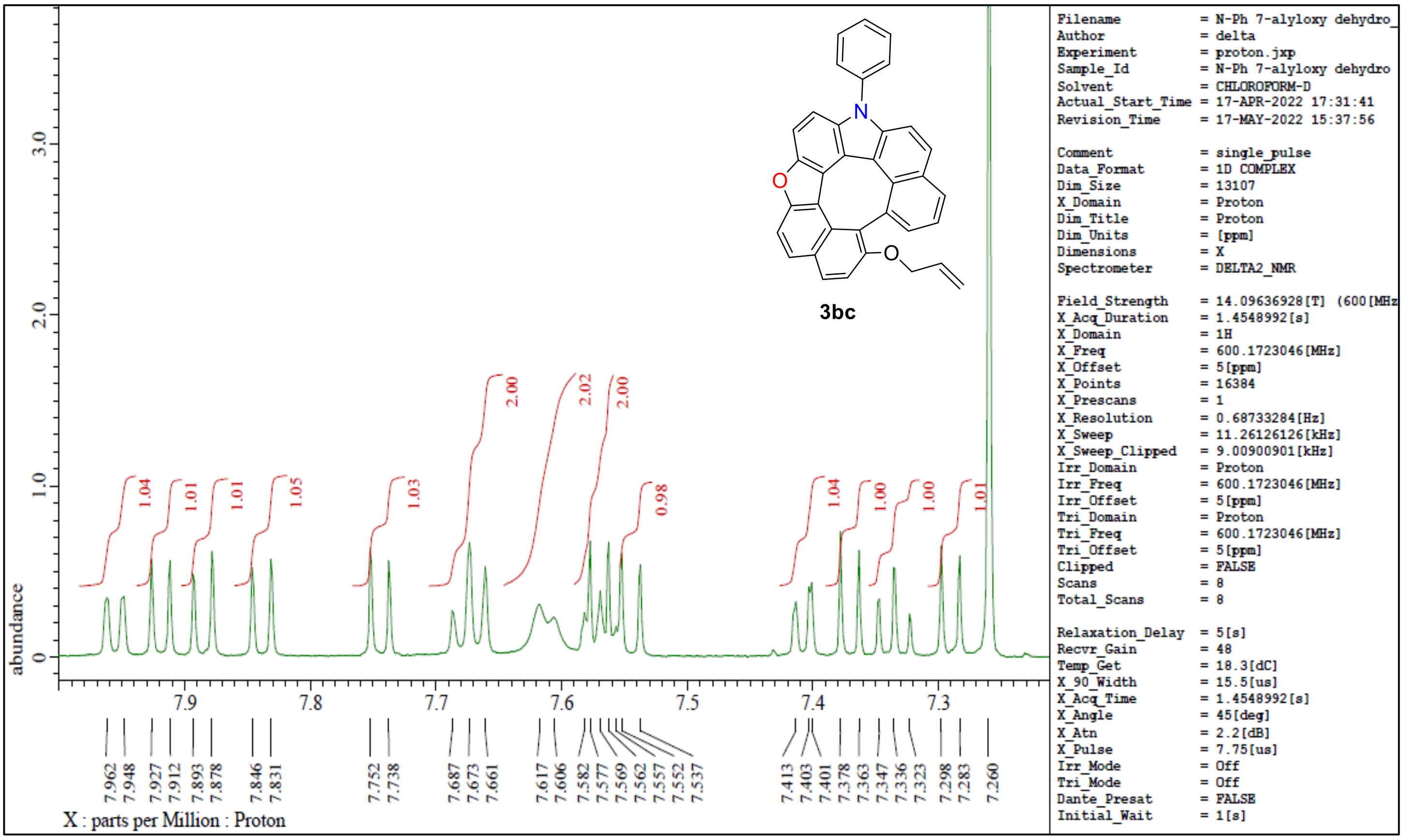

Compound **3bc** (^{1}H NMR, 600 MHz, $CDCl_3$).

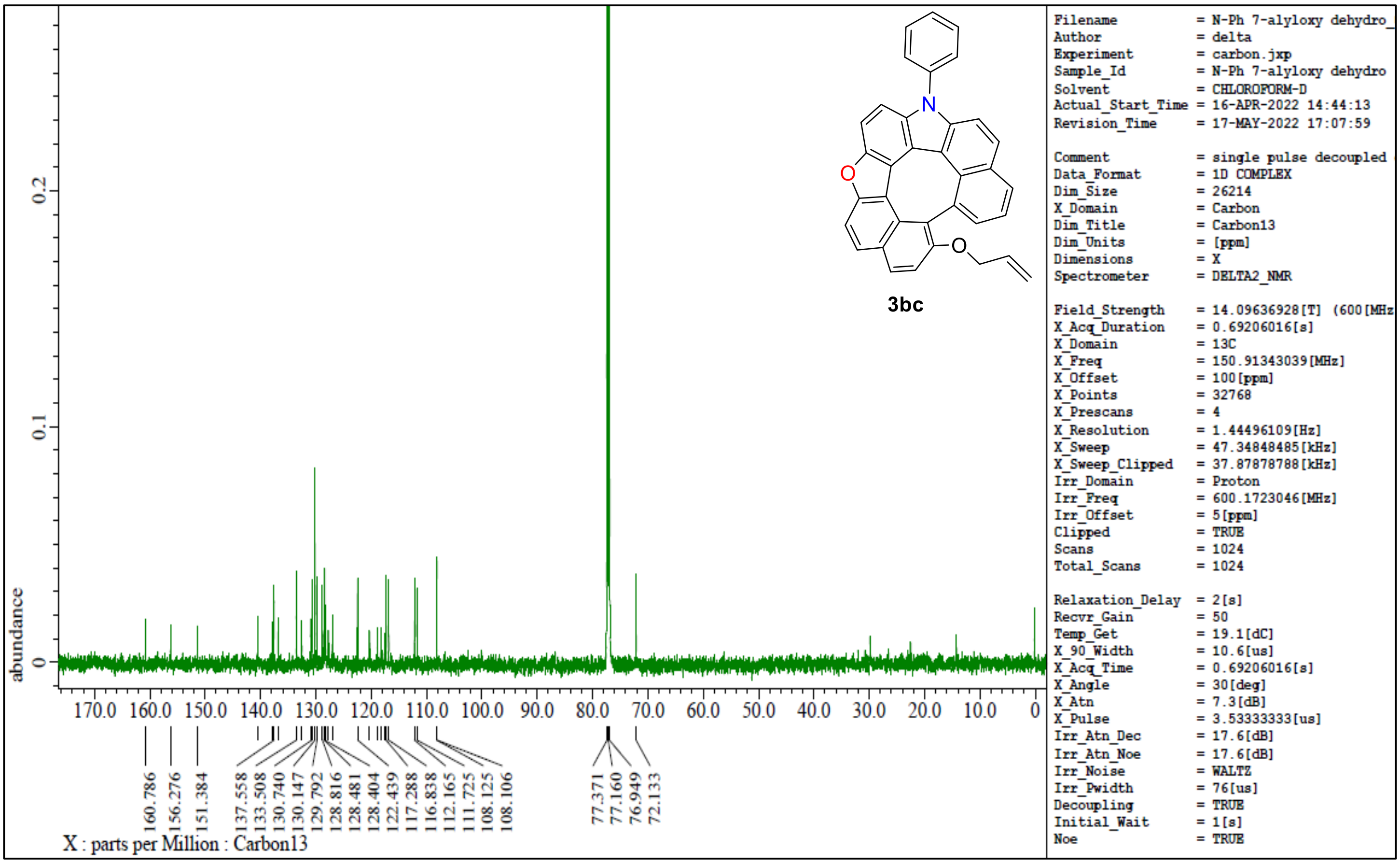

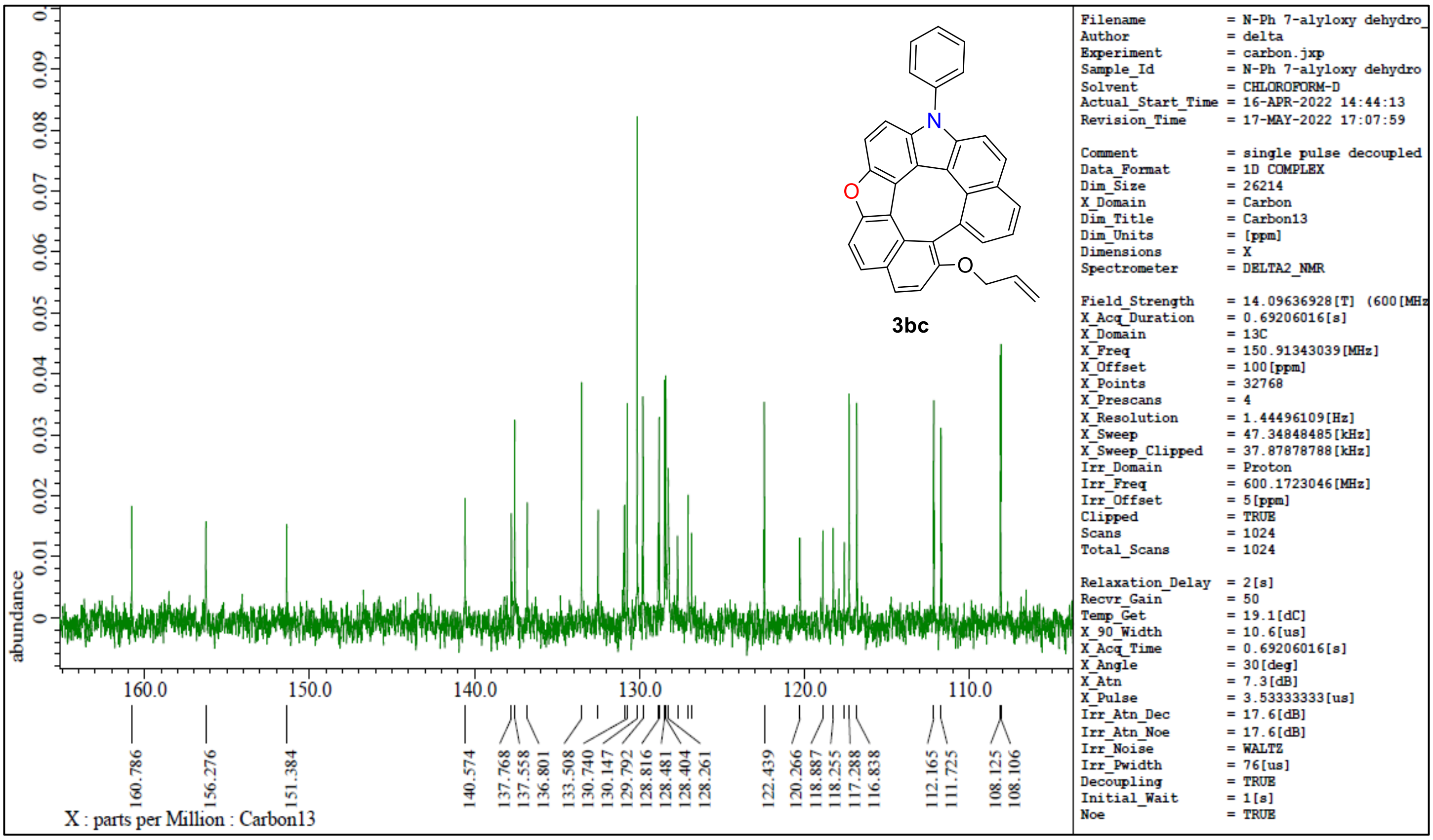

Compound **3bc** (^{13}C NMR, 150 MHz, $CDCl_3$).

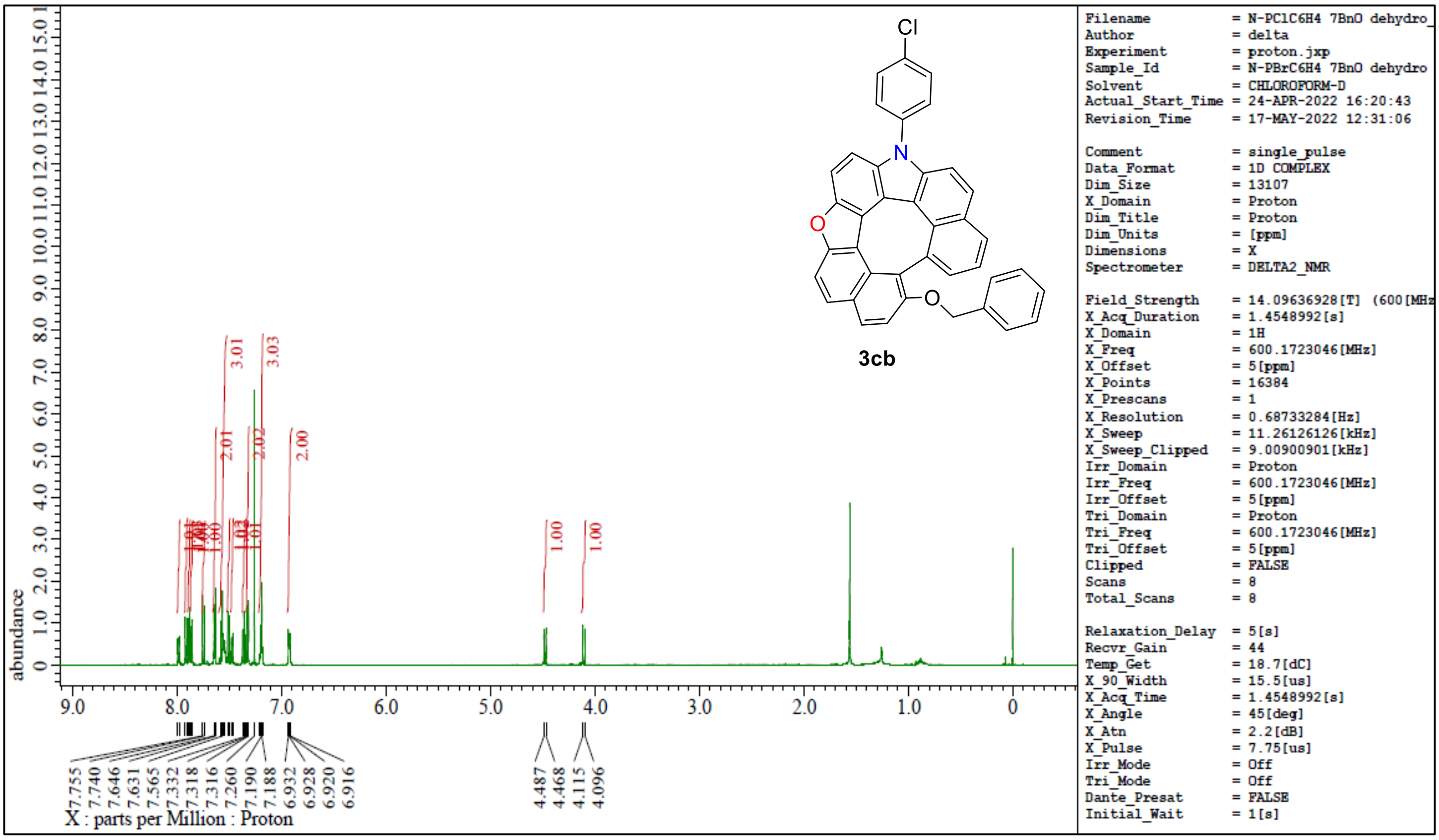

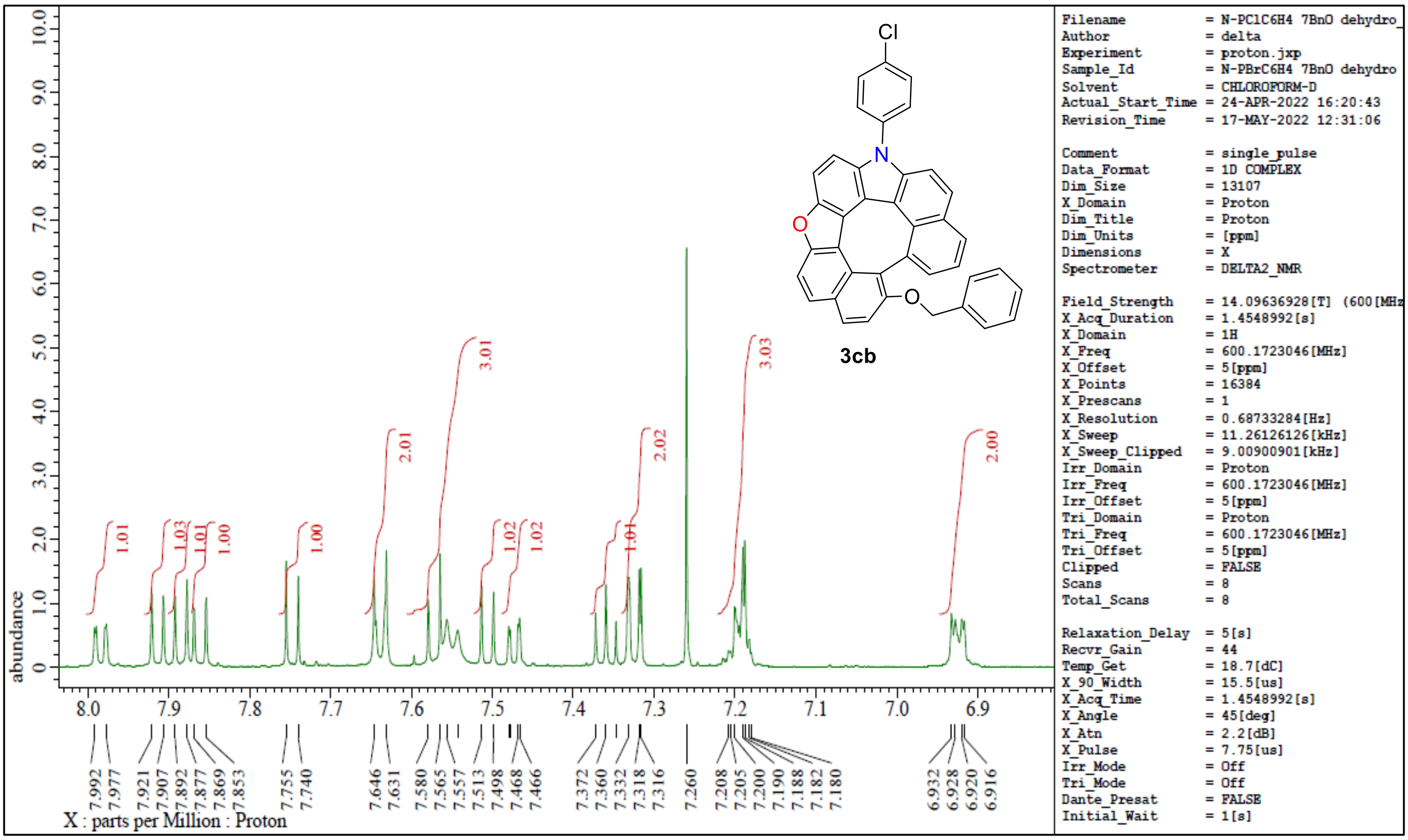

Compound **3cb** (^{1}H NMR, 600 MHz, $CDCl_3$).

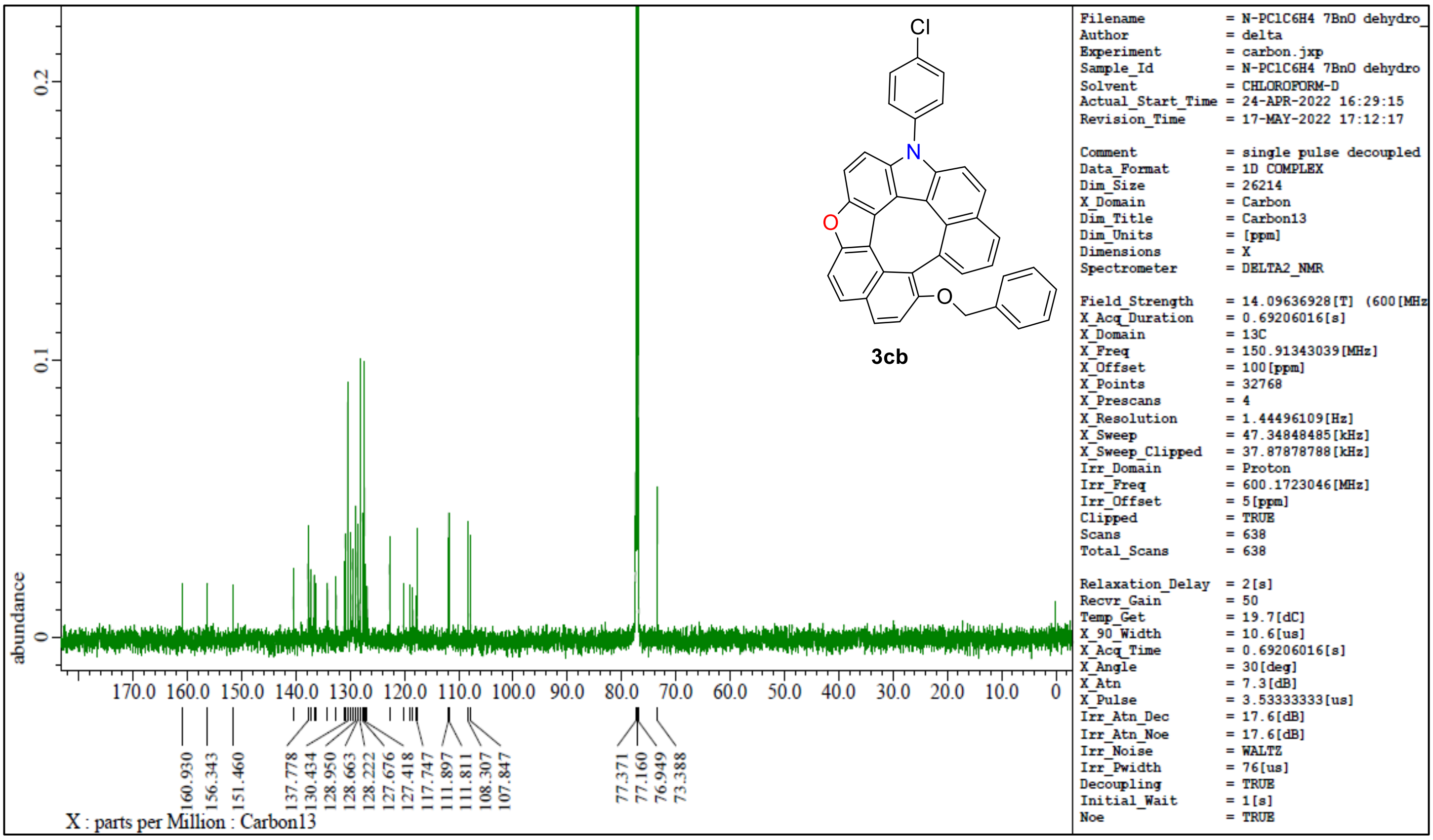

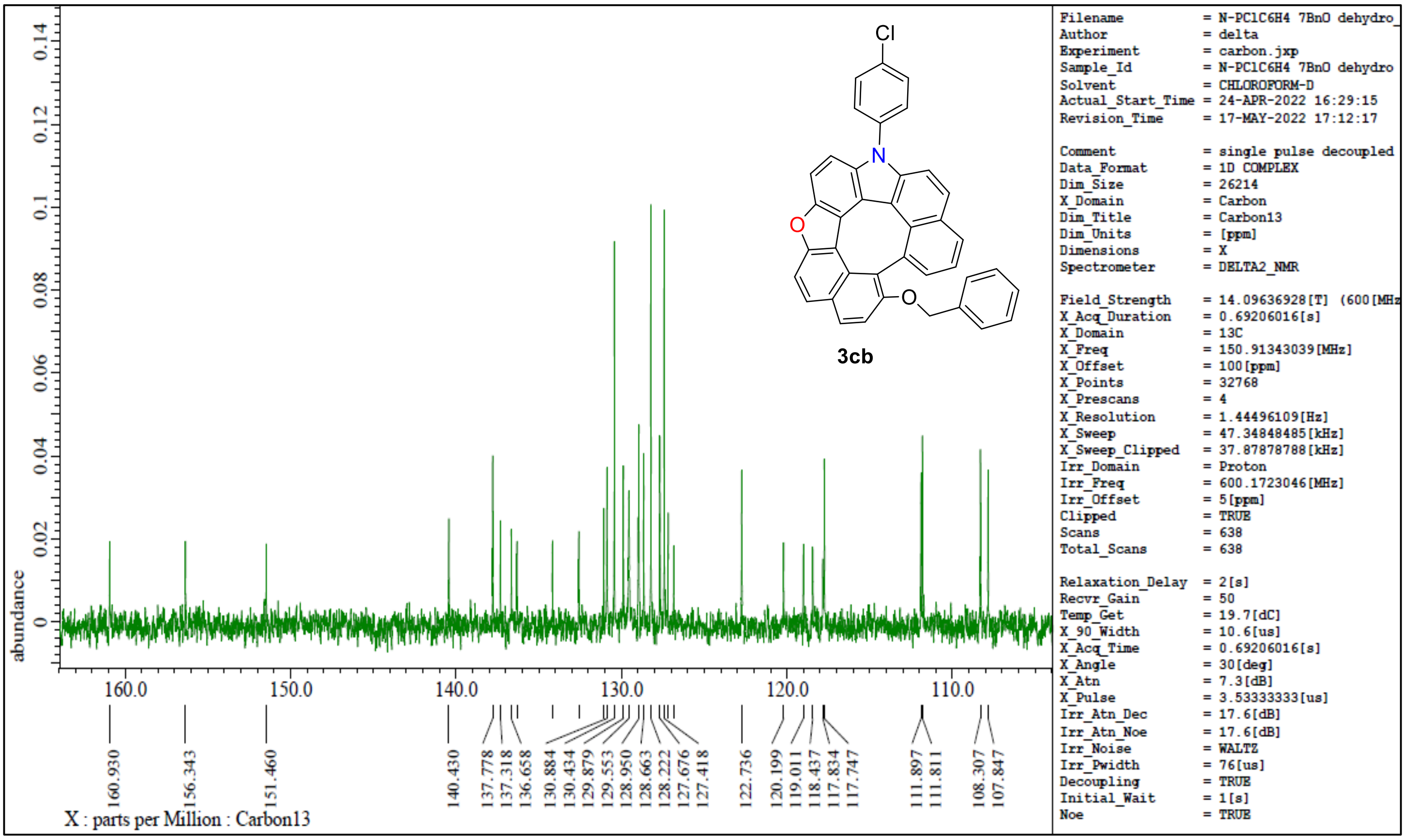

Compound **3cb** (^{13}C NMR, 150 MHz, $CDCl_3$).

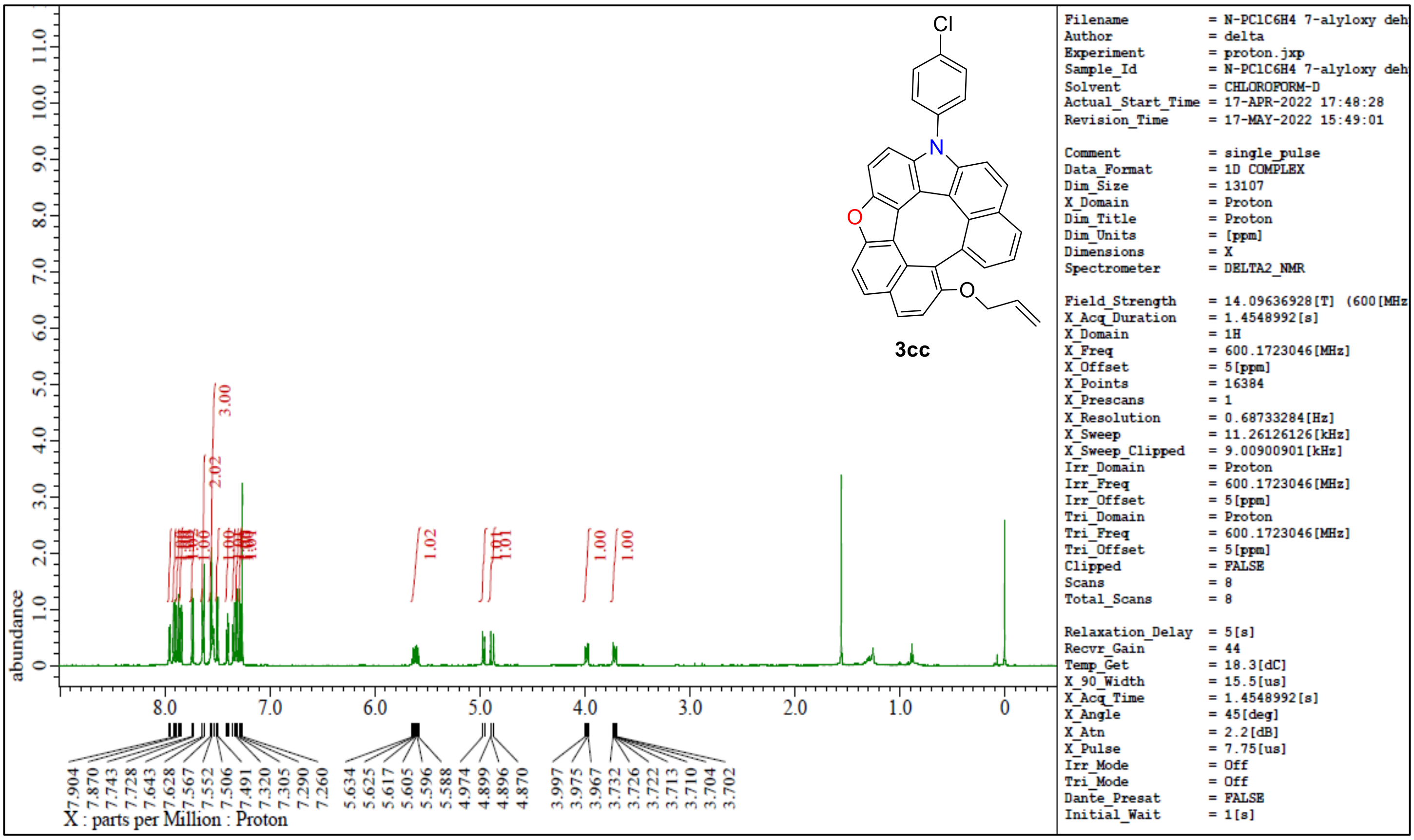

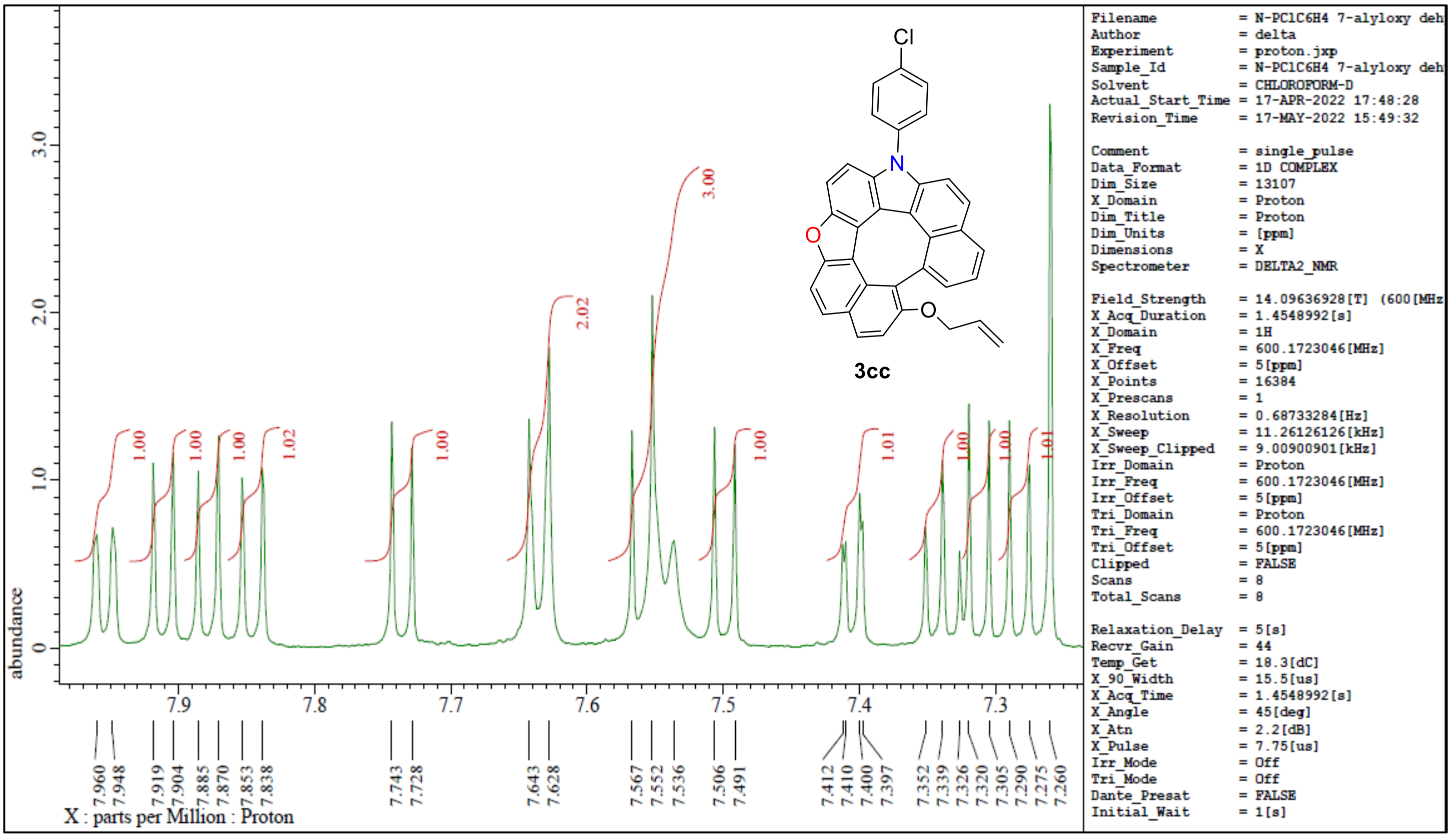

Compound **3cc** (^{1}H NMR, 600 MHz, $CDCl_3$).

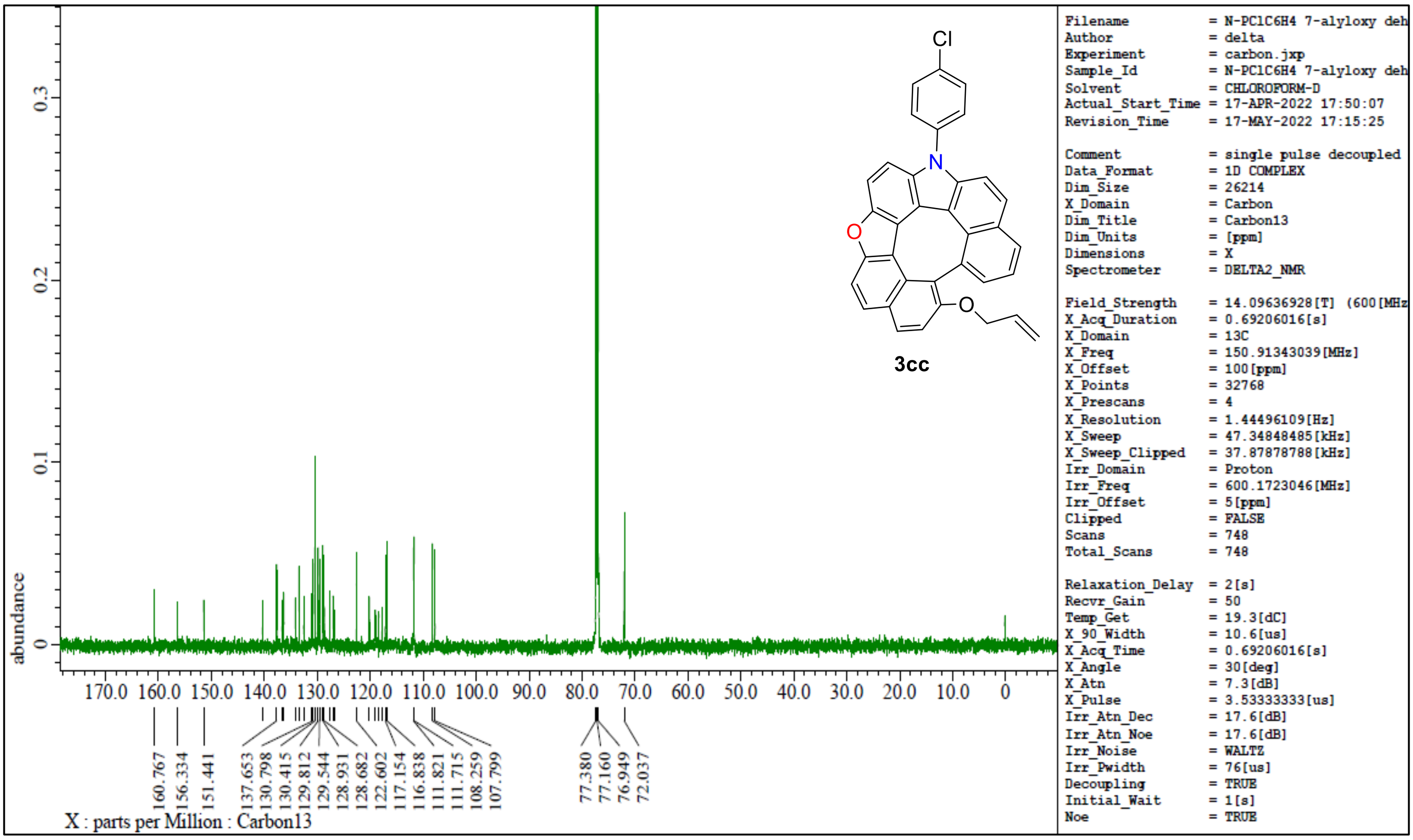

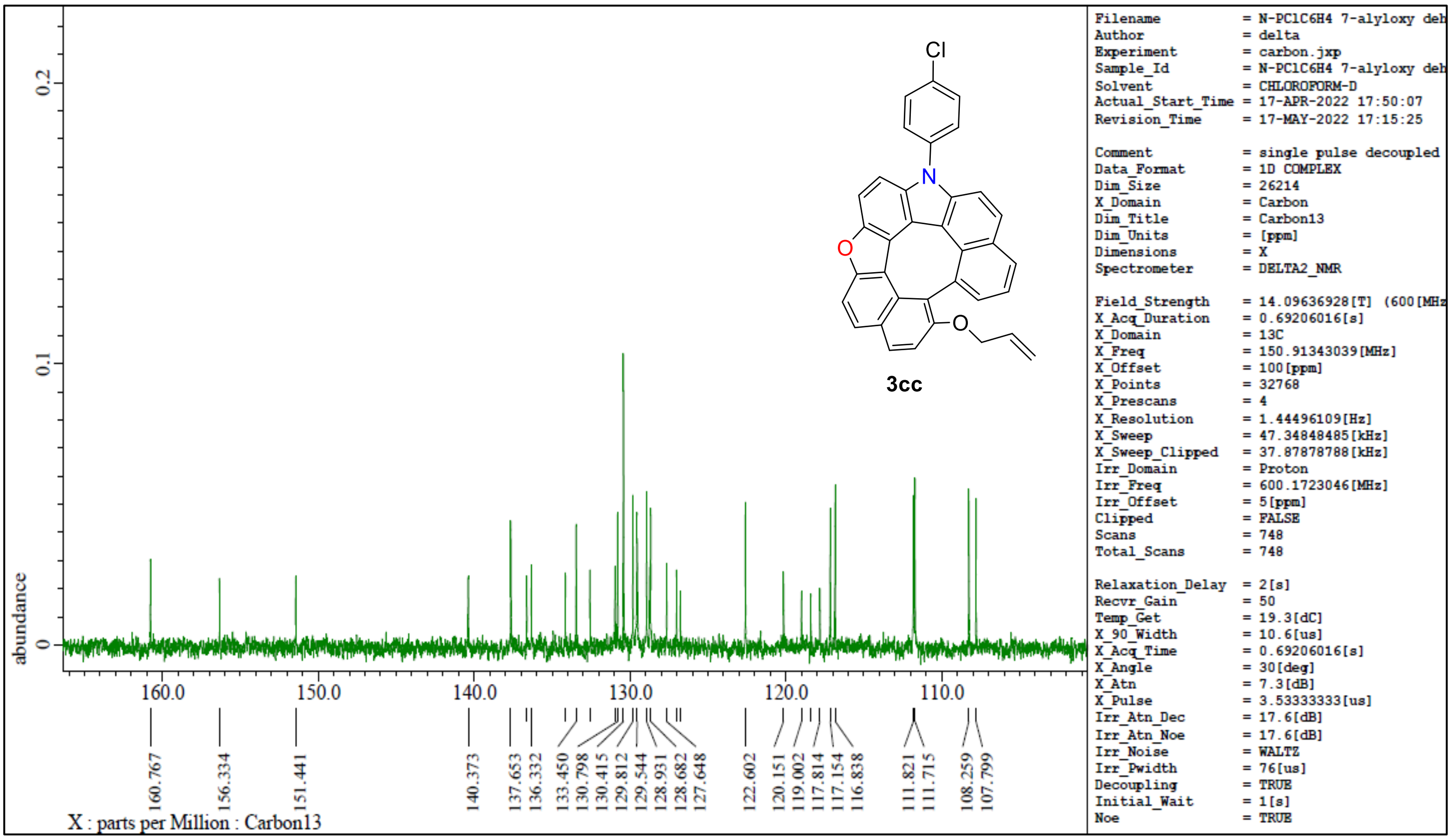

Compound **3cc** (^{13}C NMR, 150 MHz, $CDCl_3$).

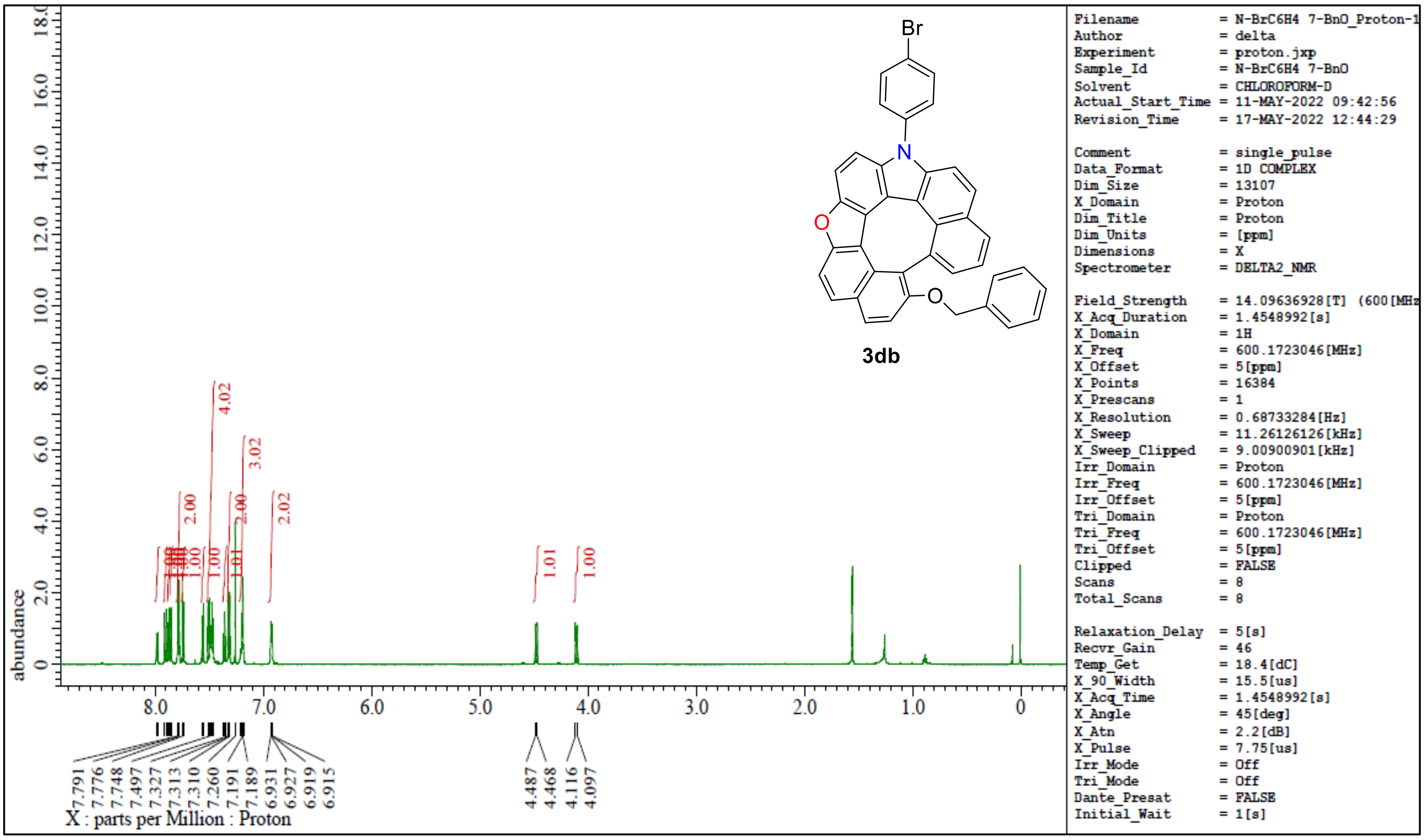

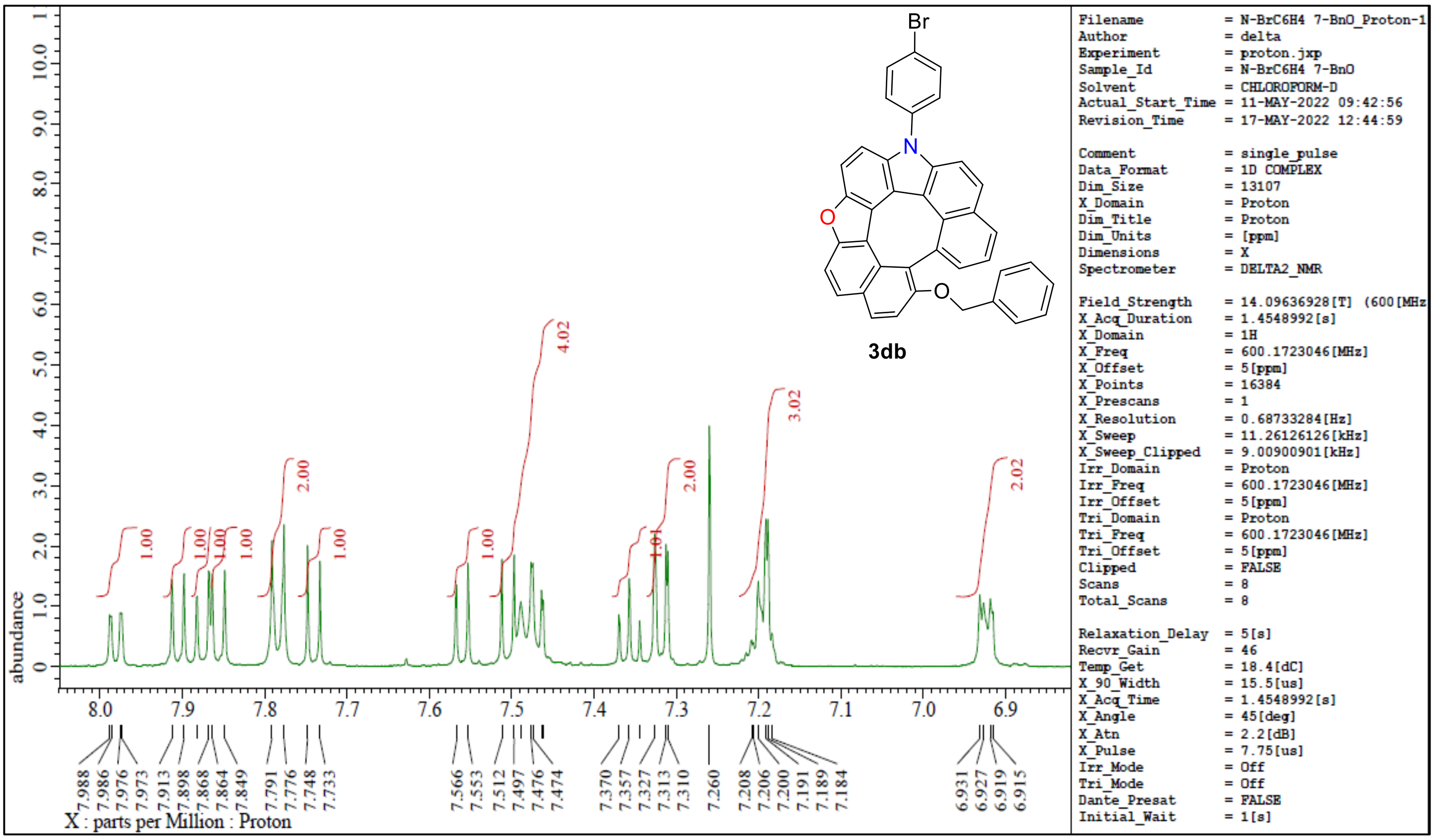

Compound **3db** (^{1}H NMR, 600 MHz, $CDCl_3$).

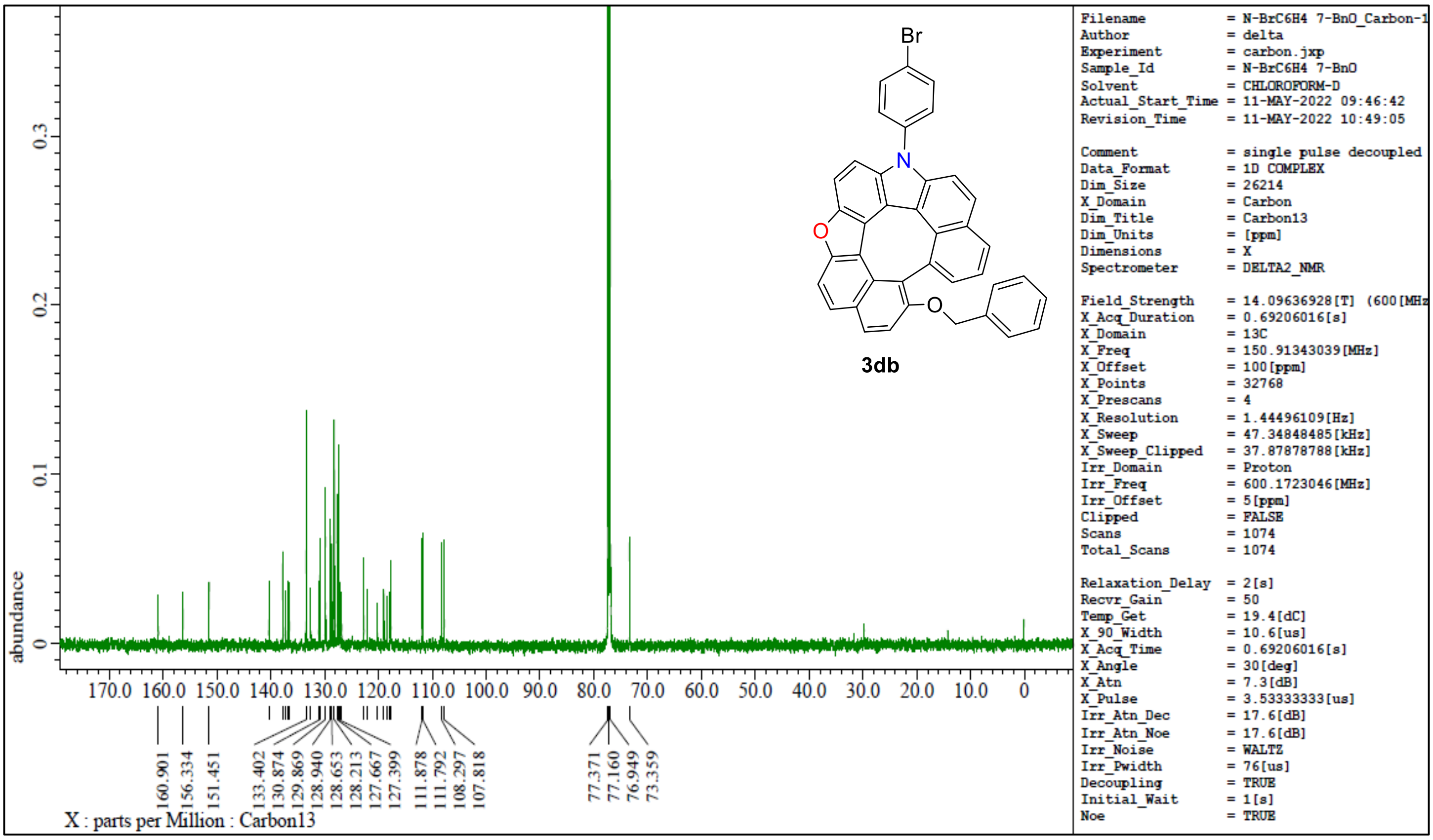

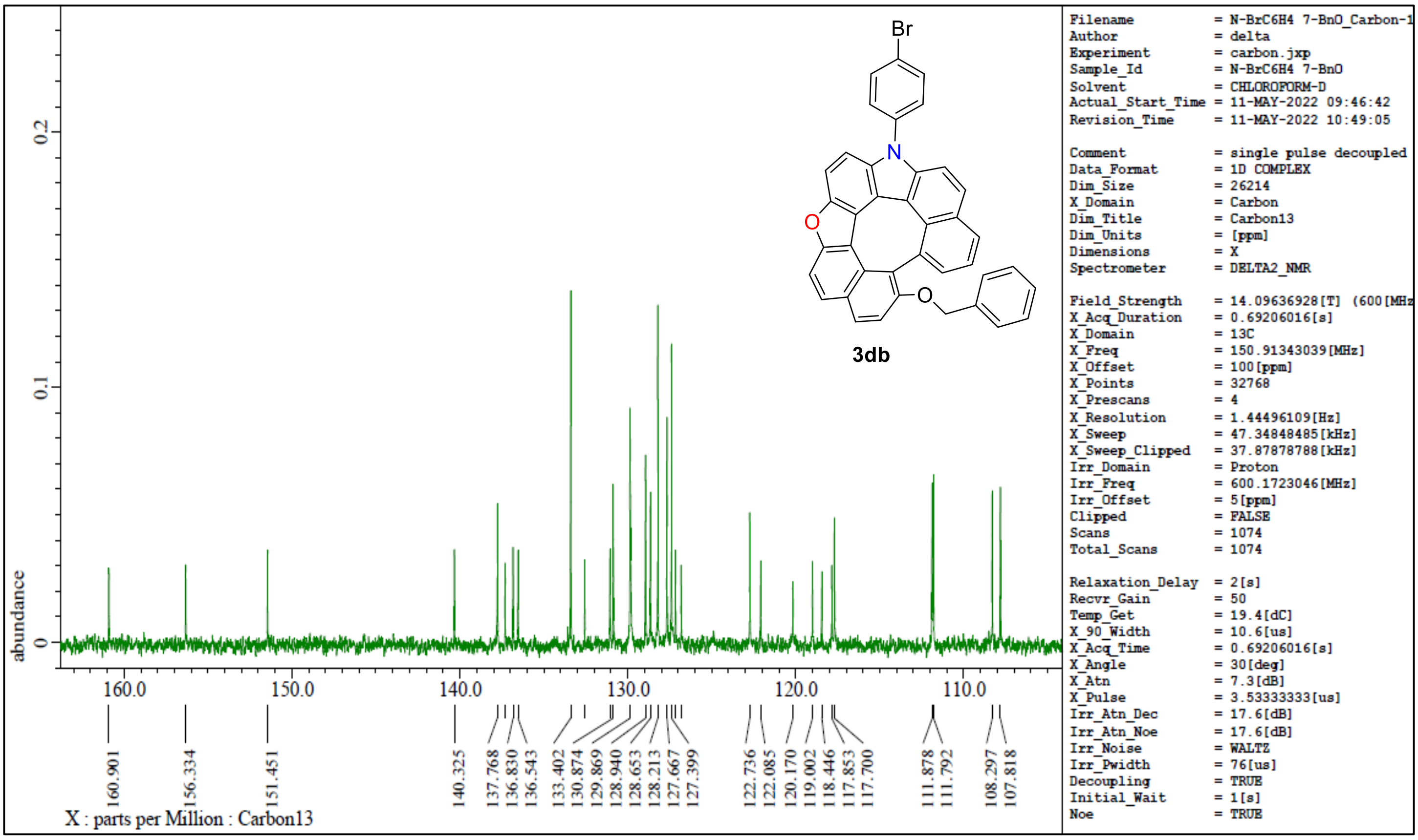

Compound **3db** (^{13}C NMR, 150 MHz, $CDCl_3$).

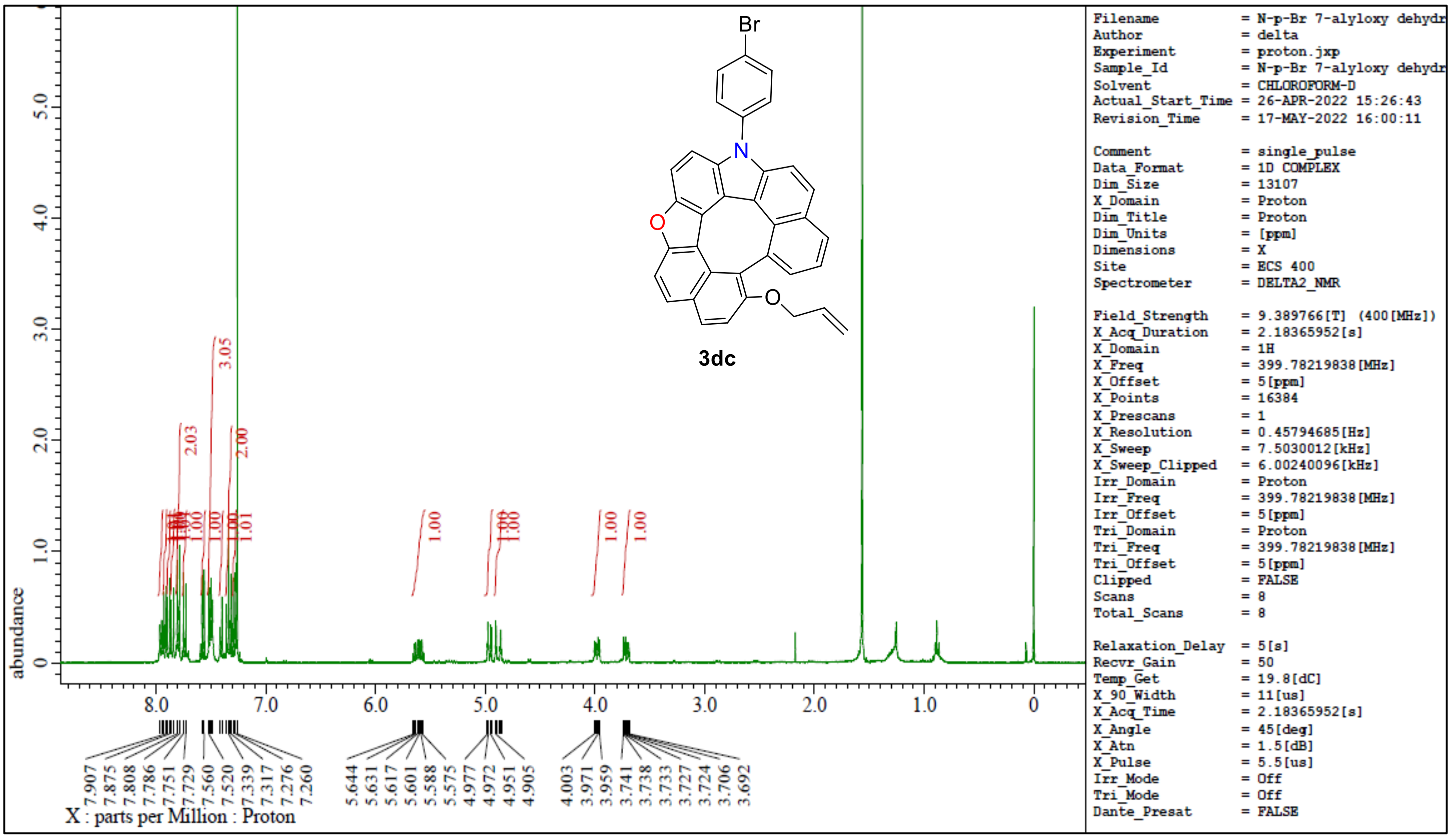

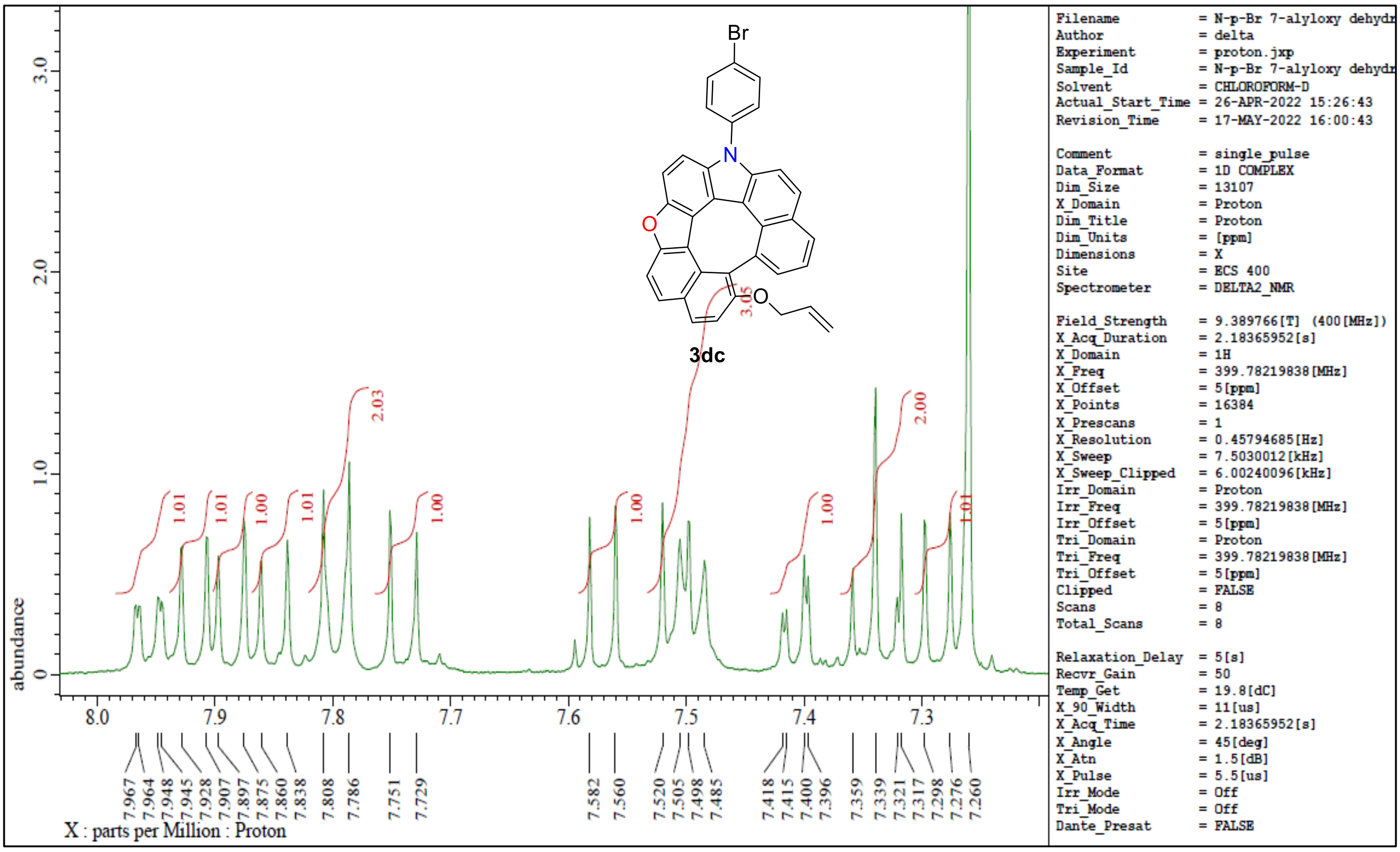

Compound **3dc** (^{1}H NMR, 400 MHz, $CDCl_3$).

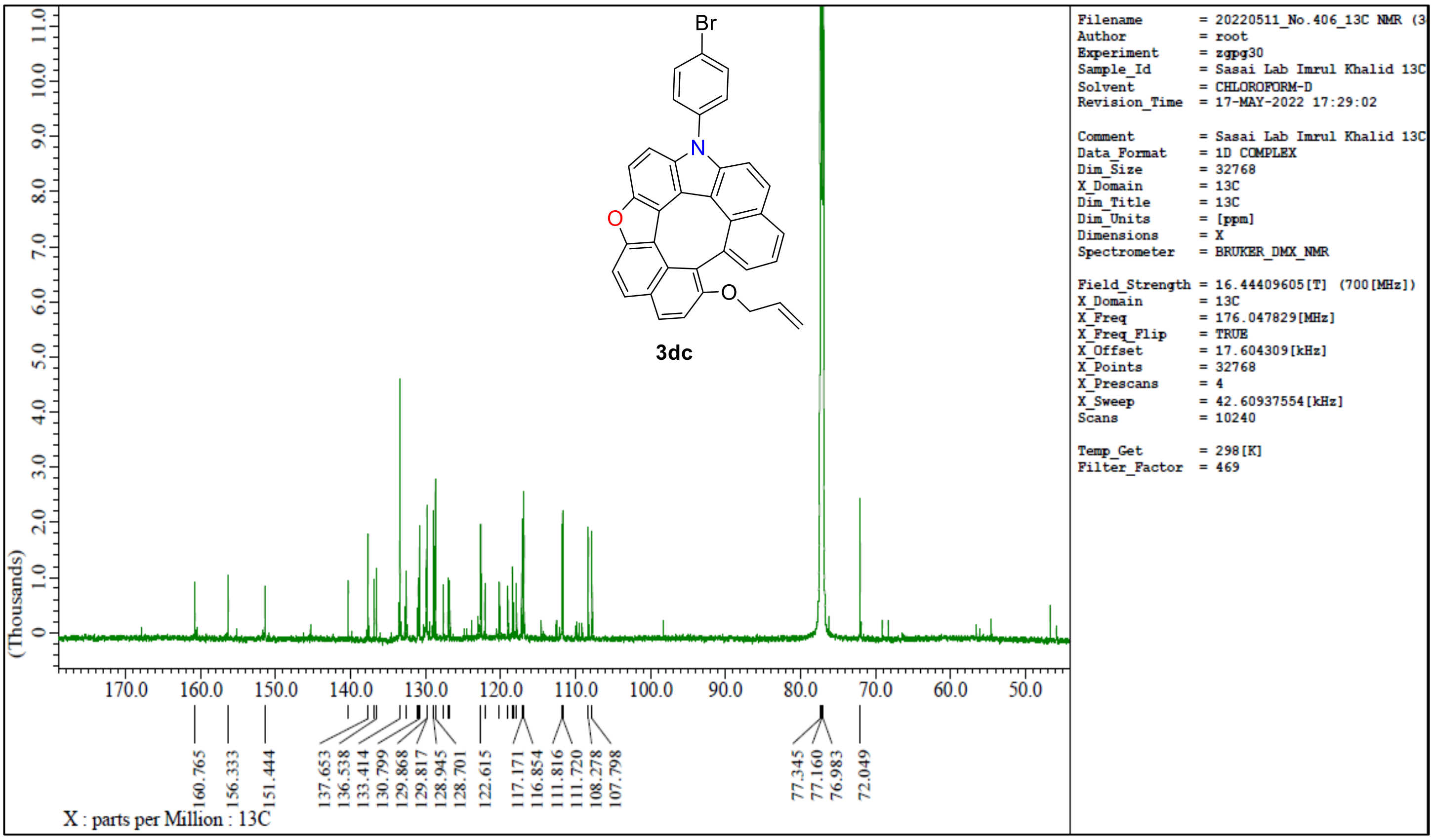

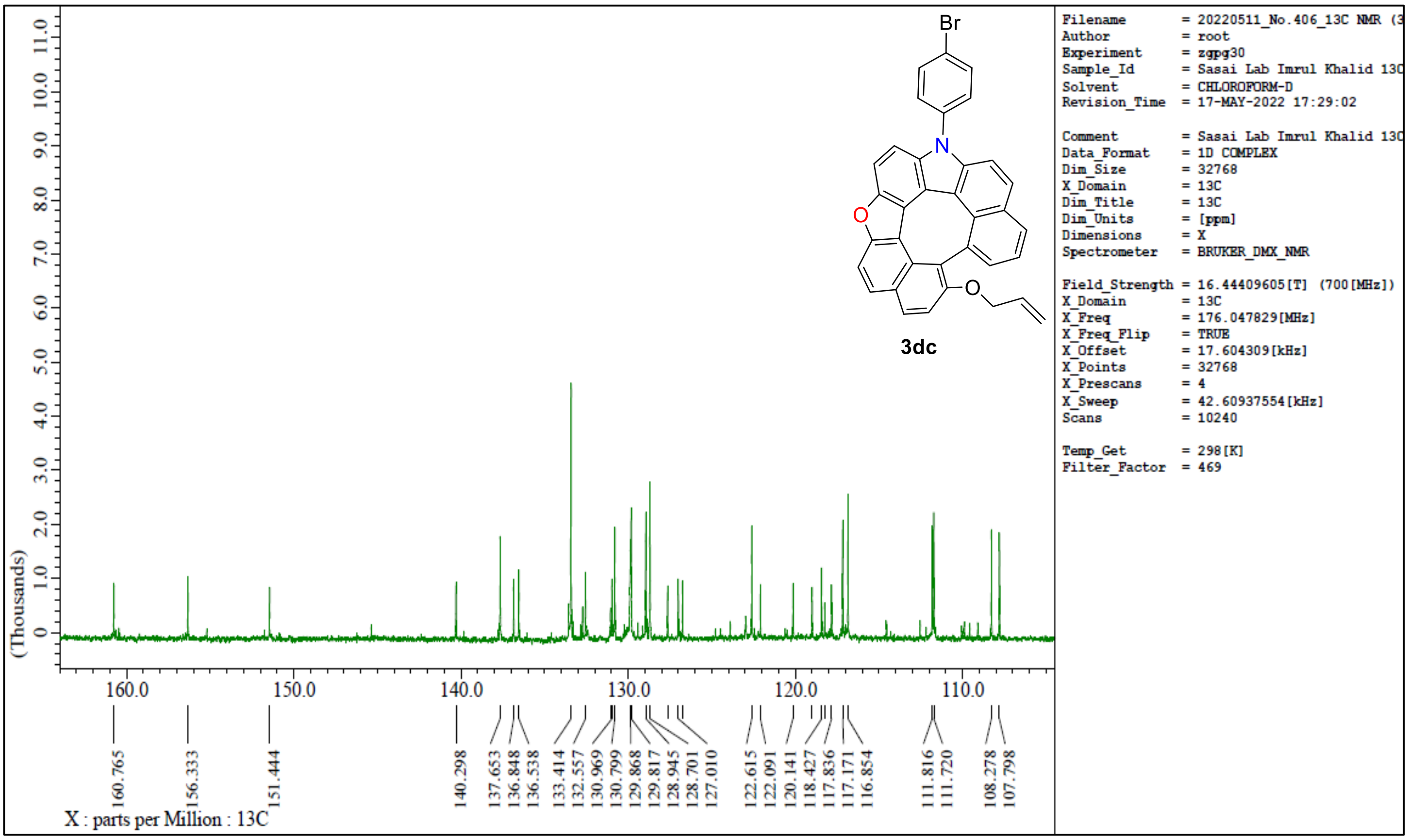

Compound **3dc** (^{13}C NMR, 175 MHz, $CDCl_3$).

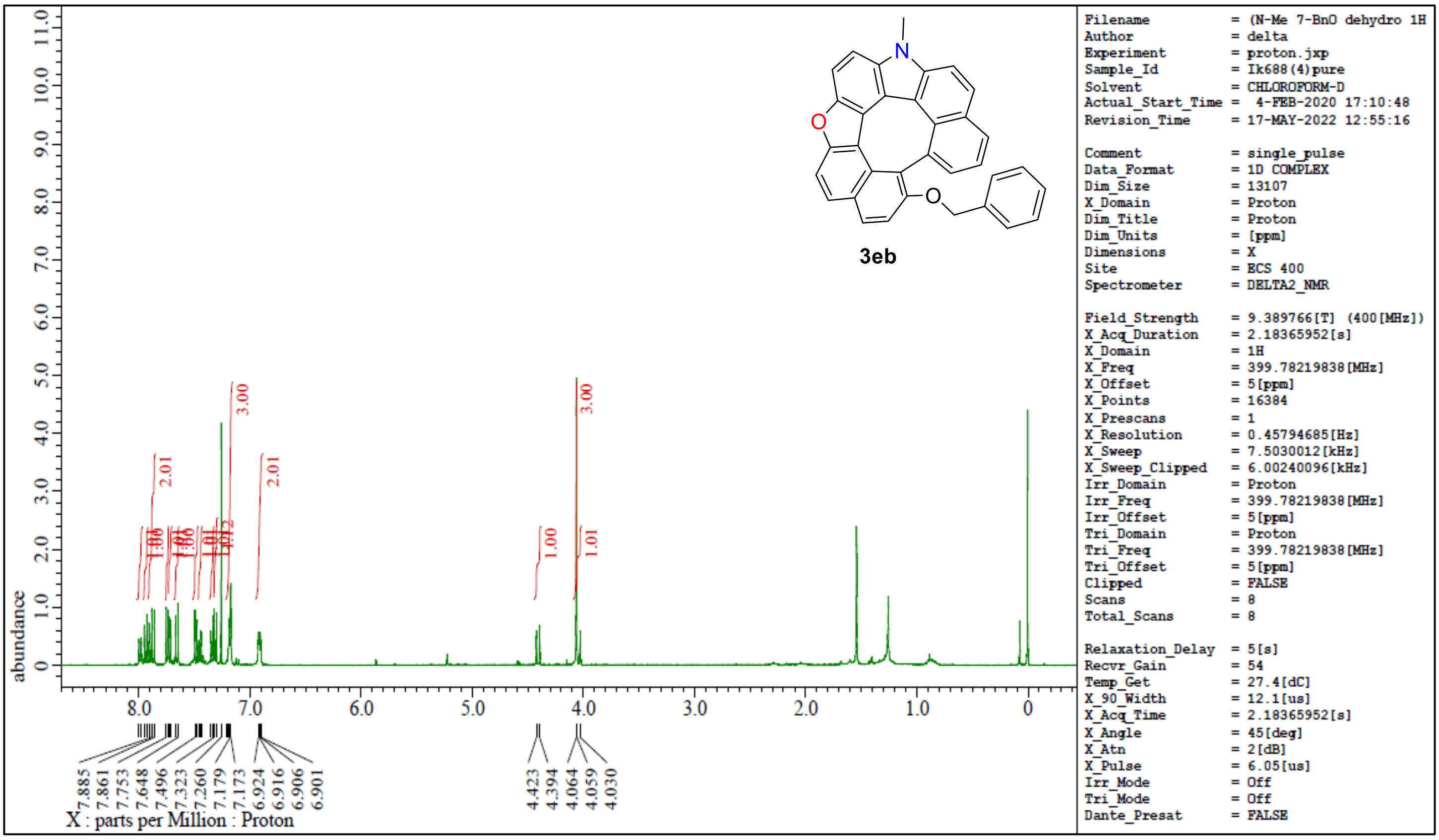

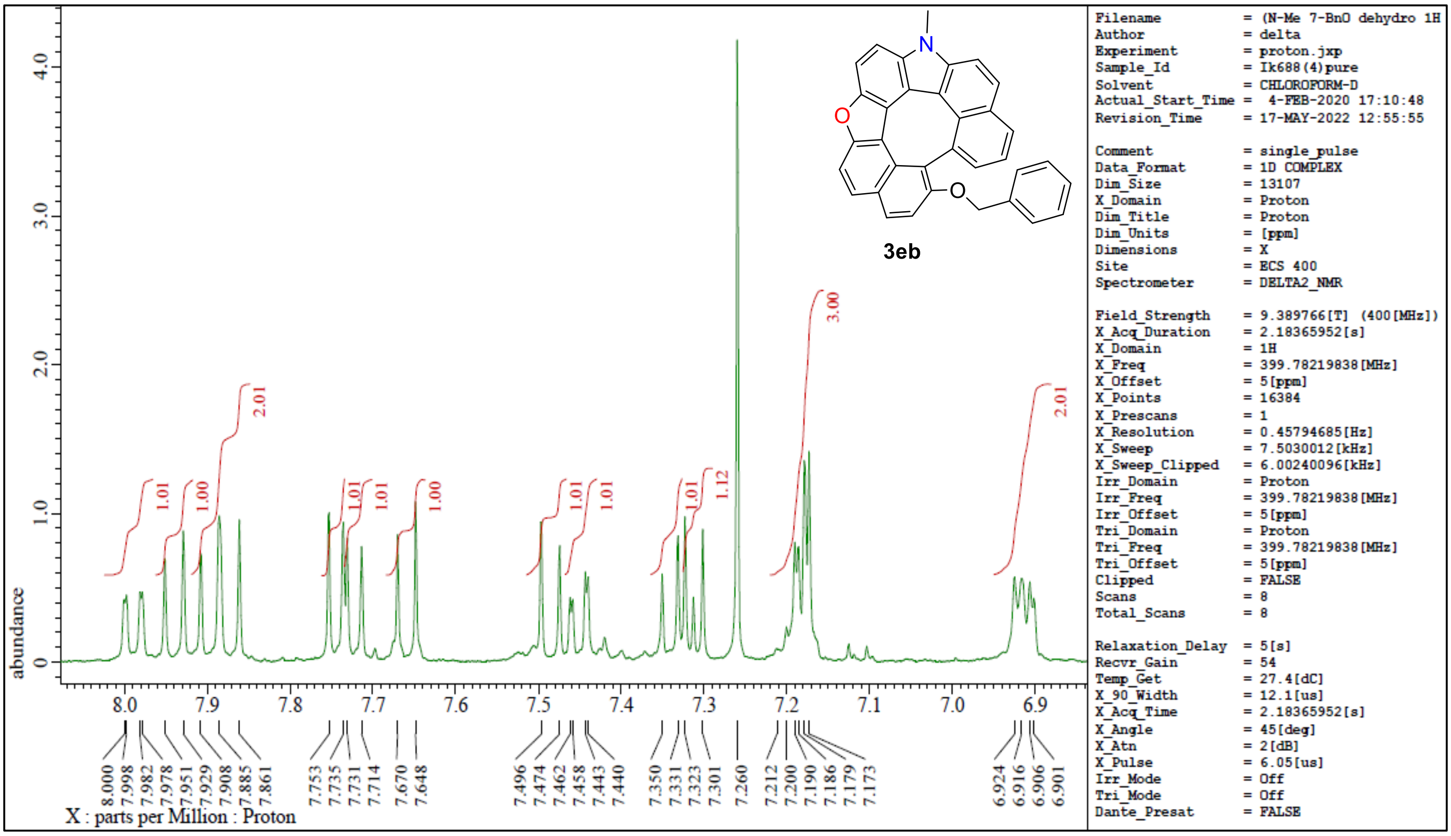

Compound **3eb** (^{1}H NMR, 400 MHz, $CDCl_3$).

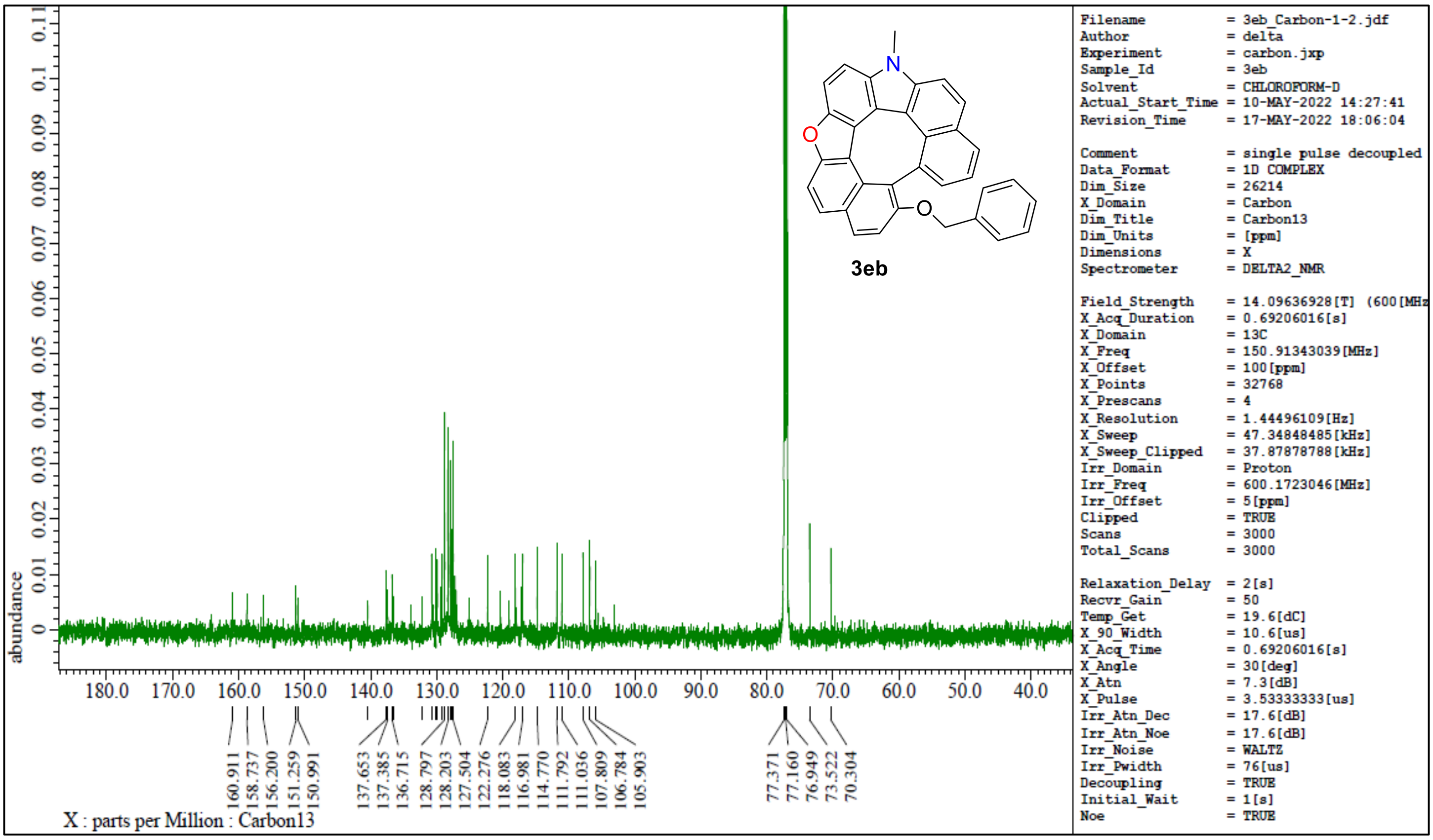

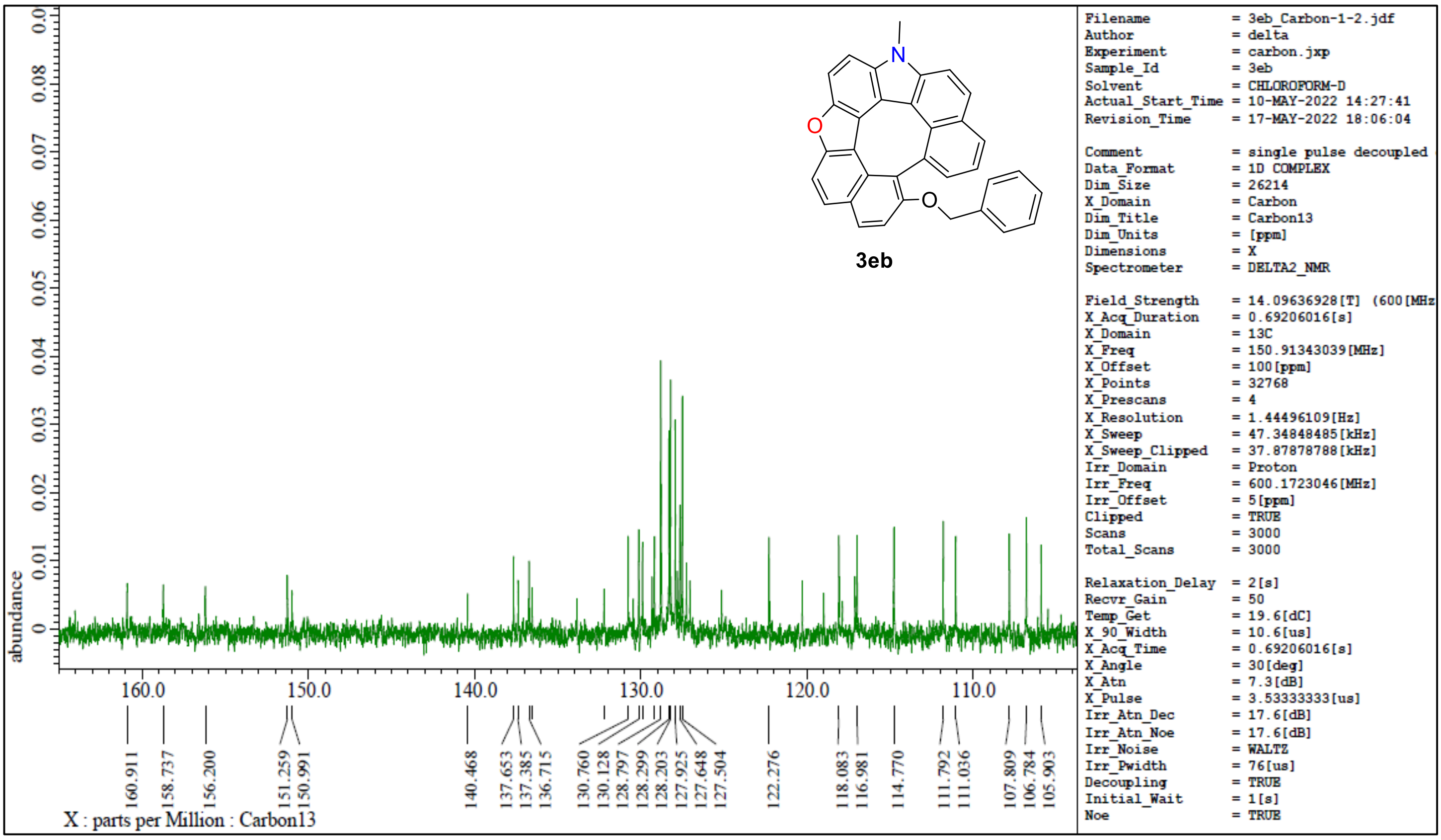

Compound **3eb** (^{13}C NMR, 150 MHz, $CDCl_3$).

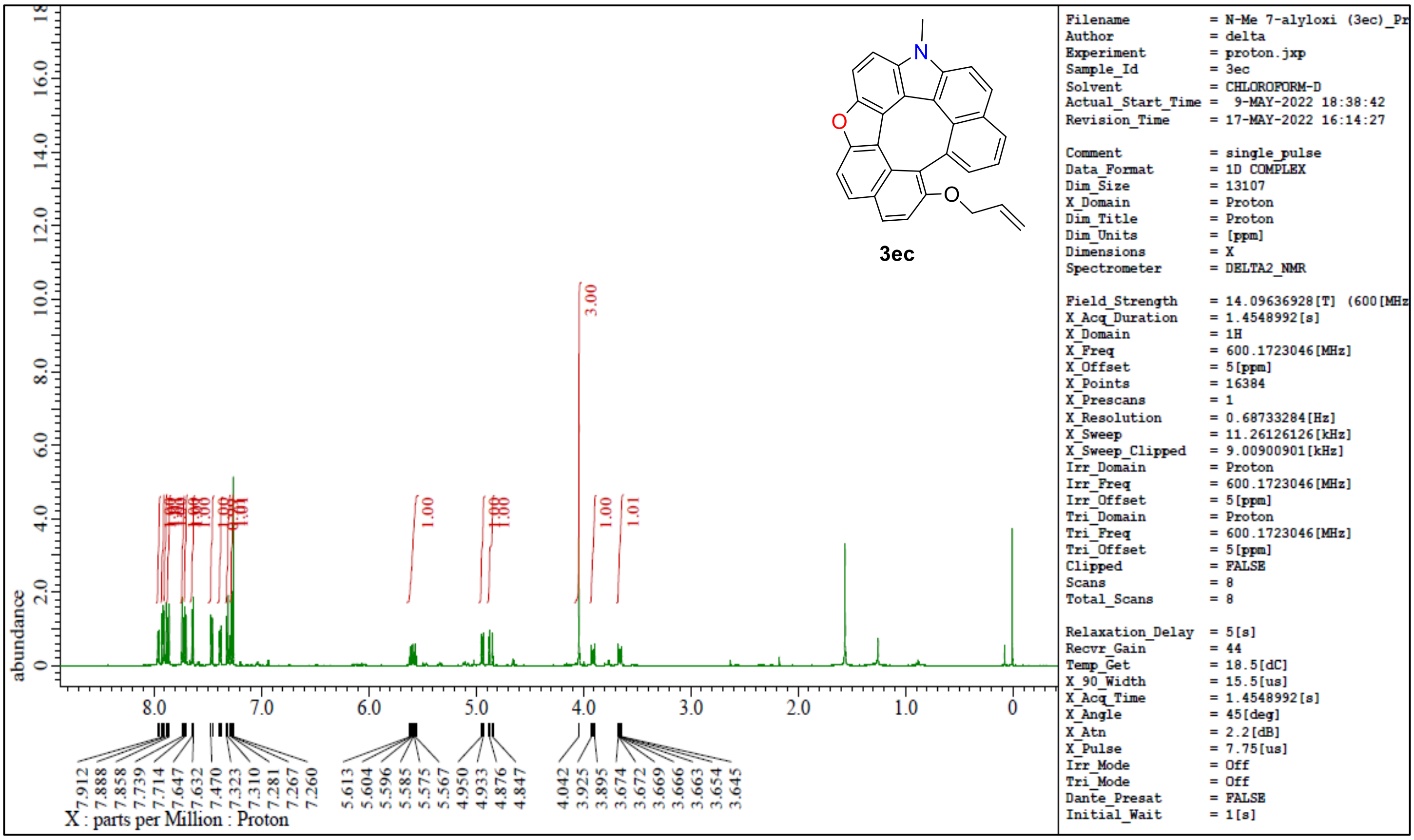

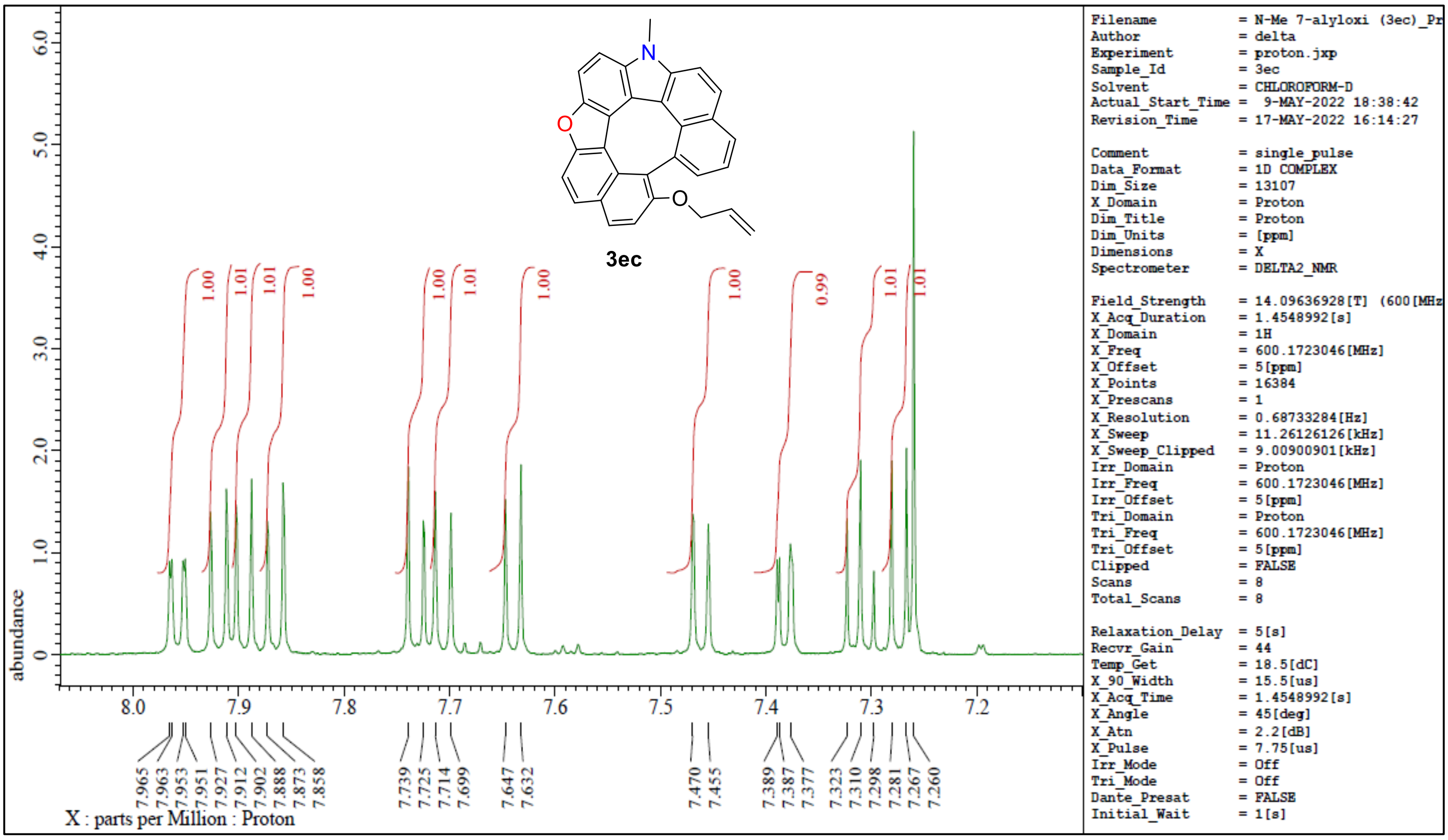

Compound **3ec** (^{1}H NMR, 600 MHz, $CDCl_3$).

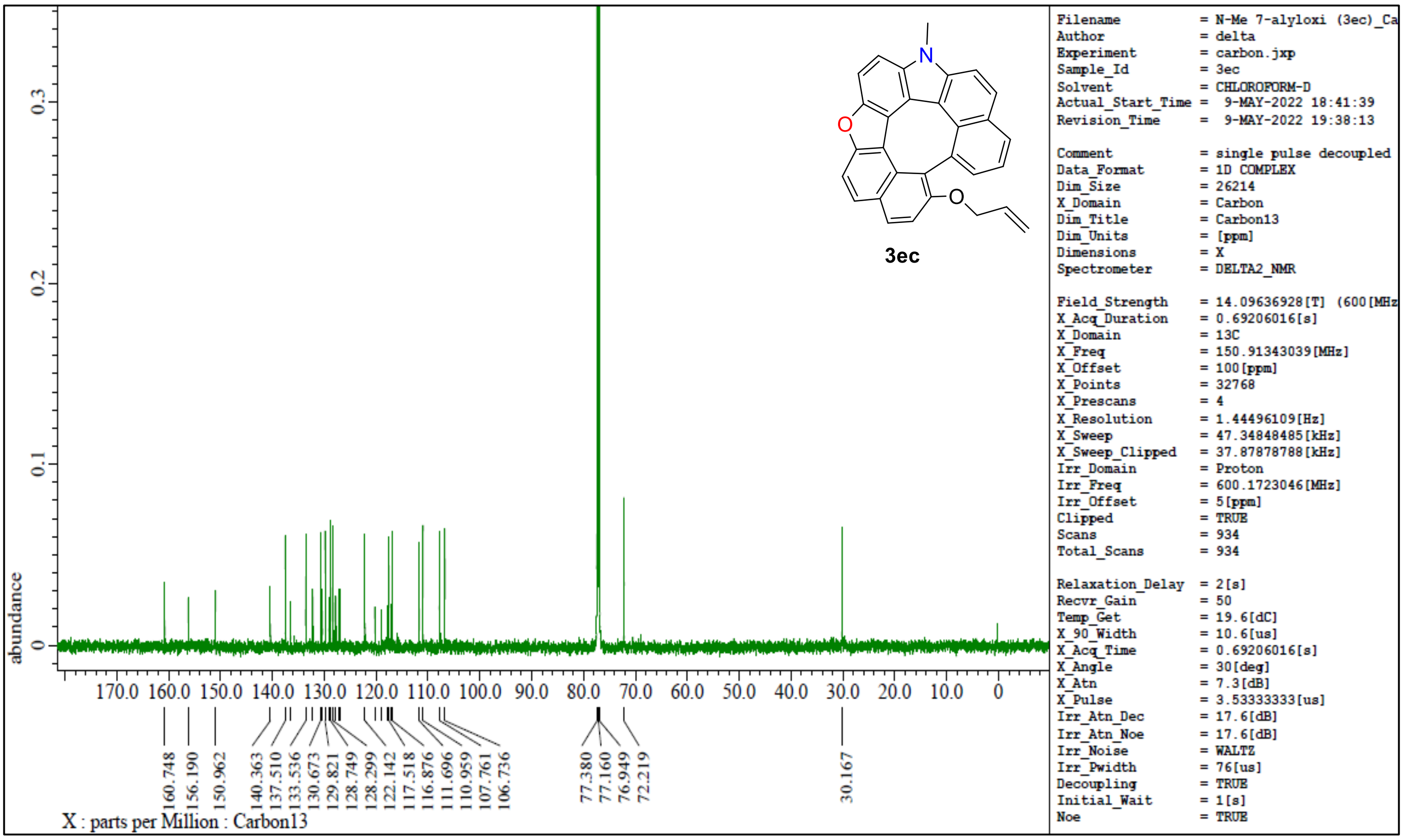

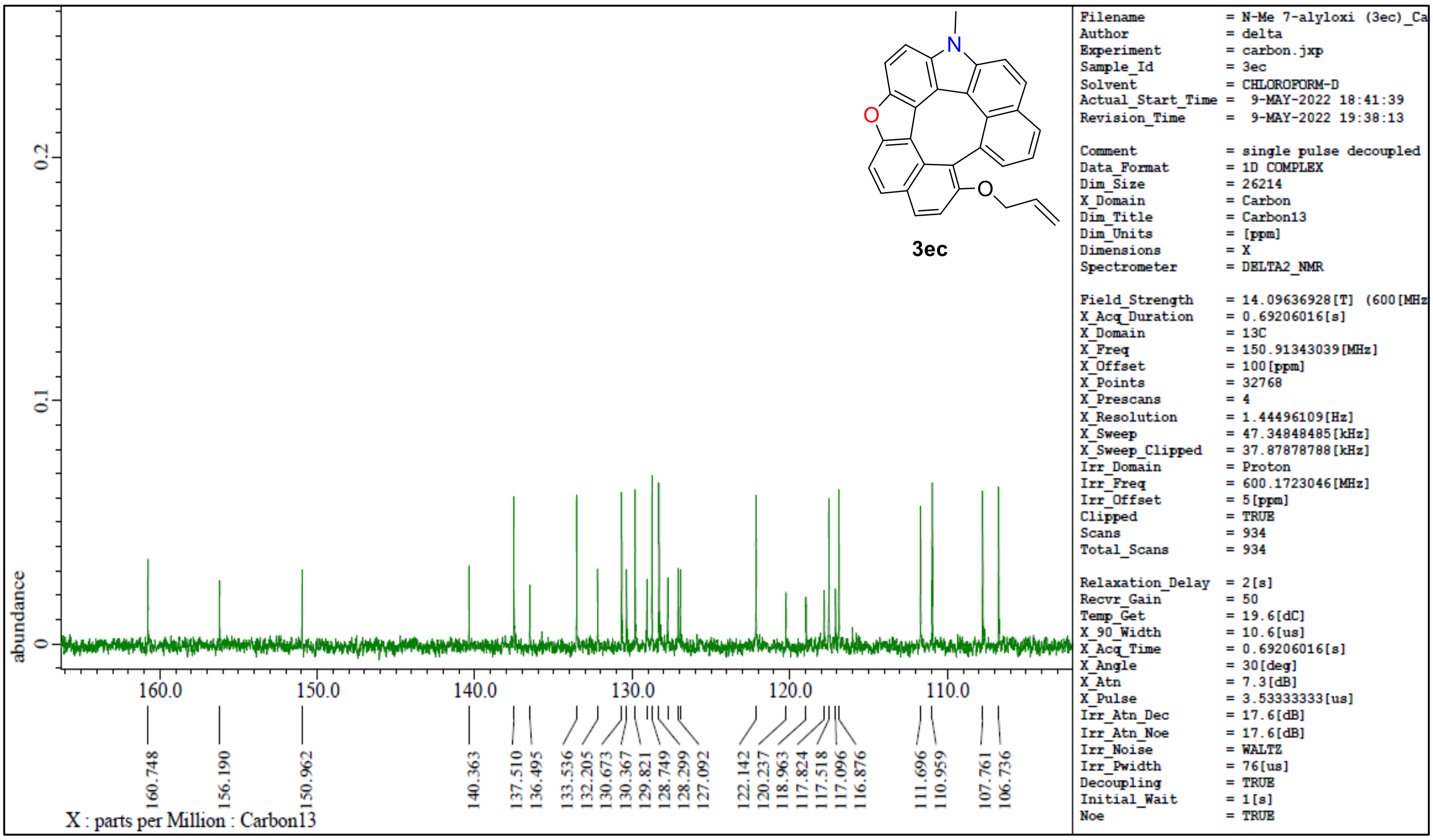

Compound **3ec** (^{13}C NMR, 150 MHz, $CDCl_3$).

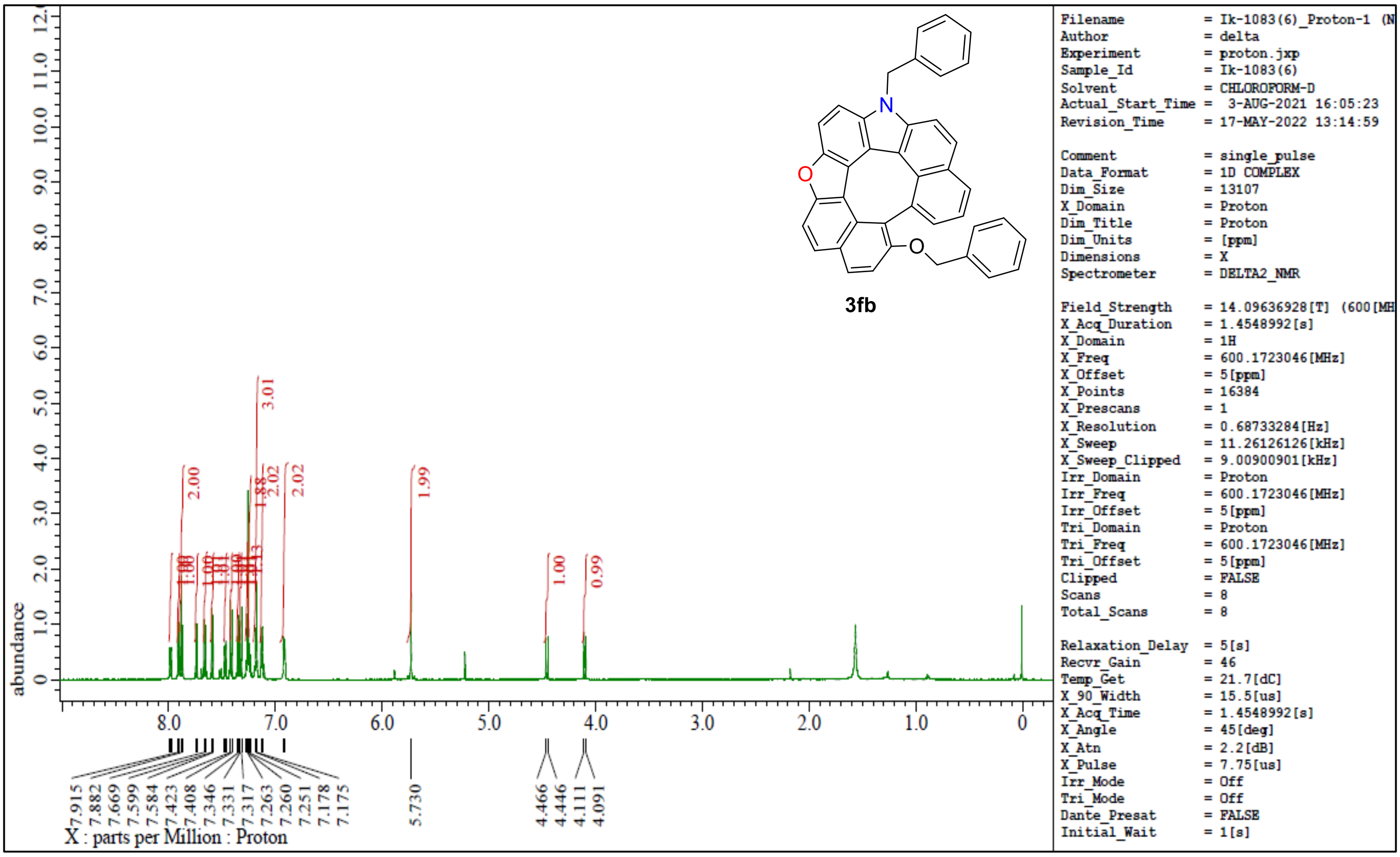

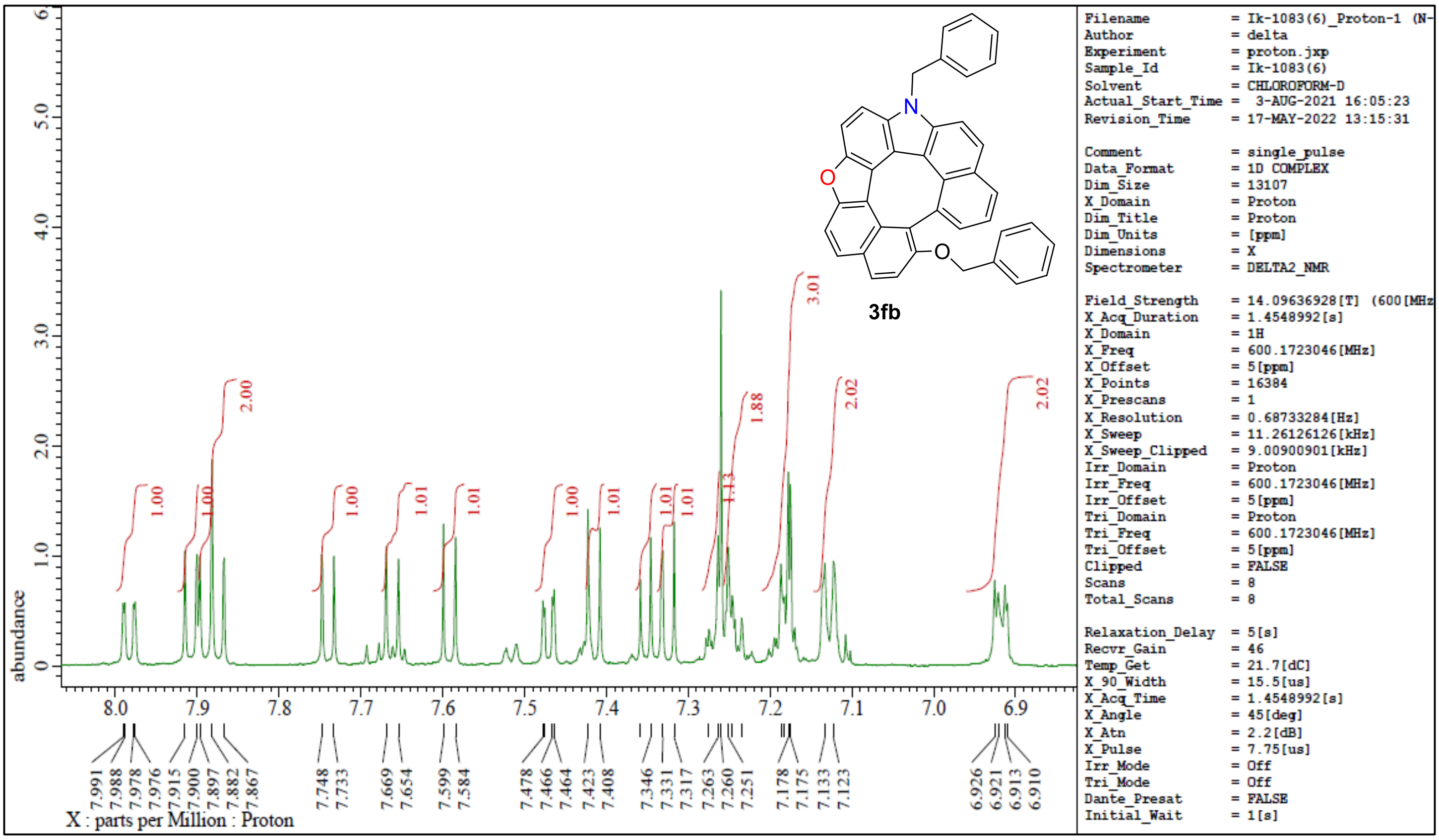

Compound **3fb** (^{1}H NMR, 600 MHz, $CDCl_3$).

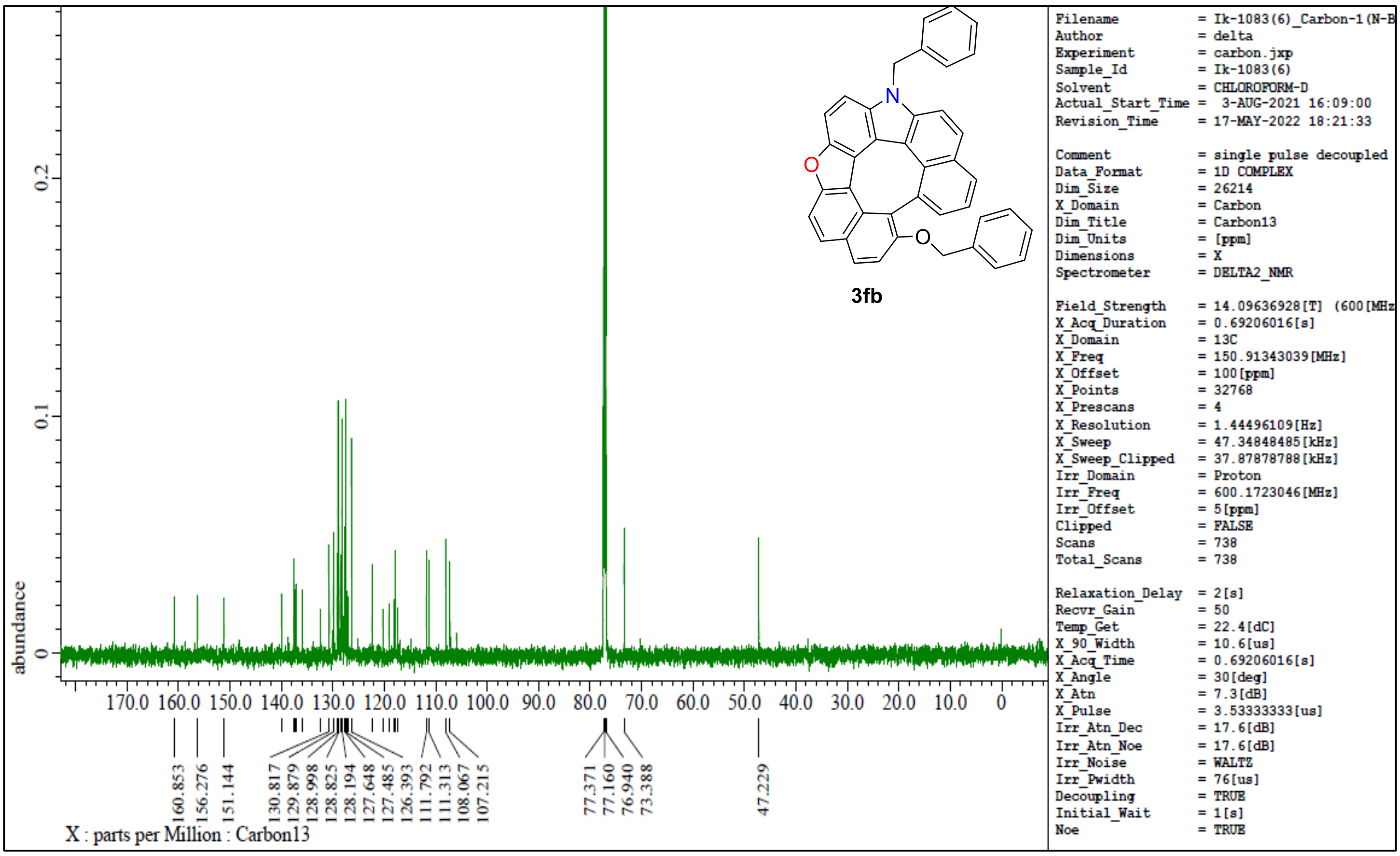

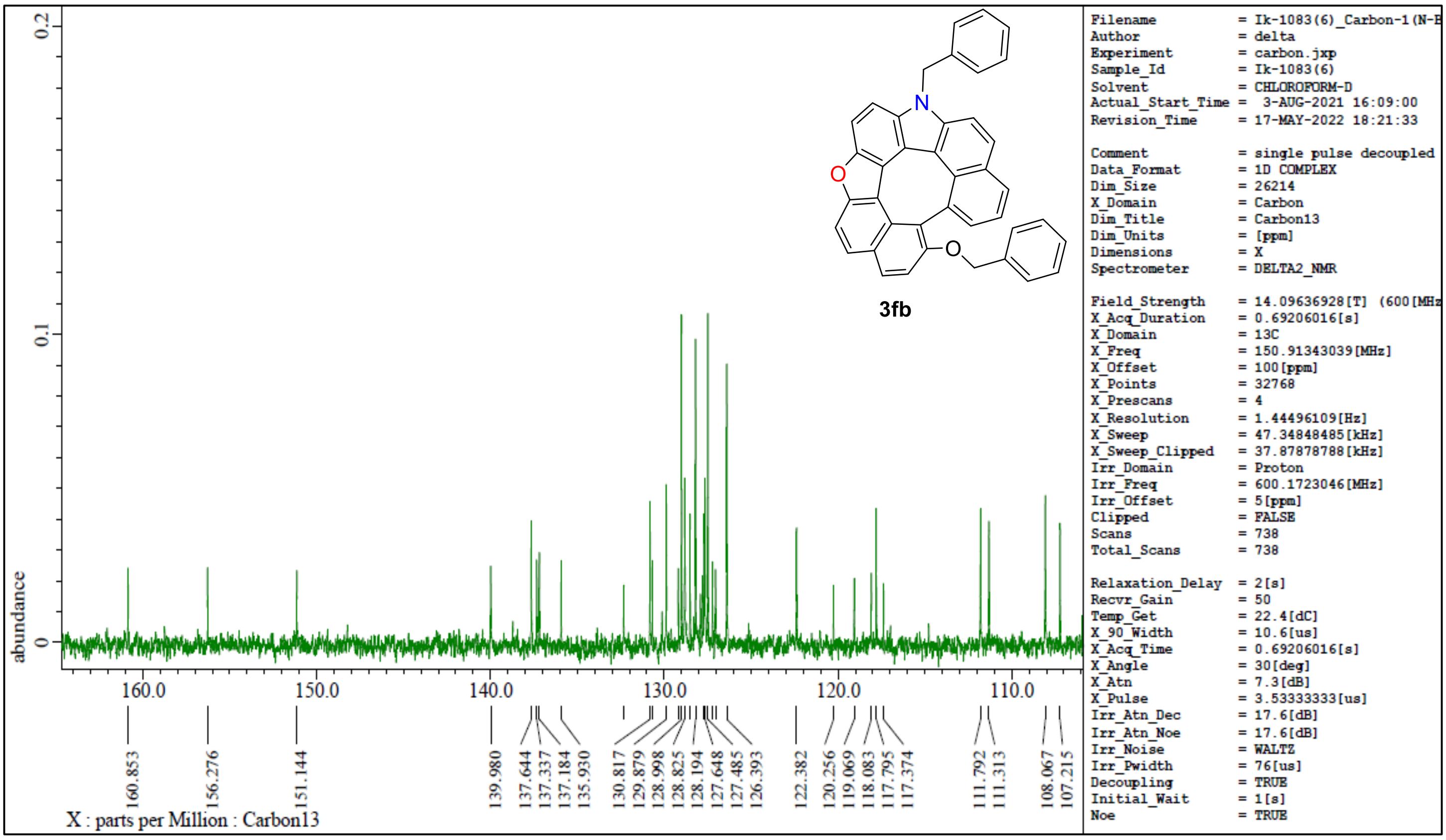

Compound **3fb** (^{13}C NMR, 150 MHz, $CDCl_3$).

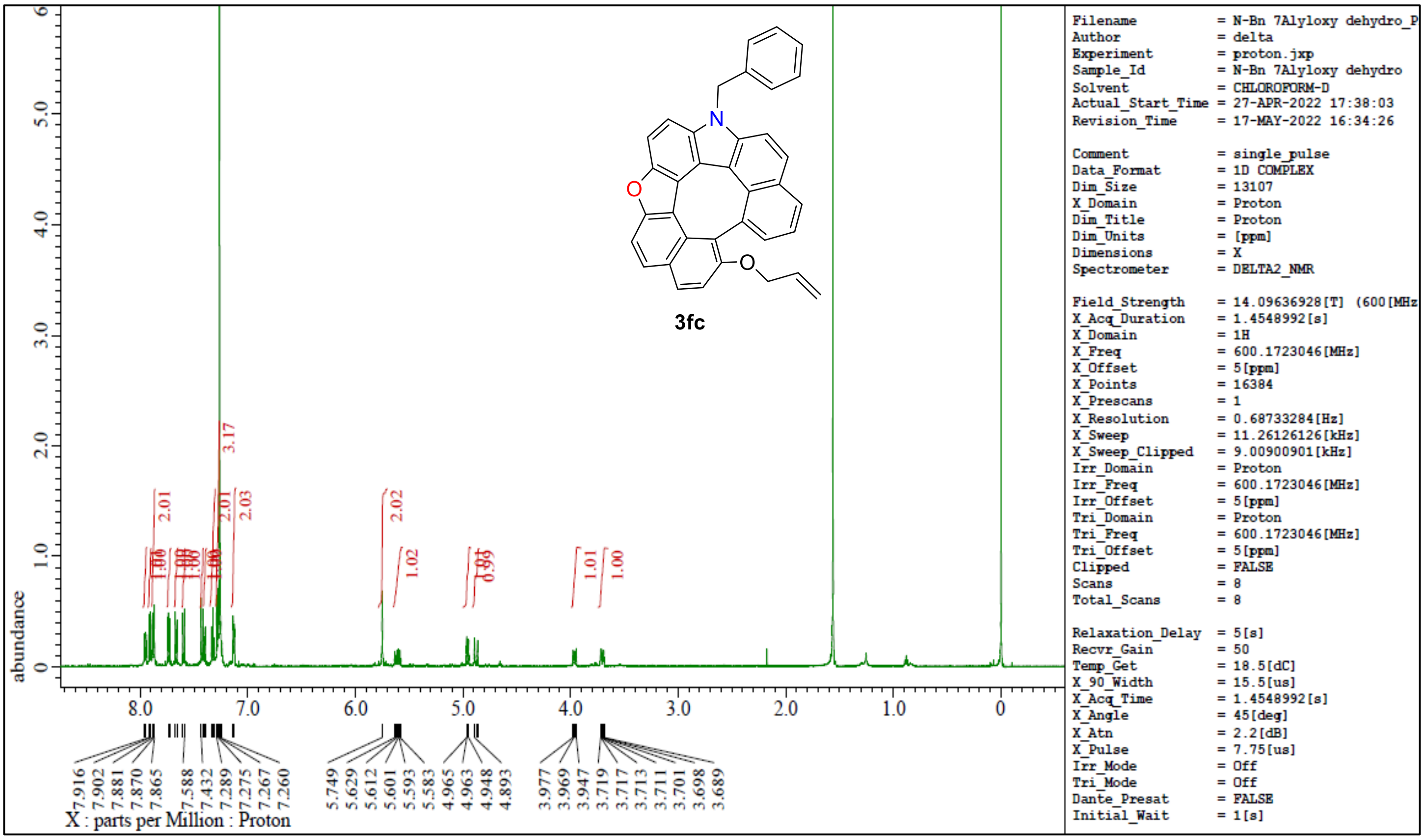

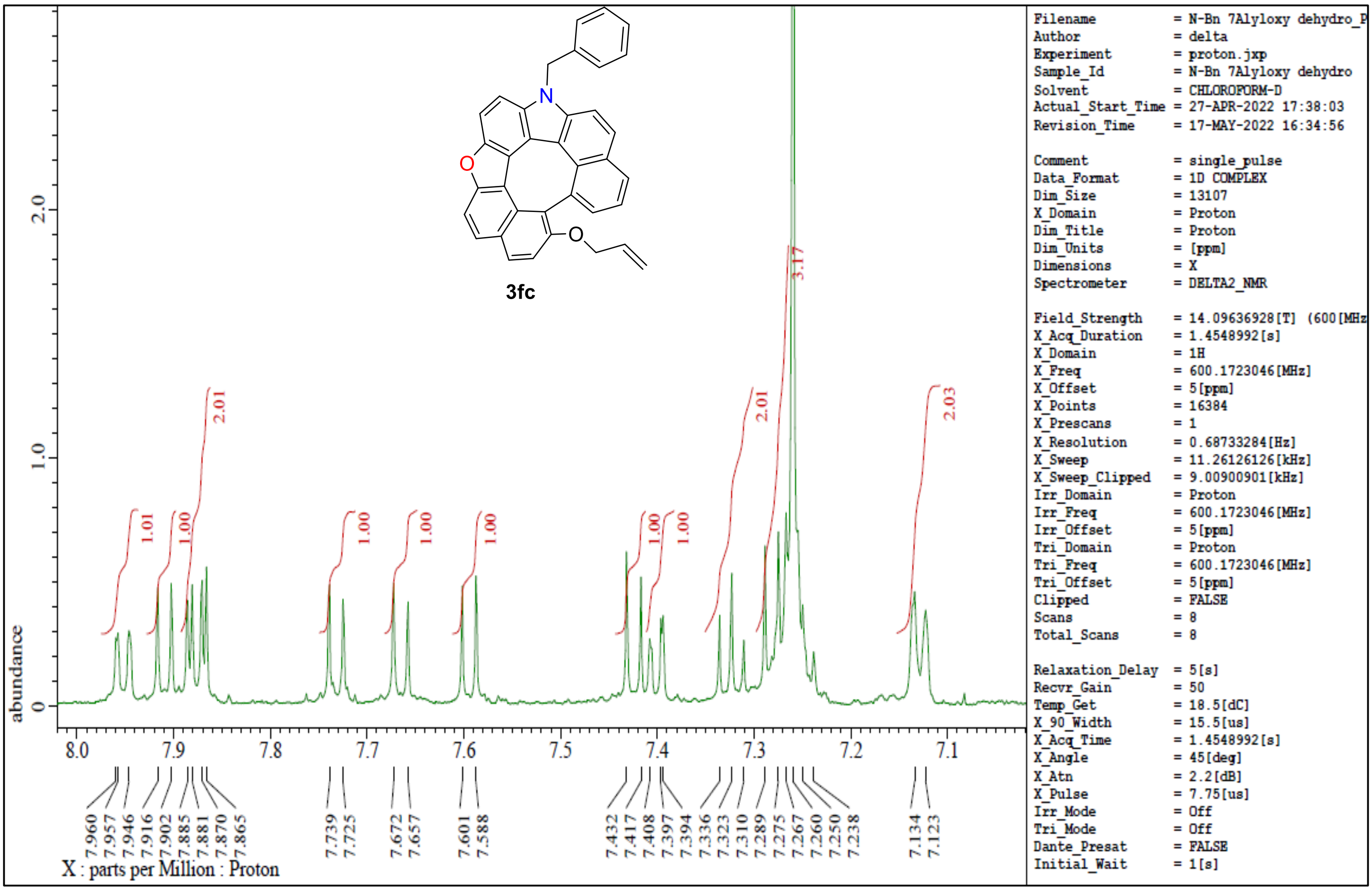

Compound **3fc** (^1H NMR, 600 MHz, $CDCl_3$).

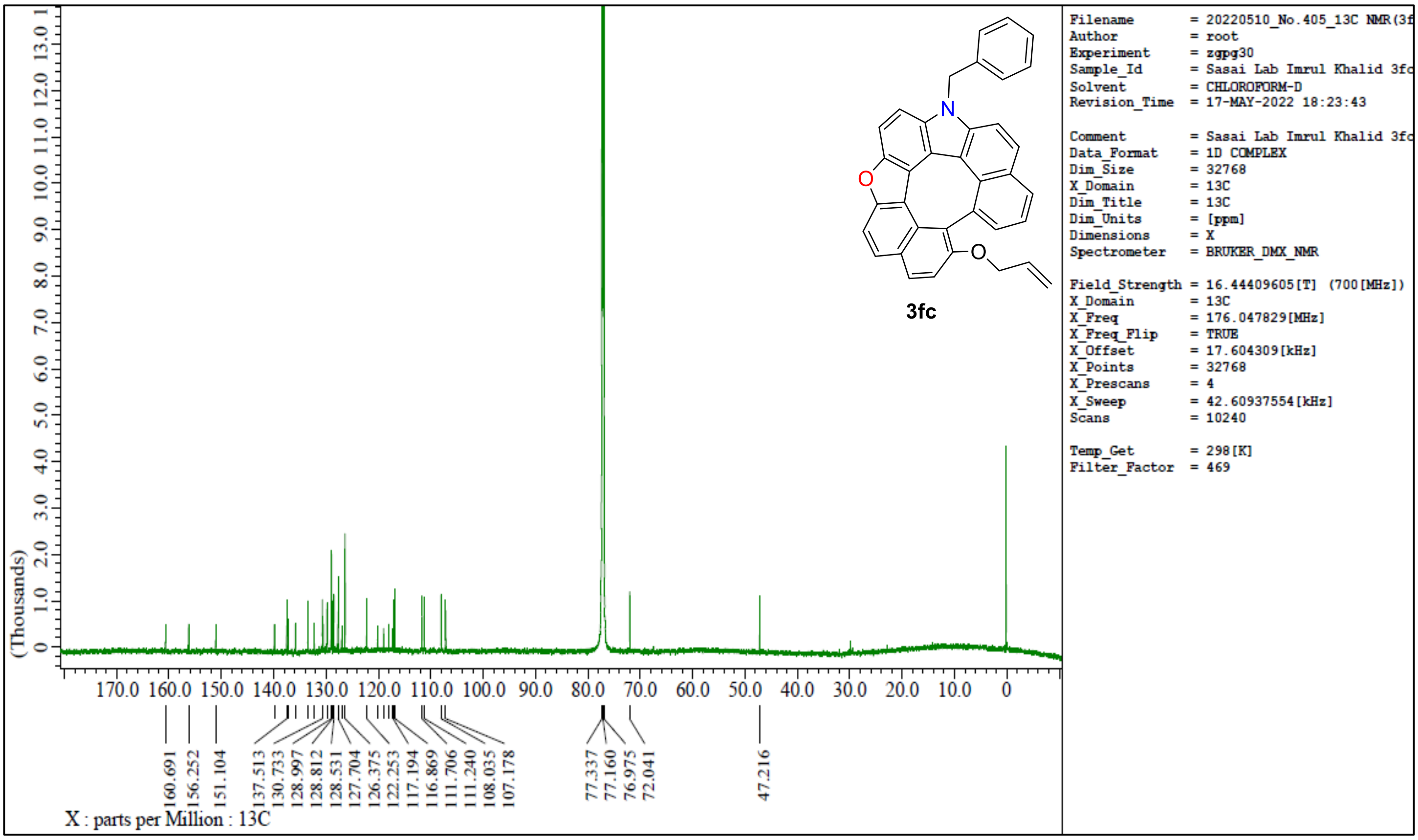

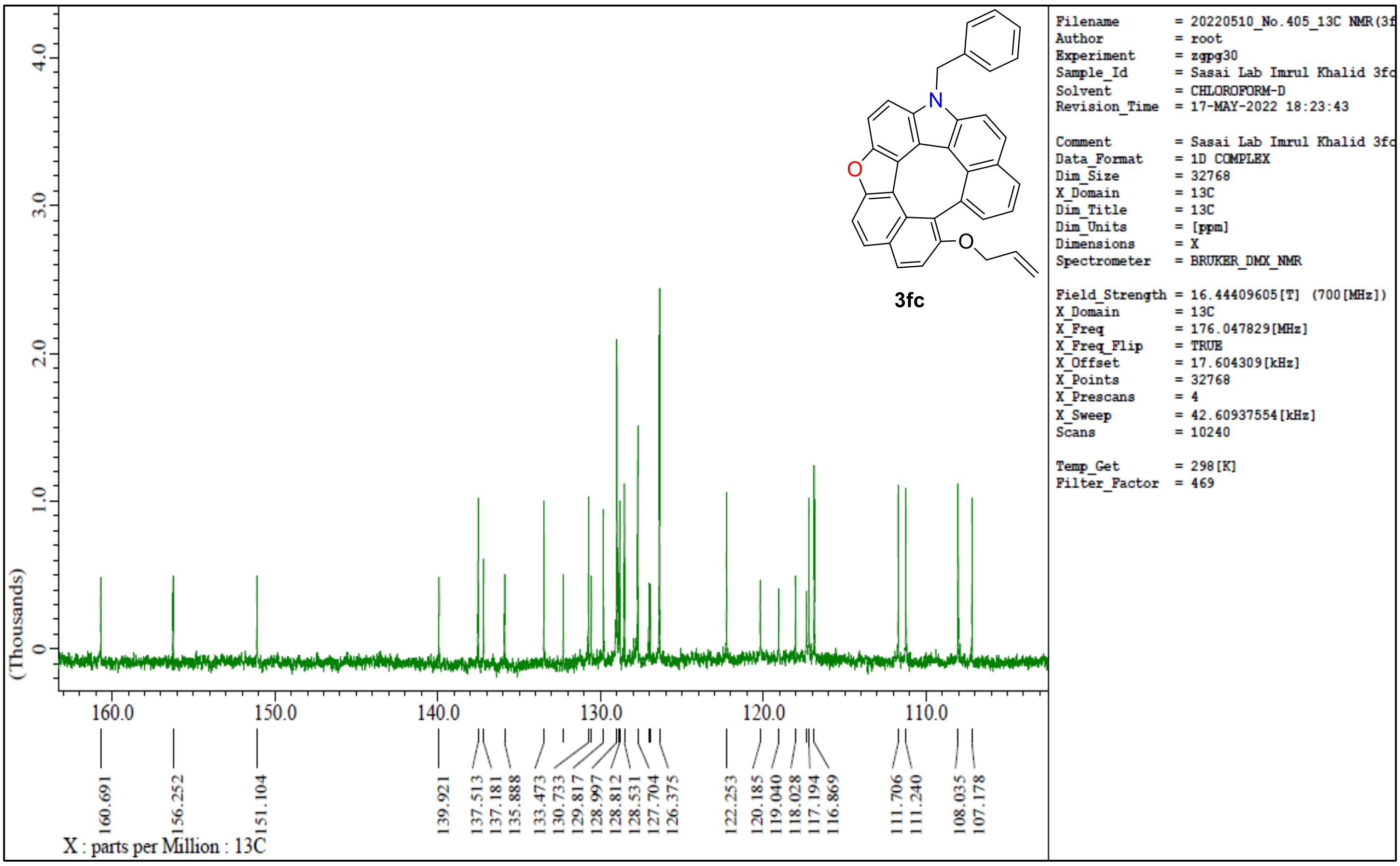

Compound **3fc** (^{13}C NMR, 175 MHz, $CDCl_3$).

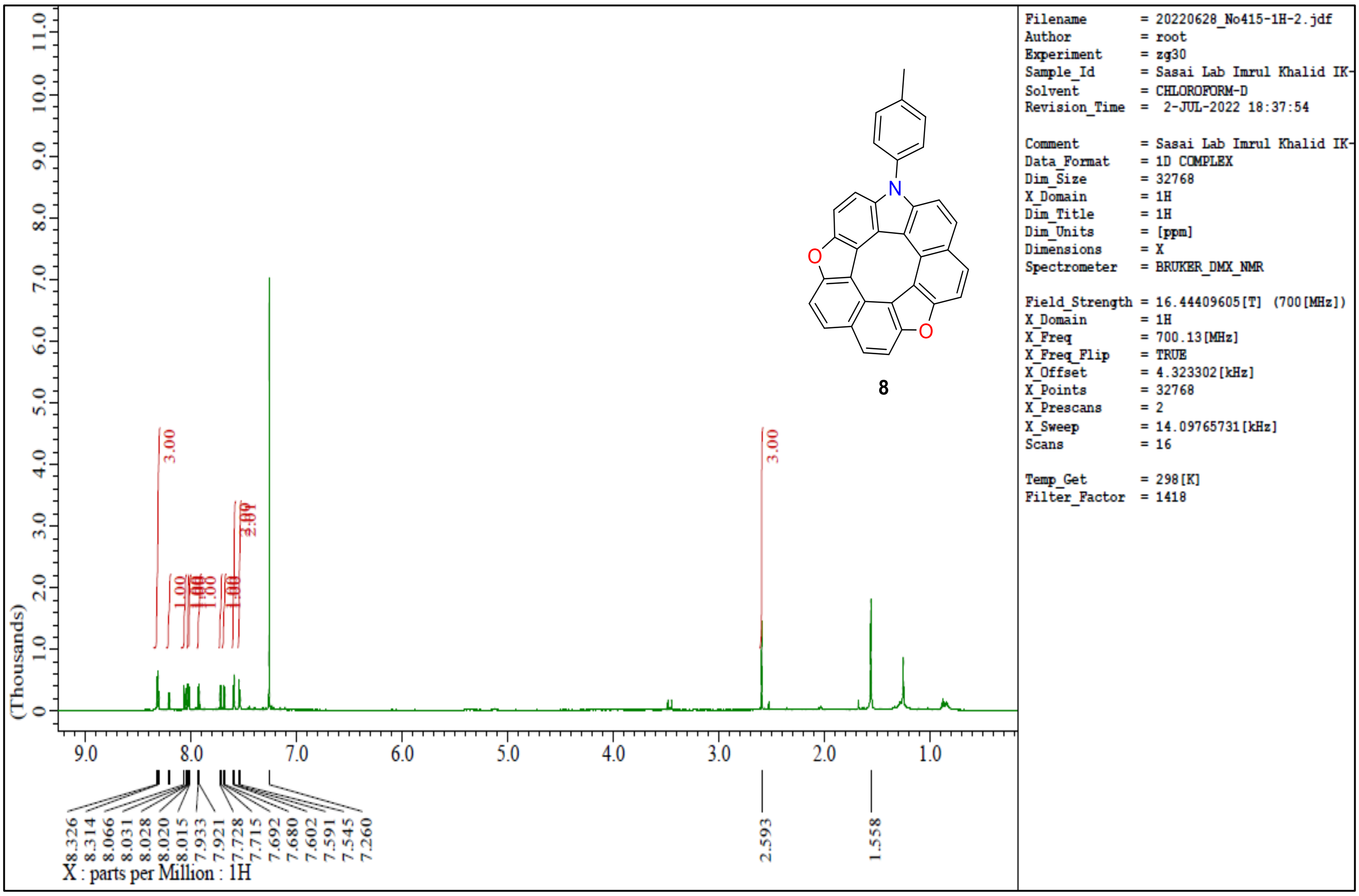

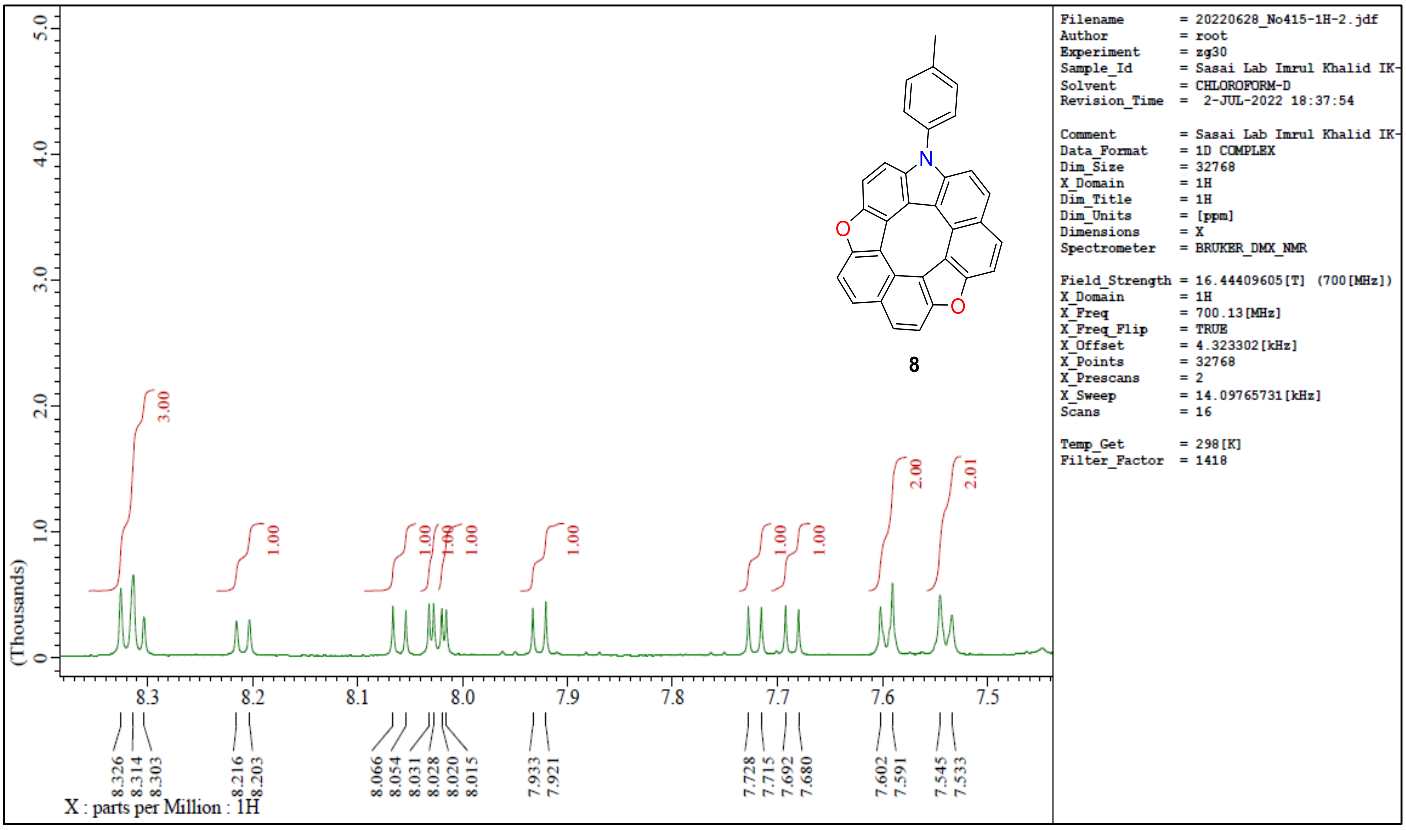

Compound **8** (^{1}H NMR, 700 MHz, $CDCl_3$).

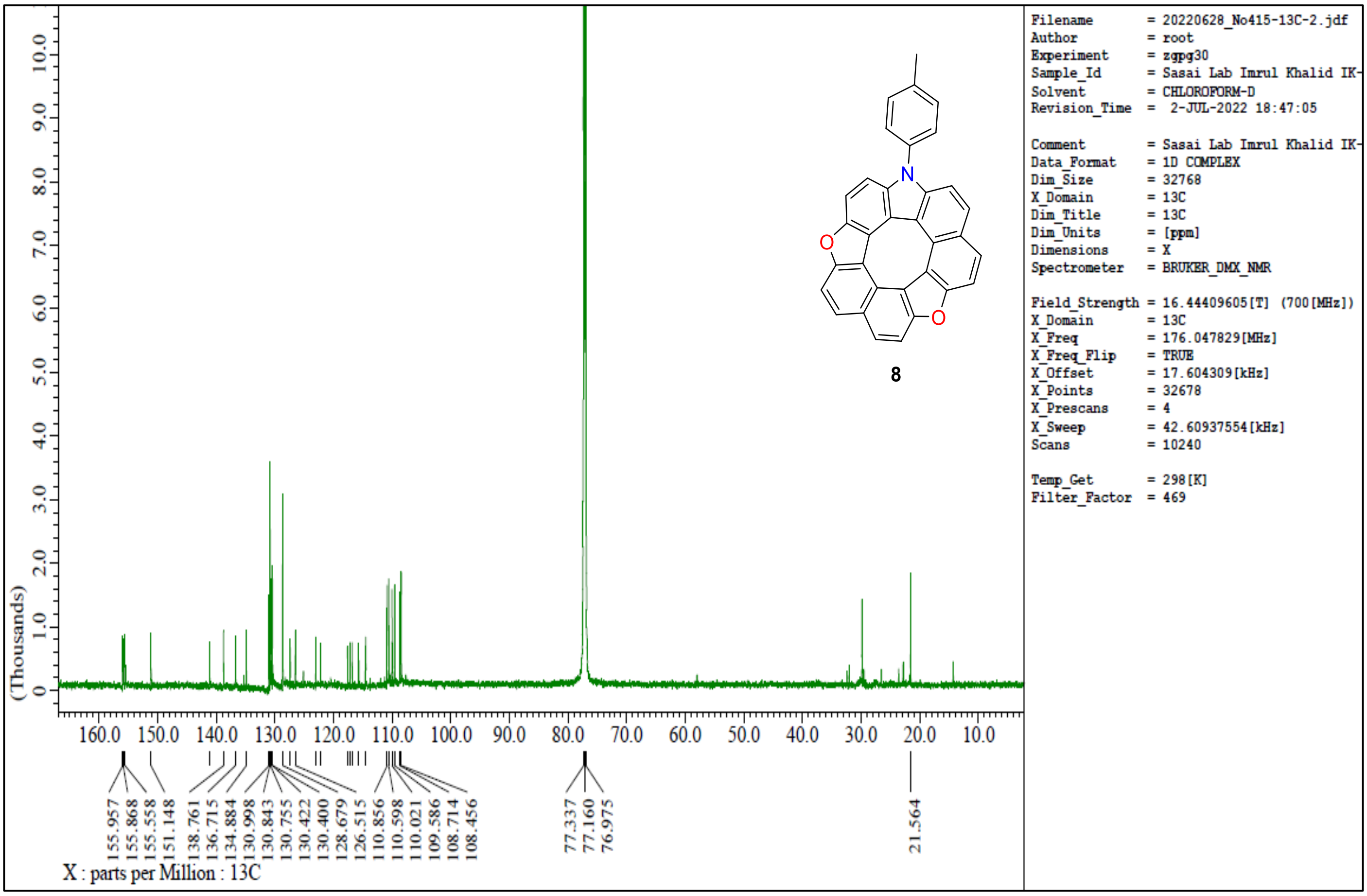

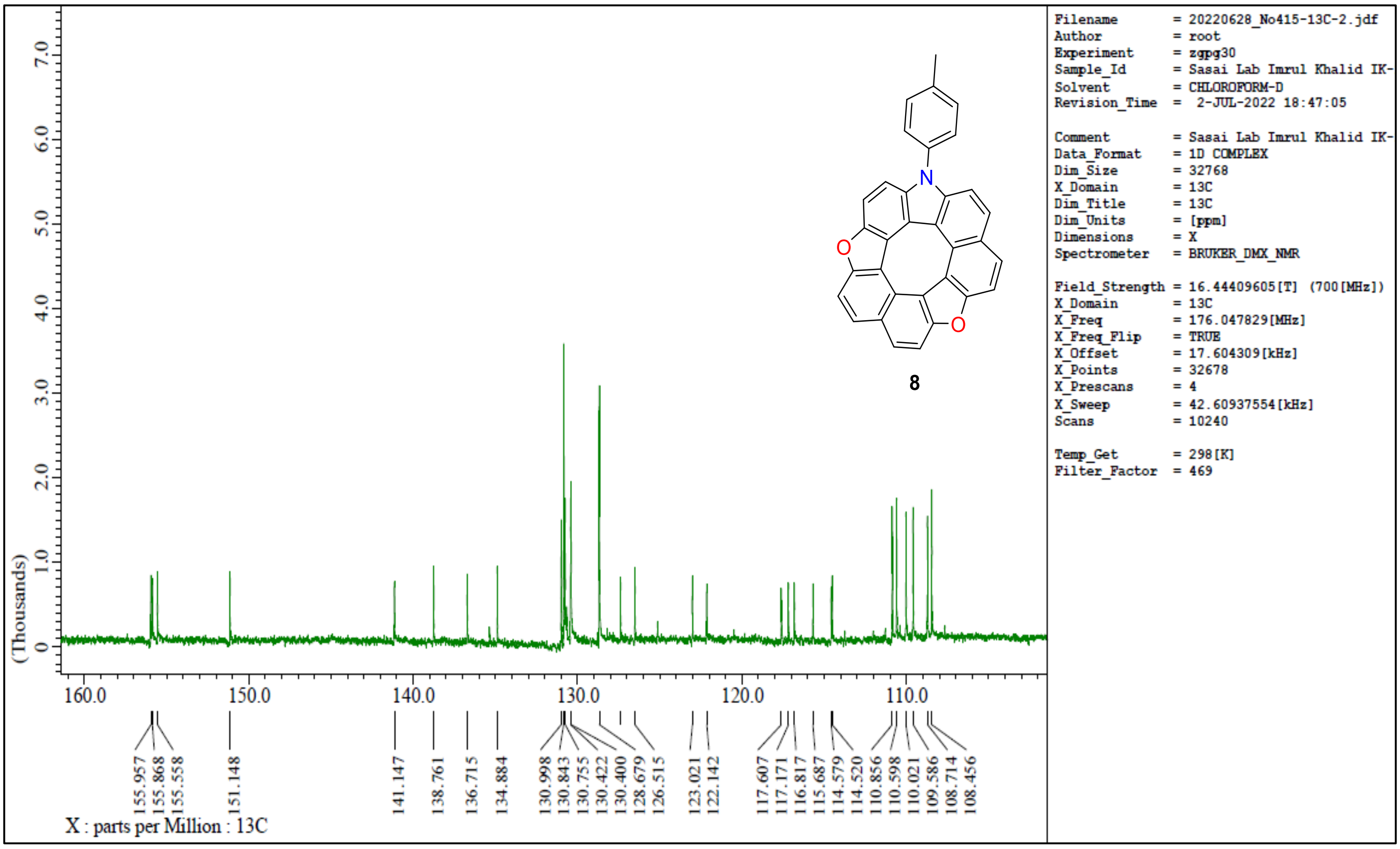

Compound **8** (^{13}C NMR, 175 MHz, $CDCl_3$).

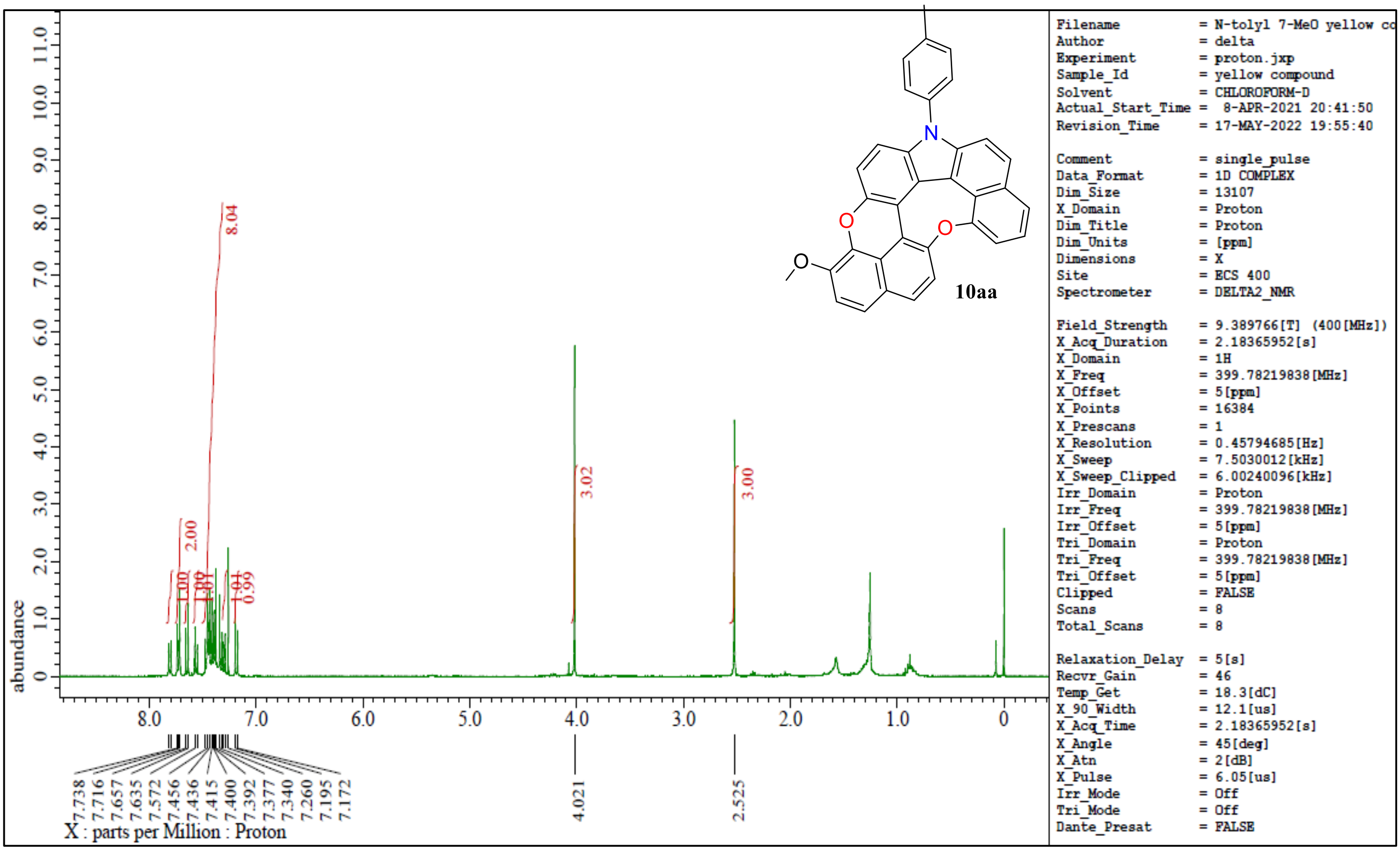

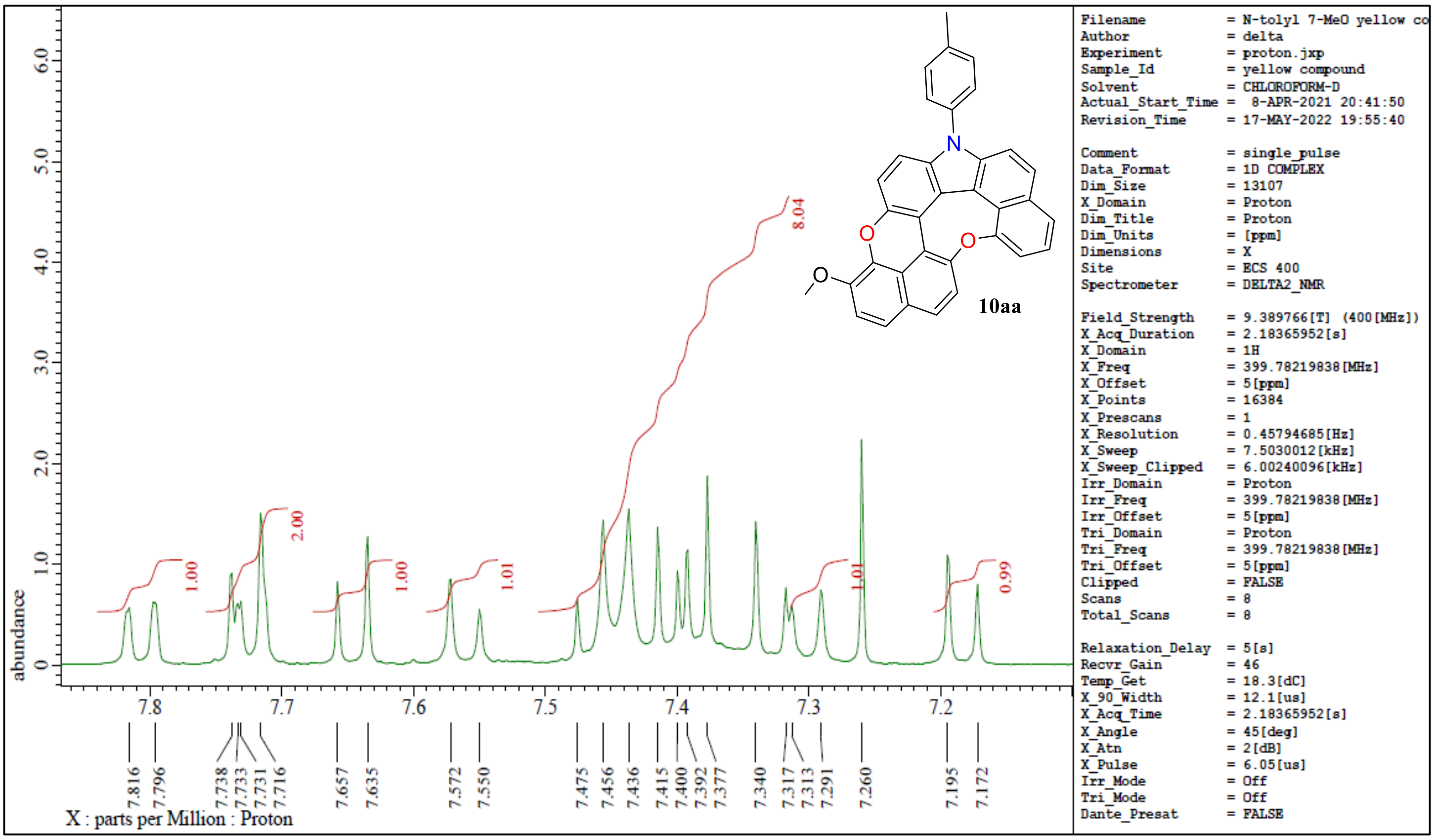

Compound **10aa** (^{1}H NMR, 400 MHz, $CDCl_3$).

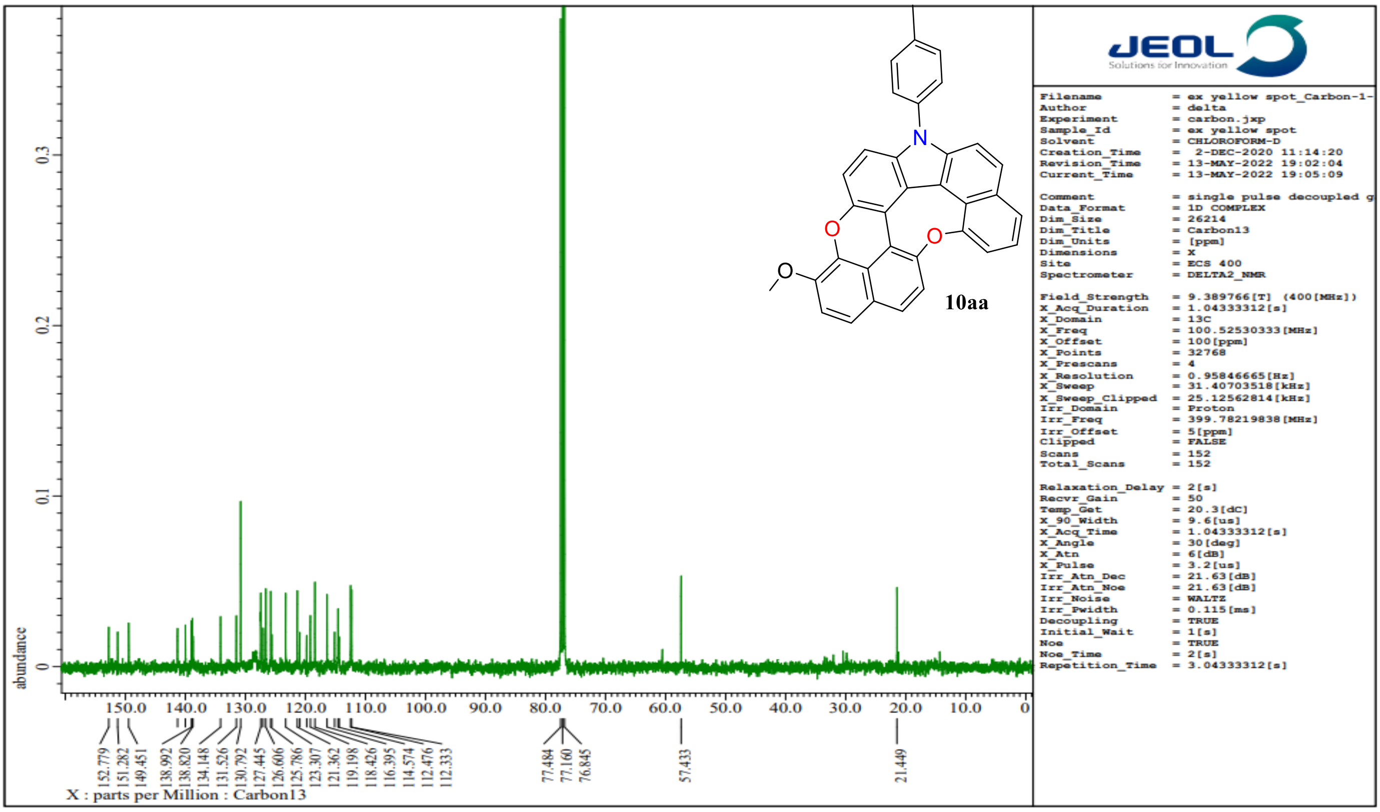

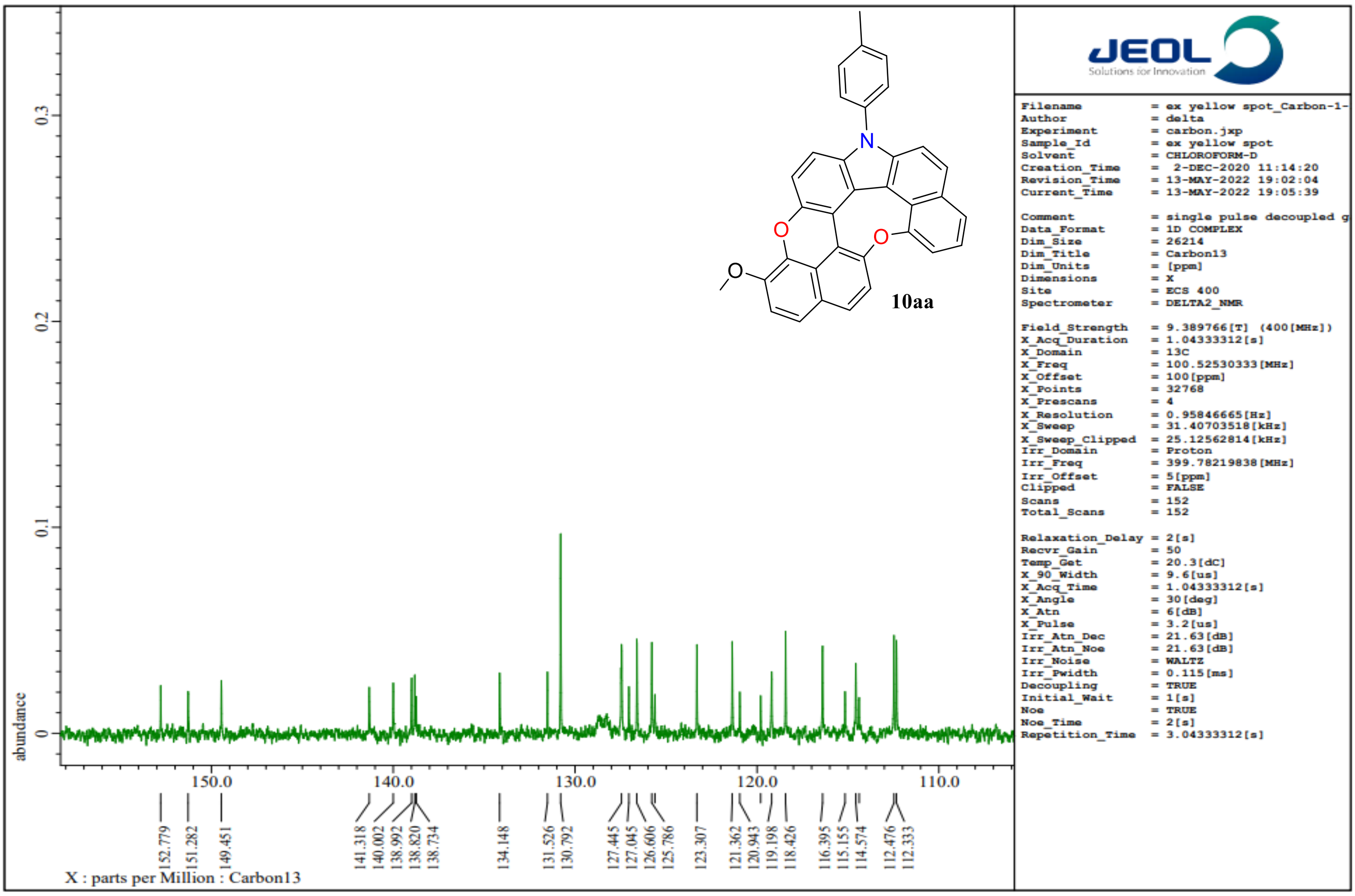

Compound **10aa** (^{13}C NMR, 100 MHz, $CDCl_3$).
