# Supplementary material for: Electrochemical synthesis of heterodehydro[7]helicenes
Source: Commun Chem. 2022 Dec 3;5:166. doi: 10.1038/s42004-022-00780-7 (PMC9814689; doi:10.1038/s42004-022-00780-7)
Supplement: Supplementary file 1 — Takizawa_PR File [file 42004_2022_780_MOESM1_ESM.pdf]

nature portfolio

Peer Review File

Electrochemical Synthesis of Heterodehydro[7]helicenes

Open Access This file is licensed under a Creative Commons Attribution 4.0 International License, which permits use, sharing, adaptation, distribution and reproduction in any medium or format, as long as you give appropriate credit to the original author(s) and the source, provide a link to the Creative Commons license, and indicate if changes were made. In the cases where the authors are anonymous, such as is the case for the reports of anonymous peer reviewers, author attribution should be to 'Anonymous Referee' followed by a clear attribution to the source work. The images or other third party material in this file are included in the article's Creative Commons license, unless indicated otherwise in a credit line to the material. If material is not included in the article's Creative Commons license and your intended use is not permitted by statutory regulation or exceeds the permitted use, you will need to obtain permission directly from the copyright holder. To view a copy of this license, visit http://creativecommons.org/licenses/by/4.0/.

This manuscript has been previously reviewed at another Nature Portfolio journal. This document only contains reviewer comments and rebuttal letters for versions considered at *Communications Chemistry.*

Reviewers' comments:

Reviewer #2 (Remarks to the Author):

The manuscript by H. Sasai, S. Takizawa et al entitled Electrochemical Synthesis of Heterodehydro[7]helicenes describes chemistry of dehydrohelicenes in terms of their efficient electrochemical synthesis, derivatization of the azaoxa-dehydro[7]helicene, and their chiroptical properties (OR, CD, CPL).

Since I have already reviewed and commented on the manuscript, I can state that as far as my reservations about the manuscript are concerned, the authors have either explained them or made appropriate corrections.

Reviewer #3 (Remarks to the Author):

The authors have revised the manuscript according to the previous reviewer's comments and now I feel this manuscript is suitable for publication in Communications Chemistry. However, there are still some errors and unclear points as shown below.

1) Figure S16, caption; this is not (M) 3ab. The assignments (P or M) should be mentioned suitably. In addition, please delete unmeaningful characters "CD" and "CPL" in the figure.

2) Why only (M) 3ab shows CPL? Because CPL spectra are generally weak, the authors should confirm both CPL and determine the g value.

3) As indicated by other reviewers, I also feel strange to see the positive delta S value for 3aa. In such a case, the authors may insert an additional dataset by measuring the racemization rate at another different temperature (110 or 120 °C).

Response to referees' comments

Reviewer #3:

The authors have revised the manuscript according to the previous reviewer's comments and now I feel this manuscript is suitable for publication in Communications Chemistry. However, there are still some errors and unclear points as shown below.

1) Figure S16, caption; this is not (M) **3ab**. The assignments (P or M) should be mentioned suitably. In addition, please delete the unmeaningful characters "CD" and "CPL" in the figure.

Our response: We appreciate your comments. We revised these parts according to reviewer 3's comment and added (*P* or *M*) instead of (*M*), we also removed "CD" and "CPL" from the figure.

2) Why only (M) 3ab shows CPL? Because CPL spectra are generally weak, the authors should confirm both CPL and determine the g value.

Our response: We again measured the g value of both enantiomers (*M*) and (*P*) **3ab** shown in Figure **S17** in the supporting information. We also measured the CD of both enantiomers and updated Figure **S16**. The *glum* was confirmed to be (2.4×10^{-3}), with little improvement compared to the previous value (2.2×10^{-3}). We have revised all related parts in the manuscript and supporting information and updated them accordingly.

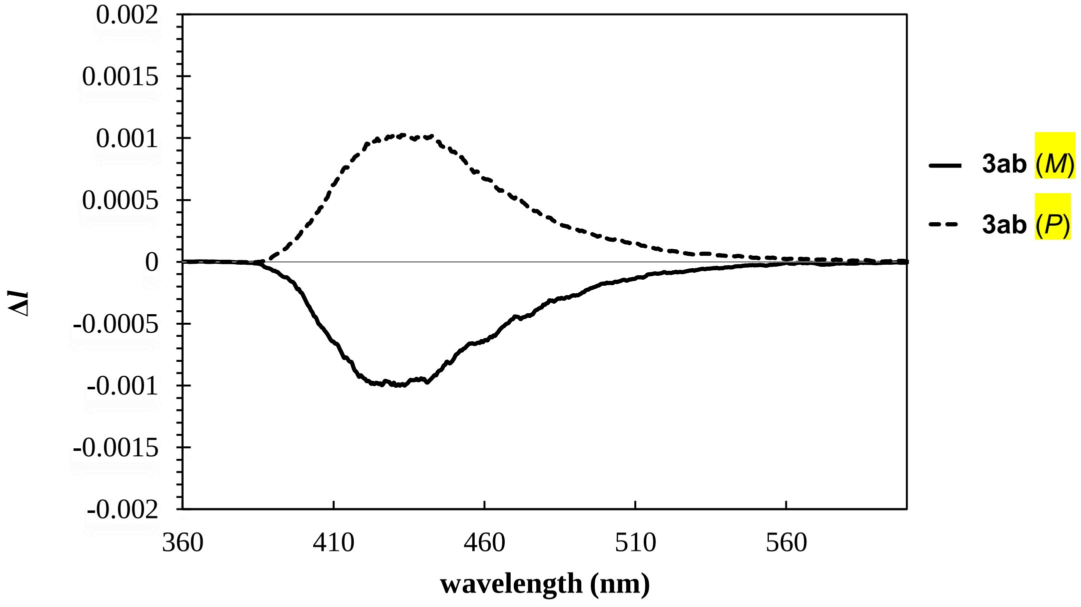

CPL spectra of compound (*P* or *M*) **3ab** in $CHCl_3$ (20 µM solutions). g_{lum} = (2.4×10^{-3})

3) As indicated by other reviewers, I also feel strange to see the positive delta S value for 3aa. In such a case, the authors may insert an additional dataset by measuring the racemization rate at another different temperature (110 or 120 °C).

Our response: We screened three additional temperatures (110 °C, 120 °C, and 140 °C) based on reviewer 3's comment. After considering these three new points, we recalculated the thermodynamic parameters again and resolved this issue for compound **3aa** and found ΔS = -2.69 J. mol^{-1} K^{-1} (See supplementary Note 2, Figure S10). According to this, the $t_{1/2}$ of racemization was changed to 9.5×10^3 years, we modified all related parts in the manuscript and SI accordingly.

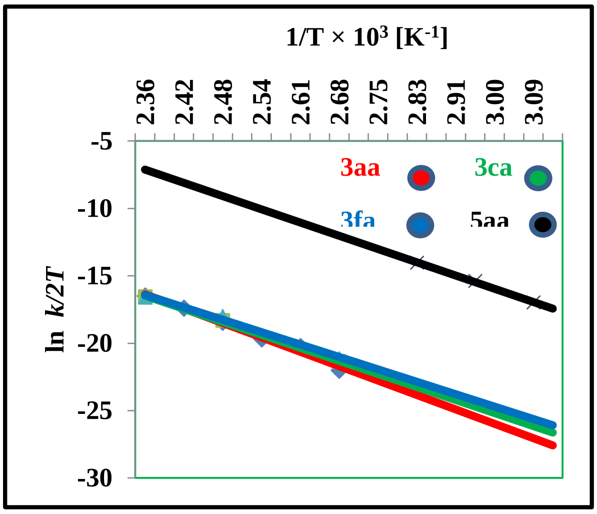

	3aa	5aa
R^2	0.999	0.998
Slope	-1.6880 x 10^4	-1.1222 x 10^4
intercept	23.4356	17.7663
k/s^{-1} at 25°C	2.32 X 10^{-12}	1.40 X 10^{-6}

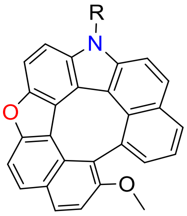

3

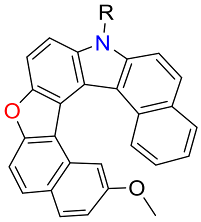

5aa

	R	ΔH≠	ΔS≠	ΔG≠	$t_{1/2}$ at 25 °C
3aa	*p*-tolyl	140.345 kJ.mol^{-1}	-2.688 J.$mol^{-1}K^{-1}$	141.4 ± 0.05 kJ.mol^{-1}	9.5 x 10^3 years
3ca	*p*-ClC_6H_4	126.404 kJ.mol^{-1}	-36.313 J.$mol^{-1}K^{-1}$	140.9 ± 0.91 kJ.mol^{-1}	1.9 x 10^3 years
3fa	Bn	120.706 kJ.mol^{-1}	-49.174 J.$mol^{-1}K^{-1}$	140.4 ± 1.24 kJ.mol^{-1}	9.2 x 10^2 years
5aa	*p*-tolyl	93.304 kJ.mol^{-1}	-49.826 J.$mol^{-1}K^{-1}$	110.2 ± 0.75 kJ.mol^{-1}	5.7 days

In the manuscript and supporting information, all revised parts have been highlighted in yellow.

Hiroaki Sasai *Shinobu Takizawa*

SANKEN, Osaka University, Mihogaoka, Ibaraki, Osaka 567-0047, JAPAN

Phone: (+81) 6-6879-8467 FAX:(+81) 6-6879-8469

e-mail: sasai@sanken.osaka-u.ac.jp taki@sanken.osaka-u.ac.jp

REVIEWERS' COMMENTS:

Reviewer #3 (Remarks to the Author):

All the points raised in the previous version have been revised in the current manuscript. Thus I recommend publication as it is.
